# Wide transcriptional investigation unravel novel insights of the on-tree maturation and postharvest ripening of ‘Abate Fetel’ pear fruit

**DOI:** 10.1038/s41438-018-0115-1

**Published:** 2019-03-01

**Authors:** Nicola Busatto, Brian Farneti, Alice Tadiello, Vicky Oberkofler, Antonio Cellini, Franco Biasioli, Massimo Delledonne, Alessandro Cestaro, Christos Noutsos, Fabrizio Costa

**Affiliations:** 1 Department of Genomics and Biology of Fruit Crops, Research and Innovation Centre, Fondazione Edmund Mach (FEM), Via E. Mach 1, 38010 San Michele all’Adige, Italy; 2 Department of Biology, University of Padova, Via G. Colombo 3, 35121 Padova, Italy; 3 Institute for Biochemistry and Biology, Karl-Liebknecht-Str. 24-25, 14476 Potsdam, Germany; 4 Department of Agricultural and Food Science, University of Bologna, Via Fanin 46, 40127 Bologna, Italy; 5 Department of Food Quality and Nutrition, Research and Innovation Centre, Fondazione Edmund Mach (FEM), Via E. Mach 1, 38010 San Michele all’Adige, Italy; 6 Department of Biotecnology, University of Verona, Strada le Grazie 15, Cà Vignal 1, 37134 Verona, Italy; 7 Unit of Computational Biology, Research and Innovation Centre, Fondazione Edmund Mach (FEM), Via E. Mach 1, 38010 San Michele all’Adige, Italy; 8 Biology Department, SUNY College at Old Westbury, Old Westbury, NY 11568 USA

**Keywords:** Plant sciences, Gene expression

## Abstract

To decipher the transcriptomic regulation of the on-tree fruit maturation in pear cv. ‘Abate Fetel’, a RNA-seq transcription analysis identified 8939 genes differentially expressed across four harvesting stages. These genes were grouped into 11 SOTA clusters based on their transcriptional pattern, of which three included genes upregulated while the other four were represented by downregulated genes. Fruit ripening was furthermore investigated after 1 month of postharvest cold storage. The most important variation in fruit firmness, production of ethylene and volatile organic compounds were observed after 5 days of shelf-life at room temperature following cold storage. The role of ethylene in controlling the ripening of ‘Abate Fetel’ pears was furthermore investigated through the application of 1-methylcyclopropene, which efficiently delayed the progression of ripening by reducing fruit softening and repressing both ethylene and volatile production. The physiological response of the interference at the ethylene receptor level was moreover unraveled investigating the expression pattern of 12 candidate genes, initially selected to validate the RNA-seq profile. This analysis confirmed the effective role of the ethylene competitor in downregulating the expression of cell wall (PG) and ethylene-related genes (ACS, ACO, ERS1, and ERS2), as well as inducing one element involved in the auxin signaling pathway (Aux/IAA), highlighting a possible cross-talk between these two hormones. The expression patterns of these six elements suggest their use as molecular toolkit to monitor at molecular level the progression of the fruit on-tree maturation and postharvest ripening.

## Pears: Genes linked to fruit maturation and ripening

Numerous genes, including many involved in the production and signaling of key growth hormones, regulate the on-tree maturation and postharvest ripening of pear fruit. Fabrizio Costa from the Edmund Mach Foundation in San Michele All’adige, Italy, and colleagues identified nearly 9,000 genes that were differentially expressed across four maturation stages of the Abate Fetel cultivar of European pear (*Pyrus communis*). From this long list of genes, the researchers selected 12 involved in hormone signaling, cell wall metabolism or aromatic volatile compound production, and evaluated their expression pattern with and without application of a chemical added to delay ripening during postharvest storage. Six of the genes, five of which controlled the activity of the hormones auxin and ethylene, showed differential expression, and could be informative as molecular biomarkers for improving fruit quality in pears.

## Introduction

Rosaceae is one of the most important botanical families in horticultural fruit crops. The genus *Pyrus* is distinguished by four main species cultivated for fruit production, such as *P. bretschneiders* Rehd, *P. ussuriensis* Maxim, *P. pyrifolia* (Burm), and *P. communis* L.^1^. While the first three species are mainly grown and cultivated in Asia, the last species, *P. communis*, represents the taxonomic group to which most of the varieties cultivated in America, Europe, Africa, and Australia belong. Italy is the fourth world-wide production country of pear, after China, Argentina and United States (with 701,928 tons in 2016; FAOSTAT), with cv. ‘Abate Fetel’ as the most important and widely grown variety. Pear fruit are particularly appreciated by consumers for their quality features, such as unique shape, buttery texture, juiciness, sweetness, and aroma^2^. All these features are the result of a physiological program that taking place from the on-tree fruit maturation, progress through the postharvest fruit ripening. In pear, as well as in other horticultural crop fruit species, such as apple, peach, or tomato, the fruit ripening is of climacteric type^3,4^, meaning that most of the modification making the fruit edible and appreciated are triggered and coordinated by the hormone ethylene^4–6^.

Unlike other climacteric fruits, the pear fruit is distinguished by a varying degree of ripening. The completion of ripening can rely, in fact, on other important factors, such as the requirement of a chilling period or ethylene treatment^7^. ‘Abate Fetel’, like other pear varieties, needs a period of exposure to cold temperature in order to ripe^8^. Therefore, pear fruit reach the optimal quality for fresh consumption only during the postharvest phase^2^. Among the several strategies that can be applied to stimulate and induce the onset of ripening (such as cold temperature storage, preharvest treatment with plant growth regulators or exogenous application of ethylene^7^), the identification of the best harvest time certainly deserves a particular attention. In pear fruit cv. ‘d’Anjou’, the dessert type of quality (buttery texture, juiciness, optimal flavor, and aromatic blend) changes, indeed, according to the harvest stage, which is finally recognized as a crucial aspect to ensure an optimal fruit quality. This is particularly true in orchard of medium-large size, where fruit are harvested in a wide time window. The tight relationship between harvest time and fruit quality has, moreover, an important role also in the postharvest management. The postharvest storage of fruits is an essential strategy allowing the constant market availability of fresh fruit, guaranteeing, at the same time, an extended commercialization and marketability. During storage, however, the fruit quality reached at the end of the on-tree maturity and ripening needs to be maintained. This result can be achieved, for instance, slowing down the progression of fruit ripening, avoiding excessive fruit softening and general quality drop-off. To this end, the cold storage of fruit can be performed together with the application of 1-methylcyclopropene (1-MCP), a molecule known to interfere with the ethylene receptor system^9^. 1-MCP is largely used to control the climacteric ripening in many fruit species, including pear. Gramashi and colleagues^10^, for instance, applied 1-MCP to improve the storability of the pear fruit cv. ‘Spadona’, while Ekman et al.^11^ studied the effect of different concentration of this ethylene competitor in the ripening of pear fruit cv. ‘Bartlett’.

Due to the importance of these processes in ensuring the economical success of the fruit production, a more detailed investigation about the physiological mechanisms controlling the fruit maturation and ripening in pear fruit is compelling. To date, the employment of next-generation sequencing techniques offers the possibility to investigate at a genome-wide scale the transcriptomic variation across samples/stages, identifying the important genes involved in the regulation of the processes of interest. This strategy has been already employed in pear fruit. Ou and colleagues^12^ identified a set of differentially expressed genes associated to dwarfing, while Nham et al.^13^ employed this technique to study the fruit maturity during the fruit development in pear cv. ‘Bartlett’.

In this work, we aimed to unravel the transcriptional variation occurring during the on-tree maturation and postharvest ripening in fruit of cv. ‘Abate Fetel’ employing both, RNA-seq and candidate gene approaches. The role of ethylene in controlling the postharvest fruit ripening was moreover investigated with the use of 1-MCP, which highlighted the existence of a possible hormonal cross-talk with auxin.

## Results

### Progression of the on-tree fruit maturity and postharvest ripening of pear

The ripening stage of the fruit collected at each harvesting date (H1–H4) was non-destructively assessed with the use of a DA-meter (Supplementary Fig. S1). Within each stage, a continuous distribution of the index of absorbance (I_AD_) was observed, and the most representative categories of fruit with a similar I_AD_ were grouped into homogeneous classes of fruit. The I_AD_ value for H1 was 2.1–2.0, while for the other three stages the I_AD_ was 2.0–1.9 for H2, 1.9–1.8 for H3 and 1.8–1.7 for H4, respectively. The selected fruit from each group represented the biological material used to perform the subsequent physical/metabolic and transcriptomic analyses. The DA-meter device was also employed to monitor the progression of the postharvest ripening (Supplementary Fig. S2). The content of chlorophyll showed a decreasing pattern over the four stages at T0 (harvest), as well as after one month of cold storage (T1). However, the most important variation in ripening was observed after 5 days of shelf-life ripening at room temperature following cold storage (T1 + 5_ctrl_), with a loss of I_AD_ spanning from 1.6 for H2 to 3.06 fold of change for H4. The use of the DA-meter also weighted the effect of 1-MCP in delaying the general ripening of pear fruit. Fruit at T1 + 5_1-MCP_ showed, in fact, an I_AD_ value closer to harvest (T0), with a fold change ranging from 1 (no change) to 1.09. The decreased I_AD_ observed between T1_ctrl_ and T1 + 5_ctrl_ was moreover supported by the increased production of ethylene measured on the same fruit (Supplementary Fig. S3), with a fold change ranging between 1.78 for H3 to 5.47 for H2. The application of 1-MCP turned down completely the ethylene production, slowing down the general fruit ripening as evidenced by the I_AD_ value.

### Assessment of the fruit quality parameters

The quality of pear fruit was assessed based on important parameters, such as fruit texture, aroma, and the soluble solids content. The two devices employed in this study to assess the fruit firmness provided consistent data (Supplementary Fig. S4). The fruit firmness linearly decreased from 6.27 Kg cm^−2^–17.7 N (in T0_H1) to 3.69 Kg cm^−2^–10.64 N (in T1_H4). As observed already for the dynamics of the I_AD_ and the ethylene pattern, the 5 days of shelf-life ripening at room temperature boosted the loss of firmness, bringing the value down to 0.93 Kg cm^−2^– 2.1 N for the T1 + 5_ctrl_ sample in H1. Also in this case, the application of 1-MCP greatly reduced the disassembly of the cell wall, showing values closer to T0, with a fold change spanning from 1 to 1.12. With the texture analyzer, the fruit firmness was moreover assessed in two zones of the fruit, located at a distal (close to the area assessed with the digital penetrometer) and at an internal portion of the cortex (close to the seed cavity), respectively. While the fruit texture assessed with the TAXT*plus* in the external area of the fruit showed a linear correlation with the penetrometer, the value of the maximum force assessed in the internal part resulted higher (Supplementary Fig. S5).

**Fig. 1:**
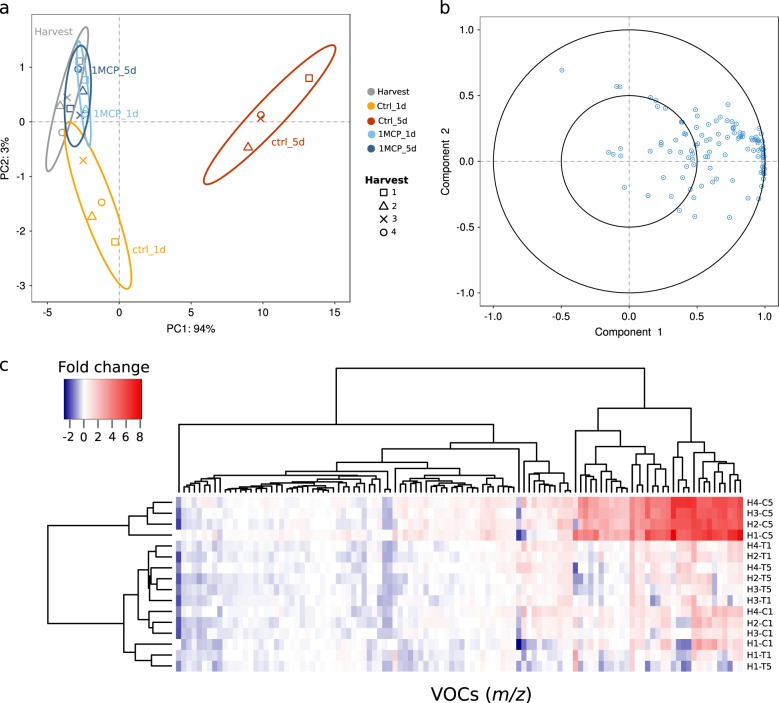
PCA analysis of the volatilome in pear fruit. In panel **a** is depicted the distribution of the pear samples at different on-tree maturity and postharvest ripening stages, together with the 1-MCP treatment. Each point in the PCA plot corresponds to the mean value for each group. The distribution of the samples was based on 110 PTR-ToF-MS masses, whose loading plot is illustrated in panel **b**. In panel **c** is instead represented the hierarchical heatmap of each single VOC assessed for each harvesting stage (H1–H2-H3–H4) after one and 5 days of shelf-life after cold storage, in both normal (C) and 1-MCP treated condition (T), respectively

The aroma of pear fruit was instead assessed through a PTR-ToF-MS. The entire volatilome was reduced from 214 to 110 VOC (volatile organic compounds) masses, applying noise and correlation coefficient thresholds (Supplementary Table S1). The aroma of pears was for the most represented by ester (m/z 61.028, 71.049, 75.042, 89.059, 117.091, 131.018, 145.123), acetaldehyde (m/z 45.032) and ethanol (m/z 47.047), with a profile similar to ethylene, being accumulated over the postharvest ripening (Fig. 1). The effect of the shelf-life ripening on the VOC production was clearly depicted in the PCA plot (Fig. 1a). The first principal component (PC1), which explained 94% of the overall variability, was oriented towards the projection of the samples assessed after 5 days of shelf-life ripening (Fig. 1b). These samples were also the only ones showing a distinct profile of VOCs, while all the others (assessed at harvest, after 1 day of shelf-life following cold storage or treated with 1-MCP), did not show any particular differences. The T1 + 5_ctrl_ stage showed in fact the highest accumulation of VOCs, and it is also interesting to note that fruit harvested at H1 produced a higher amount of VOCs compared to the other three stages (Fig. 1c, Supplementary Fig. S6).

Despite the progression observed for fruit firmness and VOCs, the accumulation of soluble solids linearly increased from harvest to postharvest stages (Supplementary Fig. S7) showing an ethylene-independent pattern, since the Brix value did not change after the exogenous application of 1-MCP.

### Global transcriptome analysis of the on-tree pear fruit maturation

**Fig. 2:**
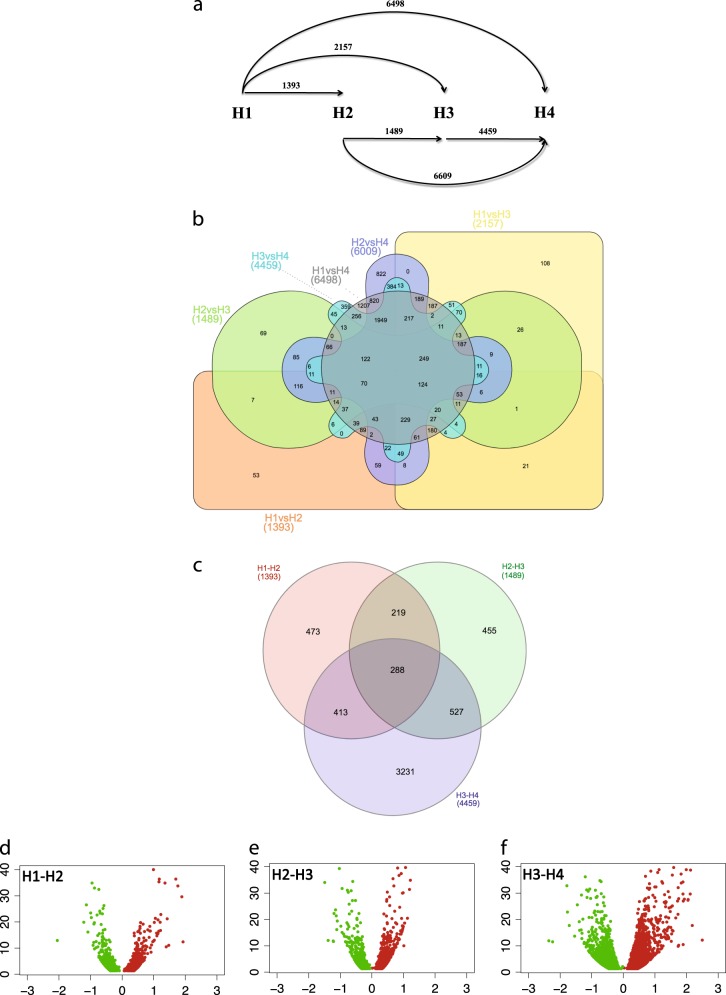
Analysis of the genes differentially expressed. In panel **a**, the number of genes differentially expressed is illustrated in the six pair-wise combinations carried out among the four harvesting stages. Distinctions between shared and unique genes are instead illustrated in the two Venn diagrams, depicting the distribution among all the samples (**b**) or considering only the transition stages (**c**). The proportion of the genes up (red color) and down (green color) regulated among the three transitions (H1–H2, H2–H3, and H3–H4) is instead illustrated with the volcano plot depicted in panel **d**, **e**, and **f**, respectively

The RNA sequencing of twelve libraries (three biological replicates per each harvesting stage, H1–H4) generated, after quality check, a total of 228.35 Million of reads (76 bp), with an average of 19 M reads/library. Only 214.93 Million sequences, with a mode at 76 bp (representing the 94.15% of the total reads), were selected and used for the alignment of the reads, while the remaining were discarded for low quality or short sequence length. The alignment of the reads on the reference genome of *Pyrus communis*, produced 45217 unigenes, assembled into 12246 scaffolds. Transcript counts were analyzed with DESeq2 that identified 8939 DEGs in a pairwise analysis across the different harvesting stages. The number of DEGs proportionally increased with the distance among stages (Fig. 2a, b). The transition between H3 and H4 was characterized by 3.2 times higher number of DEGs (4459) with regards to the other two pairwise comparisons (H1–H2: 1393 DEGs, H2–H3: 1489 DEGs), as depicted in the Venn diagram illustrated in Fig. 2c. The H3–H4 transition was also characterized by the highest transcriptomic variation (Fig. 2d–f). Moreover, while the transitions between H1–H2 and H2–H3 were characterized by 33 and 30.5% of genes uniquely expressed, respectively, the transition from H3 to H4 was distinguished by 72,5% of unique genes, while only 27.5% of the DEGs was shared with the other transitions.

**Fig. 3:**
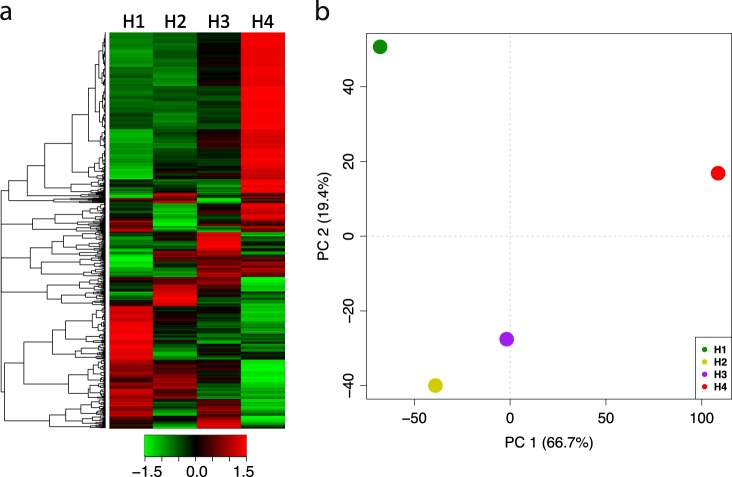
Expression profile of the DEGs across the four on-tree maturity stages. The expression dynamics of the DEGs is illustrated in panel **a** through a hierarchical clustering heatmap. In panel **b** is instead illustrated the distribution over a 2D-PCA plot of the four samples representing the four harvesting stages on the base of the DEG profile. Each point in the PCA plot corresponds to the mean value for each group. For both panels the four samples (harvesting stages) are indicated as H1, H2, H3, and H4

The expression of DEGs was furthermore graphically reordered with a hierarchical clustering heatmap (Fig. 3a) that showed a gradual and linear shift of gene expression from H1 to H3 and a decisive change in the H4 stage. The cluster of genes overexpressed at H1 was downregulated in H4, while, on the contrary, the upper cluster distinguished by genes with a lower expression in H1–H3 resulted strongly expressed in H4. The distinct transcriptional behavior observed in this latter stage, with regards to the other three samples, was furthermore magnified and illustrated in the 2D-PCA plot (Fig. 3b). The principal component analysis highlighted the distribution of the samples in a hyperspace defined by two principal components (PC1: 66.7% and PC2: 19.4%), accounting together for 86.1% of the entire variability present in the DEG dataset. From a visual inspection emerged the distinct transcriptional behavior of the fruit harvested at H4 stage. In fact, while H1, H2, and H3 were plotted in the PC1 negative area of the plot, H4 was located in the extreme area of the PC1 positive quadrant. Remarkably, in the PC1 negative area of the plot, the second principal component (PC2) distinguished the H1 stage from H2 and H3. More in detail, while H1 is positioned in the PC2 positive quadrant, H2 and H3 were located on the PC2 negative area of the PCA plot.

**Fig. 4:**
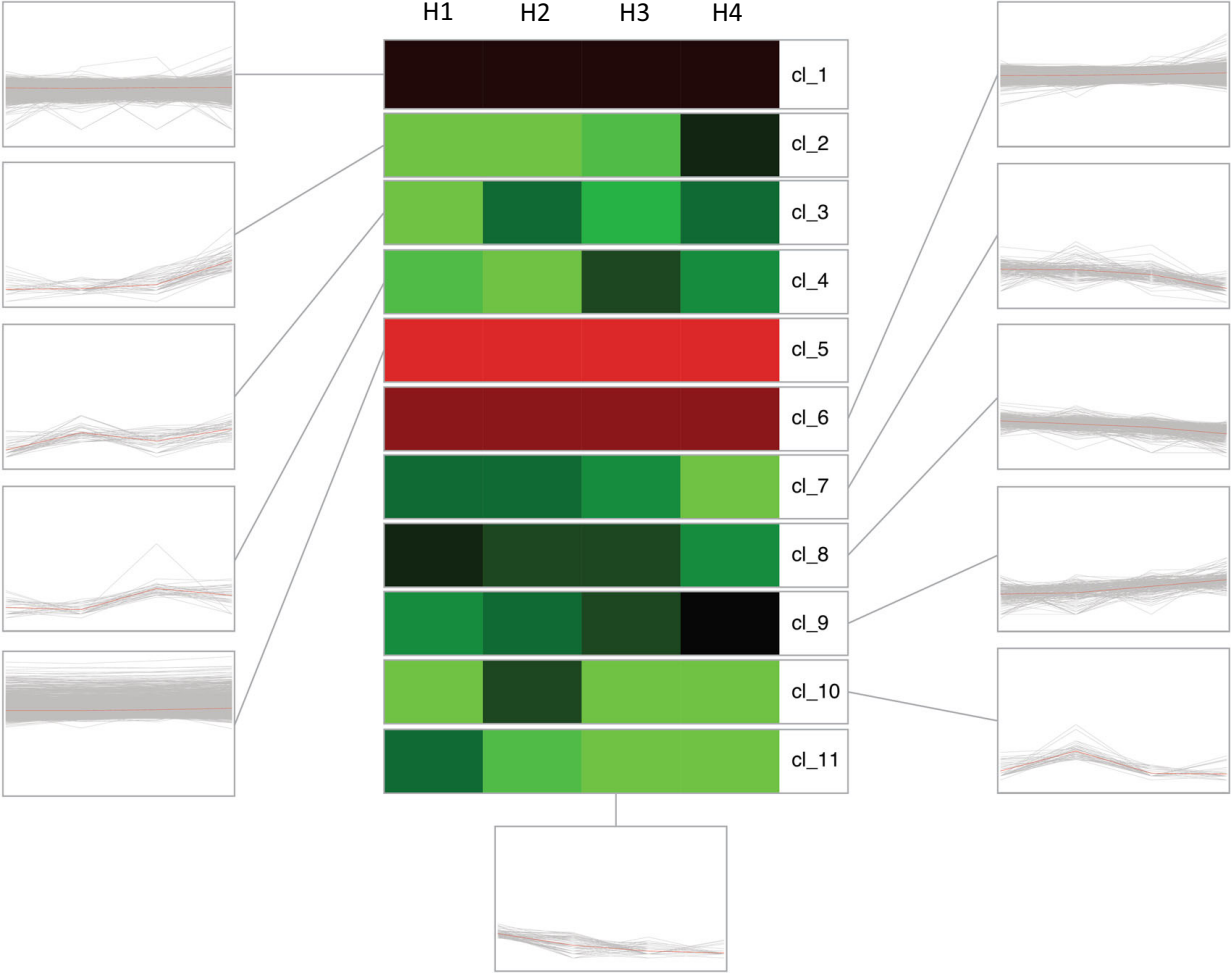
SOTA clustering of the DEG expression profile. The overall expression profile of the DEGs in RPKM was grouped in eleven clusters (coded as cl). For each cluster, the average of the expression profile for each harvesting stage (indicated with H1 to H4 on the top) is also reported

For a better characterization of the gene expression dynamics, the transcriptomic pattern of the DEGs (in RPKM) was sorted into 11 clusters based on two clustering algorithms (SOTA and k-means), and validated through an average distance method. Each cluster was defined by a specific number of elements, ranging from a minimum of 35 genes for cluster 4 to a maximum of 3352 grouped in cluster 6 (Fig. 4; Supplementary Fig. S8 and Supplementary Table 2). Amongst all, clusters 2, 3, and 9 were characterized by an upregulated expression pattern throughout the entire time course (through stage H1 to H4), while, on the contrary, clusters 7, 8, 10, and 11 were distinguished by an opposite pattern, showing a decreasing expression profile.

### DEG functional annotation and gene ontology

The genes differentially expressed were initially classified according to a functional vocabulary provided by the gene ontology (GO) assignment. The GO categorized the DEGs into three main ontologies, namely Cellular Component (83 groups), Biological Process (289 groups) and Molecular Functions (305 groups) (Supplementary Table 3). In the Cellular Component category (Supplementary Fig. S9a, showing the first ten groups counting for 82% of the total), the three main categories were represented by integral component of membrane (40%), nucleus (14%) and intracellular membrane-bounded organelle (4.9%). Similarly, for the Biological Process (Supplementary Fig. S9b, 10 groups accounting for 35% of the total) the three main groups were transcription DNA-template (8.7%), translation (0.2%) and metabolic processes (4.2%), while for the Molecular Functions (Supplementary Fig. S9c, 10 groups accounting for 41%) the three main categories were represented by DNA binding (6.25%), ATP binding (5.5%) and transferase activity (5.5%).

The GO terms were also assigned to the genes included within each SOTA cluster, especially to those showing both an up (clusters 2, 3, and 9) or downregulation (clusters 7, 8, 10, and 11). While for all the clusters the most relevant Cellular Component category was represented by the integral component of membrane, specific particularities were observed in the Biological Process category. In the group of up-regulated SOTA clusters, clusters 2 and 3 were enriched by cell wall modification (*P*-value 0.037) and response to auxin elements (*P-*value 0.0065). In the second group of clusters (with a downregulation of the gene expression across stages) cluster 7 and 8 were enriched in regulation of transcription and transcription DNA-template (*P-*value 0.007).

### Correlation analysis network between texture and DEGs

The Pearson correlation analysis computed between the DEGs and the fruit firmness assessed with both devices identified 1068 relationships considering both positive (0.95–1) and negative (−0.95 to −1) intervals. Out of these, 491 (46%) showed a negative correlation, while 577 (54%) were detected as positive (Supplementary Table 4). Amongst the elements negatively correlated with Firmness (thus positively correlated with softening) it is interesting to note the identification of four genes involved in hormone biogenesis and signaling, such as *ACO* (PCP009685 and PCP022491), and two elements involved in the auxin pathway, such as an *auxin-induced protein* (PCP019182) and *IAA-like auxin responsive factor* (PCP012166). In addition to this, in this group of genes two elements involved in the cell wall disassembly (*xyloglucan endotransglucosylase hydrolase protein*, PCP016523, and a *pectate lyase3*, PCP026151) and two transcription factors (*APETALA2*, PCP005843, and *MADS*, PCP007024) were also identified in this group of genes. Elements related to the auxin pathway were also observed in the group of genes positively correlated with the fruit firmness, such as *Auxin induced protein* (PCP036703), *Auxin Response Factor* (PCP022634) and an *Auxin efflux carrier* component (*PIN3*, PCP021016). About ethylene, only an *AP2 ethylene response factor* was identified (PCP016390). Together with this latter gene, other four transcription factors were detected, namely *COBRA-like* (PCP011926), *NAC* (PCP007739) and two *squamosa binding proteins* (PCP036084 and PCP011929). Also for this category, two cell wall modifying elements were identified (PCP039813 and PCP023195), but related to *expansin-like* genes. For the analysis of firmness carried out with the texture analyzer, 368 elements were defined in the correlation analysis network (Supplementary Table S4), out of which 141 correlations (38.3%) showed a negative pattern, while 227 (61.7%) a positive relationship. Among the negative correlation it is worth noting PCP022491, an *ACO* element involved in the ethylene pathway, while within the class of genes positively correlated with the maximum force is noteworthy the presence of two *NAC* transcription factors (PCP007739 and PCP013432) and two cell wall modifying genes, an *expansin-like* (PCP023195) and a *pectate lyase* (PCP016914).

### Candidate gene-based analysis of the postharvest ripening in pear

**Fig. 5:**
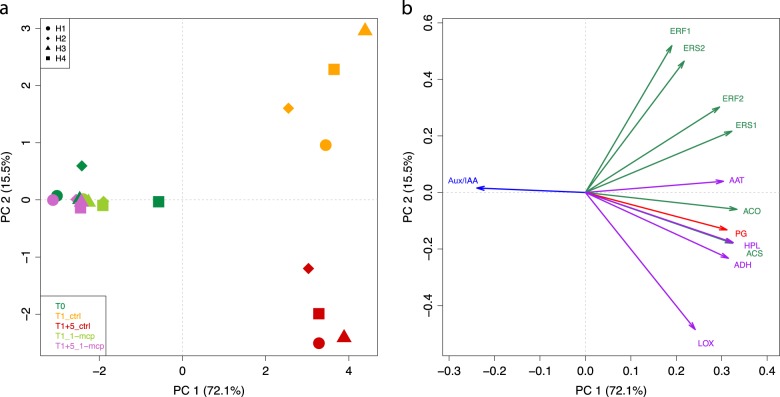
2D-PCA plot of the four samples based on the expression of the 12 candidate genes. In panel **a** is depicted the distribution of the samples representing the entire experimental design (on-tree maturity + postharvest) on a 2D-PCA plot. The stage of the sample was indicated by color, with dark green for harvest (T0), yellow (T1_ctrl_) for 1 month of cold storage in control condition, red (T1 + 5_ctrl_) for 5 days of shelf-life ripening after cold storage in control condition, pale green (T1_1-MCP_) for 1 month of cold storage 1-MCP treated and purple (T1 + 5_1-MCP_) for 5 days of shelf-life ripening after cold storage 1-MCP treated. The four harvesting stages are instead depicted with a different shape, according to the legend reported on the top-left of panel **a**. The orientation of the expression profile of this set of candidate genes was depicted in the loading plot reported in panel **b**. The color of each arrow indicate a specific pathway, such as green: ethylene, red: cell wall metabolism, purple: volatile organic compounds, blue: auxin. ACS: 1-aminocyclopropane-1-carboxylic acid synthase, ACO: 1-aminocyclopropane-1-carboxylic acid oxidase, ERS: ethylene response sensor, ERF: ethylene responsive factor, PG: polygalacturonase, AAT: alcohol acyl transferase, ADH: alcohol dehydrogenase, LOX: lipoxygenase, HPL: hydroperoxyde lyase, Aux/IAA: Auxin/indole-3-acetic acid protein

The postharvest fruit ripening, phenotypically characterized by ethylene and fruit firmness assessment, was also monitored detecting the expression of twelve candidate genes selected from the list of DEGs. The expression level of these genes was profiled by RT-qPCR, which also validated the RNA-seq pattern, showing correlation values ranging from 0.97 to 0.99. The elements employed for this investigation belong to three main functional categories. Within these classes, seven genes were involved in ethylene and auxin hormone biosynthesis and signaling (*ACS*, *ACO*, *ERS1*, *ERS2*, *ERF1*, *ERF2*, and *Aux/IAA*), while the remaining were involved in the cell wall dismantling process (*PG*) and volatile biosynthesis (*LOX*, *ADH*, *HPL*, and *AAT*). The expression profile of this gene set enabled a clear distribution of the entire array of samples (including both harvest and postharvest) over a 2D-PCA plot (Fig. 5a). The PC1 (accounting for 72.1% of the total variance of the expression detected for the 12 genes) clearly distinguished the samples collected at harvest (T0) from those of the postharvest stage (T1_ctrl_ and T1 + 5_ctrl_), indistinctively for the four harvesting stages (H1 through H4). In this plot, the role of the second principal component (PC2: 15.5%) effectively distinguished the two postharvest stages, characterizing the T1 (plotted on the positive PC2 quadrants) from T1 + 5 samples (plotted in the negative PC2 quadrants). It is also worth noting that the postharvest samples treated with 1-MCP (T1_1-MCP_ and T1 + 5_1-MCP_) grouped together with the T0 samples for all the four harvesting stages. This positioning was determined by the projection of all the genes towards the PC1, besides *Aux/IAA*, which resulted oppositely oriented (Fig. 5b).

The transcript accumulation of the genes involved in ethylene, cell wall, and volatile metabolism steadily increased from harvest (T0) to the two postharvest stages (T1 and T1 + 5 days), indistinctively for each harvesting stage (H1–H4) (Supplementary Fig. S10 and S11). Within the group of genes involved in the pathway of ethylene, however, a particular behavior was observed. While the expression of the two genes involved in the biosynthesis of the hormone (*ACS* and *ACO*) increased during the postharvest shelf-life ripening (from T1 to T1 + 5), the other elements, involved in the hormonal perception and signal transduction (*ERS1*, *ERS2*, *ERF1*, and *ERF2*), showed an opposite and decreasing pattern, with an expression profile higher in T1 than in T1 + 5 (Supplementary Fig. S10). A similar behavior was moreover observed for the VOC related genes. *LOX*, *HPL,* and *ADH* showed, in fact, an increased expression from T1_ctrl_ to T1 + 5_ctrl_, while *AAT*, involved in the production of ester, showed a decreasing trend (Supplementary Fig. S11). The expression profile of *PG*, involved in the cell wall metabolism was instead consistent with the transcriptional pattern of the genes involved in the Yang’s cycle (*ACS* and *ACO*), with an increasing expression during the shelf-life postharvest ripening. The expression of the eleven aforementioned genes resulted, moreover, severely repressed by the application of 1-MCP, showing, in some cases, an almost undetectable expression level. In the end, it is worth noting the particular expression profile of *AuxIAA*, a gene involved in the signaling of auxin (Supplementary Fig. S10). Across the four harvest stages, the expression of this gene was completely opposite to what was observed for the other elements investigated here. The exogenous application of 1-MCP induced, moreover, a burst in the expression of this gene in postharvest samples, with T1 + 5_1-MCP_ showing the highest expression value for H1, H2 and H3 and almost an equal value to T0 for H4.

**Fig. 6:**
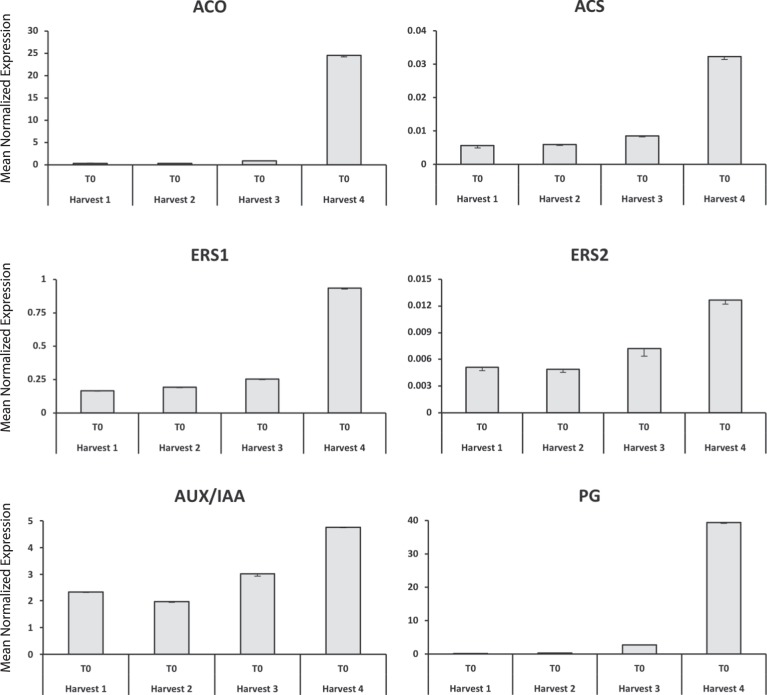
Expression profile of six candidates (*ACS*, *ACO*, *ERS1*, *ERS2*, *Aux/IAA*, and *PG*) across the four T0 samples (harvest). For each gene, the y-axis indicates the mean normalized expression, while the x-axis points to the four samples employed in this study. Bars indicate the standard error

A subset of six genes (*ACS*, *ACO*, *ERS1*, *ERS2, Aux/IAA,* and *PG*) was further employed to investigate the expression profile across the T0 samples (harvest) of the four harvesting stages (H1–H4). The expression profile of this candidate genes subset (Fig. 6), besides validating the expression pattern of the RNA-seq profile, unraveled a transcriptional pattern along the on-tree fruit maturation. For all these six genes, in fact, while the mRNA accumulation remain at a basal and constant level across the first three stages (H1–H3), a higher expression was observed in H4, which showed thus the highest gene transcript accumulation.

## Discussion

### Ripening progression in pear cv. ‘Abate Fetel’

The progression of the on-tree fruit maturity was non-destructively determined by a DA-meter. The variation in I_AD_ value analytically weighted the change in the physiological maturation of the fruit through the harvesting period of 4 weeks, but it is during the postharvest fruit ripening that the I_AD_ value showed the typical climacteric behavior of pear. It is in fact during the shelf-life ripening (T1 + 5) that an important drop in I_AD_ was observed, in coincidence with the burst of ethylene. The ethylene-dependent regulation of the postharvest fruit ripening was moreover validated by the exogenous application of the ethylene competitor 1-MCP. Similarly to what has been observed in apple^14^, the ethylene competitor treatment delayed considerably the fruit ripening process, maintaining the I_AD_ value closer to harvest (H0). Consistent with other climacteric species^4,15–20^, the production of ethylene directly influenced the regulation of physiological pathways controlling important fruit quality features, such as the dismantling of the cell wall structure (enabling fruit softening) and the production of aromatic volatile organic compounds. The most significant reduction in fruit firmness was observed in the last stage of the time course, with the T1 + 5_ctrl_ sample showing the lowest value in fruit firmness. It is in fact known that firmness degradation, in climacteric fruit, is mainly coordinated by ethylene^3,21^, as demonstrated in this survey through the application of 1-MCP. The interference with ethylene determined, in fact, an evident reduction in loss of fruit firmness, indistinctively by the four harvesting stages. About the assessment of fruit firmness operated in the two areas of the fruit with the Texture Analyzer, it is also interesting to note the two different correlation behaviors with firmness. The result suggests the presence of a cell wall dismantling gradient within the fruit, which should be taken into consideration and further exploited for a better characterization of the fruit firmness in pear fruit.

Together with ethylene, other important volatiles were assessed with the PTR-ToF-MS instrument, such as the aromatic VOCs. The aromatic blend typical of pear, and mostly represented by esters and alcohols, were in fact correlated with the production of ethylene. The main modification of the volatilome was indeed detected in the T1 + 5 stage, following an ethylene-dependent trend, since the treatment with 1-MCP completely turned down the emission of VOCs. Treated fruit, in fact, did not show any particular differences with regards to the sample at harvest. Interestingly, we observed that the maturity stage played a role in the production of aromatic compounds in fully ripe fruit. The fruit at T1 + 5_ctrl_ and collected at H1 (first harvesting date) showed the highest concentration of ester compounds identified with the masses m/z 75.042 (i.e., methyl acetate), 89.059 (i.e., ethyl acetate), 117.091 (i.e., isobutyl acetate), 131.018 (i.e., isoamyl acetate) and 145.123 (i.e., ethyl hexanoate).

Despite the climacteric behavior observed for these two quality features, the soluble solids showed a contrasting accumulation pattern. The application of 1-MCP, in fact, did not affect the accumulation of sugars, which resulted an ethylene-independent trait, as already described in other works^22,23^.

### RNA-sequencing highlights a transcription dynamic following the on-tree fruit maturation

The whole-transcriptomic survey carried out in this work generated a total number of 45217 unigenes that analyzed across the four harvesting stages allowed the identification of 8939 genes differentially expressed. The number of DEGs across the several pair-wise comparisons changed according to the distance among stages, with the smallest number observed between H1 and H2 (1393) and the highest between H1 and H4 (6498). The increased number of DEGs towards the end of the experimental design was furthermore determined by the transcriptional pattern observed for H4 with regards to the samples collected in the other three stages (Fig. 3). Although the hierarchical clustering illustrated a continuous change in the transcription dynamics throughout the on-tree fruit maturation, the most important change was observed in H4. The transition between H3 and H4 can be ascribed as the most relevant, affecting, therefore, the subsequent postharvest performance of the fruit. The expression profiles of the DEGs represented in the hierarchical clustering were further grouped into 11 SOTA clusters, of which three (2, 3, and 9) were represented by elements whose expression increased towards the H4 stage. The annotation of DEGs revealed as these clusters were particularly represented by genes known to play a crucial role in triggering the climacteric ripening such as *ACO* (PCP022491, cl-3; PCP009685, cl-9) *ACS* (PCP011500, cl-2; PCP013122, cl-2), *AP2/ERF* (PCP027843, cl-2; PCP014930, cl-9) and *auxin induced protein* (PCP011951, cl-9^3,4,21^). From the total of 8939 differentially expressed genes, 6578 showed a positive match with the UniProt-TrEMBL database. Out of this set, 69% (corresponding to 4556 elements) were functionally assigned at least to one of the following three gene ontology categories: Cellular Components (22%), Biological Process (18%) and Molecular Functions (28%). The analysis of the GO term was specifically focused on seven SOTA clusters, including genes showing both an up (cluster 2, 3, and 9) or down (cluster 7, 8, 10 and 11) regulation. All these clusters were distinguished by a high representation of integral component of membrane in the Cellular Component category, while the other two categories showed a more distinct assignment. In the group of clusters containing genes up-regulated throughout the on-tree fruit maturation, cluster 2 showed most of the elements functionally annotated in the Biological Process category, such as cell wall modification and response to auxin as well as pectin esterase activity for the Molecular Function. The identification of a *pectin esterase* (PCP011895) in cluster 2, whose expression increased throughout the progression of the fruit maturity, is consistent with the physiological role of this element in the disassembly of the pectin network^24^. The expression of this gene is moreover consistent with the discovery of two *auxin responsive proteins* genes (PCP012330 and PCP029646). The other two clusters (3 and 9) composed by elements over-expressed from H1 to H4, resulted particularly characterized by GO term assigned to internal components of membrane. Cluster 3, moreover, showed the presence of genes important for the fruit maturity, such as *ACO* (PCP022491), *auxin-induced protein* (PCP022563), and *endo-1,3-beta glucosidase*. The co-identification of genes involved in both hormonal signals was also observed in cluster 9, with the annotation of *ACO* (PCP009685), *ERF* (PCP011831, PCP014930), and *auxin-induced protein* (PCP011951).

The transcriptional association between auxin and ethylene-related genes was also observed in the correlation analysis carried out between the set of DEGs and the assessment of fruit firmness. Within the group of genes negatively correlated with fruit firmness (thus positively involved in the softening process) were in fact identified *ACO*, *auxin-induced protein* and *ARF* elements. The correlation analysis shed light also on genes specifically involved in the control of the softening process, such as *xyloglucan endotransglucosylase* and *pectate lyase*, known to be involved in the cell wall dismantling process of several fruit species^14,25,26^. Another cell-wall related gene was identified also in the group of DEGs positively correlated with the pattern of firmness (therefore negatively associated to softening) and identified as an *expansin* gene. This gene is known to play a myriad of roles during the entire plant physiology, especially on the remodeling of the cell wall architecture^27^. In the end, it is worth noting the identification of a series of transcription factors, such as *APETALA2*, *MADS*, *NAC,* and *COBRA-like*. However, while the first two regulatory elements are known to play important role in the control of ripening, with relevant consequence on the fruit firmness^28,29^, the involvement of the other categories of transcription factors is not fully elucidated yet. In tomato, the over-expression of *SlNAC1* induced a diminished accumulation of ethylene proper of the system 2 and an anticipated softening compared to wild-type fruit^30^. *COBRA-like* genes were instead correlated with the reduction of the fruit softening process. Cao et al.^31^ reported, in fact, that a *SlCOBRA-like* gene was involved in the assembling of the cell wall in fruit of tomato, and *COBRA-*overexpressing line showed a higher amount of cellulose and a reduced solubilization of pectine compared to the wild-type.

### Transcriptional signature of the postharvest ripening of pear by candidate gene approach

At the end of cold storage, the fruit of ‘Abate Fetel’ collected from the four harvesting stages completed the ripening process, contrarily to what was previously observed by Hansen^32^ and Nham et al.^13^. These authors, in fact, reported that fruit of ‘Bartlett’ and ‘d’Anjou’ pear cultivars presented a different capacity of ripening dependent to the maturity stage. However, while the maturity investigated by Nham et al.^13^ spanned from 100 to 120 DAFB, this study was more shifted towards the commercial harvesting. The time course we analyzed started, in fact, at 120 DAFB and continued until 147 DAFB, following the real harvesting carried out in a medium-size type of orchard. The progression of the postharvest ripening was characterized, in this study, through the expression of twelve candidate genes involved in three main physiological pathways: hormone (ethylene and auxin), cell wall metabolism and aromatic volatiles. The overall PCA of the transcriptional pattern of this set of candidate genes efficiently distinguished the samples into three main groups, according to the climacteric ripening behavior. The PC1 clearly differentiate the samples producing ethylene (T1_ctrl_ and T1 + 5 _ctrl_) from those with an impaired ripening (T1_1-MCP_ and T1 + 5_1-MCP_) (Fig. 5a), together with the samples harvested in a pre-climacteric phase (T0). The subsequent implementation of the PC2 further distinguished the T1 from the T1 + 5, which are characterized by a different hormonal production. The distribution of the samples over the 2D-PCA plot is determined by the loading of each single gene (Fig. 5b). The T1 + 5_ctrl_ samples are in fact projected according to the orientation of *ACS* and *ACO* genes, key elements in the biosynthesis of ethylene. The detailed inspection of the expression pattern of the genes related to the ethylene metabolism highlighted two specific transcriptional patterns (Supplementary Fig. S10 and Fig. S11). The first trend is shared by the two genes involved in the production of ethylene (*ACS* and *ACO*), whose expression was consistent with the accumulation profile of this phyto-hormone. The second transcriptional pattern was instead observed in the group of genes involved in the perception and signaling cascade (*ERSs* and *ERFs*). The mRNA accumulation of these elements was in fact higher in T1 than T1 + 5. This negative relationship with the ethylene accumulation profile was already observed in other fruit crops, such as banana^33^ and apple^34^, confirming, especially for the latter species, the results observed and discussed here. The pattern of these genes could also be justified by the fact that *ERFs* are stimulated at the end of the ethylene cascade, triggered by *ERSs*, which are, moreover, negative regulator of the ethylene signaling^35^. The ethylene-dependent regulation of important fruit quality characteristics, such as fruit firmness and aroma, was furthermore confirmed by this candidate-gene based survey. The *PG* gene here investigated, which represents the major category of cell wall modifying enzymes devoted to disassembly the cell wall-middle lamella structure^24^, showed a pattern consistent with *ACS* and *ACO*, thus more expressed in T1 + 5 sample. Similarly to this, also three genes involved in the aroma production (*LOX, ADH*, and *HPL*) showed a higher expression profile coincident with the ethylene burst. The ethylene-dependent regulation of these genes (together with the phenotype they control) was finally confirmed with 1-MCP, since the exogenous application of the ethylene competitor turned down completely their expression level.

To this end, it is moreover interesting to note the opposite behavior of the gene *Aux/IAA* related to the auxin pathway. The expression of this gene was in fact stimulated by the application of 1-MCP, and in the PCA loading plot it resulted contrarily projected with regards to the rest of genes (Supplementary Fig. S10 and Fig. 5b). In a normal ripening physiology, auxin is known to retard ripening, and in ‘Bartlett’ pear it has been demonstrated that ripening was stimulated by the breakdown of this hormone^36,37^, as well as in other fruit species, such as tomato, grape, and strawberry^38,39^. In this latter species, however, it has also been shown that a *pectin esterase-*like gene was stimulated by the presence of auxin^40^. The interplay between auxin and ethylene was also observed in apple^14^, where it was proposed that the reactivation of the auxin pathway occurred as a physiological response contrasting the block of ethylene perception during the climacteric phase, as a tentative to restore the normal ripening physiology. This theory is supported by the recent findings emphasizing the involvement of auxin in promoting the ripening process in other climacteric species. In the mesocarp of peach fruit, in fact, an increased content of both auxin and ethylene was reported by other authors^41,42^, and *Aux/IAA* and *IAA3* genes were induced during the ripening of tomato^43^. Lately, an auxin-responsive factor (*ARF2*) identified in tomato was specifically expressed during ripening, and transgenic line of tomato over-expressing this gene showed an accelerated fruit ripening^44^. These findings, in the end, are fully consistent with the results obtained in this study on the transcriptional profile of *Aux/IAA* in pear, which suggests the role of auxin in the initiation and control of the climacteric ripening in pear. The presence of auxin seems thus essential to stimulate the initiation of the climacteric ripening, which progression is thereafter allowed by the hormonal breakdown. The triggering role of auxin in the climacteric ripening is furthermore confirmed by the up-regulation of the *Aux/IAA* gene following the treatment with 1-MCP. The remove of ethylene during the climacteric phase induced a de-repression of this gene, in order to restore a normal ripening physiology. Although additional evidences about the involvement of auxin in the triggering of the climacteric ripening are compelling, these transcriptional data suggest the existence of such hormonal cross-talk also in pear, similarly to what has been recently reported for apple^14,45,46^.

### Candidate gene-based transcriptomic characterization of the on-tree fruit maturation

Amongst the set of candidate genes employed in this work, six genes resulted to be particularly interesting to distinguish the four harvesting stages (Fig. 6), therefore useful to investigate the progression of the on-tree fruit maturation. Five out of the six genes were involved in hormone regulation, such as *ACS*, *ACO*, *ERS1*, *ERS2* for ethylene and *Aux/IAA* for auxin. The last one, *PG*, was instead involved in the cell wall metabolism. Indistinctively to the physiological pathway they belong, these genes resulted uniformly over-expressed at the end of the postharvest ripening observation (Supplementary Fig. S10 and Fig. S11). However, when their expression profile was analyzed among the four harvesting stages (from H1 through H4), a distinct up-regulation was observed at the end of the on-tree fruit maturity observation (H4), while during the first three harvesting times (H1–H3) a more stable transcriptomic pattern was observed. The increased expression in H4 suggested that fruit at this stage are more advanced in their physiological ripening. The higher expression detected in H4, consistent with the RNA-seq analysis, suggested that fruit collected at this stage would probably have a shorter postharvest life and a lower postharvest performance.

The global analysis presented here shed lights on a transcriptional kinetics through the four harvesting stages, evidencing important regulation occurring in the fruit during the maturation process. The role of ethylene in the ripening control of pear was also dissected by the exogenous application of 1-MCP, supporting the theory of an existing hormonal cross-talk between ethylene and auxin. The genes identified and described here might represent moreover a useful toolbox to transcriptionally monitor the on-tree fruit maturation as well as to characterize the postharvest fruit ripening, important to improve the fruit quality in pear.

## Material and methods

### Fruit material and experimental design

Pear fruit of cv. ‘Abate Fetel’ were harvested from trees grown at the experimental orchard of the Department of Agricultural Science of the University of Bologna. Fruit were collected at four harvesting dates (H1 at 120 DAFB, days after the full bloom; H2, 127 DAFB; H3, 140 DAFB, and H4, 147 DAFB; following the harvesting ongoing in a commercial orchard). From each harvesting stage, about 1000 pears were randomly collected from 30 trees. The ripening of each fruit was assessed through the determination of the I_AD_ (index of absorbance of chlorophyll) using a DA-meter, a portable non-destructive device based on vis/NIR spectroscopy (TR, Forlì, Italy). From each stage, 100 homogeneous fruit were selected on the base of the mode of the I_AD_ distribution and used for further analysis.

In order to elucidate the role of ethylene in controlling the postharvest ripening of pear, fruit at harvest were divided in two batches, the first was used as control (ctrl), while the second was treated with 1-methylcyclopropene (1-MCP). The overnight treatment with 1-MCP was carried out with SmartFresh^®^ (0.14% active ingredient), according to the manufacturer’s instructions (AgroFresh), reaching a final concentration of 0.7 µl L^−1^. Fruit were exposed to 1-MCP for 24 h at 20 °C and subsequently placed in cold storage at 0.5 °C with 95% of relative humidity. Both treated and non-treated batches were stored with identical conditions. For each harvesting, fruit were furthermore divided in three groups, the first one (T0) was assessed right after harvest, while the other two were cold stored for a month (0.5 °C at room atmosphere) and further assessed after one (T1) and five (T1 + 5) days of shelf-life at room temperature (20 °C), respectively.

### Physical and chemical analysis of the quality of pear fruit

For each sample/harvesting, the quality of ten homogeneous fruit was characterized analyzing four main properties, such as fruit texture, ethylene concentration, VOC (volatile organic compound) production and the concentration of soluble solids. Fruit texture was assessed with the use of two devices. The first one was represented by a manual digital penetrometer (TR, Forlì, Italy), while the second was a sophisticated TAXT*plus* texture analyzer (StableMicrosystem, Godalming, UK), already employed to assess the fruit texture in apple^47,48^. The fruit firmness assessed through the penetrometer (expressed in kg cm^−2^) was carried out on whole fruit, taking two measurements/fruit at opposite sides on the radial part of the pear. For the analysis of the fruit firmness operated with the Texture Analyzer, the radial part was isolated from the fruit and measurements were taken in two areas, external (close to the measurement of the penetrometer) and internal (closer to the seed cavity), as illustrated in Supplementary Fig. S12. The concentration of ethylene in both control and 1-MCP treated fruit, was non-destructively carried out on 4 pears/harvesting stage with a Varian 3300 gas-chromatograph, equipped with a Poropak column QS 80/100 at 80 °C and a FID detector at 150 °C.

VOC fingerprinting was instead assessed with a proton transfer reaction–time of flight–mass spectrometer (PTR-ToF-MS 8000 apparatus; Ionicon Analytik GmbH, Innsbruck, Austria^49^). For this analysis, four replicates of 1 g of powdered frozen tissue of fruit flesh, stored at −80° prior the analysis, was transferred into a 20 ml glass vial with a PTFE/silicon septa (Agilent, Santa Clara, CA, USA) and mixed with a 1 ml of deionized water, 400 mg of sodium chloride, 5 mg of both ascorbic and citric acid, and then stored at 4 °C until analysis^49^. The array of masses detected with the PTR-ToF-MS was reduced by applying noise and correlation coefficient thresholds, removing peaks not significantly different from blank samples and having isotopes of monoisotopic masses^50,51^. Compound annotation was carried out comparing the spectral profile with fragmentation data of reference standards. Absolute headspace VOC concentrations expressed in ppbv(parts per billion by volume) were calculated from peak intensities^51^.

The content of soluble solid (°Brix value) was finally determined with a standard refractometer, which measured the refractive index.

### RNA extraction and quantification

Total RNA was extracted from frozen portion of cortex (peeled flesh tissue) using the Spectra Plant Total RNA Kit (Sigma-Aldrich, MO, USA), following the protocol provided by the manufacturer. Concentration and purity of the isolated RNA was further assessed with a Nanodrop 8000 Spectrophotometer (ThermoFisher Scientific, MA, USA), while the RNA integrity was analyzed with the Tapestation 2200 (Agilent Technologies, CA, USA). Only samples with a RIN ≥ 8 were used for RNA-seq analysis.

### Library preparation and RNA-sequencing

The large-scale transcriptome analysis was carried out through RNA-seq approach. To this end, single-end libraries were prepared with the NEB Next Ultra II kit (BioLabs inc., New England, following the instruction provided by the manufacturer) for each T0 samples collected from the four harvest stages (H1, H2, H3, and H4), and considering three biological replicates per sample. The transcript population for each sample was sequenced with an Illumina NextSeq 500 (read length of 75 bp) at the Functional Genomic Lab (http://ddlab.sci.univr.it/FunctionalGenomics/) of the University of Verona (Italy). RNA-seq data are available at the GEO database (Accession number: GSE113517).

### Data analysis and gene annotation

Sequencing reads were analyzed with the cyber infrastructure CyVerse^52^, implementing into the Discovery Environment the New Tuxedo protocol.

Quality check of raw sequence, adapter and read trimming (based on a default value of 20) were operated with FastQC (http://www.bioinformatics.babraham.ac.uk/projects/fastqc), Scythe (https://pods.iplantcollaborative.org/wiki/display/TUT/Scythe-0.991+using+DE) and Sickle (https://github.com/najoshi/sickle) software, respectively. The New Tuxedo protocol was composed by three main steps. The software HISAT2 (http://ccb.jhu.edu/software/hisat2/index.shtml) was initially used to align the reads on the *Pyrus communis* reference genome^53,54^. StringTie (http://ccb.jhu.edu/software/stringtie; employed with standard parameters) was further implemented to assemble and quantitate RNA-seq reads into transcripts. In the end, the R package DESeq2, with default parameters^55^, was used to identify the differentially expressed genes (DEGs) across the four harvesting stages. The number of DEGs was established through an adjusted P value (FDR) < 0.05. Since functional annotation of *Pyrus communis* available at the GDR database is dated to 2013, we decide to exploit the UniProt-TrEMBL database to take the advantage of a more updated source of information. Functional annotation of DEGs were performed by a protein sequence similarity searching (using ncbi-blast software ver 2.7.0) against the “plants” taxonomic division of UniProt-TrEMBL database, a subsection of the database holding only sequences from “viridiplantae” taxon^56^. Once aligned, results were filtered using a python script developed in house (available at https://github.com/cestaroa/tab_blast_parser). Only alignments with a level of sequence identity greater than 50% covering at least the 80% of query length sequence and with an e-value lower than 10E−6 were retained. From these results, Gene Ontology terms^57^ were obtained from the UniProt-TrEMBL annotations. Gene enrichment analysis for Gene Ontology terms was carried out with the R package topGO, implementing the Fisher’s exact test.

The Venn diagrams were generated with the web-based tool InteractiVenn^58^. SOTA (Self Organizing Tree Algorithm) clustering was computed with the R package *clvalid*^59^, while the hierarchical clustering heatmap with a complete method was created with the use of heatmap3 R package.

The correlation between the fruit firmness values, calculated with both the penetrometer and the TAXT*plus*, was calculated with the Cytoscape plugin Expression Correlation Matrix^60^, which computed a Pearson correlation relationships among entities.

### RealTime qPCR

For RT-qPCR, 2 µg of total RNA was reversibly transcribed with the SuperScript VILO cDNA Synthesis kit (Invitrogen). Real Time qPCR was performed with the ViiA7 PCR system (ThermoFisher Scientific) with the following thermal profile: 95 °C 1 m, 40 cycles of 95 °C 1 m, 60 °C 20 s and 95 °C 15 s, with the final step of 60 °C 1 m and 95 °C per 15 s. Twelve genes were retrieved from the set of DEGs detected within the RNA-seq and investigated. Six genes were related to ethylene (*ACS*, *ACO*, *ERS1*, *ERS2*, *ERF1*, and *ERF2*), one was involved in cell wall metabolism (*PG*), while the rest of the elements were involved in auxin (*Aux/IAA*) and production of VOCs (*AAT*, *HPL*, *ADH* and *LOX*) (Supplementary Tab. S5). Relative gene expression was represented as a mean of normalized expression. In order to represent the relative gene expression the mean normalized expression was calculated. The average of three threshold cycle (CT) values independently calculated was computed to obtain the normalized expression value for each sample with the Qgene software^61^.

## Supplementary information

Supplementary Info
