# Supplementary material for: Wide transcriptional investigation unravel novel insights of the on-tree maturation and postharvest ripening of ‘Abate Fetel’ pear fruit
Source: Hortic Res. 2019 Mar 1;6:32. doi: 10.1038/s41438-018-0115-1 (PMC6395599; doi:10.1038/s41438-018-0115-1)
Supplement: Supplementary file 1 — Supplementary Info [file 41438_2018_115_MOESM1_ESM.pdf]

Supplementary Fig. S1

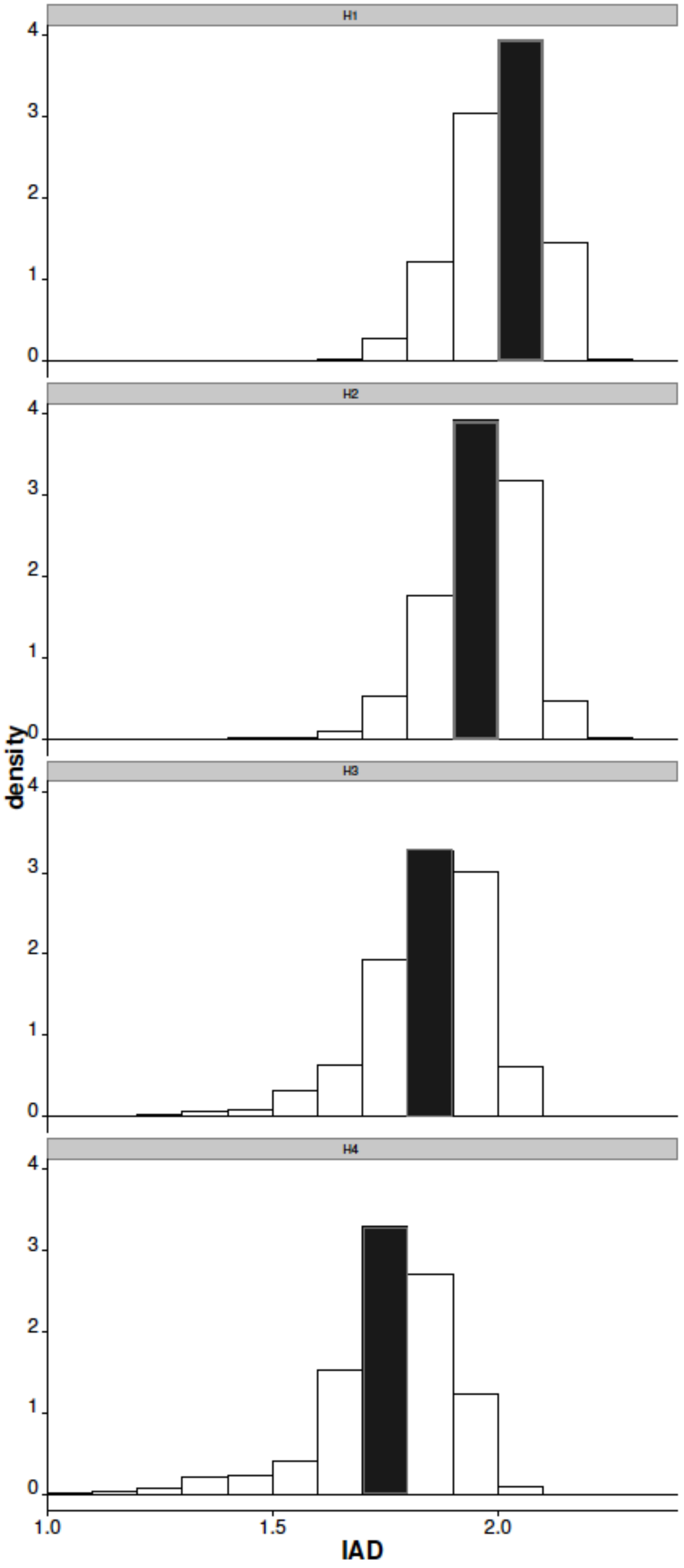

Supplementary Fig. S2

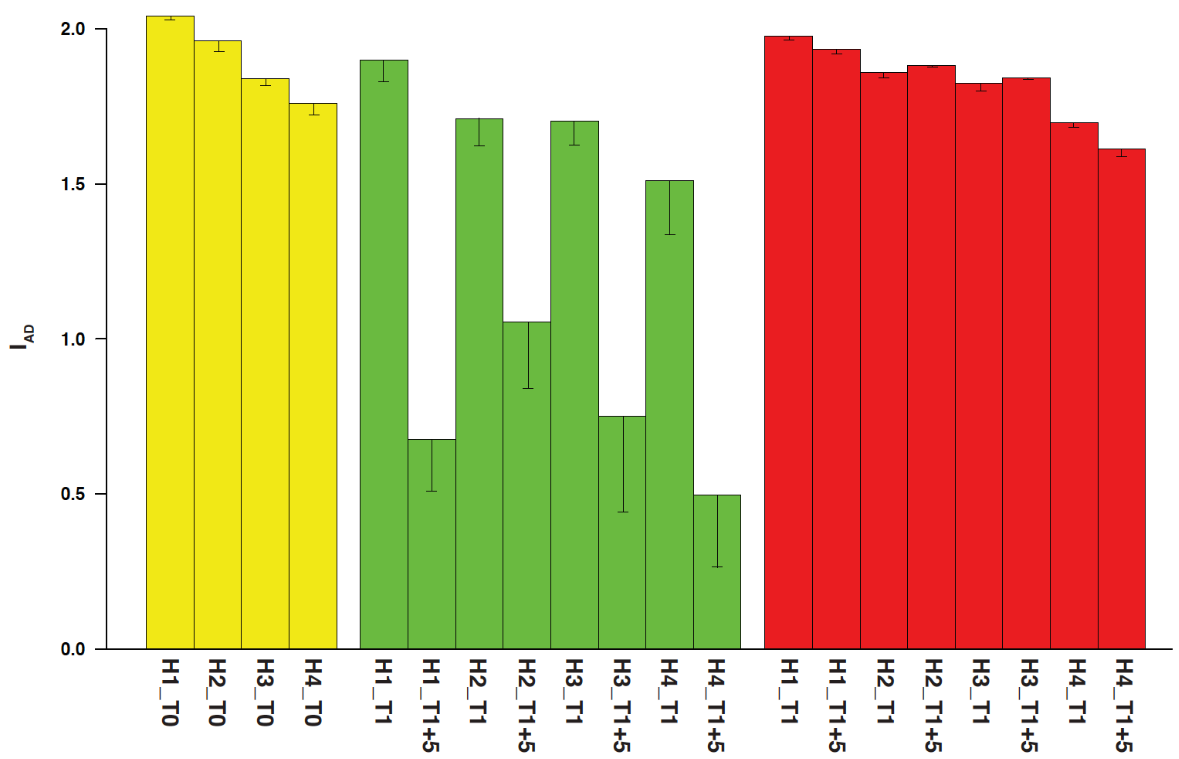

Supplementary Fig. S3

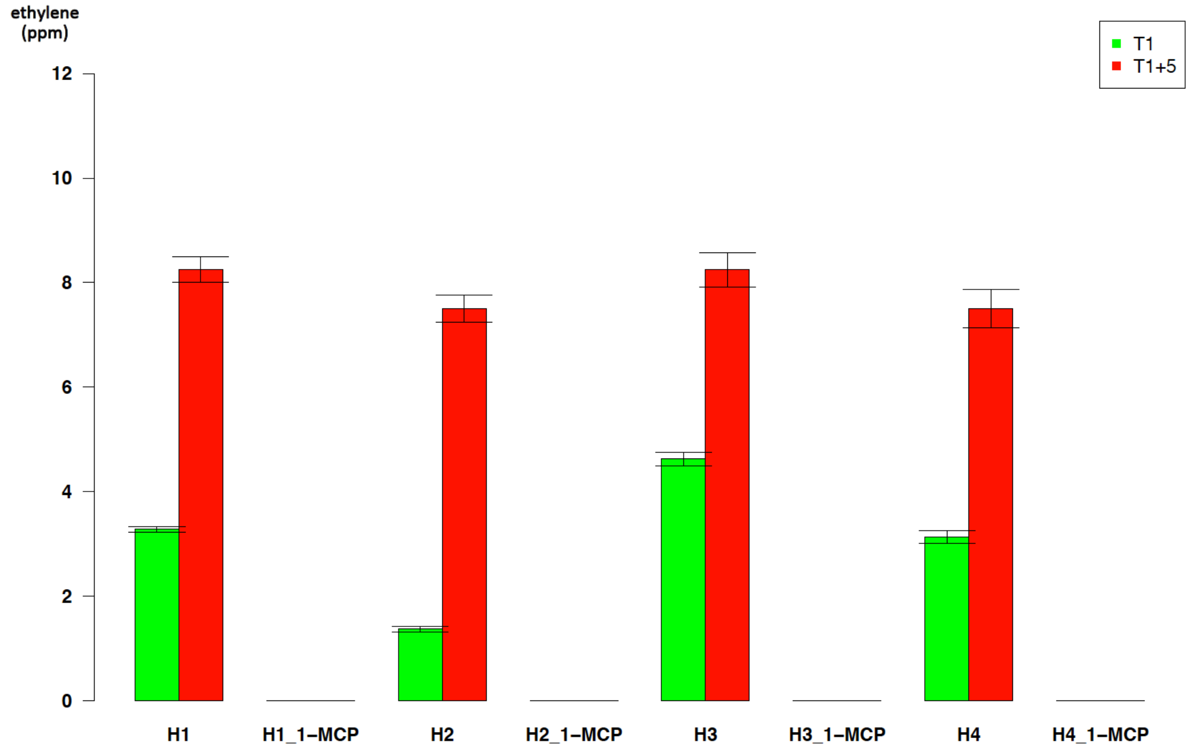

Supplementary Fig. S4

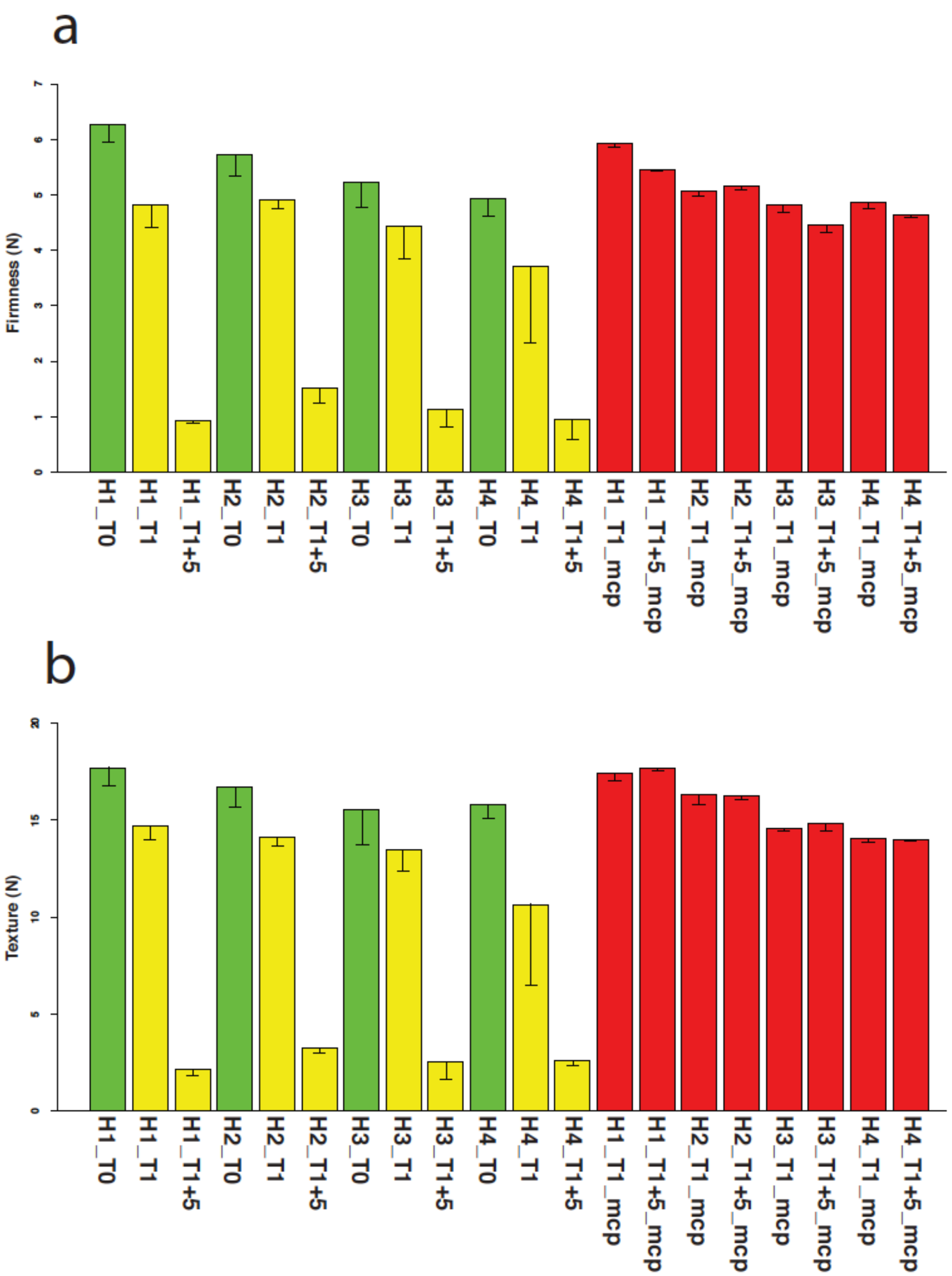

Supplementary Fig. S5

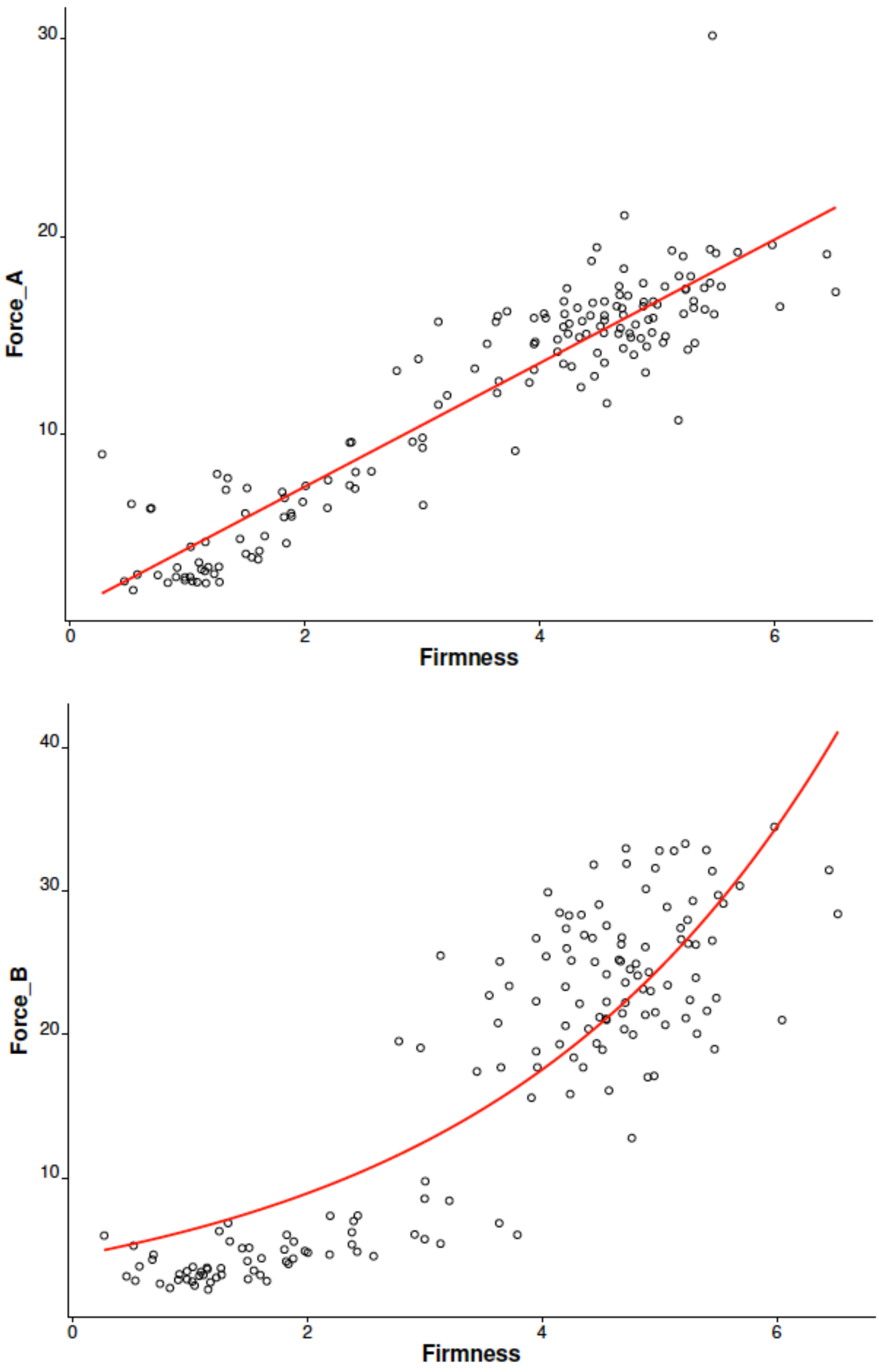

Supplementary Fig. S6

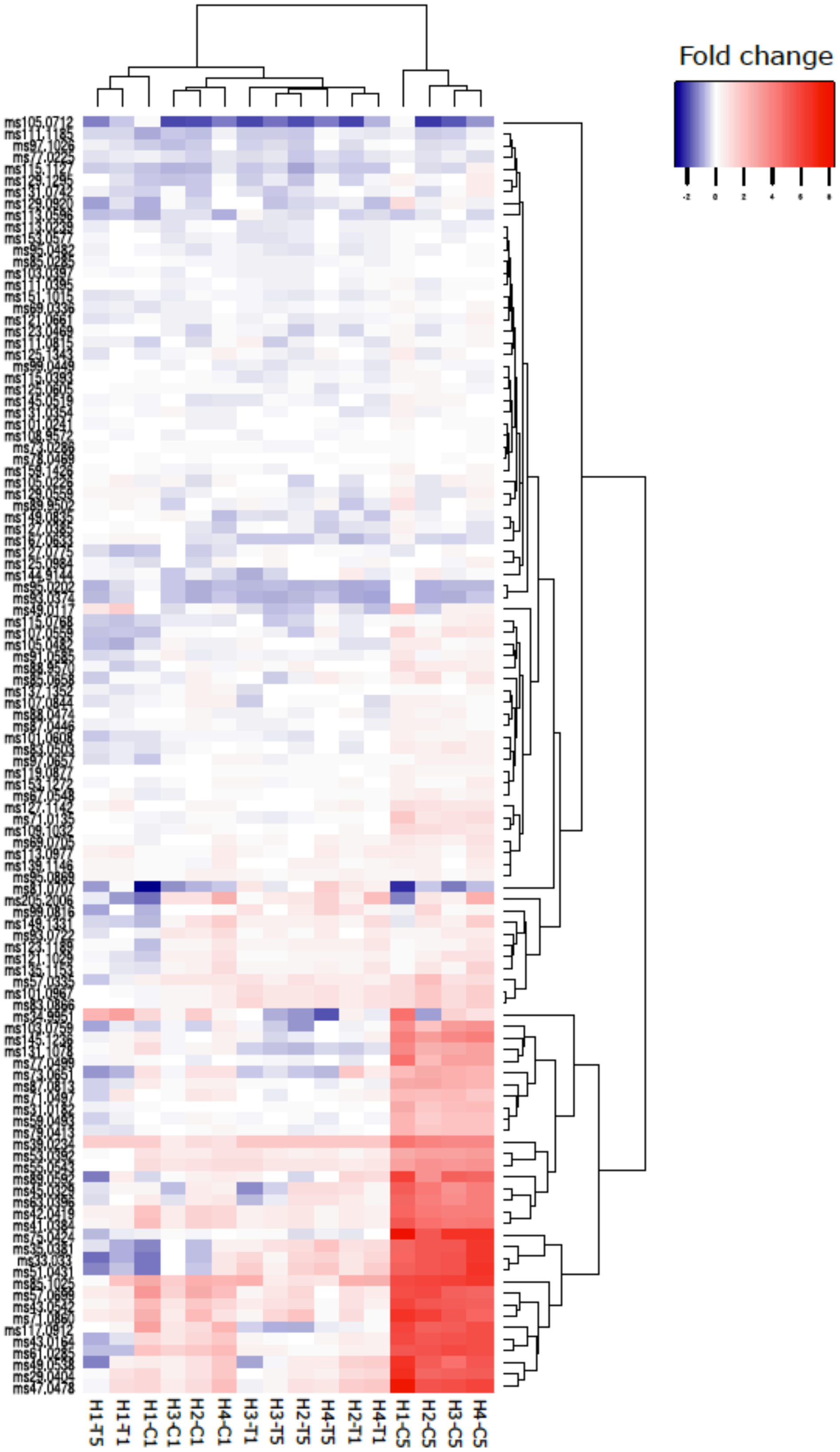

Supplementary Fig. S7

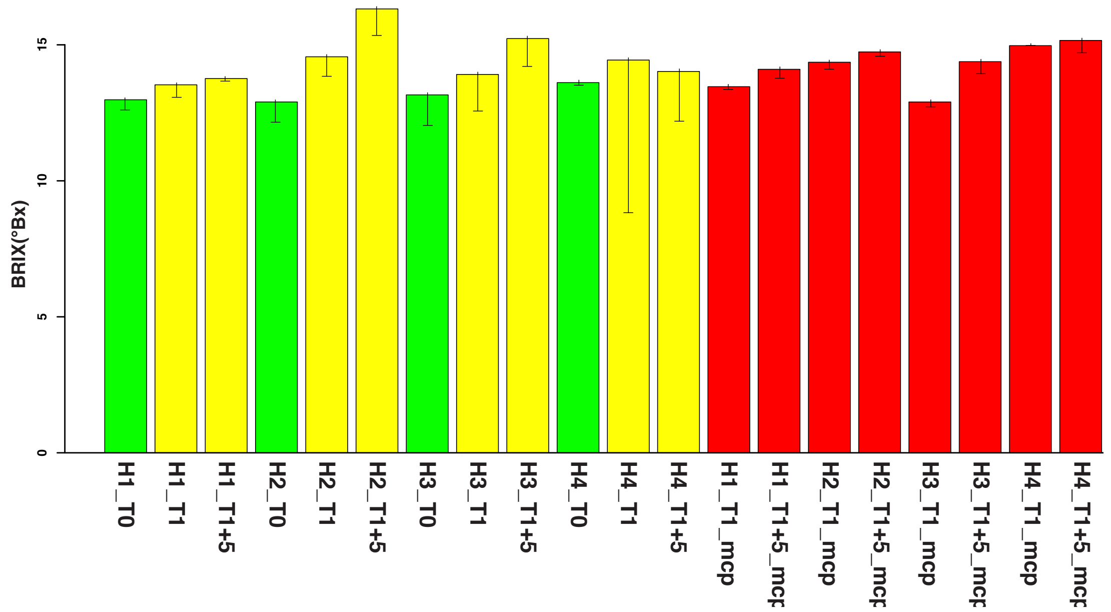

Supplementary Fig. S8

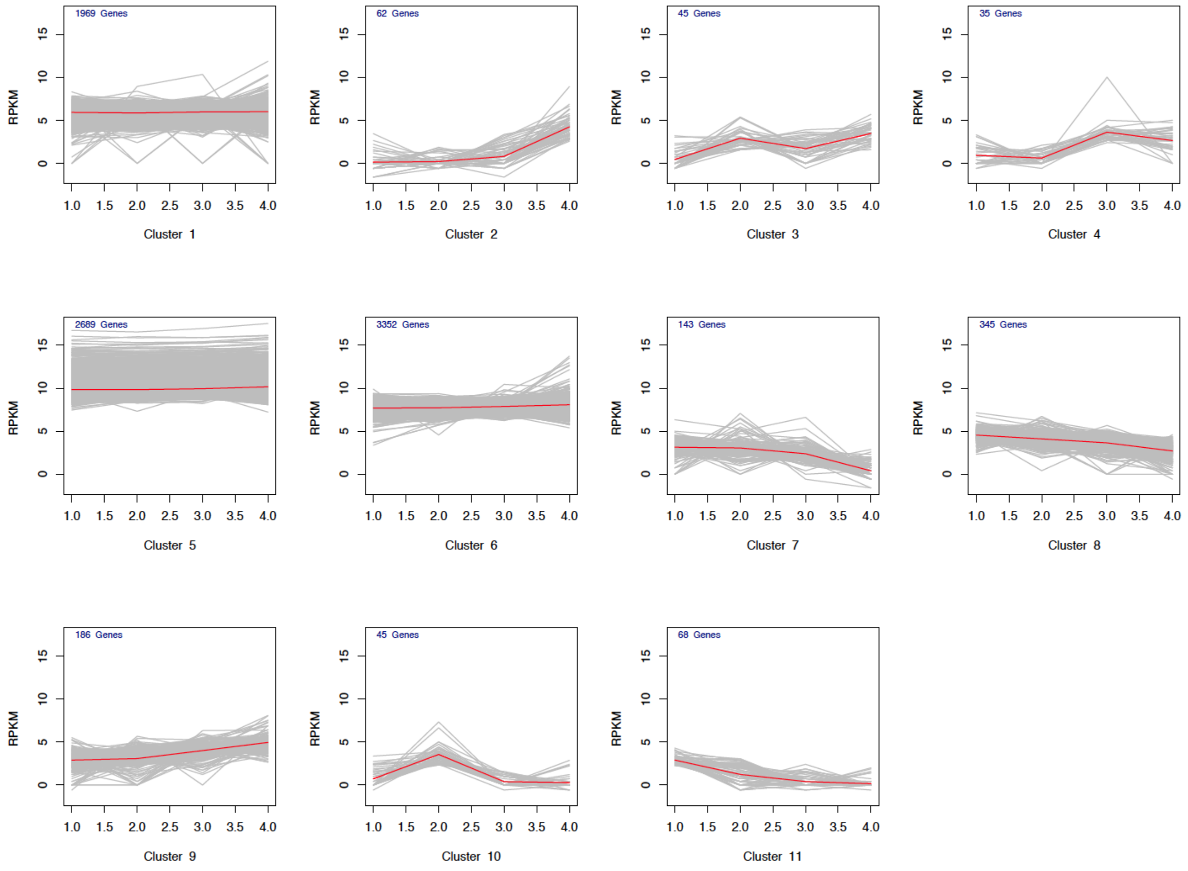

Supplementary Fig. S9

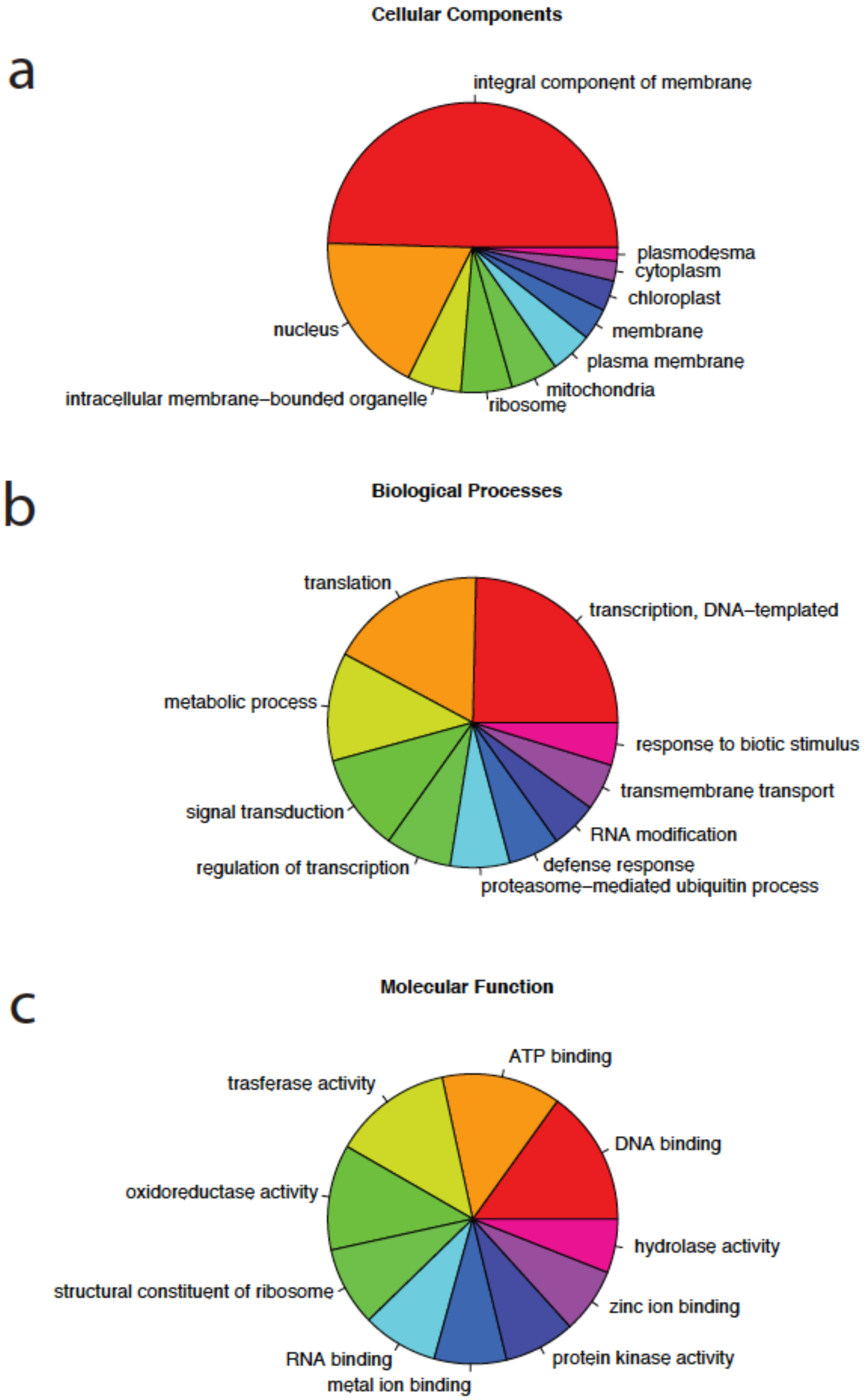

Supplementary Fig. S10

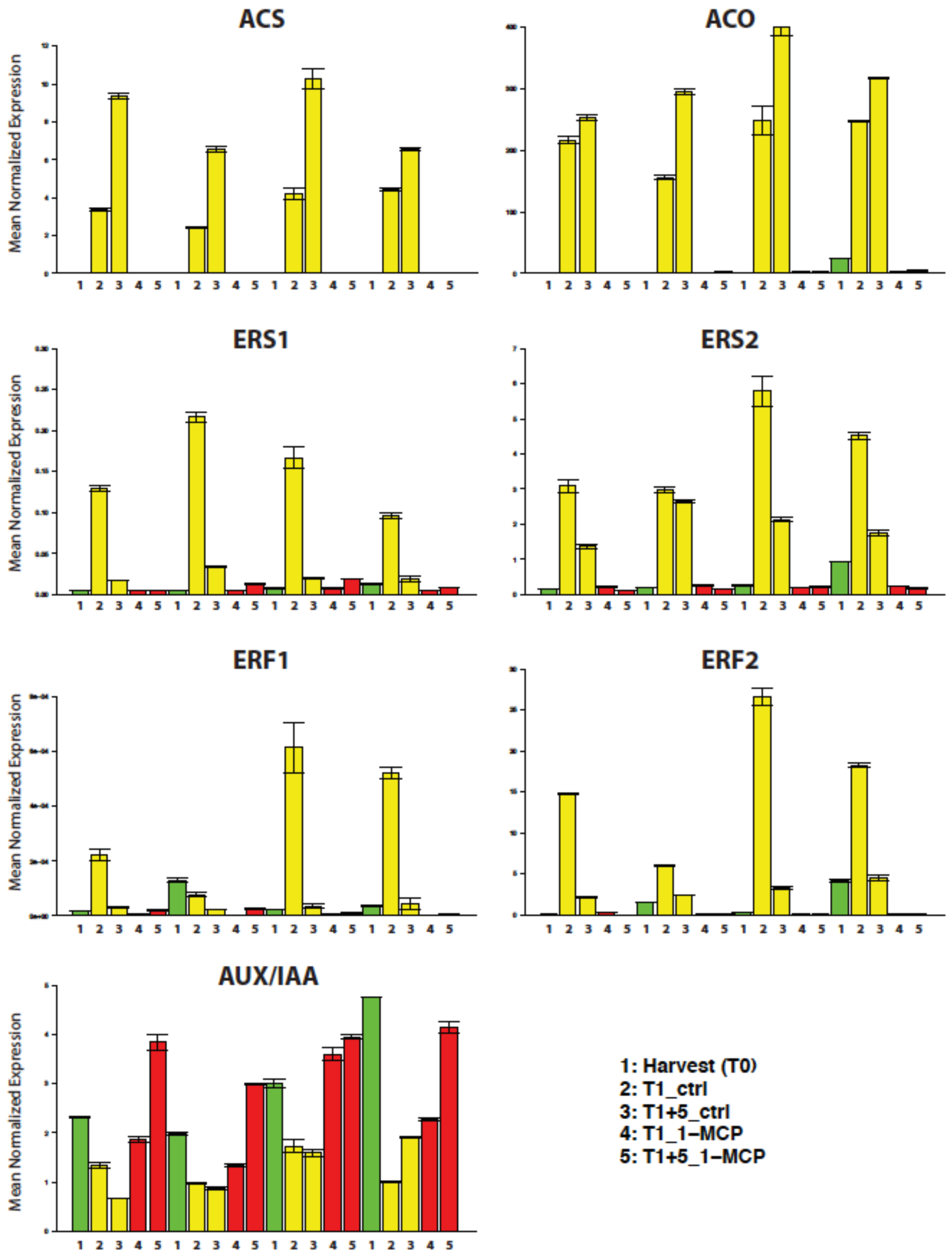

Supplementary Fig. S11

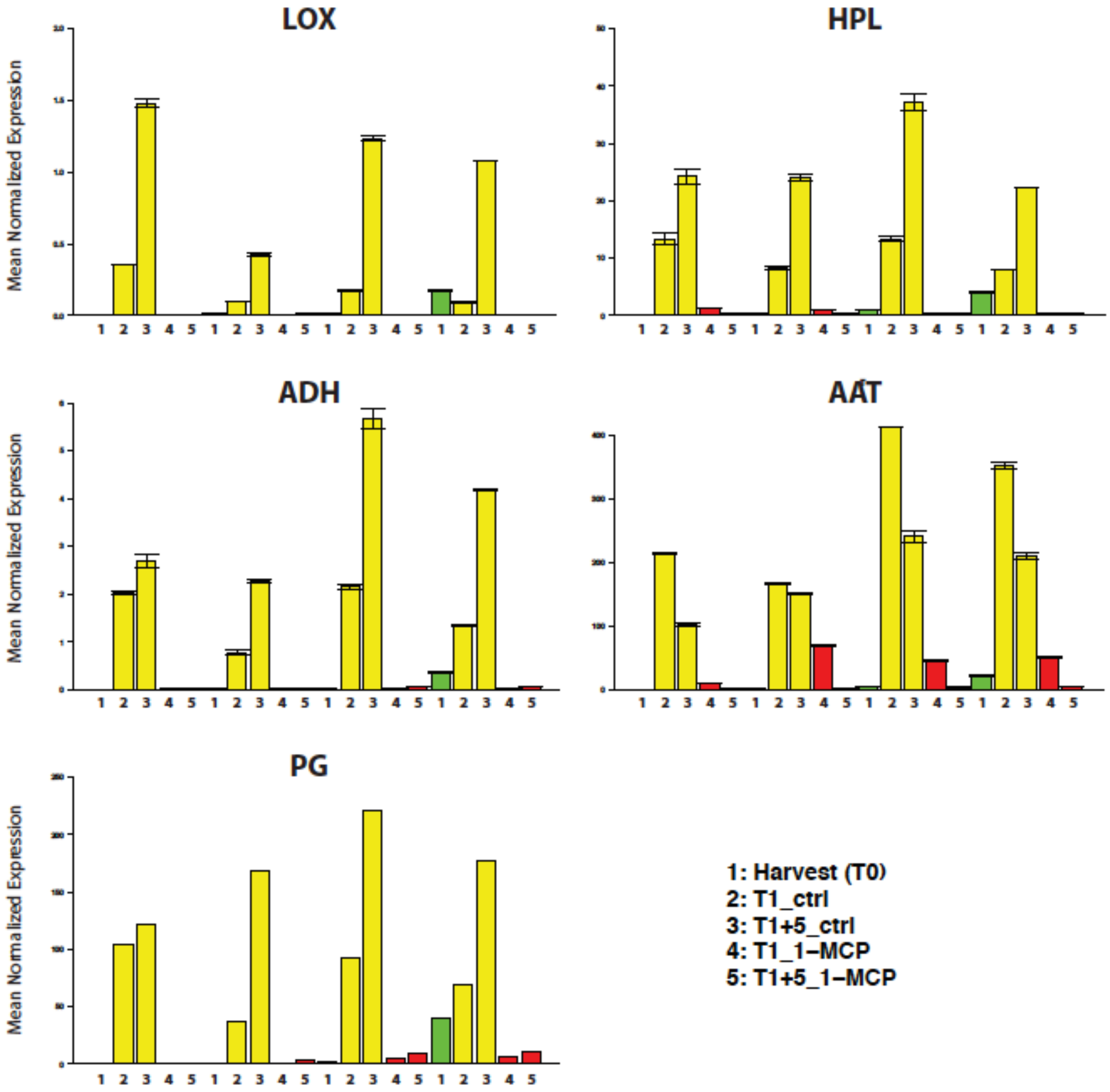

Supplementary Fig. S12

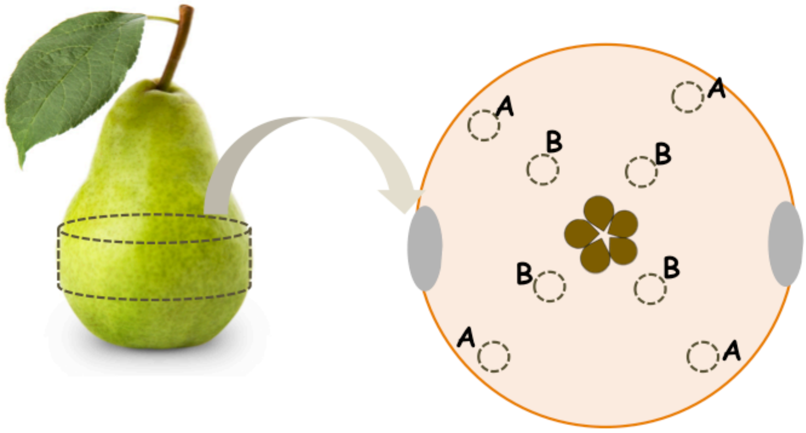

Supplementary Table S1

m/z	Formula	Identification
29,0404	C2H5	ethanol fragment
31,0182	CH2OH	Formaldehyde
33,033	CH4OH	methanol
34,9951	H2SH	Hydrogen sulfide
35,0381		
39,0234	C3H3	common fragment
41,0384	C3H5	common fragment
42,0419		
43,0164	C2H3O	common fragment
43,0542	C3H7	common fragment
45,0329	C2H4OH	Acetaldehyde
47,0478	C2H6OH	Ethanol
49,0117	CH4SH	Methanethiol
49,0538	[13]C2H6OH	second isotope of Ethanol
51,0431	CH4O*H3O	methanol cluster
53,0392	C4H5	
55,0543	C4H7	common fragment
57,0335	C3H4OH	common fragment
57,0699	C4H9	1-Butanol, high alcohol fragment
59,0493	C3H6OH	Acetone
61,0285	C2H4O2H	Acetic acid, Acetoin, common ester fragment
63,0396	C2H6O2H	Ethylene glycol
67,0548	C5H7	
69,0336	C4H4OH	furan
69,0705	C5H9	isoprene, terpene fragment
71,0135		
71,0497	C4H6OH	Esters (Ethyl butanoate, Ethyl hexanoate, Propyl butanoate, Butyl butanoate, 2-Methylbutyl acetate, Isoamyl acetate)
71,086	C5H11	3-methyl-1-butanol 2-methyl-1-butanol, 3-Pentanol, 1-Pentanol
73,0286	C3H5O2	
73,0651	C4H8OH	butanal, isobutyraldehyde
75,0424	C3H6O2H	Methyl Acetate
77,0225		
77,0499	C3H8O2H	
78,0469		
79,0413	C2H6O3O	
81,0707	C6H9	Fragment of aldehydes (Hexenals); fragment of terpenes
83,0503	C5H6OH	Methylfuran
83,0866	C6H11	(E)-3-Hexen-1-ol, (Z)-3-Hexen-1-ol, (Z)-2-Hexen-1-ol, Hexanal,2-Hexanone
85,0285	C4H5O2	2(5H)-Furanone
85,0658	C5H8OH	
85,1025	C6H13	Hexanol
87,0446	C4H6O2H	Butyrolactone
87,0813	C5H10OH	2-methyl butanal,3- methyl butanal, Pentanal
88,0474		
88,957		
89,0592	C4H8O2H	isotope of Ethyl Acetate, Butanoic Acid
89,9502		
91,0585	C7H7	Benzyl Alcohol

93,0374	C3H8OSH	
93,0722	C7H9	Toluene, Monoterpene fragment
95,0202		
95,0482	C6H7O	phenol
95,0869	C7H11	2-Heptenal, Monoterpene fragment
97,0657	C6H8OH	2,4-Hexadienal
97,1026	C7H13	Heptanal, fragment
99,0449	C5H7O2	5-Methylfuran-2(3H)-one
99,0816	C6H10OH	2-Hexenal, (2E)-Hexenal
101,0241		
101,0608	C5H8O2H	2,3-Pentanedione
101,0967	C6H12OH	Hexanal, hexanone
103,0397		
103,0759	C5H10O2H	Isovaleric Acid, Pentanoic Acid
105,0226		
105,0482		
105,0712	C8H9	Styrene
107,0559		
107,0844	C8H10H	Ethyl Benzene, Xylene
108,9572		
109,1032	C8H13	
111,0395		
111,0815	C7H10OH	Heptadienal
111,1185	C8H15	Octanal, Octenol
113,0239		
113,0596	C6H8O2H	Sorbic acid
113,0977	C7H12OH	Heptenal, heptanone
115,0393		
115,0768	C6H10O2H	Ethyl Crotonate (Ethyl (2E)-2-butenoate), 5-Ethyldihydro-2(3H)-Furanone
115,1127	C7H14OH	heptanal/heptanone
117,0912	C6H12O2H	Isobutyl Acetate, Butyl Acetate, Hexanoic Acid
119,0877	C9H11	
121,0661	C8H8OH	Phenylacetaldehyde
121,1029	C9H13	
123,0469	C4H10O2S	
123,1189	C9H15	nonenal
125,0605	C7H8O2H	Guaiacol
125,0984	C8H12OH	
125,1343	C9H17	nonanal, nonenol
127,0385		
127,0775	C7H10O2H	2(3H)-Furanone, dihydro-5-methyl-5-vinyl
127,1142	C8H14OH	1-octen-3-one, 6-Methyl-5-Hepten-2-one, (E)-2-Octenal
129,0559		
129,092	C7H12O2H	
129,1295	C8H16OH	2-octanone, Octanal, 1-Octen-3-ol
131,0354		
131,0742		
131,1078	C7H14O2H	Isoamyl Acetate, Benzene, Amyl Acetate, Heptanoic Acid
135,1153	C10H15	HO-Trienol, trans-Carveol
137,1352	C10H17	monoterpenes, fragment of sesquiterpenes
139,1146	C9H14OH	
144,9144		
145,0519		
145,1236	C8H16O2H	Ethyl Hexanoate, Hexyl Acetate, 2-Ethyl Hexanoic Acid
149,0835		
149,1331	C11H17	

151,1015		
153,0577		
153,1272	$C_{10}H_{16}OH$	HO-Trienol, Epoxylinalool, 2,4-Decadienal , 2,6-dimethyl-3,7-octadiene-2,6-diol
159,1426	$C_9H_{18}O_2H$	Nonanoic acid
167,0633	$C_{10}H_{15}O_2$	
205,2006	$C_{15}H_{24}$	sesquiterpenes, farnesene

Supplementary Table S2

Cluster_1

Gene	H1	H2	H3	H4		
PCP000026	4,841469999	4,922673593	5,426264755	6,143026004	PCP000026	XM_008242675
PCP000057	6,464831606	5,77267747	6,215872703	5,222263604	PCP000057	XM_018645828
PCP000072	7,044394119	6,695854658	6,68187088	6,700439718		
PCP000075	6,938638658	6,785681319	6,539158811	6,62935662	PCP000075	XM_009358719
PCP000093	5,333781501	5,64385619	5,781359714	6,31596467	PCP000093	XM_008375290
PCP000099	5,058749412	5,48123435	5,681730355	6,156437068	PCP000099	XM_009375193
PCP000116	4,624685811	5,02989455	5,946496941	6,291493053	PCP000116	XM_018651208
PCP000121	6,397974049	6,375039431	6,272956308	6,051589621	PCP000121	XM_008375327
PCP000144	5,914803677	5,77267747	6,828707735	6,31596467	PCP000144	XM_009378003
PCP000166	5,357552005	5,357552005	5,700439718	6,375039431	PCP000166	XR_001790963
PCP000168	5,115615931	5,403608584	4,938756261	4,437627248	PCP000168	XM_008381123
PCP000202	6,272956308	5,977279923	6,122465658	5,815831566	PCP000202	XM_009339734
PCP000205	5,544423562	5,321928095	5,209453366	4,73714592		
PCP000219	6,203005338	6,539158811	6,594996337	7,108524457	PCP000219	XM_008347515
PCP000221	6,662917555	6,62935662	7,058857621	6,709704193	PCP000221	XM_009344708
PCP000226	5,333781501	5,101397952	4,890933022	5,882643049	PCP000226	XM_018643286
PCP000248	6,291493053	6,215872703	5,718909554	4,414812061	PCP000248	XM_008380910
PCP000261	6,574858391	7,033423002	6,874428132	6,609843592		
PCP000262	4,874305166	5,64385619	6,653490009	6,279285561		
PCP000336	5,789990019	4,480911346	5,718909554	4,772413555	PCP000336	XM_008358112
PCP000359	5,260402093	5,285402219	5,321928095	4,459431619	PCP000359	XM_009371788
PCP000374	6,448405435	6,62935662	6,882643049	7,183188734	PCP000374	XM_009360918
PCP000377	5,634302842	6,564835417	6,614709844	7,684187561		
PCP000385	7,176621973	6,815959618	6,448405435	6,209453366	PCP000385	XM_008341850
PCP000393	6,448405435	7,139551352	6,285402219	5,101397952		
PCP000396	6,426264755	6,051589621	5,754887502	6,094447222	PCP000396	XM_008391439
PCP000429	7,018700931	6,798698597	6,677085351	6,518377768		
PCP000459	6,196331634	6,414981143	5,345893086	5,087462841	PCP000459	XM_009353724
PCP000479	4,890933022	5,014801602	4,841469999	5,789990019		
PCP000491	6,50779464	6,369291982	6,448405435	6,14974712		
PCP000492	6,333960351	6,196331634	6,523561956	7,051698368		
PCP000505	5,087462841	5,209453366	5,754887502	6,101608059		
PCP000507	6,539158811	6,266786541	6,397974049	6,196331634	PCP000507	XM_009364758
PCP000513	6,72342204	6,38663853	6,700439718	6,369291982		
PCP000523	7,132679654	6,942514505	6,624539604	6,448405435	PCP000523	XM_009360558
PCP000535	5,984817174	5,380937195	4,969472865	4,906890596	PCP000535	XM_009360579
PCP000571	5,369117459	4,857980995	5,087462841	4,700439718		
PCP000585	5,969703088	6,122465658	6,363346321	7,196430001		
PCP000592	6,051589621	5,781359714	5,59484709	3,938285792	PCP000592	XM_009371384
PCP000595	6,07317756	6,327866971	5,709566354	4,824258697	PCP000595	XM_009371379
PCP000616	6,03716255	5,533874777	6,235152624	6,653490009	PCP000616	XM_008364789
PCP000652	5,815831566	6,169925001	6,321928095	6,853870927	PCP000652	XM_009373896

PCP000675	5,309976492	5,746043983	6,579994693	6,303780748		
PCP000686	5,196528361	4,772413555	5,073391816	4,169925001	PCP000686	XM_008368431
PCP000690	7,554588852	6,763809907	5,48123435	2,503348735	PCP000690	XM_008380532
PCP000733	5,156639311	3,700439718	4,414812061	5,824513297	PCP000733	XM_009370513
PCP000736	6,798698597	6,68187088	6,609843592	6,272956308		
PCP000771	6,007419784	5,614709844	5,513174885	5,285402219	PCP000771	XM_008380613
PCP000857	3,321928095	4,718635616	5,115615931	5,285402219		
PCP000901	4,890933022	5,222263604	5,841218374	5,849749117	PCP000901	XM_018647720
PCP000916	6,297741678	6,189824559	6,910852562	6,768184325		
PCP000948	6,84962403	5,247927513	5,824513297	5,392317423	PCP000948	XM_009375113
PCP000979	6,345715709	6,677085351	6,579994693	6,235152624	PCP000979	XM_018642479
PCP000989	6,247927513	5,961854808	5,992541859	5,798828178		
PCP001044	6,015024705	6,345715709	6,95419631	7,235248379	PCP001044	XM_009376771
PCP001065	6,291493053	6,351734323	6,397974049	7,173227395	PCP001065	XR_528319
PCP001078	6,266786541	6,363346321	6,87036472	7,209453366	PCP001078	XM_008353511
PCP001084	6,297741678	6,375039431	6,209453366	5,369117459	PCP001084	XM_009361128
PCP001116	6,163297449	6,279285561	6,497133304	5,754887502	PCP001116	XM_009381049
PCP001143	6,380764075	5,309976492	5,533874777	5,882643049	PCP001143	XM_008342459
PCP001165	6,534030467	6,62935662	6,513016928	6,321928095	PCP001165	XM_009345168
PCP001166	5,709566354	6,718772592	6,215872703	5,672425342		
PCP001172	6,459431619	6,06608919	6,080444483	5,584962501	PCP001172	XM_018647789
PCP001176	5,914803677	6,163297449	6	7,247927513	PCP001176	XM_009344504
PCP001183	6,807354922	6,14974712	6,351734323	6,222456826	PCP001183	XM_009348813
PCP001209	3,663344619	5,309976492	4,790250739	5,914803677		
PCP001236	5,169925001	5,101397952	6,266786541	5,437294331	PCP001236	XM_009341441
PCP001270	7,037052702	6,579994693	6,564835417	6,481072857		
PCP001291	6,414981143	6,143026004	5,95419631	5,02989455	PCP001291	XM_009363494
PCP001299	6,750472519	6,38663853	6,981053471	6,574858391	PCP001299	XM_009358665
PCP001300	6,266786541	5,922911406	6,327866971	6,86628983	PCP001300	XM_009358666
PCP001313	6,60481265	5,866166169	5,564683017	4,857980995	PCP001313	XM_009370302
PCP001334	4,969472865	5,101397952	4,029452886	4,222650022	PCP001334	XM_009362985
PCP001360	6,03716255	5,807354922	5,815831566	6,589913261	PCP001360	XM_009335658
PCP001362	5,087462841	5,142821844	6,837060204	6,653490009	PCP001362	XM_018642470
PCP001389	5,403608584	5,906890596	7,398059585	6,513016928	PCP001389	XM_008390000
PCP001399	5,101397952	5,502394256	5,48123435	6,07317756	PCP001399	XM_009349029
PCP001400	4,297925053	5,260402093	6,163297449	5,48123435	PCP001400	XM_018645401
PCP001421	6,196331634	6,044394119	5,533874777	3,906890596	PCP001421	XM_017332283
PCP001434	6,321928095	6,339850003	6,327866971	5,807354922	PCP001434	XM_018644074
PCP001453	4,029452886	5,101397952	5,426264755	4,321928095	PCP001453	XM_020556076
PCP001489	6,619559738	5,961854808	5,807354922	5,672425342		
PCP001506	6,80309785	6,820178962	6,828707735	6,534030467	PCP001506	XM_009336436
PCP001513	6,24164954	5,781359714	5,832890014	6,84962403	PCP001513	XM_009344464
PCP001524	6,31596467	6,497133304	6,589913261	7,206232954		
PCP001593	6,029673735	5,914803677	6,475733431	7,423494135	PCP001593	XM_009337756
PCP001618	6,303780748	6,327866971	5,961854808	5,544423562		
PCP001681	6,339850003	6,266786541	6,62935662	6,958030641	PCP001681	XM_009351542

PCP001711	6,051589621	6,22881869	6,695854658	7,029784146	PCP001711	XR_666319
PCP001736	6,409390936	6,564835417	6,658211483	7,105070402	PCP001736	XM_017336725
PCP001741	6,828707735	7,22881869	6,22881869	5,815831566	PCP001741	XM_009378118
PCP001747	6	6,260590275	6,339850003	6,81159947		
PCP001755	5,64385619	5,634302842	5,824513297	6,491853096	PCP001755	XM_009338375
PCP001822	5,235344128	5,691254979	6,464831606	6,475733431	PCP001822	XM_009357987
PCP001823	4,64385619	5,196528361	5,235344128	4,459431619		
PCP001867	5,57500972	4,624685811	6,06608919	4,604664415	PCP001867	XM_018649989
PCP001872	6,709704193	6,961970533	6,409390936	5,815831566	PCP001872	XM_018649994
PCP001904	5,798828178	5,832890014	6,426264755	6,862079387	PCP001904	XM_008357041
PCP001959	5,60496087	6,156437068	5,624393382	4,64385619	PCP001959	XM_009372167
PCP001991	6,122465658	6,07317756	6,38663853	7,111970261	PCP001991	XM_009348250
PCP002005	6,709704193	6,68187088	6,491853096	7,087462841		
PCP002029	6,297741678	6,291493053	5,961854808	5,653346693	PCP002029	XM_008374446
PCP002050	6,841344192	6,518377768	6,523561956	6,454011343	PCP002050	XM_008380541
PCP002056	3,663344619	7,336908182	4,297925053	8,385129006		
PCP002059	4,544114402	5,448570626	5,222263604	7,269874722	PCP002059	XM_009363568
PCP002071	6,50779464	6,80309785	6,369291982	6,03716255		
PCP002073	6,247927513	6,727920455	6,68187088	6,442943496	PCP002073	XM_008346541
PCP002090	7,139551352	6,653490009	6,874428132	6,554588852		
PCP002101	5,48123435	5,857980995	5,832890014	5,369117459	PCP002101	XM_009374978
PCP002204	6,481072857	6,122465658	6,403778984	5,663059924		
PCP002226	6,977279923	6,143026004	6,272956308	6,31596467	PCP002226	XM_009343017
PCP002233	6,95419631	6,029673735	6,47037417	5,681730355	PCP002233	XM_009343008
PCP002260	6,260590275	6,22881869	6,203005338	5,866166169	PCP002260	XM_018651419
PCP002264	6,523561956	6,272956308	6,518377768	5,763677142	PCP002264	XM_009376088
PCP002281	5,333781501	4,64385619	5,691254979	6,156437068	PCP002281	XM_009353152
PCP002307	6,101608059	5,736875423	4,523561956	3,502075956	PCP002307	XM_009356432
PCP002308	3,544732656	4,247927513	4,790250739	5,544423562	PCP002308	XM_008376929
PCP002320	4,321928095	4,297925053	4,807354922	7,176621973	PCP002320	XM_009356413
PCP002354	6,639087423	6,403778984	6,72342204	7,115511897	PCP002354	XM_009365934
PCP002359	5,922911406	6,491853096	6,38663853	6,080444483		
PCP002363	5,129283017	5,129283017	5,584962501	6,297741678	PCP002363	XM_008370205
PCP002370	6,874428132	6,333960351	5,554588852	4,857980995	PCP002370	XM_009351585
PCP002371	6,737010678	6,785681319	6,481072857	6,50779464	PCP002371	XM_017324171
PCP002387	6,910852562	6,454011343	6,481072857	5,938521046	PCP002387	XM_008359999
PCP002389	6,946379968	6,54951516	6,594996337	5,754887502		
PCP002390	5,260402093	5,807354922	5,930737338	7,754887502		
PCP002403	6,209453366	5,984817174	6,667750232	7,651051691	PCP002403	XM_017331441
PCP002406	7,037052702	6,714245518	5,946496941	5,129283017	PCP002406	XM_009336338
PCP002440	6,058965822	6,291493053	6,235152624	5,614709844	PCP002440	XM_009375283
PCP002447	6,47037417	5,459431619	6,183288001	5,437294331		
PCP002453	5,02989455	5,898933872	4,906890596	4,029452886	PCP002453	XM_009349390
PCP002461	6,209453366	6,176522247	6,486553493	6,996275749	PCP002461	XM_009381151
PCP002504	4,059182199	4,369466484	6,409390936	4,985044962	PCP002504	XM_008352334
PCP002508	6,209453366	5,736875423	5,544423562	5,614709844	PCP002508	XM_008390628

PCP002510	6,442943496	6,741466986	6,653490009	6,279285561	PCP002510	XR_001788592
PCP002575	6,029673735	6,260590275	6,357552005	6,785681319	PCP002575	XM_009350276
PCP002599	5,101397952	6,176522247	5,969703088	5,634302842	PCP002599	XM_009336313
PCP002627	4,969472865	5,183089461	4,754887502	4,143230135	PCP002627	XM_018647160
PCP002653	5,914803677	6,094447222	6,143026004	6,667750232		
PCP002675	5,415150205	5,866166169	6,882643049	7,794415866	PCP002675	XM_009348026
PCP002684	5,285402219	5,634302842	5,115615931	4,841469999	PCP002684	XM_009380652
PCP002696	5,297558281	5,309976492	5,584962501	6,339850003	PCP002696	XM_009342219
PCP002709	6,60481265	6,754887502	6,87036472	6,392317423	PCP002709	XM_008358628
PCP002752	6,24164954	5,544423562	5,798828178	3,874796966		
PCP002771	4,369466484	4,222650022	5,403608584	5,824513297	PCP002771	XM_018650289
PCP002793	6,03716255	6,47037417	6,426264755	7,037052702		
PCP002802	6,828707735	6,794415866	6,87036472	6,254178286	PCP002802	XM_009357952
PCP002806	6,624539604	6,574858391	6,497133304	6,06608919	PCP002806	XM_018647437
PCP002819	6,523561956	6,648609245	6,902917719	7,186559982	PCP002819	XM_009344515
PCP002833	5,073391816	4,906890596	5,491853096	6,247927513	PCP002833	XM_009343223
PCP002841	6,539158811	6,38663853	6,718772592	7,125878364	PCP002841	XM_009337643
PCP002857	6,369291982	5,961854808	5,866166169	5,369117459	PCP002857	XM_009335975
PCP002865	5,874551087	6,222456826	5,614709844	4,604664415		
PCP002875	6,554588852	6,54951516	5,48123435	5,513174885	PCP002875	XM_018650824
PCP002905	5,746043983	5,415150205	4,523561956	3,169925001	PCP002905	XM_018651546
PCP002908	6,942514505	6,686500527	6,906890596	6,491853096	PCP002908	XR_001791436
PCP002910	6,528727582	6,705010253	6,938638658	6,491853096		
PCP002931	7,400879436	7,186559982	6,777025123	4,890933022	PCP002931	XM_009378477
PCP003006	5,700439718	5,448570626	5,285402219	4,790250739		
PCP003025	5,142821844	5,426264755	5,882643049	4,34553831	PCP003025	XM_009370219
PCP003027	5,369117459	5,882643049	5,849749117	6,486553493		
PCP003151	6,327866971	5,781359714	5,345893086	4,222650022	PCP003151	XM_009363495
PCP003185	6,946379968	7,018700931	7,115511897	6,260590275	PCP003185	XM_008386843
PCP003222	5,333781501	6,058965822	5,448570626	5,014801602	PCP003222	XM_008363041
PCP003237	5,369117459	4,922673593	5,297558281	4,624685811	PCP003237	XM_018651512
PCP003293	6,06608919	5,849749117	6,327866971	5,614709844	PCP003293	XM_008391312
PCP003311	6,260590275	6,574858391	6,741466986	6,996275749	PCP003311	XM_009362099
PCP003312	6,667750232	6,31596467	6,785681319	7,139551352		
PCP003339	5,333781501	5,807354922	6,087462841	6,189824559	PCP003339	XM_009362215
PCP003341	7,090959258	6,62935662	6,414981143	6		
PCP003365	6,777025123	6,828707735	6,403778984	5,169925001		
PCP003373	6,420718183	5,709566354	5,946496941	5,849749117	PCP003373	XM_009376412
PCP003397	4,890933022	5,321928095	4,544114402	4,029452886	PCP003397	XM_008389543
PCP003402	5,345893086	5,333781501	6,72342204	8,110248388		
PCP003418	6,559644763	5,906890596	5,426264755	6,442943496	PCP003418	XM_009361276
PCP003430	7,018700931	6,539158811	6,662917555	6,705010253	PCP003430	XM_009369095
PCP003461	5,930737338	6,594996337	6,890811455	7,437377568	PCP003461	XM_009369144
PCP003462	6,176522247	6,176522247	7,026025399	7,094552786	PCP003462	XM_009369145
PCP003470	6,080444483	6,058965822	6,203005338	5,333781501		
PCP003475	5,64385619	5,554588852	5,48123435	4,772413555	PCP003475	XM_009376835

PCP003478	5,807354922	5,309976492	5,437294331	4,222650022	PCP003478	XM_009378939
PCP003499	6,894817763	6,700439718	6,589913261	6,481072857	PCP003499	XM_009378911
PCP003511	6,569855608	6,798698597	6,448405435	6,47037417		
PCP003649	6,058965822	6,420718183	6,559644763	7,315874125	PCP003649	XM_009373744
PCP003650	6,303780748	6,518377768	6,745909573	7,062531903	PCP003650	XM_009373748
PCP003676	6,015024705	6,272956308	6,442943496	7,453929061		
PCP003679	6,420718183	7,30679083	6,574858391	6,930737338	PCP003679	XM_009347215
PCP003740	5,297558281	4,392317423	4,95419631	3,700439718	PCP003740	XM_009350469
PCP003772	6,559644763	6,777025123	6,732404887	6,442943496	PCP003772	XM_009347011
PCP003786	7,026025399	6,862079387	6,958030641	5,930737338	PCP003786	XM_009368794
PCP003789	5,380937195	5,824513297	6,176522247	6,609843592	PCP003789	XM_008361041
PCP003836	6,235152624	6,426264755	6,309794644	7,040782866	PCP003836	XM_009363653
PCP003838	6,176522247	5,961854808	6,380764075	6,732404887	PCP003838	XM_009363654
PCP003848	6,403778984	6,442943496	6,513016928	6,044394119	PCP003848	XM_008366514
PCP003869	6,222456826	6,309794644	6,454011343	7,007531912	PCP003869	XM_009367429
PCP003894	6,958030641	6,528727582	6,209453366	4,922673593		
PCP003897	4,480911346	5,57500972	4,938756261	6,044394119	PCP003897	XM_009366844
PCP003989	6,339850003	5,789990019	5,866166169	5,789990019	PCP003989	XM_009371046
PCP003997	6,357552005	6,169925001	6,464831606	7,033423002	PCP003997	XM_009371069
PCP004090	5,459431619	5,736875423	5,60496087	5,014801602	PCP004090	XM_018645966
PCP004102	6,714245518	6,044394119	6,918863237	6,837060204		
PCP004106	6,658211483	6,414981143	6,513016928	5,709566354	PCP004106	XM_009370239
PCP004114	6,279285561	6,247927513	5,849749117	5,533874777	PCP004114	XM_009376322
PCP004115	6,426264755	6,50779464	6,426264755	5,992541859	PCP004115	XM_009376320
PCP004153	5,058749412	5,746043983	6,129283017	6,183288001	PCP004153	XM_009366733
PCP004172	6,705010253	5,914803677	6,459431619	6,981053471	PCP004172	XM_009356453
PCP004203	5,448570626	5,824513297	6,222456826	6,841344192	PCP004203	XM_008344440
PCP004206	6,820178962	6,62935662	5,961854808	5,273142859	PCP004206	XM_009355920
PCP004210	6,763809907	6,619559738	6,62935662	6,115407855	PCP004210	XM_018647005
PCP004215	6,101608059	6,189824559	6,189824559	5,235344128	PCP004215	XM_009355933
PCP004272	6,291493053	6,094447222	6,203005338	5,357552005	PCP004272	XM_009375832
PCP004304	6,380764075	6,203005338	6,579994693	6,101608059	PCP004304	XM_008344062
PCP004311	4,459431619	3,663344619	4,087462841	6,837060204	PCP004311	XM_009341828
PCP004327	5,798828178	5,470211457	5,502394256	4,544114402	PCP004327	XM_009373142
PCP004333	6,357552005	6,50255338	5,984817174	5,922911406	PCP004333	XM_009373127
PCP004334	7,22881869	6,47037417	6,737010678	6,215872703	PCP004334	XM_017330527
PCP004346	5,672425342	5,984817174	5,169925001	5,491853096	PCP004346	XM_008382241
PCP004357	6,409390936	5,437294331	6,272956308	5,977279923		
PCP004358	5,115615931	5,357552005	5,60496087	6,215872703	PCP004358	XM_008370675
PCP004369	6,409390936	5,781359714	5,491853096	5,058749412		
PCP004451	4,604664415	4,790250739	5,554588852	6,619559738	PCP004451	XM_008347000
PCP004464	5,969703088	6,914923239	6,942514505	6,910852562	PCP004464	XM_009376766
PCP004528	5,142821844	6,426264755	5,866166169	4,754887502		
PCP004569	4,824258697	4,857980995	4,938756261	6,624539604	PCP004569	XM_009366988
PCP004598	6,481072857	6,454011343	6,634157606	7,215969746	PCP004598	XM_009351172
PCP004622	4,059182199	6	5,961854808	4,196134881	PCP004622	XM_008359674

PCP004639	5,798828178	5,57500972	5,437294331	5,183089461	PCP004639	XM_018645158
PCP004651	4,857980995	4,321928095	5,437294331	4,087462841	PCP004651	XM_008391856
PCP004678	6,902917719	6,95419631	6,614709844	6,639087423		
PCP004689	6,95419631	6,569855608	6,759289016	6,68187088	PCP004689	XM_008384807
PCP004738	6,554588852	6,584962501	6,763809907	6,254178286	PCP004738	XM_018647237
PCP004748	6,87036472	6,321928095	5,672425342	5,02989455	PCP004748	XM_009374004
PCP004768	5,824513297	5,115615931	5,746043983	4,824258697	PCP004768	XM_018644055
PCP004772	5,459431619	4,857980995	4,938756261	4,584962501	PCP004772	XM_017325187
PCP004776	5,513174885	5,857980995	6,824386003	7,938579853	PCP004776	XM_009348478
PCP004842	6,754887502	6,420718183	6,777025123	6,06608919	PCP004842	XM_018651285
PCP004846	5,115615931	4,857980995	4,700439718	4	PCP004846	XM_008347637
PCP004874	5,807354922	4,392317423	3,584962501	5,426264755	PCP004874	XM_009373739
PCP004900	6,737010678	6,454011343	6,327866971	6,297741678	PCP004900	XM_009361004
PCP004915	5,014801602	5,564683017	6,058965822	5,523561956	PCP004915	XM_009356579
PCP004957	6,189824559	6,101608059	5,969703088	5,554588852	PCP004957	NM_001302294
PCP004991	6,156437068	6,260590275	6,459431619	6,981053471	PCP004991	XM_008345811
PCP005012	4,790250739	5,235344128	5,156639311	5,961854808		
PCP005035	6,589913261	6,363346321	6,22881869	5,977279923	PCP005035	XM_009363354
PCP005066	5,961854808	5,59484709	5,415150205	4,922673593		
PCP005117	6,934634441	6,86628983	6,333960351	6,922792504	PCP005117	XM_009360370
PCP005141	7,30679083	7,026025399	6,589913261	5,781359714	PCP005141	XM_009369418
PCP005142	6,297741678	6,60481265	6,705010253	7,363433935	PCP005142	XM_009369420
PCP005190	5,857980995	5,59484709	5,866166169	6,599912842	PCP005190	XM_018650258
PCP005202	6,750472519	6,653490009	6,420718183	6,303780748		
PCP005204	6,272956308	6,363346321	6,122465658	6,815959618	PCP005204	XM_008358409
PCP005219	6,459431619	6,094447222	6,380764075	6,938638658	PCP005219	XM_008376356
PCP005226	7,06608919	6,841344192	6,727920455	6,763809907	PCP005226	XM_009340843
PCP005233	6,363346321	6,534030467	6,815959618	7,403693786		
PCP005239	6,345715709	6,845490051	6,745909573	7,348728154	PCP005239	XM_009353433
PCP005244	6,54426899	5,824513297	6,677085351	6,50779464	PCP005244	XM_008342230
PCP005259	6,906890596	6,898812977	6,589913261	6,357552005	PCP005259	XM_018646464
PCP005278	6,481072857	6,513016928	6,285402219	5,882643049	PCP005278	XM_009380215
PCP005280	6,247927513	5,992541859	6,72342204	7,129283017	PCP005280	XM_009380218
PCP005325	6,910852562	6,961970533	6,50779464	5,321928095	PCP005325	XM_009369266
PCP005371	6,80309785	6,772545519	7,442943496	6,426264755	PCP005371	XM_009380179
PCP005413	6,044394119	5,437294331	5,222263604	4,392317423		
PCP005424	4,890933022	5,584962501	6,101608059	10,18818083	PCP005424	XM_009357891
PCP005447	5,992541859	6,196331634	5,653346693	5,209453366	PCP005447	XM_018652102
PCP005455	6,763809907	6,409390936	6,653490009	6,321928095		
PCP005525	5,832890014	5,807354922	6,101608059	5,183089461		
PCP005533	5,857980995	6	6,528727582	5,691254979	PCP005533	XM_009375460
PCP005539	4,985044962	4,584962501	5,357552005	5,866166169	PCP005539	XM_009375464
PCP005553	5,969703088	5,789990019	6,051589621	5,513174885	PCP005553	XM_008365851
PCP005588	6,481072857	6,539158811	6,235152624	6,209453366	PCP005588	XM_009357298
PCP005592	0,739848103	5,746043983	7,745976779	11,87203226	PCP005592	XM_008364028
PCP005594	0	5,763677142	6,03716255	7,139551352	PCP005594	XM_008372022

PCP005602	6,203005338	6,266786541	6,50255338	5,95419631		
PCP005603	6,309794644	6,07317756	5,961854808	6,80309785	PCP005603	XM_009378153
PCP005613	4,297925053	4,790250739	5,087462841	5,672425342	PCP005613	XM_009352209
PCP005629	6,38663853	6,86628983	6,977279923	7,118941073	PCP005629	XR_524428
PCP005639	5,470211457	5,64385619	4,718635616	4,437627248	PCP005639	XM_008366510
PCP005647	5,77267747	5,491853096	5,459431619	5,222263604	PCP005647	XM_009338170
PCP005667	6,254178286	6,297741678	6,807354922	6,946379968	PCP005667	XM_009340274
PCP005688	6,709704193	6,594996337	6,497133304	6,169925001	PCP005688	XM_009346840
PCP005697	5,841218374	6,481072857	6,579994693	6,862079387		
PCP005700	6,513016928	6,54426899	6,64385619	7,351645995	PCP005700	XM_009352107
PCP005701	4,700439718	4,790250739	5,866166169	6	PCP005701	XM_008365769
PCP005714	4,544114402	4,906890596	4,824258697	5,672425342		
PCP005755	6,291493053	6,297741678	6,695854658	7,354646096	PCP005755	XM_009357844
PCP005775	6,639087423	6,539158811	6,584962501	6,351734323		
PCP005852	4,841469999	4,604664415	3,938285792	8,336863563	PCP005852	XM_009361807
PCP005859	4,969472865	5,459431619	5,681730355	4,700439718	PCP005859	XM_009361732
PCP005870	6,420718183	6,403778984	6,50255338	7,494495616		
PCP005875	4,906890596	5,459431619	5,727920455	6,291493053	PCP005875	XM_018645256
PCP005959	6,266786541	6,60481265	6,72342204	7,377904593		
PCP006002	4,969472865	5,115615931	5,183089461	6,129283017	PCP006002	XM_009360003
PCP006055	5,273142859	4,662775172	5,014801602	4,272769732	PCP006055	XM_009344984
PCP006069	6,169925001	6,31596467	6,662917555	7,315874125	PCP006069	XM_009379361
PCP006078	6,007419784	6,183288001	6,22881869	5,523561956		
PCP006080	5,938521046	5,614709844	5,672425342	5,403608584	PCP006080	XM_009363127
PCP006140	6,426264755	6,285402219	6,215872703	5,849749117	PCP006140	XM_009362615
PCP006206	3,058316496	3,368768349	4,95419631	6,54426899	PCP006206	XM_009363908
PCP006220	5,48123435	5,57500972	6,143026004	6,695854658	PCP006220	XM_009347604
PCP006280	5,115615931	5,142821844	5,169925001	4,297925053	PCP006280	XM_008361819
PCP006293	5,663059924	6,029673735	5,533874777	5,357552005	PCP006293	XM_009379661
PCP006306	5,523561956	5,584962501	5,196528361	4,906890596	PCP006306	XM_009364936
PCP006322	6,619559738	6,06608919	5,922911406	5,992541859	PCP006322	XM_009347655
PCP006327	6,215872703	6,584962501	6,824386003	6,902917719		
PCP006361	5,156639311	5,727920455	5,746043983	7,544346278		
PCP006364	6,169925001	6,06608919	6,169925001	5,700439718	PCP006364	XM_009379453
PCP006385	6,759289016	6,81159947	5,544423562	5,02989455		
PCP006393	5,77267747	5,073391816	5,260402093	3,840966704		
PCP006398	7,047996356	6,266786541	6,266786541	5,624393382	PCP006398	XM_018648044
PCP006414	6,853870927	6,745909573	6,554588852	6,486553493	PCP006414	XM_017331704
PCP006449	4,624685811	4,544114402	4,807354922	6,122465658	PCP006449	XM_009362257
PCP006452	7,279192684	6,781359714	5,824513297	6,285402219	PCP006452	XR_668246
PCP006535	6,108524457	5,718909554	6,087462841	6,820178962	PCP006535	XM_018645288
PCP006550	6,015024705	5,95419631	5,727920455	5,415150205		
PCP006556	4,523561956	4,874305166	5,087462841	5,736875423	PCP006556	XM_009367719
PCP006571	5,044394119	4,824258697	5,789990019	4,624685811	PCP006571	XM_009372582
PCP006591	7,033423002	6,534030467	6,594996337	6,189824559	PCP006591	XM_009372642
PCP006605	5,333781501	4,969472865	6,044394119	5,754887502	PCP006605	XM_008357515

PCP006607	6,176522247	6,574858391	6,54951516	6,894817763	PCP006607	XM_008372042
PCP006633	5,044394119	5,554588852	5,798828178	5,087462841		
PCP006655	6,209453366	6,14974712	6,357552005	7,28845085	PCP006655	XM_008366387
PCP006661	5,309976492	5,459431619	4,95419631	4	PCP006661	XM_008376221
PCP006679	6,058965822	6,321928095	6,497133304	6,832890014	PCP006679	XM_008241508
PCP006692	4,059182199	4,272769732	4,922673593	6,163297449	PCP006692	XM_009340897
PCP006696	6,513016928	6,189824559	6,486553493	7,17990909	PCP006696	XM_009341010
PCP006714	5,961854808	5,653346693	5,824513297	7,453929061	PCP006714	XM_017322560
PCP006726	6,080444483	6,80309785	6,695854658	6,122465658		
PCP006741	6,203005338	6,321928095	6,574858391	6,906890596	PCP006741	XM_018650276
PCP006748	5,357552005	5,415150205	5,392317423	4,34553831		
PCP006759	6,07317756	6,14974712	6,285402219	6,95419631		
PCP006774	5,285402219	4,922673593	4,938756261	6,497133304	PCP006774	XM_009353317
PCP006780	4,523561956	4,906890596	5,866166169	6,634157606	PCP006780	XM_009353302
PCP006791	6,718772592	5,961854808	4,874305166	4,772413555	PCP006791	XM_009353284
PCP006804	5,59484709	5,906890596	6,285402219	6,781359714	PCP006804	XM_009353252
PCP006806	6,272956308	5,815831566	5,824513297	5,115615931	PCP006806	XM_009353257
PCP006823	6,297741678	6,380764075	6,639087423	7,108524457	PCP006823	XM_017329836
PCP006832	6,297741678	5,513174885	6,247927513	5,874551087	PCP006832	XM_008345260
PCP006861	5,470211457	5,437294331	5,914803677	6,459431619		
PCP006878	5,984817174	5,564683017	5,415150205	4,890933022	PCP006878	XM_008379985
PCP006897	6,38663853	6,491853096	6,22881869	7,073284692	PCP006897	XM_009355158
PCP006914	5,235344128	5,345893086	6,420718183	5,554588852	PCP006914	XM_008373685
PCP006931	4,73714592	4,73714592	5,415150205	6,54951516	PCP006931	XM_008373663
PCP006934	6,569855608	6,420718183	6,824386003	7,156538193		
PCP006952	6,169925001	6,732404887	6,777025123	6,115407855	PCP006952	XM_008379151
PCP006965	6,594996337	6,380764075	6,327866971	7,040782866		
PCP006972	6,007419784	5,513174885	5,57500972	5,169925001	PCP006972	XM_008369271
PCP006974	6,984931073	6,339850003	6,215872703	6,662917555	PCP006974	XM_009352898
PCP006981	6,058965822	5,718909554	5,700439718	5,380937195	PCP006981	XM_009352941
PCP006990	6,890811455	6,926829678	6,898812977	6,574858391	PCP006990	XM_009363035
PCP007007	6,345715709	5,624393382	6,058965822	5,77267747		
PCP007016	6,874428132	6,38663853	6,47037417	6,235152624	PCP007016	XM_009355881
PCP007034	5,392317423	5,156639311	4,857980995	4,459431619	PCP007034	XM_009340968
PCP007047	6,029673735	5,857980995	6,044394119	6,737010678	PCP007047	XM_008377284
PCP007048	5,727920455	5,142821844	4,969472865	3,938285792	PCP007048	XM_009340246
PCP007090	6,136068312	6,345715709	6,486553493	7,011227255	PCP007090	XM_009360532
PCP007091	6,750472519	6,163297449	6,594996337	6,327866971	PCP007091	XM_009360529
PCP007096	5,634302842	5,333781501	4,857980995	4,584962501	PCP007096	XM_008361547
PCP007114	6,926829678	6,95419631	6,392317423	6,614709844	PCP007114	XM_009343168
PCP007123	5,798828178	6,235152624	6,634157606	7,366322214	PCP007123	XM_009354965
PCP007148	6,279285561	6,564835417	6,777025123	7,159871337	PCP007148	XR_001951932
PCP007151	4,662775172	5,183089461	6,07317756	7,50517639		
PCP007156	6,718772592	6,574858391	6,554588852	5,841218374	PCP007156	XM_008344754
PCP007173	4,662775172	4,247927513	5,183089461	5,992541859	PCP007173	XM_009354462
PCP007181	5,874551087	5,115615931	6,136068312	6,513016928	PCP007181	XM_009354479

PCP007201	5,380937195	5,564683017	5,403608584	4,95419631		
PCP007216	6,351734323	6,648609245	6,54951516	7,564759219		
PCP007222	6,759289016	6,579994693	6,62935662	5,564683017	PCP007222	XM_009359499
PCP007326	6,260590275	6,209453366	6,898812977	6,969587981	PCP007326	XM_009374613
PCP007354	6,794415866	6,4374608	6,634157606	6,491853096	PCP007354	XM_009348416
PCP007376	6,260590275	6,392317423	6,589913261	6,163297449	PCP007376	XM_009354931
PCP007419	4,906890596	5,672425342	6,686500527	6,700439718	PCP007419	XM_009337422
PCP007440	5,380937195	5,77267747	5,663059924	5,115615931	PCP007440	XM_009343038
PCP007456	5,48123435	5,533874777	4,841469999	4,247927513	PCP007456	XM_008340005
PCP007489	6,03716255	5,77267747	5,614709844	5,309976492	PCP007489	XM_009376585
PCP007500	6,115407855	6,609843592	6,559644763	7,206232954	PCP007500	XR_666723
PCP007501	6,129283017	6,209453366	6,539158811	7,14974712	PCP007501	XR_524153
PCP007509	5,369117459	6,297741678	5,285402219	7,662988741	PCP007509	XM_009360014
PCP007510	6,539158811	6,235152624	5,77267747	5,115615931		
PCP007537	6,176522247	5,882643049	6,414981143	6,68187088	PCP007537	XM_009354656
PCP007545	6,235152624	6,741466986	5,890689878	4,985044962	PCP007545	XM_008349395
PCP007548	5,014801602	4,564987801	5,841218374	5,554588852	PCP007548	XM_018649814
PCP007574	7,069637728	6,624539604	6,996275749	6,727920455	PCP007574	XM_018652394
PCP007579	5,403608584	5,624393382	5,333781501	4	PCP007579	XM_018643221
PCP007591	6,50255338	6,653490009	6,658211483	6,397974049		
PCP007603	6,266786541	6,686500527	6,750472519	7,315874125		
PCP007628	5,437294331	5,073391816	4,321928095	4,297925053	PCP007628	XM_018644417
PCP007634	6,958030641	6,431790083	6,321928095	5,95419631	PCP007634	XM_009369037
PCP007636	4,087462841	4,523561956	6,414981143	5,841218374	PCP007636	XM_008358141
PCP007675	7,17990909	7,209453366	6,754887502	6,222456826	PCP007675	XM_017337082
PCP007702	5	5,614709844	5,321928095	6,07317756		
PCP007720	5,634302842	5,073391816	5,807354922	5,129283017	PCP007720	XM_008380107
PCP007763	6,409390936	6,339850003	6,68187088	7,069637728	PCP007763	XM_009349923
PCP007784	6,183288001	5,672425342	6,183288001	5,736875423		
PCP007785	5,544423562	6,143026004	6,380764075	6,624539604	PCP007785	XR_664479
PCP007802	6,890811455	6,837060204	6,594996337	6,4374608	PCP007802	XM_009369485
PCP007809	5,754887502	5,890689878	6,737010678	7,011227255	PCP007809	XM_017331588
PCP007815	6,807354922	6,81159947	6,397974049	6,481072857	PCP007815	XM_008380258
PCP007825	6,022367813	5,169925001	5,533874777	5,653346693	PCP007825	XM_008369714
PCP007827	5,57500972	5,653346693	5,807354922	6,700439718		
PCP007828	6,321928095	6,497133304	6,420718183	6,14974712	PCP007828	XM_008345759
PCP007836	5,209453366	4,624685811	4,502712486	4,196134881	PCP007836	XM_008376986
PCP007841	5,992541859	5,857980995	6,101608059	5,235344128		
PCP007867	5,672425342	5,523561956	5,736875423	4,922673593	PCP007867	XM_018644378
PCP007870	3,624100895	5,502394256	4,906890596	4,321928095		
PCP007906	5,470211457	6,136068312	5,890689878	5,564683017	PCP007906	XM_009351141
PCP007912	6,942514505	6,169925001	6,357552005	6,62935662	PCP007912	XM_018643024
PCP007917	6,84962403	6,695854658	6,420718183	6,321928095	PCP007917	XM_009340052
PCP007997	4,662775172	5,222263604	5,415150205	5,798828178	PCP007997	XM_009336035
PCP008007	5,653346693	5,969703088	6,094447222	5,513174885	PCP008007	XM_018650777
PCP008012	6,50255338	6,448405435	6,691115365	7,677155841		

PCP008056	5,906890596	5,95419631	5,681730355	5,502394256	PCP008056	XM_008379949
PCP008145	6,95419631	6,403778984	6,790120385	6,321928095	PCP008145	XM_009374468
PCP008147	7,297649983	6,772545519	6,709704193	5,922911406	PCP008147	XM_018650563
PCP008155	4,321928095	6,115407855	6,695854658	5,95419631	PCP008155	XM_009372357
PCP008156	3,27351589	5,357552005	5,459431619	4,73714592	PCP008156	XM_009372356
PCP008161	5,663059924	6,136068312	7,592457037	7,273049587	PCP008161	XM_009372352
PCP008243	4,392317423	4,604664415	4,523561956	5,663059924	PCP008243	XM_008356700
PCP008295	3,700439718	4,754887502	5,115615931	5,906890596	PCP008295	XM_008391486
PCP008297	5,60496087	5,672425342	6,215872703	5,183089461	PCP008297	XM_008352714
PCP008303	5	5,169925001	5,044394119	4,169925001		
PCP008307	6,599912842	6,215872703	5,57500972	5,426264755	PCP008307	XM_008391458
PCP008323	5,60496087	5,60496087	5,95419631	6,634157606		
PCP008333	6,22881869	6,31596467	6,087462841	5,969703088	PCP008333	XM_017336994
PCP008352	4,222650022	5,073391816	4,969472865	5,672425342	PCP008352	XM_017336701
PCP008370	5,297558281	5,448570626	5,624393382	6,700439718	PCP008370	XM_009356592
PCP008395	6,426264755	6,614709844	6,86628983	7,087462841		
PCP008428	5,459431619	5,789990019	6,584962501	6,634157606	PCP008428	XM_009335817
PCP008432	6,431790083	6,351734323	6,022367813	5,59484709	PCP008432	XM_018642512
PCP008440	3,700439718	5,115615931	6,497133304	7,969645536		
PCP008507	6,768184325	6,189824559	6,369291982	5,403608584	PCP008507	XM_008393481
PCP008511	5,415150205	5,663059924	5,922911406	6,594996337	PCP008511	XM_009348572
PCP008527	4,857980995	5,77267747	5,841218374	6,691115365	PCP008527	XM_008352440
PCP008528	4,564987801	3,736604875	4,807354922	5,209453366	PCP008528	XM_018642343
PCP008538	7,189824559	6,481072857	5,426264755	5,564683017	PCP008538	XM_018642330
PCP008553	6,115407855	6,163297449	6,798698597	5,938521046	PCP008553	XM_009339388
PCP008559	4,115199749	4,662775172	3,700439718	6,667750232	PCP008559	XM_008358934
PCP008587	4,392317423	4,059182199	4,718635616	5,691254979	PCP008587	XM_009360986
PCP008602	5,309976492	5,57500972	4,890933022	5,890689878		
PCP008634	4,087462841	4,790250739	5,754887502	5,392317423	PCP008634	XM_008221236
PCP008641	5,491853096	5,209453366	5,938521046	6,72342204		
PCP008646	6,442943496	6,392317423	6,584962501	7,183188734		
PCP008712	6,209453366	6,022367813	6,898812977	7,122362117	PCP008712	XM_009363626
PCP008716	6,894817763	6,824386003	6,741466986	6,539158811	PCP008716	XR_001953539
PCP008720	6,38663853	6,189824559	5,663059924	6,414981143	PCP008720	XM_018648640
PCP008721	4,824258697	5,142821844	6,303780748	5,789990019	PCP008721	XM_018648641
PCP008722	6,539158811	5,247927513	6,569855608	5,491853096	PCP008722	XM_009363638
PCP008750	6,777025123	6,837060204	6,785681319	6,50255338		
PCP008767	6,454011343	6,569855608	6,60481265	7,321928095	PCP008767	XM_009357637
PCP008776	6,589913261	6,215872703	6,129283017	6,03716255	PCP008776	XM_009359083
PCP008831	4,437627248	5,014801602	6,209453366	8,993419626	PCP008831	XM_008395519
PCP008838	6,015024705	5,634302842	6,156437068	5,491853096	PCP008838	XM_018648395
PCP008864	5,222263604	6,22881869	5,672425342	5,614709844	PCP008864	XM_009355636
PCP008897	6,196331634	5,95419631	5,700439718	5,754887502	PCP008897	XM_009347544
PCP008900	6,22881869	6,518377768	6,820178962	6,946379968	PCP008900	XM_009347545
PCP008912	6,97349648	6,942514505	6,285402219	6,260590275	PCP008912	XM_009345310
PCP008915	6,894817763	6,857980995	6,828707735	6,247927513	PCP008915	XM_009336198

PCP008916	5,044394119	5,392317423	4,969472865	4,459431619		
PCP008954	6,700439718	6,156437068	6,686500527	6,375039431	PCP008954	XM_009346690
PCP008956	6,279285561	5,544423562	4,985044962	4,584962501	PCP008956	XM_009344506
PCP008968	5,857980995	5,681730355	7,451211112	6,491853096	PCP008968	XM_008370092
PCP008976	6,442943496	6,922792504	6,857980995	6,672425342		
PCP009006	6,309794644	6,420718183	6,333960351	7,062531903	PCP009006	XM_017323213
PCP009019	5,815831566	6,176522247	6,309794644	7,080337882		
PCP009027	7,372168569	7,08395793	6,820178962	6,058965822		
PCP009038	6,14974712	6,291493053	6,619559738	7,073284692	PCP009038	XM_008339162
PCP009039	6,122465658	6,215872703	6,841344192	7,336908182	PCP009039	XM_009375745
PCP009048	5,448570626	5,196528361	5,014801602	4,115199749	PCP009048	XM_018651325
PCP009081	5,946496941	5,285402219	4,662775172	5,044394119	PCP009081	XM_008349113
PCP009092	6,094447222	6,448405435	6,409390936	7,357552005	PCP009092	XM_009348700
PCP009123	5,448570626	4,807354922	4,64385619	4,584962501	PCP009123	XM_009373235
PCP009124	6,24164954	6,087462841	6,327866971	6,81159947	PCP009124	XM_009349296
PCP009133	6,015024705	4,34553831	5,115615931	5,357552005	PCP009133	XM_009343558
PCP009167	7,055282436	7,254272787	6,686500527	6,203005338		
PCP009177	4,906890596	4,841469999	5,247927513	5,938521046		
PCP009246	6,044394119	5,866166169	5,922911406	6,832890014	PCP009246	NM_001302309
PCP009285	5,014801602	5,044394119	4,624685811	6		
PCP009299	5,584962501	5,345893086	4,922673593	4,480911346	PCP009299	XM_018649124
PCP009373	6,518377768	6,189824559	6,327866971	5,763677142	PCP009373	XM_009373975
PCP009409	6,03716255	5,624393382	6,475733431	6,14974712	PCP009409	XM_009365665
PCP009411	6,624539604	6,297741678	6,599912842	7,062531903	PCP009411	XM_018649067
PCP009428	4,34553831	4,662775172	5,196528361	5,564683017	PCP009428	XM_018643939
PCP009429	5,614709844	5,874551087	6,345715709	5,554588852	PCP009429	XM_018651285
PCP009441	5,554588852	4,754887502	4,523561956	4,169925001	PCP009441	XM_009366778
PCP009482	6,235152624	6,143026004	6,087462841	5,459431619	PCP009482	XM_009365243
PCP009501	4,392317423	5,260402093	5,448570626	5,906890596	PCP009501	XM_008388291
PCP009538	6,559644763	6,357552005	6,574858391	6,215872703	PCP009538	XM_009354795
PCP009578	5,380937195	5,584962501	6,235152624	6,459431619		
PCP009647	5,992541859	5,77267747	6,333960351	6,768184325	PCP009647	NM_001302282
PCP009653	6,14974712	6,235152624	6,215872703	5,857980995	PCP009653	XM_008344777
PCP009717	6,203005338	6,176522247	5,882643049	4,392317423		
PCP009725	6,122465658	5,523561956	5,718909554	5,491853096	PCP009725	XM_008341439
PCP009750	5,718909554	5,914803677	5,448570626	5,209453366	PCP009750	XM_009358968
PCP009759	7,156538193	6,272956308	6,4374608	5,824513297	PCP009759	XM_009359012
PCP009782	5,922911406	5,624393382	5,403608584	5,235344128	PCP009782	XM_009359335
PCP009824	4,604664415	5,073391816	5,700439718	5,073391816	PCP009824	XM_009346175
PCP009828	5,77267747	5,781359714	6	5	PCP009828	NM_001328761
PCP009857	4,604664415	5,333781501	5,369117459	5,691254979	PCP009857	XM_018645260
PCP009889	6,790120385	6,914923239	6,291493053	6,254178286	PCP009889	XM_009357764
PCP009902	6,564835417	6,47037417	6,922792504	7,318949464	PCP009902	XM_018647387
PCP009913	6,309794644	6,189824559	6,266786541	5,841218374	PCP009913	XM_008376653
PCP009920	5,584962501	5,663059924	5,59484709	6,589913261		
PCP009944	4,272769732	4,790250739	3,874796966	6,554588852	PCP009944	XM_009372261

PCP009950	6,700439718	6,584962501	6,667750232	6,094447222		
PCP009964	6,754887502	6,80309785	6,686500527	6,303780748	PCP009964	XM_008377860
PCP010003	6,420718183	6,528727582	6,54426899	5,807354922	PCP010003	XM_009364975
PCP010025	6,486553493	5,882643049	5,523561956	4,059182199	PCP010025	XM_009365004
PCP010037	6,594996337	6,115407855	6,481072857	6,291493053	PCP010037	XM_018648935
PCP010049	6,47037417	6,291493053	5,260402093	3,772941338	PCP010049	XM_009365599
PCP010065	3,584962501	4,807354922	5,718909554	5,169925001	PCP010065	XM_009364504
PCP010098	5,824513297	4,718635616	4,682011391	4,459431619	PCP010098	XM_009342482
PCP010113	5,841218374	6,534030467	6,409390936	7,018700931		
PCP010144	5,681730355	6,015024705	6,007419784	5,60496087	PCP010144	XM_009377344
PCP010190	5,866166169	5,169925001	5,380937195	4,874305166	PCP010190	XM_009374096
PCP010192	5,672425342	6,363346321	5,961854808	5,914803677	PCP010192	XM_009374097
PCP010222	5,789990019	5,938521046	5	4,874305166	PCP010222	XM_009373515
PCP010254	6,163297449	6,667750232	6,50779464	5,984817174	PCP010254	XM_009376142
PCP010280	5,169925001	5,634302842	6,454011343	5,415150205		
PCP010326	6,491853096	6,163297449	6,569855608	6,06608919	PCP010326	XM_009376399
PCP010332	5,273142859	5,709566354	5,60496087	6,297741678	PCP010332	XM_009351819
PCP010373	6,481072857	5,403608584	5,807354922	5,196528361	PCP010373	XM_009380920
PCP010380	6,497133304	7,08395793	6,50255338	6,709704193	PCP010380	XM_008359420
PCP010392	7,327956767	6,832890014	7,169925001	5,984817174		
PCP010404	6,84962403	7,173227395	6,564835417	6,662917555	PCP010404	XM_008369112
PCP010444	4,682011391	5	5,247927513	5,807354922	PCP010444	XM_008378918
PCP010448	6,183288001	6,579994693	6,523561956	5,969703088	PCP010448	XM_008378922
PCP010451	6,807354922	6,156437068	6,183288001	6,648609245	PCP010451	XM_008379005
PCP010474	5,129283017	5,309976492	5,297558281	6,345715709		
PCP010484	6,695854658	6,599912842	6,279285561	6,101608059		
PCP010487	5,832890014	5,681730355	5,727920455	5,309976492		
PCP010543	6,528727582	6,534030467	6,420718183	6,235152624	PCP010543	XM_018644023
PCP010559	4,143230135	4,321928095	5,260402093	5,345893086		
PCP010610	4,662775172	4,480911346	4,95419631	5,807354922	PCP010610	XM_009364318
PCP010613	4,790250739	4,34553831	5,260402093	4,169925001	PCP010613	XM_009364356
PCP010635	4,029452886	5,77267747	5,523561956	6,906890596	PCP010635	XM_009352371
PCP010639	6,414981143	6,559644763	6,86628983	7,183188734		
PCP010670	4,029452886	5,058749412	5,459431619	6,62935662	PCP010670	XM_018644144
PCP010675	5,564683017	5,073391816	4,682011391	4,700439718	PCP010675	XM_018649215
PCP010701	5,345893086	5,789990019	6,403778984	7,055282436	PCP010701	XM_009341224
PCP010715	6,333960351	6,38663853	6,862079387	7,189824559	PCP010715	XM_008361427
PCP010733	6,886672074	6,559644763	6,584962501	6,363346321	PCP010733	XR_668579
PCP010754	4,143230135	4,34553831	4,604664415	6,345715709	PCP010754	XM_009343990
PCP010767	6,624539604	6,454011343	6,414981143	6,291493053	PCP010767	XM_009347851
PCP010801	6,007419784	5,247927513	5,709566354	4,874305166	PCP010801	XM_008345517
PCP010803	6,961970533	6,934634441	6,64385619	5,321928095	PCP010803	XM_009374944
PCP010804	7,033423002	6,513016928	6,705010253	6,24164954	PCP010804	NM_001293900
PCP010810	5,247927513	4,604664415	4,718635616	4,502712486	PCP010810	XM_009350215
PCP010812	6,420718183	5,874551087	5,946496941	6,007419784		
PCP010888	5,014801602	5,554588852	5,961854808	6,094447222	PCP010888	XM_009362291

PCP010943	7,282347131	6,84962403	6,369291982	6,022367813		
PCP010951	6,235152624	6,260590275	6,497133304	7,076815597	PCP010951	XM_008377808
PCP010957	5,841218374	5,169925001	4,969472865	5,115615931	PCP010957	XM_009352717
PCP010958	5,403608584	5,058749412	5,297558281	4,459431619	PCP010958	XM_021976056
PCP010960	6,094447222	6,745909573	7,297649983	6,914923239	PCP010960	XM_009365137
PCP010966	5,369117459	4,754887502	4,662775172	4,459431619	PCP010966	XM_009366760
PCP011011	5,297558281	4,969472865	4,087462841	4,321928095	PCP011011	XR_001791946
PCP011013	5,142821844	4,807354922	4,480911346	4,321928095	PCP011013	XM_008367659
PCP011024	6,624539604	5,969703088	6,176522247	6,156437068		
PCP011034	7,058857621	6,768184325	6,737010678	6,81159947	PCP011034	XM_008390591
PCP011036	6,309794644	5,746043983	5,898933872	5,448570626		
PCP011059	6,574858391	5,369117459	5,087462841	3,321928095		
PCP011069	6,658211483	6,658211483	6,589913261	6,380764075	PCP011069	XM_018651260
PCP011073	5	5,470211457	5,523561956	5,984817174	PCP011073	XM_009345365
PCP011082	4,584962501	4,824258697	5,297558281	5,946496941	PCP011082	XM_008357158
PCP011120	6,464831606	6,235152624	6,832890014	5,882643049	PCP011120	XM_008387935
PCP011149	5,48123435	6,285402219	6,309794644	5,781359714	PCP011149	XM_008377859
PCP011170	2,321928095	5,64385619	5,470211457	4,624685811	PCP011170	XM_008393210
PCP011174	6,95419631	6,196331634	6,564835417	6,403778984	PCP011174	XM_009373893
PCP011214	5,415150205	5,554588852	5,984817174	6,584962501	PCP011214	XM_009347101
PCP011255	6,579994693	6,658211483	6,677085351	6,087462841	PCP011255	XM_009342056
PCP011259	6,309794644	6,448405435	7,029784146	7,564759219	PCP011259	XR_668727
PCP011270	5,969703088	5,824513297	5,914803677	5,426264755	PCP011270	XM_009345007
PCP011275	5,977279923	5,584962501	5,345893086	5,156639311	PCP011275	XM_009339202
PCP011290	4,841469999	4,544114402	6,594996337	5,014801602	PCP011290	XM_008379204
PCP011334	5,544423562	5,961854808	5,866166169	7,569855608		
PCP011371	6,38663853	6,357552005	6,832890014	7,315874125	PCP011371	XM_009370540
PCP011399	5,321928095	5,426264755	6,176522247	6,196331634	PCP011399	XR_666213
PCP011400	6,454011343	6,321928095	6,431790083	6,080444483	PCP011400	XM_009349994
PCP011402	5,857980995	5,866166169	5,857980995	6,564835417	PCP011402	XM_009349981
PCP011451	4,890933022	4,874305166	6,448405435	7,263691734	PCP011451	XM_009380523
PCP011471	6,942514505	6,853870927	6,777025123	6,614709844	PCP011471	XM_018650179
PCP011490	5,209453366	4,841469999	6,136068312	5,922911406	PCP011490	XM_009355182
PCP011523	6,732404887	7,003714662	6,60481265	6,297741678	PCP011523	XM_008389080
PCP011550	6,260590275	7,029784146	6,777025123	7,098032083		
PCP011561	5,746043983	6,279285561	6,07317756	5,866166169	PCP011561	XM_008349879
PCP011584	5,369117459	6,06608919	5,922911406	5,073391816		
PCP011597	5,807354922	5,448570626	4,700439718	4,564987801	PCP011597	XM_009344191
PCP011626	5,849749117	6,115407855	6,279285561	6,759289016		
PCP011627	7,069637728	6,31596467	6,700439718	6,309794644	PCP011627	XM_008363573
PCP011654	6	6,051589621	6,523561956	6,926829678	PCP011654	XM_009346443
PCP011671	5,898933872	6,375039431	6,183288001	6,754887502	PCP011671	XM_008345403
PCP011679	5,297558281	4,824258697	6,029673735	6,333960351	PCP011679	XM_009346868
PCP011689	6	5,736875423	5,533874777	5,273142859	PCP011689	XM_018647179
PCP011691	6,169925001	5,95419631	6,115407855	5,624393382	PCP011691	XM_009356813
PCP011698	6,95419631	6,375039431	6,101608059	5,727920455	PCP011698	XM_009356129

PCP011705	6	4,087462841	3,221877081	7,811663685	PCP011705	XM_009354814
PCP011721	6,448405435	6,741466986	6,794415866	7,125878364		
PCP011728	5,977279923	6,84962403	6,285402219	6,007419784	PCP011728	XM_009361816
PCP011737	5,129283017	6,015024705	4,922673593	4,824258697		
PCP011738	6,763809907	6,874428132	6,777025123	6,589913261	PCP011738	XM_009361803
PCP011779	5,169925001	5,584962501	6,156437068	6,285402219	PCP011779	XR_001951337
PCP011788	7,318949464	6,662917555	6,857980995	6,594996337	PCP011788	XM_009366727
PCP011808	6,156437068	6,426264755	6,38663853	5,882643049	PCP011808	XM_008354615
PCP011809	5,415150205	4,700439718	4,297925053	4,874305166	PCP011809	XM_008341046
PCP011859	6,363346321	6,080444483	6,86628983	7,163196797	PCP011859	XM_008385585
PCP011870	6,143026004	6,523561956	6,497133304	5,832890014	PCP011870	XM_008360472
PCP011871	6,634157606	6,768184325	6,47037417	6,369291982		
PCP011885	6,03716255	6,695854658	6,38663853	6,058965822		
PCP011894	3,969933275	5,946496941	6,189824559	8,850718177	PCP011894	XM_009350624
PCP011896	5,285402219	4,874305166	5,044394119	5,930737338		
PCP011900	7,648681141	6,426264755	6,714245518	6,136068312	PCP011900	XM_008393501
PCP011929	6,54426899	6,222456826	6,06608919	5,798828178	PCP011929	XM_009347931
PCP011941	6,397974049	5,491853096	5,513174885	5,247927513		
PCP011963	5,426264755	4,824258697	4,841469999	4,34553831	PCP011963	XM_009348353
PCP011977	6,31596467	6,50255338	6,574858391	7,321928095		
PCP011986	5,285402219	4,985044962	6,266786541	7,026025399	PCP011986	XM_008370019
PCP011991	5,789990019	5,209453366	5,273142859	4,718635616		
PCP012022	6,898812977	7,166615031	6,785681319	6,594996337	PCP012022	XM_009336151
PCP012044	6,303780748	5,922911406	5,044394119	5,59484709	PCP012044	XM_009357968
PCP012052	5,718909554	5,614709844	5,849749117	7,377904593	PCP012052	XM_008393281
PCP012056	5,914803677	5,02989455	5,183089461	5,058749412	PCP012056	XM_009369342
PCP012058	6,750472519	6,397974049	7	7,17990909	PCP012058	XM_009369374
PCP012078	5,584962501	5,841218374	6,363346321	6,589913261	PCP012078	XM_009373892
PCP012101	6,942514505	6,528727582	6,634157606	6,31596467	PCP012101	XM_009350586
PCP012104	6,934634441	6,143026004	6,015024705	5,59484709	PCP012104	XM_009374302
PCP012117	6,781359714	6,431790083	6,691115365	6,448405435	PCP012117	XM_008353378
PCP012151	6,579994693	6,874428132	6,431790083	6,279285561	PCP012151	XM_009371305
PCP012193	5,297558281	5,437294331	5,523561956	6,176522247	PCP012193	XR_526733
PCP012210	4,64385619	4,437627248	4,459431619	5,882643049		
PCP012226	6,115407855	6,176522247	6,491853096	7,142923928	PCP012226	XM_009343802
PCP012254	6,569855608	5,653346693	4,938756261	3,874796966	PCP012254	XM_008362276
PCP012285	5,77267747	5,101397952	5,700439718	6,667750232	PCP012285	XM_009342989
PCP012302	5,763677142	5,513174885	5,832890014	5,196528361		
PCP012316	5,183089461	5,459431619	5,938521046	6,772545519	PCP012316	XM_018650378
PCP012355	5,95419631	5,653346693	5,624393382	4,841469999	PCP012355	XM_009367530
PCP012387	6,022367813	5,169925001	5,247927513	5,321928095		
PCP012388	5,64385619	4,857980995	4,321928095	3,502075956		
PCP012443	5,614709844	5,984817174	6,24164954	7,087462841	PCP012443	XM_009340428
PCP012479	5,235344128	5,57500972	6,051589621	6,686500527	PCP012479	XM_009378516
PCP012552	7,17990909	7,266786541	6,62935662	6,196331634	PCP012552	XM_009370290
PCP012628	6,705010253	5,392317423	3,584962501	3,058316496	PCP012628	XM_009369277

PCP012705	5,309976492	5,992541859	6,4374608	6,922792504	PCP012705	XR_001954740
PCP012771	5,614709844	5,058749412	5,115615931	4,459431619	PCP012771	XM_009354789
PCP012775	5,95419631	5,357552005	5,736875423	5,380937195	PCP012775	XM_008365076
PCP012804	6,459431619	6,122465658	6,108524457	6,03716255		
PCP012816	5,247927513	5,502394256	4,969472865	4,369466484		
PCP012817	6	5,709566354	6,534030467	7,026025399	PCP012817	XM_009373654
PCP012855	6,156437068	6	6,015024705	6,798698597	PCP012855	XM_009364145
PCP012862	5,614709844	6,392317423	5,437294331	5,357552005	PCP012862	XM_008377842
PCP012978	6,260590275	5,196528361	5,222263604	5,285402219	PCP012978	XM_018651580
PCP012980	6,015024705	6,486553493	5,523561956	4,059182199	PCP012980	XM_009376877
PCP013010	5,247927513	5,058749412	5,222263604	6,087462841	PCP013010	XM_017329476
PCP013014	6,333960351	6,247927513	6,247927513	5,857980995	PCP013014	XM_009365202
PCP013025	6,54426899	6,906890596	6,539158811	6,345715709	PCP013025	XM_009337979
PCP013032	5,824513297	6,303780748	5,700439718	4,969472865	PCP013032	XM_020562191
PCP013035	4,64385619	5,235344128	6,215872703	5,014801602	PCP013035	XM_007223879
PCP013053	6,051589621	5,196528361	4,272769732	4,196134881	PCP013053	XM_008374883
PCP013095	5,544423562	5,403608584	5,564683017	6,285402219	PCP013095	XM_008363539
PCP013100	4,73714592	5,058749412	4,938756261	7,327956767	PCP013100	XM_008363532
PCP013104	6,898812977	6,894817763	6,54426899	6,129283017	PCP013104	XM_017323790
PCP013113	6,486553493	5,624393382	5,841218374	5,882643049	PCP013113	XM_018646652
PCP013154	5,815831566	5,309976492	4,922673593	4,247927513	PCP013154	XM_009372907
PCP013202	5,513174885	5,857980995	6,129283017	6,589913261	PCP013202	XM_009370903
PCP013205	6,718772592	6,745909573	6,215872703	6,03716255	PCP013205	XM_009370899
PCP013210	6,426264755	6,24164954	5,624393382	6,369291982	PCP013210	XM_009370889
PCP013243	5,380937195	5,448570626	5,584962501	6,497133304	PCP013243	XM_009339993
PCP013247	5,297558281	4,718635616	5,101397952	4,321928095	PCP013247	XM_009339990
PCP013255	7,153095972	6,686500527	6,448405435	6,351734323	PCP013255	XM_008340167
PCP013285	5,554588852	6,209453366	6,169925001	6,426264755	PCP013285	XM_009366305
PCP013306	7,076815597	6,327866971	6,569855608	5,984817174	PCP013306	XM_009381287
PCP013310	6,339850003	6,321928095	6,619559738	7,462134139	PCP013310	XM_009381292
PCP013326	6,564835417	6,222456826	6,431790083	7,21916852	PCP013326	XM_009339860
PCP013330	5,882643049	5,653346693	6,339850003	6,579994693		
PCP013344	6,203005338	5,273142859	5,196528361	4,64385619		
PCP013350	6,759289016	6,695854658	6,68187088	7,263691734	PCP013350	XM_009376233
PCP013360	7,251056285	6,841344192	6,72342204	6,203005338	PCP013360	XM_009376250
PCP013414	5,634302842	5,914803677	5,672425342	6,448405435	PCP013414	XM_009370808
PCP013429	6,07317756	6,247927513	6,122465658	5,857980995	PCP013429	XM_009342559
PCP013453	3,169925001	3,624100895	5,614709844	5,681730355	PCP013453	XM_017333364
PCP013459	6,807354922	6,54951516	6,564835417	6,426264755	PCP013459	XM_009354885
PCP013462	5,156639311	4,772413555	4,682011391	4,222650022	PCP013462	XM_009354878
PCP013468	6,745909573	3,969933275	5,345893086	5	PCP013468	XM_009365042
PCP013469	5	4,969472865	5,906890596	5,984817174	PCP013469	XM_009365093
PCP013487	6,176522247	6,266786541	6,051589621	5,906890596	PCP013487	XM_009365346
PCP013539	5,922911406	4,874305166	3,906890596	4,115199749		
PCP013576	6,513016928	5,624393382	5,815831566	5,101397952	PCP013576	XM_009352893
PCP013597	5,209453366	5,849749117	6,215872703	6,380764075	PCP013597	XM_009345365

PCP013600	5,736875423	5,345893086	6,369291982	7,105070402	PCP013600	XM_009340444
PCP013631	5,142821844	5,832890014	5,59484709	6,07317756	PCP013631	XM_009355354
PCP013645	5,02989455	5,058749412	5,073391816	6	PCP013645	XM_009356354
PCP013657	5,129283017	5,297558281	5,922911406	6,24164954	PCP013657	XM_009356234
PCP013663	6,639087423	6,291493053	6,481072857	6,961970533	PCP013663	XM_008343352
PCP013664	5,849749117	4,73714592	4,564987801	4,564987801	PCP013664	XM_008355199
PCP013691	4,059182199	4,718635616	6,209453366	5,058749412	PCP013691	XM_009378064
PCP013730	5,781359714	5,763677142	6,189824559	7,018700931	PCP013730	XM_009381127
PCP013765	6,894817763	6,554588852	6,709704193	6,363346321	PCP013765	XM_009366538
PCP013773	6,176522247	6,215872703	6,279285561	6,938638658	PCP013773	XM_009361271
PCP013774	5,321928095	5,653346693	5,746043983	6,4374608	PCP013774	XM_018648167
PCP013775	5,309976492	6,108524457	6,058965822	6,24164954	PCP013775	XM_009361269
PCP013888	5,554588852	6,051589621	6,327866971	6,62935662		
PCP013897	5,614709844	5,345893086	4,985044962	4,604664415	PCP013897	XM_009339530
PCP013903	6,297741678	5,554588852	6,129283017	5,681730355	PCP013903	XM_009369071
PCP013911	5,459431619	5,044394119	5,209453366	6,777025123		
PCP013958	4,824258697	6,523561956	6,333960351	8,338379842	PCP013958	XM_018649522
PCP013975	5,554588852	5,709566354	4,922673593	4,64385619		
PCP013976	5,513174885	6,087462841	6,481072857	6,594996337		
PCP014012	5,874551087	6,4374608	6,930737338	7,958088658	PCP014012	XM_008242056
PCP014016	7,351645995	5,345893086	6,07317756	6,189824559	PCP014016	XR_001951611
PCP014021	6,309794644	6,363346321	6,203005338	6,022367813	PCP014021	XM_009339045
PCP014034	5,60496087	4,64385619	4,502712486	3,807354922	PCP014034	XM_009377126
PCP014040	6,420718183	6,163297449	6,392317423	7,448488033	PCP014040	XM_009346188
PCP014043	6,272956308	5,60496087	5,437294331	5,736875423	PCP014043	XM_009373655
PCP014048	4,459431619	4,73714592	5,533874777	6,431790083	PCP014048	XM_009338152
PCP014060	4,143230135	4,807354922	5,700439718	4,841469999		
PCP014153	3,116031993	3	4,64385619	9,326429487	PCP014153	XM_009371990
PCP014204	5,624393382	5,345893086	4,272769732	3,584962501	PCP014204	XM_009369473
PCP014219	3,584962501	4,922673593	5,77267747	4,247927513		
PCP014232	6,84962403	6,309794644	5,672425342	6,058965822	PCP014232	XM_009363838
PCP014233	6,38663853	6,297741678	6,828707735	7,115511897		
PCP014261	4,73714592	4,087462841	5,403608584	4,718635616	PCP014261	XM_009348584
PCP014267	6,4374608	6,420718183	6,475733431	7,247927513		
PCP014268	5,169925001	5,309976492	3,840966704	4,321928095		
PCP014294	6,115407855	6,619559738	6,189824559	6,874428132	PCP014294	XM_008353447
PCP014302	6,639087423	6,934634441	6,101608059	6,087462841		
PCP014304	5,209453366	5,882643049	6,737010678	7,018700931	PCP014304	XM_008350353
PCP014317	4,414812061	5,101397952	4,969472865	5,564683017	PCP014317	XR_529968
PCP014319	5,357552005	5,115615931	5,044394119	4,369466484	PCP014319	XM_009359146
PCP014331	6,196331634	6,426264755	5,922911406	5,922911406	PCP014331	XM_009343804
PCP014332	6,339850003	6,639087423	6,832890014	7,462134139		
PCP014350	6,709704193	6,926829678	6,691115365	4,938756261		
PCP014468	3,807354922	4	5,849749117	4,969472865	PCP014468	XM_008370092
PCP014561	6,759289016	6,686500527	6,700439718	5,77267747	PCP014561	XM_009376002
PCP014577	6,54426899	5,914803677	5,961854808	5,380937195	PCP014577	XM_009376036

PCP014600	6,080444483	6,129283017	5,321928095	3,969933275		
PCP014647	4,087462841	4,841469999	5,448570626	6,215872703	PCP014647	XM_008366360
PCP014650	5,058749412	5	6,122465658	4,95419631	PCP014650	XM_009352529
PCP014717	5,087462841	4,938756261	5,59484709	4,700439718		
PCP014736	6,619559738	6,695854658	6,574858391	6,044394119	PCP014736	XM_009366283
PCP014773	2,114367025	3,321928095	4,029452886	8,493174961	PCP014773	XM_009355003
PCP014780	6,015024705	5,624393382	6,015024705	5,273142859	PCP014780	XM_018646794
PCP014781	5,426264755	6,203005338	6,718772592	7,655852677	PCP014781	XM_009354993
PCP014784	5,129283017	6,080444483	6,961970533	8,042589623		
PCP014808	4,604664415	5,977279923	6,807354922	5,691254979	PCP014808	XM_009355728
PCP014809	6,403778984	6,609843592	5,815831566	5,196528361	PCP014809	XM_009342013
PCP014862	5,470211457	7,269874722	7,431873638	6,677085351	PCP014862	XM_008374321
PCP014863	4,196134881	4,143230135	4,906890596	5,333781501	PCP014863	XM_017332090
PCP014895	6,672425342	6,634157606	6,14974712	4,969472865		
PCP014918	7,101503009	6,785681319	7,051698368	6,528727582	PCP014918	XM_009380731
PCP014919	6,653490009	5,914803677	6,47037417	6,044394119	PCP014919	XM_009380731
PCP014983	5,333781501	5,807354922	5,754887502	6,459431619	PCP014983	XM_009377701
PCP014985	5,95419631	5,969703088	6,539158811	6,934634441		
PCP014994	5,789990019	6,285402219	6,534030467	6,942514505		
PCP015060	5,403608584	5,554588852	5,798828178	6,363346321	PCP015060	XM_009345279
PCP015070	2,22342255	4,321928095	3,969933275	7,50779464	PCP015070	XM_018649595
PCP015083	4,502712486	5,513174885	6,22881869	4,437627248	PCP015083	XM_018652126
PCP015084	3,840966704	4,969472865	5,60496087	4,392317423	PCP015084	XM_018652125
PCP015100	5,297558281	5,333781501	5,841218374	4,502712486	PCP015100	XM_009377278
PCP015101	6,886672074	7,026025399	6,672425342	6,051589621	PCP015101	XM_009377281
PCP015115	3,624100895	4,437627248	6	7,380850638		
PCP015126	6,54951516	6,790120385	6,143026004	6,403778984	PCP015126	XR_001952614
PCP015149	6,189824559	5,906890596	6,044394119	7,336908182	PCP015149	XM_009379485
PCP015163	5,922911406	5,014801602	5,357552005	4,662775172	PCP015163	XM_009374707
PCP015167	5,156639311	5,403608584	5,672425342	3,840966704	PCP015167	XM_009347113
PCP015191	6,68187088	6,454011343	6,890811455	7,212666605	PCP015191	XM_008377515
PCP015216	5,736875423	5,866166169	5,297558281	5,196528361	PCP015216	XM_009344624
PCP015227	5,437294331	5,513174885	5,624393382	6,431790083		
PCP015229	6,222456826	5,798828178	5,866166169	5,691254979	PCP015229	XM_009361942
PCP015254	6,977279923	7,044394119	6,667750232	5,866166169	PCP015254	XM_017327492
PCP015263	5,906890596	5,77267747	5,087462841	4,544114402	PCP015263	XM_009343054
PCP015268	6,554588852	6,163297449	6,420718183	5,922911406	PCP015268	XM_018649446
PCP015273	6,87036472	6,648609245	6,321928095	6,044394119	PCP015273	XM_017334122
PCP015289	6,24164954	6,058965822	5,321928095	5,357552005	PCP015289	XM_009353217
PCP015294	6,03716255	5,736875423	5,209453366	5,459431619	PCP015294	XM_008375517
PCP015301	6	6,108524457	7,429030064	7,026025399	PCP015301	XM_009371303
PCP015311	6,554588852	6,022367813	6,420718183	5,992541859	PCP015311	NM_001294001
PCP015351	5,513174885	5,554588852	5,807354922	6,454011343	PCP015351	XM_017329572
PCP015353	6,732404887	6,737010678	6,491853096	6,47037417	PCP015353	XM_008354630
PCP015360	4,143230135	4,502712486	4,874305166	5,321928095	PCP015360	XM_009344812
PCP015385	6,619559738	6,169925001	6,183288001	6,029673735	PCP015385	XM_009344166

PCP015402	6,136068312	6,677085351	6,695854658	7,156538193	PCP015402	XM_009366999
PCP015448	5,64385619	6,215872703	6,203005338	5,222263604		
PCP015463	6,815959618	6,820178962	6,594996337	6,584962501	PCP015463	XM_017336087
PCP015468	5,297558281	5,59484709	5,746043983	4,824258697		
PCP015469	6,015024705	5,073391816	5,584962501	5,763677142	PCP015469	XM_008368568
PCP015481	6,68187088	6,47037417	6,54951516	7,166615031	PCP015481	XM_009357231
PCP015486	5,832890014	5,415150205	5,681730355	4,969472865		
PCP015487	6,215872703	5,491853096	5,718909554	4,906890596		
PCP015489	6,72342204	6,554588852	6,163297449	5,709566354	PCP015489	XM_009339084
PCP015514	6,369291982	6,481072857	6,672425342	6,029673735	PCP015514	XM_018644395
PCP015536	5,183089461	5,663059924	5,415150205	6,409390936	PCP015536	XM_009344424
PCP015552	6,254178286	6,853870927	6,47037417	7,173227395	PCP015552	XM_008363354
PCP015561	4,790250739	4,807354922	5,824513297	6,285402219		
PCP015582	6,475733431	6,491853096	6,745909573	7,552054236	PCP015582	XM_009366090
PCP015611	5,64385619	6,357552005	6,222456826	6,454011343	PCP015611	XM_008367022
PCP015645	5,946496941	6,215872703	5,906890596	5,781359714		
PCP015649	6,579994693	6,448405435	6,981053471	6,539158811	PCP015649	XM_009336017
PCP015721	5,544423562	5,57500972	6,38663853	6,321928095	PCP015721	XM_009377609
PCP015722	6,824386003	6,528727582	7,06608919	6,648609245	PCP015722	XM_009347614
PCP015789	6,247927513	5,380937195	5,369117459	5,48123435	PCP015789	XM_009335970
PCP015838	6,380764075	6,50255338	5,961854808	5,824513297	PCP015838	XM_018647441
PCP015888	4,272769732	4,938756261	5,309976492	5,736875423	PCP015888	XM_008238111
PCP015991	5,807354922	6,481072857	6,029673735	7,055282436		
PCP015992	6,058965822	5,736875423	6,163297449	6,87036472	PCP015992	XM_018649386
PCP015994	6,754887502	6,87036472	6,824386003	6,639087423	PCP015994	XM_021973623
PCP016072	5,513174885	5,142821844	4,857980995	4,841469999		
PCP016079	5,763677142	4,969472865	4,790250739	4,584962501	PCP016079	XM_008374779
PCP016086	6,044394119	5,247927513	4,906890596	4,64385619	PCP016086	NM_001302317
PCP016130	5,672425342	6,448405435	6,922792504	7,536596918	PCP016130	XM_008353369
PCP016152	6,981053471	6,815959618	6,54951516	6,357552005	PCP016152	XM_008395388
PCP016154	6,914923239	7,080337882	6,878602742	6,403778984	PCP016154	XM_009364657
PCP016161	5,700439718	5,02989455	5,260402093	4,624685811	PCP016161	XM_009364109
PCP016182	5,815831566	6,339850003	6,534030467	7,392317423	PCP016182	XM_009364507
PCP016207	5,634302842	5,297558281	5,380937195	3,502075956	PCP016207	XM_009364805
PCP016213	6,129283017	5,914803677	6,459431619	7,073284692	PCP016213	XM_018648889
PCP016236	5,57500972	5,087462841	4,437627248	4	PCP016236	XM_009337840
PCP016253	6,183288001	6,279285561	6,50779464	7,087462841	PCP016253	XM_017328806
PCP016266	6,942514505	5,209453366	5,321928095	7,069637728		
PCP016321	6,291493053	6,176522247	6,183288001	5,513174885	PCP016321	XM_009374436
PCP016323	5,426264755	5,014801602	4,459431619	5,309976492	PCP016323	XM_008387666
PCP016347	5,77267747	5,59484709	5,841218374	4,682011391	PCP016347	XM_009376349
PCP016357	5,333781501	4,480911346	4,604664415	4,544114402	PCP016357	XM_009376219
PCP016372	6,745909573	6,564835417	6,50779464	7,105070402	PCP016372	XM_009363182
PCP016377	6,156437068	5,789990019	6,392317423	6,718772592		
PCP016384	5,969703088	6,22881869	6,38663853	6,926829678		
PCP016385	6,254178286	6,569855608	5,798828178	5,807354922	PCP016385	XM_009362994

PCP016387	6,31596467	6,321928095	6,475733431	7,055282436	PCP016387	XM_009362972
PCP016389	4,906890596	5,260402093	4,544114402	4,143230135	PCP016389	XM_009362946
PCP016430	6,528727582	6,54951516	6,741466986	7,300764373	PCP016430	XM_009341505
PCP016489	6,222456826	5,77267747	5,736875423	4,754887502		
PCP016503	6,824386003	6,996275749	6,653490009	6,333960351	PCP016503	NM_001293957
PCP016510	5,781359714	5,222263604	5,815831566	6,709704193		
PCP016524	6,99242856	7	6,837060204	6,414981143	PCP016524	XM_009370373
PCP016526	6,136068312	6,087462841	4,874305166	4,272769732	PCP016526	XM_009370366
PCP016560	6,291493053	6,828707735	6,894817763	5,815831566	PCP016560	XM_009359364
PCP016565	5,222263604	4,437627248	5,129283017	6,321928095	PCP016565	XM_018647740
PCP016579	5,554588852	6,189824559	5,196528361	4,480911346	PCP016579	XM_009352038
PCP016580	6,369291982	5,709566354	6,824386003	6,285402219	PCP016580	XM_009352054
PCP016600	6,54426899	6	5,781359714	6,007419784	PCP016600	XM_009353168
PCP016604	5,222263604	5,196528361	6,129283017	5,984817174		
PCP016611	6,272956308	6,459431619	6,564835417	7,058857621	PCP016611	XM_009365844
PCP016621	4,790250739	5,142821844	6,087462841	6,309794644	PCP016621	XM_009365827
PCP016623	6,094447222	5,961854808	5,824513297	5,491853096	PCP016623	XM_009365825
PCP016656	6,363346321	5,874551087	6,203005338	6,015024705	PCP016656	XM_009366590
PCP016718	7,06608919	6,584962501	6,539158811	6,007419784	PCP016718	XM_009341121
PCP016767	6,695854658	7,014913158	6,878602742	6,763809907	PCP016767	XM_009350481
PCP016776	5,073391816	2,414135533	5,333781501	5,260402093	PCP016776	XM_009336194
PCP016856	5,115615931	5,59484709	6,015024705	6,426264755	PCP016856	XM_008390327
PCP016894	6,890811455	6,996275749	7,033423002	6,518377768	PCP016894	XM_009377200
PCP016937	7,076815597	6,475733431	6,745909573	6,741466986	PCP016937	XM_008387102
PCP016999	6,058965822	6,189824559	6,454011343	7,17990909	PCP016999	XM_008387936
PCP017010	6,222456826	6,209453366	6,087462841	5,392317423	PCP017010	XM_018648178
PCP017038	5,763677142	5,798828178	5,961854808	5,297558281	PCP017038	XM_009348078
PCP017056	7,375039431	7,022367813	6,662917555	6,31596467	PCP017056	XM_008381785
PCP017094	6,254178286	5,930737338	6,403778984	6,785681319	PCP017094	XM_009353062
PCP017102	4,73714592	4,718635616	5,663059924	4,841469999	PCP017102	XM_008395264
PCP017205	5,380937195	4,523561956	4,392317423	5,333781501	PCP017205	XM_009360789
PCP017208	5,196528361	5,681730355	6,189824559	6,351734323	PCP017208	XM_008361176
PCP017212	6,754887502	6,497133304	6,81159947	7,315874125		
PCP017214	5,700439718	5,48123435	5,60496087	6,345715709	PCP017214	XM_009371554
PCP017238	5,369117459	4,985044962	5,691254979	6	PCP017238	XM_009342641
PCP017245	4,754887502	5,746043983	5,584962501	6,714245518	PCP017245	XM_009340139
PCP017328	6,910852562	7,022367813	6,785681319	6,750472519	PCP017328	XM_008380200
PCP017346	6,882643049	6,426264755	6,380764075	5,196528361	PCP017346	XM_008350207
PCP017358	5,815831566	5,321928095	5,073391816	4,143230135	PCP017358	XM_009351347
PCP017391	5,961854808	6,022367813	6,163297449	7,321928095		
PCP017403	6,380764075	6,129283017	6,38663853	6,922792504		
PCP017415	6,22881869	5,930737338	6,189824559	5,718909554	PCP017415	XM_009352580
PCP017456	4,874305166	3,736604875	5,491853096	4,857980995		
PCP017458	5,727920455	5,02989455	5,437294331	4,754887502	PCP017458	XM_008371482
PCP017479	4,34553831	3,906890596	4,247927513	6,122465658		
PCP017547	5,977279923	5,882643049	6,375039431	6,938638658		

PCP017612	6,50255338	6,564835417	6,72342204	7,257387843	PCP017612	XM_009362653
PCP017630	6,914923239	6,183288001	6,486553493	6,60481265	PCP017630	XM_009367865
PCP017645	6,029673735	5,392317423	5,235344128	5,415150205	PCP017645	XM_009347147
PCP017660	7,146390476	6,768184325	6,183288001	6,22881869	PCP017660	XM_009378673
PCP017688	6,614709844	5,914803677	5,736875423	5,321928095		
PCP017752	6,837060204	6,677085351	6,260590275	6,163297449	PCP017752	XM_009342554
PCP017765	5,874551087	5,564683017	5,798828178	5,183089461	PCP017765	XR_527841
PCP017805	5,142821844	4,95419631	4,906890596	3,772941338		
PCP017810	5,624393382	5,906890596	6,087462841	6,513016928	PCP017810	XM_009371463
PCP017870	6,72342204	6,351734323	6,579994693	6,22881869	PCP017870	XM_008365700
PCP017901	6,448405435	6,189824559	6,375039431	6,087462841	PCP017901	XR_665800
PCP017912	6,03716255	5,984817174	6,794415866	6,475733431	PCP017912	XM_009361061
PCP017913	5,554588852	5,502394256	5,727920455	4,523561956	PCP017913	XM_009360762
PCP017931	5,470211457	5,841218374	5,183089461	3,736604875	PCP017931	XM_018647567
PCP017969	5,403608584	5,087462841	5,59484709	6,667750232		
PCP017984	6,357552005	5,736875423	6,03716255	5,874551087	PCP017984	XM_008375465
PCP017994	5,513174885	5,02989455	4,906890596	4,564987801	PCP017994	XM_018650699
PCP018008	5,403608584	5,513174885	6,03716255	6,397974049	PCP018008	XM_009346786
PCP018030	4,480911346	4,73714592	5,491853096	6,094447222	PCP018030	XM_009340187
PCP018043	4,059182199	4,247927513	4,95419631	5,297558281	PCP018043	XM_008342697
PCP018056	6,108524457	5,992541859	5,906890596	4,196134881		
PCP018080	6,015024705	6,369291982	6,486553493	7,06608919		
PCP018093	6,741466986	6,475733431	6,297741678	6,247927513	PCP018093	XM_008357860
PCP018095	6,297741678	5,196528361	4,662775172	4	PCP018095	XM_017332100
PCP018116	5,014801602	5,653346693	5,448570626	6,303780748	PCP018116	XM_017324188
PCP018117	7,153095972	6,709704193	6,574858391	5,513174885	PCP018117	XM_009379031
PCP018149	3,700439718	5,380937195	5,849749117	5,087462841	PCP018149	XM_009359710
PCP018189	5,700439718	6,662917555	6,07317756	4,890933022	PCP018189	XM_017328830
PCP018200	3,502075956	5,624393382	5,426264755	4,059182199	PCP018200	XM_017329734
PCP018220	6,459431619	6,624539604	6,969587981	7,209453366	PCP018220	XM_008380648
PCP018233	6,357552005	5,681730355	6,196331634	5,824513297	PCP018233	XM_008364236
PCP018251	6,309794644	6,523561956	6,579994693	7,022367813	PCP018251	XM_008375346
PCP018254	5,02989455	5,437294331	5,513174885	6,115407855		
PCP018261	6,235152624	6,380764075	6,403778984	5,59484709		
PCP018266	6,380764075	5,48123435	6,022367813	5,824513297	PCP018266	XM_009370029
PCP018281	4,95419631	5,345893086	6,03716255	5,789990019	PCP018281	XM_008378971
PCP018283	5,663059924	6,691115365	7,156538193	6,64385619	PCP018283	XM_008378964
PCP018295	6,624539604	6,07317756	6,737010678	6,906890596	PCP018295	NM_001328771
PCP018307	5,156639311	4,906890596	5,357552005	5,946496941		
PCP018346	6,609843592	6,464831606	6,486553493	7,244791942	PCP018346	XM_017332212
PCP018389	6,815959618	6,72342204	6,727920455	6,03716255	PCP018389	XM_009351917
PCP018415	5,087462841	5,807354922	5,672425342	4,700439718	PCP018415	XM_009369516
PCP018417	6,807354922	6,882643049	6,969587981	6,4374608	PCP018417	XM_008387906
PCP018446	6,513016928	6	6,303780748	6,815959618	PCP018446	XM_018650200
PCP018461	4,297925053	4,772413555	5,544423562	6,022367813	PCP018461	XM_009374372
PCP018465	6,50255338	6,554588852	6,491853096	7,21916852	PCP018465	XM_009374362

PCP018479	5,914803677	6,691115365	6,163297449	6,291493053	PCP018479	XM_009339525
PCP018482	4,700439718	5,789990019	5,087462841	5,533874777	PCP018482	XM_009339521
PCP018486	6,222456826	6,345715709	6,589913261	7,044394119	PCP018486	XM_009339509
PCP018518	6,459431619	6,559644763	5,718909554	5,129283017	PCP018518	XM_009349645
PCP018572	5,584962501	6,058965822	6,922792504	7,437377568	PCP018572	XM_008346511
PCP018573	6,518377768	6,815959618	6,700439718	6,345715709	PCP018573	XM_008355111
PCP018586	7,42063398	7,392317423	7,080337882	5,273142859	PCP018586	XM_008379381
PCP018589	5,781359714	5,101397952	5,285402219	4,969472865	PCP018589	XM_009350157
PCP018708	7,855927425	7,567347696	5,890689878	0		
PCP018727	5,977279923	5,222263604	5,357552005	5,196528361	PCP018727	XM_009336299
PCP018735	4,682011391	4,624685811	5,815831566	5,183089461	PCP018735	XM_009367141
PCP018762	6,589913261	6,737010678	6,363346321	7,276124405		
PCP018764	5,533874777	5,448570626	5,898933872	6,279285561		
PCP018777	4,906890596	4,841469999	6,589913261	4,790250739	PCP018777	XM_009347948
PCP018815	6,369291982	6,579994693	6,357552005	6,189824559	PCP018815	XM_018644856
PCP018822	3,874796966	4,700439718	4,624685811	5,824513297	PCP018822	XM_009347695
PCP018848	5,849749117	5,807354922	5,459431619	3,221877081	PCP018848	XM_009372955
PCP018892	6,115407855	6,47037417	6,890811455	7,730164413	PCP018892	XM_021962715
PCP018907	6,464831606	6,029673735	6,029673735	5,709566354	PCP018907	XM_009340404
PCP018923	5,709566354	5,663059924	5,544423562	4,73714592		
PCP018934	6,475733431	6,403778984	6,539158811	7,139551352	PCP018934	XM_017335483
PCP019005	6,087462841	6,189824559	5,866166169	5,533874777	PCP019005	XM_018642689
PCP019013	5,357552005	5,073391816	4,857980995	4,584962501	PCP019013	XM_008381440
PCP019029	4,985044962	5,142821844	4,700439718	4,143230135	PCP019029	XM_009375083
PCP019046	6,101608059	5,502394256	4,564987801	3,840966704		
PCP019056	6,60481265	5,866166169	5,746043983	5,470211457	PCP019056	XM_009379818
PCP019099	6,741466986	6,380764075	6,431790083	6,24164954	PCP019099	XM_017326897
PCP019152	5,235344128	5,513174885	5,961854808	6,431790083	PCP019152	XM_009358194
PCP019153	5,890689878	5,95419631	5,890689878	6,781359714	PCP019153	XM_008394899
PCP019159	4,938756261	5,345893086	5,403608584	5,890689878		
PCP019167	6,50255338	6,64385619	6,824386003	6,357552005	PCP019167	XM_018643495
PCP019179	6,574858391	6,614709844	6,579994693	6,297741678	PCP019179	XR_667777
PCP019232	6,409390936	5,898933872	6,285402219	5,48123435	PCP019232	XM_009366464
PCP019274	6,247927513	6,080444483	6,24164954	7,619633096		
PCP019284	6,614709844	6,272956308	6,559644763	5,930737338	PCP019284	XR_669877
PCP019288	7,058857621	6,285402219	6,906890596	6,54951516	PCP019288	XM_009377443
PCP019325	6,022367813	6,448405435	6,554588852	5,95419631		
PCP019370	5,222263604	4,087462841	4,700439718	4,196134881		
PCP019419	7,06608919	6,599912842	6,798698597	6,718772592	PCP019419	XM_018651633
PCP019439	6,094447222	5,736875423	6,486553493	5,437294331	PCP019439	XM_009339493
PCP019467	5,977279923	5,824513297	5,297558281	4,272769732	PCP019467	XM_009338875
PCP019487	4,700439718	5,718909554	5,691254979	6,254178286	PCP019487	XM_009377658
PCP019498	5,222263604	4,297925053	4,772413555	6,183288001	PCP019498	XM_009377638
PCP019506	6,303780748	6,07317756	6,07317756	5,857980995	PCP019506	XM_009377627
PCP019526	6,080444483	6,087462841	6,007419784	5,426264755	PCP019526	XM_008378877
PCP019562	7,193081765	6,614709844	6,196331634	6,136068312	PCP019562	XM_008228004

PCP019571	4,807354922	5,392317423	5,369117459	4,502712486	PCP019571	XM_009372476
PCP019583	4,700439718	4,087462841	6,303780748	5,345893086	PCP019583	XM_018649082
PCP019586	4,480911346	4,985044962	5,459431619	5,815831566	PCP019586	XM_009365729
PCP019597	6,339850003	6,72342204	7,007531912	7,06608919	PCP019597	XM_018649078
PCP019602	6,189824559	6,619559738	6,68187088	7,30679083	PCP019602	XM_009364313
PCP019631	6,60481265	5,922911406	6,392317423	6,333960351	PCP019631	XM_009339576
PCP019640	6,648609245	6,750472519	7,094552786	6,14974712	PCP019640	XM_009339558
PCP019653	6,50255338	6,667750232	6,599912842	6,309794644	PCP019653	XM_008370512
PCP019661	5,984817174	6,080444483	5,653346693	5,64385619	PCP019661	XM_009380677
PCP019671	4,682011391	4,807354922	5,285402219	5,984817174		
PCP019682	6,534030467	6,266786541	6,266786541	5,321928095	PCP019682	XM_009357525
PCP019692	6,266786541	5,930737338	5,849749117	5,584962501	PCP019692	XM_009357814
PCP019734	6,222456826	6,03716255	5,992541859	5,64385619	PCP019734	XM_009368775
PCP019752	5,866166169	5,634302842	5,392317423	5,380937195	PCP019752	XM_009349019
PCP019776	6,518377768	6,4374608	5,824513297	5,260402093	PCP019776	XM_018646688
PCP019791	6,31596467	6,564835417	6,874428132	7,564759219	PCP019791	XM_009354616
PCP019799	6,882643049	6,247927513	6,727920455	7,238404739	PCP019799	XM_009354637
PCP019809	6,115407855	5,930737338	5,437294331	2,939226578	PCP019809	XM_008379467
PCP019829	4,564987801	5,832890014	6,554588852	8,17990909	PCP019829	XM_009335625
PCP019871	4,459431619	4,874305166	4,95419631	5,815831566	PCP019871	XM_009380423
PCP019872	6,07317756	5,297558281	5,169925001	4,480911346	PCP019872	XM_016795453
PCP019876	5,718909554	5,64385619	5,807354922	6,837060204		
PCP019878	5,169925001	4,624685811	5,142821844	5,992541859	PCP019878	XM_009368981
PCP019888	5,459431619	5,691254979	6,029673735	6,420718183	PCP019888	XM_009368964
PCP019939	3,368768349	5,073391816	5,77267747	4,544114402	PCP019939	XM_008344425
PCP019976	6,136068312	5,95419631	6,108524457	5,523561956	PCP019976	XM_009374638
PCP020013	5,984817174	6,156437068	6,321928095	5,624393382	PCP020013	XM_009355599
PCP020038	6,051589621	6,486553493	6,785681319	7,599912842	PCP020038	XM_009345977
PCP020045	6,369291982	5,754887502	5,754887502	5,922911406		
PCP020062	6,051589621	5,890689878	5,502394256	5,044394119	PCP020062	XM_008353642
PCP020074	5,502394256	5,357552005	5,624393382	4,922673593	PCP020074	XM_009355941
PCP020075	6,03716255	5,914803677	5,930737338	5,345893086	PCP020075	XM_008344599
PCP020083	5,115615931	5,073391816	5,044394119	6,442943496	PCP020083	XM_009356626
PCP020084	5,415150205	5,502394256	6,169925001	6,260590275	PCP020084	XM_009356626
PCP020091	4,369466484	5,523561956	6,143026004	7,924812504	PCP020091	XM_009360209
PCP020097	6,662917555	6,339850003	7,055282436	6,044394119	PCP020097	XM_008374811
PCP020135	6,662917555	6,569855608	6,84962403	7,212666605	PCP020135	XM_009355405
PCP020178	5,101397952	5,156639311	5,533874777	6,080444483	PCP020178	XM_017334067
PCP020200	5,874551087	6,087462841	6,254178286	5,672425342	PCP020200	XM_009366524
PCP020216	5,247927513	4,922673593	4,790250739	4,143230135	PCP020216	XM_018649094
PCP020217	5,77267747	5,789990019	5,691254979	5,209453366	PCP020217	XM_008395894
PCP020273	6,156437068	5,781359714	6,22881869	5,357552005	PCP020273	XM_009344352
PCP020280	4,64385619	4,807354922	5,824513297	5,95419631	PCP020280	XM_009347498
PCP020326	6,539158811	6,853870927	6,727920455	7,231989133		
PCP020345	4,922673593	5,969703088	5,969703088	6,272956308	PCP020345	XM_018646938
PCP020363	6,497133304	5,914803677	6,272956308	6,044394119	PCP020363	XM_021954646

PCP020371	5,058749412	5,681730355	4,985044962	4,604664415		
PCP020379	5,554588852	5,718909554	6,007419784	6,380764075	PCP020379	XM_009360513
PCP020402	5,849749117	5,073391816	4,272769732	4,95419631	PCP020402	XM_009364652
PCP020412	5,807354922	5,196528361	5,073391816	4,922673593	PCP020412	XM_009355334
PCP020418	6,86628983	6,203005338	6,196331634	6,266786541	PCP020418	XM_009355339
PCP020429	6,143026004	6,380764075	6,653490009	7,153095972	PCP020429	XM_008351666
PCP020434	6,619559738	6,4374608	7,007531912	4,480911346	PCP020434	XM_018644728
PCP020444	6,584962501	6,890811455	6,772545519	6,648609245	PCP020444	XM_009380452
PCP020458	6,38663853	6,763809907	6,86628983	7,055282436	PCP020458	XM_009374044
PCP020495	6,327866971	6,454011343	6,209453366	5,392317423	PCP020495	XM_009361690
PCP020505	6,235152624	6,677085351	6,824386003	7,459431619		
PCP020507	5,437294331	5,849749117	5,736875423	6,431790083	PCP020507	XM_009361523
PCP020543	4,459431619	4,247927513	5,746043983	4,985044962	PCP020543	XM_009355648
PCP020562	6,309794644	6,732404887	6,81159947	7,115511897	PCP020562	XM_009344480
PCP020573	5,273142859	5,02989455	5,260402093	6,163297449	PCP020573	XM_009362738
PCP020605	6,14974712	5,564683017	6,369291982	6,203005338	PCP020605	XM_018643934
PCP020618	6,189824559	6,143026004	5,849749117	5,403608584	PCP020618	XM_008352169
PCP020646	6,087462841	6,169925001	6,087462841	5,60496087	PCP020646	XM_009369010
PCP020651	5,718909554	5,222263604	4,824258697	4,502712486		
PCP020668	5,380937195	5,044394119	5,02989455	4,34553831	PCP020668	XM_008391740
PCP020692	5,357552005	4,857980995	4,790250739	4,624685811		
PCP020731	6,790120385	6,906890596	6,984931073	6,564835417	PCP020731	XM_009352840
PCP020748	4,34553831	4,502712486	4,662775172	5,898933872		
PCP020891	6,589913261	6,528727582	6,845490051	7,199672345	PCP020891	XM_009376246
PCP020896	5,297558281	4,790250739	4,922673593	4,247927513	PCP020896	XM_018651493
PCP020900	6,189824559	6,309794644	5,977279923	5,653346693	PCP020900	XM_009374470
PCP020932	7,062531903	6,459431619	6,754887502	5,798828178	PCP020932	XM_008368738
PCP020937	5,718909554	4,700439718	5,333781501	5,321928095	PCP020937	XM_021955708
PCP020945	6,203005338	6,051589621	6,07317756	5,544423562	PCP020945	XM_008352593
PCP020973	5	5,073391816	5,544423562	6,403778984	PCP020973	XM_008390892
PCP021016	5,544423562	5,209453366	4,857980995	4,414812061	PCP021016	XM_009359727
PCP021021	4,297925053	5,554588852	5,763677142	5	PCP021021	XM_009353199
PCP021025	6,426264755	5,754887502	5,369117459	5,470211457	PCP021025	XM_009353216
PCP021026	5,345893086	5,491853096	5,321928095	4,624685811	PCP021026	XM_008341317
PCP021028	6,559644763	5,653346693	5,866166169	5,961854808	PCP021028	XM_009353218
PCP021040	5,142821844	5,403608584	5,309976492	6,058965822		
PCP021057	6,442943496	6,24164954	5,437294331	4,169925001	PCP021057	XM_009337903
PCP021059	5,415150205	4,604664415	4,564987801	4,662775172		
PCP021087	6,50255338	6,486553493	6,609843592	7,285402219	PCP021087	XM_009356672
PCP021094	6,254178286	6,136068312	6,235152624	6,946379968	PCP021094	XR_667060
PCP021114	6,513016928	6,07317756	6	7,609769734	PCP021114	XM_009365486
PCP021129	6,481072857	5,961854808	5,763677142	5,922911406	PCP021129	XM_018649034
PCP021138	5,169925001	5,624393382	5,273142859	4,890933022	PCP021138	XM_009365607
PCP021188	6,754887502	7,132679654	6,80309785	6,768184325		
PCP021214	4,772413555	5,285402219	5,513174885	3,624100895	PCP021214	XM_008389126
PCP021232	6,539158811	6,375039431	6,594996337	7,30679083		

PCP021233	4,247927513	3,624100895	4,754887502	5,857980995	PCP021233	XM_009353735
PCP021240	5,357552005	5,523561956	6,03716255	6,454011343	PCP021240	XM_009355251
PCP021254	6,50255338	6,50779464	6,272956308	5,815831566	PCP021254	XM_017332056
PCP021262	6,143026004	6,297741678	6,291493053	5,930737338	PCP021262	XM_017332464
PCP021267	5,285402219	5,101397952	5,763677142	6,285402219	PCP021267	XM_017323466
PCP021315	6,599912842	6,47037417	6,327866971	6,203005338	PCP021315	XM_009345531
PCP021335	5,183089461	5,101397952	3,906890596	4,584962501	PCP021335	XM_018644639
PCP021359	6,60481265	6,64385619	6,914923239	6,414981143		
PCP021406	5,321928095	5,938521046	6,718772592	7,193081765	PCP021406	XM_021962869
PCP021411	6,351734323	5,415150205	5,938521046	6,351734323	PCP021411	XM_009346004
PCP021442	5,533874777	5,57500972	5,523561956	4,969472865	PCP021442	XM_018646325
PCP021489	6,910852562	6,051589621	6,619559738	4,754887502	PCP021489	XM_009372341
PCP021495	5,357552005	5,48123435	5,95419631	5,273142859		
PCP021498	5,634302842	5,64385619	6,163297449	6,832890014	PCP021498	XM_009372231
PCP021507	5,513174885	5,849749117	6,03716255	6,634157606	PCP021507	XM_009373866
PCP021509	6,272956308	6,624539604	6,564835417	6,996275749	PCP021509	XM_018650518
PCP021515	6,634157606	6,965784285	6,988684687	6,539158811	PCP021515	XM_009341165
PCP021558	6,47037417	7,415065677	6,768184325	6,420718183	PCP021558	XM_009375376
PCP021569	6,136068312	6,260590275	6,163297449	5,653346693	PCP021569	XM_017333412
PCP021575	6,101608059	6,518377768	6,662917555	4,969472865	PCP021575	XM_009369023
PCP021587	6,044394119	5,415150205	5,59484709	5,285402219	PCP021587	XM_008338702
PCP021594	5,321928095	4,523561956	5,235344128	6,189824559	PCP021594	XM_009340150
PCP021603	4,700439718	4,584962501	5,115615931	5,663059924	PCP021603	XM_009349013
PCP021630	5,235344128	5,653346693	5,415150205	6,339850003		
PCP021653	4,297925053	4,34553831	5,523561956	4,604664415	PCP021653	XM_008349028
PCP021689	3,840966704	4,272769732	4,985044962	5,142821844	PCP021689	XM_008351242
PCP021749	6,260590275	5,930737338	5,874551087	5,544423562		
PCP021751	6,14974712	6,196331634	6,486553493	6,926829678	PCP021751	XM_009358826
PCP021754	5,922911406	6,497133304	6,86628983	7,159871337	PCP021754	XM_009374734
PCP021761	6,321928095	6,369291982	6,634157606	7,115511897	PCP021761	XM_009359987
PCP021784	5,273142859	4,662775172	4,564987801	3,969933275	PCP021784	XM_008361015
PCP021834	5,297558281	4,922673593	5,247927513	5,977279923		
PCP021908	6,961970533	6,574858391	6,750472519	6,403778984	PCP021908	XM_009337197
PCP021940	6,209453366	5,247927513	6,122465658	6,363346321	PCP021940	XM_009381365
PCP021965	6,569855608	6,54951516	6,890811455	7,333870928	PCP021965	XM_009349424
PCP021976	3,502075956	5,222263604	5,014801602	6,351734323		
PCP021985	5,235344128	5,614709844	6,475733431	9,19640541	PCP021985	XM_018643952
PCP021996	6,68187088	6,196331634	7,294620749	6,022367813		
PCP022025	5,890689878	5,459431619	6,03716255	6,768184325		
PCP022029	5,672425342	5,183089461	5,247927513	4,718635616		
PCP022071	5,470211457	5,763677142	7,076815597	6,163297449	PCP022071	XM_009359856
PCP022126	3,624100895	4,414812061	4,772413555	5,48123435		
PCP022129	5,866166169	6,327866971	6,475733431	7,209453366	PCP022129	XM_009354771
PCP022142	5,64385619	5,789990019	6,094447222	6,653490009	PCP022142	XM_008385757
PCP022146	6,06608919	5,866166169	6,491853096	5,297558281	PCP022146	XM_009353044
PCP022157	5,533874777	7,055282436	4,087462841	4,700439718		

PCP022169	5,718909554	5,273142859	4,437627248	5,115615931	PCP022169	XM_009352986
PCP022181	4,790250739	5,235344128	6,14974712	6,203005338		
PCP022194	5,247927513	5,491853096	5,260402093	4,682011391		
PCP022198	5,77267747	6,254178286	6,06608919	5,763677142		
PCP022221	4,790250739	4,73714592	5,285402219	4,34553831	PCP022221	XM_017327504
PCP022267	6,984931073	6,781359714	6,930737338	6,72342204	PCP022267	XM_009367376
PCP022273	5,906890596	5,437294331	6,333960351	6,209453366	PCP022273	XM_009368142
PCP022283	4,682011391	4,95419631	6,203005338	5,736875423	PCP022283	XM_009344371
PCP022288	6,745909573	6,667750232	6,841344192	6,339850003		
PCP022315	6,781359714	6,528727582	6,266786541	6,129283017	PCP022315	XM_008340531
PCP022326	5,754887502	5,663059924	5,169925001	4,059182199	PCP022326	XM_018643085
PCP022442	6,491853096	6,497133304	6,832890014	7,406587689	PCP022442	XM_009354301
PCP022447	5,222263604	5,044394119	6,97349648	5,403608584	PCP022447	XM_009347943
PCP022460	5,02989455	5,101397952	5,584962501	4,584962501	PCP022460	XM_009366467
PCP022495	5,691254979	6,351734323	7,044394119	6,950351762		
PCP022521	5,857980995	5,235344128	6,080444483	6,363346321	PCP022521	XM_009343098
PCP022529	5,196528361	5,129283017	5,380937195	4,297925053	PCP022529	XM_018646906
PCP022538	4,544114402	3,969933275	4,890933022	5,513174885	PCP022538	XM_009355509
PCP022572	5,554588852	6,080444483	6,156437068	6,934634441		
PCP022591	5,746043983	4,906890596	5,222263604	4,73714592	PCP022591	XM_009337304
PCP022612	5,984817174	6,169925001	6,357552005	6,969587981	PCP022612	XM_009337543
PCP022636	5,95419631	6,122465658	6,254178286	6,930737338	PCP022636	XM_009357103
PCP022657	5,874551087	5,369117459	5,906890596	5,285402219	PCP022657	XM_009357061
PCP022679	6,143026004	5,938521046	6	6,732404887		
PCP022692	6,044394119	5,746043983	5,95419631	5,533874777	PCP022692	XM_009379773
PCP022696	7,047996356	7,062531903	6,639087423	6,599912842	PCP022696	XM_009379782
PCP022720	6,115407855	5,491853096	5,849749117	4,297925053		
PCP022730	6,122465658	5,691254979	6,345715709	6,574858391	PCP022730	XM_018652374
PCP022771	6,950351762	6,189824559	6,910852562	6,291493053	PCP022771	XM_008388188
PCP022813	6,22881869	4,969472865	5,513174885	5,222263604	PCP022813	XM_009367767
PCP022815	6,176522247	6,06608919	5,906890596	4,841469999	PCP022815	XM_009367762
PCP022849	7,303780748	6,961970533	6,667750232	6,454011343	PCP022849	XM_008383645
PCP022875	5,977279923	6,196331634	5,60496087	5,736875423	PCP022875	XM_009352437
PCP022883	6,339850003	6,22881869	6,614709844	6,988684687		
PCP022897	5,653346693	6,594996337	7,727920455	6,853870927	PCP022897	XM_009368026
PCP022903	4,700439718	7,609769734	5,815831566	8,194756854	PCP022903	XM_008375899
PCP022909	4,321928095	4,34553831	4,985044962	6,528727582	PCP022909	XM_008375895
PCP022910	5,824513297	5,247927513	6,03716255	7,176621973	PCP022910	XM_009360427
PCP022911	3,840966704	4,437627248	4,754887502	5,746043983	PCP022911	XM_009360425
PCP022920	6,156437068	6,087462841	6,54426899	7,523561956	PCP022920	XM_008380338
PCP022925	4,502712486	5,115615931	5,48123435	6,497133304	PCP022925	XM_008380413
PCP022967	5,285402219	5,60496087	4,502712486	3,906890596	PCP022967	XM_008358112
PCP022990	5,448570626	5,073391816	5,087462841	3,840966704	PCP022990	XM_009368087
PCP023036	4,95419631	4,772413555	5	6,080444483		
PCP023062	5,115615931	5,222263604	4,564987801	4,222650022	PCP023062	XM_008355791
PCP023070	6,07317756	5,874551087	6,235152624	5,448570626		

PCP023088	5,841218374	5,513174885	5,513174885	4,874305166	PCP023088	XM_009365048
PCP023105	6,215872703	6,129283017	6,950351762	5,977279923	PCP023105	XM_009343683
PCP023125	6,247927513	5,426264755	5,634302842	5,961854808	PCP023125	XM_017325084
PCP023209	5,691254979	5,502394256	5,663059924	5,058749412	PCP023209	XM_009363220
PCP023217	7,029784146	6,50255338	6,513016928	6,745909573	PCP023217	XM_021956024
PCP023226	6,235152624	6,31596467	6,426264755	7,069637728		
PCP023253	5,014801602	5,672425342	5,345893086	3,321928095	PCP023253	XM_009354140
PCP023256	6,579994693	6,574858391	6,539158811	6,176522247	PCP023256	XM_008368675
PCP023275	6,303780748	6,491853096	6,574858391	6,044394119		
PCP023290	5,345893086	4,890933022	5,222263604	4,196134881		
PCP023293	5,653346693	5,297558281	4,95419631	4,682011391	PCP023293	XM_009359100
PCP023328	5,183089461	5,584962501	6,03716255	5,459431619	PCP023328	XM_008362183
PCP023339	3,772941338	3,736604875	5,235344128	6,115407855	PCP023339	XM_009361197
PCP023349	6,129283017	5,77267747	5,832890014	5,196528361	PCP023349	XM_009364070
PCP023367	5,727920455	5,533874777	6,058965822	6,961970533	PCP023367	XM_009373527
PCP023373	5,297558281	5,564683017	5,129283017	3,624100895	PCP023373	NM_001302325
PCP023378	6,732404887	6,718772592	6,72342204	6,345715709	PCP023378	XM_008372624
PCP023393	6,442943496	6	6,203005338	5,736875423	PCP023393	XM_009374186
PCP023425	6,31596467	6,143026004	5,754887502	5,922911406	PCP023425	XM_018647818
PCP023445	6,837060204	6,853870927	6,599912842	6,497133304		
PCP023455	5,807354922	5,59484709	4,807354922	4,584962501		
PCP023468	6,513016928	6,351734323	6,609843592	7,212666605	PCP023468	XM_018647103
PCP023511	6,369291982	6,497133304	6,574858391	7,058857621	PCP023511	XM_009380788
PCP023530	6	5,727920455	5,437294331	4,985044962	PCP023530	XM_017327706
PCP023532	2,414135533	7,235248379	5,222263604	6,31596467	PCP023532	XM_008343808
PCP023537	6,741466986	6,266786541	6,129283017	6,029673735	PCP023537	NM_001328727
PCP023549	6,087462841	6,115407855	6,015024705	5,672425342	PCP023549	XM_009349145
PCP023554	6,54426899	6,392317423	6,087462841	5,946496941	PCP023554	XM_008362482
PCP023561	4,95419631	4,564987801	5,02989455	5,746043983	PCP023561	XM_018650343
PCP023564	6,528727582	6,380764075	6,727920455	7,215969746	PCP023564	XM_009371430
PCP023586	6,369291982	6,285402219	6,203005338	5,754887502	PCP023586	XM_008353427
PCP023644	4,874305166	5,415150205	5,930737338	5,663059924	PCP023644	XM_009335634
PCP023647	5,672425342	5,969703088	6,087462841	6,564835417	PCP023647	XM_009345580
PCP023681	5,663059924	6,129283017	5,890689878	5,718909554		
PCP023720	5,789990019	5,48123435	5,564683017	4,807354922	PCP023720	XM_018648609
PCP023725	5,922911406	5,691254979	5,815831566	4,938756261	PCP023725	XM_009356310
PCP023750	4,807354922	4,64385619	6,486553493	5,736875423	PCP023750	XM_009344693
PCP023759	5,513174885	4,906890596	4,34553831	4,196134881	PCP023759	XM_017335293
PCP023788	7,636624621	6,31596467	6,260590275	6,14974712	PCP023788	NM_001328792
PCP023802	6,297741678	6,327866971	6,235152624	5,95419631		
PCP023818	6,426264755	6,087462841	6,115407855	5,564683017	PCP023818	XM_009374903
PCP023836	5,222263604	5,533874777	5,653346693	6,380764075		
PCP023912	6,857980995	6,559644763	6,058965822	6,464831606		
PCP023920	3,874796966	4,437627248	4,73714592	5,584962501		
PCP023941	6,54426899	6,303780748	6,029673735	5,129283017	PCP023941	XM_018651292
PCP023958	5,101397952	5,977279923	6,375039431	6,203005338		

PCP023980	5,969703088	5,044394119	4,584962501	4,584962501	PCP023980	XM_009341151
PCP023989	5,64385619	5,874551087	6,303780748	6,624539604		
PCP024006	4,272769732	5,297558281	5,727920455	4,841469999	PCP024006	XM_009336749
PCP024112	6,614709844	6,4374608	6,62935662	6,203005338	PCP024112	XM_009368427
PCP024113	5,634302842	5,321928095	5,273142859	6,115407855	PCP024113	XM_009368445
PCP024156	6,345715709	6,403778984	6,50255338	7,440204752	PCP024156	XM_009345003
PCP024159	6,14974712	6	6,169925001	5,614709844	PCP024159	XM_008355502
PCP024172	6,108524457	5,672425342	5,938521046	5,196528361		
PCP024202	5,142821844	5,815831566	5,841218374	6,772545519	PCP024202	XM_008391557
PCP024205	6,672425342	6,686500527	6,464831606	5,663059924		
PCP024227	6,143026004	6,029673735	5,470211457	4,087462841	PCP024227	XM_008391526
PCP024235	6,491853096	6,523561956	6,857980995	6,442943496		
PCP024242	5,691254979	5,222263604	5,058749412	4,564987801		
PCP024246	6,777025123	5,977279923	6,222456826	5,369117459	PCP024246	XM_008391505
PCP024257	5,369117459	5,058749412	5,634302842	7,473056289	PCP024257	XM_008388402
PCP024261	5,746043983	5,297558281	4,754887502	3,969933275		
PCP024278	5,930737338	5,754887502	6,156437068	6,845490051	PCP024278	XM_018645359
PCP024280	4,414812061	4,459431619	4,700439718	5,663059924	PCP024280	XM_009348833
PCP024332	4,64385619	4,143230135	4,321928095	5,754887502	PCP024332	XM_009362609
PCP024346	4,95419631	5,754887502	5,380937195	5,222263604	PCP024346	XM_018648424
PCP024350	6,700439718	6,222456826	5,736875423	7,047996356	PCP024350	XM_009362580
PCP024355	7,014913158	7,06608919	6,648609245	6,297741678	PCP024355	XM_018646923
PCP024371	6,101608059	6,481072857	6,183288001	6,176522247		
PCP024443	6,513016928	5,624393382	5,789990019	4,718635616	PCP024443	XM_009377072
PCP024450	6,196331634	6,058965822	5,691254979	4,824258697	PCP024450	XM_009376885
PCP024466	3,700439718	4,414812061	4,938756261	5,369117459	PCP024466	XM_009369489
PCP024477	6,169925001	5,333781501	5,882643049	5,523561956	PCP024477	XM_009349711
PCP024488	7,08395793	7,029784146	6,72342204	6,380764075	PCP024488	XM_009376792
PCP024562	4,459431619	5,309976492	6,087462841	5,672425342	PCP024562	XM_009337531
PCP024604	5,183089461	5,392317423	6,38663853	6,727920455	PCP024604	XM_009350675
PCP024607	6,459431619	6,38663853	6,906890596	6,303780748	PCP024607	XM_009367825
PCP024641	5,554588852	5,285402219	5,285402219	6,72342204	PCP024641	XM_018652153
PCP024645	5,977279923	6,06608919	6,431790083	7,037052702	PCP024645	XM_009379462
PCP024651	5,866166169	5,513174885	6,50779464	6,785681319	PCP024651	XM_009369462
PCP024654	7,08395793	7,051698368	6,772545519	6,22881869	PCP024654	XM_009369465
PCP024659	6,459431619	5,969703088	6,297741678	5,718909554	PCP024659	XM_009354179
PCP024662	5,523561956	5	5,247927513	3,736604875		
PCP024667	5,992541859	6,015024705	5,930737338	5,57500972		
PCP024704	5,196528361	4,662775172	4,890933022	3,906890596		
PCP024710	6,481072857	6,534030467	6,667750232	6,24164954	PCP024710	XM_009365662
PCP024718	6,196331634	6,4374608	6,737010678	6,950351762	PCP024718	XR_001953425
PCP024722	5,222263604	5,470211457	5,235344128	4,059182199		
PCP024757	4,922673593	5,235344128	3,624100895	4,824258697		
PCP024767	6,727920455	6,845490051	6,772545519	6,363346321	PCP024767	XM_009369803
PCP024783	5,906890596	6,189824559	6,247927513	5,781359714		
PCP024805	7,247927513	6,559644763	5,866166169	6,285402219	PCP024805	XM_009372763

PCP024822	6,24164954	6,136068312	5,513174885	4,414812061	PCP024822	NM_001319810
PCP024823	5,014801602	5,459431619	6,22881869	6,497133304		
PCP024843	5,60496087	5,48123435	6,403778984	7,251056285	PCP024843	XM_009336933
PCP024853	5,672425342	6,051589621	6,14974712	6,820178962		
PCP024861	6,807354922	6,392317423	6,426264755	6,454011343	PCP024861	XM_008395291
PCP024891	5,874551087	5,77267747	4,890933022	4,807354922	PCP024891	XM_009338346
PCP024913	6,599912842	6,375039431	6,363346321	5,914803677	PCP024913	XM_018651107
PCP024941	4,584962501	4,790250739	6,327866971	5,235344128	PCP024941	XM_009349093
PCP024944	3,624100895	4,196134881	6,297741678	4,857980995	PCP024944	XM_009349094
PCP024946	4,584962501	4,272769732	6,686500527	5,392317423	PCP024946	XM_009349094
PCP024988	6,254178286	6,523561956	6,4374608	7,30679083	PCP024988	XM_009381176
PCP024999	6,279285561	5,781359714	5,969703088	5,48123435	PCP024999	XM_009381161
PCP025010	5,073391816	4,890933022	4,874305166	3,906890596	PCP025010	XM_009343931
PCP025011	7,761551232	6,894817763	7,033423002	5,746043983	PCP025011	XM_009343926
PCP025032	6,03716255	5,333781501	5,156639311	5,087462841		
PCP025114	6,363346321	6,759289016	6,4374608	6,051589621	PCP025114	XM_009361766
PCP025126	5,815831566	4,459431619	4,029452886	4,662775172		
PCP025143	5,333781501	5,653346693	5,906890596	6,297741678	PCP025143	XM_009379430
PCP025195	6,554588852	6,672425342	6,414981143	7,156538193	PCP025195	XM_008365465
PCP025209	5,129283017	5,64385619	4,772413555	7,014913158	PCP025209	XM_009365748
PCP025229	5,832890014	6,03716255	6,431790083	6,882643049		
PCP025231	6,718772592	6,68187088	6,639087423	6,327866971	PCP025231	XM_008339645
PCP025287	6,094447222	6,564835417	3,221877081	6,454011343	PCP025287	XM_009336213
PCP025307	6,442943496	6,523561956	6,677085351	7,098032083	PCP025307	XM_009335984
PCP025314	6,574858391	5,763677142	5,691254979	5,437294331	PCP025314	XM_009357996
PCP025316	4,772413555	4,247927513	5,285402219	5,624393382	PCP025316	XM_009357990
PCP025339	6,196331634	6,108524457	6,080444483	5,554588852	PCP025339	XM_009339004
PCP025347	4,95419631	5,681730355	5,653346693	6,029673735	PCP025347	XM_021977152
PCP025353	6,862079387	6,619559738	6,333960351	6,828707735		
PCP025388	4,906890596	4,564987801	5,77267747	5,781359714	PCP025388	XM_008367825
PCP025433	5,781359714	5,415150205	5,544423562	4,772413555	PCP025433	XM_009350589
PCP025450	6,189824559	6,403778984	6,291493053	5,222263604	PCP025450	XM_018651739
PCP025458	6,31596467	6,491853096	6,832890014	7,139551352		
PCP025481	5,653346693	5,101397952	5,196528361	4,480911346	PCP025481	XM_009351220
PCP025511	6,559644763	6,07317756	6,906890596	6,309794644	PCP025511	XM_009359922
PCP025559	4,790250739	5	4,985044962	5,938521046	PCP025559	XM_009345513
PCP025583	4,824258697	5,129283017	5,653346693	4,95419631		
PCP025596	6,06608919	5,491853096	5,196528361	4,64385619		
PCP025645	7,212666605	6,890811455	6,754887502	6,254178286		
PCP025650	6,981053471	6,569855608	6,828707735	6,677085351	PCP025650	XR_001953390
PCP025683	7,115511897	6,634157606	6,554588852	6,297741678	PCP025683	XM_009378762
PCP025709	5	4,321928095	6,824386003	5,709566354		
PCP025732	3,969933275	4,196134881	4,64385619	5,345893086	PCP025732	XM_008350125
PCP025748	5,746043983	6,122465658	5,380937195	5,142821844	PCP025748	XM_009336035
PCP025755	6,807354922	7,348728154	6,648609245	5,718909554	PCP025755	XM_009336113
PCP025769	5,196528361	4,321928095	4,807354922	5,502394256	PCP025769	XM_009336289

PCP025788	6,977279923	6,798698597	6,481072857	7,14974712	PCP025788	XM_009336525
PCP025808	5,380937195	5,584962501	5,209453366	4,564987801	PCP025808	XM_008352268
PCP025817	5,345893086	5,984817174	5,196528361	8,241601875	PCP025817	XM_009336937
PCP025818	5,634302842	5,058749412	4,459431619	4,874305166	PCP025818	XM_009359037
PCP025827	6,143026004	6,094447222	6,448405435	5,930737338	PCP025827	XM_009380488
PCP025880	4,700439718	5,309976492	5,544423562	5,890689878	PCP025880	XM_009338673
PCP025885	6,727920455	5,984817174	6,523561956	6,845490051	PCP025885	XM_009338677
PCP025891	6,589913261	5,754887502	5,961854808	5,64385619	PCP025891	XM_008388784
PCP025899	5,169925001	6	5,77267747	6,266786541	PCP025899	XM_008390302
PCP025928	3,459431619	4,564987801	6,176522247	7,965784285	PCP025928	XM_009375009
PCP025929	6	5,309976492	4,196134881	3,624100895	PCP025929	XM_009375010
PCP025973	6,50779464	6,497133304	6,790120385	7,315874125	PCP025973	XM_009358145
PCP026011	5,60496087	4,718635616	5,073391816	4,414812061	PCP026011	XM_009371442
PCP026019	4,272769732	4,64385619	5,044394119	7,011227255		
PCP026021	6,094447222	6,363346321	6,745909573	7,003714662	PCP026021	XM_018650301
PCP026023	6,143026004	6,634157606	6,579994693	6,853870927	PCP026023	XM_009371253
PCP026024	6,914923239	6,64385619	6,554588852	6,363346321		
PCP026039	5,014801602	4,857980995	4,544114402	4,169925001	PCP026039	XM_009354716
PCP026057	6,426264755	6,732404887	6,442943496	6,375039431	PCP026057	XM_009354743
PCP026058	6,798698597	6,363346321	6,442943496	6,022367813	PCP026058	XM_018646733
PCP026077	6,333960351	6,653490009	6,584962501	7,655852677	PCP026077	XM_018643387
PCP026085	6,634157606	5,183089461	5,369117459	4		
PCP026102	6,564835417	6,303780748	6,397974049	6,254178286	PCP026102	XM_009339932
PCP026125	4,682011391	5,101397952	5,087462841	6,156437068		
PCP026143	5,380937195	5,273142859	6,044394119	7,044394119	PCP026143	XM_008341722
PCP026166	5,48123435	4,938756261	5,57500972	6,203005338	PCP026166	XM_009354437
PCP026197	2,414135533	5,345893086	4,196134881	6,619559738	PCP026197	XM_018649522
PCP026207	5,709566354	5,57500972	5,882643049	5,058749412	PCP026207	XM_008381425
PCP026210	6,279285561	6,714245518	5,992541859	5,781359714	PCP026210	XM_008339944
PCP026243	5,718909554	6,129283017	6,454011343	6,750472519		
PCP026256	6,977279923	6,380764075	6,781359714	7,139551352	PCP026256	XM_009363240
PCP026267	5,209453366	6,554588852	5,807354922	6,475733431	PCP026267	XM_009364529
PCP026269	5,77267747	6,357552005	6,279285561	6,946379968	PCP026269	XM_009364449
PCP026279	5,101397952	5,614709844	6,087462841	5,890689878	PCP026279	XM_009364437
PCP026280	4,772413555	5,321928095	5,898933872	7,61227877	PCP026280	XM_009364436
PCP026309	6,763809907	5,898933872	5,663059924	5,672425342	PCP026309	XM_017327419
PCP026310	6,345715709	5,882643049	5,841218374	5,426264755	PCP026310	XM_009345051
PCP026338	5,874551087	5,95419631	6,4374608	7,153095972	PCP026338	XM_018645161
PCP026349	5,285402219	4,459431619	6	5,523561956	PCP026349	XM_018643037
PCP026350	5,437294331	6,599912842	5,815831566	4,321928095	PCP026350	XM_018643038
PCP026353	6,363346321	6,4374608	5,95419631	5,709566354		
PCP026354	5,087462841	5,333781501	5,247927513	4,437627248		
PCP026394	6,958030641	6,589913261	6,101608059	5,653346693		
PCP026423	6,022367813	5,533874777	6,279285561	5,807354922	PCP026423	XM_009360773
PCP026457	5,922911406	5,533874777	6,215872703	5,614709844	PCP026457	XM_018645334
PCP026486	7,047996356	6,464831606	6,523561956	6,523561956	PCP026486	XM_009343584

PCP026489	6,47037417	5,882643049	6,31596467	5,709566354	PCP026489	XM_008350455
PCP026507	5,824513297	5,824513297	6,279285561	6,84962403		
PCP026576	5,115615931	5,380937195	5,624393382	4,624685811	PCP026576	XM_009354031
PCP026588	4,73714592	4,700439718	5,369117459	6,087462841	PCP026588	XM_009356482
PCP026592	5,874551087	5,653346693	5,95419631	6,539158811		
PCP026615	6,4374608	6,614709844	6,667750232	6,163297449	PCP026615	XM_009362266
PCP026619	6,272956308	5,64385619	5,415150205	5,564683017	PCP026619	XM_009362361
PCP026636	5,718909554	5,235344128	5,196528361	3,906890596	PCP026636	XM_009362304
PCP026647	6,015024705	6,31596467	6,584962501	6,832890014		
PCP026661	6,291493053	6,624539604	6,902917719	7,342785837	PCP026661	XM_009379097
PCP026667	5,101397952	4,857980995	4,922673593	4,029452886	PCP026667	XM_018652374
PCP026683	5,554588852	4,73714592	5,459431619	4,874305166	PCP026683	XM_018646286
PCP026705	7,073284692	8,39373366	6,044394119	2,503348735	PCP026705	XM_009362336
PCP026706	6,351734323	6,357552005	7,087462841	6,922792504	PCP026706	XM_009362339
PCP026718	6,564835417	6,922792504	6,403778984	5,614709844		
PCP026751	5,746043983	6,087462841	6,163297449	6,614709844	PCP026751	XM_018644119
PCP026785	6,339850003	6,475733431	6,321928095	7,062531903		
PCP026797	6,24164954	6,599912842	6,667750232	5,754887502	PCP026797	XM_009337676
PCP026800	4,807354922	5,403608584	5,64385619	5,914803677		
PCP026830	5,798828178	6,321928095	6,203005338	6,695854658	PCP026830	XM_009347727
PCP026838	4,772413555	5,815831566	6,115407855	7,377904593	PCP026838	XM_018648269
PCP026863	6,732404887	6,321928095	6,22881869	6,247927513	PCP026863	XM_008349391
PCP026873	6,122465658	5,946496941	5,890689878	5,101397952		
PCP026912	6,62935662	6,058965822	6,4374608	6,215872703	PCP026912	XM_008339120
PCP026914	6,54951516	6,534030467	5,874551087	5,285402219	PCP026914	NM_001328716
PCP026971	4,059182199	4,222650022	4,34553831	5,523561956	PCP026971	NM_001293901
PCP027006	5,235344128	5,700439718	5,922911406	6,297741678		
PCP027012	5,101397952	5,247927513	5	4,222650022		
PCP027041	6,015024705	5,922911406	5,426264755	5,357552005	PCP027041	XM_009344278
PCP027070	5,470211457	4,772413555	4,841469999	4,143230135	PCP027070	XM_018648044
PCP027071	5,235344128	4,369466484	4,985044962	4,321928095		
PCP027077	3,368768349	4,890933022	5,824513297	6	PCP027077	XM_009347181
PCP027117	6,99242856	6,727920455	6,732404887	6,528727582	PCP027117	XR_001952841
PCP027119	6,634157606	6,60481265	6,333960351	7,254272787	PCP027119	XM_008365756
PCP027122	6,886672074	6,741466986	6,50779464	6,051589621	PCP027122	XM_017335132
PCP027132	7,125878364	5,564683017	5,691254979	4,564987801	PCP027132	XM_009340127
PCP027134	5,789990019	5,77267747	5,584962501	5,297558281	PCP027134	XM_018648770
PCP027142	7,094552786	6,741466986	6,97349648	6,594996337	PCP027142	XM_009346734
PCP027154	4,297925053	4,059182199	4,059182199	5,992541859	PCP027154	XM_008387978
PCP027159	6,426264755	6,737010678	7,026025399	7,062531903	PCP027159	XM_021953946
PCP027162	7,412188747	6,667750232	6,709704193	6,03716255	PCP027162	XM_009372465
PCP027194	5,841218374	5,014801602	5,426264755	4,564987801	PCP027194	XM_009372532
PCP027198	6,80309785	6,781359714	6,4374608	6,491853096	PCP027198	XM_017327449
PCP027273	5,554588852	5,48123435	5,02989455	4,857980995	PCP027273	XM_008395330
PCP027303	6,574858391	6,022367813	5,709566354	5,459431619	PCP027303	XM_018649336
PCP027331	6,235152624	5,781359714	6,044394119	6,62935662	PCP027331	XM_009346819

PCP027342	6,934634441	6,906890596	6,853870927	6,691115365	PCP027342	XM_009373107
PCP027358	5,984817174	5,922911406	5,890689878	5,222263604	PCP027358	XM_008372155
PCP027363	5,914803677	5,984817174	5,930737338	6,768184325	PCP027363	XM_018643335
PCP027430	5,653346693	5,392317423	5,798828178	6,486553493	PCP027430	XM_009349692
PCP027431	6,874428132	6,345715709	6,115407855	5,77267747	PCP027431	XM_009349687
PCP027432	3,807354922	4,059182199	4,807354922	5,77267747		
PCP027449	6,222456826	6,094447222	6,235152624	8,403736386	PCP027449	XM_009370435
PCP027458	6,431790083	6,442943496	6,4374608	7,040782866		
PCP027459	4,938756261	5,653346693	5,415150205	6,108524457	PCP027459	XM_021970599
PCP027475	6,60481265	6,351734323	6,528727582	5,984817174	PCP027475	XM_009370400
PCP027492	5,169925001	5,533874777	5,57500972	4,906890596	PCP027492	XM_009342176
PCP027505	4,906890596	6,07317756	6,554588852	4,772413555	PCP027505	XM_008354153
PCP027549	5,857980995	5,470211457	5,938521046	4,321928095	PCP027549	XM_009381253
PCP027553	5,691254979	4,874305166	5,297558281	5,058749412	PCP027553	XM_009381258
PCP027572	5,129283017	5,502394256	5,357552005	6,14974712		
PCP027590	6,409390936	6,619559738	6,297741678	5,235344128	PCP027590	XM_018645988
PCP027603	5,807354922	5,02989455	5,64385619	6,926829678		
PCP027610	5,977279923	6,14974712	6,448405435	6,732404887	PCP027610	XM_009370616
PCP027611	6,266786541	6	6,321928095	6,781359714	PCP027611	XM_009370621
PCP027619	7,369204723	6,882643049	6,768184325	6,392317423	PCP027619	XM_009355887
PCP027634	6,409390936	6,60481265	6,594996337	7,076815597	PCP027634	XM_009356212
PCP027635	4,874305166	4,321928095	6,497133304	4,624685811	PCP027635	XM_009340666
PCP027683	5,754887502	5,882643049	6,051589621	6,594996337	PCP027683	XM_008344076
PCP027684	5,709566354	5,169925001	5,914803677	6,667750232	PCP027684	XM_008364106
PCP027703	3,544732656	4,437627248	5,169925001	4,938756261		
PCP027715	5,938521046	5,448570626	5,584962501	4,624685811	PCP027715	XM_008390949
PCP027731	4,906890596	5,247927513	5,533874777	6,169925001		
PCP027737	5,727920455	5,564683017	5,681730355	5,142821844		
PCP027745	5,48123435	5,691254979	6,044394119	6,569855608	PCP027745	XM_018642889
PCP027746	6,737010678	5,709566354	6,64385619	6,714245518		
PCP027769	7,033423002	6,634157606	5,798828178	5,857980995		
PCP027840	5,309976492	6,426264755	5,260402093	5,448570626		
PCP027844	6,672425342	5,709566354	6,464831606	0		
PCP027849	2,873813198	6,853870927	7,822284016	6,832890014	PCP027849	XM_009358741
PCP027860	6,569855608	6,709704193	6,737010678	6,14974712		
PCP027900	5,115615931	4,682011391	4,502712486	4,059182199		
PCP027904	6,528727582	6,569855608	6,101608059	5,798828178		
PCP027928	5,841218374	6,392317423	6,662917555	6,824386003	PCP027928	XM_009357712
PCP027953	5,898933872	5,459431619	5,426264755	4,272769732	PCP027953	XM_017325021
PCP027958	7,011227255	6,714245518	6,988684687	6,235152624	PCP027958	XM_009336260
PCP027971	6,115407855	6,260590275	6,392317423	6,882643049		
PCP028004	4,544114402	4,874305166	5,115615931	3,700439718	PCP028004	XM_009356223
PCP028014	5,297558281	5,584962501	5,614709844	6,345715709	PCP028014	XM_009347932
PCP028021	5,857980995	5,922911406	6,534030467	6,837060204	PCP028021	XM_008354988
PCP028024	6,122465658	5,824513297	5,914803677	6,918863237	PCP028024	NM_001328731
PCP028129	5,700439718	5,345893086	5,824513297	6,614709844		

PCP028130	5,554588852	5,564683017	5,898933872	6,534030467	PCP028130	XM_009354277
PCP028162	6,95419631	7,136170875	5,77267747	5,544423562	PCP028162	XM_009361399
PCP028169	6,375039431	6,554588852	6,969587981	6,50779464	PCP028169	XM_009358300
PCP028187	4,169925001	3,906890596	3,969933275	6,163297449	PCP028187	NM_001328736
PCP028200	6,189824559	6,397974049	6,790120385	7,667679281	PCP028200	XM_009341787
PCP028218	6,648609245	6,080444483	5,938521046	5,807354922	PCP028218	XM_008352641
PCP028225	6,333960351	6,03716255	5,866166169	5,345893086	PCP028225	XM_009339443
PCP028248	5,938521046	6,080444483	6,222456826	6,965784285	PCP028248	XM_009356953
PCP028254	6,853870927	6,64385619	7,040782866	6,68187088	PCP028254	XM_009353518
PCP028262	6,705010253	6,339850003	6,513016928	6,247927513	PCP028262	XM_009372968
PCP028291	3,938285792	3,874796966	4,73714592	5,502394256	PCP028291	XM_009359545
PCP028378	5,073391816	4,841469999	4,544114402	3,906890596	PCP028378	XM_009343931
PCP028392	5,700439718	5,727920455	5,857980995	5,014801602	PCP028392	XM_009363176
PCP028413	4,754887502	3,840966704	6,68187088	5,448570626	PCP028413	XM_008392377
PCP028471	6,559644763	5,906890596	6,029673735	4,718635616	PCP028471	XM_008378350
PCP028494	6,156437068	6,491853096	6,977279923	7,351645995	PCP028494	XM_009378237
PCP028532	5,564683017	5,222263604	6,369291982	6,285402219	PCP028532	XM_017322527
PCP028603	6,209453366	6,339850003	6,015024705	5,653346693	PCP028603	XM_009371579
PCP028619	5,273142859	4,874305166	5,142821844	4,392317423	PCP028619	XM_009380536
PCP028623	6,686500527	6,589913261	6,189824559	5,922911406		
PCP028636	5,700439718	5,437294331	5,709566354	5,142821844	PCP028636	XM_008343569
PCP028684	6,321928095	6,574858391	6,639087423	7,30679083	PCP028684	XM_009352977
PCP028759	5,815831566	5,459431619	5,273142859	4,392317423	PCP028759	XM_008384005
PCP028795	5,653346693	6,058965822	6,47037417	6,481072857	PCP028795	XM_008393589
PCP028844	6,143026004	5,866166169	5,533874777	5,058749412	PCP028844	XM_009373799
PCP028848	5,502394256	5,653346693	6,662917555	7,62935662	PCP028848	XM_009373806
PCP028877	6,357552005	6,14974712	6,820178962	6,705010253	PCP028877	XM_008362033
PCP028891	6,965784285	6,824386003	6,902917719	6,279285561	PCP028891	XM_008382452
PCP028892	6,392317423	5,882643049	5,914803677	5,273142859	PCP028892	XM_008382454
PCP028912	5,754887502	5,196528361	5,260402093	4,480911346	PCP028912	XM_009341624
PCP028923	5,624393382	5,866166169	5,523561956	4,502712486	PCP028923	XM_009372873
PCP028945	5,815831566	5,101397952	5,02989455	4,772413555	PCP028945	XM_008343943
PCP028975	6,169925001	6,81159947	6,686500527	6,820178962	PCP028975	XM_009337084
PCP028985	5,309976492	6,196331634	6,339850003	6,392317423		
PCP029003	6,215872703	6,260590275	6,695854658	7,247927513	PCP029003	XM_009335758
PCP029009	5,746043983	4,087462841	5,044394119	3,663344619	PCP029009	XM_009335766
PCP029075	6,122465658	5,614709844	5,073391816	4,414812061	PCP029075	XM_018649495
PCP029100	6,523561956	6,303780748	6,50255338	7,497213158		
PCP029134	5,533874777	5,448570626	4,73714592	4,624685811	PCP029134	XM_018645093
PCP029160	4,321928095	5,058749412	6,95419631	8,560982378	PCP029160	XM_009354570
PCP029169	5,763677142	5,584962501	5,938521046	5,129283017	PCP029169	XM_009354584
PCP029194	5,426264755	5,309976492	5,824513297	5,156639311	PCP029194	XM_008380831
PCP029229	6,99242856	7,007531912	6,815959618	5,718909554	PCP029229	XM_009366901
PCP029247	6,559644763	6,634157606	6,737010678	5,95419631	PCP029247	XM_009366926
PCP029261	6,64385619	7,087462841	6,695854658	6,309794644	PCP029261	XM_008346095
PCP029293	4,718635616	5,196528361	4,857980995	5,763677142	PCP029293	XM_008241565

PCP029328	4,459431619	4,807354922	4,523561956	5,727920455	PCP029328	XM_009352603
PCP029358	5,309976492	4,718635616	4,922673593	4,502712486		
PCP029384	5,426264755	5,681730355	5,60496087	6,333960351		
PCP029386	6,272956308	5,285402219	5,058749412	3,906890596	PCP029386	XM_009336947
PCP029391	6,094447222	6,196331634	6,094447222	5,789990019	PCP029391	XM_017329960
PCP029399	5,798828178	5,906890596	6,31596467	6,84962403	PCP029399	XM_017322698
PCP029401	6,714245518	6,86628983	6,727920455	6,589913261	PCP029401	XM_018649072
PCP029429	5,285402219	6,614709844	5,426264755	5,101397952	PCP029429	XM_008382563
PCP029466	5	5,448570626	5,357552005	6,481072857	PCP029466	XM_008357809
PCP029517	6,523561956	6,569855608	6,981053471	7,263691734	PCP029517	XM_018644889
PCP029563	6,38663853	6,22881869	6,564835417	7,018700931	PCP029563	XM_009362973
PCP029565	6,101608059	5,866166169	5,824513297	5,297558281	PCP029565	XM_009362975
PCP029566	6,169925001	6,397974049	6,539158811	7,215969746	PCP029566	XM_009362977
PCP029582	5,260402093	4,841469999	5,115615931	3,700439718	PCP029582	XM_009358960
PCP029633	4,874305166	5,60496087	4,624685811	4,502712486		
PCP029649	5,491853096	5,380937195	4,115199749	5,357552005	PCP029649	XM_008339594
PCP029665	5,624393382	6,094447222	6,22881869	6,414981143	PCP029665	XM_009353536
PCP029669	6,47037417	6,203005338	5,992541859	5,930737338	PCP029669	XM_008378821
PCP029685	6,459431619	5,946496941	6,403778984	5,614709844	PCP029685	XR_001951494
PCP029690	7,251056285	6,554588852	5,64385619	5	PCP029690	XM_009380229
PCP029700	6,403778984	6,700439718	6,705010253	7,108524457		
PCP029741	4,272769732	5,073391816	5,64385619	6,38663853		
PCP029744	7,21916852	6,938638658	6,902917719	6,209453366	PCP029744	XM_018648044
PCP029772	6,709704193	6,785681319	6,80309785	6,426264755	PCP029772	XM_008366146
PCP029776	5,672425342	6,297741678	6,136068312	6,534030467	PCP029776	XM_009368519
PCP029800	3,736604875	4,247927513	5,459431619	5,48123435		
PCP029837	6,946379968	6,781359714	7,315874125	6,426264755		
PCP029857	5,209453366	4,73714592	4,906890596	4,087462841	PCP029857	XM_009376472
PCP029872	5,992541859	5,874551087	6,658211483	7,539158811	PCP029872	XM_009378739
PCP029896	6,222456826	5,691254979	5,746043983	5,709566354	PCP029896	XM_009342268
PCP029904	6,732404887	6,375039431	6,653490009	7,080337882	PCP029904	XM_009341360
PCP029931	5,727920455	5,544423562	5,459431619	4,985044962	PCP029931	XM_009344495
PCP029938	6,291493053	6,700439718	6,392317423	5,781359714	PCP029938	XM_009379212
PCP029965	4,459431619	4,922673593	4,790250739	5,984817174	PCP029965	XM_009348239
PCP029978	7,029784146	7,17990909	7,022367813	6,260590275	PCP029978	XM_008220894
PCP029990	6,86628983	6,584962501	6,291493053	6,996275749	PCP029990	XM_009358449
PCP030001	6,86628983	6,946379968	7,090959258	6,569855608	PCP030001	XM_018644084
PCP030043	5,992541859	5,624393382	6,087462841	6,634157606	PCP030043	XM_009348228
PCP030052	6,247927513	5,914803677	6,667750232	5,95419631	PCP030052	XM_009362735
PCP030056	6,303780748	6,007419784	5,95419631	5,183089461	PCP030056	XM_018650290
PCP030096	7,037052702	6,841344192	6,938638658	6,60481265	PCP030096	XM_009360068
PCP030120	6,574858391	6,272956308	5,992541859	5,95419631	PCP030120	XM_008370788
PCP030177	4,985044962	5,222263604	4,700439718	4,247927513	PCP030177	XM_008379374
PCP030182	4,544114402	4,662775172	5,235344128	5,824513297	PCP030182	XM_008376147
PCP030216	7,297649983	7,386552314	6,781359714	5,634302842	PCP030216	XM_009361438
PCP030285	6,285402219	5,992541859	6,022367813	5,59484709	PCP030285	XM_017324555

PCP030296	5,297558281	4,857980995	4,969472865	4,196134881	PCP030296	XM_009350589
PCP030306	5,533874777	5,48123435	5,403608584	6,209453366		
PCP030309	7,193081765	6,961970533	6,807354922	6,523561956	PCP030309	XM_018643903
PCP030315	7,375039431	7,238404739	7,222360218	5,554588852	PCP030315	XR_526171
PCP030343	6,534030467	5,754887502	5,333781501	5,718909554	PCP030343	NM_001302317
PCP030352	5,95419631	5,653346693	6,741466986	7,108524457		
PCP030424	6,279285561	5,807354922	5,273142859	4	PCP030424	XM_018642848
PCP030426	3,840966704	4,523561956	5,57500972	5,044394119	PCP030426	XM_008381068
PCP030445	4,143230135	4,824258697	5,95419631	4,906890596	PCP030445	XM_008359525
PCP030478	4,938756261	4,841469999	5,073391816	4	PCP030478	XM_018648399
PCP030533	6,700439718	6,653490009	6,122465658	6,007419784	PCP030533	XM_009342772
PCP030543	4,584962501	4,754887502	5,48123435	5,849749117		
PCP030558	6,094447222	5,763677142	5,841218374	5,59484709	PCP030558	XM_008391587
PCP030566	6,044394119	5,969703088	6,569855608	5,48123435	PCP030566	XM_008391598
PCP030583	5,014801602	5,691254979	5,64385619	6,266786541	PCP030583	XM_009341752
PCP030611	4,841469999	5,415150205	5,321928095	6,007419784	PCP030611	XM_009373678
PCP030646	6,094447222	5,874551087	5,709566354	5,513174885	PCP030646	XM_008359342
PCP030692	6	6,108524457	6,95419631	7,456724026	PCP030692	XM_008379132
PCP030702	5,77267747	6,392317423	7,798763389	6,950351762	PCP030702	XM_009343466
PCP030773	5,634302842	4,662775172	4,115199749	4,502712486	PCP030773	XM_021961751
PCP030787	5,196528361	6,087462841	6,491853096	7,222360218		
PCP030834	6,54951516	6,882643049	6,534030467	7,115511897	PCP030834	XM_009339276
PCP030841	7,783522135	6,890811455	6,965784285	5,60496087		
PCP030851	4,143230135	4,272769732	5,222263604	6,015024705		
PCP030922	6,658211483	6,741466986	6,333960351	6,247927513	PCP030922	XM_009343135
PCP030928	4,414812061	4,807354922	4,247927513	5,60496087	PCP030928	XM_009376990
PCP030934	6,72342204	6,672425342	7,122362117	6,403778984	PCP030934	XM_009347676
PCP030940	6,169925001	6,14974712	6,303780748	7,062531903	PCP030940	XM_009347662
PCP030946	5,285402219	5,073391816	4,64385619	3,502075956	PCP030946	XM_018643832
PCP030960	6,86628983	7,189824559	6,926829678	6		
PCP030972	6,667750232	6,750472519	6,375039431	6,291493053	PCP030972	XM_009365299
PCP030977	5,415150205	5,415150205	6,094447222	5,321928095		
PCP031009	4,604664415	5,345893086	6,108524457	5,309976492	PCP031009	XM_009342145
PCP031012	4,857980995	6,183288001	5,415150205	5,59484709	PCP031012	XM_008381628
PCP031024	6,115407855	6,481072857	6,029673735	4,718635616	PCP031024	XM_018649677
PCP031089	6,80309785	6,420718183	6,584962501	6,363346321	PCP031089	XM_009364599
PCP031103	5,849749117	5,624393382	5,60496087	4,544114402		
PCP031128	5,247927513	5,392317423	5,087462841	6,303780748	PCP031128	XM_009362243
PCP031140	5,977279923	5,961854808	5,841218374	6,569855608	PCP031140	XM_009342280
PCP031174	6,619559738	6,569855608	6,087462841	6,926829678	PCP031174	XM_009343476
PCP031181	6,584962501	6,513016928	6,481072857	6,14974712	PCP031181	XM_008394865
PCP031188	5,297558281	4,34553831	5,02989455	7,759355602	PCP031188	XM_008369315
PCP031213	7,244791942	6,426264755	6,058965822	6,614709844	PCP031213	XM_018649170
PCP031219	7,14974712	7,08395793	6,828707735	6,403778984	PCP031219	XM_018643657
PCP031225	6,007419784	6,599912842	6,475733431	5,984817174	PCP031225	XM_009336628
PCP031257	5,247927513	5,369117459	4,938756261	3,874796966	PCP031257	XM_009339136

PCP031270	5,087462841	4,922673593	4,857980995	3,807354922	PCP031270	XM_018649613
PCP031284	5,058749412	4,73714592	4,874305166	4	PCP031284	XM_008361802
PCP031293	5,763677142	6,169925001	5,961854808	6,709704193	PCP031293	XM_009368278
PCP031298	6,115407855	6,272956308	6,403778984	6,906890596	PCP031298	XM_009341375
PCP031310	6,47037417	6,339850003	6,47037417	6,058965822	PCP031310	XM_009339253
PCP031376	6,750472519	6,426264755	6,741466986	6,448405435		
PCP031379	5,369117459	4,938756261	4,718635616	4,480911346	PCP031379	XM_009375423
PCP031412	6,380764075	6,672425342	6,60481265	5,789990019	PCP031412	XM_018645425
PCP031467	6,183288001	6,47037417	6,672425342	6,906890596	PCP031467	XM_009369509
PCP031506	6,380764075	6	5,882643049	5,309976492	PCP031506	XM_009345665
PCP031508	6,662917555	5,824513297	6,523561956	6,087462841	PCP031508	XM_009363474
PCP031545	4,906890596	4,890933022	5,392317423	5,857980995		
PCP031557	5,087462841	5,59484709	5,129283017	4,414812061	PCP031557	XM_018645759
PCP031640	6,584962501	6,718772592	6,781359714	7,247927513	PCP031640	XM_009363709
PCP031656	6,303780748	6,750472519	6,431790083	7,044394119		
PCP031694	6,910852562	6,926829678	6,695854658	6,513016928	PCP031694	XM_009368583
PCP031725	4,115199749	4,564987801	4,682011391	5,60496087	PCP031725	XM_009349846
PCP031729	6,183288001	6,279285561	6,584962501	6,169925001	PCP031729	XM_018649110
PCP031774	3,544732656	4,321928095	5,321928095	8		
PCP031923	6,564835417	6,60481265	6,794415866	7,273049587	PCP031923	XM_009359651
PCP031955	6,820178962	6,634157606	7,285402219	6,574858391	PCP031955	XM_009370949
PCP032009	4,604664415	4,544114402	5,437294331	6,209453366		
PCP032081	6,4374608	5,718909554	6,392317423	5,824513297		
PCP032149	6,392317423	6,594996337	6,634157606	6,303780748		
PCP032156	5,014801602	4,143230135	4,584962501	5,345893086	PCP032156	XM_018647921
PCP032158	6,486553493	6,54951516	6,60481265	7,263691734	PCP032158	XM_009357478
PCP032163	5,754887502	6,094447222	6,222456826	6,584962501		
PCP032181	6,718772592	6,345715709	6,24164954	5,763677142	PCP032181	XM_009348093
PCP032198	6,824386003	5,938521046	6,737010678	5,746043983	PCP032198	XM_009373582
PCP032222	7,199672345	7,231989133	6,695854658	6,087462841	PCP032222	XM_009376111
PCP032223	4,222650022	4,459431619	7,06608919	5,64385619	PCP032223	XM_009376110
PCP032241	5,309976492	5,115615931	5,832890014	6,777025123	PCP032241	XM_009365931
PCP032244	5,930737338	6,24164954	6,64385619	6,64385619	PCP032244	XM_009368854
PCP032254	6,624539604	6,481072857	6,203005338	5,746043983	PCP032254	XM_008368262
PCP032282	5,727920455	5,984817174	6,266786541	6,906890596	PCP032282	XM_008359161
PCP032339	7,029784146	6,714245518	6,291493053	5,946496941	PCP032339	XM_018643374
PCP032440	4,604664415	5,309976492	6,254178286	7,173227395	PCP032440	NM_001294010
PCP032497	6,882643049	6,60481265	6,176522247	5,709566354	PCP032497	XM_008376187
PCP032535	7,132679654	6,882643049	6,554588852	5,681730355	PCP032535	XM_008356190
PCP032561	5,754887502	5,087462841	4,115199749	6,080444483	PCP032561	XM_009337834
PCP032573	6,247927513	6,448405435	6,599912842	7,022367813	PCP032573	XM_009359521
PCP032619	6,215872703	7,058857621	6,745909573	6,497133304	PCP032619	XM_009363901
PCP032628	6,357552005	6,454011343	6,163297449	6,129283017		
PCP032652	3,840966704	4,790250739	5,459431619	6,539158811	PCP032652	XR_525016
PCP032702	6,732404887	6,745909573	6,691115365	6,448405435	PCP032702	XM_008390587
PCP032785	7,044394119	6,700439718	6,981053471	6,64385619	PCP032785	XR_667498

PCP032796	4,824258697	5,523561956	5,369117459	6,375039431		
PCP032837	5,129283017	5,882643049	6,24164954	7,101503009	PCP032837	XM_009380628
PCP032952	6,554588852	6,189824559	6,431790083	5,807354922	PCP032952	XM_009352396
PCP033062	5,369117459	4,938756261	5,502394256	4,502712486	PCP033062	XM_018649575
PCP033105	5,60496087	5,59484709	5,77267747	4,985044962	PCP033105	XM_009353287
PCP033108	6,709704193	7,101503009	6,882643049	6,714245518		
PCP033136	6,648609245	6,222456826	6,129283017	6,06608919		
PCP033141	6,759289016	6,351734323	6,72342204	7,279192684	PCP033141	XM_017333118
PCP033156	6,007419784	5,663059924	5,681730355	6,481072857		
PCP033165	4,321928095	5,044394119	5,614709844	3,502075956	PCP033165	XM_009344361
PCP033199	4,790250739	6,327866971	5,95419631	5,807354922	PCP033199	XM_018650071
PCP033218	6,143026004	5,977279923	6,464831606	6,95419631		
PCP033244	5,48123435	5,977279923	6,309794644	6,841344192	PCP033244	XM_018650720
PCP033300	6,06608919	6,491853096	6,38663853	5,882643049	PCP033300	XM_009338119
PCP033326	6,80309785	6,448405435	7,212666605	6,559644763	PCP033326	XM_018651603
PCP033329	6,375039431	5,584962501	5,260402093	5,142821844	PCP033329	XM_018645168
PCP033400	6,392317423	6,459431619	6,890811455	7,142923928	PCP033400	XM_009380848
PCP033442	6,47037417	6,209453366	6,50779464	6,898812977	PCP033442	XM_008391655
PCP033563	5,832890014	5,857980995	6,297741678	6,759289016	PCP033563	XM_009375752
PCP033647	5,653346693	5,415150205	5,807354922	6,279285561	PCP033647	XM_009353696
PCP033648	6,38663853	6,176522247	5,946496941	4,969472865	PCP033648	XM_008370695
PCP033726	5,415150205	4,969472865	5,014801602	4,272769732	PCP033726	XM_009339080
PCP033744	4,938756261	5,297558281	5,285402219	6,07317756	PCP033744	XM_009358695
PCP033795	7,497213158	6,727920455	6,84962403	5,984817174	PCP033795	XM_009342436
PCP033816	6,022367813	6,080444483	6,321928095	5,533874777		
PCP033851	6,029673735	5,087462841	6,215872703	5,624393382	PCP033851	XM_009354290
PCP033853	5,984817174	6,022367813	6,272956308	6,894817763	PCP033853	XM_008369695
PCP033916	4,985044962	4,772413555	5,209453366	6,058965822		
PCP034042	5,763677142	5,653346693	5,470211457	5,129283017	PCP034042	XM_017334565
PCP034075	6,189824559	6,196331634	6,686500527	6,272956308	PCP034075	XM_009370543
PCP034119	7,022367813	6,790120385	6,772545519	6,528727582	PCP034119	XM_008341791
PCP034142	6,080444483	5,930737338	6,143026004	6,87036472		
PCP034211	6,24164954	6,754887502	0	6,392317423	PCP034211	XM_008395566
PCP034236	6,579994693	6,584962501	6,574858391	6,285402219	PCP034236	XM_009363511
PCP034254	6,209453366	6,22881869	6,375039431	5,874551087	PCP034254	XR_664451
PCP034318	6,136068312	5,798828178	5,882643049	5,513174885	PCP034318	XM_009347933
PCP034378	6,662917555	6,156437068	6,297741678	6,051589621	PCP034378	XM_009376865
PCP034410	7,183188734	7,21916852	6,815959618	6,235152624	PCP034410	XM_018643343
PCP034420	4,824258697	3,906890596	5,448570626	4,624685811	PCP034420	XM_008389115
PCP034514	4,459431619	4,824258697	4,890933022	5,969703088	PCP034514	XM_009378736
PCP034515	5,101397952	5,297558281	5,64385619	6,50255338	PCP034515	XM_009378556
PCP034521	5,02989455	4,682011391	4,95419631	5,77267747	PCP034521	XM_018643230
PCP034522	6,459431619	6,431790083	6,051589621	5,832890014	PCP034522	XM_009369314
PCP034529	0	8,950322598	10,34132979	0	PCP034529	XM_009381360
PCP034635	5,115615931	5,437294331	6,108524457	6,857980995	PCP034635	XM_017334308
PCP034690	6,321928095	6,824386003	6,68187088	7,369204723	PCP034690	XM_009342001

PCP034759	5,169925001	5,691254979	5,709566354	6,380764075	PCP034759	XM_008341113
PCP034770	5,273142859	5,77267747	5,156639311	4,604664415		
PCP034778	6,31596467	6,333960351	6,54426899	7,055282436		
PCP034796	6,321928095	6,279285561	6,554588852	6,984931073	PCP034796	XM_009344290
PCP034934	7,282347131	0	5,938521046	5,584962501		
PCP035006	7,014913158	7,122362117	6,345715709	5,470211457	PCP035006	XM_018649737
PCP035016	5,369117459	5,663059924	5,321928095	5	PCP035016	XM_008391948
PCP035017	5,392317423	5,169925001	4,969472865	4,564987801	PCP035017	NM_001293872
PCP035035	5,285402219	4,523561956	5,392317423	6,06608919		
PCP035049	6,890811455	6,727920455	7,429030064	6,414981143	PCP035049	XM_008390331
PCP035076	6,297741678	5,857980995	6,50255338	6,99242856	PCP035076	XM_008387941
PCP035077	5,874551087	5,832890014	5,992541859	5,345893086	PCP035077	XM_008387935
PCP035083	5,977279923	6,442943496	5,754887502	6,832890014		
PCP035089	6,754887502	6,339850003	6,464831606	5,437294331		
PCP035104	5,101397952	5,235344128	5,380937195	4,369466484		
PCP035129	4,772413555	4,624685811	4,969472865	5,882643049	PCP035129	XM_008371310
PCP035228	5,554588852	5,345893086	4,985044962	4,459431619	PCP035228	XR_002527554
PCP035261	6,380764075	5,513174885	6,007419784	5,866166169	PCP035261	XM_009371520
PCP035307	6,518377768	5,544423562	5,798828178	5,700439718		
PCP035341	5,653346693	5,392317423	6,50255338	6,614709844		
PCP035515	6,176522247	5,866166169	5,781359714	5,789990019	PCP035515	XM_018651445
PCP035534	4,392317423	4,624685811	4,64385619	6,080444483	PCP035534	XM_009350195
PCP035605	6,169925001	6	7,183188734	7,321928095	PCP035605	XM_009360876
PCP035668	6,31596467	6,47037417	6,68187088	7,087462841		
PCP035673	5,992541859	6,357552005	5,832890014	6,965784285	PCP035673	XM_009343634
PCP035678	7,098032083	6,624539604	6,922792504	6,397974049	PCP035678	XM_008342748
PCP035715	5,584962501	5,906890596	3,058316496	7,526147081		
PCP035740	6,094447222	6,431790083	6,965784285	7,486473046		
PCP035757	7,062531903	6,910852562	6,599912842	6,156437068	PCP035757	XM_009344501
PCP035847	5,763677142	6,015024705	6,303780748	6,68187088	PCP035847	XM_009379265
PCP035853	6,129283017	7,910912508	6,902917719	5,992541859	PCP035853	XM_009336163
PCP035855	6,95419631	6,183288001	6,841344192	5,718909554		
PCP035894	4,392317423	6,807354922	5,115615931	5,209453366	PCP035894	XM_009365630
PCP035897	5,392317423	4,502712486	4,143230135	4,222650022		
PCP035975	6,029673735	6,64385619	6,129283017	5,95419631	PCP035975	XM_009358818
PCP036026	5,691254979	6,918863237	6,209453366	7,536596918	PCP036026	XM_009361494
PCP036027	6,523561956	5,321928095	5,938521046	5,59484709		
PCP036084	6,68187088	6,481072857	5,930737338	5,634302842	PCP036084	XM_018644892
PCP036101	5,691254979	5,222263604	5,309976492	4,564987801	PCP036101	XM_008360760
PCP036104	6,285402219	6,044394119	6,50255338	6,029673735		
PCP036113	6,209453366	5,890689878	5,781359714	5,044394119		
PCP036199	6,380764075	5,653346693	4,841469999	4,222650022		
PCP036246	6,363346321	6,129283017	6	5,309976492	PCP036246	XM_009349165
PCP036314	6,464831606	5,874551087	6,420718183	6,794415866	PCP036314	XM_009350780
PCP036333	5,321928095	5,415150205	5,129283017	6,235152624	PCP036333	XM_009342526
PCP036351	4,807354922	5,073391816	5,209453366	4	PCP036351	XR_001786627

PCP036394	3,700439718	4,414812061	4,662775172	5,415150205	PCP036394	XM_009346019
PCP036429	4,297925053	5,260402093	6,22881869	10,31212284	PCP036429	XM_009352525
PCP036493	6,639087423	6,260590275	6,691115365	7,309885571	PCP036493	XM_009378139
PCP036507	6,222456826	6,115407855	6,215872703	6,95419631	PCP036507	XM_009358782
PCP036553	4,584962501	4,857980995	4,874305166	3,906890596		
PCP036630	6,589913261	6,196331634	6,648609245	7,018700931	PCP036630	XM_009353511
PCP036659	5,815831566	5,718909554	5,849749117	5,247927513	PCP036659	XM_009346249
PCP036665	5,48123435	4,754887502	6,841344192	5,824513297		
PCP036671	5,073391816	5,437294331	5,663059924	6,414981143	PCP036671	XM_009357986
PCP036745	4,297925053	4,874305166	5,209453366	5,48123435	PCP036745	XM_018646342
PCP036775	5,309976492	5,345893086	5,415150205	4,682011391		
PCP036776	5,523561956	4,624685811	4,414812061	4,143230135	PCP036776	XM_009340997
PCP036792	5,209453366	5,890689878	6,333960351	5,523561956	PCP036792	XM_018642698
PCP036927	5,681730355	5,890689878	6,101608059	6,938638658		
PCP036959	5,544423562	5	6,044394119	4,985044962		
PCP037077	4,414812061	4,624685811	5,058749412	5,59484709	PCP037077	XM_008349165
PCP037108	6,136068312	5,992541859	6,03716255	5,491853096		
PCP037161	4,969472865	5,491853096	5,807354922	6,890811455		
PCP037187	6,431790083	5,781359714	6,50779464	7,582480735	PCP037187	XM_009361324
PCP037250	5,653346693	4,754887502	4,906890596	4,459431619	PCP037250	XM_009378762
PCP037280	5,196528361	4,437627248	4,890933022	5,564683017	PCP037280	XM_009340519
PCP037403	4,718635616	4,682011391	5,285402219	5,866166169		
PCP037429	6,297741678	5,209453366	5,426264755	4,564987801	PCP037429	XM_009373848
PCP037461	5,700439718	5,544423562	6,579994693	6,38663853		
PCP037491	5,437294331	4,824258697	5,101397952	4,523561956	PCP037491	XM_009354560
PCP037519	5,700439718	6,07317756	6,333960351	6,934634441	PCP037519	XM_008390524
PCP037579	4,624685811	4,95419631	5,235344128	6,07317756	PCP037579	XM_008379822
PCP037601	6,442943496	5,554588852	6,431790083	6,475733431		
PCP037736	5,502394256	5,48123435	5,763677142	6,47037417		
PCP037739	5,57500972	5,815831566	4,938756261	6,094447222		
PCP037774	4,824258697	5,260402093	4,624685811	3,874796966	PCP037774	XM_017336419
PCP037782	6,922792504	6,534030467	6,745909573	6,14974712	PCP037782	XM_009348980
PCP037826	5,357552005	4,790250739	5,058749412	4,34553831		
PCP037843	6,029673735	6,247927513	5,946496941	5,624393382	PCP037843	XM_009336632
PCP037858	6,853870927	6,513016928	6,518377768	6,24164954	PCP037858	XM_009356574
PCP037875	5,815831566	5,754887502	5,938521046	5,209453366		
PCP037899	5,832890014	5,584962501	5,222263604	4,824258697	PCP037899	XM_008392165
PCP038003	5,984817174	6,176522247	6,403778984	6,750472519	PCP038003	XM_008380489
PCP038054	7,30679083	6,68187088	6,902917719	6,464831606	PCP038054	XM_009345707
PCP038062	5,969703088	6,31596467	6,518377768	7,312882955	PCP038062	XM_009373172
PCP038107	6,569855608	6,235152624	5,992541859	7,014913158		
PCP038130	6,203005338	5,746043983	6,4374608	6,609843592	PCP038130	XM_009363489
PCP038179	6,54426899	6,209453366	6,4374608	5,614709844	PCP038179	XM_009362983
PCP038289	7,076815597	7,051698368	6,910852562	6,403778984		
PCP038330	5,736875423	6,136068312	6,051589621	6,54951516		
PCP038362	6,351734323	6,06608919	6,357552005	6,87036472	PCP038362	XM_009356434

PCP038443	6,497133304	5,890689878	5,832890014	6,122465658		
PCP038541	5,087462841	4,824258697	4,874305166	3,807354922	PCP038541	XM_018642965
PCP038578	6,136068312	6,094447222	6,24164954	5,222263604	PCP038578	XM_008347294
PCP038603	6,279285561	5,938521046	6,209453366	5,48123435	PCP038603	XM_009378940
PCP038644	6,169925001	6,513016928	6,653490009	7,176621973	PCP038644	XR_001953877
PCP038646	0	6,431790083	5,969703088	6,176522247		
PCP038749	6,431790083	6,648609245	6,662917555	7,101503009		
PCP038754	5,906890596	5,59484709	5,59484709	5,247927513	PCP038754	XM_009352741
PCP038815	6,247927513	6,442943496	6,768184325	7,193081765	PCP038815	XM_009339659
PCP038906	6,414981143	6,087462841	6,38663853	7,17990909	PCP038906	XR_001952858
PCP038921	6,351734323	5,77267747	6,272956308	6,574858391	PCP038921	XM_008356679
PCP038971	5,64385619	5,415150205	5,448570626	4,857980995		
PCP038985	5,209453366	4,662775172	4,584962501	4,459431619	PCP038985	XM_009365894
PCP039015	5,196528361	4,392317423	6,614709844	6,351734323	PCP039015	XM_009343871
PCP039029	4,857980995	5,169925001	5,196528361	6,136068312	PCP039029	XM_009369477
PCP039030	6,648609245	6,321928095	6,754887502	6,080444483	PCP039030	XM_009369481
PCP039156	6,06608919	6,169925001	6,285402219	6,97349648	PCP039156	XM_008348513
PCP039180	4,297925053	5	5,058749412	5,691254979		
PCP039220	6,14974712	5,946496941	6,321928095	6,759289016		
PCP039241	5,736875423	6,051589621	5,754887502	7,139551352		
PCP039252	5,614709844	6,260590275	6,108524457	6,481072857		
PCP039326	5,285402219	5,369117459	5,77267747	6,38663853	PCP039326	XM_017325570
PCP039396	6,380764075	6,464831606	6,790120385	7,14974712	PCP039396	XM_018647276
PCP039433	5,196528361	5,309976492	4,682011391	4,321928095	PCP039433	XM_009346343
PCP039472	7,212666605	7,142923928	6,50255338	5,95419631	PCP039472	XM_009377143
PCP039487	5,874551087	6,375039431	6,07317756	5,653346693		
PCP039501	5,709566354	6,07317756	6,266786541	5,672425342	PCP039501	XM_008391488
PCP039516	6,339850003	6,03716255	6,07317756	5,898933872	PCP039516	XM_009356629
PCP039566	6,380764075	6,624539604	6,285402219	6,101608059		
PCP039588	6,285402219	6,732404887	6,832890014	7,215969746	PCP039588	XM_009340611
PCP039591	6,094447222	6,07317756	6,369291982	7,238404739	PCP039591	XM_009357640
PCP039602	5,321928095	4,938756261	5,014801602	5,984817174		
PCP039628	6,321928095	6,38663853	6,727920455	7,169925001	PCP039628	XM_008343792
PCP039774	5,691254979	5,115615931	5,533874777	5,073391816		
PCP039793	3,938285792	4,624685811	5,169925001	5,357552005	PCP039793	XM_009346960
PCP039891	6,594996337	6,54426899	6,448405435	7,156538193	PCP039891	XM_008339713
PCP039914	4	4,480911346	5,369117459	5,448570626	PCP039914	XM_008384790
PCP039919	6,222456826	6,695854658	6,369291982	6,934634441	PCP039919	XM_009337073
PCP039958	5,922911406	6,156437068	6,321928095	7,087462841	PCP039958	XM_009338267
PCP039969	6,14974712	6,291493053	6,380764075	5,938521046		
PCP040080	6,051589621	5,882643049	5,798828178	5,285402219		
PCP040171	3,544732656	3,969933275	4,841469999	6,022367813		
PCP040196	6,333960351	5,634302842	5,426264755	5,142821844	PCP040196	XM_018642571
PCP040256	6,431790083	5,77267747	5,309976492	4,718635616		
PCP040275	6,790120385	5,866166169	6,815959618	6,486553493	PCP040275	XM_009359179
PCP040424	5,736875423	6,094447222	6,333960351	6,653490009	PCP040424	XM_009373064

PCP040427	4,297925053	4,459431619	4,807354922	5,614709844	PCP040427	XM_009373205
PCP040458	6,475733431	6,420718183	6,794415866	7,222360218		
PCP040472	6,584962501	6,481072857	6,357552005	6,950351762	PCP040472	XR_001792061
PCP040626	5,984817174	6,209453366	6,599912842	6,759289016	PCP040626	XM_009354707
PCP040635	6,464831606	6,539158811	6,62935662	7,115511897	PCP040635	XM_009367582
PCP040639	6,574858391	6,464831606	6,691115365	7,156538193		
PCP040670	6,235152624	5,624393382	5,614709844	4,874305166	PCP040670	XM_009339080
PCP040696	6,486553493	6,363346321	6,022367813	5,533874777	PCP040696	XM_009376013
PCP040724	5,961854808	5,129283017	5,470211457	5,142821844		
PCP040727	5,209453366	5,369117459	5,815831566	8,820178962	PCP040727	XM_009353702
PCP040779	5,930737338	6,414981143	5,437294331	5,815831566	PCP040779	XR_001952614
PCP040790	5,672425342	5,946496941	5,906890596	6,47037417	PCP040790	XM_009349959
PCP040797	6,807354922	7,115511897	6,705010253	6,403778984	PCP040797	XM_008388036
PCP040799	6,513016928	6,759289016	6,285402219	5,969703088	PCP040799	XM_008375940
PCP040846	7,409390936	6,309794644	5,984817174	6,272956308	PCP040846	XM_009356338
PCP040923	6,759289016	6,745909573	7,087462841	6,54951516	PCP040923	XM_009377254
PCP040946	5,898933872	5,564683017	6,015024705	6,442943496	PCP040946	XM_018645208
PCP040990	6,695854658	6,714245518	6,38663853	6,260590275	PCP040990	XM_017334565
PCP041018	4,985044962	5,101397952	5,544423562	6,101608059	PCP041018	XM_009371261
PCP041054	6,297741678	5,700439718	4,790250739	3,321928095	PCP041054	XM_008390910
PCP041094	4,115199749	3,772941338	4,34553831	6		
PCP041151	7,139551352	6,754887502	6,523561956	6,539158811	PCP041151	XM_009361839
PCP041159	6,397974049	6,497133304	6,68187088	7,156538193	PCP041159	XM_009356443
PCP041160	5,815831566	6,414981143	6,392317423	6,080444483	PCP041160	XM_009377240
PCP041184	6,291493053	6,599912842	6,459431619	6,981053471	PCP041184	XM_009366762
PCP041194	7,101503009	6,569855608	6,950351762	6,491853096	PCP041194	XM_018644645
PCP041212	6,491853096	6,54426899	7,058857621	6,569855608	PCP041212	XM_008351045
PCP041281	7,026025399	6,454011343	6,339850003	6,81159947		
PCP041284	5,906890596	5,614709844	5,392317423	5,142821844	PCP041284	XM_018645961
PCP041305	6,291493053	6,339850003	6,62935662	6,981053471	PCP041305	XM_009377229
PCP041338	5,849749117	6,058965822	6,54426899	6,080444483	PCP041338	XM_009343067
PCP041393	6,351734323	6,750472519	3,772941338	6,459431619		
PCP041397	5,922911406	6,84962403	0	6,080444483	PCP041397	XM_018646306
PCP041446	5,691254979	6,143026004	6,369291982	7,047996356	PCP041446	XM_009336296
PCP041462	6,554588852	7,412188747	6,910852562	6,297741678	PCP041462	XM_017330039
PCP041581	6,653490009	6,497133304	6,639087423	5,789990019	PCP041581	XM_008386843
PCP041656	5,513174885	5,044394119	5,156639311	4,459431619	PCP041656	XM_017325002
PCP041680	5,832890014	5,95419631	6,392317423	6,930737338	PCP041680	XM_009362655
PCP041759	5,754887502	5,727920455	5,523561956	4,874305166	PCP041759	XM_009361527
PCP041786	5,977279923	5,169925001	5,59484709	5,285402219		
PCP041803	5,073391816	4,807354922	5,59484709	5,874551087	PCP041803	XM_009347529
PCP041881	6,080444483	6,03716255	5,906890596	5,448570626	PCP041881	XM_008352712
PCP041890	6,31596467	6,321928095	6,579994693	7,196430001	PCP041890	XM_009353206
PCP041893	6,539158811	5,321928095	4,272769732	4,169925001	PCP041893	XM_009351230
PCP041901	6,247927513	6,481072857	7,577428828	6,609843592	PCP041901	XR_001953108
PCP041912	7,209453366	6,996275749	6,99242856	6,196331634	PCP041912	XM_009337260

PCP041929	6,732404887	5,969703088	6,619559738	6,820178962	PCP041929	XM_008364741
PCP041992	5,523561956	5,196528361	5,415150205	4,437627248	PCP041992	XM_009359146
PCP041993	7,42063398	7,276124405	6,890811455	5,614709844	PCP041993	XM_009359145
PCP041994	6,718772592	6,700439718	6,486553493	3,321928095	PCP041994	XM_009342677
PCP042006	6,297741678	6,448405435	6,554588852	7,073284692		
PCP042045	5,906890596	6,196331634	6,790120385	6,857980995	PCP042045	XM_009378580
PCP042050	6,464831606	6,163297449	5,634302842	4,874305166	PCP042050	XM_009364490
PCP042076	7,189824559	6,890811455	6,837060204	6,196331634	PCP042076	XM_009355503
PCP042176	5,169925001	5,415150205	5,634302842	6,363346321		
PCP042194	7,327956767	7,026025399	7,260496187	5,60496087	PCP042194	XR_001791541
PCP042220	5,297558281	4,754887502	5,691254979	6,50779464	PCP042220	XM_009337436
PCP042226	6,247927513	6,564835417	6,136068312	5,961854808	PCP042226	XM_009345937
PCP042259	6,24164954	5,709566354	6,4374608	6,619559738	PCP042259	XM_009367927
PCP042271	6,414981143	6,589913261	6,564835417	7,183188734	PCP042271	XM_009368086
PCP042280	6,051589621	6,189824559	6,266786541	6,890811455	PCP042280	NM_001300877
PCP042320	5,815831566	0	5,824513297	6,475733431	PCP042320	XM_009356405
PCP042395	6,044394119	4,790250739	6,475733431	6,798698597	PCP042395	XM_008390952
PCP042402	6,714245518	6,189824559	6,080444483	5,807354922	PCP042402	XM_009368318
PCP042490	5,634302842	6,122465658	6,108524457	6,837060204	PCP042490	XM_009362596
PCP042501	6,272956308	6,06608919	6,303780748	6,841344192		
PCP042508	5,196528361	4,95419631	5,64385619	6,420718183	PCP042508	XM_008352409
PCP042528	6,143026004	6,235152624	6,862079387	6,392317423	PCP042528	XM_008349227
PCP042536	4,969472865	5,634302842	5,874551087	5,938521046		
PCP042607	6,183288001	6,176522247	5,614709844	4,969472865	PCP042607	XM_009354543
PCP042715	5,634302842	5,380937195	5,448570626	5		
PCP042737	6,333960351	3,772941338	6,754887502	6,648609245	PCP042737	XM_009351697
PCP042765	6,380764075	6,574858391	6,737010678	7,073284692	PCP042765	XM_009380498
PCP042778	6,653490009	6,528727582	6,333960351	6,303780748	PCP042778	XM_017336556
PCP042794	5,470211457	5,554588852	5,882643049	6,303780748	PCP042794	XM_018647467
PCP042801	6,397974049	6,54426899	6,397974049	7,076815597	PCP042801	XM_009358091
PCP042826	6,454011343	6,136068312	6,080444483	6		
PCP042882	6,272956308	6,695854658	6,409390936	6,14974712		
PCP043031	5,321928095	4,922673593	5,653346693	6,420718183	PCP043031	XM_009335653
PCP043048	6,741466986	6,080444483	6,481072857	6,24164954		
PCP043062	5,073391816	5,672425342	4,480911346	6,101608059		
PCP043093	5,357552005	5,209453366	5,95419631	6,619559738	PCP043093	XM_009346497
PCP043114	5,969703088	5,718909554	5,544423562	5,273142859	PCP043114	XM_008368766
PCP043116	7,589988142	7,639015048	6,845490051	0		
PCP043120	5,882643049	4,906890596	6,087462841	5,156639311		
PCP043157	6,518377768	6,853870927	6,662917555	6,215872703		
PCP043214	5,392317423	5,857980995	6,163297449	6,285402219		
PCP043215	6,634157606	6,279285561	6,763809907	7,269874722	PCP043215	XM_008387771
PCP043241	8,318904285	6,007419784	6,589913261	6,594996337		
PCP043243	5,60496087	5,403608584	5,169925001	4,64385619	PCP043243	XM_009345176
PCP043256	6,781359714	6,599912842	6,31596467	5,459431619		
PCP043307	6,044394119	5,60496087	6,058965822	5,554588852	PCP043307	XM_018651438

PCP043342	5,156639311	4,143230135	4,73714592	4,272769732	PCP043342	XM_008381218
PCP043369	6,513016928	6,62935662	6,691115365	7,199672345	PCP043369	XM_009341615
PCP043409	6,007419784	5,718909554	5,554588852	5,426264755		
PCP043442	5,285402219	4,502712486	4,718635616	4,414812061	PCP043442	XM_017335132
PCP043461	6,564835417	6,700439718	6,794415866	7,327956767	PCP043461	XM_009362215
PCP043632	4	4,34553831	4,874305166	5,369117459	PCP043632	XM_018649592
PCP043663	6,203005338	5,57500972	5,02989455	4,64385619		
PCP043751	5,470211457	5,235344128	5,781359714	6,235152624	PCP043751	XM_008351567
PCP043765	6,705010253	6,832890014	6,609843592	6,420718183		
PCP043823	5,544423562	6,297741678	6,996275749	7,684187561	PCP043823	XM_018642618
PCP043857	5,890689878	6,058965822	6,327866971	5,727920455	PCP043857	XM_008364922
PCP043912	5,448570626	5,101397952	5,523561956	4,584962501	PCP043912	XM_009366467
PCP043937	6,291493053	6,790120385	6,579994693	5,946496941	PCP043937	XM_009350706
PCP043963	5,798828178	5,624393382	5,426264755	5,156639311	PCP043963	XM_009359085
PCP043986	5,297558281	5,906890596	5,841218374	6,38663853	PCP043986	XM_009368536
PCP043988	6,614709844	5,992541859	6,058965822	5,260402093	PCP043988	XM_017334373
PCP044001	4,414812061	4,480911346	4,874305166	5,718909554	PCP044001	XM_009347025
PCP044003	5,142821844	4,624685811	4,247927513	5,746043983	PCP044003	XM_009338374
PCP044007	5,992541859	6,60481265	6,209453366	6,686500527	PCP044007	XM_017336489
PCP044044	6,31596467	5,938521046	5,64385619	5,874551087	PCP044044	XM_008372563
PCP044049	4,321928095	4,544114402	4,857980995	5,564683017	PCP044049	XM_009341631
PCP044070	6,101608059	5,969703088	5,992541859	4,874305166	PCP044070	XM_009357766
PCP044086	4,584962501	4,624685811	5,357552005	6,890811455	PCP044086	XM_009376463
PCP044091	5,129283017	5,392317423	5,634302842	6,101608059		
PCP044092	5,922911406	5,781359714	6,14974712	7,115511897		
PCP044109	5,938521046	6,47037417	5,798828178	6,015024705	PCP044109	XM_008387872
PCP044118	5,321928095	5,866166169	6,777025123	7,375039431	PCP044118	XM_018645358
PCP044121	6,058965822	5,02989455	5,235344128	5,938521046	PCP044121	XM_008352516
PCP044137	6,101608059	6,564835417	6,50779464	6,841344192	PCP044137	XM_009371005
PCP044152	4,754887502	5,403608584	5,470211457	5,914803677	PCP044152	XM_008362692
PCP044154	5,969703088	6,303780748	6,459431619	6,996275749	PCP044154	XM_008395057
PCP044162	6,837060204	6,176522247	6,491853096	7,225641265	PCP044162	XM_009342170
PCP044193	4,169925001	5,763677142	5,101397952	6,260590275	PCP044193	XR_667243
PCP044314	5,309976492	5,672425342	6,279285561	6,86628983	PCP044314	XM_008361622
PCP044329	6,403778984	6,22881869	6,624539604	7,153095972	PCP044329	XM_008370492
PCP044330	6,768184325	6,464831606	6,68187088	6,339850003	PCP044330	XM_009353946
PCP044344	5,634302842	5,533874777	5,544423562	4,906890596	PCP044344	XM_009353184
PCP044345	5,101397952	5,984817174	5,554588852	6,07317756	PCP044345	XM_009336486
PCP044349	6,044394119	6,094447222	5,789990019	5,273142859	PCP044349	XM_009348559
PCP044372	6,22881869	6,397974049	6,894817763	7,462134139	PCP044372	XM_008378545
PCP044399	7,040782866	6,554588852	6,727920455	6,363346321	PCP044399	XM_009367896
PCP044458	5,415150205	4,969472865	5,196528361	4,321928095	PCP044458	XM_017332682
PCP044462	5,969703088	6,044394119	6,584962501	6,965784285	PCP044462	XM_018650005
PCP044468	6,351734323	6,06608919	6,609843592	7,122362117	PCP044468	XM_009335630
PCP044472	5,273142859	4,64385619	4,369466484	4,437627248	PCP044472	XR_001953351
PCP044482	6,247927513	6,357552005	6,327866971	6,958030641		

PCP044487	6,910852562	7,044394119	6,051589621	6,136068312	PCP044487	XM_009366643
PCP044492	4,523561956	3,906890596	4,459431619	5,333781501		
PCP044494	5,02989455	5,247927513	5,906890596	7,163196797	PCP044494	XM_009365070
PCP044497	5	5,087462841	5,297558281	6,03716255	PCP044497	XM_009344385
PCP044503	6,321928095	6,31596467	6,115407855	5,798828178	PCP044503	XM_009348006
PCP044507	6,705010253	6,528727582	6,64385619	6,31596467	PCP044507	XM_009337931
PCP044528	4,772413555	5,874551087	5,992541859	6,215872703	PCP044528	XM_009376174
PCP044536	5,183089461	4,969472865	5,653346693	4,700439718		
PCP044539	5,874551087	5,789990019	6,497133304	7,073284692	PCP044539	XM_021975909
PCP044596	5,60496087	6	5,977279923	6,559644763	PCP044596	XM_009347218
PCP044598	5,984817174	6,464831606	7,142923928	7,46760555	PCP044598	XM_018647026
PCP044606	6,886672074	6,781359714	6,841344192	6,409390936	PCP044606	XM_009348505
PCP044628	4,95419631	5,087462841	4,969472865	4,169925001		
PCP044671	6,38663853	6,50779464	6,672425342	7,357552005	PCP044671	XM_009367573
PCP044687	6,06608919	5,426264755	4,544114402	3		
PCP044694	5,789990019	4,874305166	4,584962501	3,459431619	PCP044694	XM_009365818
PCP044707	7,087462841	6,589913261	6,874428132	6,68187088	PCP044707	NM_001328854
PCP044713	5	4,807354922	5,260402093	4,029452886	PCP044713	XM_009359904
PCP044765	5,672425342	6,03716255	5,841218374	6,564835417		
PCP044766	5,95419631	5,798828178	5,235344128	3,840966704	PCP044766	NM_001302310
PCP044801	6,431790083	5,64385619	5,946496941	4,807354922		
PCP044810	4,34553831	4,95419631	5,426264755	4,604664415	PCP044810	XM_009343629
PCP044816	6,72342204	5,691254979	6,06608919	5,297558281	PCP044816	XM_009361395
PCP044865	5,832890014	5,663059924	5,841218374	6,497133304	PCP044865	XM_009354021
PCP044917	5,044394119	5,357552005	5,142821844	6,015024705		
PCP044920	5,101397952	5,369117459	5,77267747	5		
PCP044935	6,108524457	5,209453366	5,309976492	5,448570626	PCP044935	XM_009353642
PCP044949	6,209453366	6,363346321	6,714245518	7,101503009		
PCP044952	5,59484709	5,700439718	5,700439718	6,459431619		
PCP044972	5,273142859	5,984817174	6,303780748	7,132679654	PCP044972	XM_009343338
PCP044973	6,414981143	6,448405435	6,380764075	6,094447222	PCP044973	XM_017336792
PCP045007	5,614709844	5,906890596	6,50255338	5,807354922		
PCP045012	5,961854808	6,579994693	6,658211483	7,029784146		
PCP045037	6,894817763	6,272956308	5,403608584	3,807354922	PCP045037	XM_009378719
PCP045076	6,559644763	6,47037417	6,677085351	5,961854808		
PCP045110	6,303780748	6,50255338	6,777025123	7,541716163	PCP045110	XR_527167
PCP045124	6,397974049	6,584962501	6,50255338	7,199672345		
PCP045125	5,624393382	5,946496941	5,544423562	6,497133304	PCP045125	XR_001788132
PCP045141	7,415065677	6,984931073	6,95419631	6,015024705	PCP045141	XM_009345316
PCP045146	6,254178286	6,03716255	6,426264755	6,882643049	PCP045146	XM_009379537
PCP045171	6,969587981	6,534030467	6,54426899	6,672425342	PCP045171	XM_018647000

Cluster_2

Gene	H1	H2	H3	H4		
PCP000177	0	-0,577766999	1,735522177	3,415488271		
PCP000383	0,411426246	0	1,416839742	3,415488271	PCP000383	XM_017337146
PCP000475	-0,577766999	0	0	3,874796966		
PCP002039	0	0	1,220329955	2,807354922		
PCP002967	0	0	1,735522177	2,737686761		
PCP003539	0	0	0	4,272769732	PCP003539	XM_009378409
PCP004758	0	0	0,411426246	4,718635616	PCP004758	XM_008354939
PCP005207	0	0	3	6,639087423	PCP005207	XM_009371003
PCP005765	0	0	1,220329955	4,321928095		
PCP006311	-0,577766999	0	0,411426246	3,502075956	PCP006311	XM_009346667
PCP007616	0	0	1	3,663344619	PCP007616	XM_009363915
PCP007647	2,22342255	0	1	2,807354922	PCP007647	XM_008357117
PCP009358	1,875780063	0	-0,577766999	3,169925001	PCP009358	XM_009361451
PCP009368	0	0	2,584962501	4,754887502		
PCP011192	0	0,411426246	-0,577766999	2,584962501	PCP011192	XM_009353816
PCP011500	0	1,416839742	0	4,029452886	PCP011500	NM_001302276
PCP011895	0	0	0	6,339850003	PCP011895	XM_009345085
PCP012324	0	0	0	4,087462841	PCP012324	XM_009351206
PCP012330	0	-0,577766999	3,221877081	5,57500972	PCP012330	XM_009351202
PCP013122	0	0	3	5,554588852	PCP013122	XM_009354335
PCP013493	0,411426246	1,416839742	1,735522177	4,34553831	PCP013493	XR_667379
PCP014159	-1,59946207	-0,577766999	0,411426246	3	PCP014159	XM_009341342
PCP014409	1,584962501	0	2,22342255	5,789990019	PCP014409	XM_009377872
PCP015327	0	0	0	6,303780748		
PCP016456	0	0	-0,577766999	2,737686761	PCP016456	XM_009375679
PCP018339	0	0,411426246	2,584962501	4,700439718	PCP018339	XM_009362609
PCP019525	1,220329955	-0,577766999	3,368768349	4,73714592		
PCP020766	-0,577766999	1,584962501	1,584962501	5,235344128		
PCP021608	0	0	0	4,222650022		
PCP021703	0	0	2,321928095	3,169925001		
PCP021992	0,411426246	0	0	4,700439718	PCP021992	XM_009376899
PCP022236	0	0	0	2,873813198	PCP022236	XM_009378442
PCP024590	-0,577766999	0	0	3,874796966	PCP024590	XM_009348778
PCP025035	1,416839742	1,220329955	0,739848103	6,254178286	PCP025035	XM_009358319
PCP025482	0,411426246	0	1	2,939226578	PCP025482	XM_008344255
PCP025771	0	0	0,411426246	3,27351589		
PCP026132	0	0	0	3,321928095	PCP026132	XM_008354775
PCP026134	-0,577766999	0	1	4,544114402	PCP026134	XM_008354774
PCP026374	0	0	1,416839742	2,737686761	PCP026374	XM_009338202
PCP027843	0	-0,577766999	0	4,392317423	PCP027843	XM_009358762
PCP029568	0	0	0	4,480911346	PCP029568	XM_009362979
PCP029646	-1,59946207	0	-1,59946207	3	PCP029646	XM_009361420
PCP030582	-0,577766999	1,875780063	0,411426246	3,321928095	PCP030582	XM_009341740

PCP031262	-0,577766999	1,584962501	1,220329955	6,882643049	PCP031262	XM_009367965
PCP032052	0	0,739848103	0	5,415150205	PCP032052	XM_008354940
PCP033855	0	0	0	8,936637939	PCP033855	XM_021951507
PCP034675	0	0	0	3,502075956	PCP034675	XM_009366330
PCP035856	0	0,739848103	2	3,840966704	PCP035856	XM_008384758
PCP036486	0	0	1,220329955	5,044394119	PCP036486	XM_008368821
PCP040164	0,739848103	0,411426246	2,321928095	4,459431619	PCP040164	XR_001953975
PCP041332	0	0	2,114367025	3,807354922	PCP041332	XM_009360390
PCP041515	0	0	0	2,939226578		
PCP041918	-0,577766999	0	0,739848103	5,247927513	PCP041918	XM_009337233
PCP041919	-1,59946207	0	0,411426246	4,985044962	PCP041919	XM_009337233
PCP041951	0	0,411426246	0	3,840966704		
PCP042384	0,739848103	0	1	3,459431619	PCP042384	XR_667961
PCP043886	0	1,735522177	0	2,939226578		
PCP044182	2,6622055	0,411426246	0	2,939226578	PCP044182	XM_009377643
PCP044298	0	0,411426246	-0,577766999	4,437627248	PCP044298	XM_009352526
PCP044465	0	0	0,739848103	5,073391816		
PCP045074	0	0	2,807354922	5,222263604	PCP045074	XM_009361465
PCP045095	3,459431619	0	0,411426246	3,27351589		

Cluster_3

Gene	H1	H2	H3	H4		
PCP000537	-0,577766999	3,700439718	0	2,321928095	PCP000537	XM_009360580
PCP001192	0,411426246	1,584962501	2,22342255	3,624100895	PCP001192	XM_018648295
PCP001276	0	2,807354922	1	2,807354922		
PCP002412	0	2,939226578	2,939226578	2,114367025	PCP002412	XM_009349437
PCP003039	0	2,114367025	1	2,939226578	PCP003039	XM_008391616
PCP003569	-0,577766999	1,584962501	2,584962501	3,459431619	PCP003569	XM_018642824
PCP006802	0,739848103	1,584962501	2,414135533	5,183089461	PCP006802	XM_008368837
PCP007786	-0,577766999	2,737686761	2,321928095	4,029452886	PCP007786	XM_017337427
PCP008995	1,416839742	2,503348735	1,735522177	4,604664415	PCP008995	XM_009361153
PCP009575	1	2,737686761	2,321928095	3,874796966		
PCP009764	0,411426246	2,737686761	1,875780063	5,700439718	PCP009764	XM_009359115
PCP010883	1,416839742	2,503348735	2,321928095	4	PCP010883	XM_009358577
PCP013542	0	3,544732656	2,737686761	4,790250739		
PCP014077	0	2,114367025	3,27351589	3,624100895	PCP014077	XM_009360493
PCP014684	0	2,584962501	2,807354922	2,873813198	PCP014684	XM_009353638
PCP015138	0,739848103	5,380937195	1,735522177	2,807354922	PCP015138	XM_009379473
PCP018320	0	2,807354922	0,739848103	2		
PCP018954	1,416839742	2,873813198	0,739848103	4,196134881	PCP018954	XM_018645229
PCP019091	0	3,116031993	1,416839742	1,584962501		
PCP019828	1	1,735522177	2,22342255	4,544114402	PCP019828	XM_009335623
PCP020117	-0,577766999	2,503348735	3,906890596	4,169925001	PCP020117	XM_018651228
PCP022491	-0,577766999	2,414135533	3,368768349	3,415488271	PCP022491	XM_008343921
PCP022563	2,321928095	2,807354922	1,220329955	4,369466484	PCP022563	XM_009336969
PCP022619	0	1,735522177	2,114367025	4,029452886		
PCP022688	0	3,736604875	0	3,368768349	PCP022688	XM_009379762
PCP023955	-0,577766999	3	2,414135533	3,584962501		
PCP025021	0	2,873813198	0	1,875780063	PCP025021	XM_017332207
PCP025421	3,221877081	2,584962501	0	3,321928095		
PCP026196	-0,577766999	5,380937195	1,584962501	4,414812061	PCP026196	XM_018649523
PCP026816	-0,577766999	5,285402219	1,584962501	2,503348735	PCP026816	XM_009350298
PCP027529	0	2,114367025	1,220329955	2,807354922	PCP027529	XM_009345414
PCP028063	-0,577766999	3,772941338	2	2,414135533		
PCP028452	0,411426246	1,584962501	2	4,604664415	PCP028452	XM_008378043
PCP030682	3,058316496	3,116031993	0	4,143230135	PCP030682	NM_001293841
PCP031248	0	2,22342255	2,321928095	3,584962501	PCP031248	XM_009364231
PCP031664	0	2,6622055	3,663344619	3,415488271		
PCP034348	0	4,087462841	2,414135533	4,502712486		
PCP035013	1,220329955	4,297925053	1,220329955	3,221877081	PCP035013	XM_009365849
PCP035060	-0,577766999	2,807354922	3,584962501	2,584962501	PCP035060	XM_009369774
PCP035233	1,735522177	3,736604875	0	3,221877081	PCP035233	XM_009346770
PCP037970	0	2,414135533	2,414135533	2,873813198		
PCP039257	2,114367025	2,873813198	0,739848103	4,718635616		
PCP040058	0,739848103	2,114367025	3,116031993	4,143230135		

PCP043452	0	3,969933275	-0,577766999	2,22342255	PCP043452	XM_008357808
PCP044681	1,735522177	2,807354922	0	2,6622055		

Cluster_4

Gene	H1	H2	H3	H4		
PCP002652	0	0,411426246	2,6622055	2,584962501	PCP002652	XM_009349728
PCP003681	1	0,411426246	2,6622055	3,938285792	PCP003681	XM_009347210
PCP003756	0,739848103	1,584962501	2,939226578	3,840966704	PCP003756	XM_008376175
PCP005668	0	1,220329955	3,368768349	4,115199749	PCP005668	XM_017330330
PCP005923	1,735522177	1,416839742	4,169925001	2,6622055	PCP005923	XM_008360216
PCP007726	0	0,739848103	2,873813198	2,737686761	PCP007726	XM_018652391
PCP011324	1,875780063	1,416839742	4,272769732	2,503348735	PCP011324	XM_009370600
PCP011327	-0,577766999	0,739848103	3,938285792	2,807354922	PCP011327	XM_009370595
PCP011336	2,114367025	0,739848103	2,6622055	4,272769732	PCP011336	XM_009370577
PCP012231	0	1,220329955	2,6622055	3,058316496		
PCP015025	0	1	3,502075956	2		
PCP015054	1,875780063	1	3,415488271	4,247927513	PCP015054	XM_009363864
PCP017991	2,114367025	0	2,737686761	3,27351589	PCP017991	XM_009372934
PCP020197	3,321928095	0	2,414135533	2,22342255		
PCP020222	1,735522177	0	3,584962501	3,544732656	PCP020222	XM_009350919
PCP023488	0,411426246	0	4,414812061	0	PCP023488	XM_009368310
PCP023657	0	0	3,116031993	1,875780063	PCP023657	XM_009347177
PCP024232	0,411426246	-0,577766999	3,415488271	3,459431619	PCP024232	XM_008391522
PCP024472	1	0	3,736604875	1	PCP024472	XM_009346054
PCP026551	0	0,739848103	3,221877081	2	PCP026551	XM_009368030
PCP027668	2,414135533	0,739848103	3,840966704	1,735522177	PCP027668	XM_009367399
PCP028412	1,416839742	1	3,906890596	2,939226578	PCP028412	XM_009349100
PCP029683	0,411426246	0	5,014801602	4,73714592	PCP029683	XM_008383683
PCP030402	2,114367025	0,411426246	3,169925001	3,874796966	PCP030402	XM_008381038
PCP030684	0	2,114367025	3	1,584962501	PCP030684	NM_001294352
PCP031312	0	0	4,169925001	5,014801602	PCP031312	XM_009339250
PCP034856	0	0,411426246	3,807354922	4,029452886	PCP034856	XM_018645673
PCP035254	-0,577766999	1,735522177	3,584962501	1,584962501	PCP035254	XM_009346397
PCP037666	3,116031993	0	3,874796966	2		
PCP038193	0	0	2,737686761	0		
PCP038399	1,875780063	0	3,058316496	1,735522177		
PCP040142	1,220329955	1,735522177	4,143230135	3,169925001	PCP040142	XM_018650159
PCP042238	0	0	10,03250037	0		
PCP042994	3,116031993	0	2,6622055	3,221877081		
PCP045114	0	1,735522177	3,116031993	2,584962501	PCP045114	XM_009340090

Cluster_5

Gene	H1	H2	H3	H4		
PCP000012	8,934663924	8,915879379	9,103287808	9,646252679	PCP000012	XM_008369041
PCP000031	8,839203788	8,949330653	8,991521846	9,388727692		
PCP000068	10,95613654	10,85200664	10,96914905	10,8930512	PCP000068	XM_018651796
PCP000073	14,62319564	14,80041107	14,85021956	15,27740837	PCP000073	XM_009358716
PCP000074	11,58135596	11,43028005	11,18920683	11,44035012	PCP000074	XM_009358717
PCP000080	9,515049296	9,146365017	9,652844973	9,849139214	PCP000080	XM_008375279
PCP000085	10,63873456	10,40442845	10,67213359	10,96938652	PCP000085	XM_018651224
PCP000090	9,740337332	9,82707272	9,801433534	9,750991645	PCP000090	XM_009375223
PCP000091	9,852264198	9,734150722	9,758223215	9,806275718		
PCP000094	8,648645193	8,970594857	9,273026268	9,614102459	PCP000094	XM_009375211
PCP000100	8,9905862	9,046223651	9,2589664	9,523561956		
PCP000129	11,66222331	11,23342195	11,52028964	11,28424575		
PCP000158	8,893817229	9,166589927	9,299963518	9,253445696	PCP000158	XM_009377990
PCP000164	9,169098221	9,095397023	9,345782228	9,585582276	PCP000164	XM_008349845
PCP000165	10,51897765	10,45771945	10,47065887	11,00469099	PCP000165	XM_008381121
PCP000189	8,984902599	9,072347016	9,120678526	9,566054038	PCP000189	XM_018643790
PCP000200	10,20252792	10	9,991054099	10,32192809	PCP000200	XM_008372600
PCP000207	9,063395081	8,967716202	9,005624549	9,337621902	PCP000207	XM_008365829
PCP000217	9,726218159	9,611024797	9,668884984	9,591204414	PCP000217	XM_009339700
PCP000218	9,49251418	9,68707819	10,15396996	10,17201489	PCP000218	XM_009339697
PCP000241	11,2644426	11,43688328	11,69087101	12,08978149	PCP000241	XM_009339665
PCP000256	9,122388003	9,125852541	9,28000515	9,840258649		
PCP000258	9,164077264	9,014941046	9,296916207	9,077696975	PCP000258	XM_008380915
PCP000280	9,88519302	9,781359714	9,688827272	10,06474276	PCP000280	XM_009342576
PCP000295	9,731879027	9,543669869	9,790625442	9,986794882	PCP000295	XR_001788324
PCP000299	9,323437626	9,559051993	9,507140523	9,275356315	PCP000299	XM_008367147
PCP000300	10,40691714	10,92208632	11,04666703	11,33278065	PCP000300	XM_008380971
PCP000311	10,43775207	10,52061868	10,70102049	11,029515	PCP000311	XM_009357352
PCP000320	10,19229281	9,988216019	10,08569817	10,13484983		
PCP000330	9,809494835	9,727920455	9,971069283	10,16908569	PCP000330	XM_008379508
PCP000331	14,10451685	13,78391905	13,56625268	13,25571758	PCP000331	XM_009357380
PCP000357	10,29729461	10,39553414	10,54624819	10,78408678	PCP000357	XM_009371792
PCP000363	11,7926561	11,63903767	11,73442596	11,71094846		
PCP000381	9,408690635	9,453949632	9,588096205	9,506466274	PCP000381	XM_008341858
PCP000390	9,095397023	9,235224441	9,28771238	9,674774879	PCP000390	XM_008362488
PCP000394	8,76927586	8,938609256	9,037080165	8,918863237	PCP000394	XM_017323742
PCP000395	10,67771964	10,92530964	10,91787179	11,203348	PCP000395	XM_008341829
PCP000399	11,3824457	11,26346288	11,31948585	11,59479112		
PCP000401	11,62555817	11,63964369	11,70663847	11,68357804		
PCP000432	8,797661526	8,689997971	8,887738491	9,145499145		
PCP000433	9,004698008	9,022367813	9,082149041	9,45532722	PCP000433	XM_009371230
PCP000456	8,911901261	8,775873612	8,946409212	8,721099189	PCP000456	XM_008355035
PCP000457	10,84470576	11,04165915	10,63873456	10,81991561	PCP000457	NM_001301125

PCP000464	8,85277917	9,131008067	9,433940073	9,988684687	PCP000464	XM_009353736
PCP000465	9,328674927	9,596804728	9,862637358	10,73244729		
PCP000469	9,28000515	9,439477697	9,532687093	9,786808699	PCP000469	XM_009351676
PCP000472	9,714245518	9,423473125	9,860574891	10,08391805	PCP000472	XM_009361882
PCP000476	8,784634846	8,860062694	8,857980995	8,842350343		
PCP000497	10,12412131	10,26912668	10,16741815	10,28424575		
PCP000501	9,724513853	9,736401931	9,718241739	9,640841416	PCP000501	XM_008369305
PCP000504	9,736401931	9,836050355	9,959523851	10,44224893		
PCP000508	10,01634867	9,434628228	9,387306529	9,433940073		
PCP000521	9,916372141	10,00748986	10,03021191	10,56478779		
PCP000522	9,82707272	9,862637358	9,843921051	10,29883012		
PCP000528	10,14932797	10,5685927	10,74483384	10,70908381	PCP000528	XM_009360561
PCP000562	10,66681871	10,75043065	10,87651695	10,74034577	PCP000562	XM_009373156
PCP000563	9,393025715	9,174101763	9,152284842	9,169098221	PCP000563	XM_009373154
PCP000574	8,953236133	9,177419538	9,123267853	9,636624621	PCP000574	XM_008348731
PCP000583	9,406566431	9,452591317	9,538518764	9,813781191	PCP000583	XM_009352013
PCP000589	9,429762402	9,225617167	9,537218401	9,438791853	PCP000589	XR_668982
PCP000600	10,02375435	10,14551188	10,10895563	10,16783208		
PCP000605	9,52160044	9,299963518	9,342808055	9,626931876	PCP000605	XM_008345725
PCP000614	11,19557522	11,44983474	11,63994208	11,63359922	PCP000614	XM_008387554
PCP000624	8,922822231	8,984902599	9,083027168	9,574271841		
PCP000631	9,380828997	9,458057945	9,64385619	10,00656452		
PCP000643	9,792790294	9,646252679	10,0018726	10,07056375	PCP000643	XM_009374201
PCP000668	11,65015421	11,44432645	11,05934446	10,29653771	PCP000668	XM_009359625
PCP000670	11,23002044	10,83736775	11,04598485	10,97561156	PCP000670	XR_667423
PCP000676	9,573647187	9,510427928	9,729620744	10,18156227		
PCP000693	9,019590728	8,939579214	9,056177063	8,976334992	PCP000693	XM_018652432
PCP000709	9,415044544	9,362667132	9,320416982	9,646252679		
PCP000724	9,266786541	9,275356315	9,358277568	10,32117274		
PCP000735	11,89772069	11,95830983	12,31665771	13,12487575	PCP000735	XM_008376055
PCP000737	11,66118576	11,49984589	11,60994976	11,47082662	PCP000737	XM_009370526
PCP000739	8,614709844	8,694636474	8,883651361	9,216745858	PCP000739	XM_018643516
PCP000762	8,875503635	9,023283079	8,960001932	9,441554026		
PCP000765	11,11069862	10,97513146	10,97799537	10,90889545	PCP000765	XM_008392669
PCP000777	9,742595757	10,07012094	10,20579325	11,08679969	PCP000777	XM_008367071
PCP000798	9,949345246	9,952260754	9,836050355	9,884170519		
PCP000825	9,255807556	9,401583539	9,361943774	9,69406183	PCP000825	XM_008349140
PCP000826	9,634212071	9,904890476	10,41961263	11,56160777	PCP000826	XM_008378979
PCP000839	10,22560512	10,2467406	10,31212284	10,23920469	PCP000839	XM_008395642
PCP000852	9,33911511	9,162391329	10,32942637	11,00819749	PCP000852	XM_009351836
PCP000858	10,11504365	9,257387843	9,600526229	9,874474241	PCP000858	XM_009370938
PCP000865	10,46318716	10,36850646	10,22962397	9,788441022	PCP000865	XM_009370949
PCP000876	10,99175566	11,29347165	10,9427564	10,77423524		
PCP000891	10,81618368	10,54496443	10,95201316	11,33222121	PCP000891	XM_009377296
PCP000897	10,93737382	10,76983784	10,63390341	10,78244952	PCP000897	XM_009377225
PCP000898	8,938609256	8,908902953	9,013099307	9,469641817	PCP000898	XM_008353496

PCP000909	10,72678865	10,79143154	10,90312868	11,11157259		
PCP000941	9,915386449	9,564778269	9,752096261	10,14974712	PCP000941	XM_008351495
PCP000965	11,65344075	11,32230563	11,44570291	11,97441459	PCP000965	XM_009363475
PCP000983	9,840258649	9,792253453	9,711374448	9,824433739		
PCP000985	9,77807713	9,899356923	9,193944186	8,942514505	PCP000985	XM_008383768
PCP001008	12,3836152	11,64084593	11,80829253	12,01564851		
PCP001032	9,071462363	9,274564521	9,240791332	9,646252679	PCP001032	XM_017325543
PCP001035	10,12066557	10,07771032	10,15692998	9,866243459	PCP001035	XM_009359620
PCP001039	9,272233193	9,818582177	10,02329694	10,46658634	PCP001039	XM_009345447
PCP001047	8,891783703	8,890781061	9,010304306	8,54689446	PCP001047	XM_008394323
PCP001097	10,28038801	10,46079375	10,55298603	10,67360059	PCP001097	XM_009352961
PCP001098	9,296159109	9,691743519	10,01029032	10,43948809	PCP001098	XM_008380259
PCP001110	8,883651361	8,713111526	8,80196697	9,345782228	PCP001110	XM_018644259
PCP001114	9,155653112	9,05709822	9,207819661	9,570481906	PCP001114	XM_009336912
PCP001119	11,241584	11,22359752	11,18281022	10,41397693	PCP001119	XM_009381040
PCP001129	10,66415392	11,13164478	11,18693752	11,00328599	PCP001129	XM_009348332
PCP001130	8,87857209	8,862110391	9,145499145	9,535916864	PCP001130	XM_009348333
PCP001150	9,353874607	9,229611955	9,167418146	9,118084543		
PCP001169	10,00281502	9,936152052	9,941531848	9,924322617	PCP001169	XM_009345160
PCP001194	12,01970539	11,93725243	11,93135434	11,39998815		
PCP001204	8,988684687	9,568583198	9,400879436	9,350939182	PCP001204	XM_008347810
PCP001220	10,80467153	10,65553072	10,79928162	11,07258149	PCP001220	XM_009366886
PCP001223	8,776992236	8,723387907	8,952246191	9,211888295		
PCP001226	9,327170869	9,112882543	9,334653186	9,288473922	PCP001226	XM_009366877
PCP001232	10,35718909	10,24475627	10,26561505	10,20171962	PCP001232	XM_008349189
PCP001238	12,77197015	11,82959194	12,15671514	12,22691488		
PCP001264	11,46624474	11,06877828	11,20742315	11,49685378	PCP001264	XM_018652431
PCP001283	9,46760555	9,581821975	9,648051932	10,2199792	PCP001283	XM_009361921
PCP001286	9,458755197	9,377926277	9,607940556	9,915386449		
PCP001294	11,03800673	10,9653009	11,04006364	11,35112144		
PCP001298	8,478446064	8,866258916	8,894817763	9,189009107		
PCP001308	8,541754876	8,924812504	9,562872035	10,26951244		
PCP001317	11,65194861	11,44121683	11,7641791	12,00842862	PCP001317	XM_009370295
PCP001326	9,2644426	9,845490051	9,990103964	9,959031118		
PCP001335	10,72763971	10,72820114	10,71967117	10,66917739	PCP001335	XM_009362989
PCP001339	9,581200582	9,517019734	9,657014692	9,962896005	PCP001339	XM_017324176
PCP001345	8,913876736	9,017727086	8,862110391	8,892785649	PCP001345	XM_008376091
PCP001368	9,762664404	9,754336368	9,633594681	9,779177627	PCP001368	XM_017336506
PCP001385	9,13442632	9,41715631	9,544326957	9,251884747	PCP001385	XM_009348684
PCP001467	9,758223215	9,648663167	9,616548844	9,532024681	PCP001467	XM_009340916
PCP001503	11,09824926	11,20273604	11,37395266	12,39419471	PCP001503	XM_009358640
PCP001684	9,112882543	9,164077264	9,559702114	9,706789535		
PCP001686	10,84418005	11,18177344	11,18920683	11,26912668	PCP001686	XM_009350898
PCP001691	12,75641663	12,5525082	12,15228484	12,1424262	PCP001691	XM_009350840
PCP001700	10,16616308	9,897845456	10,41045135	9,767638247	PCP001700	XM_009350755
PCP001721	9,796575043	9,668884984	9,899356923	10,2911707		

PCP001731	8,705044823	8,705044823	8,988684687	9,169925001		
PCP001754	9,571126898	9,330177419	9,742023058	9,302250498		
PCP001757	11,48112837	11,46284476	11,5996014	11,34059008	PCP001757	XM_009358251
PCP001783	9,314401976	9,469641817	9,419264989	9,891282469	PCP001783	XM_009358191
PCP001794	9,609788199	9,726780139	9,813781191	10,30378075		
PCP001801	9,535916864	9,589969422	9,74819285	9,626931876	PCP001801	XM_018647479
PCP001804	10,11591499	10,04484489	10,12196082	10,05301628	PCP001804	XM_018647477
PCP001833	9,312882955	9,312882955	9,419960178	9,824958741		
PCP001840	11,00211178	11,20130917	11,378116	11,36540931	PCP001840	XM_009357761
PCP001841	9,358277568	9,185693134	9,810041371	9,206208529		
PCP001843	9,119797095	9,090985714	9,142949447	8,966736149		
PCP001844	10,7428736	10,54271269	10,79116289	10,60084211	PCP001844	XM_009357656
PCP001857	10,26717293	10,41855893	10,29920802	10,74455637	PCP001857	XM_009345357
PCP001863	9,318926875	9,142107057	8,970594857	9,401583539	PCP001863	XM_018649986
PCP001903	10,22921538	10,31628153	10,3829806	10,66917739	PCP001903	XM_018645108
PCP001938	10,56192752	10,58183139	10,65612986	10,23801057		
PCP001943	9,973453429	9,842868866	10,02236781	10,34170783	PCP001943	XM_009372138
PCP001958	10,97704375	10,82283369	10,89506779	10,65105169		
PCP001961	11,04143329	10,97680038	10,95177282	10,91163916		
PCP001964	8,684152487	9,041659152	8,876516947	8,788979283	PCP001964	XM_009372174
PCP001992	9,318926875	9,272233193	9,385862401	9,794951903		
PCP002024	10,18156227	10,05799172	10,10852446	10,13528603	PCP002024	XM_018648019
PCP002028	8,731319031	8,861086906	8,977279923	9,195569065	PCP002028	XM_009360660
PCP002037	10,70879938	10,47876962	10,64685568	10,94227257		
PCP002041	10,28386391	10,28038801	10,54592977	10,90538701	PCP002041	XM_009349034
PCP002045	9,218369492	9,468970167	9,442943496	9,650459419		
PCP002046	9,371406396	9,117201525	9,236014192	9,142107057	PCP002046	XM_008379446
PCP002057	11,01889562	11,58104519	11,82323586	12,21401549	PCP002057	XM_008380466
PCP002062	9,336127147	9,557789157	9,47100451	9,444331629	PCP002062	NM_001319253
PCP002094	10,63147721	10,69494112	10,56921954	10,18818083	PCP002094	XM_009364164
PCP002114	9,598666678	9,441554026	9,964340868	10,79224532	PCP002114	XM_018652341
PCP002124	10,10197567	10,22480965	10,37359384	10,48750846	PCP002124	XM_008357034
PCP002127	11,46658634	11,2805794	11,3376219	11,10219226		
PCP002128	9,349458168	9,315149562	9,173252384	9,019590728	PCP002128	XM_008375402
PCP002131	10,28617653	10,18982456	10,17533747	9,934177372		
PCP002132	8,873444113	8,615923847	8,832890014	9,315896762	PCP002132	XM_009371405
PCP002143	9,332417038	9,345782228	9,438791853	9,813252106	PCP002143	XM_008376178
PCP002164	10,61654884	10,52877641	9,960479854	10,82601612	PCP002164	XM_017327118
PCP002219	9,524208672	9,796039609	10,00842862	10,2644426	PCP002219	XM_008378559
PCP002232	12,14932479	11,54737656	11,82602009	11,48196589	PCP002232	XM_009343010
PCP002234	13,94727165	13,71224002	13,88877464	14,23097136	PCP002234	XM_009343007
PCP002236	8,46760555	8,576181982	9,262094845	9,956129273	PCP002236	XM_009359572
PCP002252	9,485165154	9,267559206	9,483150695	9,933203774	PCP002252	XM_009376117
PCP002254	9,944463173	9,364134655	9,599912842	9,996713565	PCP002254	XM_009376113
PCP002261	8,74708524	8,74929961	8,85277917	9,33315535		
PCP002272	10,14168568	10,65284497	10,30947635	11,15080716		

PCP002278	10,81191249	10,21674586	10,64175373	10,99152185	PCP002278	XM_009353149
PCP002288	10,28270621	10,06564501	10,12799432	10,17990909	PCP002288	XM_018646379
PCP002289	9,477758266	9,499845887	9,868313278	9,920352855		
PCP002292	9,541096615	9,605479518	9,548821908	9,483815777	PCP002292	XM_017322807
PCP002293	11,55682694	11,90312868	11,30701292	11,28327939	PCP002293	XM_009357557
PCP002297	10,07992473	9,896831887	10,1980398	10,4101015	PCP002297	XM_018647371
PCP002298	9,017727086	9,392317423	9,803049401	10,0018726	PCP002298	XM_009357564
PCP002299	10,5300844	10,72649918	10,65075559	10,67977037	PCP002299	XM_009357622
PCP002300	9,079484784	9,299963518	9,189009107	9,28077077	PCP002300	XM_018647377
PCP002301	9,356077767	9,223205318	9,254249162	9,242388143		
PCP002317	9,193106413	9,194756854	9,331655958	9,878050913	PCP002317	XM_009356415
PCP002336	10,01541505	9,837107523	10,03937147	10,22520744		
PCP002337	10,38909352	10,17367714	10,18941689	10,33352436	PCP002337	XM_009380960
PCP002348	9,80843332	9,907897125	9,990572019	10,34725594	PCP002348	XM_009365943
PCP002380	10,01587497	10,11243951	10,03617361	10,44224893		
PCP002399	9,950803736	9,886199551	10,0768156	10,6747837		
PCP002422	9,047123912	9,114601277	9,144658243	9,478425839	PCP002422	XM_009361495
PCP002431	8,75043902	8,664162806	8,869347076	9,131008067		
PCP002434	9,796575043	9,937623934	10,03112222	9,975375111	PCP002434	XM_009375274
PCP002435	8,640244936	8,856954575	9,124121312	9,293862446	PCP002435	XM_009375273
PCP002436	11,00492268	11,18920683	11,28847969	11,65672428	PCP002436	XM_008382252
PCP002442	9,064284694	9,031577154	8,963849777	9,001886668	PCP002442	XM_018647522
PCP002444	9,449829585	9,260519709	9,273026268	9,238404739	PCP002444	XM_018643976
PCP002470	10,19352536	10,17741954	10,3376219	10,57016879	PCP002470	XR_001786033
PCP002523	9,290018847	9,403012024	9,557138173	9,929258409		
PCP002525	9,273795599	9,169098221	9,119797095	9,050746552	PCP002525	XM_017332537
PCP002550	8,74708524	8,864186145	8,949330653	9,37286506		
PCP002559	9,381542951	9,45532722	9,667111542	9,956622999	PCP002559	XM_009363619
PCP002576	12,1176431	12,71152287	13,10618177	14,10876946	PCP002576	XM_017336284
PCP002604	9,105044204	9,092757141	9,309089764	9,539798574		
PCP002606	9,138706975	9,192292814	9,05709822	9,165736112	PCP002606	XM_017323217
PCP002612	9,004698008	8,76927586	8,923832563	8,846524657	PCP002612	XM_008360149
PCP002617	9,16824589	9,130132926	9,014941046	8,642665475	PCP002617	XM_009371100
PCP002623	8,798730993	8,797661526	9,159871337	9,49119171	PCP002623	XM_021952634
PCP002626	10,9708249	11,34077922	10,70130646	11,73329926	PCP002626	XM_008356207
PCP002631	9,790071499	9,534633604	9,741466986	8,956143797	PCP002631	XM_009356484
PCP002648	9,705062108	9,651051691	9,671841781	9,543669869	PCP002648	XM_009376744
PCP002649	10,18652284	9,680060579	10,11504365	11,78299821	PCP002649	XM_009376645
PCP002655	10,6070251	10,57080444	10,70418897	10,06249143	PCP002655	XM_009360716
PCP002679	9,158180476	9,236803511	9,21269092	9,173252384		
PCP002682	9,294620749	9,435316054	9,423473125	9,384416825	PCP002682	XM_018652403
PCP002703	9,327170869	9,431142371	9,394462695	9,30986284	PCP002703	XM_009381309
PCP002711	8,548166865	8,72904287	8,929761415	9,281535984	PCP002711	XM_008377342
PCP002734	9,556506055	9,59058705	9,637838439	10,15059775		
PCP002735	11,46471421	11,45857579	11,61409786	11,7965791	PCP002735	XM_009368364
PCP002740	10,46794173	10,87805091	11,04211762	11,25306153	PCP002740	XM_009354706

PCP002743	9,163221961	9,290018847	9,270692451	9,307587905		
PCP002744	11,25404243	11,25128712	11,22661088	11,10721708	PCP002744	XM_018645898
PCP002747	8,832890014	9,021451966	9,216745858	9,744278848	PCP002747	XM_009368056
PCP002748	13,44303029	13,63314623	13,89630118	14,54049133	PCP002748	XM_008367198
PCP002749	9,216745858	9,019590728	9,015888903	8,668884984	PCP002749	XM_008350248
PCP002753	10,10983065	10,07771032	10,24436384	10,52323849		
PCP002767	8,75598914	8,87958325	8,889747307	8,833933692		
PCP002775	9,021451966	9,06520069	9,033423002	9,400879436	PCP002775	XM_009371287
PCP002805	10,68503783	10,85096815	10,86573327	10,78600002	PCP002805	XM_018647438
PCP002811	10,25738784	9,962418883	10,15566576	10,53365094	PCP002811	XM_009355815
PCP002832	10,00516135	10,11547288	10,13057056	10,04439412	PCP002832	XM_018644060
PCP002873	9,71596199	9,998124969	9,762099618	9,614709844	PCP002873	XM_009367639
PCP002891	10,32904509	10,23082505	10,25856603	10,26912668		
PCP002899	9,820705514	9,54239349	9,532024681	9,267559206	PCP002899	XM_009373046
PCP002914	8,530094158	8,653454181	9,06250492	9,410090898	PCP002914	XM_009365576
PCP002915	9,008428622	9,059804103	9,227206781	9,582461918		
PCP002943	12,72785027	12,70735913	12,73096468	13,02750346	PCP002943	XM_009361127
PCP002947	9,72395099	9,701011827	9,716527978	9,682994584		
PCP002971	9,126704473	9,199672345	9,368506462	9,274564521		
PCP002982	9,243959396	9,456703495	10,08613623	11,35773343	PCP002982	XM_009338561
PCP003028	9,416438656	9,481133419	9,281535984	9,296916207	PCP003028	XM_009345875
PCP003035	10,73131903	12,16165784	10,53527538	10,45635442	PCP003035	XM_009341337
PCP003104	10,7153872	10,62662165	10,81003334	10,6183855		
PCP003219	9,700439718	9,82707272	9,872936465	10,38658465		
PCP003291	9,026053072	9,335390355	9,247144259	9,44362737	PCP003291	XM_009362131
PCP003305	10,01029032	9,82124782	9,937623934	9,36047402	PCP003305	XM_009362103
PCP003319	10,57837269	10,78953364	10,88111396	11,72720999	PCP003319	XM_009362086
PCP003346	10,92925841	11,42504277	10,73301532	10,41574177	PCP003346	XM_009362050
PCP003359	8,779161208	8,790087794	8,854868383	9,285402219	PCP003359	XM_009358062
PCP003361	9,574271841	9,769837844	9,84757418	10,18239435	PCP003361	XM_009358066
PCP003367	11,86328418	11,77492292	11,74160602	12,13442632	PCP003367	XM_009358078
PCP003374	9,481133419	9,530737953	9,574271841	10,13528603		
PCP003375	9,240004193	9,076815597	9,017727086	9,340584522	PCP003375	XM_008368572
PCP003387	14,22020221	13,84185915	14,10876946	14,6912348		
PCP003398	9,204571144	9,430452552	9,680060579	9,988684687	PCP003398	XM_009351180
PCP003423	10,3479867	10,36377339	10,31288296	10,3140167	PCP003423	XM_009361193
PCP003445	10,85823749	10,91039539	10,88722062	10,71166697	PCP003445	XM_008369453
PCP003459	10,85383195	10,76928413	10,64655871	10,56160299	PCP003459	XM_018649832
PCP003473	9,840258649	9,965784285	10,00516135	10,29539013		
PCP003486	9,813252106	9,786269628	10,01029032	10,52519778		
PCP003500	12,20447027	11,88226093	11,77971936	11,8917837	PCP003500	XM_009378911
PCP003502	9,445014846	9,447765141	9,560963278	9,444331629	PCP003502	XM_009378915
PCP003527	11,357552	11,8045344	11,75098746	12,39043769		
PCP003531	9,991521846	10,15860969	10,14720492	9,872413248	PCP003531	XM_009336058
PCP003553	8,810571635	8,787902559	8,776992236	9,125852541	PCP003553	XM_008365757
PCP003555	9,175749131	9,2589664	9,367043381	9,82336724	PCP003555	XR_001789798

PCP003566	9,54689446	9,355351096	8,994353437	8,606109055		
PCP003575	11,4238145	11,33650656	11,44329065	11,33892019	PCP003575	XM_018646494
PCP003576	12,7120261	12,96692537	13,01367161	13,86134575	PCP003576	XM_009353623
PCP003579	10,23442228	10,26209485	10,43184231	10,90589088		
PCP003601	9,699867382	9,569229039	9,698131642	9,971543554	PCP003601	XM_009353579
PCP003606	8,742578916	8,925791779	8,947403166	9,188168462	PCP003606	XM_009353563
PCP003612	9,805211831	9,675374899	9,634212071	10,40479032	PCP003612	XM_009353553
PCP003625	9,989635757	9,598666678	9,749316372	9,878556764	PCP003625	XM_008367573
PCP003630	10,58433306	10,28617653	10,17326488	10,22560512	PCP003630	XM_009362382
PCP003645	9,059804103	9,247927513	9,479113318	9,950308015	PCP003645	XM_008390134
PCP003666	10,56478779	10,42975194	10,54528307	10,81698362		
PCP003669	12,23371853	11,96662441	12,01611184	12,25355206	PCP003669	XM_018648263
PCP003693	9,408690635	9,335390355	9,490509963	9,802516365	PCP003693	XM_009361842
PCP003707	9,406566431	9,193944186	8,950322598	8,555854491	PCP003707	XM_009374889
PCP003716	9,046223651	9,142107057	9,140395236	9,495195473	PCP003716	XM_009374879
PCP003736	10,42801464	10,21310422	10,46079375	10,65224447	PCP003736	XM_009368377
PCP003751	10,4307975	10,4604559	10,57111741	10,85330956		
PCP003768	9,934177372	9,026053072	9,487840034	9,534633604		
PCP003769	9,463524373	9,637838439	9,613476402	9,942030594	PCP003769	XM_008355943
PCP003791	9,887220615	9,811101704	9,887220615	9,506466274		
PCP003795	9,677719642	9,852264198	10,24594482	10,69319944	PCP003795	NM_001302283
PCP003800	9,709083813	9,552035018	9,776433032	10,12325492	PCP003800	XM_009351268
PCP003805	9,560332834	9,612260337	9,648051932	9,990572019	PCP003805	XM_009369706
PCP003821	9,993886607	9,884170519	10,14168568	10,37286506		
PCP003827	8,825499451	9,105044204	9,486493158	10,00422047	PCP003827	XM_009365328
PCP003839	12,6713929	12,82017896	12,75697323	13,13939147		
PCP003842	10,10764864	10,11286951	10,10022868	9,892284763		
PCP003856	10,34836301	10,30529798	10,72849027	10,63147721	PCP003856	XM_008368962
PCP003858	9,745959978	9,420655031	9,417852515	9,583082768	PCP003858	XM_009345808
PCP003866	9,125852541	9,339850003	9,49319498	9,330177419	PCP003866	XM_009345818
PCP003892	9,338357556	10,01076585	9,792253453	9,661190213		
PCP003901	11,33966631	11,43810048	11,5372184	11,80372753		
PCP003907	9,115485887	9,014941046	9,102421932	8,910882535	PCP003907	XM_009344263
PCP003908	8,820178962	8,81483878	9,367786025	9,892800824		
PCP003911	10,02974274	10,03799987	10,11981006	9,959031118	PCP003911	XM_009379821
PCP003944	9,235224441	9,294620749	9,316666199	9,317412614	PCP003944	XM_009374254
PCP003953	8,842350343	9,14974712	9,328674927	9,701011827	PCP003953	XM_009374245
PCP003956	8,876516947	8,899870461	8,913876736	9,442259297		
PCP003971	9,040755473	8,95805965	8,735826768	8,911901261	PCP003971	XM_009362309
PCP003973	11,01309233	11,0116955	11,13335417	10,63632554	PCP003973	XM_018648358
PCP003984	12,16165784	11,83302052	11,73414649	11,89847669	PCP003984	XM_008364327
PCP004009	10,48011362	10,49518548	10,74315139	10,91014051	PCP004009	XM_009371078
PCP004024	15,62321479	15,99952288	15,89359377	16,15593825		
PCP004025	9,82124782	9,919861451	10,2772874	10,62632049		
PCP004048	10,42241173	10,57333476	10,77916942	11,42504277		
PCP004101	9,961927137	9,865733271	10,07414146	10,5714398	PCP004101	XM_009370232

PCP004104	9,207819661	8,957102042	9,107661718	9,153121312	PCP004104	XM_017327067
PCP004111	9,323437626	9,283088353	9,490509963	9,965308117	PCP004111	XM_009376325
PCP004144	8,950322598	9,035266491	9,328674927	9,919861451		
PCP004145	10,9734606	11,05889143	11,42888355	11,56684844		
PCP004149	9,687673116	9,599299194	9,775330438	9,192292814	PCP004149	XM_018649493
PCP004150	9,08127038	9,19640541	9,250298418	9,535916864	PCP004150	XM_018649293
PCP004162	9,044394119	9,28000515	9,30833903	9,305309377		
PCP004163	8,824417827	8,893817229	8,968666793	9,40017499	PCP004163	XM_009366640
PCP004167	10,94348917	11,08658078	11,19332206	11,61163809	PCP004167	XM_009356458
PCP004169	11,2039597	11,20660538	11,41362793	11,92184094	PCP004169	XM_018647112
PCP004170	8,804131021	8,960001932	9,020507757	8,921840937	PCP004170	XM_009356461
PCP004181	8,885696373	9,207819661	9,625708843	9,960001932		
PCP004185	10,64985792	10,70735913	10,73893668	11,00702727		
PCP004264	10,5805695	10,61685205	10,55394602	10,58308277		
PCP004269	9,374322251	9,236014192	9,429762402	9,960972093		
PCP004275	12,28559584	12,18384315	12,24822409	12,04484831	PCP004275	XM_008388892
PCP004278	9,922331667	9,880088564	9,95419631	10,37359384		
PCP004293	10,45601552	10,28693889	10,52160044	10,85200664	PCP004293	XM_009357281
PCP004294	10,5685927	10,4498399	10,77148947	11,16113188	PCP004294	XM_009357279
PCP004303	9,806823474	9,791700135	9,954676159	10,36705431	PCP004303	XM_008344061
PCP004306	10	10,0018726	10,01726776	10,03112222	PCP004306	XM_008370319
PCP004326	10,02974274	9,795503976	10,09364204	9,784634846		
PCP004331	10,58088979	10,41609025	9,940063949	10,05165759	PCP004331	XM_009373135
PCP004344	9,938109326	9,942514505	10,13528603	10,45635442	PCP004344	XM_009367583
PCP004347	10,43948809	10,57994757	10,60887391	10,39874369		
PCP004356	9,811101704	9,805211831	10,08480839	10,27844946	PCP004356	XM_008370672
PCP004361	10,06564501	10,55362929	10,82230792	10,84809866		
PCP004374	10,56288157	10,38046107	10,50944843	10,46862404	PCP004374	XR_530954
PCP004384	10,19023211	9,939094317	10,08967557	10,31137369	PCP004384	XM_018651498
PCP004396	13,96662531	13,64903158	13,71799765	14,01468917	PCP004396	XM_009343297
PCP004411	8,798730993	8,79983204	9,224797591	9,883666633	PCP004411	XM_008357532
PCP004423	8,684152487	8,966736149	9,229611955	9,512404684	PCP004423	XM_008390609
PCP004438	11,65999589	11,71051804	11,60208176	11,61025435	PCP004438	XM_018648757
PCP004439	11,19824244	10,64024494	10,666224	9,948849023	PCP004439	XM_009364138
PCP004449	9,026053072	9,038918989	9,071462363	9,706789535		
PCP004480	10,63178639	10,45532722	10,52160044	10,44052662		
PCP004488	10,20457114	10,29768437	10,35351076	10,62021983		
PCP004517	8,696967526	8,536558066	8,836050355	9,30833903		
PCP004531	11,20293802	11,24595076	11,3846057	11,67918537	PCP004531	XM_018646621
PCP004555	10,74118887	10,67330908	10,76680218	10,76293841	PCP004555	XM_009341823
PCP004576	9,766528909	9,870873272	9,991989442	9,825483551	PCP004576	XM_018652414
PCP004591	8,628153873	8,593689902	8,98205238	9,354624059	PCP004591	XM_009359166
PCP004607	9,613476402	10	10,23840474	10,52388535		
PCP004632	9,075024475	8,966736149	9,054387253	9,405843489		
PCP004636	9,214319121	9,160703421	9,340584522	9,591204414	PCP004636	XM_008344483
PCP004662	8,762647796	9,010304306	9,505156537	10,35974956	PCP004662	XM_009364562

PCP004665	10,07190476	10,08347933	10,12454785	10,41574177		
PCP004708	9,176597042	9,186535222	9,049848549	8,935666001		
PCP004739	8,827596761	9,156512913	9,080364533	9,515049296	PCP004739	XM_018643059
PCP004763	9,884170519	9,974887762	10,06339508	10,80197505		
PCP004963	8,957102042	9,229611955	9,14381685	9,477090385	PCP004963	XM_009338924
PCP004966	11,11265453	11,11092042	11,2898287	11,59525748	PCP004966	XM_009338930
PCP004978	10,91637961	10,19188584	10,19967234	9,685326985		
PCP005009	8,568602197	8,677120596	8,86727874	9,735843688	PCP005009	XM_009363387
PCP005028	11,07636803	11,09209641	11,23182118	11,54962586	PCP005028	XM_018648558
PCP005045	9,582461918	9,428360173	9,587458757	9,564778269		
PCP005049	10,98061802	10,70015358	11,13656183	13,58812901		
PCP005072	9,257387843	9,263668263	9,434628228	9,741466986	PCP005072	XM_009341266
PCP005091	9,63481105	9,706789535	9,879077758	10,29920802	PCP005091	XM_009347721
PCP005114	10,92728167	11,26268214	11,29443121	11,97309821		
PCP005120	8,915879379	8,898843202	8,918863237	8,699294818		
PCP005123	11,33706434	11,30187911	11,24812326	11,06339508		
PCP005134	9,332417038	9,365600686	9,408690635	9,208648936	PCP005134	XM_009360346
PCP005137	10,05528244	10,2510918	10,31514956	10,61132917	PCP005137	XM_009360342
PCP005138	10,62844554	10,49585503	10,51274046	10,59588217	PCP005138	XM_009360341
PCP005180	9,808964175	9,725076497	10,13699111	10,06653324	PCP005180	XM_017330903
PCP005181	9,040755473	9,829199465	9,41715631	7,231989133	PCP005181	XM_008388366
PCP005211	9,07325791	9,101529272	9,385862401	9,795503976		
PCP005217	9,957580925	9,825483551	9,956129273	10,2644426	PCP005217	XM_009340859
PCP005227	9,625708843	9,718241739	9,880609006	10,15987134	PCP005227	XM_009340834
PCP005236	9,380828997	9,296159109	9,530074645	9,80843332		
PCP005237	9,393755108	9,333893285	9,162391329	8,956143797		
PCP005243	9,990103964	9,914878405	10,07012094	10,34429591	PCP005243	XM_018646459
PCP005268	11,13013936	10,92431519	10,71996202	10,88874325	PCP005268	XM_009380206
PCP005281	10,17949861	10,24594482	9,987732989	9,874981348	PCP005281	XM_009380222
PCP005299	9,224001674	9,355351096	9,486493158	9,820178962		
PCP005300	8,941546519	8,885696373	9,076815597	8,911901261	PCP005300	XM_009356265
PCP005305	8,606109055	9,089238597	9,008428622	8,70276142	PCP005305	XM_009356260
PCP005306	8,953236133	8,931712601	9,102421932	8,982993575	PCP005306	XM_009356258
PCP005311	9,955649908	10,0918717	10,09671515	10,39695792	PCP005311	XM_009369235
PCP005313	8,707359132	8,890781061	8,887738491	9,466932951	PCP005313	XM_008246533
PCP005320	9,776975792	9,901364723	10,05120894	10,44363773		
PCP005324	10,09583214	10,15186643	10,50845837	10,64295422		
PCP005335	12,38154295	12,31477299	12,38954261	12,26873789	PCP005335	XM_008365068
PCP005372	10,09011242	10,28193003	10,23561937	10,51503944		
PCP005384	10,34096276	10,44639065	10,61225112	10,54754044		
PCP005389	9,534633604	9,840258649	9,800366069	10,04257594	PCP005389	XM_017326810
PCP005400	10,06160071	10,18858885	10,13057056	10,11504365		
PCP005409	9,774243476	9,648663167	9,586839788	9,564778269	PCP005409	XM_017336962
PCP005418	8,505136683	8,74708524	8,843387202	9,285402219		
PCP005432	10,66799854	10,83605036	10,94617524	11,41626974	PCP005432	XM_009357906
PCP005443	10,67683861	10,32530546	10,18570552	10,83605036	PCP005443	XM_009357925

PCP005452	11,17097033	11,09824926	11,20559167	10,93540818		
PCP005453	9,236014192	8,962896005	9,219967107	8,965784285		
PCP005459	10,30415735	10,21916852	10,13741386	9,54173552	PCP005459	XM_009346124
PCP005468	9,691743519	9,972017669	10,24397129	10,53267735	PCP005468	XM_009346132
PCP005508	9,118941073	9,354624059	9,605479518	9,850186838	PCP005508	XM_008388539
PCP005510	9,743706817	9,86727874	9,856939018	9,838683946	PCP005510	XM_009380647
PCP005511	11,26756506	11,23062093	11,09077405	10,90137227	PCP005511	XM_008370901
PCP005517	10,93344723	10,82362996	10,86753358	11,21310422		
PCP005521	10,38729576	10,58840545	10,48984796	10,05799172	PCP005521	XM_018650697
PCP005537	10,43567026	10,52747701	10,52127653	10,86289302	PCP005537	XM_018651278
PCP005545	10,99811792	11,20314608	11,12820704	10,82654849	PCP005545	XM_008343599
PCP005563	9,745959978	10,45840661	10,12110608	9,816983623	PCP005563	XM_009376245
PCP005567	9,479113318	9,529430554	9,709652505	9,596189756	PCP005567	XM_009366615
PCP005571	9,079484784	9,174101763	9,183213552	8,979196518		
PCP005574	8,754887502	8,803065551	8,856954575	9,324923085		
PCP005605	9,835529375	9,63540978	9,775890069	10,09319305		
PCP005616	8,657032562	9,203763987	8,717676423	8,89684702	PCP005616	XM_008350730
PCP005628	9,485165154	9,483150695	9,536577492	9,905883363		
PCP005634	8,978224236	9,002815016	9,174101763	9,627533884	PCP005634	XM_009352933
PCP005638	7,886733033	8,915879379	9,553302899	9,645658432		
PCP005713	8,959045612	8,779161208	9,02699366	9,29156212	PCP005713	XM_009374923
PCP005719	11,95952023	12,10339601	12,10656294	12,36677845		
PCP005769	9,995767151	10,08967557	10,16323454	10,47404051	PCP005769	XM_009364868
PCP005771	9,151447887	9,362667132	8,976334992	8,973467779	PCP005771	XM_009378275
PCP005786	9,539798574	10,17201489	10,15396996	10,51241456	PCP005786	XM_008366971
PCP005790	10,50216549	10,33390446	10,3818998	10,49852015		
PCP005808	9,545601627	9,387306529	9,529430554	9,406566431	PCP005808	XM_009346174
PCP005828	10,31098761	10,67183294	11,00187963	11,4176785		
PCP005843	10,27883273	10,48817153	10,56985561	10,7968508	PCP005843	XM_009374151
PCP005863	11,6251015	11,35259523	11,18673329	11,20701432	PCP005863	XM_009361670
PCP005872	9,453270634	9,369226538	9,532024681	9,248710343		
PCP005877	9,637838439	9,915879379	9,425572598	8,120652609	PCP005877	XM_009348635
PCP005878	10,00936677	9,960001932	9,910897522	9,983948403	PCP005878	XM_009336356
PCP005899	14,13736503	14,7343217	14,35291725	14,50767078	PCP005899	XM_009378211
PCP005934	9,381542951	9,197216693	9,428360173	9,364856916		
PCP005943	9,550746785	9,501837185	9,640841416	9,953716302		
PCP005969	11,69682412	11,35332881	11,43462823	11,59789891	PCP005969	XM_008383021
PCP005981	8,544307635	8,880593701	9,159871337	9,412929682	PCP005981	XM_009350948
PCP005987	10,14805706	10,13057056	10,04984855	10,13143258		
PCP005988	10,11591499	10,30073006	10,30985147	9,898344415		
PCP005990	12,54407817	12,64393058	12,67970001	13,00462955	PCP005990	XM_009348327
PCP005992	8,662953148	8,844454703	8,894817763	9,222384371	PCP005992	XM_009337475
PCP006014	8,696967526	8,857980995	9,135273206	9,439477697	PCP006014	XM_009370463
PCP006015	10,14551188	9,892284763	10,1852843	10,31628153	PCP006015	XM_018650119
PCP006018	9,135273206	9,02699366	9,230428801	9,120678526	PCP006018	XM_009379343
PCP006032	8,764871591	8,918863237	9,055282436	9,40017499		

PCP006041	8,61717357	8,752648252	8,859006685	9,183213552	PCP006041	XM_009369480
PCP006062	9,412209922	9,551381432	9,641455713	9,434628228	PCP006062	XM_008385564
PCP006066	8,791162889	8,812722827	8,900866808	9,501837185		
PCP006087	9,018673116	8,829722735	9,022367813	9,262094845		
PCP006118	10,47505448	10,67624801	10,62113611	11,15987134	PCP006118	XM_009349951
PCP006126	8,988684687	9,068778278	9,123267853	8,968666793	PCP006126	XM_009369674
PCP006158	9,470313155	9,399448837	9,719388821	9,972505988		
PCP006168	8,714245518	8,847589839	9,18157469	10,30035256		
PCP006180	8,803065551	8,831845581	8,798730993	9,44362737	PCP006180	XM_009358356
PCP006195	10,00235092	9,915386449	10,27418011	10,95443625		
PCP006212	9,598666678	9,71539578	10,02329694	10,47269084	PCP006212	XM_009350374
PCP006218	10,38729576	10,00748986	10,31250862	10,72962074	PCP006218	XM_009374162
PCP006236	9,09011242	9,215945486	9,247144259	9,564778269		
PCP006250	11,07124111	10,39732167	10,28693889	10,11677281	PCP006250	XM_008365255
PCP006260	9,678899386	9,764871591	9,896332404	10,23361968		
PCP006304	9,992938336	9,686500527	10,17367714	10,55074679	PCP006304	XM_009375301
PCP006308	9,377926277	9,282323962	9,273795599	10,04347848	PCP006308	XM_009341004
PCP006335	9,275356315	9,262870027	9,271463028	9,673609422	PCP006335	XM_008354757
PCP006343	9,528121969	8,886702554	9,014941046	8,75598914	PCP006343	XM_009352192
PCP006345	10,41609025	10,20783186	10,29499975	9,970580478		
PCP006351	10,76818432	10,67213359	10,91637961	11,1219673	PCP006351	XM_009365035
PCP006451	9,600526229	9,796039609	9,761551232	9,784634846	PCP006451	XM_009359189
PCP006464	9,20537785	8,9905862	9,194756854	8,375039431		
PCP006547	9,764324258	9,769292392	9,72395099	9,393025715		
PCP006553	9,532024681	9,49319498	9,754887502	9,977279923	PCP006553	XM_009367725
PCP006555	8,781359714	8,797661526	9,200481794	9,299208018		
PCP006562	10,41996018	10,39553414	10,57868403	10,26600174	PCP006562	XM_009372569
PCP006572	9,316666199	9,506466274	9,560963278	9,906394584	PCP006572	XM_009372583
PCP006576	9,19640541	9,013099307	9,215120395	9,478425839	PCP006576	XM_009372586
PCP006582	9,326429487	9,44362737	9,594940371	9,829199465	PCP006582	XM_018650610
PCP006597	10,03525274	10,017741	10,08922535	10,02883157	PCP006597	XM_009372629
PCP006627	9,930737338	9,79387963	9,773139207	9,646252679	PCP006627	XM_009374857
PCP006628	9,517019734	9,199672345	9,442943496	9,19063955	PCP006628	XM_009374854
PCP006652	10,20619632	10,26130749	10,24198315	10,60021957	PCP006652	XM_008376204
PCP006653	9,657014692	9,761002638	9,721099189	9,625708843		
PCP006663	9,567956075	9,578693464	10,21513253	10,62509694	PCP006663	XM_008376223
PCP006667	10,02836195	10,10328781	10,18694371	10,49852015	PCP006667	XM_008376236
PCP006670	10,4841482	10,03846636	10,30833903	10,50613895		
PCP006682	10,89153311	10,64626168	10,61868833	10,37685252	PCP006682	XM_008376247
PCP006694	8,402287298	8,63542792	9,008428622	9,423473125	PCP006694	XM_009340927
PCP006705	8,874458871	8,810571635	8,993419626	9,235224441	PCP006705	XM_008394927
PCP006707	9,974887762	9,803049401	9,995300778	10,22560512		
PCP006727	11,35038674	11,32324058	11,39696327	11,66237019	PCP006727	XM_009345153
PCP006732	10,8917837	10,87088098	10,99270471	10,69232781		
PCP006739	9,221587121	9,28077077	9,211888295	9,092757141		
PCP006751	9,195569065	9,317412614	9,105044204	9,09011242	PCP006751	XM_009371189

PCP006752	9,021451966	9,184057088	8,836050355	8,881634033	PCP006752	XM_009371190
PCP006768	9,911391988	10,17158965	10,4101015	10,5552314	PCP006768	XM_009371164
PCP006775	9,599912842	9,719388821	10,32080055	10,71109047	PCP006775	XM_009353309
PCP006777	8,851749041	8,892785649	8,985841937	8,794415866	PCP006777	XM_009353307
PCP006779	10,12799432	10,06383996	10,12541347	9,976807535	PCP006779	XM_009353303
PCP006789	12,95474158	12,82542194	12,99735593	13,3076747	PCP006789	XM_008390936
PCP006795	9,8008999	9,628755372	9,807886174	10,06699059	PCP006795	XR_001952855
PCP006796	9,271463028	9,440869168	9,582461918	9,824958741	PCP006796	XM_009353278
PCP006805	10,74455637	10,75126788	10,98536523	11,50448633	PCP006805	XM_018646423
PCP006809	9,337621902	9,530074645	9,537218401	9,47100451		
PCP006825	9,602383386	9,193106413	9,288473922	9,230428801	PCP006825	XM_008364446
PCP006845	9,068778278	8,957102042	9,126704473	9,596804728		
PCP006847	9,630576566	9,553955615	9,630576566	10,01355996	PCP006847	XM_008359933
PCP006851	10,13228122	10,11200937	10,29155061	10,2463487	PCP006851	XR_669353
PCP006853	10,5533125	10,46352437	10,68999797	10,96698843	PCP006853	XM_009373548
PCP006862	9,269898093	9,141264175	9,353874607	9,254249162	PCP006862	XM_008379862
PCP006863	12,47725232	12,05923291	12,42556473	12,87466059	PCP006863	XM_008379861
PCP006868	9,147204925	9,184875343	9,497193195	9,654045226	PCP006868	XM_008349404
PCP006883	9,856939018	10,03021191	10,15101654	10,60733031	PCP006883	XM_009355137
PCP006884	11,44086917	11,32792871	11,40123686	11,42678889	PCP006884	XM_009355140
PCP006889	9,768184325	9,822299952	9,96337297	10,32680023		
PCP006894	10,8159196	10,98513037	11,17471975	10,89860139	PCP006894	XM_009355153
PCP006903	9,508448461	9,895832748	9,86004717	10,15650027	PCP006903	XM_009355165
PCP006904	9,625708843	9,656424863	9,839723443	10,07547915		
PCP006917	10,23760435	10,25817734	10,38118602	10,19681111	PCP006917	XM_017332383
PCP006932	8,956143797	8,874458871	8,972491628	8,652235507	PCP006932	XM_008373662
PCP006989	9,371406396	9,477758266	9,555873659	9,924322617		
PCP007005	10,2495046	10,10983065	10,24475627	10,18198458	PCP007005	XM_009350243
PCP007018	9,340584522	9,049848549	9,06520069	9,080364533	PCP007018	XM_009355876
PCP007033	8,946409212	8,951284715	9,138706975	8,797661526		
PCP007052	11,5533077	11,64790807	11,72309348	11,59494503	PCP007052	XM_009340241
PCP007060	10,28308835	10,04667385	10,27844946	10,31250862	PCP007060	XM_009340221
PCP007061	10,45463859	10,61746747	10,87369787	10,63269536		
PCP007073	9,790625442	9,839723443	9,975847968	10,32305476	PCP007073	XM_009359388
PCP007079	8,782441329	8,653454181	9,135273206	9,303780748	PCP007079	XM_009337632
PCP007089	9,430452552	9,318926875	9,380828997	9,374322251		
PCP007098	9,584342459	9,74819285	9,654045226	10,27184816		
PCP007145	9,075933681	9,2589664	9,048949988	8,956143797	PCP007145	XR_664419
PCP007174	9,273795599	9,247144259	9,442943496	9,857467877	PCP007174	XM_018646698
PCP007215	8,943481842	8,923832563	9,098900598	9,378663341	PCP007215	XM_018643510
PCP007219	12,31127434	12,68897803	12,86708565	13,24520655	PCP007219	XM_009359442
PCP007235	8,654636029	8,882643049	9,17990909	9,028817757		
PCP007238	11,27068661	11,31288296	11,05979734	10,86598839	PCP007238	XM_008379760
PCP007251	9,648051932	9,673609422	9,655226589	9,669469742	PCP007251	XM_009355817
PCP007256	8,753785022	8,782441329	8,882643049	9,546257836		
PCP007306	12,12422796	12,0780407	12,08657746	12,44914865		

PCP007308	8,526186214	8,666508075	8,866258916	9,567956075	PCP007308	XM_009378237
PCP007329	9,388017285	9,438791853	9,557138173	9,248710343	PCP007329	XM_009374604
PCP007362	10,33464201	10,40194612	10,49852015	10,41609025		
PCP007377	9,224797591	9,282323962	9,73357466	10,43740878	PCP007377	XM_009354932
PCP007383	9,84862294	9,77478706	9,81805485	9,709652505	PCP007383	XM_018649622
PCP007385	9,749316372	9,732455766	9,817511342	9,742595757	PCP007385	XM_009367859
PCP007394	9,773683206	9,402308618	9,667111542	8,821231873	PCP007394	XM_018642796
PCP007404	8,943481842	9,145499145	9,124121312	8,985841937	PCP007404	XM_009361591
PCP007405	10,09364204	10,26053147	10,17201489	10,07146236	PCP007405	XM_009361593
PCP007407	9,016808288	9,058884672	9,160703421	9,4743752	PCP007407	XM_009361598
PCP007417	9,701601036	9,73357466	9,850186838	10,37431138	PCP007417	XM_009337451
PCP007422	11,16679075	11,06922821	11,07859783	11,40939094	PCP007422	XM_009337450
PCP007430	9,994353437	9,962896005	9,869855987	10,22118833		
PCP007459	8,61717357	8,845490051	8,875503635	9,253445696	PCP007459	XM_009370214
PCP007484	9,693486957	9,54689446	9,730181399	10,01262454		
PCP007502	10,51471405	10,53883882	10,49252419	10,48683502	PCP007502	XM_009353491
PCP007514	8,970594857	8,965784285	9,092757141	8,854868383	PCP007514	XM_018647897
PCP007530	11,55458885	11,70836401	11,59463719	11,72195361	PCP007530	XM_008385520
PCP007555	10,28886607	10,01076585	10,23442228	10,58120058	PCP007555	XM_009369054
PCP007557	8,857980995	9,045295522	9,300741498	9,688250309	PCP007557	XM_008361573
PCP007582	10,91214086	11,0577684	11,02813399	11,53251665		
PCP007590	9,555873659	9,438105682	9,696967526	10,09759763		
PCP007604	8,961941603	8,568602197	9,21916852	9,607940556	PCP007604	XM_008351562
PCP007642	10,25384748	10,37431138	10,50216549	10,84549005	PCP007642	XM_008362479
PCP007654	9,925302224	9,889245365	10,00468396	9,901364723		
PCP007658	9,068778278	9,137836494	9,531381461	9,861086906	PCP007658	XM_008356085
PCP007669	9,402308618	9,460803985	9,904890476	10,39695792	PCP007669	XM_009379019
PCP007676	9,850186838	9,765982204	9,907386437	10,14082977	PCP007676	XM_009359602
PCP007677	12,00796282	11,91251514	11,92518352	11,74062404		
PCP007681	10,58683979	9,815383296	10,14507876	10,18404469	PCP007681	XM_009380262
PCP007714	8,978224236	8,923832563	8,811631578	8,85277917		
PCP007715	9,716527978	9,839203788	9,918371326	10,26600174	PCP007715	XM_009372514
PCP007718	9,296159109	8,969616759	9,416438656	8,689997971	PCP007718	XM_008369462
PCP007734	9,910897522	9,899356923	10,00562455	9,87036472	PCP007734	XM_008387377
PCP007737	10,20252792	10,22680954	10,36814629	10,61440618	PCP007737	XM_008341105
PCP007740	9,019590728	8,927777962	8,922822231	8,613494819	PCP007740	XM_009349550
PCP007754	9,535275377	9,126704473	9,047123912	8,715378619	PCP007754	XM_009336728
PCP007757	9,089238597	9,243959396	9,566681988	9,925302224	PCP007757	XM_018648832
PCP007805	10,45498809	10,27612441	10,41397693	10,21634573	PCP007805	XM_009348277
PCP007806	10,03983752	10,1476247	10,11331242	10,65612986	PCP007806	XM_008375617
PCP007816	9,192292814	9,49251418	9,77478706	10,30947635	PCP007816	XM_008384221
PCP007829	11,19680496	11,04553444	11,35296482	11,25306153	PCP007829	XM_008395375
PCP007897	9,220789431	9,460803985	9,391608782	9,38945926	PCP007897	XM_009368334
PCP007902	11,09341756	11,08214904	11,03891899	11,35718359	PCP007902	XM_009355935
PCP007936	9,050746552	9,10938668	9,140395236	9,080364533		
PCP007952	12,49460562	12,43297767	12,2533541	12,52102865		

PCP008057	10,20416762	9,835529375	10,26678654	10,84287672	PCP008057	XM_018649862
PCP008105	9,147204925	9,558420713	9,503169818	10,27146303	PCP008105	XM_009341766
PCP008150	9,911886285	9,779719355	10,1628067	9,594940371	PCP008150	XM_009372373
PCP008163	8,517000043	8,803065551	9,131856961	9,582461918	PCP008163	XM_009353105
PCP008186	9,359024737	9,225617167	9,097163045	9,364856916		
PCP008209	9,533972085	9,315896762	9,647458426	9,692911856	PCP008209	XM_009338484
PCP008262	9,654636029	10,00562455	10,17492568	11,41503926		
PCP008269	9,468277836	9,891282469	9,814854798	9,375756255	PCP008269	XM_009363972
PCP008298	11,9854862	11,98144955	12,00199571	11,39981196	PCP008298	XM_017336967
PCP008327	10,38478375	10,68035952	10,86005493	10,80654962	PCP008327	XM_008391641
PCP008339	9,697541014	9,863675229	9,63481105	9,571752644		
PCP008341	10,10852446	10,1476247	10,37576711	10,77093639	PCP008341	XM_018644469
PCP008351	8,97441459	9,19640541	9,421370591	9,795503976		
PCP008365	11,15713847	11,4807902	11,49951208	11,38118061	PCP008365	XM_009356600
PCP008367	9,889245365	9,520952554	9,422064766	9,55522181		
PCP008387	8,929761415	9,148044344	9,382256552	9,566054038		
PCP008388	8,952246191	9,121533517	9,347244868	9,720534991		
PCP008407	9,093628836	9,117201525	9,307587905	10,02928723	PCP008407	XM_009335859
PCP008418	12,16312445	11,84862294	11,60625251	11,62631593	PCP008418	XM_009335836
PCP008419	11,41433112	11,71853288	11,83486604	11,12649154	PCP008419	XM_009335835
PCP008427	8,77807713	8,705044823	8,709669735	9,167418146	PCP008427	XM_009335818
PCP008430	9,584342459	9,627533884	9,52028473	9,652253436	PCP008430	XM_009335813
PCP008442	9,359024737	9,342074668	9,544326957	9,814854798		
PCP008444	9,396604781	9,589332801	9,682994584	10,43288635	PCP008444	XM_009368900
PCP008445	9,4743752	9,693486957	9,756005825	10,29270124	PCP008445	XM_009368895
PCP008446	9,816455711	9,919861451	9,975375111	10,49485558	PCP008446	XM_008352737
PCP008463	9,142107057	9,146365017	9,274564521	9,095397023	PCP008463	XM_008345008
PCP008469	9,841831634	9,857980995	9,869331651	10,23401505	PCP008469	XM_008388140
PCP008471	10,86882255	10,8963324	10,75098327	10,71910642		
PCP008478	9,752096261	9,689997971	9,878050913	10,11547288	PCP008478	XM_009336880
PCP008497	13,45549804	13,04239981	12,91849059	13,24545318	PCP008497	XR_531926
PCP008500	10,10895563	10,19023211	10,18198458	10,12498719	PCP008500	XM_009354827
PCP008509	9,230428801	9,593708574	9,405141463	9,487176804	PCP008509	XM_008361698
PCP008520	11,09341756	11,05596023	11,08236862	10,98299357	PCP008520	XM_009348551
PCP008526	9,449148645	9,435316054	9,621447519	9,545601627	PCP008526	XM_017326472
PCP008545	8,821231873	8,90288758	8,930737338	8,850718177	PCP008545	XM_018643209
PCP008554	11,01541505	10,86495992	10,90989308	10,68650053	PCP008554	XM_009339387
PCP008555	8,87857209	8,649866903	8,979196518	9,093628836		
PCP008557	10,55586408	10,51503944	10,46283965	10,13400269	PCP008557	XR_664628
PCP008561	9,819652219	9,215120395	9,772595002	10,02513956		
PCP008585	10,47978026	10,40868002	10,36084708	10,675666	PCP008585	XM_009360984
PCP008586	9,08833774	9,297672907	9,824958741	10,41996018	PCP008586	XR_667585
PCP008598	9,815383296	9,648663167	9,749869427	9,727920455	PCP008598	XM_009368121
PCP008612	10,67801027	10,73273557	10,9945868	10,86083092	PCP008612	XM_009358964
PCP008618	8,659389441	8,861086906	8,927777962	9,513727596	PCP008618	XM_009344741
PCP008658	8,899870461	8,95419631	9,08127038	9,413627929	PCP008658	XM_008376727

PCP008660	10,27029533	10,42486961	10,52649924	10,8262863		
PCP008689	9,569229039	9,765418716	9,928770029	10,17700835	PCP008689	XM_008350869
PCP008691	10,74230944	11,00889777	10,95564991	11,2636624	PCP008691	XR_529409
PCP008693	8,826548487	8,813781191	9,144658243	9,356826076		
PCP008723	10,6694786	10,64985792	10,49818105	10,6794801	PCP008723	XM_009363642
PCP008724	11,10721708	11,37250053	11,14784089	11,49502054	PCP008724	XM_018648642
PCP008771	9,687673116	9,605479518	9,692911856	10,12282799	PCP008771	XM_009357649
PCP008797	9,14974712	9	9,208648936	10,19229281	PCP008797	XM_009338219
PCP008799	9,79387963	10,27068077	10,01495499	10,25148241		
PCP008809	12,52307183	12,72245108	12,73781138	13,06944977		
PCP008814	9,273026268	9,304533858	9,361943774	9,716527978	PCP008814	XM_009361027
PCP008822	9,082149041	9,051671182	9,00750388	8,744833837		
PCP008824	11,54592977	11,3779317	11,58072965	11,85005788		
PCP008827	9,878050913	9,690574236	9,705632387	9,658211483	PCP008827	XM_018648356
PCP008832	9,442259297	9,553955615	9,569229039	9,880088564		
PCP008837	10,49485558	10,38046107	10,43671154	10,48449062	PCP008837	XM_008369969
PCP008855	9,70333261	9,846540327	9,820178962	9,84757418	PCP008855	XM_017333417
PCP008880	9,775330438	9,77478706	10,06339508	10,33650656		
PCP008903	11,5849625	11,56890615	11,68854319	11,51733968	PCP008903	XM_008368496
PCP008924	10,33203655	10,54754044	10,63390341	10,45909345		
PCP008925	11,10307139	11,3192939	11,37159698	11,2879028	PCP008925	XM_009357300
PCP008929	12,19865069	12,39169201	12,40585146	12,81097322		
PCP008932	8,787902559	8,887738491	8,919846558	9,324923085	PCP008932	XM_008343798
PCP008934	8,996247498	8,849655303	9,034331283	9,484480553	PCP008934	XM_008343794
PCP008953	9,014941046	8,730198386	8,949330653	8,825499451	PCP008953	XM_009346689
PCP008964	11,95080009	11,5849625	11,61240779	11,51191569	PCP008964	XM_018644326
PCP008987	10,46590306	10,11243951	10,19844504	10,4076183	PCP008987	XM_009361162
PCP008993	9,967701794	10,19023211	10,28308835	11,33389887	PCP008993	XM_009361158
PCP009003	9,944463173	10,30035256	10,43671154	10,93565864	PCP009003	XM_009345767
PCP009020	9,615942233	9,585582276	9,563520437	9,553302899		
PCP009028	9,785174528	10,01495499	9,991521846	9,964817355		
PCP009037	9,38945926	9,240004193	9,422064766	9,997179481	PCP009037	XM_009375743
PCP009082	8,942514505	9,085684897	9,093628836	9,442259297	PCP009082	XM_008378839
PCP009104	10,37612539	10,25068924	10,43983088	10,33016621	PCP009104	XM_009365977
PCP009135	10,08170978	9,992938336	10,12325492	10,37756845	PCP009135	XM_009343510
PCP009155	10,74090227	10,70793713	10,77313921	11,84326549	PCP009155	XM_009346208
PCP009170	9,730181399	9,286950435	9,872936465	9,631777296	PCP009170	XM_009378021
PCP009173	10,24555271	10,26717293	10,34651373	10,63269536		
PCP009196	9,743706817	9,637838439	9,640841416	9,711374448		
PCP009202	9,632995197	9,646864677	9,535916864	9,866768918	PCP009202	XM_008378897
PCP009225	11,64385619	11,51208365	11,65150022	11,90438249	PCP009225	XM_008387923
PCP009258	9,701011827	9,497851837	9,606090543	9,62935662	PCP009258	XM_009340087
PCP009259	8,868297843	8,843387202	9,014941046	9,480446902		
PCP009274	9,721099189	9,480446902	9,631777296	9,599299194		
PCP009283	8,842350343	8,929761415	9,006550496	9,503825738		
PCP009286	9,024225474	8,893817229	9,353146825	9,106772296	PCP009286	XM_009351984

PCP009305	9,156512913	9,213517401	9,29156212	9,18157469	PCP009305	XM_009380834
PCP009349	9,354624059	9,47370575	9,251884747	8,724513853	PCP009349	XM_009356382
PCP009388	8,98205238	9,345028167	9,301496195	9,892284763		
PCP009415	9,50118036	9,476401941	9,353146825	9,719388821		
PCP009440	9,709652505	10,0588982	10,07324452	10,53398182	PCP009440	XM_009352267
PCP009477	8,752648252	9,160703421	9,312882955	9,876516947	PCP009477	XM_008349805
PCP009490	11,14847658	11,02583167	11,16050175	11,49951208		
PCP009557	9,372146798	9,326429487	9,696967526	11,65269711	PCP009557	XM_008392473
PCP009572	11,31250295	11,24693651	11,26131337	11,19167615	PCP009572	XM_009341313
PCP009577	11,27573459	11,15312765	11,1243346	11,09781487		
PCP009586	10,02004932	9,99859043	10,08391805	9,957102042	PCP009586	XM_009355812
PCP009611	9,132705355	9,407267764	9,350209917	10,10852446	PCP009611	XM_009341766
PCP009695	9,413627929	9,477758266	9,517019734	9,917864332	PCP009695	XM_018647494
PCP009714	10,31967212	9,662970945	9,444331629	9,606719814	PCP009714	XM_009358472
PCP009723	8,686500527	8,835008208	8,936637939	9,348728154		
PCP009731	9,730181399	9,790625442	9,906890596	10,1670041	PCP009731	XM_009358725
PCP009734	8,743723645	8,745943176	9,189009107	9,299208018	PCP009734	XM_009358775
PCP009753	9,232828614	9,578693464	9,744278848	9,842350343	PCP009753	XM_009358994
PCP009768	10,1050311	10,31288296	10,38154295	10,80332392	PCP009768	XM_009359139
PCP009774	8,72904287	8,68183575	9,240004193	9,726218159		
PCP009780	10,99270471	11,06474276	11,15692367	11,63057202		
PCP009783	9,685922633	9,938594555	10,01634867	10,1438041	PCP009783	XM_009359318
PCP009791	11,51455133	11,26170122	11,02744299	10,8494051	PCP009791	XM_009359401
PCP009806	8,517000043	8,636624621	9,192292814	9,595574522	PCP009806	XM_009349275
PCP009825	9,90287251	9,993405473	10,03479896	10,02697983	PCP009825	XM_009346261
PCP009830	9,882138629	9,874474241	9,930737338	9,903384798	PCP009830	XM_018644781
PCP009840	14,48228298	14,34948858	14,32166954	14,65228481		
PCP009848	10,52388535	9,453270634	10,01634867	9,594940371	PCP009848	XM_009343287
PCP009851	9,620824641	9,626311362	9,8159116	9,747639151		
PCP009862	9,350209917	9,479113318	9,651051691	10,04667385	PCP009862	XM_009341453
PCP009869	9,796039609	9,783538504	9,993886607	10,67683861		
PCP009881	9,332417038	9,356826076	9,380828997	9,268354853		
PCP009899	10,2179577	10,21391832	10,41890673	10,87523483		
PCP009904	9,744833837	9,510427928	9,630576566	9,609788199		
PCP009916	9,036173613	9,223205318	9,147204925	9,536577492		
PCP009926	10,75822321	10,70936819	10,94227257	11,25365251		
PCP009933	9,564778269	9,574271841	9,557789157	9,369968059		
PCP009934	10,69435789	10,42206477	10,67065625	10,55810497	PCP009934	XM_009371782
PCP009935	13,00369358	12,83302249	13,13837901	13,42381581	PCP009935	XM_009372281
PCP009938	9,703903573	9,176597042	9,246336825	8,67479253	PCP009938	XR_664329
PCP009942	10,64475759	10,72934033	10,87446656	11,16259903	PCP009942	XM_009372264
PCP009957	10,70505347	11,17659081	11,75502525	12,49193575	PCP009957	XR_001790491
PCP009972	9,696967526	9,614709844	9,601157931	9,382991408	PCP009972	XM_017333450
PCP009976	9,387306529	9,655834793	9,751544059	10,16951167	PCP009976	XR_670079
PCP009992	8,781359714	8,788979283	8,952246191	9,60856902		
PCP010017	8,888743249	8,703903573	8,931712601	9,319672121	PCP010017	XM_009364996

PCP010026	8,568602197	8,73809226	8,76927586	9,60856902		
PCP010032	14,31743593	14,12096682	14,24916309	14,27736006	PCP010032	XM_009365012
PCP010045	9,54303182	9,666525827	9,691167722	10,1050311		
PCP010048	11,26561505	11,0609188	11,2137118	11,58511747	PCP010048	XM_008376508
PCP010053	12,60184738	13,11406765	12,95110255	13,53729971	PCP010053	NM_001302280
PCP010062	13,38113734	13,31788317	13,47598669	13,80318969	PCP010062	XM_009364511
PCP010072	9,392317423	8,8008999	9,098900598	8,827596761	PCP010072	XM_009364498
PCP010075	9,327170869	8,736976865	8,775873612	8,795487741	PCP010075	XM_008364881
PCP010104	9,002815016	8,790087794	9,094526396	9,217545787		
PCP010106	9,621447519	9,477758266	9,569229039	9,945443836	PCP010106	XM_009357457
PCP010111	9,407968756	8,860062694	9,321184013	9,366322214	PCP010111	XM_009357468
PCP010112	11,54448152	11,41398221	11,34207467	11,09890718	PCP010112	XM_009357471
PCP010119	8,98205238	8,788979283	8,835008208	8,903881846	PCP010119	XM_009357480
PCP010122	10,01726776	9,730181399	9,785174528	9,855911856	PCP010122	XM_018647362
PCP010123	9,447765141	9,119797095	9,388727692	9,548821908		
PCP010130	10,83130724	10,53267735	11,07547915	11,21533278	PCP010130	XM_018645143
PCP010141	8,951284715	8,937609223	9,186535222	9,459431619	PCP010141	XM_009377347
PCP010154	11,25108588	11,44931891	11,88988417	12,59502432	PCP010154	XM_008358773
PCP010163	9,884170519	9,880088564	9,888743249	10,32904509	PCP010163	XM_018651406
PCP010181	9,499188155	9,468277836	9,524208672	9,479113318	PCP010181	XM_018651401
PCP010198	11,66637048	11,47116713	11,55810019	11,36996261		
PCP010201	9,201315296	9,011227255	9,182394353	9,090985714	PCP010201	XM_009374117
PCP010207	8,731319031	8,820178962	9,01402047	9,256609709		
PCP010214	10,57962707	10,42870562	10,4015942	10,09407769	PCP010214	XR_001954170
PCP010223	10,2387988	10,24237623	10,26717293	10,59121377	PCP010223	XM_009373519
PCP010229	10,03617361	10,41890673	10,52519778	10,37250597	PCP010229	XM_017331298
PCP010235	11,74076316	11,74202727	11,83328544	11,82004729		
PCP010246	9,752664976	9,68707819	9,897346324	10,09978174		
PCP010249	10,41080112	10,32830467	10,31741261	10,35019886	PCP010249	XM_009376153
PCP010262	10,18031945	10,14507876	10,24039782	10,70102049	PCP010262	XM_009376124
PCP010264	10,15101654	9,754336368	9,689421477	8,925791779	PCP010264	XM_009376132
PCP010294	10,25817734	10,15945511	10,17825402	10,05256805		
PCP010307	9,722244002	9,742023058	9,675957033	10,25266543	PCP010307	XM_009356185
PCP010322	9,996713565	10,39732167	10,34355217	11,41292439	PCP010322	XM_009376403
PCP010327	10,07592031	10,15355203	10,11547288	10,45704223	PCP010327	XM_009376398
PCP010336	9,275356315	9,033423002	9,318926875	9,640244936	PCP010336	XM_009351812
PCP010352	10,54561128	10,55394602	10,57270023	11,07503116	PCP010352	XM_009371419
PCP010355	10,9398216	10,92901424	10,99576715	10,88289519		
PCP010361	8,796591265	9,00750388	9,147204925	8,946409212		
PCP010389	9,628755372	9,934678666	10,31514956	10,87267488	PCP010389	XM_009357045
PCP010401	9,559702114	9,889245365	9,772595002	10,27961058		
PCP010440	10,43045255	10,64745843	10,68445935	10,99010396	PCP010440	XM_008378913
PCP010445	9,489185654	8,903881846	9,356077767	9,318158642	PCP010445	XM_008378915
PCP010454	9,224797591	8,899870461	9,157346935	9,626931876	PCP010454	XM_008378919
PCP010455	10,61010206	9,789533645	9,316666199	8,336863563	PCP010455	XM_008389727
PCP010491	10,47201045	10,65939836	10,76901132	10,85927853		

PCP010499	8,713111526	8,782441329	9,130132926	9,374322251		
PCP010518	10,39767463	10,30720081	10,18198458	9,501837185	PCP010518	XM_009354312
PCP010520	13,75766815	12,65702139	12,50787638	12,73928908	PCP010520	XM_009341200
PCP010537	10,68970975	10,65582585	10,76570049	10,59525748	PCP010537	XM_008374730
PCP010539	9,625708843	9,19063955	8,751544059	9,340584522		
PCP010553	10,22319325	9,525520809	10,34132979	10,26405548	PCP010553	XM_009378648
PCP010554	10,16197583	10,06160071	10,09364204	9,88519302	PCP010554	XM_009378639
PCP010557	8,518338423	8,574896225	8,945443836	9,300741498	PCP010557	XM_009358699
PCP010561	9,376494429	9,306813609	9,195569065	8,830768706	PCP010561	XM_009358693
PCP010565	9,239192751	8,552015799	8,485145023	10,42731284	PCP010565	XM_009361563
PCP010568	12,75021917	12,57175264	12,6956633	13,03451184	PCP010568	XM_009361561
PCP010570	9,412929682	9,5980525	9,895832748	10,53818893	PCP010570	XM_009361558
PCP010573	9,768184325	9,69406183	9,84757418	10,16323454	PCP010573	XM_009361551
PCP010588	8,999069838	9,017727086	8,890781061	8,787902559	PCP010588	XM_009340015
PCP010594	12,91257689	12,75196241	12,8726114	13,24208292	PCP010594	XM_009359969
PCP010603	10,6744836	10,68064962	10,68123841	10,67213359	PCP010603	XM_008383375
PCP010607	10,29309224	10,39588754	10,49452562	10,437055	PCP010607	XM_008362977
PCP010620	9,208648936	9,176597042	9,221587121	9,142949447	PCP010620	XM_018650439
PCP010633	9,082149041	9,003742767	9,152284842	9,44362737	PCP010633	XM_009359938
PCP010640	8,948367232	9,309089764	9,173252384	9,266786541	PCP010640	XM_009365016
PCP010646	9,278449458	9,285402219	9,315896762	9,692911856		
PCP010647	9,566681988	9,475733431	9,505156537	9,374322251		
PCP010655	10,38046107	10,20539007	10,35424938	10,24990749	PCP010655	XM_008356805
PCP010690	10,00842862	9,72395099	9,557138173	8,907882108	PCP010690	XM_009380995
PCP010694	9,669469742	9,526831754	9,571126898	9,140395236	PCP010694	XM_017322967
PCP010702	10,05483491	9,975375111	10,09319305	10,37612539	PCP010702	XR_001951751
PCP010718	8,868297843	8,822315887	9,010304306	9,8339495	PCP010718	XM_009347492
PCP010728	9,779177627	9,733015322	9,930737338	9,872413248		
PCP010736	9,776433032	9,8008999	9,781359714	9,654045226	PCP010736	XM_009378201
PCP010773	10,42661071	10,28617653	10,36449583	10,13228122	PCP010773	XM_018645865
PCP010800	8,247927513	8,781359714	9,50249371	9,842868866		
PCP010829	9,350209917	9,693486957	9,73809226	9,963864223	PCP010829	XM_018645484
PCP010846	9,184057088	9,315896762	9,087462841	8,806291831	PCP010846	XM_009339261
PCP010864	11,35920049	11,28886607	11,29462075	11,35277728	PCP010864	XM_009351048
PCP010869	10,03112222	10,10022868	10,28308835	10,77616158	PCP010869	XM_008341640
PCP010874	8,783554874	8,80196697	8,807354922	9,187352073		
PCP010879	10,97393409	10,90288004	11,02698675	11,38711801	PCP010879	XM_008346333
PCP010886	10,15355203	10,14040802	9,8159116	9,916372141	PCP010886	XM_009345141
PCP010900	7,602364826	8,700439718	9,846540327	10,40939094	PCP010900	XM_009348130
PCP010907	9,917864332	10,11591499	10,3786525	10,86521517	PCP010907	XM_004302135
PCP010922	8,78571401	8,916864735	9,161560218	9,438105682	PCP010922	XM_009352223
PCP010925	9,505156537	9,575539247	9,794415866	10,00235092	PCP010925	XM_009340712
PCP010926	9,224797591	9,262870027	9,326429487	9,842350343	PCP010926	XM_009340714
PCP010944	10,59183086	10,59276535	10,70793713	10,64175373	PCP010944	XM_018643563
PCP010950	8,809510912	9,022367813	9,10938668	8,890781061	PCP010950	XM_009349581
PCP010955	8,649866903	9,795503976	10,59835962	9,863148641	PCP010955	XM_009344090

PCP010964	10,22118833	10,04757383	10,21391832	11,01518504	PCP010964	XM_009344697
PCP010992	9,826024064	9,979182225	10,0274499	9,896332404	PCP010992	XM_009368277
PCP010997	9,712527	9,621447519	9,792790294	10,07503785	PCP010997	XM_009378729
PCP010998	10,21228966	10,09143539	10,52779952	11,14402087	PCP010998	XM_009378713
PCP010999	10,14465824	10,14974712	10,21714588	10,65165269	PCP010999	XM_009378690
PCP011023	9,449829585	9,688250309	9,955170382	10,03754695		
PCP011054	9,356077767	9,415741768	9,043492153	9,078604498	PCP011054	XM_018651149
PCP011155	8,741466986	9,402308618	9,883147293	10,58527242	PCP011155	XM_009369313
PCP011348	9,754887502	9,988216019	10,01168851	10,10328781	PCP011348	XM_009370567
PCP011351	9,327934318	9,222384371	9,357552005	10,30301583		
PCP011354	9,130132926	9,078604498	9,22881869	9,642051693		
PCP011355	8,928755227	8,999069838	9,1807297	9,6794801	PCP011355	XM_018650136
PCP011376	8,79983204	8,687655621	8,745943176	9,108524457	PCP011376	XM_008363009
PCP011427	11,66991701	11,68766874	11,88747577	11,81578354	PCP011427	XM_009368210
PCP011432	9,900866808	9,682994584	9,830784548	9,852264198	PCP011432	XM_009368201
PCP011441	9,520952554	9,6794801	9,670656249	9,92135004	PCP011441	XM_018649648
PCP011453	10,72451385	10,72649918	10,87728413	11,16595589	PCP011453	XM_009355236
PCP011457	10,68766437	10,68533575	10,75014588	10,66681871	PCP011457	XM_009355230
PCP011464	9,436711542	9,359024737	9,41715631	9,971543554	PCP011464	XM_009341250
PCP011482	10,92703717	10,60517391	10,74819285	11,07458973	PCP011482	XM_009355202
PCP011491	9,37721053	8,639051236	8,759322309	8,648645193	PCP011491	XM_009355179
PCP011495	9,626931876	9,422758607	9,241578042	9,460803985	PCP011495	XR_665238
PCP011497	10,38658465	10,29001885	10,41644921	10,33203655	PCP011497	XM_018644223
PCP011498	9,564778269	9,606719814	9,750991645	10,01216358		
PCP011520	11,15587443	11,17013162	11,12520034	10,97799537	PCP011520	XM_018650376
PCP011541	10,25345751	10,31212284	10,47302584	10,79711839		
PCP011555	9,985372344	10,21593336	9,171602161	8,849655303	PCP011555	XM_008355283
PCP011562	11,19926131	11,14911199	11,34466763	11,5370582	PCP011562	XM_008381243
PCP011569	10,15734694	10,27961058	10,72366094	10,3376219		
PCP011577	9,380093039	9,516350087	9,695228291	9,991054099	PCP011577	XM_009380033
PCP011582	9,925302224	10,39802751	10,19311874	10,35608877	PCP011582	XM_009380038
PCP011586	10,24237623	9,99859043	10,17700835	10,39196315	PCP011586	XM_009380043
PCP011593	10,19023211	9,616548844	9,889245365	9,306813609	PCP011593	XM_009380052
PCP011602	9,850186838	9,889762515	9,92381771	9,761002638	PCP011602	XM_018650184
PCP011614	8,884689491	9,045295522	9,162391329	9,361220052		
PCP011643	9,932214752	9,517669388	9,533329732	9,919354981	PCP011643	XM_008341786
PCP011658	11,48549224	11,61117699	11,88557055	12,49352275	PCP011658	XM_008235525
PCP011669	12,68109124	12,72380384	12,47784173	12,62586178	PCP011669	XM_009342585
PCP011687	10,35314683	10,1050311	10,35681507	10,71338651	PCP011687	XM_018647245
PCP011688	9,404439095	9,299963518	9,382256552	9,373582965	PCP011688	XM_008365865
PCP011708	10,40123153	10,84025078	10,71681946	10,98631849		
PCP011720	10,43184231	10,39196315	10,52290515	10,87190524	PCP011720	XM_008353703
PCP011722	10,31816994	10,34207467	10,41503398	11,06429143		
PCP011731	9,686500527	9,900368721	10,1711768	10,30985147		
PCP011760	9,688250309	9,147204925	8,947403166	8,321928095	PCP011760	XM_009350263
PCP011772	10,02052165	9,946906274	10,08524671	10,46522967	PCP011772	XM_018643271

PCP011774	12,06933899	11,81204491	11,80829253	12,14136318		
PCP011789	11,11482899	10,98820892	11,08547246	11,36850646		
PCP011829	12,62060245	12,73428623	12,62472008	13,01883826	PCP011829	XM_008343259
PCP011836	9,908887946	9,580578921	9,610415859	9,736959959	PCP011836	XM_009380193
PCP011843	8,833933692	8,833933692	9,255807556	9,599912842	PCP011843	XM_018650019
PCP011844	8,776992236	8,899870461	9,306813609	9,779719355	PCP011844	XM_009370095
PCP011856	10,29194193	10,14296221	10,27224486	10,17658458		
PCP011865	8,963849777	8,853839746	8,876516947	8,733591606	PCP011865	XM_008360466
PCP011875	10,22118833	10,18239435	10,47573343	10,57175264	PCP011875	XM_009344381
PCP011907	8,383704292	8,936637939	9,171602161	10,12498719	PCP011907	XM_009338906
PCP011910	10,23082505	10,17783061	10,34836301	10,82708066	PCP011910	XM_009347671
PCP011926	9,622655867	9,569229039	9,304533858	9,14974712	PCP011926	XM_009347911
PCP011954	8,61717357	8,795487741	9,260519709	9,568583198	PCP011954	XR_001953335
PCP011960	9,925302224	9,970580478	10,06877828	10,42731284	PCP011960	XM_008374653
PCP011965	9,327170869	9,544964433	9,6794801	9,648663167	PCP011965	XM_009348355
PCP011996	13,3568192	13,53462753	13,25699441	13,62544288	PCP011996	XM_008363560
PCP012003	9,879077758	10,13356609	10,02606691	10,1309952	PCP012003	XM_009359881
PCP012015	10,04165915	10,14974712	10,16741815	10,16071602	PCP012015	XM_009351619
PCP012016	9,536577492	9,797661526	9,851233701	10,14932797		
PCP012023	9,343563277	9,30986284	9,292321633	9,090985714	PCP012023	XM_009336154
PCP012037	9,364856916	8,9905862	8,82336724	9,503169818	PCP012037	XM_009358036
PCP012039	10,03296865	9,643260955	9,350939182	9,312882955	PCP012039	XM_009357980
PCP012050	8,920858975	9,103287808	9,262094845	9,450530824	PCP012050	XM_009369208
PCP012051	11,03020501	10,6891244	11,09187831	11,67537049	PCP012051	XM_018649862
PCP012059	9,419264989	9,589332801	9,738649634	9,937623934	PCP012059	XM_009369386
PCP012068	12,61501574	12,79894358	12,25463597	12,01518155		
PCP012077	9,235224441	9,37721053	9,388017285	9,759888183		
PCP012102	10,50878516	10,4033636	10,53365094	10,42381975		
PCP012127	10,84313591	10,84077792	10,91761823	11,32455749	PCP012127	XM_009342245
PCP012145	10,63026713	10,44086917	10,55937709	10,56510208	PCP012145	XM_009371293
PCP012146	9,957580925	9,75710661	9,750991645	10,203348	PCP012146	XM_008386527
PCP012148	10,65224447	10,68445935	10,43428419	10,22158712	PCP012148	XM_009371299
PCP012149	9,854354156	9,736401931	9,801983132	9,699277464		
PCP012166	9,505156537	9,927289081	10,40266036	10,56795608	PCP012166	XM_009339019
PCP012168	9,349458168	9,318926875	9,437398376	9,336885873		
PCP012170	8,935666001	9,070577167	9,299963518	9,657014692	PCP012170	XM_009363728
PCP012171	8,86727874	8,79983204	8,987747198	9,52028473	PCP012171	XM_018648644
PCP012186	9,156512913	9,139551352	9,24951645	9,555873659	PCP012186	XM_009351552
PCP012189	10,97608434	11,0863552	10,90337727	10,63147721	PCP012189	XM_018644128
PCP012211	10,13955135	10,09143539	10,10590851	10,03617361	PCP012211	XM_009366769
PCP012214	9,84497247	9,649256178	9,711374448	9,646252679	PCP012214	XM_008351989
PCP012215	9,255807556	9,336885873	9,517669388	10,11374217		
PCP012225	10,32493433	10,23202512	10,07236042	10,15566576	PCP012225	XM_009343782
PCP012232	10,01076585	9,964817355	10,22842189	10,87958325		
PCP012274	9,949826711	9,991989442	10,08037786	10,36777511		
PCP012289	9,699867382	9,497193195	9,283852339	9,336885873		

PCP012292	10,38694025	10,47337091	10,59089576	10,8566823	PCP012292	XM_009361613
PCP012305	9,16824589	9,032514148	8,95419631	9,115485887	PCP012305	XM_009368653
PCP012310	9,97441459	9,931727372	10,05754504	10,37721053		
PCP012332	8,961941603	8,934663924	9,182394353	9,056177063	PCP012332	XM_008342025
PCP012339	10,10678538	9,941531848	10,03846636	10,06383996	PCP012339	XM_009378488
PCP012383	8,661778098	9,245552706	9,325687724	10,45155114	PCP012383	XM_009380747
PCP012385	10,42940674	10,13741386	10,47944683	10,49685378	PCP012385	XM_009381445
PCP012413	11,91911286	12,14072434	12,72387422	11,9799003	PCP012413	XM_009351897
PCP012416	10,9710621	10,80842528	10,84862294	11,25974326	PCP012416	XM_009351886
PCP012432	9,072347016	9,038918989	9,219967107	9,603626345		
PCP012437	10,78763325	10,28193003	10,16449216	9,725655961	PCP012437	XM_008364399
PCP012474	9,14381685	9,195569065	9,091884919	8,30833903		
PCP012487	10,00702727	9,709083813	10,17033821	10,20741705	PCP012487	XM_008370016
PCP012489	9,713678633	9,601157931	9,630576566	9,633594681	PCP012489	XM_017327120
PCP012493	14,14768193	14,02695563	13,48242984	12,86186234	PCP012493	XM_009359175
PCP012525	9,601157931	9,315149562	9,545601627	9,631777296	PCP012525	XM_009344611
PCP012541	9,839203788	9,526166647	9,469641817	9,661778098	PCP012541	XM_017333448
PCP012548	8,74929961	9,064284694	10,10895563	12,73146117	PCP012548	XM_009363912
PCP012558	10,10852446	10,38010386	10,4118605	10,66562905	PCP012558	XM_009360867
PCP012578	9,64385619	9,468970167	9,591204414	9,886199551	PCP012578	XM_009350440
PCP012637	9,348728154	9,450530824	9,121533517	8,874458871		
PCP012645	8,842350343	9,160703421	9,182394353	11,19557522	PCP012645	XM_009381085
PCP012685	8,577428828	8,696967526	9,151447887	9,352418676		
PCP012760	9,193944186	9,206208529	10,00936677	9,857980995	PCP012760	XM_009367001
PCP012901	8,845490051	9,193944186	9,146365017	9,08833774	PCP012901	XM_009380354
PCP012921	11,32717648	11,62311788	12,20263504	13,53121939	PCP012921	XM_009336405
PCP012929	8,903881846	8,722226922	9,006550496	9,54239349	PCP012929	XM_009338878
PCP012933	15,23818079	14,98801892	15,3043039	15,63226623	PCP012933	XM_009338871
PCP012976	9,605479518	9,317412614	9,083027168	8,826548487	PCP012976	XM_009376873
PCP012985	9,82707272	9,537218401	9,735268302	9,743706817	PCP012985	XM_008358084
PCP012989	8,714245518	8,818038868	9	9,721099189	PCP012989	XM_009379912
PCP013001	9,237616296	8,976334992	9,164906927	9,494515616	PCP013001	XM_009365221
PCP013020	10,91513245	11,03800673	11,13485624	11,33501623		
PCP013046	8,926800034	8,893817229	8,963849777	9,416438656		
PCP013051	9,471675214	9,615316973	9,962896005	10,9213426	PCP013051	XM_008381326
PCP013055	9,615316973	9,524208672	9,584962501	9,448467384		
PCP013058	10,30796352	10,22641219	10,34687935	10,64806092	PCP013058	XM_008354748
PCP013075	9,965784285	10,08967557	10,12841972	10,10197567	PCP013075	XM_008354741
PCP013081	11,47775827	11,67168701	11,67154107	11,69508471		
PCP013084	9,836571147	10,11286951	10,16490693	10,1176431	PCP013084	XM_008370014
PCP013107	11,69884789	11,53592172	11,56446868	11,59820607		
PCP013109	9,904378722	9,75710661	9,988684687	10,19639311	PCP013109	XM_017334107
PCP013117	10,39446269	10,40372574	10,60733031	10,82681063		
PCP013123	9,842350343	9,925302224	10,39732167	11,42871086		
PCP013134	9,683573652	9,689997971	9,627533884	9,456005251	PCP013134	XM_008339473
PCP013137	10,6079498	10,57648435	10,90212635	11,55474712	PCP013137	XR_001952935

PCP013139	8,946409212	8,811631578	9,042562264	9,403715086		
PCP013141	10,36158196	10,74819285	11,72692911	13,71077093	PCP013141	XM_009354377
PCP013157	9,159871337	9,209453366	9,250298418	9,219967107	PCP013157	XM_009372971
PCP013180	9,772034091	9,273795599	9,588096205	9,693486957		
PCP013209	10,25738784	9,707359132	9,842868866	9,562872035	PCP013209	XM_009370890
PCP013216	10,77011049	10,41785251	11,23002044	11,01239408	PCP013216	XM_009350502
PCP013239	13,31226894	13,10678047	13,27306708	13,68090889	PCP013239	XM_018643342
PCP013256	9,049848549	8,903881846	9,33911511	10,06564501		
PCP013263	10,45738088	10,35167912	10,55362929	10,72280753	PCP013263	XM_009365990
PCP013269	9,391608782	9,464198563	9,600526229	9,873951581	PCP013269	XM_009366052
PCP013276	9,659995892	9,750422271	9,898344415	10,26091953		
PCP013286	8,751544059	8,731319031	8,866258916	9,19063955	PCP013286	XM_009366317
PCP013305	13,34003506	14,22947001	14,05547988	14,56347456	PCP013305	XM_009381288
PCP013307	9,336127147	10,57900473	10,3527828	10,81804686	PCP013307	XM_009381285
PCP013311	9,251079962	8,634193917	9,005624549	9,423473125	PCP013311	XM_009381293
PCP013313	9,372146798	9,321184013	9,430452552	9,315149562		
PCP013315	8,980139578	9,226412193	9,187352073	9,722807531	PCP013315	XM_009339845
PCP013331	8,810571635	8,689997971	8,792237182	9,186535222		
PCP013356	9,789533645	9,822299952	9,869855987	10,45087101		
PCP013361	10,10939974	10,30073006	10,13656824	9,854868383		
PCP013374	9,72395099	9,790625442	9,687673116	10,04530918	PCP013374	XM_009351446
PCP013379	9,06250492	9,074141463	8,970594857	9,459431619		
PCP013399	9,900866808	9,970105891	9,991054099	10,01495499	PCP013399	XM_009368005
PCP013403	13,08464232	12,97620609	13,20584364	13,80685166		
PCP013407	9,269898093	9,330916878	9,438791853	9,240004193	PCP013407	XM_009341676
PCP013415	10,6617781	11,21148073	11,70246275	12,4388776	PCP013415	XM_009370809
PCP013423	9,834992412	10,12240095	10,12412131	10,23999226		
PCP013427	10,46658634	10,23801057	10,53818893	10,66326455	PCP013427	XM_009370827
PCP013428	12,66006945	12,24406646	12,72841587	13,10579886	PCP013428	XM_009370828
PCP013445	8,142974967	9,051671182	9,951284715	9,630576566		
PCP013449	10,32830467	10,24911345	10,43948809	10,3818998	PCP013449	XM_008242209
PCP013457	10,17533747	9,960972093	10,30757652	10,69232781	PCP013457	XM_009354889
PCP013472	11,22380263	11,22741739	11,24198315	11,81043909	PCP013472	XM_009365139
PCP013492	10,22921538	10,32530546	10,80842528	10,83657904	PCP013492	XM_009359230
PCP013495	8,678318438	8,811631578	9,210257347	9,675957033	PCP013495	XM_009359248
PCP013496	9,22881869	9,50118036	9,611633479	9,669469742	PCP013496	XM_009359240
PCP013513	10,05663772	10,28733146	10,41397693	10,64595559		
PCP013514	10,71739368	10,73893668	10,83368075	10,69174352	PCP013514	XM_017326078
PCP013522	9,28077077	9,369968059	9,513076164	10,00936677	PCP013522	XM_008385922
PCP013546	8,77478706	8,882643049	8,89684702	9,411510988		
PCP013561	9,539158811	9,465566405	9,593073602	9,887220615	PCP013561	XM_009376740
PCP013601	9,388017285	9,405843489	9,71195944	10,68328415	PCP013601	XM_008395673
PCP013603	8,632995197	8,760453835	8,928755227	9,141264175	PCP013603	XM_009369647
PCP013609	11,17263377	11,42678889	11,43062504	11,65835878	PCP013609	XM_009369635
PCP013624	9,023283079	8,917879246	8,929761415	8,953236133		
PCP013627	9,089238597	9,075024475	9,083931345	9,482465137		

PCP013672	11,78067509	11,68460399	11,75126369	12,16898544	PCP013672	XM_008343339
PCP013673	9,685326985	9,48984796	9,799281622	10,28733146	PCP013673	XM_008343340
PCP013693	9,604238155	9,548821908	9,439477697	8,947403166	PCP013693	XM_009350397
PCP013700	9,938594555	9,734150722	10,09671515	10,23442228		
PCP013715	8,382278171	8,481113232	9,267559206	9,604868234	PCP013715	XM_018650665
PCP013718	9,10938668	9,111996335	9,14381685	8,844454703		
PCP013722	9,629957618	9,604868234	9,702744108	9,600526229		
PCP013729	10,78708632	10,2332242	11,15797845	12,18353304	PCP013729	XM_009381126
PCP013731	9,855911856	10,06025009	10,05979059	10,4015942	PCP013731	XM_009381129
PCP013734	11,19187968	11,20171349	11,3089078	11,21067134	PCP013734	XM_008338751
PCP013739	8,939579214	8,751544059	9,029756544	9,28925812	PCP013739	XM_009346870
PCP013752	9,286164981	9,415044544	9,433230722	9,388017285		
PCP013754	11,3149627	11,08347933	11,09649775	11,06317259	PCP013754	XM_008356823
PCP013758	8,832890014	8,706219712	8,884689491	9,142107057		
PCP013761	9,722244002	9,730181399	9,925302224	10,12928302	PCP013761	XM_009366543
PCP013768	10,26288177	10,08436993	10,10416627	9,915879379	PCP013768	XM_008380471
PCP013820	9,098032083	9,227206781	9,393025715	9,835529375	PCP013820	XM_020568996
PCP013830	10,85512543	10,76983784	10,86676119	11,13870058	PCP013830	XM_009340659
PCP013836	9,706202442	9,690574236	9,726780139	9,706789535	PCP013836	XM_009337818
PCP013854	10,04165915	10,05347794	10,22641219	10,17700835	PCP013854	XM_009358903
PCP013880	10,04939934	10,06383996	10,15228484	10,49752255	PCP013880	XM_009341587
PCP013881	10,36995716	10,28115343	10,36777511	10,36959735	PCP013881	XM_009341586
PCP013939	8,941546519	9,202931899	9,450530824	9,834992412	PCP013939	XM_008384928
PCP013944	8,660602089	9,370687407	9,456703495	9,472345607		
PCP013948	8,554588852	8,28692734	9,09011242	9,438791853	PCP013948	XM_009336352
PCP013950	11,88836274	11,87382857	12,12863881	12,47666268	PCP013950	XM_009336347
PCP013974	12,38235383	12,53584155	12,31741261	12,01250931	PCP013974	NM_001294026
PCP013989	8,376472723	8,667714757	9,094526396	9,361943774	PCP013989	XM_009374398
PCP013992	9,104153166	9,077696975	9,220789431	9,086587411	PCP013992	XM_009361467
PCP014003	10,78272799	11,36959735	11,9690267	12,75481862	PCP014003	XM_009369314
PCP014004	9,893301531	9,882643049	10,05979059	9,850702552		
PCP014018	10,25463892	10,56001751	10,71052234	10,71224325	PCP014018	XM_018643152
PCP014019	10,46658634	10,46692276	10,41256985	10,05619061	PCP014019	XM_009339047
PCP014028	10,11981006	10,23760435	10,33464201	10,77176181		
PCP014031	8,522267655	8,967716202	9,086587411	9,068778278	PCP014031	XM_009368322
PCP014041	10,4101015	10,11894107	9,497851837	11,203348	PCP014041	XM_009381360
PCP014047	10,11070515	10,48716675	10,52094273	11,16574239	PCP014047	XM_009352804
PCP014056	9,840258649	9,567956075	9,628135642	9,938109326	PCP014056	XM_018651171
PCP014070	10,80170836	10,71138305	10,83709964	11,14465824	PCP014070	XM_008366259
PCP014074	9,801433534	9,750422271	9,845490051	9,705062108	PCP014074	XM_008387452
PCP014076	9,582461918	9,727920455	9,611633479	9,402308618	PCP014076	XM_009360486
PCP014082	9,72395099	9,722244002	9,842868866	10,20945337	PCP014082	XM_009350030
PCP014089	9,324180547	9,273026268	9,446400988	9,698704667	PCP014089	XR_001788859
PCP014090	8,667714757	8,772578508	8,968666793	9,344295908		
PCP014107	9,952260754	10,08569817	10,12369465	9,937123663		
PCP014123	9,202931899	9,162391329	9,468970167	9,320416982		

PCP014138	12,47505701	12,44613472	12,67249827	12,73760016	PCP014138	XR_001951679
PCP014154	9,156512913	9,189009107	9,355351096	9,634212071	PCP014154	XM_009371928
PCP014183	8,868297843	8,886702554	9,116343961	9,341340908		
PCP014191	8,607330314	8,951284715	9,705062108	10,11504365	PCP014191	XM_009380238
PCP014209	9,452591317	9,303027245	9,453949632	9,353146825	PCP014209	XM_009345146
PCP014309	9,163221961	9,115485887	8,904875427	9,040755473	PCP014309	XM_009360623
PCP014347	11,29672697	11,1486863	11,19987475	11,67360501	PCP014347	XM_008390584
PCP014512	10,26013154	10,37250597	10,23720996	9,750991645		
PCP014532	14,34281569	14,20061742	14,15151844	13,87408515	PCP014532	XM_008356432
PCP014541	9,544964433	9,530737953	9,63540978	9,979668125		
PCP014543	11,21936821	11,27631636	11,30492167	10,9398216		
PCP014559	9,71539578	9,893301531	9,97441459	10,25266543	PCP014559	XM_018652252
PCP014562	9,712527	9,434628228	9,598666678	9,429050993	PCP014562	XM_009376004
PCP014567	9,571752644	10,01216358	9,967701794	9,987732989	PCP014567	XM_017327685
PCP014569	9,485165154	8,759322309	8,860062694	9,366322214	PCP014569	XM_009376025
PCP014578	10,11461429	9,961927137	10,15186643	10,05165759		
PCP014579	10,25935488	10,63571812	10,71138305	10,66829112	PCP014579	XM_009376045
PCP014583	9,30986284	9,457380879	9,336127147	9,390168956		
PCP014586	9,816455711	9,691743519	9,70333261	9,7409107	PCP014586	XM_009376061
PCP014590	8,827596761	8,861086906	9,618385502	8,873444113	PCP014590	XM_008357539
PCP014593	9,57680554	9,018673116	9,30986284	9,710238196	PCP014593	XM_008387790
PCP014601	8,918863237	8,912889336	8,987747198	9,567956075		
PCP014605	9,846540327	9,96337297	9,92381771	9,982993575		
PCP014624	9,299963518	9,290779173	9,466932951	9,793326936		
PCP014625	10,34207467	10,3586512	10,35093918	10,68591388		
PCP014646	8,908902953	8,861086906	9,013099307	9,499188155	PCP014646	XR_001790310
PCP014654	10,09891375	10,16574867	10,56573996	11,10000522	PCP014654	XM_009351501
PCP014656	10,33091688	10,13484983	10,21310422	10,49785184	PCP014656	XM_009351482
PCP014665	8,760453835	8,892785649	8,972491628	9,236803511	PCP014665	XM_008393957
PCP014697	8,591223118	8,884689491	9,469641817	10,49618469	PCP014697	XM_009353663
PCP014698	8,75043902	8,79983204	8,907882108	9,594324604		
PCP014700	10,81191249	10,87267488	10,77313921	10,95831345	PCP014700	XM_009353671
PCP014706	8,988684687	9,434628228	9,020507757	9,309089764		
PCP014707	9,33911511	9,452591317	9,54303182	9,369226538		
PCP014714	9,77148947	9,695802471	9,989635757	9,915386449	PCP014714	XM_009353697
PCP014715	10,01402047	9,875488276	9,985372344	10,52845411	PCP014715	XM_008382994
PCP014722	9,756005825	9,753216749	9,951765533	9,40017499	PCP014722	XM_009375655
PCP014743	9,137836494	9,293080746	9,330916878	8,91489335		
PCP014756	11,66533592	11,70289558	11,71496239	11,99870677		
PCP014760	9,895832748	9,915879379	10,01076585	10,45909345	PCP014760	XM_009348851
PCP014772	13,06159565	13,02317737	13,27394126	13,74770313		
PCP014810	10,1980398	10,09978174	10,05165759	10,11156607	PCP014810	XM_009355725
PCP014816	8,345760055	8,710806434	8,985841937	9,423473125		
PCP014820	11,2380046	11,23421868	11,31439631	11,14082977		
PCP014832	10,23720996	10,83709964	10,87395927	11,22359752	PCP014832	XM_009365815
PCP014836	9,20701432	8,885696373	9,119797095	9,133578937	PCP014836	XM_018649105

PCP014859	10,13869418	10,35351076	10,21513253	10,23840474		
PCP014876	9,187352073	8,937609223	9,048023611	9,064284694	PCP014876	XM_018650483
PCP014882	8,763776717	9,056177063	9,02699366	9,030667136		
PCP014885	9,8159116	9,714812179	9,733015322	10,33502182	PCP014885	XM_009373449
PCP014886	9,131856961	8,941546519	8,824417827	8,537878433		
PCP014900	9,894817763	9,970105891	9,953716302	9,90287251	PCP014900	XM_018650833
PCP014914	9,381542951	9,159038773	9,1807297	9,348728154		
PCP014945	10,80224977	10,68006937	10,71195084	11,04984855		
PCP014979	8,944477815	9,095397023	8,936637939	8,912889336	PCP014979	XM_009377678
PCP014984	9,82124782	10,07190476	10,3786525	11,2380046	PCP014984	XM_009377770
PCP014986	11,80695233	11,82535634	11,75112558	11,89089883	PCP014986	XM_008353091
PCP015003	9,912395383	9,696967526	9,819652219	10,15440043		
PCP015023	9,042562264	8,950322598	9,213517401	9,503825738	PCP015023	XM_009340339
PCP015033	9,794951903	9,809494835	9,828136484	9,786269628	PCP015033	XM_009365594
PCP015051	10,86805085	10,88696923	10,98560716	11,34872815	PCP015051	XM_009363859
PCP015078	11,09715646	11,04780555	11,15860969	11,13485624	PCP015078	XR_001953838
PCP015098	10,63026713	10,61991732	10,77944852	10,39767463	PCP015098	XM_009377332
PCP015111	10,36705431	10,18031945	10,11156607	10,61409325		
PCP015119	8,84024291	8,940548521	8,887738491	9,620219826		
PCP015123	9,394462695	9,407267764	9,658800582	10,00281502		
PCP015137	9,97632067	10,04028972	10,11981006	10,0588982		
PCP015170	9,02699366	9,195569065	9,136991112	9,155653112	PCP015170	XM_009347109
PCP015176	8,886702554	8,938609256	9,47370575	10,0018726	PCP015176	XM_009347101
PCP015179	10,10459875	10,18982456	10,42171772	11,00982862		
PCP015196	8,922822231	8,75598914	9,011227255	9,254249162	PCP015196	XM_008382982
PCP015210	8,741466986	8,759322309	8,980139578	9,345028167	PCP015210	XM_009344629
PCP015230	13,28939502	13,09967655	13,34914298	13,70040395	PCP015230	XM_009361941
PCP015234	9,80843332	9,63481105	9,762664404	9,699867382	PCP015234	XM_009361957
PCP015260	11,85992297	11,80547385	12,08724072	12,66259495	PCP015260	XM_008374843
PCP015267	10,39267161	9,814854798	9,982523054	9,494515616	PCP015267	XM_009365894
PCP015281	9,422758607	9,037080165	9,240791332	9,337621902	PCP015281	XM_009353349
PCP015283	9,419960178	9,512404684	10,01216358	9,975375111	PCP015283	XR_666712
PCP015303	10,25856603	10,08569817	10,09539702	10,14551188	PCP015303	XM_009370958
PCP015309	11,77684423	11,59385794	11,54077221	11,73626239		
PCP015317	10,2179577	9,972017669	9,892284763	10,06069593	PCP015317	XM_009368263
PCP015318	9,722244002	9,24951645	9,581200582	9,763212367	PCP015318	XM_009368471
PCP015341	11,42836017	11,47590059	11,57301753	11,51799902	PCP015341	XM_009372841
PCP015350	8,91489335	9,053464362	9,167418146	9,468277836	PCP015350	XM_008363435
PCP015354	10,59276535	10,46522967	10,67830963	11,00211178		
PCP015369	9,606719814	9,630576566	9,47100451	8,895817605	PCP015369	XM_018647842
PCP015390	9,066977144	8,89684702	9,104153166	9,342808055	PCP015390	XM_009363153
PCP015415	8,709669735	8,76927586	8,946409212	9,321184013	PCP015415	XM_009360686
PCP015419	9,731879027	10,11113567	10,09671515	11,46267097		
PCP015475	10,83709964	10,73696841	10,80547788	11,07770365		
PCP015480	9,574271841	9,534633604	9,357552005	8,64385619	PCP015480	XM_018647298
PCP015482	9,748746336	9,527477006	9,862637358	9,83447105	PCP015482	XM_018647296

PCP015491	11,33408889	11,41309898	11,44811631	11,476407	PCP015491	XM_009339086
PCP015512	9,124974266	9,283088353	9,218369492	9,567956075	PCP015512	XM_009344547
PCP015517	11,53915881	11,42294252	11,4252159	11,42992974	PCP015517	XM_018647282
PCP015555	10,09099894	10,017741	10,18115226	10,50548408	PCP015555	XM_008370343
PCP015562	9,185693134	9,054387253	9,123267853	9,037080165	PCP015562	XM_009355740
PCP015567	10,62357975	10,39909631	10,59681404	10,77616158	PCP015567	XM_009355668
PCP015613	9,743151394	9,804131021	9,983463942	9,895832748	PCP015613	XM_008356635
PCP015674	8,735826768	8,719970574	8,765965634	9,510427928	PCP015674	XM_009336000
PCP015676	9,170751309	9,098032083	9,077696975	9,54173552		
PCP015692	10,0945132	10,04028972	10,08304047	10,06877828	PCP015692	XR_664979
PCP015702	10,73583523	10,87190524	10,8677961	11,11525828		
PCP015735	8,956143797	8,689997971	8,997179481	9,195569065		
PCP015740	10,27999355	10,32229999	10,30415735	10,31967212	PCP015740	XM_009361502
PCP015797	9,193106413	9,154818109	9,276892087	9,709083813		
PCP015798	11,3170338	11,32867493	11,25876034	10,92950254	PCP015798	XM_009341361
PCP015804	11,29001885	11,28173881	11,38909352	11,66058872		
PCP015821	10,00982862	9,812177306	9,984888362	10,29155061		
PCP015824	11,22339841	11,32624408	11,25778264	11,20864284	PCP015824	XM_009379993
PCP015830	11,54930337	11,39642818	11,34522226	11,26893376	PCP015830	XM_009351968
PCP015889	10,05301628	9,911886285	10,05619061	10,3383687	PCP015889	XM_017331478
PCP015919	12,09967655	12,15618739	12,40247917	12,74651432	PCP015919	XM_009370512
PCP015930	10,67006312	10,41433641	10,39446269	10,19475685		
PCP015933	10,67860014	10,68035952	10,9346713	11,11807805	PCP015933	XM_009346384
PCP015937	10,89809496	10,97226185	11,09847299	11,29901908	PCP015937	XM_008393049
PCP015950	8,722226922	8,939579214	8,763776717	9,414325838	PCP015950	XM_008359023
PCP015963	12,16386034	11,99258434	11,87651695	12,07380685	PCP015963	XM_008375970
PCP016036	9,89985536	9,691743519	9,923327485	10,68678939	PCP016036	XM_009378737
PCP016074	10,72792045	10,55906156	10,68941274	10,09143539	PCP016074	XR_666943
PCP016214	10,45738088	10,11070515	10,08304047	10,16449216	PCP016214	XM_009364967
PCP016221	9,598666678	9,619614757	9,535916864	9,529430554	PCP016221	XM_009337864
PCP016230	8,647458426	8,782441329	8,919846558	9,250298418		
PCP016231	10,29806257	10,03066714	10,21148681	10,49352525		
PCP016252	8,925791779	9,047123912	9,283088353	9,633594681	PCP016252	XM_009371267
PCP016272	9,11113567	9,028817757	9,200481794	9,489185654		
PCP016282	8,637820329	8,78571401	8,95805965	9,122388003	PCP016282	XM_008339920
PCP016297	10,11026144	10,09407769	10,0768156	10,48449062	PCP016297	XM_009373336
PCP016303	9,54239349	9,598666678	9,812177306	10,01168851	PCP016303	XM_009380274
PCP016304	9,223205318	8,924812504	9,182394353	9,372146798	PCP016304	XM_009380264
PCP016317	11,02859678	10,66711154	10,73047014	11,05799172		
PCP016332	9,118084543	9,202931899	9,30986284	9,668884984	PCP016332	XM_009376592
PCP016358	8,723387907	9,044394119	9,318158642	9,722244002	PCP016358	XM_009376207
PCP016363	10,65761321	10,85330956	10,77505877	10,91637961	PCP016363	XM_009363414
PCP016365	14,3408703	14,34988038	14,51438552	14,82580351	PCP016365	XM_009363331
PCP016369	9,640841416	9,660584264	9,764324258	10,12541347		
PCP016370	8,727920455	8,851749041	8,907882108	9,252665432		
PCP016421	8,993419626	9,064284694	9,05709822	9,066977144	PCP016421	XM_009341514

PCP016439	11,19023829	10,45361017	10,9331964	11,56953761	PCP016439	XM_009341499
PCP016478	9,391608782	9,821773982	9,986311377	10,39695792	PCP016478	XM_009377791
PCP016496	10,71195084	10,75655632	10,76597392	11,16637024	PCP016496	XM_009337686
PCP016500	8,98205238	9,057991723	8,982993575	8,929761415		
PCP016563	9,039823818	8,862110391	9,017727086	9,285402219	PCP016563	XM_009359362
PCP016572	10,33278624	9,969631147	10,01634867	9,775330438		
PCP016593	12,19013332	12,31061278	12,68489542	13,21376191	PCP016593	XM_009352246
PCP016609	11,83683936	11,4971882	11,83828606	12,23999525	PCP016609	XM_009365848
PCP016614	8,666508075	8,759322309	8,851749041	9,107661718		
PCP016618	9,794951903	9,951765533	9,721099189	9,895317771	PCP016618	XM_009365835
PCP016635	9,096267125	9,020507757	9,161560218	8,909893084	PCP016635	XM_008339177
PCP016638	11,02075081	11,09781487	11,11894107	11,18487534	PCP016638	XM_018644862
PCP016655	8,943481842	9,473035989	9,505156537	9,489185654	PCP016655	XM_009366596
PCP016661	9,49651428	8,265239967	8,383704292	10,87190524		
PCP016681	11,65090365	11,81297947	11,87075	12,28655776		
PCP016688	10,02974274	10,00235092	9,920352855	9,749869427	PCP016688	XM_018649396
PCP016695	10,10634047	9,731319031	10,01262454	9,875488276		
PCP016731	9,55522181	9,56414949	9,710806434	9,594324604	PCP016731	XM_018652152
PCP016740	9,139551352	8,91489335	8,929761415	8,938609256		
PCP016754	9,151447887	9,344295908	9,456005251	9,790625442		
PCP016768	9,154818109	9,24951645	9,283088353	9,624484772	PCP016768	XM_009350480
PCP016773	10,5955652	10,30263892	10,69783636	10,72052644	PCP016773	XM_008354674
PCP016775	9,851233701	10,00046486	10,10939974	9,898344415	PCP016775	XM_009350081
PCP016789	8,860062694	8,943481842	8,910882535	9,321184013		
PCP016810	13,79136641	13,91382439	14,00140819	14,98272625	PCP016810	XM_009371764
PCP016816	8,804131021	8,654636029	8,967716202	9,119797095	PCP016816	XM_009347006
PCP016824	9,8159116	10,14635229	9,734150722	10,04484489	PCP016824	XM_008361564
PCP016825	9,816983623	9,384416825	9,22881869	9,399448837	PCP016825	NM_001319808
PCP016829	10,31174832	10,18198458	10,20863674	10,51668495	PCP016829	XM_008368939
PCP016830	10,18198458	10,21350525	10,39410894	11,05753827	PCP016830	XM_008392471
PCP016840	11,80654962	11,96458274	12,03720374	12,15555509		
PCP016881	8,960957618	9,173252384	9,131856961	9,184875343		
PCP016889	9,006550496	8,787902559	8,740354199	8,934663924	PCP016889	XM_009340545
PCP016918	8,692336525	8,816983623	8,993419626	9,345028167		
PCP016921	9,026053072	9,16824589	9,71195944	9,536577492		
PCP016926	9,749869427	9,7409107	9,477090385	9,441554026	PCP016926	XM_009360940
PCP016928	8,796591265	9,003742767	9,342074668	9,849139214	PCP016928	XM_018648100
PCP016929	9,21916852	9,067891433	9,199672345	9,73809226	PCP016929	XM_009360948
PCP016936	9,889245365	9,816983623	9,908392621	9,805211831	PCP016936	XM_017328848
PCP016939	9,032514148	9,098900598	9,336127147	10,1301458	PCP016939	XM_017326188
PCP016957	11,11850638	11,03043954	11,0500731	10,83235998		
PCP016966	10,98631849	11,45601039	11,28905635	11,30416306	PCP016966	XM_009351815
PCP016971	9,651051691	9,929258409	10,19926745	10,41925445		
PCP016974	9,837627933	9,6171552	9,981567282	10,14889598		
PCP016986	9,445697739	9,048023611	9,490509963	9,224001674	PCP016986	XM_008387634
PCP017003	8,816983623	9,123267853	9,324180547	10,02052165	PCP017003	XM_009361436

PCP017004	9,216745858	9,286950435	9,136991112	8,815927606		
PCP017009	10,3305472	9,660584264	9,162391329	8,625708843		
PCP017043	8,727920455	8,953236133	9,072347016	9,28000515	PCP017043	XM_009344061
PCP017051	13,92944336	13,8162837	13,90162116	14,22188916	PCP017051	XM_009344070
PCP017060	11,90149672	11,8761332	12,12788795	12,19752388	PCP017060	XM_008381783
PCP017080	9,012149614	9,113742166	9,391608782	9,424166289	PCP017080	XM_009367525
PCP017098	10,86444153	10,91014051	11,00772811	11,38980877		
PCP017106	10,31061278	10,26482961	10,31628153	10,22881869	PCP017106	XM_009349798
PCP017122	11,49001349	11,22138774	11,21249639	11,05234389	PCP017122	XM_009345705
PCP017143	8,939579214	8,67479253	9,071462363	9,505156537	PCP017143	XM_009354248
PCP017148	10,84549005	10,72308921	10,84339505	10,82681063		
PCP017158	10,2463487	10,32117274	10,36120908	10,833151	PCP017158	XM_009369880
PCP017166	9,672425342	9,720534991	9,64385619	9,961927137	PCP017166	XM_009356442
PCP017183	9,131008067	9,06608919	9,375039431	9,285402219	PCP017183	XM_018651648
PCP017219	9,625708843	9,869855987	9,346513733	8,209453366		
PCP017220	11,84901407	11,95152879	12,0665366	11,92864051	PCP017220	XM_009361932
PCP017232	9,609788199	9,501837185	9,303780748	8,743723645	PCP017232	XM_009371923
PCP017237	9,072347016	9,25502857	9,324923085	9,713678633		
PCP017265	10,90036117	10,72678865	10,80681542	10,79007965	PCP017265	XM_009343309
PCP017287	14,13752514	14,06513588	13,90488577	13,61455687		
PCP017297	11,19290922	11,24555271	11,23541594	11,2524703		
PCP017315	9,531381461	9,204571144	9,055282436	8,80196697	PCP017315	XM_008371311
PCP017317	10,04028972	10,54464573	10,52715442	11,08015131		
PCP017318	10,10721708	10,08304047	10,48280796	10,70505347	PCP017318	XM_018648700
PCP017338	10,89051509	10,99387953	11,06317259	11,01982004	PCP017338	XM_008357736
PCP017352	11,11612949	11,27204652	11,57159623	12,09879864	PCP017352	XM_009376302
PCP017414	9,796575043	9,84497247	9,848106482	9,813781191	PCP017414	XM_009376191
PCP017436	8,942514505	8,842350343	9,169925001	9,505811554		
PCP017447	9,243173983	9,28925812	9,372146798	9,258165557	PCP017447	NM_001302323
PCP017457	10,05301628	10,02375435	10,31816994	11,04143329		
PCP017494	11,18156848	10,78653919	10,55906156	10,31929955	PCP017494	XM_008379527
PCP017504	12,30947635	11,8264174	11,56906286	11,39142083	PCP017504	XM_009380445
PCP017507	11,35388012	11,43653978	11,43862034	11,04280163	PCP017507	XM_018647237
PCP017519	9,925806611	10,31703945	10,37395266	10,46760555		
PCP017533	9,462154593	9,558420713	10,07948478	11,39178061	PCP017533	XM_009355415
PCP017590	9,037080165	9,094526396	9,19063955	8,721099189	PCP017590	XM_008373343
PCP017600	11,32717648	10,94763694	10,65791684	10,82708066	PCP017600	XM_018652266
PCP017611	11,52127162	11,52028964	11,60594244	11,94153552	PCP017611	XM_008384851
PCP017690	10,32979634	10,18982456	10,22077734	10,04530918	PCP017690	XM_008366547
PCP017748	9,437398376	9,419960178	9,753216749	10,19475685		
PCP017754	10,02190996	10,21714588	9,804131021	9,661778098	PCP017754	XM_009346388
PCP017774	9,408690635	9,226412193	9,268354853	9,019590728	PCP017774	XM_009371517
PCP017785	9,753216749	9,669469742	9,862125893	10,12454785	PCP017785	XM_009371500
PCP017787	9,230428801	9,286164981	8,792237182	8,199672345	PCP017787	XM_009371497
PCP017818	9,895832748	9,950308015	10,01262454	9,974887762	PCP017818	XM_009371452
PCP017822	8,721099189	8,730198386	8,939579214	9,200481794		

PCP017827	13,15407917	12,72742487	12,88410754	13,22208998		
PCP017835	9,142107057	9,259743264	8,991521846	9,439477697	PCP017835	XM_009353982
PCP017838	13,41256985	13,43423727	13,47231006	13,86053706	PCP017838	XM_009353977
PCP017858	9,048023611	8,966736149	9,020507757	9,016808288	PCP017858	XM_009353949
PCP017860	9,11113567	9,048949988	9,079484784	9,456005251	PCP017860	XM_009353948
PCP017864	11,33017182	11,12326139	11,2805794	11,57127862		
PCP017877	9,371406396	9,401583539	9,414325838	9,356826076	PCP017877	NM_001328903
PCP017896	8,700439718	8,714245518	9,269126679	9,779177627	PCP017896	XM_009347269
PCP017906	9,622655867	9,330177419	8,897845456	8,113742166	PCP017906	XM_009362362
PCP017907	9,385862401	9,50249371	9,754336368	10,05754504	PCP017907	XM_009361771
PCP017911	11,85901834	11,91724899	11,92159179	11,9009913	PCP017911	XM_008386023
PCP017928	9,815383296	9,36047402	9,463524373	9,075933681	PCP017928	XM_009359330
PCP017936	8,900866808	8,970594857	9,324923085	9,703903573	PCP017936	XM_009340785
PCP017938	9,354624059	9,315896762	9,528786176	9,759888183	PCP017938	XM_018649155
PCP017940	9,54689446	9,423473125	9,428360173	9,315149562	PCP017940	XM_009341468
PCP017977	10,71881539	10,72052644	10,96048709	10,8960826		
PCP017989	10,41222051	10,41468524	10,34466208	10,5372184	PCP017989	XR_001954281
PCP017995	9,701011827	9,609178738	9,911391988	9,968191576		
PCP018010	11,36741475	11,29270699	11,50382574	11,7396414	PCP018010	XR_001953854
PCP018041	11,9684256	11,90199819	12,12896095	12,66133275	PCP018041	XM_008363765
PCP018052	10,44535633	10,32268306	10,58371275	10,3858624	PCP018052	XM_018650943
PCP018057	9,650459419	9,546257836	9,522248035	9,466932951		
PCP018065	10,78626963	10,81511907	10,84392105	11,23302043	PCP018065	XM_009339222
PCP018076	9,884674229	10,34021731	10,44948916	10,8587581		
PCP018082	9,895317771	9,858493931	9,969631147	10,31477582		
PCP018103	8,710806434	8,636624621	8,912889336	9,153121312		
PCP018105	13,4043872	13,5156173	13,38698469	14,03714625	PCP018105	XR_001952838
PCP018112	9,161560218	9,052568051	9,198862442	9,130132926	PCP018112	XM_009378974
PCP018135	9,533972085	9,479780264	9,772034091	10,05256805		
PCP018150	10,67830963	10,57711722	10,67065625	11,24634276		
PCP018181	8,856954575	8,901862467	8,739206792	8,828676005	PCP018181	XM_009362853
PCP018182	9,033423002	9,265216522	9,276892087	9,591840209	PCP018182	XM_009362849
PCP018183	10,00796633	10,20579325	10,53656778	10,99293834	PCP018183	XM_009362852
PCP018217	8,73809226	9,079484784	8,899870461	8,83709175	PCP018217	XM_009356958
PCP018223	10,27961058	9,843402907	9,915879379	10,21674586	PCP018223	XM_009356973
PCP018242	8,876516947	8,851749041	8,899870461	8,795487741	PCP018242	XM_008369155
PCP018259	9,207819661	9,198862442	9,13442632	9,20701432	PCP018259	XM_009370017
PCP018267	9,410811715	9,424166289	9,514378734	9,855382427	PCP018267	XM_009370028
PCP018285	10,64955259	10,34614803	10,43010752	10,52061868		
PCP018290	11,28289729	11,22339841	11,72352017	11,48465677	PCP018290	XM_008378962
PCP018291	10,05619061	9,958552715	10,2617071	10,41397693	PCP018291	XM_008358920
PCP018293	13,85327739	13,88753765	13,95022143	13,87325184	PCP018293	XM_009337351
PCP018315	10,76791131	10,64595559	10,52519778	10,45327063		
PCP018329	9,752664976	9,718823954	9,472345607	9,815383296		
PCP018343	9,152284842	9,08127038	9,086587411	9,511100329		
PCP018357	9,14890869	8,976334992	9,089238597	9,466932951	PCP018357	XM_009338813

PCP018360	9,593708574	9,68707819	9,626311362	9,56732868	PCP018360	XM_009338810
PCP018364	9,619614757	9,728481771	9,901364723	9,77807713	PCP018364	XM_009365164
PCP018366	11,60300964	11,64865867	11,84718657	12,04939593		
PCP018381	11,1372025	10,97489493	11,29825163	11,60779264	PCP018381	XM_009369341
PCP018382	13,24406646	13,26781082	13,4192584	13,73085963		
PCP018424	8,553322101	8,732438807	8,968666793	9,112882543		
PCP018453	10,09054913	9,806823474	10,04257594	9,810571635		
PCP018454	9,694653884	9,740337332	9,853824155	9,762099618	PCP018454	XM_009374392
PCP018456	10,78108918	10,46726929	10,70707436	11,05166439	PCP018456	XM_009374389
PCP018468	9,078604498	9,072347016	8,877529547	9,563520437		
PCP018483	9,553955615	9,497851837	9,589969422	9,220789431	PCP018483	XM_009339513
PCP018526	9,013099307	9,043492153	8,941546519	8,940548521	PCP018526	XM_009379531
PCP018528	9,326429487	9,412929682	9,461479447	9,731879027	PCP018528	XM_009361208
PCP018535	10,83025374	10,96914905	10,97154355	10,97561156	PCP018535	XM_009361226
PCP018536	8,677120596	8,707359132	8,85277917	9,476401941	PCP018536	XM_009361224
PCP018547	9,894317583	9,86469688	9,864186145	9,878050913	PCP018547	XM_009336798
PCP018551	9,716527978	9,82707272	10,23720996	10,47742436		
PCP018559	9,436024381	8,899870461	8,885696373	8,390899794		
PCP018588	11,07258149	11,07280253	11,25345161	11,53300358	PCP018588	XM_008379382
PCP018592	8,762647796	8,761551232	8,97441459	9,599912842		
PCP018596	10,86418614	10,88315493	10,98489548	10,93147623	PCP018596	XM_009380992
PCP018613	9,968666793	9,879077758	9,713094337	9,19802752	PCP018613	XM_018648240
PCP018617	10,79982395	10,64775521	10,6694786	10,85642553		
PCP018636	10,77204234	10,95927751	11,2628759	11,49385545	PCP018636	XM_009375251
PCP018637	9,799281622	9,792253453	9,986311377	9,857467877	PCP018637	XM_018651229
PCP018649	9,105908509	9,176597042	9,243959396	9,187352073	PCP018649	XM_009343607
PCP018656	9,365600686	9,086587411	9,226412193	8,84024291		
PCP018661	9,795503976	9,892284763	10,14252831	11,0409746		
PCP018662	11,74244839	11,26052559	11,65597786	11,89190898		
PCP018707	9,146365017	9,046223651	10,66562905	11,05956754		
PCP018709	9,02699366	9,251884747	9,479113318	9,836571147	PCP018709	XM_008367695
PCP018714	11,11547938	10,93393403	11,14656868	11,41960736	PCP018714	XM_009345685
PCP018721	9,095397023	9,080364533	9,243173983	9,688250309		
PCP018730	9,282323962	9,333893285	9,336127147	9,215945486	PCP018730	XM_009336296
PCP018734	12,74237892	12,7077905	12,81090697	12,7322374	PCP018734	XM_009367140
PCP018747	10,37359384	10,39839099	10,4419067	10,74846962	PCP018747	XM_018644209
PCP018766	9,805743872	9,692319087	9,755438426	10,29462075	PCP018766	XM_008387204
PCP018774	9,087462841	9,057991723	9,131856961	9,000929563	PCP018774	XM_008379808
PCP018781	9,405843489	9,393025715	9,729620744	9,30833903		
PCP018798	10,56826967	10,68533575	10,60146439	10,54592977	PCP018798	XM_009381121
PCP018811	8,336863563	8,661778098	9,05709822	9,46760555	PCP018811	XM_009338146
PCP018823	11,14550551	11,15101654	11,30359241	11,22057785	PCP018823	XM_008342956
PCP018845	8,819125283	8,887738491	8,910882535	9,50118036		
PCP018852	10,37322406	10,48616127	10,45532722	10,53495453	PCP018852	XM_009377933
PCP018870	12,47893895	12,5533101	12,4482015	12,39151212	PCP018870	XM_009355890
PCP018876	10,48984796	10,43532647	10,5526691	11,01471793	PCP018876	XM_009361581

PCP018886	9,910897522	10,04939934	10,27922751	10,833151	PCP018886	XM_009335941
PCP018909	9,058884672	9,054387253	9,02699366	8,967716202	PCP018909	XR_001954674
PCP018912	9,873951581	9,897845456	9,817511342	10,13570929	PCP018912	XM_009370484
PCP018944	10,81618368	10,5143886	10,33352436	10,16154762	PCP018944	XM_018645961
PCP018948	10,00422047	10,55458885	10,85564717	11,55203021		
PCP018963	10,95177282	10,2199792	10,48482289	10,5307282	PCP018963	XM_018645714
PCP018969	9,011227255	9,040755473	9,275356315	9,691167722	PCP018969	XM_008356302
PCP019004	8,668884984	8,998110862	9,114601277	9,609788199	PCP019004	XM_009336610
PCP019009	10,53786873	10,47438534	10,64505493	10,93245838		
PCP019076	9,089238597	9,105908509	9,561612539	10,09364204	PCP019076	XM_008385562
PCP019095	9,159038773	9,094526396	9,091884919	9,08127038		
PCP019173	14,22972009	13,0760924	13,45763738	12,89904153	PCP019173	XM_008385924
PCP019195	9,00750388	9,113742166	9,33315535	9,786269628		
PCP019253	10,23521247	10,18818083	10,89658217	10,76597392		
PCP019277	9,330916878	9,195569065	9,352418676	9,616548844	PCP019277	XM_008379925
PCP019290	9,654045226	9,651051691	9,73470962	9,601770788		
PCP019300	13,24495839	12,90224885	12,85759229	13,17539361	PCP019300	XM_009360205
PCP019301	10,06249143	10,06474276	10,16364968	10,45052051	PCP019301	XM_009360204
PCP019312	12,35700209	12,04814625	12,10612778	11,82588498	PCP019312	XM_009360184
PCP019318	12,48137816	12,38891331	12,57538088	12,95546829		
PCP019321	10,99246397	11,19660213	11,20904511	11,45635442	PCP019321	XM_008369525
PCP019322	10,3527828	10,37395266	10,54721267	10,85356688		
PCP019323	10,61256445	10,61900026	10,74959293	11,10153584	PCP019323	XM_009360156
PCP019338	10,59525748	10,55554777	10,75182019	11,10546987		
PCP019339	9,285402219	9,294620749	9,255807556	9,637838439		
PCP019351	11,51405813	11,54512376	11,48213737	11,76873433	PCP019351	XM_009372514
PCP019353	9,510427928	9,417852515	9,700439718	9,408690635		
PCP019367	11,05912135	11,34318572	11,62174512	11,891404		
PCP019373	8,792237182	8,903881846	8,833933692	9,433940073		
PCP019416	13,18941226	12,56811526	12,41565463	11,67815996	PCP019416	XM_009377170
PCP019418	11,26404962	10,11504365	10,91886324	11,03732044	PCP019418	XM_009377173
PCP019445	10,36084708	10,42906146	10,51142653	10,77698401	PCP019445	XM_009339373
PCP019446	8,917879246	8,874458871	9,053464362	8,955155849	PCP019446	XM_009339353
PCP019449	9,767638247	9,936152052	9,703903573	9,633594681	PCP019449	XM_018643178
PCP019461	9,730758817	9,799281622	9,705062108	9,662970945	PCP019461	XM_009339105
PCP019484	9,367043381	9,133578937	9,413627929	9,192292814		
PCP019486	9,068778278	9,164906927	9,522248035	10,14040802	PCP019486	XM_009377659
PCP019496	9,484480553	9,573647187	9,595574522	9,985372344	PCP019496	XM_009377640
PCP019517	10,12153352	10,11547288	10,63026713	11,15101654	PCP019517	XM_008378869
PCP019521	15,50965131	15,31455952	15,38783795	15,25355206	PCP019521	NM_001328803
PCP019545	10,11851287	10,06114164	10,75544677	11,24476222	PCP019545	XM_017334000
PCP019555	10,57774037	10,3383687	10,29424165	10,35938719	PCP019555	XM_008370322
PCP019572	8,994353437	9,125852541	8,939579214	8,905898402	PCP019572	XM_008370568
PCP019596	8,813781191	8,809510912	8,908902953	9,244768161		
PCP019615	12,72905349	12,73612072	12,84529402	12,76459588		
PCP019619	12,6071754	12,49493806	12,32418055	12,62357747	PCP019619	XM_018649575

PCP019641	11,60501645	11,78803719	11,87023755	12,09627701	PCP019641	XM_008352363
PCP019651	10,48515509	10,6435586	10,65314065	10,47269084	PCP019651	XR_526443
PCP019657	10,22520744	10,35240764	10,47370575	10,39588754	PCP019657	XR_670338
PCP019666	10,16867212	10,49951706	11,02283245	11,41609553	PCP019666	XM_018652402
PCP019670	9,159871337	9,203763987	9,2589664	9,615316973		
PCP019674	10,40016431	10,38873845	10,57490568	10,85953479	PCP019674	XM_021969724
PCP019678	9,458057945	9,472345607	9,607330314	9,994353437		
PCP019687	12,68189723	12,70570366	12,6959525	12,5049009	PCP019687	XM_009357531
PCP019722	11,78599593	10,99647349	11,4236412	11,44931891	PCP019722	XM_009372996
PCP019724	11,43375755	11,07392063	11,37467545	10,9331964	PCP019724	XM_009372993
PCP019748	9,38945926	9,440183984	9,539798574	9,421370591	PCP019748	XM_018645402
PCP019753	10,46726929	10,59308294	10,73921523	10,98085361	PCP019753	XM_009360844
PCP019759	9,498510178	9,560963278	9,703903573	10,22721882	PCP019759	XM_009360855
PCP019781	9,717110885	8,962896005	9,414325838	9,719953466		
PCP019783	9,035266491	9,215945486	9,094526396	9,16824589	PCP019783	XM_009354606
PCP019798	8,572397068	8,70276142	8,73809226	9,320416982	PCP019798	XM_009354636
PCP019801	9,155653112	9,113742166	9,274564521	9,654045226	PCP019801	XM_009354639
PCP019816	8,960957618	8,631795481	8,983934156	8,758223215	PCP019816	XM_009336466
PCP019826	10,67977037	10,34170783	10,29844067	10,20579325	PCP019826	XM_009336440
PCP019830	9,330916878	9,197216693	9,859534786	10,63934071	PCP019830	XM_009335627
PCP019831	10,83736775	10,47201045	10,98037525	11,48900999	PCP019831	XM_009335629
PCP019838	9,442259297	9,528786176	9,667697019	10,23999226	PCP019838	XM_009335557
PCP019847	8,764871591	8,915879379	9,059804103	9,392317423	PCP019847	XM_008375764
PCP019850	8,816983623	8,846524657	8,979196518	9,271463028	PCP019850	XM_008375760
PCP019856	9,163221961	9,135273206	9,371406396	9,613476402		
PCP019882	9,399448837	9,369968059	9,385150582	9,374322251	PCP019882	XM_009368975
PCP019895	9,269898093	9,082149041	9,182394353	8,975360779	PCP019895	XM_009381200
PCP019898	8,893817229	8,806291831	9,01402047	9,236803511	PCP019898	XM_009381196
PCP019925	9,775890069	9,267559206	9,797661526	9,425572598		
PCP019929	8,951284715	9,022367813	8,939579214	8,831845581	PCP019929	XM_009370184
PCP019949	9,558420713	9,012149614	9,486493158	9,124974266	PCP019949	XM_018649528
PCP019956	9,30833903	8,906890596	9,086587411	8,905898402		
PCP019968	9,19063955	9,618991087	9,73753467	11,19741944	PCP019968	XM_009346732
PCP019994	9,721663165	9,742595757	9,805743872	10,17158965		
PCP019997	9,997645247	9,363412032	9,73809226	9,140395236	PCP019997	XM_009364001
PCP020015	10,5943246	10,58714931	10,66651695	11,21735191	PCP020015	XM_018646931
PCP020016	9,451891079	9,513727596	9,524874682	9,873444113	PCP020016	XM_008385223
PCP020025	9,217545787	9,191478757	9,324180547	9,612868497	PCP020025	XM_008382293
PCP020027	11,51191569	11,6249462	11,68576936	11,68576936	PCP020027	XM_008382298
PCP020044	9,390168956	9,514378734	9,759888183	10,47505448	PCP020044	XM_009335417
PCP020055	11,08768162	10,90137227	11,43393486	12,03880585	PCP020055	XM_009339681
PCP020056	9,807886174	9,875488276	9,983948403	10,35388563	PCP020056	XM_009339736
PCP020058	10,56064809	10,37177664	10,26288177	9,827612638		
PCP020086	11,09319966	10,97775931	11,30701292	11,76914773	PCP020086	XM_009356593
PCP020092	10,84444686	10,92332749	10,97249881	11,29710542	PCP020092	XM_009376135
PCP020105	8,961941603	8,998110862	9,070577167	9,814854798	PCP020105	XM_009351878

PCP020126	10,34355217	10,26874082	10,09934781	10,15186643	PCP020126	XM_009368544
PCP020129	9,781359714	9,529430554	9,49251418	9,488502959	PCP020129	XM_009368554
PCP020132	10,44742422	10,30720081	10,36120908	10,18570552	PCP020132	XM_008386904
PCP020137	9,383704292	9,489185654	9,379378367	10,50845837		
PCP020138	14,65476635	15,12286843	15,40659267	15,92101966		
PCP020143	9,806275718	9,641455713	9,867788381	10,47099435	PCP020143	XM_009378188
PCP020156	8,780277286	8,73470962	8,84862294	9,177419538		
PCP020157	10,42940674	10,29844067	10,4415644	10,15186643		
PCP020171	10,96674336	10,91488588	10,95855272	10,75933063		
PCP020174	12,59346816	12,35351352	12,20965745	12,50696951	PCP020174	XM_009361980
PCP020190	11,23481743	11,27670602	11,38009845	11,27068661	PCP020190	XM_009344688
PCP020191	9,312882955	9,447083226	9,439477697	9,780818601	PCP020191	XM_018644424
PCP020224	10,17825402	10,28924659	10,51897765	11,09737377	PCP020224	XM_009349570
PCP020228	10,91811785	10,66562905	10,7851827	10,87036472	PCP020228	XM_009346705
PCP020240	10,43844881	10,42661071	10,58402294	11,05505869	PCP020240	XM_009356808
PCP020247	9,336885873	9,148044344	8,658211483	9,40017499	PCP020247	XM_008373650
PCP020248	8,759322309	8,545620932	8,84024291	9,220789431		
PCP020256	9,996713565	10,45327063	10,48616127	10,83788807		
PCP020285	11,44363255	11,48230378	11,64865867	11,87741068		
PCP020292	9,960001932	10,19311874	10,30910114	10,66799854	PCP020292	XM_009370168
PCP020304	9,722807531	9,893301531	9,842868866	9,833411948	PCP020304	XM_009338999
PCP020336	8,780277286	8,651051691	8,865207434	9,445697739	PCP020336	XM_008371416
PCP020347	9,652844973	9,747639151	9,825483551	9,680060579	PCP020347	XM_009355653
PCP020358	12,42574042	12,0175044	12,18817774	12,1233681	PCP020358	XM_009379811
PCP020372	9,123267853	9,200481794	9,445697739	9,832367892	PCP020372	XM_009349512
PCP020374	9,039823818	8,652235507	9,072347016	9,330916878	PCP020374	XM_009346796
PCP020380	10,4118605	10,50680344	10,67918977	10,85823749	PCP020380	XM_009360512
PCP020410	9,286950435	9,186535222	9,195569065	9,138706975	PCP020410	XM_009360470
PCP020446	9,121533517	9,260519709	9,225617167	9,051671182	PCP020446	XM_009380456
PCP020448	10,6489597	10,76901132	10,80332392	10,62113611	PCP020448	XM_009380449
PCP020452	9,719953466	9,55522181	9,581200582	9,547530803		
PCP020464	9,061587209	9,214319121	9,382991408	9,689421477	PCP020464	XM_008391410
PCP020491	11,60501645	11,77409931	11,79319686	11,67198327		
PCP020538	8,857980995	8,924812504	9,039823818	9,361943774	PCP020538	XM_009348536
PCP020544	10,83552148	10,89986291	10,87983593	10,53818893	PCP020544	XM_009341761
PCP020545	12,707646	12,84143265	12,99888479	13,19419204		
PCP020577	12,38685135	12,05889482	12,17679648	12,32192809	PCP020577	XM_008391444
PCP020615	9,695228291	10,04302728	10,1514352	10,68766437		
PCP020663	9,244768161	9,124974266	9,306061689	9,683573652		
PCP020749	10,5372184	10,23002044	10,37648358	10,81244207		
PCP020843	9,311362334	9,215945486	9,357552005	9,623277955		
PCP020848	8,832890014	10,48482289	9,479113318	8,462175047	PCP020848	XM_009336163
PCP020855	9,578051847	9,554588852	9,908392621	11,81244608		
PCP020856	10,6709483	10,72735041	11,1271367	11,6446089	PCP020856	XM_009381159
PCP020868	9,329415156	8,742578916	8,730198386	8,976334992	PCP020868	XM_009379561
PCP020872	10,25423735	10,33016621	10,33315535	10,67918977	PCP020872	XM_009378982

PCP020873	11,49918317	11,14189638	11,32867493	11,8753654	PCP020873	XM_009378653
PCP020889	8,832890014	8,788979283	8,820178962	9,236803511	PCP020889	XM_009376551
PCP020914	10,25187292	10,20783186	10,35204343	10,82151093	PCP020914	XM_008352581
PCP020917	8,541754876	8,692336525	8,955155849	9,288473922		
PCP020925	8,969616759	8,947403166	9,131856961	9,422758607		
PCP020926	10,1581931	9,959523851	9,252665432	8,06786455	PCP020926	XM_008361775
PCP020933	9,929258409	9,78026088	9,730758817	10,04667385	PCP020933	XM_008368734
PCP020934	8,932716728	9,203763987	9,06608919	8,939579214	PCP020934	XM_008390843
PCP020954	10,72649918	10,65582585	10,76735685	11,26795708		
PCP020956	10,45498809	10,31250862	10,48817153	10,71938882		
PCP020957	9,054387253	9,210257347	9,367043381	9,290018847		
PCP020963	8,744833837	8,901862467	8,895817605	8,86727874	PCP020963	XM_008390878
PCP020985	8,863164132	8,758223215	8,98205238	9,202931899	PCP020985	XM_009375957
PCP021013	9,382991408	8,97441459	9,16824589	9,039823818	PCP021013	XM_008341341
PCP021038	11,69667635	11,53770375	11,5256823	11,39124359	PCP021038	XM_009353230
PCP021043	8,857980995	9,207819661	9,346513733	9,898344415	PCP021043	XM_009353236
PCP021047	10,65731845	10,68737568	10,86521517	11,45069577		
PCP021062	9,129283017	9,498510178	9,796575043	10,36377339		
PCP021064	9,055282436	9,150585062	9,086587411	9,45532722	PCP021064	XM_009337889
PCP021066	11,89190898	11,99659	12,04712391	12,27428205	PCP021066	XM_009337888
PCP021078	9,217545787	9,026053072	9,273795599	9,674192268		
PCP021098	8,859006685	8,995286644	8,891783703	8,675957033		
PCP021113	9,787902559	9,931727372	9,899356923	9,849670939	PCP021113	XM_009365481
PCP021120	10,16616308	10,18652284	9,980139578	10,29039906		
PCP021123	9,746514321	9,009352771	9,064284694	9,470313155		
PCP021155	8,718806834	8,938609256	8,865207434	8,798730993	PCP021155	XM_009337265
PCP021165	9,494515616	9,395898244	9,612260337	9,481799432	PCP021165	XM_009337254
PCP021168	8,829722735	8,844454703	9,032514148	9,325687724	PCP021168	XM_009337239
PCP021174	8,680640826	8,780277286	8,90288758	9,611024797	PCP021174	XM_009337234
PCP021185	8,810571635	9,035266491	9,153982623	9,298452123	PCP021185	XM_009344285
PCP021189	8,550746785	8,77478706	8,98205238	9,122388003	PCP021189	XM_009344293
PCP021201	9,690574236	9,239192751	9,383704292	8,861086906	PCP021201	XM_008364739
PCP021215	11,44086917	11,37974116	11,4604559	10,97393409	PCP021215	XM_008364811
PCP021216	10,06249143	10,04575966	10,24237623	10,52975264		
PCP021237	8,978224236	8,784634846	8,812722827	9,211888295		
PCP021238	9,93516505	9,745959978	9,662365743	10,03572012	PCP021238	XM_018646514
PCP021245	8,475733431	8,636624621	9	9,273026268	PCP021245	XM_017325138
PCP021268	9,244768161	9,398038202	9,524874682	9,941531848	PCP021268	XM_008342360
PCP021274	10,23401505	10,44224893	10,57080444	10,9186173	PCP021274	XM_008342368
PCP021282	9,586220553	9,64385619	9,569229039	9,994820116	PCP021282	XM_008371445
PCP021286	9,208648936	9,059804103	9,241578042	9,114601277	PCP021286	XM_008370110
PCP021287	10,4807902	10,53915881	10,67330908	10,86056713		
PCP021297	11,17471975	11,2984464	11,15122589	11,14826684		
PCP021311	9,372146798	9,54303182	9,612260337	9,841297012		
PCP021313	8,642665475	8,947403166	9,412209922	10,15313398		
PCP021317	9,14890869	9,21916852	9,170751309	9,084808388	PCP021317	XM_009345554

PCP021322	10,27999355	9,930737338	10,20007713	10,35718909	PCP021322	XM_009345592
PCP021329	9,869855987	9,806275718	9,911391988	10,23481743	PCP021329	XM_009345652
PCP021356	10,71080643	10,58025857	10,67360059	10,64144668	PCP021356	XM_008377327
PCP021360	9,19063955	9,298452123	9,383704292	9,630576566		
PCP021362	13,3025918	13,11715606	13,28583566	13,4467822	PCP021362	XM_008377335
PCP021367	10,29653771	10,490179	10,7626561	10,87830386		
PCP021370	8,912889336	9,079484784	9,172427509	9,535275377		
PCP021375	11,01842971	11,04871164	11,14656868	11,0622688		
PCP021379	10,4401736	10,50051328	10,70102049	10,8917837	PCP021379	XM_009347643
PCP021393	9,475733431	9,351668078	9,441554026	9,361220052	PCP021393	XM_009335469
PCP021399	9,984888362	10,10547642	10,10109587	10,46011797		
PCP021400	8,820178962	8,660602089	8,897845456	9,114601277	PCP021400	XM_009335459
PCP021410	10,37032778	10,42066556	10,40620501	10,33240585	PCP021410	XM_009335444
PCP021420	9,074141463	9,731319031	10,1378493	11,00655751	PCP021420	XM_009370256
PCP021421	8,921840937	9,169098221	9,353874607	10,02375435	PCP021421	XM_009376938
PCP021428	10,16574867	9,950308015	10,18941689	10,44674215	PCP021428	XM_008376025
PCP021441	9,645063941	9,565425815	9,613476402	9,489185654	PCP021441	XM_017335026
PCP021472	13,33841188	13,05081286	13,12933615	13,30985574		
PCP021473	9,25502857	9,362667132	9,861086906	10,52323849	PCP021473	XR_664527
PCP021476	9,745959978	9,670656249	9,855911856	10,20741705	PCP021476	XM_009338575
PCP021477	9,832890014	9,678899386	9,52291495	9,483150695	PCP021477	XM_008388846
PCP021492	9,546257836	9,819109312	9,812177306	10,15650027	PCP021492	XM_009372314
PCP021503	8,811631578	9,146365017	9,123267853	9,188168462	PCP021503	XM_009372173
PCP021522	11,32717648	10,8008999	11,22821744	11,77190208	PCP021522	XM_008348384
PCP021531	8,657032562	8,685344507	8,873444113	9,506466274	PCP021531	XM_008369041
PCP021535	9,958074154	9,477090385	9,777534785	9,693486957	PCP021535	XM_018643483
PCP021544	9,041659152	9,131008067	9,039823818	8,985841937		
PCP021566	9,588096205	9,318158642	9,559702114	9,482465137	PCP021566	XM_009339238
PCP021600	9,331655958	9,335390355	9,449829585	9,769837844		
PCP021611	10,41785251	10,53267735	10,436368	10,64415371	PCP021611	XM_009378452
PCP021626	9,888240957	10,1438041	10,21634573	10,03525274	PCP021626	XM_009359465
PCP021650	8,129283017	8,63542792	10,14040802	9,191478757	PCP021650	XM_009377294
PCP021654	9,699867382	9,923327485	9,906890596	9,581821975	PCP021654	XM_009353798
PCP021657	9,624484772	9,637838439	9,701601036	9,640244936		
PCP021660	8,944477815	8,921840937	9,099794885	9,577428828	PCP021660	XM_009366681
PCP021679	9,28925812	9,723370841	10,11156607	10,169925	PCP021679	XM_018647055
PCP021687	9,19802752	9,136145235	9,104153166	9,083027168	PCP021687	XM_009356110
PCP021705	10,11113567	10,31816994	10,28848546	10,14507876		
PCP021706	10,34503926	10,45976971	10,38010386	10,45771945		
PCP021717	10,67419227	10,516025	10,74118887	11,09627371		
PCP021719	9,334653186	8,767091963	8,851749041	8,770399609	PCP021719	XM_009351671
PCP021728	10,58777752	10,87061902	10,98726401	10,75572215	PCP021728	XM_009368322
PCP021730	8,120652609	8,913876736	9,323437626	9,282323962	PCP021730	XM_008354953
PCP021763	10,44570808	10,3840606	10,38873845	10,34836301	PCP021763	XM_018647887
PCP021776	12,13677649	11,89228856	11,99929539	12,22590933	PCP021776	XM_009366215
PCP021781	9,192292814	9,288473922	9,388017285	9,760437201		

PCP021782	9,571126898	9,647458426	9,536577492	9,485829309	PCP021782	XM_009366226
PCP021785	9,5980525	9,487176804	9,523561956	9,333893285	PCP021785	XM_018649207
PCP021796	11,85901834	11,72465453	11,46250227	11,54222419	PCP021796	XM_009364856
PCP021800	8,518338423	8,931712601	9,019590728	8,916864735		
PCP021802	9,357552005	9,171602161	9,28077077	9,234410306		
PCP021806	8,335390355	9,085684897	9,155653112	11,25306153	PCP021806	XM_008371654
PCP021811	9,759338956	9,831307244	9,911391988	10,54624819	PCP021811	XM_009340478
PCP021822	11,02744299	11,09275714	11,06204614	10,86959384		
PCP021824	8,739206792	8,732438807	8,844454703	9,33911511	PCP021824	XM_009350345
PCP021832	10,47742436	10,37648358	10,95951661	11,90626304		
PCP021842	10,4101015	10,45704223	10,49485558	10,8008999		
PCP021844	9,153121312	9,172427509	9,17990909	9,008428622		
PCP021845	9,71596199	9,711374448	9,84757418	10,23801057	PCP021845	XM_008371977
PCP021852	10,10328781	9,699867382	9,710806434	9,314401976	PCP021852	XM_008346493
PCP021857	11,57616781	11,51240962	11,65105169	11,86147468		
PCP021874	13,07207732	13,12912199	13,04439412	13,10530781		
PCP021879	10,45463859	10,31477582	10,22077734	10,31552321	PCP021879	XM_009354260
PCP021880	9,45532722	9,636624621	9,742595757	9,906890596		
PCP021893	7,781359714	8,501160452	9,061587209	10,39124359	PCP021893	XM_009345830
PCP021906	9,750422271	9,854354156	9,894817763	10,34687935		
PCP021917	11,30359241	11,37721053	11,44121683	11,23840474		
PCP021921	9,061587209	8,810571635	9,147204925	9,449148645		
PCP021924	9,362667132	9,576163082	9,649848944	9,774243476	PCP021924	XM_009358394
PCP021945	11,23381737	11,07235372	11,23999823	11,53462874	PCP021945	XM_009374936
PCP021952	8,760453835	8,903881846	9,20537785	9,514378734	PCP021952	XM_009335434
PCP021954	9,662365743	9,482465137	9,594324604	9,988684687	PCP021954	XM_009335436
PCP021960	8,872428639	8,751544059	9,191478757	9,245552706		
PCP021968	8,727920455	8,366322214	9,155653112	9,266013461	PCP021968	XM_018645518
PCP021994	9,345028167	9,405141463	9,479780264	9,839203788	PCP021994	XM_008375993
PCP021997	10,3586512	10,04712391	9,675957033	9,451211112		
PCP022000	10,44639065	10,49585503	10,43671154	10,43219737	PCP022000	XM_009363605
PCP022004	9,532687093	9,273026268	9,524874682	9,400879436	PCP022004	XM_008392898
PCP022018	9,819652219	9,67124029	9,891282469	10,11721451		
PCP022024	9,242388143	9,327170869	9,471675214	9,908392621	PCP022024	XM_009339454
PCP022048	12,86953023	13,25660234	13,11829223	13,02531262		
PCP022090	10,19065189	10,4129191	10,42486961	10,54913969		
PCP022106	9,055282436	8,856954575	8,949330653	8,992456886	PCP022106	XM_017331589
PCP022113	10,05165759	9,994820116	10,02883157	10,63511045	PCP022113	XM_018652344
PCP022123	9,82707272	9,855911856	10,04302728	10,50680344		
PCP022143	8,986780664	8,820178962	9,086587411	9,578051847	PCP022143	XM_009352758
PCP022151	9,805743872	9,628135642	9,702172685	9,600526229	PCP022151	XM_009378667
PCP022159	9,305309377	9,707359132	9,477758266	9,255807556	PCP022159	XM_018643121
PCP022161	9,664145025	9,689421477	9,561612539	9,696967526	PCP022161	XM_008233837
PCP022164	9,944960906	10,01448083	10,06025009	10,43671154	PCP022164	XM_018651421
PCP022166	9,015888903	8,952246191	9,111996335	9,498510178		
PCP022172	10,54399845	10,29768437	10,47809209	10,57175264	PCP022172	XR_001952737

PCP022186	11,70289558	11,82031062	11,92382142	12,08148344	PCP022186	XM_008364211
PCP022327	9,320416982	9,383704292	9,125852541	9,305309377	PCP022327	XM_018644914
PCP022404	9,064284694	8,932716728	9,015888903	9,003742767		
PCP022526	10,15945511	10,13228122	10,40229796	10,64054321	PCP022526	XM_009343085
PCP022531	10,45224124	10,18899675	10,2179577	10,215533	PCP022531	XM_009355521
PCP022537	9,494515616	9,22881869	9,429050993	9,266013461		
PCP022546	9,8339495	9,962896005	10,38262403	11,0863552	PCP022546	XM_009355507
PCP022547	8,91489335	9,083027168	9,207819661	9,504481359		
PCP022553	9,293080746	9,138706975	9,451211112	9,546257836	PCP022553	XM_009355475
PCP022554	10,21148681	10,21674586	10,17201489	10,20741705	PCP022554	XM_009355472
PCP022568	9,62935662	9,682994584	9,719953466	9,682415283	PCP022568	XM_009351762
PCP022573	8,938609256	8,923832563	8,903881846	9,390168956		
PCP022579	9,705062108	10,80197505	8,938609256	9,959031118	PCP022579	XM_009337215
PCP022587	9,28000515	9,202123824	9,171602161	9,203763987	PCP022587	XM_009337261
PCP022594	12,66029234	12,57546125	12,58488501	12,46675711	PCP022594	XM_009337351
PCP022613	10,31741261	10,41855893	10,61256445	10,94982671	PCP022613	XM_009337617
PCP022614	12,46335322	12,27941616	12,49685378	12,77917147	PCP022614	XM_009337598
PCP022617	10,53689798	10,28578365	10,54399845	10,53430288		
PCP022634	9,460107723	9,415044544	9,385862401	9,339850003	PCP022634	XM_018647277
PCP022637	8,75598914	9,023283079	9,145499145	9,436024381		
PCP022658	10,88620717	10,72792045	10,6709483	10,64925618		
PCP022660	8,9905862	8,835008208	8,997179481	9,37721053		
PCP022685	8,917879246	9,002815016	9,124121312	9,789533645	PCP022685	XR_001954829
PCP022710	10,19105921	10,25148241	10,14338321	10,34170783	PCP022710	XM_008355136
PCP022713	9,332417038	9,321928095	9,749869427	10,4841482	PCP022713	XM_009338182
PCP022760	10,46794173	10,03204573	10,30263892	9,943496494	PCP022760	XM_018643576
PCP022763	10,39732167	10,26248249	10,5849625	10,99976752	PCP022763	XM_008388186
PCP022778	9,449148645	9,242388143	9,572378118	9,642051693	PCP022778	XM_017328894
PCP022787	9,037080165	9,35974956	8,967716202	9,461479447	PCP022787	XM_009339364
PCP022819	10,19475685	9,498510178	9,63540978	9,314401976		
PCP022826	9,348728154	9,188168462	9,000929563	8,869347076	PCP022826	XM_009346332
PCP022827	9,850702552	9,92381771	10,05392588	10,41925445	PCP022827	XM_009346323
PCP022830	14,42757475	14,17086395	13,98684909	14,27236224		
PCP022837	10,10809315	9,814310082	9,97441459	10,22360355		
PCP022851	11,52780441	11,00398866	10,98845037	11,16407098	PCP022851	XM_009346078
PCP022853	10,65224447	10,3527828	10,18570552	10,48146646	PCP022853	XM_009346029
PCP022854	9,266786541	8,995286644	8,792237182	8,868297843	PCP022854	XM_009346040
PCP022865	8,554588852	8,705044823	9,001886668	9,604238155	PCP022865	XM_008355302
PCP022886	9,475733431	9,414325838	9,408690635	9,356826076		
PCP022892	9,769292392	9,77807713	9,979182225	10,4419067	PCP022892	XM_008374978
PCP022894	8,835008208	9,1807297	9,307587905	9,522248035	PCP022894	XM_009368075
PCP022930	10,1476247	10,15059775	10,39802751	10,5943246		
PCP022939	10,35571448	10,3140167	10,42906146	10,76845729		
PCP022952	9,99859043	9,835529375	10,17533747	10,31023786	PCP022952	XM_009367929
PCP022953	9,433230722	9,068778278	9,225617167	9,505156537	PCP022953	XM_009367921
PCP022959	9,915386449	9,974887762	9,996713565	10,02468259	PCP022959	XM_009367906

PCP022963	11,15228484	11,41802651	11,37847369	11,59851781	PCP022963	XM_009367893
PCP022964	12,96253456	12,58980795	12,75175325	12,57285889	PCP022964	NM_001328820
PCP022985	10,01820018	10,39518065	10,58777752	10,80063301		
PCP022986	9,962896005	9,892284763	9,775330438	9,478425839		
PCP023012	9,77807713	9,562242424	9,605479518	9,671841781	PCP023012	XM_008376567
PCP023043	9,571126898	9,471675214	9,527477006	9,978710459	PCP023043	XM_009378085
PCP023046	12,85168463	12,93030676	13,11347033	13,52278625		
PCP023057	9,913891691	10,01168851	10,02606691	10,0370939		
PCP023065	10,23601419	10,05572982	10,08347933	10,05979059		
PCP023077	9,426264755	9,646252679	10,00235092	10,61746747	PCP023077	XM_008367925
PCP023078	9,262870027	9,225617167	9,543669869	9,503825738		
PCP023081	9,20537785	9,153982623	9,078604498	9,411510988		
PCP023095	11,55506839	11,3705076	11,34614249	11,40567333	PCP023095	XR_001953634
PCP023100	9,966750567	10,03296865	10,36011184	10,63087684		
PCP023120	10,31741261	9,860574891	10,25777675	10,40479032	PCP023120	NM_001294044
PCP023142	10,60547952	10,30947635	9,972505988	10,15566576	PCP023142	XM_018643388
PCP023146	9,912889336	9,770383089	9,762664404	9,913891691	PCP023146	XM_009340122
PCP023148	9,852264198	9,815383296	10,07369975	10,306825	PCP023148	XM_009340120
PCP023149	11,40247917	11,42433953	11,35810171	11,51389041	PCP023149	XM_009340118
PCP023164	9,219967107	9,203763987	9,397310972	9,656424863	PCP023164	XM_009356064
PCP023197	9,903881846	10,10459875	10,38513979	11,11525828		
PCP023210	9,189009107	9,21916852	9,303027245	8,95419631		
PCP023216	10,9427564	10,69841818	10,78408678	10,7428736		
PCP023237	9,178241565	8,920858975	8,77478706	8,925791779	PCP023237	XM_017334664
PCP023242	10,28231238	9,305309377	9,120678526	9,658211483	PCP023242	XM_008367335
PCP023245	11,02005626	10,83762793	10,85772446	11,12498073	PCP023245	XM_008359766
PCP023246	9,380093039	9,699867382	9,943496494	10,35571448	PCP023246	XM_007216948
PCP023247	8,957102042	8,97441459	9,013099307	9,531381461	PCP023247	XM_009348303
PCP023254	12,0023474	12,26766454	12,22951883	12,28905923		
PCP023262	9,269898093	9,237616296	9,29156212	9,628755372		
PCP023263	9,167418146	9,170751309	9,275356315	9,839203788	PCP023263	XM_009363892
PCP023277	9,136991112	9,41715631	9,411510988	9,867788381	PCP023277	XM_009357275
PCP023282	10,12799432	10,24397129	10,65403627	11,30187911	PCP023282	XM_009357346
PCP023284	9,247927513	9,685326985	9,222384371	9,488502959	PCP023284	XM_009357360
PCP023291	8,875503635	9,019590728	9,099794885	9,321184013		
PCP023296	11,31797782	11,19332206	11,45755018	11,08414401	PCP023296	XM_009349800
PCP023322	10,05979059	9,691167722	9,940063949	9,818582177		
PCP023324	8,922822231	8,79983204	8,861086906	8,854868383		
PCP023331	9,388727692	9,488502959	9,551381432	10,12498719	PCP023331	XM_009342697
PCP023333	9,807886174	9,683573652	9,785730354	9,769292392	PCP023333	XM_018642666
PCP023352	10,39695792	10,31364267	10,25581936	10,13656824	PCP023352	XM_009364388
PCP023411	8,790087794	8,713111526	8,742578916	9,178241565		
PCP023415	8,885696373	9,236803511	9,203763987	9,67124029	PCP023415	XM_008341749
PCP023429	9,659995892	9,609788199	9,802516365	10,28540222		
PCP023450	9,19640541	9,25502857	9,406566431	9,8159116	PCP023450	XM_008341631
PCP023460	9,182394353	9,235224441	9,240791332	9,20537785	PCP023460	XM_018645204

PCP023464	13,00457162	12,83880999	12,94416299	12,86611979	PCP023464	XM_009356400
PCP023476	9,293080746	9,18157469	9,369226538	9,850186838		
PCP023482	10,35901375	10,31174832	10,20252792	9,930249459	PCP023482	XM_009366393
PCP023483	8,730198386	9,189009107	9,133578937	9,621447519	PCP023483	XR_530846
PCP023503	9,124974266	8,826548487	9,123267853	8,932716728		
PCP023523	8,951284715	8,998110862	8,90288758	8,87036472	PCP023523	XM_009348741
PCP023541	9,687673116	9,184875343	9,424166289	9,330177419		
PCP023542	9,6171552	9,473035989	9,636026394	9,927777962	PCP023542	XM_017323651
PCP023545	10,90111579	10,86984828	10,97704375	11,45618498	PCP023545	XM_009359793
PCP023553	11,2772874	10,98108916	11,47150757	11,84261356	PCP023553	XM_008362483
PCP023574	9,193106413	9,151447887	9,344295908	9,565425815		
PCP023598	9,563520437	9,766528909	9,744833837	10,12885787	PCP023598	XM_009362017
PCP023599	11,30587365	11,36303963	11,241584	11,53996816	PCP023599	XM_009362018
PCP023623	10,64175373	10,80600982	10,98322878	11,08126372		
PCP023658	9,398743692	9,433940073	9,447083226	9,070577167		
PCP023679	8,63542792	8,710806434	8,74708524	9,324180547	PCP023679	XM_009348364
PCP023700	8,493174961	8,968666793	8,204571144	10,29385095	PCP023700	XM_009362009
PCP023728	8,971543554	9,194756854	9,21916852	9,07325791	PCP023728	XM_009338475
PCP023744	10,62143836	10,49785184	10,59681404	11,02882466		
PCP023746	8,761551232	8,657032562	8,821231873	9,167418146		
PCP023755	8,95419631	9,048023611	9,142107057	9,47100451	PCP023755	XM_017333788
PCP023763	8,641437649	8,804131021	8,903881846	9,223205318		
PCP023794	9,751544059	9,813252106	9,33315535	9,762664404	PCP023794	XM_009360626
PCP023797	9,221587121	9,468970167	9,419960178	9,720534991	PCP023797	XM_009374049
PCP023823	11,19434442	11,14656868	11,10481495	11,13249973		
PCP023824	9,214319121	9,105908509	9,130132926	9,050746552		
PCP023837	9,118084543	9,251884747	9,279215904	9,593708574		
PCP023975	10,41574177	10,41996018	10,16908569	10,28733146		
PCP024032	8,692336525	8,994353437	8,880593701	8,917879246	PCP024032	XM_009342359
PCP024069	11,42818742	11,85096815	11,76210377	12,00351791	PCP024069	XM_018645319
PCP024094	11,91239164	11,22761594	11,53494966	11,59277002		
PCP024119	9,296916207	9,303027245	9,381542951	9,215945486	PCP024119	XM_009368529
PCP024123	9,537218401	9,68707819	9,818582177	10,14551188		
PCP024140	10,37431138	10,82839834	11,31628153	11,53138146	PCP024140	XM_018649777
PCP024187	11,55347091	11,65672428	11,77643303	12,07012094	PCP024187	XM_008344561
PCP024215	8,437419184	8,602401945	9,033423002	9,828136484	PCP024215	XR_528432
PCP024216	10,22641219	10,2487222	10,15186643	10,11286951	PCP024216	XM_008391541
PCP024220	9,278449458	9,381542951	9,393755108	9,396604781	PCP024220	XM_017337009
PCP024221	9,055282436	9,038013592	9,206208529	9,10938668		
PCP024222	10,08878824	10,4415644	10,64716158	10,78408678		
PCP024237	9,428360173	8,963849777	9,051671182	8,689997971	PCP024237	XM_008391513
PCP024241	9,095397023	9,276124405	8,911901261	8,694636474	PCP024241	XM_008391510
PCP024243	9,14381685	9,214319121	9,299208018	9,901364723	PCP024243	XM_008391508
PCP024247	12,2125967	12,19249934	12,09781158	11,87945689	PCP024247	XM_008391501
PCP024256	9,86004717	9,787902559	9,884170519	10,31778568		
PCP024265	9,552669098	9,342808055	9,449148645	9,481799432	PCP024265	XM_008388394

PCP024284	8,696967526	8,828676005	8,95805965	9,462849868	PCP024284	XM_017335976
PCP024306	10,68533575	8,73470962	10,09275714	11,18900293	PCP024306	NM_001302313
PCP024307	11,22961796	10,74174505	11,42713996	11,5926112	PCP024307	XM_009378121
PCP024326	8,95419631	9,018673116	9,216745858	9,102421932	PCP024326	XM_008390091
PCP024333	10,65284497	10,66681871	10,74118887	11,16427844	PCP024333	XM_009362605
PCP024335	8,951284715	8,908902953	9,047123912	9,479780264		
PCP024373	10,46318716	10,69551541	10,66533592	10,56921954	PCP024373	XM_009358772
PCP024382	14,58453214	14,5773108	14,65964309	14,52454172	PCP024382	XM_009358786
PCP024388	9,19640541	9,58370335	9,379378367	9,724513853	PCP024388	XM_008349892
PCP024405	9,026053072	8,873444113	9,028817757	9,471675214		
PCP024406	10,82469626	10,45293102	10,78490471	10,16783208	PCP024406	XM_009343749
PCP024418	10,84051831	10,82151093	10,85096815	10,80224977		
PCP024425	10,52029455	10,18611424	10,2644426	10,49418557		
PCP024429	10,80950287	11,04848687	11,31571566	11,77533455	PCP024429	NM_001294022
PCP024435	9,223205318	9,199672345	9,199672345	9,537859025	PCP024435	XM_008372461
PCP024455	12,00714293	11,97894994	12,10558118	11,87305955	PCP024455	XM_018651591
PCP024497	8,779161208	8,703903573	9,053464362	8,985841937		
PCP024515	8,687655621	8,963849777	9,159871337	9,488502959		
PCP024517	9,652844973	9,288473922	9,493855449	9,42485912	PCP024517	XM_008379173
PCP024522	9,645063941	9,451891079	9,879077758	10,25423735	PCP024522	XM_009367304
PCP024573	10,35461304	9,97441459	10,28771238	10,31967212		
PCP024574	9,678899386	9,682415283	9,63964821	9,979182225	PCP024574	XM_009337545
PCP024596	8,41502341	8,566054038	8,884689491	9,682415283	PCP024596	XM_009335370
PCP024610	9,602383386	9,685326985	9,941047606	10,41433641	PCP024610	XM_018646363
PCP024613	10,94251451	10,99293834	10,97010589	11,28674834	PCP024613	XM_009367819
PCP024630	10,35828856	10,6329952	10,68999797	10,93492188	PCP024630	XM_009341660
PCP024647	9,148044344	9,008428622	9,045295522	8,886702554	PCP024647	XM_009369455
PCP024655	9,248710343	9,047123912	9,048949988	9,222384371	PCP024655	XM_009369469
PCP024656	8,940548521	9,118084543	9,329415156	9,829722735	PCP024656	XM_009369470
PCP024663	8,932716728	8,680640826	8,85277917	9,251884747		
PCP024664	10,76597392	10,70534728	10,80251637	10,62235387		
PCP024687	7,872397856	9,390878304	10,19352536	12,38927371	PCP024687	XM_018649522
PCP024698	8,971543554	9,033423002	9,113742166	8,998110862	PCP024698	XM_009365641
PCP024706	8,97441459	8,797661526	9,211888295	9,019590728	PCP024706	XM_009365657
PCP024728	10,63390341	10,6399466	10,74679141	11,25049384		
PCP024753	11,65060302	11,48011867	11,49419057	11,62738341	PCP024753	XM_009375416
PCP024755	11,73216743	11,76639225	11,69101501	11,80264964		
PCP024756	9,338357556	9,760437201	9,415044544	9,826548487	PCP024756	XM_009352080
PCP024758	10,12153352	10,05483491	10,0370939	10,07236042	PCP024758	XM_009352079
PCP024777	9,841831634	10,00235092	10,04712391	10,2467406	PCP024777	XM_009369813
PCP024786	9,984888362	9,77148947	9,987732989	9,952260754	PCP024786	XM_009341572
PCP024791	10,37685252	10,57774037	10,74707685	10,23242093		
PCP024809	8,517000043	8,857980995	8,948367232	9,743706817		
PCP024827	9,476401941	9,471675214	9,185693134	8,511752654		
PCP024828	8,937609223	9,038918989	9,206208529	9,405141463	PCP024828	XM_009356802
PCP024835	9,232828614	9,023283079	8,908902953	8,95419631		

PCP024857	8,904875427	9,027905997	8,960001932	8,810571635	PCP024857	XM_009361256
PCP024865	9,837107523	9,463524373	9,537218401	9,407267764	PCP024865	XM_008395297
PCP024889	9,247144259	8,804131021	8,779161208	8,631795481	PCP024889	XM_008352647
PCP024900	9,006550496	9,035266491	9,254249162	9,094526396	PCP024900	XM_009374845
PCP024911	9,174925683	9,142949447	9,262094845	9,5980525	PCP024911	XM_009349769
PCP024918	10,83973131	10,81831854	10,92703717	11,39499851	PCP024918	XM_009374705
PCP024933	10,88899433	10,7153872	10,73358313	10,85642553	PCP024933	XM_009371692
PCP024936	8,714245518	9,284639053	9,059804103	9,128432607	PCP024936	XM_009349492
PCP024958	12,25748508	11,79007557	11,73033002	12,36768776		
PCP024959	9,63481105	9,004698008	9,153121312	9,034331283	PCP024959	XM_009365919
PCP024971	10,47606772	10,42661071	10,24911345	10,33650656		
PCP024976	9,648051932	9,589969422	9,396604781	9,317412614	PCP024976	XM_009367332
PCP024981	9,188168462	9,123267853	9,406566431	9,562872035	PCP024981	XM_009353893
PCP024995	9,105908509	9,105908509	9,14381685	9,101529272	PCP024995	XM_009381171
PCP025001	9,557789157	9,350939182	9,387306529	9,06250492	PCP025001	XM_009381157
PCP025002	10,40974096	10,75014588	10,53850906	10,23041679	PCP025002	XM_018652474
PCP025016	9,146365017	9,08127038	9,06520069	8,872428639	PCP025016	XM_009370504
PCP025039	11,77300317	11,57884439	11,86057101	12,40771123	PCP025039	XM_009358308
PCP025043	9,150585062	9,130132926	8,89684702	8,757123282	PCP025043	XM_009358294
PCP025050	11,30701292	11,30168481	11,30624971	11,30168481		
PCP025057	9,448467384	9,82336724	10,14678503	10,57617253	PCP025057	XM_009346318
PCP025073	9,454648871	9,364134655	9,615942233	9,944463173	PCP025073	XM_009363420
PCP025079	11,03181835	10,94397991	10,88289519	11,41943355	PCP025079	XM_009347225
PCP025100	9,875488276	9,790071499	10,07190476	10,41468524	PCP025100	XM_009377195
PCP025113	8,788979283	9,160703421	9,536577492	10,13869418	PCP025113	XM_009361765
PCP025125	9,892800824	9,54689446	9,336127147	9,22881869	PCP025125	XM_009362546
PCP025144	9,557789157	9,426956579	9,525520809	9,537859025		
PCP025169	10,95274125	11,15924065	11,05166439	11,4446681	PCP025169	XM_008345047
PCP025186	9,01402047	9,300741498	9,652844973	9,701011827		
PCP025189	9,956129273	10,56700537	10,14551188	10,37503943	PCP025189	XM_009354534
PCP025205	11,64054773	11,73442596	11,67830523	11,72593283	PCP025205	XM_008371467
PCP025210	10,11113567	10,18652284	10,01726776	9,936637939	PCP025210	XM_009365746
PCP025225	14,10800737	13,76248586	13,87331626	14,15426434		
PCP025260	9,603014275	9,513727596	9,612260337	9,552035018	PCP025260	XM_009339807
PCP025272	9,971543554	10,0274499	10,02928723	9,990103964	PCP025272	XM_018646246
PCP025321	8,790087794	8,891783703	8,999069838	8,849655303	PCP025321	XM_008231497
PCP025338	10,64565843	10,56541629	10,61777048	10,91936243		
PCP025357	9,016808288	9,048023611	9,06520069	9,017727086	PCP025357	XM_008374939
PCP025373	9,705632387	9,914878405	9,803049401	9,917864332	PCP025373	XM_018648248
PCP025380	9,82124782	9,88519302	9,872413248	9,910387894	PCP025380	XM_008361033
PCP025387	10,43740878	10,47573343	10,53786873	10,4307975	PCP025387	XM_018645899
PCP025403	10,17700835	9,82707272	10,05934446	9,742023058	PCP025403	XM_008377802
PCP025440	10,34429591	10,3586512	10,47539906	10,40479032		
PCP025461	10,01029032	9,927289081	10,1275688	9,99859043	PCP025461	XM_009344095
PCP025462	9,872936465	9,68474862	9,69406183	10,603005	PCP025462	XR_002271563
PCP025476	8,406545173	8,735826768	9,372146798	9,272233193		

PCP025495	10,45635442	10,47032332	10,42451275	10,78299821	PCP025495	XM_008370786
PCP025508	9,14381685	10,26405548	10,81644771	14,02502447	PCP025508	XR_668418
PCP025509	8,856954575	8,901862467	8,943481842	9,380828997	PCP025509	XM_009369638
PCP025674	9,913891691	10,28578365	9,553955615	10,05619061	PCP025674	XM_009367378
PCP025752	13,5419835	13,60002903	13,71978132	13,91210997	PCP025752	XM_009336070
PCP025757	10,215533	10,16028744	10,33687472	10,59089576		
PCP025764	10,41750445	10,00468396	9,914878405	10,12240095	PCP025764	XM_009336190
PCP025773	11,29920802	10,86134285	11,26795708	11,15334302	PCP025773	XM_009336400
PCP025774	9,811647632	9,770944642	9,803598384	10,14551188		
PCP025794	10,033423	9,725076497	9,713678633	9,654636029	PCP025794	XM_009336604
PCP025799	10,66059318	10,85564717	10,84836473	10,92654062	PCP025799	XM_009336652
PCP025803	9,009352771	9,301496195	9,343563277	9,203763987	PCP025803	XM_008368526
PCP025816	8,875503635	8,75598914	8,917879246	9,200481794	PCP025816	XM_009336928
PCP025826	11,21208899	11,58527712	11,37666806	11,18920683	PCP025826	XM_009359025
PCP025832	9,100662339	9,215945486	9,579938142	9,600526229	PCP025832	XM_009380502
PCP025843	13,05081286	13,11645281	13,27491244	13,77591887	PCP025843	XM_008372488
PCP025849	8,632995197	8,775873612	8,715378619	9,297672907	PCP025849	XM_009339333
PCP025857	9,916372141	10,24990749	10,2495046	10,31023786		
PCP025858	9,618991087	9,334653186	9,574271841	9,552669098		
PCP025874	9,690574236	9,895832748	10,12627857	10,35681507		
PCP025898	10,34872815	10,69841818	10,65702363	10,86521517		
PCP025900	11,46743743	11,53948357	11,34374092	11,25384748		
PCP025902	10,31665489	10,17533747	10,39660478	10,57522723		
PCP025908	10,22641219	9,983948403	10,19311874	10,14847658	PCP025908	XM_017336066
PCP025910	9,925302224	9,894317583	10,033423	10,42381975	PCP025910	XM_017331992
PCP025915	8,426264755	8,541754876	8,890781061	10,65850606	PCP025915	XM_017336537
PCP025923	10,11504365	10,45704223	9,845490051	10,58683979		
PCP025924	9,991521846	10,05437369	10,13528603	10,51175265		
PCP025934	10,12240095	10,05256805	10,20661148	10,63511045	PCP025934	XM_009364752
PCP025935	10,436368	10,50647619	10,79658315	11,20660538	PCP025935	XM_009364748
PCP025941	10,64565843	10,57931594	10,66917739	10,52323849	PCP025941	XM_009364740
PCP025942	11,15629169	10,99529371	11,18218948	11,6414512	PCP025942	XM_009364741
PCP025974	9,324923085	9,426956579	9,290018847	9,188168462	PCP025974	XM_009358144
PCP025990	10,7808104	10,79901443	10,67624801	10,84392105	PCP025990	XM_009358102
PCP026006	9,604868234	9,582461918	9,644451179	10,10109587	PCP026006	XM_009361114
PCP026051	9,276892087	9,276892087	9,238404739	9,261319247	PCP026051	XM_009354731
PCP026056	11,53786388	11,54673533	11,66666784	11,98903964		
PCP026064	10,4307975	10,3818998	10,33128647	10,2632692	PCP026064	XM_009354757
PCP026096	9,861598739	9,814854798	9,93516505	10,32268306	PCP026096	XM_009340008
PCP026115	8,945443836	8,80841723	8,949330653	9,405843489	PCP026115	XM_009348161
PCP026148	9,864186145	10,06743436	10,3894485	11,13506795	PCP026148	XM_008341716
PCP026149	10,87779792	10,91413843	10,90086681	10,93171999	PCP026149	XM_017323701
PCP026150	9,322671793	9,346513733	9,47370575	9,928770029	PCP026150	XM_008391303
PCP026151	10,14678503	10,38478375	10,58934217	10,8191173	PCP026151	XM_008341712
PCP026158	9,383704292	9,18157469	9,412209922	9,692911856	PCP026158	XM_008391291
PCP026160	8,818038868	8,938609256	8,869347076	8,809510912	PCP026160	XM_018648320

PCP026164	9,398743692	9,19640541	8,659389441	8,620842965		
PCP026171	8,805195705	8,501160452	9,117201525	9,208648936	PCP026171	XM_009354446
PCP026175	8,95805965	8,708497652	9,19640541	9,626311362	PCP026175	XM_018646694
PCP026198	10,12066557	9,984888362	10,42066556	10,43809528	PCP026198	XM_009367479
PCP026202	9,257387843	9,061587209	9,276892087	9,203763987	PCP026202	XM_009367483
PCP026205	10,07592031	9,626311362	9,749869427	9,693486957		
PCP026223	9,158180476	9,05709822	9,058884672	9,136145235	PCP026223	XM_009347791
PCP026241	10,59276535	10,49818105	10,56288157	10,89835197	PCP026241	XM_009345655
PCP026250	9,367786025	9,18157469	9,176597042	9,269126679	PCP026250	XM_009363253
PCP026292	9,931727372	9,628755372	9,726780139	9,626311362	PCP026292	XM_009360277
PCP026297	9,606719814	9,601157931	9,561612539	9,589332801	PCP026297	XM_009360283
PCP026299	9,226412193	9,362667132	9,517019734	9,705062108	PCP026299	XM_009342378
PCP026304	9,104153166	9,238404739	8,979196518	9,235224441	PCP026304	XM_009342388
PCP026305	12,69385063	12,88054013	12,78742716	12,63496302	PCP026305	XM_008338767
PCP026318	8,87857209	8,827596761	8,743723645	9,160703421	PCP026318	XM_018644518
PCP026321	9,45532722	9,553302899	9,55522181	9,451891079	PCP026321	XM_009345287
PCP026335	10,82681063	10,84496463	11,00702727	10,84182377	PCP026335	XM_008375547
PCP026345	8,880593701	8,803065551	8,847589839	9,234410306	PCP026345	XM_008364713
PCP026348	9,426264755	9,567956075	9,598666678	9,574271841	PCP026348	XM_009338597
PCP026355	9,664145025	9,73357466	9,872936465	10,20089861		
PCP026385	9,468277836	9,535275377	9,695802471	9,890264277		
PCP026388	9,206208529	8,901862467	9,293080746	9,042562264	PCP026388	XM_009362759
PCP026399	10,16490693	10,18446627	10,17158965	9,97632067	PCP026399	XM_009362780
PCP026409	9,428360173	9,342074668	9,21916852	9,802516365		
PCP026432	10,64955259	10,8273427	11,02721489	11,06877828		
PCP026452	8,999069838	9,087462841	9,255807556	9,478425839		
PCP026455	9,394462695	9,37286506	9,589969422	9,871397049	PCP026455	XM_009348953
PCP026460	14,54323305	14,13059711	14,40786522	14,68697482	PCP026460	XM_009348866
PCP026472	9,592457037	9,614102459	9,631777296	9,29156212	PCP026472	XM_009374430
PCP026473	9,247144259	9,159871337	9,413627929	9,630576566		
PCP026501	11,90275194	12,13153547	12,20314303	12,85940472	PCP026501	XM_009343151
PCP026527	9,370687407	9,510427928	9,422064766	9,525520809	PCP026527	XM_009359897
PCP026533	10,00235092	10,00562455	9,960001932	9,887723262	PCP026533	XM_009359909
PCP026534	9,787363896	9,6171552	9,862637358	10,03983752		
PCP026564	8,964831792	9,290779173	9,339850003	9,458057945	PCP026564	XM_009367687
PCP026580	11,62753388	11,58057421	11,46539805	11,42486437		
PCP026611	10,34318572	10,480457	10,56319627	10,94128975	PCP026611	XM_009362263
PCP026612	8,918863237	9,024225474	9,09011242	9,693486957		
PCP026623	9,266013461	9,88519302	9,940563202	9,865733271	PCP026623	XM_009362281
PCP026629	9,066977144	9,055282436	9,067891433	8,864186145	PCP026629	XM_008381164
PCP026644	9,448467384	9,387306529	9,439477697	9,916879659	PCP026644	XM_008391621
PCP026654	9,70333261	9,72395099	9,916879659	9,803598384		
PCP026671	10,83051521	10,64385619	10,77478706	10,86238165	PCP026671	XM_008350181
PCP026677	9,367043381	9,472345607	9,611633479	9,845490051		
PCP026697	9,807886174	9,63540978	9,752664976	10,09407769		
PCP026702	9,261319247	9,356826076	9,929746623	10,20741705		

PCP026713	9,315149562	9,476401941	9,689997971	9,603626345	PCP026713	XM_009362344
PCP026792	9,390878304	9,111996335	9,782457711	9,743151394	PCP026792	XM_009370473
PCP026837	8,581200582	8,86727874	9,201315296	10,01634867	PCP026837	XM_009361776
PCP026865	10,97704375	10,95951661	11,14720492	11,46437727	PCP026865	XM_008379799
PCP026881	9,286164981	9,286164981	9,536577492	9,305309377	PCP026881	XM_009345707
PCP026895	10,98394128	11,12045174	11,31420369	11,57884439	PCP026895	XM_008356623
PCP026905	10,66651695	10,5143886	10,54464573	10,88213099		
PCP026917	9,010304306	9,01402047	9,410811715	9,670656249	PCP026917	XM_016792767
PCP026922	9,930249459	9,735268302	9,866243459	9,662365743	PCP026922	XM_009359000
PCP026929	10,29039906	10,47742436	10,32605865	10,5635109	PCP026929	XM_009351410
PCP026932	10,38081818	10,49785184	10,480457	11,01262454	PCP026932	XM_009351431
PCP026935	11,39088368	11,32605303	11,27029533	11,38550114	PCP026935	XM_009367063
PCP026939	9,671841781	9,751544059	9,916372141	10,13271821	PCP026939	XM_018643434
PCP026958	9,402308618	9,445697739	9,764871591	10,017741	PCP026958	XM_008357142
PCP026960	11,60994976	11,82283768	11,59011917	11,30453956		
PCP026966	12,25571611	12,40221267	12,37259303	12,37883671	PCP026966	XM_008365956
PCP026970	10,17783061	10,2765083	10,32755264	11,03043954		
PCP026972	9,540457428	9,581821975	9,633594681	10,01402047		
PCP027002	9,575539247	9,383704292	9,243959396	8,912889336	PCP027002	XM_008366547
PCP027021	15,10612655	15,16477615	15,2306836	15,81264537		
PCP027103	11,72948055	11,5436747	11,58402294	11,88671398	PCP027103	XM_009374437
PCP027125	9,910387894	9,946906274	9,999534994	10,61654884		
PCP027137	8,891783703	9,213517401	9,269898093	9,239192751		
PCP027329	9,079484784	9,075933681	9,341340908	9,72905987	PCP027329	XM_009346823
PCP027338	9,495855027	9,48984796	9,675374899	9,931727372		
PCP027356	9,697541014	9,281535984	9,662970945	9,990572019	PCP027356	XM_009339370
PCP027365	12,94933977	12,73174329	12,75217155	13,12751076	PCP027365	XR_664702
PCP027366	11,28636716	11,47201553	11,43427898	11,71881967		
PCP027373	9,319672121	9,297672907	9,462154593	9,704474311		
PCP027377	9,565425815	9,383704292	9,223205318	9,677719642	PCP027377	XR_001951673
PCP027387	10,21391832	9,905883363	10,05347794	10,09671515		
PCP027402	10,24397129	10,08258817	10,15650027	10,23161721		
PCP027407	8,978224236	9,238404739	9,283088353	9,450530824	PCP027407	XM_009378349
PCP027435	9,499845887	9,512404684	9,62388149	9,932701967	PCP027435	XM_018650116
PCP027439	11,05979734	10,86856794	10,90212635	11,03937833		
PCP027480	9,925806611	9,982523054	9,938594555	9,688827272	PCP027480	XM_009370389
PCP027494	8,810571635	8,918863237	8,807354922	8,84862294	PCP027494	XM_018643807
PCP027504	8,640244936	8,659389441	8,937609223	9,208648936	PCP027504	XM_017326947
PCP027512	10,19229281	10,23281662	10,31590808	10,64385619	PCP027512	XM_018645926
PCP027519	9,010304306	8,87036472	9,18157469	9,312882955	PCP027519	XR_665912
PCP027526	8,807354922	8,87857209	9,053464362	9,251884747	PCP027526	XM_009345390
PCP027533	12,22761594	12,11656165	12,3133564	12,73795077	PCP027533	XM_009345415
PCP027537	11,18900293	11,27980208	11,404785	11,60933113		
PCP027577	12,73336917	12,58480517	13,07347215	13,69877736	PCP027577	XM_009342337
PCP027628	8,949330653	8,824417827	8,895817605	8,805195705	PCP027628	XM_009356066
PCP027636	9,691167722	9,688250309	9,334653186	9,067891433	PCP027636	XM_009340670

PCP027647	9,240004193	9,267559206	9,623277955	9,688250309	PCP027647	XM_009340640
PCP027680	9,392317423	9,327170869	9,507140523	9,412209922	PCP027680	XR_666143
PCP027681	11,1221809	11,07793058	11,32192809	11,29806257		
PCP027682	11,81284714	11,65895674	11,72266667	11,50167301	PCP027682	XM_018645554
PCP027685	9,217545787	9,203763987	9,253445696	9,920352855	PCP027685	XM_008364107
PCP027692	9,292321633	9,136145235	9,158180476	8,887738491	PCP027692	XM_009353230
PCP027694	9,441554026	9,569855608	9,589332801	9,998124969	PCP027694	XM_008390973
PCP027697	10,64955259	10,83051521	11,04074863	11,42818742		
PCP027705	9,139551352	9,284639053	9,374322251	9,292321633	PCP027705	XM_008368764
PCP027711	9,084808388	9,156512913	9,083027168	9,024225474	PCP027711	XM_008390953
PCP027712	8,700439718	8,792237182	9,20537785	9,47100451	PCP027712	XM_008350521
PCP027723	9,128432607	9,566054038	9,539158811	9,735843688	PCP027723	XM_018642907
PCP027724	10,07992473	9,859534786	9,947388554	10,08170978		
PCP027728	9,132705355	9,25502857	9,362667132	9,204571144	PCP027728	XM_009336647
PCP027776	10,18487534	10,27184816	10,27107779	10,25856603		
PCP027780	10,25541812	9,911886285	10,10372055	10,47742436		
PCP027789	9,283088353	9,397310972	9,362667132	9,333893285	PCP027789	XM_009338834
PCP027800	9,826548487	9,895317771	10,02836195	10,46488265	PCP027800	XM_009374281
PCP027845	9,098032083	9,106772296	9,102421932	9,441554026		
PCP027856	12,53503231	12,36714169	12,70080165	12,75968017	PCP027856	XM_009348395
PCP027880	9,928770029	9,933690655	10,26130749	10,20661148		
PCP027888	9,159871337	8,913876736	9,016808288	8,983934156		
PCP027907	11,71152502	11,78149496	11,71881967	11,80681945		
PCP027909	10,13699111	10,01402047	10,17033821	10,51109044	PCP027909	XM_009345837
PCP027924	9,632995197	9,457380879	9,49119171	9,430452552		
PCP027933	8,89684702	9,004698008	9,528121969	9,903881846	PCP027933	XM_009357721
PCP027959	9,111996335	9,140395236	9,020507757	9,405141463	PCP027959	XM_009352614
PCP027964	10,43948809	10,6060998	10,78381676	11,00445223	PCP027964	XM_009345916
PCP027980	8,727920455	9,13442632	9,431142371	10,23082505	PCP027980	XM_009363009
PCP027981	10,07190476	10,17492568	10,19269967	10,15902615		
PCP028002	11,47438027	11,35351628	11,46760555	11,27999935	PCP028002	XM_009356154
PCP028011	9,651051691	9,37721053	9,295378654	8,918863237	PCP028011	XM_018645151
PCP028017	10,89456769	10,86289302	10,92851838	10,9331964		
PCP028020	9,97632067	9,999534994	9,881113961	10,19926745	PCP028020	XM_008354995
PCP028022	9,951765533	9,932214752	9,97441459	10,41855893		
PCP028025	9,186535222	9,215120395	9,365600686	10,00327897	PCP028025	XM_008354989
PCP028028	8,586201784	8,637820329	8,890781061	9,564778269	PCP028028	XM_009355292
PCP028048	9,589332801	9,364134655	9,403715086	9,54239349	PCP028048	XM_018642372
PCP028053	10,02004932	10,05572982	10,17076382	10,5949497	PCP028053	XM_018648327
PCP028058	9,451211112	9,36047402	9,488502959	9,760437201	PCP028058	XM_009350741
PCP028097	11,5888692	11,35259523	11,55378766	11,96313812	PCP028097	XM_009360319
PCP028114	9,571126898	9,523561956	9,640841416	10,07992473	PCP028114	XM_008393191
PCP028135	9,056177063	8,989621566	9,304533858	9,089238597	PCP028135	NM_001328717
PCP028143	13,34669794	13,48947169	13,49047486	13,6129065		
PCP028146	10,04803724	9,578693464	9,68707819	9,353146825	PCP028146	XM_009349821
PCP028177	9,490509963	9,762664404	9,782998209	10,03296865	PCP028177	XM_009377065

PCP028194	8,956143797	8,495855027	8,74819285	9,928266677		
PCP028203	9,720534991	9,820705514	10,08304047	10,03387721		
PCP028208	8,944477815	9,42485912	9,165736112	9,426264755	PCP028208	XM_009341772
PCP028210	8,898843202	9,629957618	9,29156212	9,070577167		
PCP028216	9,925806611	9,993886607	10,00327897	10,52290515	PCP028216	XM_008394460
PCP028217	9,872936465	10,18570552	10,18899675	10,43844881		
PCP028249	9,318158642	9,320416982	9,50249371	13,3928983	PCP028249	XM_009356948
PCP028279	9,312134193	9,637222599	9,746514321	9,972505988		
PCP028283	9,322671793	9,437398376	9,972505988	9,971543554	PCP028283	XM_008390094
PCP028284	9,895832748	9,856939018	10,1412514	9,87958325	PCP028284	XR_531593
PCP028294	9,200481794	8,982993575	8,73470962	8,779161208	PCP028294	XM_009359548
PCP028323	9,534633604	9,484480553	9,497851837	9,939579214	PCP028323	XM_009346570
PCP028353	12,9708249	12,78497626	12,88073144	12,78756184	PCP028353	XM_009364053
PCP028356	9,654045226	9,822299952	9,790625442	9,653436267	PCP028356	XM_008339136
PCP028376	9,837107523	9,943979914	10,06743436	10,37937837	PCP028376	XM_008369209
PCP028381	9,461479447	8,959045612	8,850718177	8,089212109	PCP028381	XM_009343940
PCP028386	8,928755227	8,831845581	8,723387907	8,875503635	PCP028386	XM_009381338
PCP028408	9,246336825	9,382991408	9,385862401	9,387306529		
PCP028409	11,30092449	11,02467567	11,13335417	11,39356743	PCP028409	XM_009348361
PCP028416	10,54303182	10,55842071	11,11850638	10,94763694	PCP028416	XM_018644245
PCP028428	9,695802471	10,10852446	9,508448461	9,544326957	PCP028428	XM_009365279
PCP028442	8,937609223	8,888743249	8,863164132	8,836050355	PCP028442	XM_009347598
PCP028473	8,984902599	8,935666001	9,107661718	8,743723645	PCP028473	XM_008358817
PCP028477	10,80976813	10,85174904	10,80789422	11,162813	PCP028477	XM_009370687
PCP028497	10,94104761	10,96168844	11,23222304	11,65314513	PCP028497	XM_009376293
PCP028507	9,634212071	9,689421477	9,606090543	9,292321633		
PCP028515	11,50498281	11,92097432	11,82906069	12,04017668	PCP028515	XM_009341445
PCP028519	10,59276535	10,73470962	10,8844224	11,34411279	PCP028519	XM_008356623
PCP028540	10,11461429	10,12627857	10,033423	10,1382718		
PCP028544	9,28077077	9,08127038	9,22881869	9,086587411	PCP028544	XM_009343599
PCP028568	9,978710459	10,14720492	9,767075406	8,961941603	PCP028568	XM_009345221
PCP028576	12,33352716	12,31165467	12,42057346	12,76480319	PCP028576	XM_018651975
PCP028593	9,591840209	9,687673116	9,765982204	10,03112222	PCP028593	XM_018647857
PCP028626	9,797126495	10,1959873	9,914385132	10,02928723	PCP028626	XM_009345109
PCP028724	9,175749131	9,259743264	9,353874607	9,684170024	PCP028724	XM_009359454
PCP028832	9,921840937	9,922837094	9,952260754	10,28963853	PCP028832	XR_669371
PCP028841	8,918863237	8,742578916	9,063395081	9,459431619	PCP028841	XM_018650906
PCP028842	9,749316372	9,691743519	9,871905238	10,27806608	PCP028842	XM_009373796
PCP028846	9,398038202	9,485829309	9,5980525	9,848106482		
PCP028853	8,682994584	8,795487741	8,855896288	9,232013125		
PCP028856	7,971543554	9,335390355	8,989621566	10,47471995	PCP028856	XM_009373824
PCP028858	8,75598914	8,977279923	8,864186145	8,845490051	PCP028858	XR_001954347
PCP028873	8,716545126	9,037080165	9,113742166	9,300741498		
PCP028884	9,564778269	9,427669021	9,702744108	9,819109312		
PCP028885	10,84809866	10,79496002	10,79305864	11,06317259	PCP028885	XM_009371666
PCP028895	10,37503943	10,33539035	10,47302584	10,906139		

PCP028911	12,61800229	12,57214834	12,36723181	12,52601011	PCP028911	XM_009341623
PCP028922	9,574271841	9,514378734	9,694653884	9,980139578		
PCP028932	8,912889336	8,957102042	8,863164132	8,804131021	PCP028932	XM_008343929
PCP028936	10,9186173	10,95637616	11,02190302	11,32848981	PCP028936	XM_008355371
PCP028946	11,77739095	11,80426415	11,35608327	11,14465824	PCP028946	XM_008355376
PCP028959	10,35204343	10,27534467	10,34170783	10,6635581	PCP028959	XM_008343976
PCP028972	11,4920184	11,28231817	11,32549098	11,50150383	PCP028972	XM_009337079
PCP028973	13,79235006	13,57262089	13,60775451	13,76545912	PCP028973	XM_009337080
PCP028976	11,3777528	11,38657926	11,41662338	11,3376219	PCP028976	XM_021962424
PCP029008	10,95782756	11,16113188	11,08436329	11,19187968	PCP029008	XM_009335765
PCP029027	8,807354922	8,827596761	8,960001932	8,740354199	PCP029027	XM_009358516
PCP029037	9,836571147	9,750991645	9,986794882	10,15523567	PCP029037	XM_009358541
PCP029042	9,055282436	9,083027168	9,299208018	9,533329732	PCP029042	XM_018647550
PCP029046	9,004698008	8,941546519	8,733591606	8,84024291		
PCP029049	9,382991408	9,751544059	9,964817355	9,938594555	PCP029049	XM_018647551
PCP029086	8,716545126	8,754887502	8,869347076	9,173252384	PCP029086	XM_009364793
PCP029093	10,36158196	10,10328781	10,20863674	10,37937837		
PCP029098	9,643260955	9,842350343	9,911886285	10,71338651	PCP029098	XM_018651817
PCP029102	8,91489335	8,895817605	8,888743249	9,478425839	PCP029102	XM_018648892
PCP029105	11,74763496	11,49402052	11,62859591	11,76694294	PCP029105	XM_009354232
PCP029117	9,075024475	9,248710343	9,122388003	9,244768161	PCP029117	XM_009354208
PCP029118	9,539158811	9,378663341	9,422758607	10,07280253	PCP029118	XM_008354069
PCP029130	9,705062108	9,787902559	9,997645247	9,864186145		
PCP029162	10,49818105	10,5526691	10,49452562	10,15355203	PCP029162	XM_009354572
PCP029177	9,871905238	9,970105891	10,03846636	10,31288296		
PCP029187	9,215120395	9,255807556	9,376494429	9,756005825	PCP029187	XM_009346900
PCP029191	11,68313937	11,75836065	11,7828631	11,81070417	PCP029191	XM_008349764
PCP029197	9,987264012	9,950803736	10,16532158	10,55906156		
PCP029216	9,051671182	9,176597042	8,81483878	8,70276142		
PCP029217	10,19188584	10,1438041	10,34614803	10,89127487		
PCP029231	13,90174653	13,21366168	12,88385179	11,71195514	PCP029231	XM_008355376
PCP029240	8,77478706	8,865207434	8,945443836	9,35974956	PCP029240	XM_008381747
PCP029242	9,113742166	9,01402047	9,051671182	9,418548384	PCP029242	XM_009366918
PCP029254	9,094526396	8,895817605	8,967716202	8,846524657	PCP029254	XM_009366938
PCP029256	12,61279249	11,17409552	12,47581701	12,44086917	PCP029256	XM_009378962
PCP029264	11,32399485	11,17409552	11,20457114	11,2959583	PCP029264	XM_009342851
PCP029288	9,784094961	9,651051691	9,674774879	9,741466986		
PCP029317	9,730758817	9,605479518	9,77478706	9,534633604	PCP029317	XM_009352686
PCP029326	13,94873224	13,88270704	13,70606427	13,78817488	PCP029326	XM_009352611
PCP029327	8,731319031	8,716545126	8,851749041	9,223205318	PCP029327	XM_008394704
PCP029330	9,884170519	9,891282469	10,03387721	10,5173446	PCP029330	XM_009352573
PCP029336	10,4604559	10,61501344	10,55618989	10,14507876	PCP029336	XM_018647187
PCP029338	9,821773982	9,883147293	9,99905574	9,866243459	PCP029338	XM_009356744
PCP029345	10,30910114	10,34614803	10,5193119	10,44121164		
PCP029349	9,010304306	9,184057088	9,222384371	8,950322598		
PCP029362	10,72649918	10,55170826	10,44052662	10,54044774	PCP029362	XM_009381465

PCP029387	9,027905997	9,216745858	9,293080746	9,520952554		
PCP029395	11,14444794	10,34281916	10,7067809	10,86056713	PCP029395	XM_009365678
PCP029400	10,76514518	10,73527677	10,98347107	11,17783684		
PCP029403	9,736959959	9,842350343	9,981567282	10,2495046	PCP029403	XM_009365692
PCP029405	9,864186145	9,782457711	9,786808699	9,761551232	PCP029405	XM_008356139
PCP029420	8,593689902	8,904875427	9,28000515	9,70333261		
PCP029427	9,245552706	9,293080746	9,361943774	9,247927513	PCP029427	XM_008382565
PCP029430	10,67360059	10,67271703	10,78026908	11,06137119	PCP029430	XR_530532
PCP029443	9,258165557	9,159871337	9,118941073	9,079484784	PCP029443	XM_017335266
PCP029457	8,806291831	9,001886668	9,244768161	9,915879379	PCP029457	XM_009356357
PCP029475	9,028817757	9,047123912	9,142107057	9,506466274	PCP029475	XM_009347386
PCP029481	9,692911856	9,60856902	9,774243476	10,26951244		
PCP029486	8,531381461	8,687655621	8,887738491	9,290018847	PCP029486	XM_018649627
PCP029490	11,07503116	11,15755537	11,11113567	11,43289157	PCP029490	XM_008383313
PCP029503	9,33911511	9,116343961	9,253445696	9,025139562		
PCP029506	9,783538504	9,726218159	9,784634846	10,10983065		
PCP029515	10,16867212	10,30947635	10,25068924	10,26678654	PCP029515	XM_009345954
PCP029518	11,94422522	11,90701457	12,22198882	12,85142307	PCP029518	XM_009346662
PCP029519	12,9938813	12,89330153	13,31037563	14,02011184	PCP029519	XM_009346655
PCP029520	10,73555602	10,54850406	10,18074213	10,40372574	PCP029520	XM_018644892
PCP029529	9,648051932	9,77807713	10,06787799	10,52779952		
PCP029535	9,244768161	9,40017499	9,383704292	9,795503976	PCP029535	XM_008342349
PCP029545	8,923832563	8,959045612	8,957102042	8,911901261		
PCP029550	9,299963518	9,275356315	9,263668263	9,600526229	PCP029550	XM_009362942
PCP029552	10,04484489	9,937623934	10,08524671	10,03250037		
PCP029567	8,75043902	8,765965634	8,957102042	9,84443901	PCP029567	XM_008378623
PCP029575	11,42836017	11,46454575	11,38298601	11,44811631		
PCP029580	10,50548408	10,19311874	10,48984796	11,2179577	PCP029580	XR_001953260
PCP029581	9,251884747	9,240791332	8,982993575	8,851749041	PCP029581	XM_009358955
PCP029585	9,806823474	10,05708468	10,08347933	10,3623055	PCP029585	XM_008380340
PCP029589	9,645063941	9,578051847	9,775330438	10,07414146	PCP029589	XM_008359330
PCP029611	9,878556764	9,695802471	10,53559616	11,39178061	PCP029611	XM_009345150
PCP029614	10,24118474	10,29270124	10,42030765	10,27574041	PCP029614	XM_018642585
PCP029619	8,558420713	8,779161208	8,920858975	9,637838439	PCP029619	XM_009336251
PCP029620	10,08702519	9,983463942	10,15692998	10,60671056		
PCP029636	10,01122726	10,50017464	10,4252159	11,8031907	PCP029636	XM_009346238
PCP029660	9,873951581	10,07280253	10,20863674	10,48549727	PCP029660	XM_008345609
PCP029661	9,124121312	9,076815597	9,2644426	9,074141463	PCP029661	XM_008345610
PCP029662	10,07904471	9,936637939	9,947885279	9,722244002	PCP029662	XM_008383968
PCP029739	10,3011189	10,45771945	10,46488265	10,52421847	PCP029739	XM_009360761
PCP029756	8,872428639	9,047123912	9,206208529	9,396604781	PCP029756	XM_009356548
PCP029764	9,775890069	9,725076497	9,652844973	9,965784285	PCP029764	XM_008366608
PCP029769	9,01402047	9,2644426	9,156512913	9,286164981	PCP029769	XM_009339125
PCP029770	8,892785649	8,880593701	8,932716728	9,588714636		
PCP029786	12,62258495	12,67168922	12,75565331	13,12648992	PCP029786	XM_008386294
PCP029802	8,81483878	8,918863237	9,061587209	9,380093039	PCP029802	XM_008395768

PCP029819	9,069664578	9,422758607	9,84862294	10,82839834	PCP029819	XM_009368568
PCP029825	9,124974266	9,102421932	9,21916852	9,712527		
PCP029833	11,95274125	11,92283338	12,03399762	12,32998409	PCP029833	XM_009363531
PCP029834	11,26659331	10,15734694	11,31458324	12,28549759	PCP029834	XR_001953532
PCP029838	9,219967107	10,29653771	10,32530546	13,19352536	PCP029838	XM_009342691
PCP029843	9,674774879	9,807354922	9,901877548	10,13869418	PCP029843	XM_009358895
PCP029868	8,925791779	8,889747307	9,166589927	9,649256178	PCP029868	XM_008384884
PCP029874	10,23801057	10,25541812	10,42870562	10,64446019		
PCP029877	11,01471793	11,07881795	11,08790699	10,86108691		
PCP029879	10,04984855	10,53689798	10,5101015	9,758772867	PCP029879	XM_009377605
PCP029897	8,884689491	9,02699366	8,946409212	8,871381646		
PCP029909	9,067891433	9,010304306	9,118084543	9,47100451	PCP029909	XM_009362538
PCP029910	11,83578594	12,03353657	12,10131915	12,46130805	PCP029910	XM_009362536
PCP029912	8,934663924	8,926800034	8,985841937	9,405843489	PCP029912	XM_009365060
PCP029941	8,655816908	8,60855054	9,265216522	8,819125283	PCP029941	XM_008393980
PCP029948	10,65880951	10,63390341	10,54946703	10,77313921	PCP029948	XM_009381238
PCP029966	9,206208529	9,310612782	9,450530824	9,81805485	PCP029966	XM_009348233
PCP029997	10,8917837	11,18838485	11,03937833	10,65731845	PCP029997	XM_009371229
PCP030023	10,22641219	10,12066557	10,25777675	10,60979743	PCP030023	XM_008346182
PCP030037	9,833411948	9,884674229	9,990572019	10,33650656	PCP030037	XM_009340469
PCP030065	10,04712391	9,90287251	10,02375435	9,144658243		
PCP030076	10,99270471	10,78463485	10,83815604	10,24079133	PCP030076	XM_009379206
PCP030097	9,927289081	9,784634846	9,791162889	10,12970803	PCP030097	XM_009360074
PCP030098	13,16553514	12,50729912	12,7373889	12,84542536	PCP030098	XM_009369549
PCP030115	9,73753467	9,905883363	10,01541505	10,3479867	PCP030115	XM_009352085
PCP030165	13,3256807	13,321036	13,26405108	13,37132335	PCP030165	XM_009367147
PCP030207	11,04485172	10,3786525	9,618991087	9,509775004	PCP030207	XM_008355283
PCP030268	10,27767098	10,20294414	10,78653919	10,34983409		
PCP030312	10,93024207	11,22057785	11,2380046	11,99964774	PCP030312	XM_009341766
PCP030330	10,69145565	11,00515433	10,9168722	11,215533	PCP030330	XM_009355881
PCP030349	9,342074668	9,454648871	9,285402219	8,916864735	PCP030349	XM_009357450
PCP030351	12,03514275	11,95601308	11,9906961	11,83539121		
PCP030401	9	9,221587121	9,253445696	9,136145235	PCP030401	XM_018642518
PCP030409	9,831307244	9,689997971	9,782998209	9,799281622		
PCP030413	10,2510918	10,2219858	10,13741386	10,18404469	PCP030413	XM_018651569
PCP030432	9,500503319	9,419264989	9,780818601	10,21714588		
PCP030436	8,21757002	8,540438054	9,420655031	9,37286506	PCP030436	XM_008367278
PCP030454	9,623277955	9,591204414	9,939579214	10,51142653	PCP030454	XM_009362447
PCP030456	10,51142653	10,54528307	10,5666915	10,50812159		
PCP030482	9,026053072	9,017727086	9,110274492	9,002815016		
PCP030510	9,78026088	9,898858314	9,915879379	9,837107523	PCP030510	XM_009365249
PCP030515	8,76927586	8,872428639	8,947403166	9,47100451	PCP030515	NM_001293876
PCP030518	9,179088013	9,340584522	9,55011186	9,446400988	PCP030518	XM_018643861
PCP030537	10,49218368	10,36120908	10,203348	10,51471405	PCP030537	XM_018643956
PCP030542	10,35828856	10,46658634	10,60239267	10,75849807		
PCP030544	10,68299458	10,70936819	10,74483384	10,77561028	PCP030544	XM_009369278

PCP030555	12,2217895	12,47969691	12,67632516	14,04860011	PCP030555	XM_018645678
PCP030576	9,994353437	10,11026144	10,169925	10,39124359	PCP030576	XM_008352731
PCP030592	9,782457711	10,06653324	10,17033821	10,68796174	PCP030592	XM_009341858
PCP030595	10,36850646	10,32380913	10,44224893	10,7128107	PCP030595	XM_009341910
PCP030598	9,364856916	9,161560218	9,349458168	9,622655867		
PCP030599	8,856954575	9,096267125	9,158180476	9,649256178	PCP030599	XM_009341987
PCP030602	9,138706975	9,124121312	9,4325419	9,691743519	PCP030602	XM_008388014
PCP030605	9,701601036	9,685922633	10,04848687	10,32605865	PCP030605	XM_009342106
PCP030607	9,830245815	9,604238155	9,806275718	9,696393811		
PCP030614	9,363412032	9,327170869	9,352418676	9,930249459	PCP030614	XM_008392758
PCP030615	8,548166865	9,119797095	9,347997771	9,436024381		
PCP030634	7,462134139	8,524855097	8,95419631	12,21785776	PCP030634	XR_668418
PCP030688	11,57380338	11,42975717	11,44000225	11,53673289	PCP030688	XM_009341731
PCP030706	9,407968756	9,090985714	9,246336825	8,977279923	PCP030706	XM_009349312
PCP030727	9,437398376	9,459431619	9,255807556	9,039823818	PCP030727	XM_009353361
PCP030740	9,33911511	9,094526396	9,064284694	8,68183575	PCP030740	XM_008342317
PCP030751	9,630576566	9,58370335	9,533972085	9,546257836	PCP030751	XM_009353326
PCP030767	9,336885873	8,987747198	8,963849777	9,538518764	PCP030767	XM_017336014
PCP030771	9,701601036	9,689997971	9,652253436	9,47100451	PCP030771	XM_008390348
PCP030784	10,96818437	10,54303182	10,57679609	10,42940674		
PCP030785	9,696967526	9,890765865	9,158180476	9,813781191		
PCP030796	8,922822231	8,988684687	9,106772296	9,637222599		
PCP030800	9,327934318	9,429762402	9,657622143	9,698131642	PCP030800	XM_017325722
PCP030810	8,678318438	8,969616759	9,066977144	9,477090385	PCP030810	XM_008380884
PCP030829	9,6171552	9,717110885	9,768730196	9,396604781	PCP030829	XM_009355581
PCP030833	14,58386609	14,13003396	14,1126024	13,7111292		
PCP030844	9,040755473	9,010304306	8,97441459	8,909893084	PCP030844	XM_009339292
PCP030859	9,762664404	10,23002044	10,3064377	10,32418055	PCP030859	XM_009371940
PCP030874	9,618385502	9,770944642	9,571126898	9,201315296		
PCP030879	10,4419067	10,2644426	10,39802751	10,25935488	PCP030879	XM_008356700
PCP030891	10,82654849	10,95201316	11,0945198	11,25207404	PCP030891	XM_018647857
PCP030939	9,178241565	9,193106413	9,204571144	9,588714636		
PCP030965	10,71653655	10,36194377	10,1382718	10,58934217		
PCP030969	9,870873272	9,971069283	9,991054099	9,967226259	PCP030969	XM_008362946
PCP030994	10,33091688	10,27496047	10,26757091	10,26091953	PCP030994	XM_008381818
PCP030998	9,28077077	9,352418676	9,618991087	9,477758266	PCP030998	NM_001328845
PCP031008	9,334653186	9,301496195	9,217545787	9,18157469		
PCP031013	12,3858624	12,61562964	12,58355293	12,4301049	PCP031013	XM_018649546
PCP031026	10,73301532	10,22400167	10,63541885	10,81644771	PCP031026	XM_009368350
PCP031030	11,12971447	10,73921523	11,17762509	11,33799535	PCP031030	XM_009379255
PCP031031	10,5533125	10,44570808	10,61868833	10,41327885		
PCP031065	9,344295908	9,145499145	9,515049296	9,637838439	PCP031065	XM_009358688
PCP031090	9,232013125	9,141264175	9,162391329	9,202931899	PCP031090	XM_009364604
PCP031098	11,28847969	11,17659081	11,48163296	11,8163157	PCP031098	XM_009380877
PCP031115	9,983463942	9,92381771	9,987732989	10,31288296	PCP031115	XM_009360071
PCP031134	11,17679648	11,50382574	11,54012804	11,49685378	PCP031134	XM_009370350

PCP031139	9,742595757	9,691167722	9,835529375	9,651051691		
PCP031156	8,78571401	8,863164132	9,320416982	9,431142371	PCP031156	XM_009338084
PCP031157	11,78762917	11,76404222	11,69827058	12,02848282	PCP031157	XM_009338086
PCP031166	9,052568051	9,2589664	9,19063955	9,561612539	PCP031166	XM_009381012
PCP031172	10,08480839	10,08391805	10,29232163	10,69522829		
PCP031177	9,657014692	9,90287251	10,16574867	10,63178639		
PCP031185	10,61010206	10,39446269	10,34021731	10,69494112	PCP031185	XM_008353586
PCP031200	9,505811554	9,515049296	9,299963518	9,453270634	PCP031200	XM_016796525
PCP031216	9,124121312	9,274564521	9,475064611	9,991521846	PCP031216	XM_009366079
PCP031235	10,50315988	10,603005	10,64536122	10,92555444		
PCP031236	9,08833774	9,395169935	9,251884747	9,532024681		
PCP031260	9,681818185	9,20701432	9,424166289	9,452591317	PCP031260	XR_668417
PCP031263	9,662970945	9,725655961	9,931727372	10,19311874	PCP031263	XM_009367964
PCP031277	11,4238145	11,70173964	12,21937123	12,68547811	PCP031277	XM_008367804
PCP031281	10,34244141	10,20375175	10,07503785	9,614709844	PCP031281	XM_008368835
PCP031291	10,45943162	10,52649924	10,51076417	10,47370575		
PCP031300	8,965784285	8,915879379	9,140395236	9,664749481	PCP031300	XM_008375558
PCP031301	10,43844881	10,42101286	10,46828802	10,93957921		
PCP031304	10,11286951	9,922837094	10,07725635	10,29997496	PCP031304	XM_009340269
PCP031305	10,56255726	10,50581155	10,61868833	10,85823749	PCP031305	XM_009372672
PCP031307	9,108524457	9,075024475	9,257387843	9,57680554	PCP031307	XM_018651811
PCP031308	9,441554026	9,479780264	9,982523054	9,986311377	PCP031308	XM_018651810
PCP031358	9,840258649	9,805743872	10,03250037	10,4429435	PCP031358	XM_009372812
PCP031360	9,390878304	9,442943496	9,645063941	9,892800824	PCP031360	XM_009375329
PCP031396	9,02699366	9,209453366	9,468970167	9,580578921	PCP031396	XM_009356749
PCP031402	8,992456886	8,968666793	9,10938668	9,468277836	PCP031402	XM_018650263
PCP031409	11,64700864	11,69551976	11,71610351	11,98702591		
PCP031422	10,32418055	10,37032778	10,29039906	10,36558975	PCP031422	XM_009379893
PCP031426	10,78681687	10,84287672	10,85149139	10,91936243	PCP031426	XM_008382512
PCP031445	9,320416982	9,402308618	9,456703495	9,332417038	PCP031445	XM_008360225
PCP031447	8,911901261	8,819125283	8,978224236	9,475733431	PCP031447	XM_018646091
PCP031449	10,41961263	10,4419067	10,44121164	10,4307975	PCP031449	XR_001951836
PCP031468	10,01865921	10,45155114	10,43532647	10,37177664		
PCP031481	8,856954575	8,975360779	8,925791779	8,860062694	PCP031481	XM_018651795
PCP031523	11,21431912	11,13293022	11,17200864	11,21087221	PCP031523	XM_009379374
PCP031534	10,01726776	9,840258649	9,946423834	10,52388535		
PCP031552	9,151447887	9,202123824	9,395169935	9,791700135	PCP031552	XM_009380701
PCP031553	13,13517539	12,88760142	13,08707659	13,33636708		
PCP031577	9,207819661	9,393755108	9,293862446	9,350939182	PCP031577	XM_008387088
PCP031718	9,706789535	9,44362737	9,417852515	9,440183984	PCP031718	XM_009341767
PCP031886	10,3200446	10,46624984	10,3305472	10,71681946		
PCP031889	11,58965115	11,70144942	11,73569563	12,26883437		
PCP031895	9,661778098	9,340584522	9,537859025	9,82866014	PCP031895	XM_018646058
PCP031941	9,451211112	9,752664976	9,772595002	9,905883363		
PCP031963	9,116343961	9,157346935	9,30986284	9,743706817	PCP031963	XM_009377271
PCP031978	10,82813648	10,87728413	10,82892983	10,86289302		

PCP031996	9,537859025	9,621447519	9,497193195	9,552035018	PCP031996	XM_018652140
PCP032024	11,03204573	10,65702363	10,74034577	10,69522829		
PCP032041	9,794415866	9,855911856	9,849139214	9,848106482	PCP032041	XM_009338650
PCP032108	9,004698008	9,053464362	9,037080165	8,941546519		
PCP032112	9,618385502	9,541096615	9,668299989	8,671257984	PCP032112	XM_008350577
PCP032125	10,54399845	10,43115282	10,57206541	10,83447105	PCP032125	XM_009351502
PCP032137	9,556506055	9,400879436	9,607330314	9,982038114		
PCP032168	10,10372055	10,37793712	10,26951244	10,29001885	PCP032168	XM_009337959
PCP032285	11,07926476	11,18446007	11,18363538	11,25029842	PCP032285	XM_009357953
PCP032325	12,91849059	12,78613279	12,91469718	12,79705352	PCP032325	XM_009358786
PCP032426	9,721099189	9,707928505	9,896831887	10,20252792	PCP032426	XM_009362120
PCP032427	12,92851838	13,13083919	13,23017058	13,46718139	PCP032427	XM_009362119
PCP032549	13,37910757	13,14412765	13,18280867	13,53097565	PCP032549	XM_009366892
PCP032611	11,05030442	10,99104701	11,01866616	10,94860815	PCP032611	XM_008340344
PCP032736	10,12713025	10,66593099	10,60146439	10,76680218	PCP032736	XM_009337907
PCP032784	11,77561028	11,5943246	11,86534664	12,12066881	PCP032784	XM_009360370
PCP032824	9,739223673	9,876516947	10,15270314	10,26130749	PCP032824	XM_009341393
PCP032847	10,77971936	10,43532647	10,45429929	10,91761823	PCP032847	XM_009344811
PCP032877	9,822299952	9,667111542	9,785174528	9,721099189	PCP032877	XM_009357866
PCP032883	9,855911856	9,898858314	9,940563202	10,25068924		
PCP032928	8,820178962	8,692336525	9,024225474	9,232013125	PCP032928	XM_009368091
PCP033033	9,42485912	10,77505877	9,10938668	9,112882543		
PCP033076	11,99776872	11,89746734	12,05403785	12,31146452	PCP033076	XM_009372631
PCP033145	11,0425691	10,96457913	11,22319928	11,41309898	PCP033145	XR_664847
PCP033148	9,928770029	10,14168568	10,0692215	10,4325419	PCP033148	XM_009359409
PCP033155	11,71953	11,77780187	11,82760867	12,20344895	PCP033155	XM_009337634
PCP033179	9,010304306	8,87036472	8,791162889	8,74819285	PCP033179	XM_009347081
PCP033196	9,097163045	9,188168462	9,63540978	9,632395464	PCP033196	XM_018642793
PCP033208	9,482465137	9,599299194	9,702172685	9,943496494		
PCP033212	9,113742166	9,103287808	9,274564521	9,525520809	PCP033212	XM_009353501
PCP033216	10,57238759	10,66029011	11,14508513	11,80587685	PCP033216	XM_009373638
PCP033237	9,075024475	8,853839746	9,017727086	9,375756255	PCP033237	XM_008357384
PCP033443	9,944463173	9,945926605	10,11591499	10,05572982		
PCP033444	9,52028473	9,083027168	9,298452123	9,548821908	PCP033444	XM_009344863
PCP033451	10,24555271	10,53850906	10,77093639	11,30245044	PCP033451	XM_018643552
PCP033494	10,57806128	10,6972543	10,64475759	11,11525828		
PCP033537	9,097163045	9,229611955	8,993419626	8,933690655	PCP033537	XM_009345298
PCP033547	8,839203788	8,995286644	9,067891433	9,395169935		
PCP033606	9,591840209	9,563520437	9,636624621	9,433940073	PCP033606	XM_009350052
PCP033690	11,02120903	11,41749918	11,39838565	12,02017089	PCP033690	XM_009353884
PCP033703	9,548186135	10,01076585	9,775330438	10,48951685	PCP033703	XM_009359512
PCP033732	11,2072157	10,80170836	11,22641219	11,84392105	PCP033732	XM_018649862
PCP033774	8,686500527	9,247927513	9,038013592	9,169098221	PCP033774	XM_009364972
PCP033840	8,548166865	8,758223215	9,034331283	9,525520809	PCP033840	XM_018649864
PCP033872	11,07325121	11,09495518	11,09275714	11,44207783		
PCP033926	11,72891112	11,54206454	11,73188327	12,0166934	PCP033926	NM_001294121

PCP033927	9,820178962	9,636624621	9,824958741	10,03250037	PCP033927	XM_008394295
PCP033970	9,250298418	9,316666199	9,202123824	9,027905997	PCP033970	XR_001952506
PCP034107	10,64685568	10,63723166	11,24891784	11,69913863	PCP034107	XM_018652271
PCP034115	8,28077077	8,698114274	9,038013592	9,636026394	PCP034115	XM_008341770
PCP034117	10,20539007	10,21431912	10,21431912	10,21714588	PCP034117	XM_017323738
PCP034151	9,306813609	9,236803511	8,889747307	8,70276142	PCP034151	XM_008385565
PCP034171	8,886702554	9	9,201315296	9,497851837		
PCP034173	10,4153932	10,35388563	10,2510918	10,7299011	PCP034173	XR_667312
PCP034181	8,614709844	8,903881846	9,297672907	9,607940556	PCP034181	XM_009374293
PCP034239	9,113742166	8,9905862	9,189009107	8,988684687	PCP034239	XM_009375779
PCP034245	9,422064766	9,354624059	9,99859043	11,97835296	PCP034245	XM_021978226
PCP034370	10,78681687	10,82097669	10,87958325	11,38424412		
PCP034384	12,03891899	12,02074734	12,14402087	12,46913302		
PCP034386	10,09054913	10,25974326	10,31929955	9,977279923	PCP034386	XM_008340674
PCP034391	10,25699883	10,21916852	10,24911345	10,64655871		
PCP034393	8,645045922	8,628153873	8,696967526	9,448467384		
PCP034394	8,77148947	8,84024291	9,033423002	9,594940371		
PCP034415	8,706219712	8,862110391	8,95805965	9,333893285		
PCP034422	12,07202204	11,95080009	11,96024453	11,45069577	PCP034422	XM_009347509
PCP034439	8,889747307	9,164906927	9,051671182	9,438791853	PCP034439	XM_009365102
PCP034444	9,750991645	9,670656249	9,880088564	10,18280401	PCP034444	XM_009365241
PCP034458	9,148044344	9,106772296	9,069664578	8,984902599	PCP034458	XM_009355353
PCP034470	9,183213552	9,665922115	9,880609006	10,06474276	PCP034470	XM_009372829
PCP034502	9,194756854	9,208648936	9,411510988	9,747639151		
PCP034512	11,27068661	11,27165561	11,32624408	11,268542		
PCP034653	10,78026908	10,85174904	10,79873909	10,84679886	PCP034653	XM_009379936
PCP034659	9,754336368	9,463524373	9,84757418	9,785174528		
PCP034694	13,80980128	13,45103978	13,6998251	13,84490188	PCP034694	XM_009350086
PCP034702	9,189009107	9,113742166	9,131008067	9,46760555	PCP034702	XM_008374418
PCP034758	8,660602089	8,675957033	8,886702554	9,461479447	PCP034758	XM_017323276
PCP034945	10,82390059	10,82787459	10,8574601	11,36157648		
PCP034959	9,546257836	7,324900589	9,552035018	9,001886668		
PCP034992	10,21067134	10,18735207	10,47302584	10,35974956		
PCP035015	10,03983752	10,01865921	9,968191576	10,26600174	PCP035015	XM_009365834
PCP035024	12,84156435	12,5574637	12,65381466	12,76203524	PCP035024	XM_018645916
PCP035025	11,50564783	11,61148594	11,7300455	12,53576138		
PCP035033	9,318158642	9,214319121	9,213517401	9,553302899		
PCP035099	9,692911856	9,792790294	10,02236781	10,36814629	PCP035099	XM_009354240
PCP035118	9,799815854	9,961927137	10,12627857	10,64595559		
PCP035130	14,12169539	13,89494373	13,91089284	13,94626664	PCP035130	XM_018648701
PCP035151	10,37539789	10,40832974	10,44777547	10,8917837	PCP035151	XM_009350132
PCP035216	9,541096615	9,529430554	9,791700135	10,15440043	PCP035216	NM_001294071
PCP035259	9,709652505	9,426956579	9,529430554	9,906394584		
PCP035302	10,488513	10,48280796	10,5291084	11,04280163	PCP035302	XM_009340172
PCP035325	9,207819661	9,024225474	8,909893084	9,003742767	PCP035325	XM_009362877
PCP035387	9,507140523	9,398038202	9,614102459	9,856425529	PCP035387	XM_009346341

PCP035393	9,265216522	8,995286644	9,193944186	9,202123824		
PCP035412	9,312134193	9,169925001	8,977279923	8,904875427	PCP035412	XM_008396074
PCP035432	9,314401976	9,312134193	9,571126898	9,917372079		
PCP035462	10,94641652	11,91786806	11,66563349	12,19670355	PCP035462	XM_009381288
PCP035546	11,53931878	11,36121457	11,78667803	12,84575073		
PCP035557	9,048023611	9,113742166	9,378663341	9,517669388	PCP035557	XM_009339476
PCP035558	9,553955615	9,46760555	9,606090543	9,895832748		
PCP035631	9,177419538	9,466239641	9,568583198	10,26365653	PCP035631	XM_009368990
PCP035647	9,121533517	9,208648936	9,332417038	9,811647632	PCP035647	XM_009374629
PCP035851	10,0962803	9,511100329	9,735268302	9,694653884		
PCP035884	9,70333261	9,877023336	9,960972093	10,44845706	PCP035884	XM_009336923
PCP035895	12,72664392	13,22641219	12,77561028	13,44858998	PCP035895	NM_001302302
PCP035898	10,89405985	11,08347933	11,00609461	11,36121457	PCP035898	XM_009365589
PCP035907	9,445697739	9,363412032	9,462849868	9,429762402	PCP035907	XM_009375374
PCP035910	9,396604781	9,345782228	9,484480553	9,032514148	PCP035910	XM_008365958
PCP035921	9,219967107	9,184057088	9,356077767	9,594324604		
PCP035930	9,574915142	9,634212071	9,526166647	9,471675214		
PCP035932	10,29806257	10,69696753	10,70015358	11,18652903	PCP035932	XM_009341641
PCP036007	9,251079962	8,901862467	8,731319031	8,581200582	PCP036007	XM_018651156
PCP036047	9,531381461	9,7409107	9,7409107	10,19269967	PCP036047	XM_009353874
PCP036155	8,649866903	8,742578916	8,866258916	9,233619677	PCP036155	XM_009343105
PCP036161	9,804663461	9,566054038	9,830784548	10,0923211		
PCP036169	10,89254282	10,75461196	10,86933936	11,18281022	PCP036169	XM_009355517
PCP036303	8,722226922	8,833933692	8,982993575	8,82336724		
PCP036312	10,02559639	9,816455711	9,882138629	10,01076585	PCP036312	XM_009347985
PCP036361	10,56985561	10,18694371	10,80035798	11,19085557	PCP036361	XM_009337016
PCP036378	9,254249162	9,192292814	9,2644426	9,765982204	PCP036378	XM_008384313
PCP036479	10,15481811	10,06653324	9,970580478	10,20252792		
PCP036481	8,596189756	8,839203788	9,09011242	8,905898402	PCP036481	XR_524149
PCP036534	11,12131982	10,89127487	11,02721489	11,26931957	PCP036534	XM_009376878
PCP036557	9,325687724	9,395898244	9,350939182	9,377926277		
PCP036558	9,075024475	8,959045612	8,970594857	8,85277917	PCP036558	XM_009337547
PCP036562	9,445697739	9,642647426	9,714812179	9,886717794	PCP036562	XM_008343320
PCP036643	9,131856961	9,08127038	9,419960178	8,855896288		
PCP036765	10,1015424	10,05483491	10,25974326	10,47370575	PCP036765	XM_009362766
PCP036805	9,160703421	8,832890014	8,919846558	9,223205318	PCP036805	XM_009336367
PCP036814	9,019590728	8,900866808	8,942514505	8,791162889	PCP036814	XM_009336772
PCP036877	10,62632049	10,59121377	10,73075033	10,9580669	PCP036877	XM_009364445
PCP036886	11,20028561	11,49452062	11,41168575	11,76016688	PCP036886	XM_009338599
PCP037129	9,063395081	8,783554874	8,982993575	9,649256178	PCP037129	XM_009342166
PCP037138	10,49652427	10,49984589	10,60021957	10,92753354		
PCP037284	10,17700835	10,01308535	10,32830467	10,42241173	PCP037284	XM_009356785
PCP037299	9,718823954	9,371406396	9,760437201	9,382256552	PCP037299	XM_009381294
PCP037309	10,29576893	10,38118602	10,31288296	10,62965715		
PCP037432	10,33985	10,39874369	10,4767462	10,78681687	PCP037432	XM_008392157
PCP037479	9,00750388	9,08833774	9,642051693	10,3376219	PCP037479	XM_009354230

PCP037501	10,06114164	10,07547915	10,10852446	10,42556211	PCP037501	XR_514408
PCP037505	9,245552706	9,329415156	9,327934318	9,966260295	PCP037505	XM_009378963
PCP037522	12,08702188	12,20436634	12,31675387	12,28867289	PCP037522	XM_008386853
PCP037523	9,367786025	9,140395236	9,240004193	8,909893084	PCP037523	XM_009365667
PCP037560	10,85044472	10,61440618	10,71681946	10,78708632	PCP037560	XM_009358949
PCP037720	12,55042695	11,98216293	10,98513037	12,60779264		
PCP037779	10,31741261	9,225617167	9,794951903	9,045295522		
PCP037788	9,342808055	9,248710343	9,477758266	9,813252106	PCP037788	XM_009371936
PCP037789	9,791162889	9,968666793	10,0274499	9,957102042	PCP037789	XR_526917
PCP038025	9,063395081	8,97441459	9,115485887	9,403715086	PCP038025	XM_009339705
PCP038032	10,30910114	10,48213232	10,59960605	10,81057163		
PCP038045	10,11591499	10,33798978	10,33016621	10,36340108	PCP038045	XM_008368918
PCP038051	9,539798574	9,475064611	9,658211483	9,918863237	PCP038051	XM_008363738
PCP038118	9,021451966	8,951284715	9,187352073	9,479780264	PCP038118	XM_009359194
PCP038129	8,862110391	8,978224236	8,980139578	9,358277568	PCP038129	XM_009375115
PCP038156	9,97632067	10,0945132	10,19269967	10,4580682		
PCP038182	10,89860139	10,50878516	11,0114579	10,79658315		
PCP038298	9,708514896	9,616548844	9,71539578	10,02421162	PCP038298	XM_009345354
PCP038363	10,46283965	10,5865302	10,65761321	10,9346713	PCP038363	XM_009356423
PCP038409	9,619614757	9,580578921	9,702744108	10,1581931	PCP038409	XM_017330986
PCP038418	9,300741498	8,783554874	8,827596761	8,87036472		
PCP038500	9,588714636	9,525520809	9,680658406	9,960001932		
PCP038520	9,710238196	9,619614757	9,820178962	10,04939934	PCP038520	XM_009362766
PCP038628	9,144658243	9,099794885	9,210257347	9,791700135		
PCP038640	9,745959978	9,834992412	9,933690655	10,41433641		
PCP038676	9,874981348	9,944960906	9,960001932	9,880609006		
PCP038699	9,251079962	9,446400988	9,436711542	9,817511342	PCP038699	XM_009356466
PCP038744	9,193106413	9,208648936	9,320416982	9,177419538	PCP038744	XM_009351304
PCP038764	9,057991723	9,050746552	9,014941046	9,020507757		
PCP038800	9,830784548	10,00140819	9,87036472	10,10721708	PCP038800	XM_009372514
PCP038873	8,903881846	8,943481842	9,08833774	9,409390936	PCP038873	XM_009341266
PCP038959	8,805195705	8,787902559	9,008428622	9,315149562		
PCP038990	11,94580958	11,72778009	11,87344411	12,23461688	PCP038990	XM_008366971
PCP039020	10,52454172	10,6329952	10,5685927	10,68562484	PCP039020	XM_009370457
PCP039200	9,615942233	9,536577492	9,548821908	9,19640541		
PCP039280	8,72904287	8,532667615	9,05709822	9,414325838		
PCP039287	8,659389441	8,62935662	8,68183575	9,419264989	PCP039287	XM_009337460
PCP039303	10,12713025	9,897845456	10,10721708	10,41468524		
PCP039314	9,144658243	8,890781061	9,092757141	10,06518722	PCP039314	XM_009369685
PCP039510	11,08790699	11,30111318	11,3819052	11,30833903		
PCP039533	9,232013125	8,744833837	8,897845456	9,142107057	PCP039533	XM_009335833
PCP039548	10,34059565	10,29462075	10,44224893	10,73216743		
PCP039555	8,953236133	9,011227255	9,160703421	9,017727086	PCP039555	XM_009361001
PCP039570	9,214319121	9,388017285	9,320416982	9,40017499		
PCP039586	10,16532158	10,15355203	10,17617315	10,1412514		
PCP039592	9,042562264	9,159871337	9,325687724	9,744833837	PCP039592	XM_009357650

PCP039596	9,302250498	9,125852541	9,419960178	10,07236042	PCP039596	XM_009367213
PCP039600	9,074141463	8,895817605	9,07325791	9,024225474	PCP039600	XR_667600
PCP039616	11,82588498	11,78135971	11,74090648	11,89228856	PCP039616	XM_008355364
PCP039637	10,72252579	10,31552321	10,26209485	9,892800824	PCP039637	XM_009353371
PCP039638	8,977279923	8,939579214	8,75043902	9,080364533	PCP039638	XM_009353370
PCP039642	9,460803985	9,539798574	9,534633604	9,874474241		
PCP039652	9,500503319	9,592457037	9,559051993	9,939094317		
PCP039679	9,933203774	9,911391988	10,20985541	10,42030765	PCP039679	XM_009359475
PCP039709	12,18466772	12,06260948	12,22480663	12,1378461	PCP039709	XM_009358786
PCP039746	9,668884984	10,0588982	9,876516947	10,34170783	PCP039746	XM_009359512
PCP039792	9,28771238	9,133578937	9,250298418	9,141264175	PCP039792	XM_009347182
PCP039800	10,15101654	10,06653324	10,3505746	10,44467328		
PCP039809	8,335390355	8,309840108	8,470333494	10,91163916	PCP039809	XM_009341809
PCP039810	9,259743264	8,882643049	9,048949988	8,865207434	PCP039810	XM_009341652
PCP039836	8,82336724	8,794415866	9,193944186	9,782998209	PCP039836	XM_008345417
PCP039837	8,416459769	8,60855054	9,103287808	9,738649634	PCP039837	XM_008345418
PCP039847	9,573647187	9,385150582	9,385150582	9,468277836	PCP039847	XM_018645142
PCP039938	10,32530546	10,68533575	10,74230944	11,16616308	PCP039938	XM_008356806
PCP039961	9,231221181	8,471675214	8,904875427	8,84862294		
PCP039979	8,615923847	8,740354199	8,868297843	9,152284842		
PCP040026	9,394462695	8,794415866	8,70276142	10,58527242		
PCP040138	8,716545126	9,093628836	9,299963518	9,642647426	PCP040138	XM_009370695
PCP040177	10,13057056	10,35901375	10,73131903	11,10874006	PCP040177	XM_009349061
PCP040215	9,393755108	9,316666199	9,545601627	9,218369492	PCP040215	XR_525567
PCP040242	11,2628759	11,1165584	11,15777008	11,1600794	PCP040242	XM_009371569
PCP040249	9,87036472	10,01262454	10,12885787	10,44604941	PCP040249	XM_009351639
PCP040265	9,971543554	9,769837844	10,00327897	10,23481743	PCP040265	XM_008345170
PCP040329	10,00982862	9,485829309	9,22881869	9,402308618	PCP040329	XM_008379079
PCP040396	10,18363538	10,31098761	10,2737956	10,61960559		
PCP040460	8,080391184	8,666508075	9,939094317	11,09077405	PCP040460	XM_009376263
PCP040561	8,664162806	8,725638921	8,825499451	9,147204925	PCP040561	XM_009377717
PCP040704	9,760437201	9,793326936	10,35424938	11,15587443	PCP040704	XM_008357523
PCP040809	9,406566431	9,599299194	9,67124029	9,972979786		
PCP040818	11,17430154	10,77999014	10,84758201	11,21411873	PCP040818	XM_009344811
PCP040830	9,945443836	10,08878824	10,25068924	10,15987134	PCP040830	XM_009377725
PCP040863	10,3790209	10,46964182	10,49485558	10,82362996	PCP040863	XM_009366091
PCP040965	8,84024291	8,839203788	8,784634846	9,340584522		
PCP041029	10,14974712	9,792790294	9,82707272	9,916879659	PCP041029	XM_009380213
PCP041111	9,747639151	10,09496177	10,11026144	10,11981006	PCP041111	XM_018650406
PCP041181	9,139551352	9,206208529	9,151447887	9,20701432	PCP041181	XM_009372321
PCP041349	10,46352437	10,47404051	10,47337091	10,77588184	PCP041349	XM_009373989
PCP041357	10,0588982	9,560963278	9,479113318	9,186535222		
PCP041385	9,345782228	9,637222599	9,533329732	9,650459419	PCP041385	XM_009370019
PCP041401	9,643260955	9,897346324	9,863148641	9,799281622	PCP041401	XM_018649882
PCP041443	16,05172577	15,86843676	15,89937274	16,0931493		
PCP041613	9,25502857	9,115485887	9,183213552	9,526831754	PCP041613	XM_008354832

PCP041614	9,469641817	9,098032083	8,979196518	8,969616759		
PCP041629	9,417852515	8,84024291	9,053464362	8,888743249	PCP041629	XM_008228004
PCP041645	10,05347794	10,28771238	10,87395927	11,67286728	PCP041645	XM_009335626
PCP041866	10,33427329	10,37503943	10,40016431	10,40797937	PCP041866	XM_017326527
PCP041883	8,924812504	8,976334992	9,048949988	9,37286506		
PCP041888	9,09011242	9,454648871	9,338357556	9,399448837		
PCP041932	9,564778269	9,683573652	9,681818185	10,12196082		
PCP041966	11,52552081	11,4446681	11,63541432	11,58762283	PCP041966	XM_008340338
PCP041977	12,01216009	12,16741815	12,57380574	13,07903971	PCP041977	XM_009352908
PCP042003	12,56502351	12,2850178	12,42539164	12,83368075	PCP042003	XM_018643930
PCP042026	9,550746785	9,535275377	9,54173552	9,197216693	PCP042026	XM_018648246
PCP042027	8,874458871	8,816983623	9,286950435	9,36047402	PCP042027	XM_009358423
PCP042229	10,29039906	10,07725635	10,42975194	10,43010752	PCP042229	XM_009379749
PCP042242	9,144658243	9,286164981	9,184057088	9,123267853	PCP042242	XM_009367765
PCP042243	8,985841937	8,910882535	8,913876736	8,855896288	PCP042243	XM_009367751
PCP042291	8,731319031	8,882643049	8,825499451	9,17990909	PCP042291	XM_008359765
PCP042296	8,944477815	9,233619677	9,098032083	9,442943496		
PCP042298	9,982523054	9,985841937	10,07012094	10,62965715	PCP042298	XM_009363893
PCP042327	8,889747307	8,856954575	9,136145235	9,385150582	PCP042327	XM_009380795
PCP042450	9,86004717	9,856425529	9,784094961	9,749316372	PCP042450	XM_008393669
PCP042476	9,119797095	8,788979283	9,006550496	8,850718177	PCP042476	XM_008388412
PCP042484	9,435316054	9,618385502	9,622051819	9,593708574	PCP042484	XM_009348829
PCP042525	9,447765141	9,654636029	9,659995892	10,42136007		
PCP042530	9,538518764	9,904890476	10,34983409	10,97775931	PCP042530	XM_009379544
PCP042548	9,511100329	9,842350343	9,647458426	9,491853096	PCP042548	XM_009365768
PCP042552	8,707359132	8,818038868	8,87036472	9,444331629		
PCP042562	9,495195473	9,129283017	9,2589664	9,449148645		
PCP042567	9,127555902	9,356826076	9,42485912	9,610415859	PCP042567	XM_008395285
PCP042568	9,154818109	9,412209922	9,452591317	9,987732989	PCP042568	XM_008395282
PCP042572	9,764324258	9,698704667	9,805211831	10,34392964		
PCP042645	10,31778568	10,68503783	9,837107523	10,37177664	PCP042645	XM_009367378
PCP042800	10,13656824	9,854354156	10,0060876	9,910897522	PCP042800	XM_009358099
PCP042834	9,466932951	9,368506462	9,509121785	9,394462695	PCP042834	XM_018645119
PCP042854	10,13741386	10,14252831	10,19926745	10,51799411	PCP042854	XM_009362769
PCP042889	10,50812159	10,51142653	10,56605404	11,20803312		
PCP042931	9,347997771	9,306813609	9,485165154	9,304533858	PCP042931	XM_008366146
PCP042987	9,19802752	8,483815777	9,014941046	9,396604781	PCP042987	NM_001302391
PCP043059	12,99429509	12,46735081	12,43679741	12,21644274		
PCP043070	9,719953466	9,999534994	9,839203788	9,591204414	PCP043070	XM_018651665
PCP043133	8,977279923	8,788979283	8,950322598	9,441554026	PCP043133	XM_009374285
PCP043134	10,47337091	10,44432127	10,58371275	10,91313625		
PCP043135	9,686500527	9,763212367	9,652844973	10,22077734	PCP043135	XM_009374263
PCP043175	11,6731633	11,75697323	11,78012962	11,79306271	PCP043175	XM_009356145
PCP043201	9,246336825	9,097163045	9,159871337	9,498510178	PCP043201	XM_009377056
PCP043202	10,66977089	10,66059318	10,61900026	10,95564991	PCP043202	XM_009377057
PCP043223	14,80408059	14,66742593	14,79475447	15,08881598	PCP043223	XM_008368217

PCP043260	9,594324604	9,73809226	9,898858314	9,586220553	PCP043260	XM_009357823
PCP043280	8,383704292	8,470333494	8,922822231	9,761002638	PCP043280	XM_009361776
PCP043373	9,375756255	8,821231873	9,095397023	9,193106413	PCP043373	XM_009372878
PCP043390	16,72577457	16,54815467	16,94022951	17,52497267		
PCP043425	9,618991087	9,665335917	9,751544059	10,0692215		
PCP043445	9,663558104	9,745959978	10,0060876	10,22480965	PCP043445	XM_008384010
PCP043541	9,757673353	9,791700135	9,892800824	10,16406469	PCP043541	XM_008382397
PCP043652	10,64806092	10,25227514	10,07056375	9,553955615	PCP043652	XM_009346285
PCP043654	8,501160452	9,266013461	9,061587209	8,892785649	PCP043654	XM_009346271
PCP043658	9,020507757	9,031577154	9,245552706	9,108524457	PCP043658	XM_009337484
PCP043660	9,750991645	9,510427928	9,769292392	10,00327897	PCP043660	XM_009337506
PCP043691	13,25684703	13,4767462	13,52918651	13,77882971	PCP043691	XM_008391582
PCP043697	10,11070515	10,17990909	10,30492167	10,12153352	PCP043697	NM_001294083
PCP043711	8,652235507	8,784634846	9,035266491	9,217545787		
PCP043712	9,409390936	9,312882955	9,285402219	9,120678526	PCP043712	XM_017328371
PCP043715	10,87138935	10,87677016	11,15312765	11,23940162	PCP043715	NM_001293866
PCP043717	10,1176431	10,34170783	10,34466208	10,86625119		
PCP043745	9,130132926	9,21916852	9,336127147	9,743706817		
PCP043746	9,473035989	9,524874682	9,552669098	10,03159094		
PCP043766	9,110274492	9,413627929	9,62935662	9,86004717	PCP043766	XM_009367569
PCP043796	9,965308117	9,968191576	10,02421162	10,4307975		
PCP043858	9,676538932	9,670071972	9,894317583	9,435316054	PCP043858	XR_001953869
PCP043863	10,34687935	10,58777752	10,4118605	10,66059318	PCP043863	XM_009341298
PCP043938	9,819109312	9,613476402	9,838148156	10,06429817	PCP043938	XM_009350694
PCP043940	10,23202512	10,07236042	10,04302728	10,18818083		
PCP043977	9,306061689	9,213517401	9,226412193	9,275356315	PCP043977	XM_009371509
PCP044040	8,967716202	9,191478757	9,359024737	9,475733431	PCP044040	XM_009337659
PCP044053	9,136145235	9,422064766	9,402308618	9,769292392	PCP044053	XM_009344592
PCP044059	10,57080444	10,77834823	10,37829486	10,18115226	PCP044059	XM_009369127
PCP044128	9,086587411	8,955155849	8,980139578	8,930737338	PCP044128	XM_009365240
PCP044134	9,682994584	9,507140523	9,861086906	9,933690655	PCP044134	XM_009336078
PCP044144	9,314401976	9,316666199	9,555873659	9,359024737	PCP044144	XM_008377866
PCP044150	9,718241739	9,784094961	9,935651269	10,14168568	PCP044150	XM_009376686
PCP044153	9,004698008	8,807354922	9,216745858	9,230428801	PCP044153	XM_009339456
PCP044197	8,390899794	8,572397068	8,806291831	9,603014275		
PCP044249	9,21916852	9,231221181	9,347244868	9,664749481	PCP044249	XM_009368143
PCP044270	10,7128107	10,86547811	10,98655315	11,1501725	PCP044270	XM_009343258
PCP044271	9,211888295	9,306061689	9,553955615	9,779719355	PCP044271	XM_009343249
PCP044272	8,271463028	8,795487741	9,066977144	9,344295908		
PCP044318	9,112882543	9,238404739	9,223205318	9,531381461	PCP044318	XM_017331072
PCP044390	9,046223651	8,829722735	9,009352771	9,283852339	PCP044390	XM_009357891
PCP044393	8,812722827	9,074141463	9,06520069	9,339850003	PCP044393	XM_008350437
PCP044429	8,597419437	8,6794801	9,036173613	9,124974266	PCP044429	XM_008375136
PCP044438	9,319672121	9,183213552	9,121533517	9,038013592	PCP044438	XM_009363252
PCP044484	11,3459596	11,31722605	11,2736033	11,05279218	PCP044484	XM_009356972
PCP044511	9,488502959	9,856939018	9,668884984	9,773139207	PCP044511	NM_001328944

PCP044548	9,949826711	9,516350087	9,723370841	9,642051693		
PCP044563	10,37431138	10,24198315	10,38478375	10,37973575	PCP044563	XM_018647373
PCP044576	10,01168851	10,20089861	10,14720492	10,0370939	PCP044576	XM_009340112
PCP044586	9,642647426	9,650459419	9,731879027	10,06608919	PCP044586	XM_009370520
PCP044601	9,269126679	9,584342459	9,810571635	9,664145025	PCP044601	XM_017333957
PCP044613	8,855896288	8,838163917	8,78571401	9,19802752		
PCP044659	10,70822173	10,37177664	10,57049139	10,79873909	PCP044659	XM_009369176
PCP044677	9,531381461	9,390878304	9,189824559	9,164906927	PCP044677	XM_009355609
PCP044688	10,03754695	9,872936465	10,03021191	10,30226192		
PCP044698	10,08125706	10,16113188	10,3858624	10,63117706	PCP044698	XM_021948712
PCP044811	10,80467153	10,92950254	11,3555328	12,51035127		
PCP044830	8,884689491	8,828676005	9,271463028	8,821231873	PCP044830	XM_018642976
PCP044833	8,960957618	8,994353437	9,142949447	9,473035989	PCP044833	XM_018650045
PCP044846	8,775873612	8,688844752	8,73809226	9,141264175	PCP044846	XM_009358311
PCP044863	8,736976865	8,83709175	9,099794885	9,136991112	PCP044863	XM_018650254
PCP044872	9,303780748	9,102421932	9,369968059	8,804131021	PCP044872	XM_009369078
PCP045020	8,70158371	8,705044823	8,776992236	9,479113318	PCP045020	XM_018651990
PCP045021	8,645045922	8,626913629	9,010304306	9,309089764		
PCP045028	13,6462583	13,36422633	13,64835758	14,21011349	PCP045028	XR_665791
PCP045034	11,1825992	11,28270042	11,35792033	11,88937847	PCP045034	XM_009351735
PCP045057	10,00046486	10,4065558	10,28424575	10,5552314		
PCP045101	9,769837844	9,87958325	9,845490051	10,18776033	PCP045101	XM_009344367
PCP045115	9,300741498	9,277682605	9,251079962	9,083027168		
PCP045133	11,28828934	11,46267097	11,25286054	13,00883651		

Cluster_6

Gene	H1	H2	H3	H4		
PCP000030	7,979167931	7,900866808	7,950293432	7,803033252	PCP000030	XM_017326326
PCP000046	7,44567705	7,528805707	7,857980995	8,375039431	PCP000046	XM_008393849
PCP000064	7,805227956	8,027905997	8,013071384	8,338379842		
PCP000071	7,244791942	7,266786541	7,235248379	7,90285744	PCP000071	XM_018647602
PCP000101	6,594996337	6,759289016	6,890811455	7,3458044	PCP000101	XM_018651212
PCP000103	7,339850003	7,231989133	7,785746699	8,178266468	PCP000103	XM_008375314
PCP000110	7,315874125	7,520972191	7,572359168	7,946438456		
PCP000127	7,453929061	7,486473046	7,587440004	8,094500005	PCP000127	XM_017328477
PCP000150	7,163196797	7,473056289	8,087462841	8,305286574	PCP000150	XM_009377998
PCP000156	8,071462363	8,257387843	8,122413888	7,169925001	PCP000156	XM_009377994
PCP000178	7,74819285	8,501160452	8,214319121	8,495855027	PCP000178	XM_009344591
PCP000190	8,016808288	7,920829209	8,060695932	8,489165579	PCP000190	XM_009342109
PCP000195	7,792269724	7,665335917	7,677155841	8,188193194	PCP000195	XM_009342065
PCP000196	7,826548487	7,908872939	8,048050866	8,803065551	PCP000196	XM_008347526
PCP000204	7,876516947	8,09976859	8,302273349	8,386595423	PCP000204	XM_009339725
PCP000214	8,268331458	8,178266468	8,209453366	8,607330314	PCP000214	XM_009339702
PCP000231	8,118941073	8,233619677	8,315919398	8,714245518		
PCP000244	8,194756854	8,283875484	8,740354199	8,835008208	PCP000244	XM_020565026
PCP000254	7,080337882	7,241554209	7,327956767	7,83080039		
PCP000257	7,105070402	6,946379968	7,732472726	7,776959347	PCP000257	XM_017334679
PCP000264	8,201290788	8,129283017	8,369248353	8,978224236	PCP000264	XR_525486
PCP000269	8,449850215	8,209453366	8,448446735	8,199672345	PCP000269	XM_008380929
PCP000279	7,254272787	7,614709844	7,584962501	8,026080745		
PCP000284	6,902917719	6,772545519	7,06608919	7,541716163	PCP000284	XM_021957035
PCP000287	8,739206792	8,336863563	8,027905997	7,481153605	PCP000287	XM_008349789
PCP000289	8,354602022	8,389437748	8,615923847	9,071462363	PCP000289	XM_008359501
PCP000292	8,844454703	8,606109055	8,553322101	8,78571401		
PCP000294	7,348728154	7,486473046	7,380850638	7,327956767	PCP000294	XM_008359643
PCP000316	6,914923239	6,882643049	7,186559982	7,549438149		
PCP000321	7,497213158	6,996275749	7,285402219	7,336908182	PCP000321	XM_009357369
PCP000326	7,285402219	7,481153605	7,426264755	7,857980995	PCP000326	XM_009357376
PCP000329	7,351645995	7,033423002	7,297649983	7,06608919	PCP000329	XM_009357379
PCP000332	7	7,037052702	7,122362117	6,714245518		
PCP000350	7,920829209	8,115459877	7,904905525	7,101503009	PCP000350	XM_009371806
PCP000358	7,400879436	7,569855608	7,646234675	7,969645536		
PCP000368	9,025139562	8,398016818	8,630558365	8,929761415	PCP000368	XM_009371778
PCP000384	8,668884984	8,564797319	8,668884984	9,108524457	PCP000384	XM_008341852
PCP000388	7,646234675	8,705044823	7,992485211	8,087462841		
PCP000389	8,306836389	8,089212109	8,212715235	8,668884984		
PCP000407	8,909893084	8,708497652	8,345760055	8,442943496	PCP000407	XM_008341816
PCP000415	7,992485211	7,619633096	7,928784831	8,227230852	PCP000415	XM_008357306
PCP000418	7,866228001	7,979167931	7,886733033	7,841281284		
PCP000439	6,222456826	7,101503009	8,233619677	12,15966009	PCP000439	XM_009352525

PCP000467	7,986809101	7,882643049	7,998139076	8,5824431	PCP000467	XM_009351678
PCP000482	7,805227956	7,426264755	7,624466495	7,569855608	PCP000482	XM_009353776
PCP000489	8,810571635	8,744833837	8,478446064	7,389480771		
PCP000495	7,798763389	7,884658968	7,839203788	7,702726796	PCP000495	XM_008394823
PCP000496	8,723387907	8,459431619	8,557157324	9,101529272	PCP000496	XM_008394826
PCP000498	8,69115027	8,279239123	8,519636253	9,460107723	PCP000498	XM_017337494
PCP000515	6,857980995	7,094552786	7,333870928	7,783522135		
PCP000519	6,87036472	7,263691734	7,462134139	7,965784285	PCP000519	XM_018647984
PCP000529	7,77036657	7,969645536	7,948367232	8,519636253	PCP000529	XM_009360568
PCP000538	6,714245518	7,058857621	7,395148508	7,562242424	PCP000538	XM_018648522
PCP000539	8,127581695	7,874489611	8,136119594	8,448446735	PCP000539	XM_009340603
PCP000540	8,153146652	8,214319121	8,265239967	8,618385502	PCP000540	XM_009340602
PCP000543	7,118941073	7,279192684	7,251056285	7,864186145	PCP000543	XM_017323092
PCP000548	7,193081765	7,539158811	7,890750668	8,694636474	PCP000548	XM_008345752
PCP000551	7,894817763	7,73470962	8,159871337	8,487840034	PCP000551	XM_009373184
PCP000555	7,579919292	7,781359714	7,892816	8,519636253	PCP000555	XM_009357147
PCP000564	7,597456684	7,639015048	7,536596918	6,763809907	PCP000564	XM_009373147
PCP000584	7,115511897	7,448488033	7,743689989	8,350187812	PCP000584	XR_001792081
PCP000591	7,805227956	7,366322214	7,303780748	6,754887502	PCP000591	XM_009371386
PCP000597	8,389437748	8,515699838	8,276124405	7,602364826	PCP000597	XM_009371376
PCP000598	7,549438149	6,910852562	7,244791942	6,677085351	PCP000598	XM_018652222
PCP000604	6,794415866	6,454011343	6,80309785	7,395148508	PCP000604	XM_008356158
PCP000632	7,619633096	7,636624621	8,44431092	9,06608919	PCP000632	XM_009374219
PCP000633	7,437377568	7,705079392	7,752681699	8,33391564	PCP000633	XM_008379677
PCP000637	7,202907418	6,926829678	6,878602742	6,934634441		
PCP000640	8,013071384	7,807354922	8,252665432	8,309840108		
PCP000647	8,220765252	8,089212109	7,996219247	8,113742166	PCP000647	XM_009375710
PCP000651	7,118941073	6,785681319	7,139551352	8,044394119	PCP000651	XM_009375714
PCP000660	6,81159947	6,68187088	6,95419631	7,42063398	PCP000660	XM_009345488
PCP000665	8,881634033	8,859006685	8,818038868	8,445718428	PCP000665	XM_018644616
PCP000677	7,22881869	6,950351762	7,285402219	7,634230226	PCP000677	XM_009359650
PCP000682	7,636624621	7,868328714	7,843418611	7,626950122		
PCP000698	7,136170875	6,958030641	7,235248379	7,908872939	PCP000698	XM_009381235
PCP000700	7,22881869	7,617136829	7,841281284	7,6794801	PCP000700	XM_009381297
PCP000704	8,517000043	8,388017285	8,498530123	8,385129006	PCP000704	XM_009381379
PCP000741	8,198052084	8,426264755	8,82336724	9,543669869	PCP000741	XM_009340781
PCP000751	8,274587815	8,533991546	8,398016818	8,243173983		
PCP000772	8,153146652	8,405141463	8,320439548	8,739206792	PCP000772	XM_008369953
PCP000782	7,040782866	6,99242856	7,312882955	7,700439718	PCP000782	XM_008380631
PCP000785	7,536596918	6,984931073	7,366322214	6,727920455	PCP000785	XM_009373565
PCP000789	7,142923928	7,619633096	7,592457037	7,714245518	PCP000789	XM_009373644
PCP000797	8,481113232	8,630558365	8,523561956	8,568602197	PCP000797	XM_009373754
PCP000803	8,163247124	8,016808288	8,103287808	7,960001932	PCP000803	XM_009340573
PCP000807	7,17990909	7,333870928	6,969587981	7,785746699	PCP000807	XM_009340567
PCP000809	8,249492747	8,416459769	8,758223215	9,267559206	PCP000809	XM_018643480
PCP000811	8,257387843	8,274587815	8,254225537	8,640244936		

PCP000845	7,963878669	8,517000043	8,087462841	8,276124405	PCP000845	XM_009359336
PCP000854	8,037107627	8,314379315	8,385129006	8,124121312		
PCP000863	7,459431619	7,572359168	7,324900589	7,294620749	PCP000863	XM_018650240
PCP000872	7,940577883	8,473015688	8,3994702	8,28692734		
PCP000895	7,994353437	8,091012169	8,22881869	8,745943176	PCP000895	XM_009377235
PCP000918	8,183238369	8,158205728	8,335390355	8,641437649	PCP000918	XM_008360140
PCP000925	7,139551352	7,105070402	7,115511897	7,896816753	PCP000925	XM_009371897
PCP000939	8,440163216	8,473015688	8,568602197	9,117201525		
PCP000970	7,752681699	8,434628228	7,730164413	7,602364826		
PCP000971	6,965784285	6,910852562	7,282347131	7,626950122	PCP000971	XM_017332751
PCP000988	7,254272787	7,44567705	7,672425342	8,113742166		
PCP000993	8,315919398	8,462175047	8,752648252	8,168270966		
PCP001005	7,377904593	7,266786541	7,351645995	7,934693407	PCP001005	XM_009370752
PCP001017	7,276124405	7,426264755	7,491853096	8,276124405	PCP001017	XM_009364950
PCP001020	9,06520069	8,611024797	8,828676005	8,477070141	PCP001020	XM_008366707
PCP001021	7,711976642	7,486473046	7,813781191	7,360452072		
PCP001026	8,696967526	8,482485305	8,795487741	9,166589927	PCP001026	XM_008366705
PCP001045	8,459431619	8,553322101	8,63542792	9,028817757	PCP001045	XM_009345440
PCP001058	8,058911723	8,011227255	8,198052084	8,628153873	PCP001058	XM_018652144
PCP001059	8,780277286	8,658211483	8,515699838	8,348728154	PCP001059	XR_665215
PCP001067	8,17990909	8,235200503	8,3994702	8,647458426	PCP001067	XM_008373115
PCP001072	8,45532722	8,495855027	8,70276142	8,578674597		
PCP001089	7,562242424	7,351645995	7,688809791	8,026080745		
PCP001093	8,336863563	8,440163216	8,458078458	8,291539098		
PCP001108	8,288496992	8,798730993	8,122413888	7,475733431	PCP001108	XM_018644263
PCP001120	7,818070833	8,225593068	7,824449651	8,129283017	PCP001120	XM_009381039
PCP001127	8,06786455	7,677155841	7,994353437	7,884658968		
PCP001137	8,191454081	8,360495967	8,522267655	8,895817605		
PCP001142	7,807354922	7,700439718	7,792269724	8,388017285	PCP001142	XM_009343345
PCP001146	7,960001932	8,042589623	7,857980995	7,92677039		
PCP001148	8,460783512	8,614709844	8,501160452	8,449850215		
PCP001160	7,92677039	7,930737338	7,967687386	8,37794796		
PCP001188	7,309885571	6,926829678	7,254272787	7,06608919	PCP001188	XM_009375255
PCP001193	8,293103743	8,471675214	8,933690655	9,356826076		
PCP001196	7,473056289	7,515699838	7,470292816	7,363433935	PCP001196	XM_009362023
PCP001213	6,648609245	6,832890014	6,837060204	7,315874125	PCP001213	XM_009344146
PCP001215	7,531381461	7,624466495	7,639015048	7,459431619		
PCP001218	7,855927425	7,582480735	7,665335917	7,626950122	PCP001218	XM_018649367
PCP001219	8,453970202	8,049848549	7,845490051	7,377904593		
PCP001228	7,06608919	7,273049587	7,318949464	6,741466986		
PCP001240	7,936637939	7,321928095	7,693486957	7,876516947	PCP001240	XM_021966688
PCP001248	6,62935662	6,772545519	6,798698597	7,483815777		
PCP001256	7,572359168	7,377904593	7,523561956	8,042589623	PCP001256	XM_009378663
PCP001266	8,048050866	8,04072808	8,449850215	8,73470962	PCP001266	XM_009361340
PCP001268	8,909893084	8,520932916	8,922822231	8,735826768		
PCP001319	7,792269724	7,890750668	8,268331458	8,661778098	PCP001319	XM_009377024

PCP001320	6,442943496	6,942514505	7,156538193	7,497213158		
PCP001323	8,092757141	8,639051236	9,057991723	8,569855608	PCP001323	XM_009366598
PCP001337	6,857980995	7,42063398	7,400879436	7,342785837	PCP001337	XM_009358339
PCP001348	7,037052702	7,473056289	7,417852515	7,440204752	PCP001348	XM_009370066
PCP001357	8,503825738	8,612241904	8,578674597	8,540438054	PCP001357	XM_009350515
PCP001367	8,28077077	8,781359714	8,462175047	9,046223651	PCP001367	XM_008352450
PCP001373	7,536596918	7,73470962	7,592457037	7,375039431	PCP001373	XM_009379621
PCP001390	7,429030064	7,828644274	8,026080745	7,462134139	PCP001390	XM_008390002
PCP001402	8,082149041	8,268331458	8,33391564	8,727920455	PCP001402	XM_009357906
PCP001422	8,212715235	8,201290788	8,194756854	7,826548487	PCP001422	XM_018652316
PCP001442	7,975389442	8,038918989	8,357552005	8,63542792	PCP001442	XM_009375054
PCP001466	7,406587689	7,549438149	7,725673	8,174925683		
PCP001487	7,426264755	7,74819285	7,597456684	6,794415866		
PCP001492	6,62935662	6,794415866	7,06608919	7,50247382	PCP001492	XM_009346860
PCP001499	8,148069774	8,069691427	8,148069774	8,876516947	PCP001499	XM_018648989
PCP001500	6,95419631	7,06608919	6,914923239	6,64385619		
PCP001516	7,315874125	7,142923928	7,291585141	7,040782866	PCP001516	XM_018652464
PCP001527	6,86628983	6,837060204	6,965784285	7,510408147	PCP001527	XM_008342000
PCP001575	8,283875484	7,892816	7,924812504	7,523561956	PCP001575	XM_008365889
PCP001692	6,420718183	7,21916852	7,294620749	7,263691734	PCP001692	XM_009350813
PCP001697	6,667750232	6,890811455	7,189824559	7,609769734	PCP001697	XM_009350802
PCP001699	7,279192684	7,330916878	7,536596918	7,920829209		
PCP001702	8,395191361	8,106798463	8,423452115	8,944477815	PCP001702	XM_018649377
PCP001714	8,235200503	8,057071136	7,98111057	8,091012169	PCP001714	XM_008362383
PCP001724	8,148069774	8,110248388	8,174925683	8,031604721	PCP001724	XM_009350361
PCP001725	8,260543232	8,379378367	8,531381461	9,230428801		
PCP001726	7,815895594	8,247927513	7,969645536	7,928784831	PCP001726	XM_009350340
PCP001732	8,029728942	8,22881869	8,541754876	8,825499451		
PCP001748	7,641473777	7,868328714	7,922851957	7,71651083		
PCP001766	7,754887502	7,700439718	8,42488011	8,198052084	PCP001766	XM_009358223
PCP001793	6,81159947	7,037052702	7,324900589	7,442943496		
PCP001799	6,015024705	6,677085351	7,389480771	7,330916878	PCP001799	XM_018647481
PCP001807	8,363390129	7,977279923	7,50779464	7,209453366	PCP001807	XR_001953207
PCP001817	7,212666605	7,50779464	7,807354922	8,21757002		
PCP001820	8,235200503	8,622051819	8,279239123	8,09976859	PCP001820	XM_009358005
PCP001821	7,54689446	7,464913269	8,024197765	8,194756854	PCP001821	XM_009357995
PCP001826	8,204571144	8,224001674	8,174925683	8,118941073		
PCP001835	8,804131021	8,664162806	8,718806834	8,739206792	PCP001835	XM_009357816
PCP001846	7,14974712	7,965784285	7,541716163	7,481153605	PCP001846	XM_008341503
PCP001847	7,159871337	7,186559982	7,111970261	8,146339557	PCP001847	XM_009357611
PCP001858	8,758223215	8,664162806	8,646270682	8,523561956	PCP001858	XM_009345356
PCP001888	7,785746699	8,094500005	7,986809101	8,347267018		
PCP001922	8,075051225	7,92677039	8,018645301	7,518299077	PCP001922	XM_018647018
PCP001952	7,617136829	7,189824559	7,92677039	7,400879436	PCP001952	XM_009374554
PCP001953	7,273049587	7,003714662	7,108524457	7,634230226	PCP001953	XM_009374555
PCP001954	8,491853096	8,269921462	8,254225537	8,436003553	PCP001954	XM_009374556

PCP001977	8,136119594	7,536596918	7,622051819	7,196430001	PCP001977	XM_008376445
PCP001980	7,483815777	7,872397856	7,912889336	8,21106088		
PCP001986	8,058911723	8,560982378	8,713111526	9,059804103	PCP001986	XM_009348148
PCP001995	6,691115365	7,403693786	7,431873638	7,839203788	PCP001995	XM_009348276
PCP001997	7,934693407	8,222408523	8,279239123	8,95805965	PCP001997	XM_008376466
PCP002002	7,725673	7,662988741	7,884658968	7,094552786	PCP002002	XM_009348366
PCP002025	8,403736386	8,32791187	8,430452552	8,279239123	PCP002025	XM_009360661
PCP002033	7,990557838	8,21106088	8,154818109	8,622051819		
PCP002038	8,39373366	8,664162806	8,536558066	9,604238155	PCP002038	XR_001953384
PCP002064	6,513016928	6,882643049	6,984931073	7,634230226	PCP002064	XM_008365537
PCP002069	8,583722152	8,473015688	8,58747751	9,136991112		
PCP002075	7,30679083	7,589988142	8,009380767	8,309840108		
PCP002083	8,572397068	8,402287298	8,453970202	8,453970202	PCP002083	XM_017332622
PCP002086	8,493174961	8,364878797	8,588714636	8,965784285		
PCP002087	6,961970533	7,176621973	7,539158811	8,120652609	PCP002087	XM_009380128
PCP002091	7,723353774	7,587440004	7,896816753	8,413627929	PCP002091	XM_009364159
PCP002105	8,64385619	8,567309664	8,639051236	9,049848549	PCP002105	XM_009364191
PCP002106	7,579919292	7,960001932	8,290018847	8,64385619	PCP002106	XM_009364206
PCP002107	8,43325159	8,782441329	8,80196697	9,152284842	PCP002107	XM_009364205
PCP002115	8,176572111	8,139551352	8,130982335	8,577428828	PCP002115	XM_009380346
PCP002125	7,377904593	6,97349648	7,321928095	6,906890596	PCP002125	XM_009373581
PCP002129	8,517000043	8,518338423	8,770399609	9,211888295		
PCP002133	7,528805707	7,574934058	7,50517639	6,922792504	PCP002133	XM_009371408
PCP002151	8,357552005	8,277659361	8,426264755	8,888743249	PCP002151	XM_009347503
PCP002163	7,622051819	7,723353774	7,672425342	7,426264755		
PCP002165	7,105070402	7,014913158	6,874428132	6,768184325	PCP002165	XM_008393013
PCP002167	7,231989133	7,714245518	7,884658968	8,478446064	PCP002167	XM_009363083
PCP002197	7,303780748	7,073284692	7,254272787	7,076815597	PCP002197	XM_009338137
PCP002199	8,194756854	8,344295908	8,519636253	9,194756854	PCP002199	XM_009378432
PCP002212	7,803033252	7,839203788	7,772611496	7,453929061	PCP002212	XM_009341065
PCP002217	8,589950702	8,376472723	8,28077077	8,560982378	PCP002217	XM_009341054
PCP002258	7,440204752	7,727920455	7,761551232	8,188193194	PCP002258	XM_008386446
PCP002259	8,868297843	8,874458871	8,062477937	8,829722735	PCP002259	XM_009376105
PCP002263	7,910912508	7,46760555	7,579919292	7,098032083	PCP002263	XM_009376090
PCP002271	7,934693407	8,400879436	8,3994702	8,560982378		
PCP002273	6,898812977	6,691115365	6,981053471	7,688809791	PCP002273	XM_009353092
PCP002283	7,794415866	7,936637939	7,992485211	7,807354922	PCP002283	XM_018646366
PCP002304	6,763809907	6,857980995	7,098032083	7,510408147	PCP002304	XM_009356437
PCP002318	7,321928095	7,641473777	7,779194046	8,024197765		
PCP002339	7,698149009	7,732472726	7,92677039	7,684187561	PCP002339	XM_009366527
PCP002341	8,069691427	8,303780748	8,305286574	9,907386437		
PCP002344	7,582480735	7,765998774	7,882643049	8,129283017	PCP002344	XM_009366533
PCP002353	7,826548487	7,779194046	8,035238993	8,956143797	PCP002353	XM_008392634
PCP002376	8,782441329	8,581200582	8,74929961	8,609806663		
PCP002383	7,146390476	7,395148508	7,282347131	7,807354922	PCP002383	XM_009368859
PCP002384	8,396604781	8,058911723	8,106798463	7,557119022	PCP002384	XM_008359999

PCP002391	6,950351762	6,754887502	6,737010678	7,845490051	PCP002391	XM_009338448
PCP002398	7,285402219	7,212666605	7,481153605	7,783522135	PCP002398	XM_008382533
PCP002400	8,752648252	8,860062694	8,956143797	8,657032562		
PCP002418	8,776992236	8,80196697	8,78571401	8,794415866	PCP002418	XM_009361497
PCP002432	9,204571144	8,730198386	7,872397856	5,727920455	PCP002432	XM_009375270
PCP002439	8,181549844	8,026080745	7,599912842	6,745909573	PCP002439	XM_009375280
PCP002441	7,389480771	7,279192684	7,047996356	6,183288001	PCP002441	XM_009375284
PCP002446	7,718841075	8,429071922	8,335390355	8,833933692	PCP002446	XM_009359671
PCP002448	6,454011343	6,594996337	7,377904593	7,932687205	PCP002448	XM_021946109
PCP002455	7,022367813	8,042589623	8,477070141	9,515699838	PCP002455	XM_009350325
PCP002475	8,426264755	8,631795481	8,675957033	8,971543554	PCP002475	XM_008393403
PCP002494	7,938579853	7,888743249	8,029728942	8,62450305	PCP002494	XM_009363143
PCP002495	8,891783703	8,564797319	8,463524373	8,84862294	PCP002495	XM_009363140
PCP002517	8,215921225	7,813781191	8,04072808	7,832890014	PCP002517	XM_008373634
PCP002534	7,990557838	7,918863237	8,21106088	8,80196697	PCP002534	XM_009354782
PCP002551	7,129283017	6,996275749	7,574934058	7,300764373	PCP002551	XM_017330684
PCP002552	8,942514505	8,048050866	7,562242424	7,646234675	PCP002552	XM_009378264
PCP002562	8,722226922	8,742578916	8,795487741	8,68183575	PCP002562	XM_008356674
PCP002569	8,076815597	8,108524457	8,549476655	8,631795481	PCP002569	XM_009339879
PCP002573	7,423494135	7,562242424	7,783522135	7,448488033		
PCP002603	7,667679281	7,448488033	7,431873638	6,750472519	PCP002603	XM_008369615
PCP002625	7,888743249	8,09976859	8,122413888	8,481113232	PCP002625	XM_009356526
PCP002646	6,584962501	6,785681319	6,890811455	7,523561956	PCP002646	XM_008385605
PCP002656	8,173277373	8,353146825	8,483815777	8,743723645	PCP002656	XM_009360715
PCP002657	7,132679654	7,318949464	7,153095972	7,862141394	PCP002657	XM_009360714
PCP002680	7,251056285	7,617136829	8,060695932	8,960001932		
PCP002697	6,700439718	6,768184325	6,938638658	7,431873638	PCP002697	XM_009342221
PCP002700	8,920858975	8,828676005	8,695819867	8,348728154	PCP002700	XM_008359312
PCP002701	7,928784831	7,963878669	8,312882955	8,412231097		
PCP002706	7,636624621	7,494495616	7,400879436	7,360452072	PCP002706	XM_008366547
PCP002707	8,558420713	8,263644792	8,373604714	8,24474438	PCP002707	XM_008366547
PCP002708	7,942514505	7,759355602	7,798763389	7,785746699	PCP002708	XM_008358626
PCP002716	8,321928095	8,294620749	8,312882955	8,736976865		
PCP002718	8,103287808	7,967687386	8,144658243	8,571107931	PCP002718	XM_009358404
PCP002731	8,466260038	8,583722152	8,609806663	9,054387253		
PCP002751	8,112022407	7,658211483	7,785746699	7,225641265	PCP002751	XM_008382486
PCP002759	7,3458044	7,688809791	7,587440004	7,386552314		
PCP002761	7,199672345	7,312882955	7,403693786	7,866228001		
PCP002800	8,057071136	7,437377568	7,429030064	7,222360218	PCP002800	XM_009353048
PCP002812	7,395148508	6,837060204	7,539158811	8,481113232	PCP002812	XM_018646973
PCP002813	8,174925683	8,214319121	8,625708843	8,744833837	PCP002813	XM_009355813
PCP002870	7,845490051	7,403693786	7,366322214	7,462134139		
PCP002872	8,757123282	8,860062694	8,89684702	8,770399609	PCP002872	XM_009367619
PCP002876	8,420676082	8,288496992	8,330916878	8,779161208	PCP002876	XM_009372023
PCP002879	7,730164413	8,009380767	8,038918989	8,518338423		
PCP002896	8,675957033	8,501160452	8,670054263	8,306836389		

PCP002903	7,453929061	7,672425342	7,783522135	8,164906927		
PCP002911	6,579994693	6,938638658	7,415065677	7,456724026	PCP002911	XM_008394335
PCP002920	8,555854491	8,672425342	8,667714757	8,586201784	PCP002920	XM_009377751
PCP002949	8,252665432	8,092757141	8,314379315	7,984874125	PCP002949	XM_009360955
PCP002960	7,790055203	8,225593068	8	7,544346278	PCP002960	XM_009340877
PCP002987	7,807354922	7,695785075	7,826548487	7,30679083	PCP002987	XM_009348671
PCP003036	7,139551352	7,251056285	7,238404739	6,894817763	PCP003036	XM_018643502
PCP003059	7,321928095	7,142923928	7,330916878	7,910912508	PCP003059	XM_008377890
PCP003076	7,510408147	7,641473777	7,882643049	8,038918989		
PCP003083	8,21916852	8,17990909	7,90285744	8,118941073	PCP003083	XM_009342581
PCP003098	8,666508075	8,536558066	8,564797319	8,925791779	PCP003098	XM_008395081
PCP003108	7,960001932	8,091012169	8,166564822	7,686500527		
PCP003130	7,111970261	7,241554209	7,686500527	8,14974712	PCP003130	XM_009340054
PCP003146	8,247927513	8,41079052	8,37794796	9,06608919	PCP003146	XM_008387090
PCP003164	7,06608919	6,619559738	6,969587981	7,609769734	PCP003164	XM_008353311
PCP003171	6,732404887	6,639087423	7,163196797	7,369204723	PCP003171	XM_009349107
PCP003215	6,95419631	6,965784285	6,988684687	7,531381461	PCP003215	XM_009353599
PCP003272	7,115511897	7,163196797	7,651051691	7,269874722	PCP003272	XM_018648325
PCP003285	8	7,914863459	7,589988142	7,670089681	PCP003285	XM_009374603
PCP003286	8,33391564	7,251056285	7,273049587	6,72342204	PCP003286	XM_009362138
PCP003288	7,914863459	7,888743249	8,026080745	8,431831861	PCP003288	XM_008352706
PCP003290	6,906890596	7,029784146	7,285402219	7,526147081		
PCP003297	8,572397068	8,980139578	8,693486957	8,530094158	PCP003297	XR_667719
PCP003310	8,648645193	8,511752654	8,63542792	8,438791853		
PCP003318	7,765998774	7,587440004	7,787902559	8,440163216	PCP003318	XM_018648315
PCP003329	6,54426899	6,691115365	6,965784285	7,549438149		
PCP003331	8,016808288	7,922851957	8,018645301	8,67479253		
PCP003348	8,677120596	8,552015799	8,554588852	8,581200582		
PCP003360	6,894817763	7,169925001	7,076815597	6,984931073	PCP003360	XM_009358061
PCP003368	7,815895594	7,839203788	8,075051225	8,361943774		
PCP003400	7,392317423	7,257387843	7,339850003	7,173227395	PCP003400	XM_009351185
PCP003427	6,898812977	6,727920455	7,17990909	8,395191361	PCP003427	XM_009369089
PCP003439	7,383704292	7,475733431	7,609769734	8,094500005	PCP003439	XM_018649828
PCP003458	7,395148508	7,412188747	7,42063398	7,928784831		
PCP003471	6,898812977	6,938638658	7,300764373	7,705079392	PCP003471	XM_009376822
PCP003472	7,597456684	7,309885571	7,8008999	8,380807357	PCP003472	XM_008343241
PCP003477	7,336908182	7,475733431	7,321928095	7,044394119	PCP003477	XM_009378940
PCP003485	8,28077077	8,520932916	8,700439718	9,380828997	PCP003485	XM_009378931
PCP003490	7,849686575	7,965784285	7,992485211	7,826548487	PCP003490	XM_009378924
PCP003515	8,314379315	8,314379315	8,305286574	8,678318438		
PCP003530	7	6,554588852	7,186559982	6,84962403	PCP003530	XM_009336069
PCP003556	8,75043902	8,693486957	8,754887502	8,596189756	PCP003556	XM_008372409
PCP003574	7,412188747	7,247927513	7,285402219	7,894817763	PCP003574	XM_009353633
PCP003580	8,158205728	7,853808564	8,300718622	8,064311643	PCP003580	XM_009353620
PCP003581	6,619559738	6,745909573	6,969587981	7,510408147	PCP003581	XM_009353615
PCP003595	7,541716163	7,592457037	7,475733431	8,075051225		

PCP003609	7	6,857980995	7,094552786	7,847558521	PCP003609	XM_008364601
PCP003618	8,535275377	8,212715235	8,614709844	8,939579214	PCP003618	XM_008383445
PCP003626	7,018700931	7,263691734	7,681800619	8,592457037	PCP003626	XM_008360041
PCP003627	7,557119022	7,118941073	7,641473777	7,930737338		
PCP003632	7,062531903	7,251056285	7,142923928	6,686500527	PCP003632	XM_009362388
PCP003634	6,890811455	6,886672074	7,125878364	7,841281284	PCP003634	XM_009362390
PCP003636	7,415065677	7,159871337	7,559721231	8,290018847	PCP003636	XM_008383484
PCP003637	7,845490051	7,739240553	7,992485211	8,282300792	PCP003637	XM_009362402
PCP003640	7,794415866	8,026080745	8,154818109	8,557157324		
PCP003644	7,209453366	7,206232954	7,118941073	7,61227877		
PCP003651	7,406587689	7,101503009	6,946379968	7,183188734	PCP003651	XM_017336465
PCP003655	8,001858526	7,918863237	8,132731056	7,960001932	PCP003655	XM_008372027
PCP003662	9,13442632	8,536558066	8,654636029	8,763776717	PCP003662	XM_018650885
PCP003665	7,564759219	7,254272787	7,523561956	7,266786541	PCP003665	XM_009373718
PCP003673	7,372168569	7,303780748	7,196430001	6,64385619	PCP003673	XM_009361876
PCP003686	8,705044823	8,694636474	8,75043902	9,128432607	PCP003686	XM_009361852
PCP003696	7,273049587	7,377904593	7,481153605	7,824449651		
PCP003706	7,554588852	7,843418611	7,592457037	7,231989133	PCP003706	XM_018651145
PCP003708	7,406587689	7,526147081	7,688809791	8,001858526	PCP003708	XM_009374888
PCP003715	7,822284016	8,031604721	8,196380818	8,420676082	PCP003715	XM_009374882
PCP003723	7,912889336	7,745976779	7,882643049	8,348728154		
PCP003725	7,998139076	8,293103743	8,5824431	8,983934156	PCP003725	XM_009368394
PCP003727	7,315874125	8,141238626	8,17990909	7,898873426	PCP003727	XM_009368385
PCP003752	8,396604781	8,262094845	8,326429487	8,194756854	PCP003752	XM_008376230
PCP003753	8,767091963	8,87958325	8,711942238	8,43325159	PCP003753	XM_008376220
PCP003754	8,891783703	8,685344507	8,522267655	8,907882108	PCP003754	XM_008376199
PCP003757	7,982993575	8,184875343	8,271463028	8,466260038	PCP003757	XM_008376152
PCP003758	7,50247382	7,639015048	7,604886762	8,035238993	PCP003758	XM_008376144
PCP003773	6,62935662	6,862079387	6,874428132	7,400879436		
PCP003787	7,684187561	7,71651083	7,665335917	7,602364826	PCP003787	XM_009368796
PCP003790	8,064311643	7,986809101	8,199672345	8,880593701	PCP003790	XM_009368798
PCP003793	7,273049587	7,372168569	7,055282436	6,714245518	PCP003793	XM_009368804
PCP003796	8,554588852	8,266786541	8,225593068	8,247927513		
PCP003806	6,303780748	6,448405435	7,136170875	7,528805707	PCP003806	XM_009369711
PCP003813	7,950293432	7,526147081	7,531381461	7,857980995	PCP003813	XM_008362831
PCP003814	7,674757228	7,348728154	7,736943052	7,95419631	PCP003814	XM_018649922
PCP003817	8,33391564	8,233619677	8,459431619	8,798730993	PCP003817	XM_009369730
PCP003826	8,451211112	8,318904285	8,262094845	8,254225537	PCP003826	XM_018649001
PCP003867	8,296136161	8,323415108	8,584962501	8,312882955	PCP003867	XM_009345820
PCP003879	8,536558066	8,494535617	8,579956993	8,154818109	PCP003879	XM_009367445
PCP003885	8,057071136	7,759355602	7,790055203	8,329437582	PCP003885	XM_009366817
PCP003886	7,244791942	7,453929061	7,658211483	7,894817763		
PCP003899	8,239980333	9,017727086	9,297672907	8,375039431	PCP003899	XM_009366846
PCP003906	6,454011343	6,81159947	7,166615031	7,589988142	PCP003906	XM_009344264
PCP003910	8,156487632	8,024197765	7,796558821	7,409390936	PCP003910	XM_009379819
PCP003912	7,166615031	7,282347131	7,569855608	8,39373366	PCP003912	XM_009379825

PCP003916	8,482485305	8,614709844	8,614709844	8,513056419		
PCP003917	7,813781191	7,826548487	7,952275317	8,473015688	PCP003917	XM_008370686
PCP003930	8,042589623	7,862141394	8,04072808	8,660602089	PCP003930	XM_009379856
PCP003931	8,230404783	8,348728154	8,297695831	8,696967526	PCP003931	XM_009379857
PCP003939	8,494535617	8,112022407	8,513056419	8,136119594	PCP003939	XR_665079
PCP003965	7,269874722	7,018700931	7,641473777	7,624466495	PCP003965	XM_009362300
PCP003966	7,868328714	7,934693407	8,124121312	8,42488011	PCP003966	XM_009362299
PCP003983	6,988684687	6,853870927	7,42063398	7,541716163	PCP003983	NM_001293861
PCP003988	8,21106088	8,222408523	8,440163216	8,762647796	PCP003988	XM_009371045
PCP004005	7,894817763	7,584962501	7,672425342	8,227230852	PCP004005	XM_009371076
PCP004014	8,201290788	8,54303182	8,732438807	8,898843202		
PCP004051	7,50779464	7,166615031	7,153095972	6,486553493	PCP004051	XM_008343777
PCP004052	8,482485305	8,724513853	8,531381461	8,347267018	PCP004052	XM_017327284
PCP004058	7,456724026	7,587440004	7,828644274	7,619633096		
PCP004080	7,330916878	7,63175911	7,878541438	7,409390936	PCP004080	XM_008344331
PCP004083	6,815959618	6,759289016	7,062531903	7,609769734		
PCP004116	7,291585141	7,559721231	7,646234675	7,934693407	PCP004116	XM_018651464
PCP004123	7,156538193	7,403693786	7,686500527	8,166564822		
PCP004138	7,285402219	7,429030064	7,562242424	7,894817763	PCP004138	XM_009335967
PCP004148	8,129283017	8,468949809	8,77478706	9,350939182	PCP004148	XM_009337807
PCP004151	6,31596467	6,700439718	6,918863237	7,864186145	PCP004151	XM_009366774
PCP004152	6,981053471	7,173227395	7,574934058	7,757089938	PCP004152	XM_009337834
PCP004186	8,189824559	8,350187812	8,405141463	9,20701432		
PCP004188	8,891783703	8,901862467	8,908902953	8,398016818	PCP004188	XM_008384441
PCP004200	7,14974712	7,324900589	7,62935662	7,741466986		
PCP004202	8,54303182	8,296136161	8,48651327	7,990557838	PCP004202	XM_009347819
PCP004212	7,395148508	7,321928095	7,50247382	7,965784285	PCP004212	XM_018647006
PCP004213	7,231989133	7,129283017	7,714245518	8,224001674	PCP004213	XM_009355930
PCP004234	7,047996356	7,266786541	7,247927513	7,754887502	PCP004234	XM_009369571
PCP004254	7,815895594	7,539158811	7,723353774	7,64385619		
PCP004276	5,101397952	5,841218374	7,646234675	9,094526396	PCP004276	XM_008359795
PCP004282	8,125826717	7,988684687	8,139551352	8,572397068	PCP004282	XM_009371716
PCP004291	6,828707735	6,589913261	6,737010678	7,336908182	PCP004291	XM_009357283
PCP004319	7,604886762	7,721099189	7,684187561	7,42063398	PCP004319	XM_009347248
PCP004320	7,651051691	7,934693407	7,890750668	7,587440004	PCP004320	XM_009347250
PCP004345	7,199672345	7,475733431	6,857980995	6,914923239	PCP004345	XM_018651235
PCP004349	7,464913269	7,491853096	7,241554209	7,336908182		
PCP004359	7,176621973	7,549438149	7,431873638	7,324900589	PCP004359	XM_017324919
PCP004379	7,273049587	6,934634441	6,926829678	6,798698597	PCP004379	XM_009376546
PCP004380	8,420676082	8,416459769	8,258942852	8,142974967		
PCP004400	8,592457037	8,715378619	8,438791853	8,360495967		
PCP004408	7,582480735	7,646234675	7,884658968	8,498530123	PCP004408	XM_009366026
PCP004410	8,584962501	8,341318667	8,485145023	9,18157469	PCP004410	XM_008357533
PCP004418	7,651051691	8,078577814	7,914863459	7,982993575		
PCP004420	8,215921225	8,463524373	8,803065551	8,899870461	PCP004420	XM_008390607
PCP004434	8,559682997	8,495855027	8,584962501	8,972491628		

PCP004437	8,623259662	8,297695831	8,427648072	8,137862104	PCP004437	XM_009364151
PCP004440	6,569855608	6,857980995	7,040782866	7,515699838	PCP004440	XM_008354562
PCP004450	6,448405435	6,958030641	7,115511897	8,948367232	PCP004450	XM_009380469
PCP004471	8,118941073	8,174925683	8,17990909	8,092757141	PCP004471	XM_008340343
PCP004473	7,515699838	6,934634441	7,069637728	6,737010678	PCP004473	XM_008389353
PCP004478	8,618385502	8,779161208	8,758223215	8,757123282	PCP004478	XM_018645962
PCP004485	7,464913269	7,260496187	7,860031646	7,653418353	PCP004485	XM_009348803
PCP004494	7,963878669	8,171577143	8,495855027	8,912889336	PCP004494	XR_001952113
PCP004498	8,791162889	8,776992236	8,84024291	8,688844752	PCP004498	XM_008368145
PCP004553	7,44567705	7,291585141	7,539158811	7,866228001	PCP004553	XM_009345191
PCP004567	7,28845085	7,339850003	7,336908182	7,193081765		
PCP004573	6,464831606	6,894817763	7,291585141	7,499845887	PCP004573	XM_008343615
PCP004584	8,247927513	7,599912842	8,422064766	9,695228291	PCP004584	XM_009341259
PCP004593	7,372168569	7,536596918	7,528805707	8,117227504	PCP004593	XM_018647710
PCP004611	7,018700931	7,047996356	7,294620749	7,807354922	PCP004611	XM_009371591
PCP004618	6,832890014	6,579994693	6,99242856	8,243173983	PCP004618	XM_008381594
PCP004653	8,481113232	8,773699688	8,626913629	8,21916852	PCP004653	XM_008341806
PCP004666	7,434628228	7,74819285	7,599912842	7,50247382	PCP004666	XM_009370964
PCP004672	8,502513601	8,416459769	8,453970202	8,347267018	PCP004672	XM_018648007
PCP004695	8,30833903	8,379378367	8,21757002	8,277659361	PCP004695	XM_008350202
PCP004720	7,855927425	7,886733033	7,88062431	7,489205728		
PCP004744	6,890811455	7,029784146	7,28845085	6,841344192	PCP004744	XM_009355812
PCP004764	8,703903573	8,662953148	8,792237182	9,101529272	PCP004764	XM_009361366
PCP004786	8,910882535	8,735826768	8,658211483	8,475733431		
PCP004810	8,249492747	8,564797319	8,314379315	8,696967526	PCP004810	XM_008369003
PCP004822	7,50779464	7,260496187	7,518299077	7,193081765	PCP004822	XM_009361983
PCP004852	7,315874125	7,202907418	7,040782866	6,614709844		
PCP004960	7,61227877	7,963878669	7,798763389	7,765998774	PCP004960	XM_009338918
PCP004961	6,662917555	7,372168569	6,902917719	7,658211483	PCP004961	XM_008390706
PCP004970	7,462134139	6,798698597	7,30679083	7,426264755	PCP004970	XM_009338940
PCP004997	7,389480771	7,327956767	7,459431619	8,271463028	PCP004997	XM_009363407
PCP005005	7,602364826	7,718841075	7,42063398	7,567347696		
PCP005015	8,14974712	7,860031646	8,038918989	7,794415866		
PCP005016	8,08390476	7,750405521	8,382278171	9,606090543	PCP005016	XM_009363378
PCP005044	8,087462841	7,992485211	7,910912508	7,63175911		
PCP005062	7,882643049	7,900866808	8,003770871	7,478405614	PCP005062	XM_018652121
PCP005073	8,28692734	8,159871337	8,265239967	8,029728942		
PCP005074	7,924812504	7,761551232	7,924812504	8,30833903	PCP005074	XM_009341260
PCP005076	7,062531903	8,014968933	8,382278171	10,83709964	PCP005076	XM_009341258
PCP005082	6,392317423	7,321928095	6,392317423	7,790055203		
PCP005088	7,796558821	7,868328714	8,073231127	8,536558066		
PCP005096	8,251103639	8,075051225	7,832890014	7,122362117	PCP005096	XM_009380061
PCP005110	7,247927513	7,173227395	7,333870928	8,423452115	PCP005110	XR_001953357
PCP005116	8,266786541	8,159871337	8,35169016	8,642665475	PCP005116	XM_009360373
PCP005119	8,460783512	8,501160452	8,659389441	8,945443836		
PCP005129	6,759289016	6,961970533	7,389480771	7,624466495	PCP005129	XM_009360353

PCP005143	7,098032083	7,159871337	7,040782866	6,965784285		
PCP005149	7,672425342	7,526147081	7,688809791	7,357552005	PCP005149	XM_009358490
PCP005162	7,073284692	7,357552005	7,564759219	7,71651083	PCP005162	XM_009358469
PCP005167	8,153146652	8,198052084	8,562242424	9,123267853	PCP005167	XM_009358462
PCP005168	7,988684687	8,037107627	8,001858526	8,493174961	PCP005168	XM_009358459
PCP005172	7,406587689	7,50517639	7,832890014	7,241554209	PCP005172	XM_008388353
PCP005176	6,930737338	6,691115365	7,003714662	7,351645995	PCP005176	XM_009358447
PCP005183	7,142923928	7,46760555	7,28845085	7,040782866		
PCP005184	7,609769734	7,711976642	7,711976642	7,54689446	PCP005184	XM_011466072
PCP005199	7,604886762	7,990557838	8,252665432	9,172427509	PCP005199	XM_018650255
PCP005224	6,81159947	7,297649983	7,238404739	7,058857621	PCP005224	XM_009340847
PCP005240	8,164906927	8,108524457	7,944448531	7,28845085	PCP005240	XM_017323704
PCP005254	7,750405521	7,952275317	8,39373366	9,026053072	PCP005254	XR_666720
PCP005266	8,282300792	8,204571144	8,532667615	8,407989993	PCP005266	XM_018652320
PCP005269	7,562242424	7,607330314	7,759355602	8,254225537	PCP005269	XM_009380207
PCP005274	8,69115027	8,420676082	8,282300792	7,912889336		
PCP005284	7,225641265	7,389480771	7,718841075	7,944448531		
PCP005301	8,220765252	8,458078458	8,710806434	8,41079052	PCP005301	XM_018647077
PCP005315	8,273002948	8,390899794	8,372125027	8,922822231	PCP005315	XM_009369242
PCP005323	9,1807297	8,239980333	9,012149614	8,146339557	PCP005323	XM_009369261
PCP005332	7,196430001	7,46760555	7,357552005	7,231989133		
PCP005334	8,626913629	8,780277286	8,97441459	8,782441329	PCP005334	XM_008350718
PCP005338	8,39373366	8,497173232	8,666508075	9,002815016	PCP005338	XR_001787178
PCP005339	8,014968933	7,714245518	7,963878669	7,462134139	PCP005339	XM_009374468
PCP005355	7,986809101	8,037107627	8,181549844	8,522267655		
PCP005357	8,016808288	7,684187561	7,783522135	7,624466495	PCP005357	XM_009380153
PCP005364	7,50517639	6,54426899	7,260496187	6,80309785	PCP005364	XM_009380161
PCP005374	7,597456684	8,382278171	8,406545173	9,272233193	PCP005374	NM_001328902
PCP005379	7,318949464	7,462134139	7,670089681	8,060695932	PCP005379	XM_009375909
PCP005380	7,456724026	7,291585141	7,922851957	7,815895594	PCP005380	XM_009375907
PCP005385	8,762647796	8,831845581	8,924812504	8,76927586	PCP005385	XM_018651378
PCP005399	6,380764075	6,695854658	7,142923928	8,315919398	PCP005399	XM_009341412
PCP005425	6,559644763	6,750472519	6,878602742	7,372168569	PCP005425	XM_009357893
PCP005440	8,441574774	8,586201784	8,692336525	8,532667615	PCP005440	XM_009357924
PCP005473	7,163196797	7,080337882	7,263691734	6,624539604	PCP005473	XM_009346139
PCP005474	7,864186145	7,655852677	8,098032083	8,24474438		
PCP005484	7,073284692	7,562242424	7,894817763	8,554588852	PCP005484	XM_008344092
PCP005486	8,448446735	8,463524373	8,541754876	8,241601875	PCP005486	XM_009378561
PCP005507	7,166615031	7,108524457	7,665335917	8,458078458		
PCP005518	7,910912508	7,592457037	7,890750668	8,142974967		
PCP005527	8,545620932	8,350187812	8,75043902	8,711942238		
PCP005528	8,46760555	8,449850215	8,836050355	9,418548384	PCP005528	XM_009375454
PCP005530	7,536596918	7,559721231	7,843418611	8,489165579	PCP005530	XM_009375456
PCP005531	8,470333494	8,156487632	8,080391184	7,862141394	PCP005531	XM_009375457
PCP005570	8,204571144	8,514398461	8,225593068	7,936637939	PCP005570	XM_009362501
PCP005572	8,350187812	7,969645536	7,63175911	7,166615031	PCP005572	XM_009355756

PCP005573	7,681800619	8,029728942	7,73470962	8,04072808	PCP005573	XM_009351615
PCP005578	7,868328714	7,42063398	7,730164413	7,339850003	PCP005578	XM_009344811
PCP005591	6,564835417	6,874428132	6,934634441	7,202907418	PCP005591	XM_009378141
PCP005606	8,35169016	8,564797319	8,929761415	9,378663341	PCP005606	XM_009342522
PCP005608	8,206184105	8,220765252	8,345760055	8,158205728		
PCP005610	7,058857621	7,54689446	7,423494135	7,772611496	PCP005610	XM_009342514
PCP005620	6,857980995	6,609843592	7,051698368	7,247927513	PCP005620	XM_008384299
PCP005621	8,215921225	8,209453366	8,314379315	8,978224236	PCP005621	XM_008384300
PCP005627	8,082149041	8,174925683	7,952275317	7,619633096	PCP005627	XM_009346199
PCP005636	7,63175911	7,87036472	7,982993575	8,196380818		
PCP005650	8,110248388	8,236827423	8,258942852	8,139551352		
PCP005656	7,330916878	7,660566438	7,412188747	7,544346278		
PCP005665	7,360452072	7,254272787	7,403693786	7,876516947	PCP005665	XM_009370664
PCP005675	8,273002948	8,501160452	8,436003553	7,826548487	PCP005675	XM_009356135
PCP005678	8,583722152	8,74708524	8,725638921	8,206184105	PCP005678	XM_009356125
PCP005684	7,46760555	7,473056289	7,42063398	6,781359714	PCP005684	XM_009352772
PCP005707	8,607330314	8,603626345	8,636624621	8,584962501	PCP005707	XM_009378491
PCP005721	8,294620749	8,311385042	8,41079052	8,775873612	PCP005721	XM_009378773
PCP005741	8,053491515	7,95419631	7,979167931	8,526186214		
PCP005744	7,634230226	7,159871337	6,996275749	6,934634441		
PCP005748	6,845490051	6,727920455	7,040782866	7,429030064	PCP005748	XM_009357861
PCP005750	7,677155841	7,62935662	7,88062431	7,677155841	PCP005750	XM_009357855
PCP005751	6,918863237	7,579919292	7,453929061	7,725673	PCP005751	XM_009357850
PCP005757	8,441574774	8,54303182	9,049848549	9,203763987	PCP005757	XM_009357841
PCP005761	7,090959258	6,934634441	7,199672345	6,81159947	PCP005761	XM_009364826
PCP005762	7,730164413	7,906890596	7,95419631	8,383704292		
PCP005776	8,110248388	7,732472726	8,033423002	8,290018847		
PCP005777	8,033423002	7,992485211	8,173277373	8,564797319		
PCP005784	8,044394119	7,944448531	8,201290788	7,924812504		
PCP005793	8,089212109	8,120652609	8,136119594	7,822284016	PCP005793	XM_018645479
PCP005795	8,296136161	8,215921225	8,184875343	8,603626345		
PCP005813	7,409390936	7,686500527	7,300764373	7,557119022		
PCP005817	7,77478706	7,956114749	8,158205728	7,960001932	PCP005817	XM_018645949
PCP005827	6,981053471	7,173227395	6,763809907	6,737010678	PCP005827	XM_018647575
PCP005855	7,736943052	7,61227877	7,574934058	7,50517639	PCP005855	XM_009361775
PCP005862	7,257387843	7,058857621	7,518299077	7,942514505	PCP005862	XM_009361683
PCP005874	6,677085351	6,853870927	7,047996356	7,572359168	PCP005874	XM_009348614
PCP005886	7,847558521	7,423494135	7,417852515	6,695854658	PCP005886	XM_018649286
PCP005889	8,17990909	8,239980333	8,178266468	8,662953148	PCP005889	XM_009366560
PCP005890	6,727920455	7,163196797	7,366322214	7,920829209	PCP005890	XM_009366559
PCP005898	8,299208018	8,569855608	8,306836389	7,815895594	PCP005898	XM_018651877
PCP005903	6,853870927	7,662988741	7,098032083	7,037052702	PCP005903	XM_009368630
PCP005917	7,377904593	8,110248388	8,299208018	8,847589839	PCP005917	XM_009342616
PCP005925	7,950293432	7,8008999	7,918863237	7,617136829	PCP005925	XM_017325949
PCP005926	8,016808288	7,63175911	7,655852677	7,153095972	PCP005926	XM_008384235
PCP005944	8,353146825	8,268331458	8,395191361	8,255831156	PCP005944	XM_009342617

PCP005953	6,930737338	7,539158811	7,868328714	8,668884984		
PCP005968	7,146390476	7,3458044	7,315874125	7,711976642	PCP005968	XM_009372450
PCP005975	8,39373366	8,591223118	8,62450305	9,059804103	PCP005975	XM_009340497
PCP005977	7,336908182	7,115511897	7,386552314	8,031604721	PCP005977	XM_009340492
PCP005980	7,431873638	7,732472726	7,783522135	8,026080745		
PCP005991	7,906890596	7,544346278	8,154818109	8,214319121	PCP005991	XM_008346989
PCP005993	7,956114749	8,026080745	7,992485211	7,912889336	PCP005993	XM_009337471
PCP006039	7,415065677	7,639015048	7,658211483	8,08390476	PCP006039	XM_009369479
PCP006044	7,864186145	8,176572111	7,864186145	7,199672345		
PCP006047	8,35169016	8,255831156	8,344295908	8,075051225	PCP006047	XM_009375889
PCP006048	7,08395793	7,324900589	7,285402219	7,768184325	PCP006048	XR_664884
PCP006086	7,437377568	7,206232954	7,330916878	7,260496187	PCP006086	XM_008350269
PCP006091	7,723353774	7,670089681	7,651051691	7,531381461		
PCP006093	8,098032083	8,071462363	8,051643995	7,440204752	PCP006093	XM_009375915
PCP006100	8,342830273	7,904905525	8,110248388	8,417852515		
PCP006108	7,950293432	7,815895594	8,075051225	8,68183575	PCP006108	XM_008345096
PCP006113	7,660566438	7,851749041	7,022367813	5,832890014	PCP006113	XM_009375132
PCP006121	6,988684687	7,321928095	7,494495616	8,001858526	PCP006121	XM_018648841
PCP006172	7,876516947	7,839203788	7,946438456	7,739240553	PCP006172	XM_009359150
PCP006175	6,874428132	6,777025123	6,84962403	7,636624621	PCP006175	XM_009359154
PCP006176	8,166564822	8,096293483	8,265239967	8,044394119		
PCP006190	6,54951516	7,30679083	7,670089681	7,815895594		
PCP006214	7,08395793	6,914923239	7,212666605	7,592457037	PCP006214	XM_009374163
PCP006216	8,519636253	8,54689446	8,540438054	8,901862467		
PCP006224	6,926829678	7,489205728	7,723353774	8,009380767	PCP006224	XM_008361449
PCP006226	8,950322598	8,139551352	8,527477006	8,713111526	PCP006226	XM_009358466
PCP006229	7,42063398	7,406587689	7,417852515	7,146390476	PCP006229	XM_009344656
PCP006235	8,613494819	8,609806663	8,591223118	8,491853096	PCP006235	XM_009335949
PCP006241	8,495855027	8,375039431	8,436003553	9,000929563	PCP006241	XM_009367544
PCP006299	7,967687386	7,904905525	7,641473777	6,977279923	PCP006299	XM_008360236
PCP006305	7,403693786	7,489205728	7,523561956	7,324900589	PCP006305	XM_018645795
PCP006315	7,567347696	7,604886762	7,6794801	8,120652609	PCP006315	XM_008342446
PCP006342	7,456724026	7,478405614	7,442943496	7,354646096	PCP006342	XM_009342491
PCP006369	7,087462841	7,330916878	7,602364826	7,922851957	PCP006369	XM_009378066
PCP006383	7,584962501	7,541716163	7,294620749	7,040782866		
PCP006413	8,32791187	8,113742166	7,948367232	7,602364826	PCP006413	XM_018651433
PCP006433	6,732404887	6,732404887	7,040782866	7,348728154		
PCP006438	7,189824559	7,051698368	7,406587689	7,815895594	PCP006438	XM_009340480
PCP006486	7,958088658	8,024197765	8,198052084	8,703903573	PCP006486	XM_018647112
PCP006487	6,902917719	6,87036472	7,014913158	7,533952624	PCP006487	XM_018649333
PCP006548	8,046196362	8,303780748	8,416459769	8,022367813		
PCP006549	7,908872939	7,796558821	7,88062431	7,772611496	PCP006549	XM_009367739
PCP006568	6,705010253	6,857980995	6,965784285	7,658211483		
PCP006583	8,722226922	8,583722152	8,596189756	8,39373366	PCP006583	XM_009372596
PCP006585	7,309885571	7,266786541	7,486473046	7,996219247	PCP006585	XM_018650613
PCP006596	7,464913269	6,981053471	7,297649983	7,481153605	PCP006596	XM_009372630

PCP006599	6,409390936	6,906890596	6,853870927	7,423494135	PCP006599	XM_008372051
PCP006608	7,940577883	8,092757141	7,655852677	6,981053471	PCP006608	XM_008372038
PCP006629	7,087462841	6,824386003	6,950351762	6,714245518		
PCP006647	8,523561956	8,576181982	8,592457037	8,54689446	PCP006647	XM_008376195
PCP006654	8,873444113	8,735826768	8,545620932	8,139551352	PCP006654	XM_008358374
PCP006662	8,075051225	8,038918989	8	7,924812504	PCP006662	XM_008358378
PCP006673	8,194756854	8,176572111	8,386595423	9,120678526	PCP006673	XM_008376237
PCP006674	7,173227395	7,136170875	8,191454081	9,905883363		
PCP006688	8,344295908	8,61717357	8,912889336	9,369226538	PCP006688	XM_009340819
PCP006691	7,383704292	7,189824559	8,057071136	7,17990909	PCP006691	XM_009340881
PCP006697	8,060695932	8,550746785	8,759322309	9,184875343	PCP006697	XM_009340998
PCP006710	8,589950702	8,398016818	8,677120596	9,184057088		
PCP006745	8,434628228	8,5824431	8,736976865	8,614709844	PCP006745	XM_009371206
PCP006746	8,321928095	8,338379842	8,555854491	8,919846558	PCP006746	XM_009371204
PCP006747	7,040782866	7,235248379	7,21916852	7,665335917	PCP006747	XM_009371202
PCP006762	7,992485211	8,189824559	8,360495967	8,601139355		
PCP006766	8,810571635	8,372125027	8,268331458	8,320439548	PCP006766	XM_009371166
PCP006767	8,258942852	8,246360579	8,491853096	8,845490051		
PCP006769	6,981053471	7,026025399	7,442943496	7,672425342	PCP006769	XM_009371232
PCP006771	3,368768349	6,351734323	9,54303182	12,97560977	PCP006771	NM_001293928
PCP006776	8,50779464	8,568602197	8,645045922	8,540438054	PCP006776	XM_009353306
PCP006778	6,996275749	6,97349648	7,047996356	8,347267018		
PCP006817	7,653418353	7,636624621	8,236827423	8,176572111	PCP006817	XM_017332533
PCP006822	6,815959618	7,14974712	7,244791942	7,783522135	PCP006822	XM_008235058
PCP006833	8,055282436	8,571107931	8,604849706	9,004698008	PCP006833	XM_008370962
PCP006835	7,051698368	7,63175911	7,820178962	8,423452115		
PCP006839	7,30679083	7,125878364	7,169925001	7,026025399	PCP006839	XM_008368155
PCP006840	7,342785837	7,497213158	7,602364826	7,087462841	PCP006840	XM_009359506
PCP006852	8,158205728	8,363390129	8,660602089	8,474395481		
PCP006864	8,189824559	7,303780748	8,020535537	8,350187812	PCP006864	XM_008356073
PCP006866	8,662953148	8,096293483	8,024197765	7,50517639	PCP006866	XM_017328303
PCP006906	7,101503009	7,022367813	7,440204752	8,018645301		
PCP006908	7,960001932	7,944448531	8,161535025	8,795487741	PCP006908	XM_008347833
PCP006916	8,77148947	8,835008208	8,809510912	8,709669735	PCP006916	XM_008373679
PCP006933	7,129283017	6,589913261	7,040782866	7,073284692	PCP006933	XM_008373658
PCP006945	7,202907418	7,567347696	7,582480735	7,936637939		
PCP006957	7,698149009	7,569855608	7,6794801	7,50247382	PCP006957	XM_009367364
PCP006967	7,193081765	7,254272787	7,058857621	6,996275749		
PCP006973	7,839203788	7,938579853	7,761551232	8,290018847	PCP006973	XM_009352882
PCP006975	8,347267018	7,803033252	7,83080039	7,739240553	PCP006975	XM_018646292
PCP006991	7,483815777	7,392317423	7,375039431	7,857980995		
PCP006996	7,470292816	7,971543554	7,50779464	8,038918989		
PCP006999	7,539158811	7,667679281	8,016808288	8,294620749		
PCP007000	7,363433935	7,707359132	7,843418611	7,95419631	PCP007000	XM_008378548
PCP007008	8,944477815	8,796591265	8,77478706	8,762647796		
PCP007024	6,60481265	7,058857621	7,520972191	7,920829209	PCP007024	XM_009355866

PCP007025	7,924812504	8,110248388	8,625708843	8,926800034	PCP007025	XM_009355862
PCP007053	7,950293432	7,988684687	8,096293483	8,658211483	PCP007053	XM_009338069
PCP007059	8,127581695	8,420676082	8,632995197	9,277682605	PCP007059	XM_009350042
PCP007067	7,969645536	8,151422517	7,898873426	8,569855608	PCP007067	XM_009359409
PCP007080	7,429030064	7,42063398	7,459431619	6,97349648		
PCP007085	7,884658968	7,781359714	7,845490051	8,548166865		
PCP007088	8,604849706	8,83709175	8,646270682	8,166564822		
PCP007099	8,341318667	8,141238626	7,95419631	7,499845887	PCP007099	XM_009360519
PCP007103	7,727920455	7,938579853	7,634230226	7,95419631	PCP007103	XM_009360517
PCP007115	7,824449651	7,674757228	7,918863237	8,985841937		
PCP007126	8,515699838	8,498530123	8,716545126	9,054387253		
PCP007153	8,073231127	7,723353774	7,77478706	8,033423002	PCP007153	XM_018644347
PCP007160	9,167418146	8,820178962	8,528766645	8,255831156	PCP007160	XM_008383213
PCP007167	7,357552005	7,497213158	7,518299077	7,973439079	PCP007167	XM_009364844
PCP007168	8,235200503	7,662988741	7,415065677	5,746043983	PCP007168	XM_009364840
PCP007169	6,62935662	6,878602742	7,058857621	7,448488033	PCP007169	XM_009364831
PCP007189	8,082149041	7,779194046	7,494495616	7,489205728		
PCP007191	8,422064766	8,395191361	8,243173983	8,212715235	PCP007191	XM_009354495
PCP007205	7,398059585	7,494495616	7,790055203	8,001858526	PCP007205	XR_001952619
PCP007224	8,379378367	8,364878797	8,479780264	9,027905997	PCP007224	XM_018647760
PCP007225	7,189824559	7,440204752	7,386552314	7,860031646	PCP007225	XM_018647759
PCP007240	7,473056289	7,189824559	7,073284692	6,886672074	PCP007240	XM_018646976
PCP007243	7,169925001	7,159871337	8,144658243	7,50247382	PCP007243	XM_009355829
PCP007244	8,315919398	8,430452552	8,586201784	9,396604781		
PCP007245	6,686500527	6,961970533	8,684152487	12,60478486		
PCP007270	7,303780748	7,868328714	7,868328714	8,254225537	PCP007270	XM_008359473
PCP007277	7,260496187	7,303780748	7,765998774	8,511752654	PCP007277	XM_009348038
PCP007278	8,173277373	8,189824559	8,290018847	8,013071384	PCP007278	XM_009347081
PCP007282	7,807354922	7,961912672	7,956114749	7,626950122		
PCP007292	8,274587815	8,348728154	8,949330653	9,296916207		
PCP007296	7,886733033	7,886733033	7,979167931	7,665335917	PCP007296	XM_009374328
PCP007305	6,857980995	7,241554209	7,273049587	7,730164413	PCP007305	XM_009374344
PCP007310	7,653418353	7,868328714	7,62935662	7,327956767	PCP007310	XM_009341430
PCP007327	7,667679281	7,587440004	7,400879436	6,841344192	PCP007327	XM_009372196
PCP007333	8,144658243	8,060695932	8,014968933	8,011227255		
PCP007336	7,743689989	7,54689446	7,562242424	7,470292816	PCP007336	XM_018644293
PCP007338	8,136119594	7,900866808	7,886733033	7,6794801	PCP007338	XM_018644805
PCP007345	7,46760555	7,564759219	7,660566438	8,146339557		
PCP007379	6,832890014	7,515699838	7,303780748	7,098032083	PCP007379	XM_008381355
PCP007380	7,206232954	7,146390476	7,403693786	7,77036657	PCP007380	XM_009380873
PCP007381	7,163196797	6,845490051	6,934634441	7,491853096	PCP007381	XM_018644153
PCP007382	7,725673	8,241601875	8,13442632	8,178266468		
PCP007390	7,884658968	7,752681699	7,837123296	7,651051691	PCP007390	XM_008340838
PCP007398	7,244791942	7,282347131	7,279192684	7,813781191	PCP007398	XM_009349847
PCP007412	8,481113232	8,60855054	8,533991546	8,380807357	PCP007412	XM_009370201
PCP007415	7,257387843	7,225641265	7,531381461	7,87036472		

PCP007420	7,209453366	7,225641265	7,212666605	7,772611496	PCP007420	XM_018642842
PCP007439	8,001858526	8,091012169	8,169925001	7,990557838	PCP007439	XM_017324307
PCP007455	7,541716163	7,510408147	7,950293432	8,41079052	PCP007455	XM_008340006
PCP007464	7,723353774	7,794415866	8,018645301	8,975360779		
PCP007472	8,009380767	8,055282436	8,406545173	9,208648936	PCP007472	XM_008340197
PCP007476	7,040782866	7,330916878	7,730164413	8,053491515	PCP007476	XM_009351553
PCP007488	7,672425342	7,855927425	7,772611496	7,489205728	PCP007488	XM_009341709
PCP007495	7,294620749	7,473056289	7,375039431	7,910912508	PCP007495	XM_009376575
PCP007498	8,341318667	8,188193194	8,315919398	9,184057088	PCP007498	XM_008342183
PCP007508	7,783522135	8,055282436	7,321928095	7,386552314	PCP007508	XM_009360013
PCP007512	7,212666605	7,196430001	6,807354922	6,99242856	PCP007512	XM_009352463
PCP007524	7,938579853	8,046196362	8,315919398	8,163247124	PCP007524	XM_018649941
PCP007525	7,750405521	8,129283017	8,620842965	9,01402047	PCP007525	XR_668727
PCP007526	7,354646096	7,173227395	7,886733033	7,988684687	PCP007526	XM_009369777
PCP007534	6,619559738	6,785681319	7,098032083	7,718841075	PCP007534	XM_009354650
PCP007542	7,333870928	7,567347696	7,572359168	7,380850638	PCP007542	XR_530190
PCP007554	7,111970261	7,594921715	7,809478757	8,357552005	PCP007554	XM_018649812
PCP007564	8,037107627	8,115459877	8,031604721	7,577428828	PCP007564	XM_009369666
PCP007566	7,960001932	7,481153605	7,564759219	6,837060204	PCP007566	XM_009369676
PCP007570	7,837123296	8,144658243	8,357552005	8,619596417	PCP007570	XM_018652392
PCP007575	7,932687205	8,306836389	8,266786541	8,45532722	PCP007575	XM_009380594
PCP007578	7,826548487	8,158205728	8,137862104	7,765998774	PCP007578	XM_009339434
PCP007585	7,46760555	7,71651083	7,826548487	8,087462841		
PCP007592	8,08571145	8,091012169	8,136119594	8,539158811	PCP007592	XM_008371011
PCP007599	8,214319121	8,291539098	8,194756854	8,122413888	PCP007599	XM_009373954
PCP007609	7,456724026	7,451211112	7,46760555	7,193081765		
PCP007611	7,754887502	7,662988741	8,21106088	7,741466986	PCP007611	XM_018646928
PCP007613	7,333870928	7,375039431	7,251056285	6,327866971	PCP007613	XM_009363917
PCP007624	8,478446064	8,260543232	8,452570726	8,407989993		
PCP007629	8,026080745	7,934693407	7,785746699	7,781359714	PCP007629	XM_009347088
PCP007637	7,238404739	7,622051819	7,98111057	8,451211112	PCP007637	XM_009348216
PCP007656	7,826548487	7,862141394	7,95419631	8,400879436	PCP007656	XM_008394018
PCP007660	8,144658243	8,379378367	8,46760555	9,036173613		
PCP007662	8,181549844	8,341318667	8,426264755	8,667714757	PCP007662	XM_009347174
PCP007666	8,74819285	8,736976865	8,885696373	8,732438807	PCP007666	XM_009338368
PCP007707	8,574896225	8,470333494	8,584962501	8,423452115		
PCP007719	8,3994702	8,048050866	8,247927513	8,409390936	PCP007719	NM_001328974
PCP007724	7,977279923	7,653418353	7,792269724	7,768184325		
PCP007729	7,044394119	7,14974712	6,988684687	6,807354922	PCP007729	XM_018645307
PCP007730	8,535275377	8,649866903	8,68183575	9,023283079	PCP007730	XM_018644911
PCP007742	7,845490051	7,702726796	7,594921715	7,215969746	PCP007742	XM_009368366
PCP007777	7,634230226	7,567347696	7,497213158	7,17990909	PCP007777	XM_009378370
PCP007797	7,139551352	8,071462363	7,587440004	7,554588852	PCP007797	XM_009359008
PCP007868	8,342830273	8,108524457	7,768184325	7,375039431	PCP007868	XM_018643374
PCP007886	8,653454181	8,918863237	8,724513853	8,654636029	PCP007886	XM_009371586
PCP007951	7,303780748	7,541716163	7,592457037	7,932687205	PCP007951	XM_018643059

PCP007999	8,578674597	8,537878433	8,363390129	8,225593068	PCP007999	XM_009362647
PCP008037	7,658211483	7,607330314	7,523561956	7,263691734		
PCP008072	6,882643049	7,186559982	7,156538193	7,579919292	PCP008072	XM_017330193
PCP008107	6,894817763	6,902917719	7,132679654	6,81159947	PCP008107	XM_017333913
PCP008121	6,926829678	6,820178962	6,781359714	7,743689989	PCP008121	XM_007210528
PCP008146	8,057071136	7,803033252	7,77036657	7,244791942	PCP008146	XR_526770
PCP008179	6,357552005	6,464831606	7,541716163	7,843418611		
PCP008184	7,044394119	7,111970261	7,169925001	7,695785075	PCP008184	XM_009356845
PCP008188	7,257387843	7,166615031	7,442943496	7,824449651	PCP008188	XM_009356840
PCP008208	8,204571144	7,794415866	7,648681141	7,475733431		
PCP008223	7,111970261	7,115511897	7,63175911	8,330916878		
PCP008227	7,412188747	7,366322214	7,660566438	7,383704292		
PCP008231	8,186510462	7,77036657	7,779194046	7,798763389	PCP008231	XM_009338531
PCP008236	8,317412614	8,544307635	8,927777962	8,962896005		
PCP008240	8,277659361	8,403736386	8,385129006	8,321928095		
PCP008252	8,265239967	8,156487632	8,127581695	8,122413888	PCP008252	XM_009363946
PCP008255	8,363390129	8,087462841	7,969645536	8,159871337	PCP008255	XM_009363949
PCP008263	8,144658243	7,998139076	8,06786455	7,714245518	PCP008263	XM_009363962
PCP008274	7,369204723	7,453929061	7,77036657	8,645045922	PCP008274	XM_009363978
PCP008277	7,723353774	7,318949464	7,309885571	7,22881869	PCP008277	XM_008373989
PCP008290	8,117227504	8,392317423	8,567309664	8,532667615	PCP008290	XM_008361898
PCP008306	8,263644792	7,938579853	7,695785075	7,22881869		
PCP008311	7,609769734	7,71651083	8,158205728	8,87036472		
PCP008317	7,448488033	7,572359168	7,684187561	8,171577143		
PCP008328	6,785681319	6,634157606	6,700439718	8,548166865	PCP008328	XM_008391642
PCP008332	7,464913269	7,665335917	7,739240553	8,024197765		
PCP008336	7,176621973	7,238404739	7,587440004	7,950293432	PCP008336	XM_009344880
PCP008347	6,491853096	6,837060204	7,08395793	7,333870928	PCP008347	XM_009344855
PCP008350	7,375039431	7,609769734	8,073231127	9,240791332	PCP008350	XM_009344852
PCP008361	7,988684687	7,779194046	7,845490051	7,705079392	PCP008361	XM_009356613
PCP008363	8,14974712	8,139551352	8,373604714	8,918863237		
PCP008374	8,130982335	7,918863237	8,176572111	8,511752654	PCP008374	XM_018647159
PCP008378	7,839203788	7,820178962	7,695785075	7,426264755		
PCP008379	7,864186145	7,745976779	7,776959347	7,6794801	PCP008379	XM_009356583
PCP008393	8,742578916	8,21916852	7,900866808	7,324900589	PCP008393	XM_018643553
PCP008399	7,28845085	7,46760555	7,584962501	7,942514505	PCP008399	XM_009340928
PCP008405	6,653490009	6,845490051	6,926829678	7,360452072	PCP008405	XM_009335868
PCP008429	8,714245518	8,760453835	8,762647796	8,630558365	PCP008429	XM_009335815
PCP008435	7,776959347	7,765998774	7,811663685	7,475733431	PCP008435	XM_009335808
PCP008439	7,426264755	7,105070402	7,369204723	7,153095972	PCP008439	XM_009335796
PCP008451	7,481153605	7,069637728	7,30679083	7,176621973	PCP008451	XM_009368882
PCP008453	8,725638921	8,713111526	8,739206792	8,62935662	PCP008453	XM_009343236
PCP008470	7,63175911	7,718841075	7,759355602	8,77807713	PCP008470	XM_009336863
PCP008483	8,634193917	8,639051236	8,753785022	9,282323962	PCP008483	XM_009336884
PCP008484	7,90285744	7,515699838	7,42063398	6,50779464	PCP008484	XM_009354790
PCP008486	8,853839746	8,438791853	8,356099782	7,934693407		

PCP008489	8,485145023	8,544307635	8,618385502	8,501160452	PCP008489	XM_009354806
PCP008492	6,898812977	7,087462841	6,785681319	6,718772592	PCP008492	XM_009354816
PCP008521	8,279239123	8,631795481	8,779161208	9,031577154	PCP008521	XM_018645290
PCP008524	8,215921225	8,382278171	8,359002767	8,094500005		
PCP008525	7,136170875	7,033423002	7,285402219	7,805227956	PCP008525	XM_017326474
PCP008532	7,183188734	7,486473046	7,559721231	8,013071384		
PCP008537	8,130982335	7,884658968	7,757089938	7,721099189		
PCP008540	8,740354199	8,597419437	8,742578916	8,531381461		
PCP008550	8,191454081	8,038918989	8,091012169	8,044394119	PCP008550	XM_009339396
PCP008552	6,946379968	6,918863237	6,946379968	7,453929061	PCP008552	XM_009339391
PCP008570	6,491853096	6,491853096	7,051698368	7,536596918		
PCP008579	8,347267018	8,60855054	8,69115027	9,395169935	PCP008579	XM_009360977
PCP008581	8,285402219	7,973439079	8,122413888	8,092757141	PCP008581	XR_667583
PCP008593	7,241554209	7,058857621	7,094552786	6,47037417		
PCP008595	8,513056419	8,43325159	8,431831861	8,207844058	PCP008595	XM_009361007
PCP008599	6,777025123	6,984931073	6,965784285	8,56350137	PCP008599	XM_009368122
PCP008601	8,309840108	8,016808288	8,130982335	8,188193194		
PCP008606	7,330916878	7,406587689	7,562242424	8,057071136	PCP008606	XM_008369883
PCP008610	7,597456684	7,614709844	6,996275749	7,318949464		
PCP008611	8,06608919	7,908872939	7,754887502	7,732472726	PCP008611	NM_001293880
PCP008617	6,988684687	6,87036472	7,523561956	8,980139578	PCP008617	NM_001319807
PCP008622	7,483815777	7,483815777	7,456724026	7,330916878		
PCP008629	8,031604721	8,816983623	7,950293432	7,244791942	PCP008629	NM_001319813
PCP008639	8,698114274	8,882643049	8,692336525	8,909893084	PCP008639	XM_009374580
PCP008645	7,510408147	7,022367813	7,037052702	6,718772592	PCP008645	XM_018643538
PCP008647	8,48651327	8,528766645	8,653454181	8,459431619	PCP008647	XM_009340820
PCP008649	8,04072808	8,422064766	8,471675214	9,034331283	PCP008649	XM_008348559
PCP008650	7,486473046	8,129283017	8,13442632	8,440163216	PCP008650	XM_008348559
PCP008655	7,908872939	7,61227877	6,977279923	6,589913261	PCP008655	XM_008376392
PCP008679	8,743723645	8,72904287	8,780277286	8,631795481	PCP008679	XM_008373783
PCP008704	7,518299077	7,189824559	7,247927513	7,321928095	PCP008704	XM_018648461
PCP008707	8,357552005	8,154818109	8,037107627	8,191454081	PCP008707	XM_008228891
PCP008708	8,452570726	8,027905997	8,158205728	8,154818109	PCP008708	XM_009348498
PCP008711	8,773699688	8,649866903	8,230404783	7,30679083	PCP008711	XM_009363630
PCP008746	7,965784285	8,014968933	7,878541438	8,434628228		
PCP008762	7,08395793	7,142923928	7,225641265	7,688809791	PCP008762	XM_009361351
PCP008763	8,653454181	9,039823818	8,830768706	8,606109055	PCP008763	XM_009348952
PCP008779	8,360495967	8,370687407	8,341318667	8,201290788	PCP008779	XM_009359079
PCP008790	7,977279923	7,986809101	7,988684687	8,482485305	PCP008790	XM_009367212
PCP008796	8,005624549	7,510408147	7,572359168	7,423494135	PCP008796	XM_008350552
PCP008798	7,763743526	7,781359714	8,069691427	9,599912842	PCP008798	XM_009338220
PCP008806	7,805227956	7,781359714	7,832890014	7,658211483	PCP008806	XM_018648114
PCP008808	6,695854658	6,97349648	7,029784146	7,377904593	PCP008808	XM_009361039
PCP008813	7,914863459	7,851749041	7,894817763	7,743689989	PCP008813	XM_009361025
PCP008815	7,950293432	7,641473777	7,839203788	7,711976642	PCP008815	XM_009361028
PCP008835	7,389480771	7,63175911	7,824449651	8,336863563	PCP008835	XM_009362322

PCP008839	7,63175911	7,139551352	7,125878364	6,886672074	PCP008839	XM_009362442
PCP008865	8,236827423	8,293103743	8,54689446	8,791162889	PCP008865	XM_009355631
PCP008866	7,757089938	7,50517639	7,843418611	8,06608919	PCP008866	XM_009355627
PCP008872	8,456682963	8,21916852	8,752648252	7,912889336	PCP008872	XM_009355621
PCP008879	7,372168569	8,204571144	8,020535537	10,00562455		
PCP008881	7,718841075	7,990557838	8,101555535	8,330916878		
PCP008921	7,77478706	7,956114749	8,485145023	9,283088353		
PCP008922	8,626913629	8,732438807	8,447083226	8,601139355	PCP008922	XM_009380252
PCP008951	8,661778098	8,353146825	8,527477006	8,812722827	PCP008951	XM_009346688
PCP008960	6,709704193	6,942514505	6,930737338	7,684187561	PCP008960	XM_009344473
PCP008973	8,029728942	7,71651083	7,470292816	7,132679654	PCP008973	XM_008343267
PCP008988	7,003714662	6,878602742	7,069637728	7,528805707	PCP008988	XM_009361161
PCP008990	8,060695932	8,049848549	8,064311643	8,588714636	PCP008990	XM_018648139
PCP008992	7,366322214	7,520972191	7,541716163	8,163247124		
PCP009012	7,415065677	7,380850638	7,386552314	6,667750232		
PCP009014	8,266786541	8,125826717	8,227230852	8,611024797	PCP009014	XM_009344910
PCP009023	6,886672074	6,828707735	6,958030641	7,772611496	PCP009023	XM_008342278
PCP009040	8,125826717	8,227230852	8,39373366	8,171577143	PCP009040	XM_009375746
PCP009044	7,339850003	7,101503009	7,440204752	6,741466986	PCP009044	XM_009375752
PCP009045	7,183188734	7,014913158	7,196430001	6,47037417	PCP009045	XM_009375754
PCP009064	8,049848549	8,220765252	7,752681699	7,772611496	PCP009064	NM_001302278
PCP009067	7,681800619	7,567347696	7,569855608	7,415065677	PCP009067	XR_001951378
PCP009076	8,602401945	8,367764188	8,398016818	8,370687407	PCP009076	XM_017333814
PCP009080	8,073231127	7,922851957	8,239980333	8,464872438	PCP009080	XM_008349114
PCP009089	7,380850638	7,342785837	7,451211112	6,984931073	PCP009089	XM_009348734
PCP009099	8,429071922	8,075051225	8,283875484	8,154818109	PCP009099	XR_668149
PCP009100	8,130982335	8,181549844	8,501160452	8,707359132		
PCP009105	7,475733431	7,589988142	7,526147081	8,007475849		
PCP009114	8,247927513	8,290018847	8,35169016	8,797661526	PCP009114	XM_008368462
PCP009128	8,572397068	8,257387843	8,58747751	8,17990909	PCP009128	XM_018651364
PCP009130	8,120652609	8,236827423	8,680640826	8,70158371	PCP009130	XM_009375863
PCP009164	8,388017285	8,448446735	8,733591606	9,036173613		
PCP009168	8,813781191	8,642665475	8,614709844	8,745943176	PCP009168	XM_009378026
PCP009200	7,339850003	7,554588852	7,73470962	7,922851957	PCP009200	XM_009364558
PCP009266	6,95419631	6,266786541	6,934634441	7,312882955		
PCP009269	7,434628228	7,309885571	7,544346278	7,924812504		
PCP009276	8,191454081	8,055282436	8,39373366	9,19802752	PCP009276	XM_009340422
PCP009292	8,612241904	8,458078458	8,485145023	8,875503635		
PCP009303	7,166615031	6,958030641	7,383704292	7,677155841	PCP009303	XM_009338247
PCP009318	7,783522135	7,754887502	7,807354922	7,533952624	PCP009318	XM_008373639
PCP009325	7,098032083	7,186559982	7,872397856	8,299208018	PCP009325	XM_009351731
PCP009330	8,426264755	8,297695831	8,475733431	8,332394659	PCP009330	XM_009349554
PCP009332	7,375039431	7,111970261	7,50517639	8,011227255	PCP009332	XM_009358875
PCP009343	8,589950702	8,354602022	8,423452115	8,431831861	PCP009343	XM_009343488
PCP009356	7,037052702	7,222360218	7,464913269	7,686500527	PCP009356	XM_009361448
PCP009369	7,291585141	7,693486957	7,90285744	8,257387843		

PCP009370	7,574934058	7,87036472	7,824449651	8,412231097	PCP009370	XM_009373971
PCP009377	7,330916878	7,589988142	7,339850003	6,639087423		
PCP009392	8,520932916	8,288496992	8,341318667	8,055282436	PCP009392	XM_009343278
PCP009400	9,186535222	7,739240553	6,695854658	7,984874125	PCP009400	XM_007226460
PCP009401	8,919846558	7,714245518	7,309885571	8,552015799	PCP009401	XM_009341425
PCP009402	8,241601875	8,19313106	8,087462841	8,06608919		
PCP009457	7,297649983	7,176621973	7,50247382	7,90285744	PCP009457	XR_001952562
PCP009464	6,695854658	6,820178962	6,95419631	7,8008999		
PCP009481	7,330916878	6,981053471	6,886672074	7,459431619	PCP009481	XM_008341786
PCP009484	7,336908182	7,604886762	7,634230226	7,475733431	PCP009484	XM_021967594
PCP009494	7,383704292	7,300764373	7,572359168	8,096293483	PCP009494	XM_009344778
PCP009518	7,360452072	7,470292816	7,225641265	7,193081765		
PCP009532	8,74819285	8,660602089	8,522267655	8,506486109	PCP009532	XM_009367546
PCP009561	7,30679083	6,705010253	7,849686575	7,309885571	PCP009561	XM_008377173
PCP009562	8,626913629	8,198052084	8,490530019	8,32494558	PCP009562	XM_018644783
PCP009568	6,60481265	6,759289016	6,977279923	7,619633096	PCP009568	XM_009359255
PCP009587	7,209453366	7,209453366	7,193081765	7,918863237	PCP009587	XM_008390293
PCP009594	6,564835417	6,594996337	7,153095972	7,54689446	PCP009594	XM_009362026
PCP009610	6,918863237	7,090959258	7,260496187	7,518299077	PCP009610	XM_008387276
PCP009612	3,700439718	5,700439718	7,494495616	13,51545346	PCP009612	NM_001302321
PCP009615	8,695819867	8,523561956	8,459431619	8,622051819	PCP009615	XM_009362416
PCP009702	6,464831606	7,21916852	7,209453366	6,81159947		
PCP009707	7,007531912	6,958030641	7,826548487	8,348728154	PCP009707	XM_009358413
PCP009721	6,639087423	6,930737338	7,094552786	8,033423002		
PCP009727	8,875503635	8,434628228	8,19313106	8,202956379	PCP009727	XM_009358601
PCP009746	7,212666605	7,199672345	7,437377568	7,105070402	PCP009746	XM_009358928
PCP009754	7,235248379	7,269874722	6,878602742	6,820178962	PCP009754	XM_009359050
PCP009773	7,998139076	8,049848549	8,332394659	8,509101985	PCP009773	XM_009359213
PCP009781	6,303780748	6,50779464	7,058857621	8,024197765		
PCP009786	7,478405614	6,579994693	7,549438149	6,375039431	PCP009786	XM_009360622
PCP009792	6,695854658	6,857980995	7,118941073	7,377904593	PCP009792	XM_009359413
PCP009798	8,285402219	8,199672345	8,353146825	8,727920455	PCP009798	XM_009359457
PCP009831	7,624466495	7,776959347	7,636624621	7,639015048	PCP009831	XM_009345697
PCP009835	7,497213158	7,235248379	7,718841075	7,636624621	PCP009835	XM_009344877
PCP009836	8,08390476	7,494495616	8,178266468	8,13442632	PCP009836	XM_009344798
PCP009837	8,35169016	8,120652609	7,886733033	7,956114749	PCP009837	XM_009344446
PCP009846	8,891783703	8,453970202	8,839203788	8,667714757	PCP009846	XM_009354801
PCP009850	6,988684687	7,014913158	7,528805707	8,214319121	PCP009850	XM_009343193
PCP009864	6,732404887	6,60481265	6,882643049	7,691185174	PCP009864	XM_009341364
PCP009865	7,982993575	8,184875343	8,265239967	7,732472726	PCP009865	XM_008376597
PCP009876	8,247927513	8,224001674	7,936637939	6,934634441	PCP009876	XM_009357786
PCP009879	7,624466495	7,938579853	8,044394119	8,235200503	PCP009879	XM_009357782
PCP009884	7,975389442	7,971543554	7,853808564	7,372168569	PCP009884	XM_009357772
PCP009901	7,743689989	7,952275317	7,936637939	7,837123296		
PCP009906	8,273002948	8,19313106	8,026080745	7,658211483	PCP009906	XM_009357736
PCP009912	8,395191361	8,33391564	8,74708524	9,447765141	PCP009912	XM_009357734

PCP009918	7,294620749	7,437377568	7,589988142	7,285402219	PCP009918	XM_018647403
PCP009931	7,564759219	7,597456684	7,574934058	8,044394119		
PCP009945	7,533952624	7,429030064	7,478405614	7,982993575		
PCP009952	6,86628983	6,97349648	7,300764373	7,554588852	PCP009952	XM_017333468
PCP009961	7,705079392	7,395148508	6,820178962	6,50779464	PCP009961	XM_008377864
PCP009971	7,798763389	7,333870928	7,582480735	7,003714662	PCP009971	NM_001293824
PCP009995	6,886672074	7,363433935	7,076815597	7,202907418		
PCP010002	7,044394119	7,582480735	7,499845887	7,539158811	PCP010002	XM_008347883
PCP010027	8,04072808	8,092757141	7,714245518	8,21106088		
PCP010031	8,062477937	8,243173983	8,33391564	7,958088658	PCP010031	XM_009365010
PCP010043	7,528805707	7,757089938	7,681800619	7,572359168	PCP010043	XM_008376470
PCP010055	7,453929061	7,118941073	7,026025399	6,609843592		
PCP010074	8,716545126	8,392317423	8,30833903	8,031604721	PCP010074	XM_009351730
PCP010081	7,297649983	7,183188734	7,423494135	7,92677039	PCP010081	XM_008356440
PCP010087	7,824449651	7,884658968	8,161535025	8,564797319	PCP010087	XM_009342465
PCP010094	8,326429487	8,490530019	8,474395481	8,874458871	PCP010094	XR_001951834
PCP010107	8,007475849	7,95419631	8,154818109	7,809478757	PCP010107	XM_009357459
PCP010121	8,120652609	8	8,24474438	8,62450305	PCP010121	XM_018645141
PCP010131	8,471675214	8,548166865	8,880593701	9,407267764	PCP010131	XM_009340847
PCP010132	7,377904593	7,646234675	7,594921715	8,215921225		
PCP010134	7,369204723	6,594996337	7,136170875	7,768184325	PCP010134	XM_008389148
PCP010135	8,402287298	8,464872438	8,453970202	7,431873638	PCP010135	XM_009346157
PCP010156	8,110248388	7,940577883	7,824449651	7,417852515		
PCP010157	6,965784285	7,389480771	7,750405521	8,533991546	PCP010157	XM_009375997
PCP010165	8,499845887	8,649866903	8,708497652	7,572359168	PCP010165	XM_009375993
PCP010167	7,351645995	7,584962501	7,700439718	7,992485211	PCP010167	XM_009375986
PCP010168	7,750405521	7,849686575	8,539158811	8,968666793		
PCP010174	7,864186145	8,014968933	8,161535025	7,906890596		
PCP010194	7,837123296	7,973439079	8,367764188	7,723353774	PCP010194	XM_009374105
PCP010205	8,003770871	7,74819285	8,171577143	8,528766645	PCP010205	XM_009342413
PCP010218	7,536596918	7,646234675	7,562242424	7,423494135		
PCP010232	8,151422517	8,400879436	8,442943496	8,238404739		
PCP010238	7,17990909	7,058857621	6,845490051	6,662917555		
PCP010245	8,291539098	8,451211112	8,474395481	8,807354922	PCP010245	XM_009376160
PCP010251	7,111970261	7,153095972	7,539158811	7,942514505	PCP010251	XM_009376144
PCP010261	8,598685286	8,423452115	8,306836389	8,020535537		
PCP010266	7,579919292	7,339850003	7,330916878	7,022367813	PCP010266	XM_009376170
PCP010274	8,311385042	8,21916852	8,42488011	9,008428622	PCP010274	XM_009351257
PCP010286	7,544346278	7,707359132	7,805227956	8,14974712	PCP010286	XM_009339593
PCP010287	7,273049587	7,225641265	7,646234675	8,222408523	PCP010287	XM_018643260
PCP010297	8,33391564	8,553322101	8,725638921	9,177419538		
PCP010299	8,431831861	8,568602197	8,660602089	8,894817763	PCP010299	XM_009351169
PCP010311	7,336908182	8,247927513	8,482485305	8,659389441		
PCP010328	7,199672345	7,139551352	7,718841075	7,982993575	PCP010328	XM_009376393
PCP010331	7,594921715	7,375039431	7,486473046	7,415065677		
PCP010333	6,99242856	7,225641265	7,176621973	7,768184325		

PCP010344	7,579919292	7,357552005	7,579919292	7,920829209	PCP010344	XM_018651517
PCP010353	7,429030064	6,732404887	7,087462841	7,263691734	PCP010353	XM_009371421
PCP010385	7,998139076	8,120652609	7,772611496	8,224001674	PCP010385	XM_009357033
PCP010386	8,233619677	8,417852515	8,357552005	8,117227504	PCP010386	XM_009357040
PCP010393	7,672425342	7,202907418	7,189824559	6,554588852	PCP010393	XM_009357051
PCP010395	8,354602022	8,596189756	8,860062694	9,099794885	PCP010395	XM_008346038
PCP010402	8,110248388	8,024197765	8,031604721	7,765998774	PCP010402	XM_008393671
PCP010410	8,584962501	8,583722152	8,571107931	8,451211112	PCP010410	XR_524695
PCP010413	7,513095909	7,163196797	7,383704292	7,090959258	PCP010413	XM_009369351
PCP010414	7,389480771	7,073284692	7,50779464	8,332394659		
PCP010419	7,866228001	7,988684687	8,204571144	8,466260038		
PCP010422	7,375039431	7,453929061	7,624466495	8,064311643	PCP010422	XM_009369405
PCP010424	8,21916852	8,108524457	8,262094845	8,06608919	PCP010424	XM_009369408
PCP010433	8,184875343	7,942514505	8,215921225	8,602401945	PCP010433	XM_009374172
PCP010439	7,667679281	6,62935662	6,942514505	7,609769734	PCP010439	XM_008366708
PCP010442	7,572359168	7,63175911	7,559721231	7		
PCP010446	7,055282436	7,014913158	7,342785837	7,732472726	PCP010446	XM_008378914
PCP010456	7,491853096	7,520972191	7,222360218	6,672425342	PCP010456	XM_008358909
PCP010457	8,369248353	8,463524373	8,68183575	8,937609223		
PCP010473	8,859006685	8,645045922	8,660602089	8,482485305	PCP010473	XM_009351511
PCP010540	6,667750232	6,950351762	7,026025399	7,614709844	PCP010540	XM_009337073
PCP010551	8,623259662	8,442943496	8,678318438	8,976334992		
PCP010606	7,992485211	8,493174961	8,202956379	7,820178962	PCP010606	XM_018646178
PCP010627	7,431873638	7,055282436	7,011227255	7,06608919		
PCP010628	6,196331634	6,475733431	6,922792504	8,057071136	PCP010628	XM_018642724
PCP010630	7,977279923	7,910912508	7,667679281	8,168270966		
PCP010644	6,914923239	7,260496187	7,440204752	7,938579853	PCP010644	XM_008373724
PCP010650	8,144658243	8,277659361	8,586201784	9,079484784	PCP010650	XM_008355007
PCP010651	8,21757002	8,189824559	8,158205728	7,958088658	PCP010651	XM_008356815
PCP010665	7,98111057	7,475733431	7,956114749	8,164906927	PCP010665	XM_009343748
PCP010666	8,762647796	8,586201784	8,753785022	8,632995197	PCP010666	XM_009343726
PCP010687	8,482485305	8,103287808	8,129283017	8,497173232		
PCP010712	8,606109055	8,599912842	8,845490051	9,064284694		
PCP010725	8,271463028	8,207844058	8,285402219	8,09976859		
PCP010762	8,631795481	8,623259662	8,752648252	9,24951645		
PCP010765	7,552054236	7,88062431	7,998139076	8,388017285		
PCP010770	8,721099189	8,357552005	8,660602089	8,84862294	PCP010770	XM_009350864
PCP010805	8,651051691	8,523561956	8,620842965	9,080364533		
PCP010814	7,783522135	8,303780748	8,613494819	8,80196697	PCP010814	XM_009344328
PCP010816	8,54689446	8,682994584	8,398016818	8,329437582	PCP010816	XR_530144
PCP010819	7,451211112	7,952275317	8,049848549	7,736943052	PCP010819	XM_008349312
PCP010822	7,324900589	7,655852677	7,499845887	7,266786541	PCP010822	XR_001953538
PCP010826	8,031604721	8,215921225	8,798730993	8,744833837	PCP010826	XM_009352047
PCP010839	8,463524373	8,62935662	8,827596761	9,080364533		
PCP010843	8,220765252	8,098032083	8,17990909	8,087462841	PCP010843	XM_017333412
PCP010847	7,843418611	7,646234675	7,592457037	7,50779464	PCP010847	XM_008394498

PCP010853	8,537878433	8,354602022	8,483815777	8,468949809		
PCP010880	8,106798463	7,952275317	8,001858526	8,523561956	PCP010880	XM_008340484
PCP010897	8,022367813	7,992485211	8,139551352	8,541754876	PCP010897	XM_008383599
PCP010905	8,716545126	8,632995197	8,593689902	8,591223118		
PCP010915	7,30679083	7,125878364	7,622051819	8,202956379	PCP010915	XM_009368117
PCP010920	7,992485211	8,463524373	8,503825738	9,52160044	PCP010920	XM_009359644
PCP010921	6,409390936	6,946379968	7,076815597	7,670089681	PCP010921	XM_009380101
PCP010924	7,533952624	8,014968933	7,785746699	7,470292816	PCP010924	XM_009352440
PCP010932	8,815927606	8,593689902	8,874458871	9,028817757	PCP010932	XM_009373100
PCP010959	7,912889336	8,033423002	7,876516947	7,536596918	PCP010959	XM_009365138
PCP010961	8,6794801	8,703903573	8,894817763	8,73470962	PCP010961	XM_009365143
PCP010973	8,43325159	8,311385042	8,515699838	9,130132926		
PCP011002	8,606109055	8,268331458	8,405141463	8,532667615	PCP011002	XM_009338795
PCP011016	6,965784285	6,922792504	7,17990909	7,63175911	PCP011016	XM_009378158
PCP011018	7,714245518	7,971543554	8,209453366	8,453970202	PCP011018	XM_009335707
PCP011038	7,658211483	7,743689989	7,942514505	7,375039431		
PCP011044	8,257387843	8,257387843	8,189824559	8,62935662	PCP011044	XM_008368995
PCP011071	7,395148508	6,798698597	7,209453366	7,251056285	PCP011071	XM_009364325
PCP011104	7,688809791	7,412188747	7,297649983	6,579994693	PCP011104	XM_009351445
PCP011156	8,062477937	8,265239967	8,224001674	8,199672345	PCP011156	NM_001328944
PCP011182	7,321928095	7,263691734	7,464913269	8,910882535	PCP011182	XR_529553
PCP011201	7,818070833	7,721099189	7,818070833	7,691185174	PCP011201	XM_009343143
PCP011241	8,291539098	7,820178962	7,807354922	7,518299077	PCP011241	XM_009370892
PCP011263	7,398059585	7,122362117	7,417852515	8,073231127	PCP011263	XM_009374633
PCP011271	7,732472726	7,464913269	7,478405614	7,46760555	PCP011271	XM_009376700
PCP011280	7,898873426	7,658211483	8,08571145	8,45532722	PCP011280	XM_009379265
PCP011284	7,826548487	7,813781191	7,928784831	7,714245518	PCP011284	XM_009365143
PCP011318	8,344295908	8,41079052	8,762647796	9,315896762	PCP011318	XM_017323165
PCP011322	7,691185174	8,176572111	8,35169016	8,464872438	PCP011322	XM_009370602
PCP011333	7,732472726	7,681800619	7,914863459	8,369248353		
PCP011338	8,380807357	8,369248353	8,698114274	9,061587209	PCP011338	XM_009370578
PCP011343	8,005624549	8,105018005	8,356099782	9,009352771		
PCP011356	8,560982378	8,592457037	8,655816908	9,017727086	PCP011356	XM_009370560
PCP011360	7,440204752	7,721099189	7,878541438	8,153146652	PCP011360	XM_008339088
PCP011366	8,188193194	8,112022407	7,898873426	8,297695831	PCP011366	XM_009370547
PCP011368	8,148069774	8,398016818	8,441574774	8,740354199	PCP011368	XM_009370545
PCP011381	7,88062431	8,347267018	7,528805707	7,688809791	PCP011381	XM_008363016
PCP011397	8,196380818	8,422064766	8,649866903	9,119797095	PCP011397	XM_009354771
PCP011408	6,853870927	6,890811455	7,101503009	7,489205728	PCP011408	XM_009344772
PCP011417	7,462134139	7,238404739	7,003714662	6,574858391		
PCP011423	8,458078458	8,619596417	8,70276142	8,594959026		
PCP011437	7,169925001	7,462134139	7,515699838	7,759355602		
PCP011440	7,843418611	8,151422517	7,698149009	8,053491515		
PCP011447	8,603626345	8,596189756	8,773699688	9,049848549	PCP011447	XM_009368175
PCP011448	7,080337882	7,303780748	7,336908182	7,809478757	PCP011448	XM_009380530
PCP011452	7,044394119	7,478405614	7,691185174	7,853808564		

PCP011454	7,339850003	7,400879436	7,660566438	8,142974967	PCP011454	XM_018646845
PCP011484	7,241554209	7,61227877	7,478405614	7,98111057		
PCP011485	7,636624621	7,592457037	7,375039431	7,238404739	PCP011485	XM_009355194
PCP011513	7,798763389	7,080337882	6,832890014	6,392317423	PCP011513	XM_009343512
PCP011516	7,303780748	7,333870928	7,707359132	8,071462363		
PCP011521	8,781359714	8,54303182	8,806291831	9,079484784	PCP011521	XM_008389102
PCP011528	8,376472723	8,513056419	9,006550496	8,54303182	PCP011528	XM_009351613
PCP011543	6,857980995	7,247927513	7,183188734	6,910852562	PCP011543	XM_008355082
PCP011558	7,132679654	6,95419631	7,169925001	7,727920455	PCP011558	XM_009380008
PCP011559	7,044394119	7,022367813	7,055282436	6,80309785	PCP011559	XM_009380007
PCP011563	7,515699838	7,412188747	7,315874125	7,069637728	PCP011563	XM_018652270
PCP011581	8,011227255	8,046196362	8,437419184	8,273002948	PCP011581	XM_009380037
PCP011583	8,345760055	8,314379315	8,517000043	8,862110391	PCP011583	XM_009380039
PCP011606	7,741466986	8,089212109	8,648645193	8,672425342	PCP011606	XM_018650169
PCP011619	8,320439548	8,354602022	8,615923847	8,813781191	PCP011619	XM_017327154
PCP011661	7,61227877	7,478405614	7,677155841	8,029728942	PCP011661	XR_665703
PCP011678	7,007531912	7,648681141	8,249492747	8,479780264	PCP011678	XM_009346850
PCP011683	6,662917555	6,375039431	6,624539604	10,1959873	PCP011683	XM_009357643
PCP011684	8,098032083	8,173277373	8,373604714	8,937609223	PCP011684	XM_009357426
PCP011715	8,027905997	7,843418611	7,916894583	7,609769734	PCP011715	XM_009350649
PCP011716	7,813781191	7,944448531	7,994353437	8,46760555	PCP011716	XM_009349720
PCP011725	6,902917719	6,599912842	6,874428132	7,254272787	PCP011725	XM_009361821
PCP011729	6,828707735	7,183188734	7,282347131	8,058911723	PCP011729	XM_009361815
PCP011734	7,431873638	7,260496187	6,902917719	6,459431619	PCP011734	XM_008395198
PCP011739	8,658211483	8,588714636	8,714245518	9,046223651	PCP011739	XM_008395206
PCP011745	7,510408147	7,864186145	8,238404739	8,631795481		
PCP011754	7,888743249	7,920829209	8,169925001	7,965784285	PCP011754	XM_009336099
PCP011759	7,189824559	6,768184325	7,166615031	6,965784285	PCP011759	XM_009336108
PCP011775	8,344295908	8,317412614	8,50779464	8,963849777	PCP011775	XM_018649333
PCP011777	7,594921715	7,709635275	7,743689989	8,201290788		
PCP011782	7,61227877	7,667679281	8,022367813	8,483815777	PCP011782	XM_009366744
PCP011798	8,564797319	8,753785022	8,727920455	9,265216522		
PCP011807	8,265239967	8,299208018	8,48651327	9,227206781		
PCP011812	8,207844058	8,051643995	8,163247124	8,60855054		
PCP011815	8,453970202	8,554588852	8,686500527	8,528766645	PCP011815	XM_008363424
PCP011817	8,903881846	8,768184325	8,568602197	8,872428639		
PCP011818	7,705079392	7,641473777	8,057071136	8,199672345	PCP011818	XM_008363430
PCP011825	8,442943496	8,120652609	9,100662339	8,018645301	PCP011825	XM_007209715
PCP011842	7,6794801	7,834976616	8,436003553	8,096293483	PCP011842	XM_009370079
PCP011848	8,013071384	8,299208018	8,440163216	8,409390936	PCP011848	NM_001294041
PCP011851	7,348728154	7,564759219	7,539158811	7,312882955	PCP011851	XM_008385573
PCP011855	7,691185174	7,159871337	7,282347131	7,06608919	PCP011855	XM_008385576
PCP011858	7,50517639	6,569855608	7,260496187	6,845490051	PCP011858	XM_008385581
PCP011867	8,087462841	7,944448531	7,624466495	7,599912842	PCP011867	XM_008351036
PCP011893	7,754887502	8,003770871	8,32494558	9,041659152	PCP011893	XM_009345083
PCP011905	7,273049587	7,520972191	7,437377568	8,053491515		

PCP011931	7,257387843	7	6,763809907	6,539158811		
PCP011935	8,395191361	8,44431092	8,612241904	8,342830273	PCP011935	XM_018650776
PCP011946	6,853870927	7,159871337	7,273049587	7,843418611		
PCP011950	8,24474438	8,174925683	8,108524457	8,082149041	PCP011950	XM_008386854
PCP011957	8,854868383	8,875503635	8,882643049	8,645045922	PCP011957	XM_009359954
PCP011967	7,855927425	7,681800619	8,112022407	8,436003553		
PCP011976	8,477070141	8,581200582	8,723387907	9,094526396		
PCP011984	8,033423002	7,986809101	8,173277373	8,612241904		
PCP011985	7,653418353	7,714245518	8,442943496	8,447083226	PCP011985	XM_008354764
PCP011994	7,339850003	7,437377568	7,417852515	8,06608919	PCP011994	XM_008363562
PCP012009	7,579919292	8,186510462	8,431831861	8,827596761		
PCP012025	8,509101985	8,50779464	8,554588852	8,942514505	PCP012025	XM_009373764
PCP012034	7,451211112	7,383704292	7,456724026	8,001858526		
PCP012049	6,969587981	6,54951516	7,018700931	7,321928095	PCP012049	XM_009369197
PCP012054	8,62935662	8,703903573	8,72904287	8,628153873	PCP012054	XM_008393279
PCP012055	7,851749041	7,011227255	7,473056289	7,146390476	PCP012055	XM_009369310
PCP012098	8,791162889	8,531381461	8,692336525	8,713111526	PCP012098	XM_008380159
PCP012114	6,491853096	7,115511897	7,333870928	8,144658243	PCP012114	XM_008382154
PCP012139	7,695785075	7,930737338	8,191454081	8,735826768	PCP012139	XM_008355189
PCP012143	7,380850638	7,569855608	7,247927513	6,714245518	PCP012143	XM_008343276
PCP012153	8,623259662	8,536558066	8,604849706	8,427648072	PCP012153	XM_009371307
PCP012163	6,837060204	6,015024705	9,274564521	8,257387843		
PCP012164	8,115459877	8,191454081	8,380807357	8,695819867	PCP012164	XM_008341444
PCP012167	6,910852562	7,076815597	7,257387843	8,471675214	PCP012167	XM_008353052
PCP012173	8,273002948	7,888743249	7,811663685	7,215969746	PCP012173	XR_001953538
PCP012179	8,767091963	8,815927606	8,851749041	8,517000043		
PCP012200	7,282347131	7,146390476	7,453929061	8,503825738	PCP012200	XM_008381224
PCP012203	7,254272787	6,969587981	7,348728154	7,61227877		
PCP012204	7,244791942	6,977279923	6,798698597	6,68187088		
PCP012218	8,759322309	8,894817763	8,978224236	8,625708843	PCP012218	XM_008340437
PCP012220	7,651051691	8,092757141	7,992485211	8,022367813	PCP012220	XM_008221392
PCP012228	8,315919398	8,487840034	8,41079052	8,347267018		
PCP012246	8,181549844	8,158205728	8,214319121	7,914863459	PCP012246	XM_008375646
PCP012264	8,395191361	8,482485305	8,583722152	8,856954575	PCP012264	XM_008370756
PCP012266	7,862141394	8,154818109	8,233619677	8,42488011	PCP012266	XM_009351129
PCP012281	8,154818109	8,341318667	8,466260038	8,732438807	PCP012281	XM_008378806
PCP012288	7,946438456	7,686500527	7,878541438	8,403736386	PCP012288	XM_009340280
PCP012291	7,796558821	7,369204723	7,377904593	7,614709844	PCP012291	XM_009361637
PCP012295	8,168270966	7,969645536	8,45532722	8,296136161	PCP012295	XM_008395566
PCP012309	7,667679281	7,732472726	7,92677039	8,282300792	PCP012309	XM_009371563
PCP012334	7,029784146	7,494495616	7,653418353	8,087462841	PCP012334	XM_008342024
PCP012340	8,576181982	8,390899794	8,366322214	8,176572111	PCP012340	XM_008378102
PCP012356	8,396604781	8,396604781	8,317412614	8,342830273	PCP012356	XM_009343894
PCP012365	5,459431619	6,718772592	7,199672345	9,083931345	PCP012365	XM_009348862
PCP012374	7,914863459	7,303780748	7,429030064	6,984931073		
PCP012392	7,956114749	8,427648072	8,236827423	8,493174961	PCP012392	XM_018643059

PCP012403	8,087462841	7,996219247	7,653418353	7,294620749	PCP012403	XM_009345505
PCP012406	7,6794801	7,489205728	7,602364826	7,303780748	PCP012406	XM_009347253
PCP012414	8,760453835	8,445718428	8,359002767	7,930737338		
PCP012418	7,06608919	7,215969746	7,940577883	8,027905997		
PCP012439	8,41502341	8,096293483	7,95419631	7,641473777	PCP012439	XM_009377772
PCP012445	8,318904285	8,311385042	8,029728942	8,363390129		
PCP012473	8,363390129	8,554588852	8,54689446	9,20537785	PCP012473	XM_009345077
PCP012475	8,342830273	8,227230852	8,406545173	8,049848549		
PCP012505	7,990557838	8,339850003	9,052568051	9,348728154		
PCP012506	8,416459769	8,151422517	8,120652609	8,078577814	PCP012506	XM_008363326
PCP012507	7,725673	7,960001932	7,888743249	7,448488033		
PCP012530	8,285402219	8,236827423	8,545620932	8,723387907		
PCP012537	7,607330314	7,639015048	7,691185174	8,144658243	PCP012537	XM_008366557
PCP012551	7,375039431	7,783522135	8,207844058	8,586201784	PCP012551	XM_009370292
PCP012564	7,624466495	7,569855608	7,552054236	7,456724026		
PCP012568	8,554588852	8,509101985	8,581200582	8,942514505		
PCP012575	7,855927425	7,473056289	7,372168569	7,235248379	PCP012575	XM_009365133
PCP012585	7,153095972	7,142923928	7,273049587	7,946438456	PCP012585	XM_009366792
PCP012600	8,807354922	8,552015799	8,97441459	8,979196518	PCP012600	XM_018645380
PCP012644	7,348728154	7,462134139	7,386552314	7	PCP012644	XM_009381084
PCP012653	7,494495616	7,069637728	7,46760555	7,260496187	PCP012653	XM_018646410
PCP012682	7,499845887	7,520972191	7,62935662	8,026080745	PCP012682	XM_009379863
PCP012688	7,961912672	8,113742166	8,092757141	7,71651083	PCP012688	XM_009369707
PCP012731	7,942514505	8,046196362	7,894817763	8,372125027	PCP012731	XM_018650540
PCP012824	8,299208018	8,405141463	8,283875484	8,120652609	PCP012824	XM_008389083
PCP012846	8,908902953	8,752648252	8,699294818	8,743723645	PCP012846	XM_008370412
PCP012905	8,370687407	8,17990909	8,338379842	8,103287808	PCP012905	XM_009336424
PCP012906	7,470292816	7,459431619	7,624466495	7,257387843		
PCP012927	8,652235507	8,344295908	8,471675214	8,431831861	PCP012927	XM_009338883
PCP012937	8,622051819	8,434628228	8,053491515	7,876516947	PCP012937	XM_009338868
PCP012954	8,022367813	8,115459877	7,847558521	7,392317423	PCP012954	XM_008381874
PCP012959	6,60481265	6,922792504	7,206232954	7,54689446	PCP012959	XM_008381898
PCP012967	7,990557838	7,794415866	8,022367813	8,320439548	PCP012967	XM_009352058
PCP012973	7,574934058	7,71651083	7,723353774	7,602364826		
PCP012974	7,475733431	7,392317423	7,684187561	8,174925683		
PCP013003	7,554588852	7,711976642	7,977279923	8,212715235	PCP013003	XM_009365219
PCP013034	8,341318667	8,342830273	8,566054038	9,029756544		
PCP013067	8,386595423	7,936637939	7,950293432	7,50517639	PCP013067	XM_008342088
PCP013072	8,096293483	8,296136161	7,743689989	7,235248379		
PCP013073	7,125878364	6,857980995	7,037052702	6,81159947	PCP013073	XM_017323521
PCP013083	8,637820329	8,890781061	8,948367232	8,744833837	PCP013083	XM_008341593
PCP013087	8,092757141	8,268331458	8,41079052	9,167418146	PCP013087	XM_017323783
PCP013088	8,347267018	8,553322101	8,412231097	8,409390936	PCP013088	XM_008341588
PCP013089	7,963878669	8,262094845	8,163247124	8,136119594	PCP013089	XM_008354734
PCP013091	7,928784831	7,826548487	7,736943052	7,796558821	PCP013091	XM_008354736
PCP013092	7	7,011227255	7,260496187	7,946438456		

PCP013124	7,832890014	7,940577883	8,064311643	8,474395481	PCP013124	XM_008393284
PCP013125	7,380850638	7,266786541	7,785746699	8,341318667	PCP013125	XM_009354338
PCP013128	7,206232954	7,655852677	7,975389442	8,413627929	PCP013128	XM_018646661
PCP013135	6,942514505	6,832890014	7,392317423	8,080391184	PCP013135	XM_009354364
PCP013136	6,845490051	6,691115365	7,30679083	8,224001674	PCP013136	XM_009354367
PCP013138	7,787902559	7,977279923	8,141238626	8,445718428	PCP013138	XM_009354371
PCP013140	6,906890596	6,658211483	6,984931073	7,3458044	PCP013140	XM_009354376
PCP013152	8,363390129	8,442943496	8,364878797	8,031604721		
PCP013158	7,918863237	8,071462363	8,361943774	7,783522135		
PCP013165	8,640244936	8,431831861	8,752648252	9,045295522		
PCP013173	8,788979283	8,631795481	8,762647796	9,087462841		
PCP013178	7,434628228	7,609769734	7,497213158	7,434628228	PCP013178	XM_018650789
PCP013181	6,667750232	6,754887502	6,853870927	7,309885571	PCP013181	XM_017330149
PCP013182	8,101555535	8,191454081	8,271463028	8,62450305	PCP013182	XM_009370935
PCP013184	6,845490051	7,193081765	7,315874125	7,688809791	PCP013184	XM_009370934
PCP013187	8,117227504	8,30833903	8,373604714	8,653454181		
PCP013189	7,646234675	7,973439079	7,942514505	7,855927425		
PCP013208	7,156538193	7,30679083	7,312882955	7,153095972	PCP013208	XM_009370894
PCP013214	7,395148508	7,282347131	7,403693786	8,098032083	PCP013214	XM_009370882
PCP013215	7,539158811	7,807354922	7,87036472	8,212715235	PCP013215	XM_009350501
PCP013218	7,996219247	8,153146652	8,422064766	9,670656249	PCP013218	XM_009340068
PCP013228	7,156538193	7,294620749	7,324900589	7,026025399		
PCP013236	7,71651083	7,686500527	7,860031646	8,463524373	PCP013236	XM_008379392
PCP013240	7,98111057	8,098032083	8,101555535	8,46760555	PCP013240	XM_009340003
PCP013262	7,483815777	7,813781191	8,087462841	8,612241904	PCP013262	XM_009365947
PCP013265	7,851749041	7,837123296	7,845490051	8,395191361	PCP013265	XM_009365996
PCP013273	7,375039431	7,380850638	7,30679083	7,115511897	PCP013273	XM_009366117
PCP013288	6,878602742	6,772545519	6,798698597	7,417852515	PCP013288	XM_009366379
PCP013296	8,321928095	7,77478706	8,206184105	7,860031646	PCP013296	XM_009346990
PCP013298	7,003714662	6,837060204	7,047996356	7,641473777	PCP013298	XM_009346998
PCP013309	7,222360218	7,129283017	7,196430001	7,866228001	PCP013309	XM_018645903
PCP013314	7,641473777	7,62935662	7,54689446	8,212715235		
PCP013318	8,43325159	8,445718428	8,764871591	9,210257347	PCP013318	XM_009339849
PCP013319	8,018645301	7,98111057	8,239980333	8,517000043	PCP013319	XM_008393227
PCP013359	8,658211483	8,772578508	8,687655621	9,053464362	PCP013359	XM_009376244
PCP013362	6,906890596	6,528727582	6,894817763	7,330916878	PCP013362	XM_009376254
PCP013366	7,924812504	7,992485211	8,049848549	7,820178962		
PCP013373	7,276124405	6,169925001	6,87036472	7,129283017		
PCP013377	8,537878433	8,380807357	8,294620749	8,372125027	PCP013377	XM_009367891
PCP013378	7,92677039	7,518299077	7,956114749	7,714245518	PCP013378	XM_009367887
PCP013382	7,841281284	7,648681141	7,602364826	7,569855608		
PCP013388	8,434628228	8,296136161	8,198052084	8,158205728		
PCP013394	6,99242856	6,523561956	7,263691734	6,950351762	PCP013394	XM_009343531
PCP013398	8,051643995	7,798763389	7,794415866	7,834976616		
PCP013401	6,254178286	6,874428132	7,718841075	7,946438456	PCP013401	XM_009343521
PCP013404	8,356099782	8,118941073	8,419243918	8,636624621		

PCP013418	7	6,942514505	7,225641265	6,853870927		
PCP013434	8,592457037	8,297695831	8,169925001	7,847558521	PCP013434	XM_018643903
PCP013437	7,206232954	6,81159947	6,996275749	7,557119022	PCP013437	XM_017334839
PCP013489	7,554588852	7,796558821	7,752681699	7,263691734		
PCP013494	3,663344619	5,727920455	7,597456684	13,73064739	PCP013494	NM_001302321
PCP013501	7,199672345	7,209453366	7,069637728	6,87036472	PCP013501	XM_009359249
PCP013509	7,988684687	7,832890014	8,016808288	8,360495967	PCP013509	XM_009359265
PCP013549	8,530094158	8,48651327	8,636624621	8,420676082	PCP013549	XM_009348936
PCP013550	7,497213158	7,691185174	7,559721231	6,874428132	PCP013550	XM_008340574
PCP013564	7,247927513	7,209453366	7,375039431	8,04072808	PCP013564	XM_009376727
PCP013588	6,845490051	6,910852562	7,094552786	8,046196362		
PCP013589	8,239980333	8,04072808	7,967687386	7,684187561	PCP013589	XM_009350024
PCP013590	7,772611496	8,156487632	8,689997971	9,13442632		
PCP013598	8,120652609	7,890750668	8,020535537	7,864186145	PCP013598	XM_018642525
PCP013610	7,315874125	7,260496187	7,533952624	7,818070833	PCP013610	XM_009369631
PCP013619	8,144658243	8,400879436	8,510447709	8,899870461	PCP013619	XM_009369618
PCP013625	8,348728154	8,181549844	8,168270966	7,960001932	PCP013625	XM_009355348
PCP013632	7,904905525	8,014968933	8,288496992	8,110248388	PCP013632	XM_009355355
PCP013642	8,62935662	8,863164132	8,732438807	8,335390355	PCP013642	XM_009377978
PCP013644	8,405141463	8,72904287	8,793343194	8,97441459		
PCP013648	7,783522135	8,348728154	8,315919398	8,222408523	PCP013648	XM_009356330
PCP013652	8,022367813	7,660566438	8,096293483	8,677120596	PCP013652	XM_009356267
PCP013653	8,376472723	8,306836389	8,317412614	8,277659361	PCP013653	XM_009356256
PCP013666	8,43325159	8,300718622	8,350187812	8,857980995	PCP013666	XM_021832237
PCP013675	8,306836389	8,930737338	8,470333494	8,787902559	PCP013675	XM_009348772
PCP013684	7,076815597	6,934634441	6,902917719	6,81159947	PCP013684	XM_018651846
PCP013696	8,132731056	8,024197765	8,103287808	7,597456684		
PCP013702	7,882643049	7,71651083	7,845490051	8,588714636	PCP013702	XM_018650281
PCP013757	9,200481794	8,943481842	8,572397068	8,32791187	PCP013757	XM_009352072
PCP013764	8,640244936	8,754887502	8,68183575	8,557157324	PCP013764	XM_009366541
PCP013770	7,22881869	7,691185174	8,009380767	8,786825032	PCP013770	XM_009361280
PCP013771	8,08571145	8,21106088	8,101555535	8,156487632	PCP013771	XM_009361284
PCP013796	7,14974712	7,129283017	7,3458044	7,805227956	PCP013796	XM_017324484
PCP013799	6,906890596	6,906890596	6,898812977	7,646234675	PCP013799	XM_008377320
PCP013808	7,820178962	7,912889336	8,148069774	8,964831792	PCP013808	XM_018647648
PCP013812	9,211888295	8,429071922	8,429071922	7,818070833	PCP013812	XM_009364327
PCP013835	6,662917555	7,073284692	6,988684687	7,398059585	PCP013835	XM_009337800
PCP013841	7,515699838	7,327956767	7,215969746	6,058965822	PCP013841	XM_009337790
PCP013846	7,948367232	8,32791187	7,741466986	6,946379968	PCP013846	XM_009345512
PCP013882	7,754887502	7,752681699	8,038918989	8,474395481	PCP013882	XM_009341584
PCP013899	8,776992236	8,833933692	8,865207434	8,824417827	PCP013899	XM_009375409
PCP013900	8,326429487	8,069691427	8,249492747	8,092757141	PCP013900	XM_008340688
PCP013918	7,464913269	7,475733431	7,520972191	7,294620749	PCP013918	XM_009354498
PCP013921	7,105070402	6,772545519	7,579919292	6,403778984	PCP013921	NM_001328880
PCP013929	7,018700931	7,276124405	7,574934058	7,494495616		
PCP013935	6,700439718	7,14974712	7,448488033	8,451211112	PCP013935	XM_009378738

PCP013936	7,499845887	7,617136829	7,727920455	8,430452552	PCP013936	XM_009378555
PCP013964	8,395191361	8,320439548	8,130982335	8,078577814	PCP013964	XM_008341951
PCP013977	8,909893084	8,831845581	8,780277286	8,459431619	PCP013977	XM_017331339
PCP013980	6,996275749	7,330916878	7,166615031	6,727920455	PCP013980	XM_009347466
PCP013995	7,462134139	7,475733431	7,231989133	6,798698597	PCP013995	XM_009374408
PCP014011	7,247927513	7,398059585	7,785746699	8,596189756	PCP014011	XM_009361912
PCP014015	7,636624621	7,622051819	7,651051691	8,176572111	PCP014015	XM_009361885
PCP014017	7,969645536	8,064311643	8,184875343	7,904905525	PCP014017	XM_018643146
PCP014042	8,106798463	8,28692734	8,688844752	8,930737338	PCP014042	XM_009381361
PCP014088	7,977279923	8,338379842	8,637820329	8,865207434	PCP014088	XM_008384177
PCP014098	6,339850003	6,584962501	6,922792504	7,721099189	PCP014098	XM_008374306
PCP014106	8,026080745	7,904905525	7,986809101	8,456682963	PCP014106	XM_008379660
PCP014112	8,940548521	8,765965634	8,84862294	8,37794796	PCP014112	XM_009348591
PCP014120	7,960001932	8,038918989	8,214319121	8,520932916	PCP014120	XM_017323318
PCP014129	7,047996356	7,076815597	7,206232954	7,811663685	PCP014129	XR_001953275
PCP014145	7,426264755	7,473056289	8,080391184	8,511752654	PCP014145	XM_009371364
PCP014151	8,132731056	8,106798463	8,199672345	7,924812504		
PCP014157	8,341318667	8,420676082	8,477070141	8,832890014	PCP014157	XM_008371456
PCP014171	7,74819285	8,178266468	8,509101985	8,84862294	PCP014171	XM_009381068
PCP014182	7,434628228	7,169925001	7,247927513	7,055282436		
PCP014259	8,106798463	8,224001674	8,199672345	8,588714636	PCP014259	XM_008379346
PCP014264	6,853870927	7,076815597	7,21916852	7,475733431		
PCP014270	6,648609245	7,279192684	7,761551232	8,566054038	PCP014270	XM_009353911
PCP014299	7,936637939	7,768184325	7,62935662	7,475733431		
PCP014314	8,857980995	8,54303182	8,603626345	8,312882955		
PCP014356	8,348728154	8,257387843	8,625708843	9,415044544		
PCP014364	8,09976859	8,022367813	7,894817763	7,888743249	PCP014364	XM_018651584
PCP014412	6,491853096	6,890811455	6,965784285	7,199672345		
PCP014426	8,478446064	8,222408523	8,392317423	7,886733033	PCP014426	XM_009347952
PCP014515	6,448405435	7,244791942	7,539158811	7,866228001	PCP014515	XM_018645477
PCP014517	6,918863237	7,417852515	7,166615031	8,24474438	PCP014517	XM_009349335
PCP014530	7,996219247	7,577428828	7,577428828	8,169925001	PCP014530	XM_008356436
PCP014542	6,906890596	7,018700931	7,28845085	7,876516947	PCP014542	XM_008339807
PCP014545	8,159871337	8,332394659	7,878541438	7,266786541		
PCP014553	7,42063398	7,339850003	7,324900589	7,173227395	PCP014553	XM_009340385
PCP014557	6,946379968	6,80309785	6,942514505	7,437377568	PCP014557	XM_009379939
PCP014572	6,832890014	6,653490009	7,029784146	7,523561956	PCP014572	XM_009376029
PCP014581	8,118941073	8,018645301	8,076815597	7,975389442		
PCP014584	7,855927425	7,754887502	7,845490051	7,372168569	PCP014584	XM_009376060
PCP014587	8,459431619	8,786825032	8,739206792	8,456682963	PCP014587	XM_018651412
PCP014608	7,614709844	7,076815597	7,448488033	7,321928095		
PCP014611	7,321928095	7,014913158	6,938638658	6,741466986	PCP014611	XM_008349405
PCP014629	8,646270682	8,276124405	8,528766645	8,445718428		
PCP014633	7,406587689	7,398059585	7,453929061	8,176572111		
PCP014653	7,442943496	7,069637728	7,08395793	6,853870927	PCP014653	XM_018646015
PCP014658	8,477070141	8,360495967	8,409390936	8,118941073	PCP014658	XM_017322467

PCP014662	7,324900589	7,513095909	7,958088658	8,106798463	PCP014662	XM_008393969
PCP014691	7,718841075	7,900866808	7,990557838	8,615923847		
PCP014694	7,115511897	7,395148508	7,670089681	8,092757141	PCP014694	XM_009353659
PCP014701	8,241601875	8,437419184	8,375039431	8,788979283	PCP014701	XM_018646501
PCP014713	7,312882955	7,339850003	7,681800619	8,166564822	PCP014713	XM_009353696
PCP014731	6,965784285	7,196430001	7,50779464	7,098032083		
PCP014733	8,183238369	7,996219247	8,220765252	8,80841723		
PCP014739	7,609769734	7,415065677	7,688809791	7,988684687	PCP014739	XM_009366279
PCP014750	8,153146652	8,005624549	8,115459877	8,506486109		
PCP014759	8,589950702	8,589950702	8,657032562	8,490530019	PCP014759	XM_009355020
PCP014779	8,396604781	8,523561956	8,813781191	8,266786541	PCP014779	XM_009355002
PCP014788	8,487840034	8,597419437	8,626913629	9,194756854		
PCP014796	6,81159947	6,97349648	7,269874722	7,442943496		
PCP014799	8,552015799	8,517000043	8,711942238	8,527477006	PCP014799	XM_009341992
PCP014813	7,665335917	7,186559982	7,670089681	7,202907418	PCP014813	XM_009342020
PCP014814	8,241601875	8,477070141	8,607330314	9,06250492	PCP014814	XM_009342024
PCP014818	7,222360218	7,225641265	7,173227395	8,654636029	PCP014818	XM_009342027
PCP014838	7,898873426	8,069691427	7,898873426	7,564759219	PCP014838	XM_009365803
PCP014867	6,87036472	6,934634441	7,163196797	7,6794801	PCP014867	XM_008374324
PCP014872	8,249492747	8,577428828	8,427648072	8,064311643	PCP014872	XM_018650826
PCP014897	8,485145023	8,409390936	8,672425342	9,161560218	PCP014897	XM_009373459
PCP014905	7,730164413	7,857980995	7,986809101	8,438791853	PCP014905	XM_009373478
PCP014920	8,075051225	7,757089938	7,732472726	8,035238993	PCP014920	XM_008393487
PCP014925	7,868328714	7,785746699	8,016808288	8,348728154	PCP014925	XM_008392965
PCP014940	7,166615031	6,60481265	7	6,794415866		
PCP014944	7,765998774	8,048050866	8,550746785	9,243173983		
PCP014950	7,932687205	8,04072808	8,279239123	8,427648072	PCP014950	XM_009355433
PCP014963	8	8,744833837	8,735826768	8,611024797	PCP014963	XM_017325447
PCP014980	8,279239123	8,396604781	8,188193194	7,868328714	PCP014980	XM_009377685
PCP014982	6,714245518	6,64385619	6,938638658	7,42063398		
PCP015001	7,952275317	8,13442632	8,202956379	8,463524373		
PCP015019	7,417852515	7,169925001	7,619633096	7,878541438	PCP015019	XM_009340331
PCP015047	7,624466495	7,360452072	7,336908182	7	PCP015047	XM_009363852
PCP015048	7,494495616	7,354646096	7,536596918	8,037107627	PCP015048	XM_009363853
PCP015053	8,530094158	8,640244936	8,830768706	8,996247498		
PCP015080	5,781359714	6,815959618	7,392317423	10,44570808	PCP015080	XR_668418
PCP015090	7,417852515	7,481153605	7,660566438	8,263644792	PCP015090	XM_009379315
PCP015094	8,136119594	8,141238626	8,372125027	8,198052084	PCP015094	XM_009379304
PCP015102	8,001858526	7,83080039	7,832890014	7,874489611	PCP015102	XM_009377283
PCP015124	7,528805707	7,702726796	7,743689989	8,338379842	PCP015124	XM_018645785
PCP015128	7,380850638	7,062531903	7,434628228	7,757089938	PCP015128	XM_009337404
PCP015141	8,044394119	8,120652609	8,130982335	7,491853096	PCP015141	XM_009379476
PCP015171	8,323415108	8,271463028	8,344295908	8,168270966	PCP015171	XM_009347108
PCP015172	8,77807713	8,961941603	9,021451966	8,243173983		
PCP015206	8,024197765	7,617136829	7,886733033	7,818070833	PCP015206	XM_009367006
PCP015207	7,196430001	7,14974712	7,470292816	8,136119594		

PCP015209	8,154818109	8,110248388	8,117227504	7,95419631		
PCP015232	7,398059585	7,040782866	7,209453366	7,101503009		
PCP015261	7,440204752	7,260496187	7,266786541	7,209453366	PCP015261	XM_008366226
PCP015270	7,448488033	6,87036472	7,741466986	7,967687386	PCP015270	XM_009365879
PCP015282	7,579919292	7,582480735	7,912889336	8,441574774		
PCP015290	7,709635275	7,822284016	7,874489611	8,241601875	PCP015290	XM_018646415
PCP015296	7,080337882	6,768184325	6,977279923	7,655852677	PCP015296	XM_009349932
PCP015297	8,282300792	8,509101985	8,513056419	8,941546519	PCP015297	XM_008393897
PCP015312	7,904905525	8,013071384	7,851749041	7,888743249	PCP015312	NM_001294001
PCP015322	7,011227255	7,315874125	7,222360218	6,143026004		
PCP015326	7,569855608	7,604886762	7,63175911	8,13442632	PCP015326	XM_018649884
PCP015329	7,215969746	7,206232954	6,64385619	6,619559738	PCP015329	XM_009369529
PCP015352	7,279192684	7,526147081	7,497213158	8,263644792	PCP015352	XM_008341121
PCP015404	7,327956767	7,206232954	7,372168569	7,761551232		
PCP015412	6,958030641	6,464831606	7,462134139	7,677155841		
PCP015418	7,994353437	7,750405521	7,837123296	7,672425342		
PCP015435	6,732404887	7,257387843	7,515699838	8,003770871	PCP015435	XM_018648522
PCP015439	8,285402219	8,274587815	8,449850215	8,73470962	PCP015439	XM_009363102
PCP015441	8,055282436	8,235200503	8,514398461	8,968666793		
PCP015446	8,113742166	7,924812504	8,08571145	8,576181982	PCP015446	XM_009373493
PCP015457	7,878541438	7,333870928	7,906890596	6,497133304	PCP015457	XM_008386040
PCP015460	7,018700931	7,324900589	7,890750668	8,513056419	PCP015460	NM_001328737
PCP015466	8,557157324	8,375039431	8,519636253	8,124121312	PCP015466	XM_008371987
PCP015488	8,21916852	8,141238626	8,132731056	7,839203788	PCP015488	XM_018643155
PCP015492	7,105070402	7,105070402	7,241554209	6,658211483	PCP015492	XM_009339090
PCP015523	7,62935662	7,853808564	7,984874125	8,257387843		
PCP015524	7,809478757	7,619633096	7,686500527	7,651051691	PCP015524	XM_008394195
PCP015527	8,188193194	8,388017285	8,475733431	8,727920455		
PCP015540	7,988684687	8,020535537	8,159871337	8,677120596	PCP015540	XM_009344444
PCP015551	7,50517639	8,146339557	7,662988741	6,80309785	PCP015551	XM_017327045
PCP015554	6,691115365	6,539158811	7,022367813	7,372168569	PCP015554	XM_008370342
PCP015560	7,459431619	7,549438149	7,98111057	8,024197765	PCP015560	XM_009355762
PCP015570	8,364878797	8,288496992	8,572397068	8,791162889		
PCP015574	7,101503009	7,380850638	7,251056285	7,674757228	PCP015574	XM_008352642
PCP015585	7,872397856	7,619633096	7,743689989	7,698149009		
PCP015586	7,531381461	7,339850003	7,383704292	7,276124405		
PCP015595	6,837060204	7,118941073	7,17990909	7,634230226	PCP015595	XM_009365118
PCP015604	8,407989993	8,475733431	8,768184325	9,312134193	PCP015604	XM_009380907
PCP015633	7,624466495	7,820178962	7,003714662	5,841218374	PCP015633	XM_009375134
PCP015634	7,17990909	6,807354922	6,882643049	7,448488033	PCP015634	XM_009375132
PCP015635	7,279192684	7,06608919	7,395148508	7,702726796	PCP015635	XM_009375129
PCP015644	7,930737338	7,948367232	7,711976642	7,607330314		
PCP015648	6,898812977	7,222360218	7,215969746	7,651051691	PCP015648	XM_009336018
PCP015686	8,204571144	8,345760055	8,466260038	8,890781061		
PCP015720	8,482485305	8,5824431	8,62935662	9,130132926		
PCP015729	8,098032083	8,571107931	9,000929563	9,295378654		

PCP015756	7,61227877	7,634230226	7,607330314	8,19313106	PCP015756	XM_021951602
PCP015759	7,007531912	6,857980995	7,007531912	6,777025123	PCP015759	XM_009344640
PCP015783	7,54689446	7,206232954	7,166615031	6,420718183		
PCP015785	7,587440004	7,61227877	7,961912672	8,263644792	PCP015785	XM_008393418
PCP015790	8,163247124	8,101555535	8,363390129	8,108524457	PCP015790	XM_008375273
PCP015802	8,158205728	8,254225537	8,350187812	8,718806834	PCP015802	XM_009374124
PCP015812	7,163196797	7,166615031	7,594921715	7,730164413	PCP015812	XM_008366530
PCP015829	8,335390355	8,290018847	8,494535617	8,826548487	PCP015829	XM_009381060
PCP015839	6,686500527	6,86628983	6,95419631	7,528805707	PCP015839	XM_009357960
PCP015846	7,330916878	7,655852677	7,878541438	8,723387907	PCP015846	XM_018652354
PCP015848	7,853808564	8,051643995	8,186510462	8,768184325		
PCP015856	8,146339557	8,255831156	7,996219247	7,073284692		
PCP015860	7,707359132	7,587440004	7,366322214	7,360452072		
PCP015868	7,956114749	7,757089938	7,759355602	7,783522135	PCP015868	XM_021968888
PCP015876	7,670089681	8,154818109	7,853808564	8	PCP015876	XM_009345547
PCP015878	7,572359168	7,564759219	7,818070833	7,544346278	PCP015878	XM_018643030
PCP015927	7,510408147	7,499845887	7,62935662	8,137862104	PCP015927	XM_008378850
PCP015944	7,254272787	7,440204752	7,389480771	6,996275749	PCP015944	XM_018650320
PCP015952	8,873444113	8,520932916	8,732438807	8,597419437	PCP015952	XM_009373718
PCP016030	7,146390476	7,108524457	6,938638658	6,700439718	PCP016030	XM_018651423
PCP016063	6,930737338	7,222360218	7,122362117	7,837123296	PCP016063	XM_009341766
PCP016065	7,276124405	7,105070402	6,820178962	6,339850003	PCP016065	XM_009349739
PCP016141	7,677155841	8,183238369	7,811663685	8,106798463	PCP016141	XM_009363863
PCP016147	8,481113232	8,209453366	8,326429487	8,347267018	PCP016147	XM_009363930
PCP016155	7,77036657	7,828644274	8,191454081	7,967687386	PCP016155	XM_009364065
PCP016160	6,984931073	7,08395793	7,28845085	7,634230226	PCP016160	XM_009364123
PCP016167	7,451211112	7,617136829	7,634230226	7,366322214	PCP016167	XM_009364281
PCP016179	7,324900589	7,189824559	7,044394119	7,118941073	PCP016179	XM_009364488
PCP016180	8,948367232	8,724513853	8,302273349	6,926829678	PCP016180	XM_009364497
PCP016184	7,429030064	7,069637728	7,562242424	7,044394119	PCP016184	XM_018648807
PCP016185	8,715378619	8,189824559	8,564797319	8,909893084	PCP016185	XM_009364554
PCP016199	7,924812504	8,366322214	8,260543232	8,422064766	PCP016199	XR_667973
PCP016205	7,998139076	7,890750668	7,878541438	8,303780748	PCP016205	XM_009364774
PCP016211	7,750405521	7,572359168	7,670089681	7,541716163	PCP016211	XM_009364917
PCP016227	8,117227504	8,279239123	8,35169016	8,761551232		
PCP016260	7,77036657	7,557119022	7,641473777	7,279192684	PCP016260	XM_009345037
PCP016271	7,483815777	7,456724026	7,412188747	7,862141394	PCP016271	XM_009373296
PCP016278	8,481113232	8,559682997	7,88062431	7,526147081	PCP016278	XM_009373316
PCP016292	6,714245518	6,686500527	6,996275749	7,725673	PCP016292	XM_009373330
PCP016298	8,405141463	8,189824559	8,321928095	8,186510462		
PCP016322	7,619633096	7,843418611	7,998139076	8,312882955		
PCP016343	6,922792504	6,700439718	7,557119022	7,54689446		
PCP016345	7,132679654	7,276124405	7	6,272956308	PCP016345	XM_009376395
PCP016360	8,238404739	8,427648072	8,151422517	7,948367232	PCP016360	XM_009349772
PCP016361	8,548166865	8,532667615	8,483815777	8,483815777	PCP016361	XM_009363451
PCP016364	8,04072808	7,918863237	8,318904285	8,576181982		

PCP016368	8,341318667	7,969645536	8,101555535	8,003770871	PCP016368	XM_009363269
PCP016394	8,21106088	8,255831156	8,449850215	7,6794801	PCP016394	XM_009362806
PCP016401	7,641473777	7,377904593	7,094552786	6,828707735	PCP016401	XM_009362722
PCP016407	7,868328714	7,805227956	8,124121312	8,350187812		
PCP016419	7,026025399	7,30679083	7,437377568	7,77036657	PCP016419	XM_009341516
PCP016458	7,526147081	7,478405614	7,815895594	8,018645301	PCP016458	XM_009375684
PCP016462	7,499845887	7,44567705	7,297649983	6,691115365		
PCP016464	8,089212109	7,796558821	7,914863459	8,309840108		
PCP016479	6,686500527	6,691115365	6,768184325	7,309885571	PCP016479	XM_009368757
PCP016506	8,011227255	7,963878669	7,876516947	8,379378367	PCP016506	XM_008352603
PCP016523	8,279239123	8,43325159	8,567309664	8,780277286	PCP016523	XM_008352607
PCP016525	8,293103743	7,961912672	7,994353437	7,988684687	PCP016525	XM_009370371
PCP016533	7,811663685	7,754887502	7,752681699	7,330916878	PCP016533	XM_009368716
PCP016534	7,392317423	7,745976779	7,781359714	8,288496992	PCP016534	XM_008340571
PCP016567	8,853839746	8,742578916	8,788979283	8,515699838		
PCP016570	8,779161208	8,642665475	8,639051236	9,050746552	PCP016570	XM_009359352
PCP016578	6,777025123	6,84962403	6,781359714	7,309885571	PCP016578	XM_008386101
PCP016592	8,646270682	8,709669735	8,639051236	8,682994584	PCP016592	XM_009352255
PCP016595	8,21757002	8,862110391	8,718806834	8,944477815	PCP016595	XM_009352228
PCP016596	7,040782866	7,665335917	7,309885571	7,309885571	PCP016596	XM_008364022
PCP016606	8,151422517	8,003770871	8,318904285	8,075051225	PCP016606	XM_018649117
PCP016610	7,648681141	7,725673	7,686500527	7,333870928	PCP016610	XM_009365846
PCP016615	8,288496992	8,354602022	8,474395481	8,916864735	PCP016615	XM_009365842
PCP016627	8,266786541	8,708497652	8,055282436	6,874428132	PCP016627	XM_009346530
PCP016632	8,174925683	8,434628228	8,626913629	9,344295908	PCP016632	XM_009346534
PCP016633	8,189824559	8,445718428	8,536558066	8,786825032	PCP016633	XM_009346536
PCP016645	7,022367813	7,244791942	6,750472519	6,672425342		
PCP016647	7,677155841	7,440204752	7,74819285	8,112022407	PCP016647	XM_008395580
PCP016652	8,204571144	8,357552005	8,445718428	8,72904287		
PCP016665	8,156487632	8,449850215	8,303780748	8,383704292	PCP016665	XM_009366577
PCP016674	7,562242424	7,215969746	7,695785075	8,173277373	PCP016674	XR_001953775
PCP016682	7,321928095	7,824449651	8,127581695	8,868297843	PCP016682	XM_009367040
PCP016694	7,956114749	7,946438456	8,373604714	8,971543554		
PCP016720	7,584962501	7,423494135	7,653418353	8,290018847	PCP016720	XM_009373607
PCP016723	7,087462841	6,950351762	7,17990909	7,572359168		
PCP016724	7,794415866	7,607330314	7,569855608	7,440204752	PCP016724	XM_018650861
PCP016729	7,805227956	8,252665432	8,503825738	9,159871337	PCP016729	XM_009370109
PCP016755	8,372125027	8,146339557	8,042589623	7,725673	PCP016755	XM_009371753
PCP016758	7,992485211	7,707359132	7,982993575	8,28077077	PCP016758	XM_009371756
PCP016760	7,076815597	7,260496187	6,853870927	8,233619677	PCP016760	XM_009347557
PCP016764	7,90285744	8,007475849	8,105018005	8,39373366	PCP016764	XM_009347561
PCP016770	7,453929061	7,464913269	7,798763389	8,363390129	PCP016770	XM_009350476
PCP016784	7,412188747	7,50517639	7,6794801	8,103287808	PCP016784	XM_008344750
PCP016790	7,54689446	7,166615031	7,125878364	6,890811455	PCP016790	XM_009340499
PCP016791	7,982993575	7,757089938	7,63175911	7,832890014	PCP016791	XM_009366206
PCP016799	8,493174961	8,347267018	7,494495616	6,667750232	PCP016799	XM_008361670

PCP016808	6,918863237	6,624539604	7,029784146	7,539158811	PCP016808	XM_009366182
PCP016831	7,058857621	7,294620749	7,544346278	8,058911723		
PCP016835	8,946409212	8,174925683	7,87036472	7,541716163	PCP016835	XM_009348049
PCP016838	8,341318667	8,233619677	8,361943774	8,13442632		
PCP016842	7,847558521	8,105018005	8,309840108	8,251103639	PCP016842	XM_008344392
PCP016850	8,429071922	8,489165579	8,45532722	8,406545173	PCP016850	XM_017336317
PCP016858	8,279239123	8,348728154	8,464872438	8,90288758	PCP016858	XM_008390328
PCP016862	7,520972191	7,189824559	7,453929061	7,994353437	PCP016862	XM_008368614
PCP016883	8,164906927	8,188193194	8,285402219	8,692336525	PCP016883	XM_009339217
PCP016912	7,515699838	7,63175911	7,499845887	7,513095909	PCP016912	XM_018649934
PCP016932	7,285402219	7,018700931	6,72342204	6,523561956	PCP016932	XM_009360950
PCP016934	8,268331458	8,335390355	8,288496992	8,296136161	PCP016934	XM_008368139
PCP016935	8,804131021	8,58747751	8,611024797	8,640244936	PCP016935	XM_008360909
PCP016960	8,354602022	8,129283017	8,029728942	8,098032083	PCP016960	XM_009351364
PCP016982	7,14974712	6,84962403	6,777025123	6,910852562		
PCP016983	7,794415866	7,617136829	7,890750668	8,407989993	PCP016983	XM_009351081
PCP016994	8,514398461	8,3994702	8,976334992	9,312134193	PCP016994	XM_008351763
PCP016996	8,601139355	8,320439548	8,510447709	8,380807357	PCP016996	XM_009352755
PCP017016	8,016808288	8,222408523	8,549476655	8,705044823	PCP017016	XM_009379519
PCP017021	7,375039431	7,779194046	7,912889336	9,122388003		
PCP017030	7,614709844	7,686500527	7,952275317	8,3994702	PCP017030	XM_009373116
PCP017048	8,407989993	8,139551352	8,296136161	8,61717357	PCP017048	XM_009344066
PCP017099	7,711976642	8,013071384	7,960001932	7,750405521	PCP017099	XM_009337013
PCP017127	7,42063398	7,811663685	7,677155841	8,30833903	PCP017127	XM_009361830
PCP017130	8,37794796	8,064311643	8,196380818	8,115459877	PCP017130	XM_009361870
PCP017134	7,50517639	6,874428132	6,946379968	7,552054236	PCP017134	XM_008369429
PCP017142	7,17990909	7,845490051	8,241601875	9,337621902	PCP017142	XM_009354251
PCP017179	6,518377768	6,714245518	6,934634441	7,269874722	PCP017179	XM_009379328
PCP017181	8,060695932	7,562242424	7,811663685	7,845490051	PCP017181	XM_009379333
PCP017182	7,055282436	7,196430001	7,111970261	6,686500527	PCP017182	XM_009379271
PCP017187	8,060695932	8,19313106	8,39373366	8,645045922	PCP017187	XM_018991081
PCP017228	7,153095972	6,648609245	9,830245815	8,933690655	PCP017228	XM_009359507
PCP017230	8,485145023	8,86727874	8,943481842	9,028817757	PCP017230	XM_009359510
PCP017235	7,922851957	8,007475849	8,046196362	7,828644274		
PCP017243	7,961912672	7,417852515	6,807354922	6,942514505		
PCP017251	7,037052702	7,098032083	7,383704292	7,932687205	PCP017251	XM_009340147
PCP017255	8,159871337	8,407989993	8,54689446	9,279215904	PCP017255	XM_009359853
PCP017258	7,440204752	7,186559982	7,50779464	7,896816753		
PCP017262	8,024197765	8,622051819	8,171577143	8,389437748	PCP017262	XM_017329987
PCP017275	6,790120385	7,037052702	7,536596918	7,473056289	PCP017275	XM_008374865
PCP017278	7,28845085	7,526147081	7,389480771	7,037052702	PCP017278	XM_009344784
PCP017302	7,672425342	7,142923928	6,50779464	6,534030467	PCP017302	XM_009364919
PCP017329	8,372125027	8,386595423	8,541754876	8,851749041		
PCP017342	9,188168462	8,74708524	8,344295908	8,21916852	PCP017342	XM_008365099
PCP017353	8,797661526	8,872428639	8,886702554	8,632995197	PCP017353	XM_008371771
PCP017355	7,417852515	7,392317423	7,584962501	8,603626345	PCP017355	XM_009347357

PCP017386	7,22881869	7,499845887	7,515699838	7,779194046	PCP017386	XM_009381184
PCP017395	7,709635275	7,815895594	8,299208018	7,641473777	PCP017395	XM_009350007
PCP017397	6,938638658	7,022367813	7,235248379	7,544346278	PCP017397	XM_008342691
PCP017402	6,97349648	6,961970533	7,007531912	7,711976642	PCP017402	XM_008363442
PCP017427	6,648609245	6,658211483	6,874428132	7,400879436		
PCP017437	8,463524373	8,290018847	8,441574774	8,827596761	PCP017437	XR_527756
PCP017438	8,04072808	8,141238626	7,975389442	7,882643049	PCP017438	XM_008394178
PCP017440	7,153095972	7,549438149	7,918863237	8,078577814		
PCP017448	7,510408147	7,614709844	7,602364826	8,186510462		
PCP017451	8,274587815	8,41502341	8,767091963	9,242388143	PCP017451	XM_009339659
PCP017481	8,335390355	8,347267018	8,317412614	8,260543232	PCP017481	XM_009343348
PCP017484	8,137862104	8,139551352	8,42488011	8,764871591	PCP017484	XM_009356941
PCP017485	8,318904285	8,194756854	7,920829209	7,577428828	PCP017485	XM_018647257
PCP017526	6,894817763	7,303780748	7,21916852	7,798763389	PCP017526	XM_009340532
PCP017589	7,930737338	7,952275317	7,992485211	7,61227877	PCP017589	XM_018650315
PCP017591	7,824449651	7,87036472	8,044394119	9,021451966		
PCP017595	7,044394119	7,202907418	7,029784146	6,754887502	PCP017595	XM_018645609
PCP017605	6,81159947	7,273049587	7,667679281	8,060695932	PCP017605	XM_018644791
PCP017610	8,491853096	8,32494558	8,86727874	8,320439548	PCP017610	XM_009346733
PCP017615	7,651051691	6,741466986	7,282347131	6,95419631	PCP017615	XM_009352083
PCP017657	7,073284692	7,269874722	7,037052702	6,946379968	PCP017657	XM_008384947
PCP017686	7,776959347	7,617136829	7,604886762	7,594921715	PCP017686	XM_009352395
PCP017721	7,677155841	7,681800619	8,04072808	8,731319031	PCP017721	XM_009337416
PCP017722	6,705010253	6,950351762	7,607330314	7,544346278		
PCP017734	8,787902559	8,382278171	8,412231097	8,232037117	PCP017734	XM_009353431
PCP017750	6,634157606	7,282347131	7,266786541	8,956143797	PCP017750	XM_008373542
PCP017758	7,080337882	7,354646096	7,63175911	7,845490051	PCP017758	XM_009346391
PCP017762	8,609806663	8,578674597	8,273002948	8,673591756	PCP017762	XM_009346404
PCP017793	8,173277373	7,888743249	7,853808564	7,709635275	PCP017793	XM_009371487
PCP017808	8,191454081	7,851749041	8,035238993	7,979167931	PCP017808	XM_009371466
PCP017811	8,594959026	8,670054263	8,74819285	9,236803511	PCP017811	XM_009371461
PCP017820	8,866258916	8,849655303	8,796591265	8,567309664	PCP017820	XM_008392419
PCP017830	8,294620749	8,524855097	8,601139355	8,792237182	PCP017830	XM_009353991
PCP017832	7,494495616	7,523561956	7,745976779	8,064311643	PCP017832	XM_009353985
PCP017833	7,153095972	7,395148508	7,486473046	7,828644274	PCP017833	XR_666756
PCP017842	7,321928095	7,375039431	7,711976642	7,324900589	PCP017842	XM_009353968
PCP017862	8,54303182	7,958088658	8,336863563	8,696967526	PCP017862	XM_018645065
PCP017875	6,820178962	6,942514505	7,464913269	7,291585141	PCP017875	XM_008384513
PCP017878	7,811663685	7,994353437	7,92677039	7,693486957	PCP017878	XM_008367726
PCP017890	6,772545519	7,156538193	7,357552005	7,700439718		
PCP017894	8,495855027	8,648645193	8,612241904	9,118941073		
PCP017902	8,919846558	8,431831861	8,803065551	8,449850215	PCP017902	XM_009362683
PCP017943	8,075051225	8,027905997	8,042589623	7,960001932		
PCP017946	8,22881869	8,296136161	8,335390355	8,159871337	PCP017946	XM_018643525
PCP017955	7,50247382	7,805227956	8,141238626	8,282300792		
PCP017958	7,17990909	7,395148508	7,50247382	7,77036657	PCP017958	XM_008382775

PCP017983	7,21916852	7,520972191	7,426264755	7,276124405		
PCP017987	8,395191361	8,665335917	8,557157324	8,482485305	PCP017987	XM_017333049
PCP017988	8,005624549	7,916894583	8,108524457	8,434628228	PCP017988	XM_009372927
PCP017990	7,491853096	7,50247382	7,750405521	8,022367813	PCP017990	XM_009372932
PCP018013	7,076815597	6,862079387	7,251056285	7,878541438	PCP018013	XM_009368153
PCP018014	8,583722152	8,611024797	8,658211483	8,545620932	PCP018014	XM_018649646
PCP018040	8,770399609	8,478446064	8,539158811	8,501160452	PCP018040	XM_009340210
PCP018051	8,730198386	8,622051819	8,779161208	8,453970202	PCP018051	XM_009373988
PCP018054	8,044394119	8,156487632	8,576181982	9,100662339	PCP018054	NM_001328811
PCP018055	7,557119022	7,348728154	7,464913269	7,251056285	PCP018055	XM_018650944
PCP018060	8,338379842	8,291539098	8,479780264	8,907882108		
PCP018067	8,553322101	8,031604721	8,555854491	8,652235507		
PCP018070	8,74819285	8,765965634	8,62450305	8,536558066	PCP018070	XM_009374034
PCP018083	7,383704292	7,260496187	7,653418353	8,009380767	PCP018083	XM_008373564
PCP018084	8,753785022	8,686500527	8,618385502	8,589950702		
PCP018086	6,914923239	7,324900589	7,46760555	7,607330314	PCP018086	XM_008373557
PCP018088	8,101555535	7,982993575	8,201290788	8,73470962		
PCP018097	7,122362117	6,54951516	6,86628983	7,08395793	PCP018097	XM_018651997
PCP018113	8,144658243	7,95419631	7,992485211	7,77478706	PCP018113	XM_009378987
PCP018127	8,885696373	8,897845456	8,877529547	8,577428828		
PCP018133	7,44567705	7,363433935	7,156538193	6,80309785	PCP018133	XM_009335319
PCP018152	8,396604781	8,599912842	8,851749041	8,726797165	PCP018152	XM_009359706
PCP018160	8,447083226	8,265239967	8,285402219	8,341318667		
PCP018211	7,453929061	7,456724026	7,809478757	8,053491515	PCP018211	XM_009359684
PCP018214	7,950293432	7,828644274	7,95419631	7,839203788	PCP018214	XM_009359679
PCP018216	8,505136683	8,338379842	8,567309664	8,924812504	PCP018216	XM_009359677
PCP018218	8,101555535	7,732472726	7,811663685	7,813781191	PCP018218	XM_009356960
PCP018230	8,113742166	7,87036472	8,073231127	8,522267655	PCP018230	XM_009356986
PCP018234	7,776959347	7,544346278	7,622051819	7,076815597	PCP018234	XM_009352196
PCP018250	7,389480771	7,163196797	7,448488033	7,866228001	PCP018250	XM_008375347
PCP018262	8,409390936	8,506486109	8,518338423	8,440163216	PCP018262	XM_008383965
PCP018273	7,599912842	7,539158811	7,309885571	7,260496187	PCP018273	XM_009370037
PCP018275	8,75598914	8,562242424	8,831845581	9,058884672	PCP018275	XM_008395043
PCP018278	6,763809907	6,926829678	7,282347131	8,051643995		
PCP018284	7,779194046	7,714245518	6,857980995	5,403608584	PCP018284	XM_008378967
PCP018286	7,166615031	7,189824559	7,330916878	7,757089938	PCP018286	XM_008349136
PCP018306	8,201290788	8,127581695	8,198052084	8,73809226	PCP018306	XM_009341187
PCP018330	7,763743526	7,900866808	7,896816753	7,688809791		
PCP018333	7,497213158	6,926829678	6,454011343	6,772545519	PCP018333	XM_008354010
PCP018338	7,807354922	7,888743249	8,075051225	8,354602022		
PCP018349	6,62935662	7,22881869	7,018700931	7,584962501	PCP018349	XM_008373368
PCP018358	7,946438456	7,434628228	7,745976779	8,048050866	PCP018358	XM_017322866
PCP018365	8,130982335	7,622051819	7,757089938	7,809478757	PCP018365	XM_009338802
PCP018371	7,763743526	7,61227877	8,171577143	7,998139076	PCP018371	XM_018646306
PCP018400	8,849655303	8,723387907	8,72904287	8,639051236	PCP018400	XM_008369265
PCP018403	7,312882955	7,486473046	7,855927425	8,8008999		

PCP018423	6,564835417	7,22881869	6,894817763	7,342785837	PCP018423	XM_018650035
PCP018449	6,599912842	6,599912842	6,958030641	7,324900589	PCP018449	XM_009352376
PCP018455	8,906890596	9,023283079	8,762647796	8,449850215	PCP018455	XM_018651009
PCP018466	7,979167931	8,403736386	8,825499451	9,582461918	PCP018466	XM_009374359
PCP018485	7,90285744	7,857980995	8,058911723	8,3994702	PCP018485	XM_018643249
PCP018502	6,634157606	6,984931073	7,199672345	8,576181982	PCP018502	XM_009340709
PCP018509	7,437377568	7,499845887	7,783522135	7,44567705	PCP018509	XM_008371846
PCP018515	8,136119594	8,125826717	8,336863563	8,740354199		
PCP018530	8,718806834	8,589950702	8,686500527	9,213517401	PCP018530	XM_009361212
PCP018549	8,640244936	8,336863563	8,348728154	8,458078458	PCP018549	XM_009336804
PCP018554	7,011227255	7,129283017	7,336908182	7,843418611	PCP018554	XM_008384109
PCP018562	6,714245518	6,759289016	7,222360218	7,395148508	PCP018562	XM_009347259
PCP018576	7,437377568	7,577428828	8,035238993	7,975389442	PCP018576	XM_009376701
PCP018604	8,456682963	8,364878797	8,569855608	8,97441459	PCP018604	XM_008353409
PCP018610	7,321928095	7,459431619	7,584962501	7,942514505	PCP018610	XM_018648886
PCP018614	9,066977144	8,847589839	8,540438054	8,238404739		
PCP018624	8,48651327	8,514398461	8,79983204	9,005624549		
PCP018635	7,539158811	7,42063398	7,730164413	8,078577814		
PCP018642	8,031604721	8,19313106	8,254225537	8,628153873	PCP018642	XM_009366009
PCP018646	6,86628983	7,062531903	7,118941073	6,695854658	PCP018646	XM_009343613
PCP018647	7,589988142	7,718841075	7,626950122	7,539158811	PCP018647	XM_009343612
PCP018650	7,206232954	7,266786541	7,483815777	7,88062431	PCP018650	XM_017329461
PCP018657	8,363390129	8,398016818	8,357552005	8,936637939	PCP018657	XM_009363783
PCP018666	7,186559982	7,497213158	7,843418611	8,183238369	PCP018666	XM_009366181
PCP018670	8,28692734	8,395191361	8,549476655	8,782441329		
PCP018692	7,518299077	7,351645995	7,375039431	7,073284692	PCP018692	XR_667777
PCP018700	7,878541438	8,106798463	8,154818109	8,459431619	PCP018700	XM_009362721
PCP018705	8,470333494	8,321928095	8,06608919	7,442943496		
PCP018726	7,768184325	7,681800619	7,826548487	8,430452552	PCP018726	XM_009373402
PCP018736	7,87036472	7,398059585	7,456724026	7,470292816	PCP018736	XM_009367146
PCP018742	8,046196362	8,291539098	8,46760555	9,055282436	PCP018742	XM_009380290
PCP018752	8,332394659	8,360495967	8,451211112	8,80841723	PCP018752	XM_009343793
PCP018756	8,024197765	7,928784831	8,153146652	8,576181982	PCP018756	XM_009381383
PCP018770	8,344295908	8,3994702	8,652235507	9,078604498	PCP018770	XM_008375544
PCP018771	8,709669735	8,659389441	8,740354199	9,105044204		
PCP018787	8,317412614	7,725673	7,686500527	7,822284016		
PCP018819	7,736943052	7,483815777	7,98111057	7,824449651	PCP018819	XM_009352191
PCP018836	7,403693786	7,670089681	7,822284016	8,293103743		
PCP018847	8,204571144	8,051643995	7,916894583	7,757089938	PCP018847	XM_009343801
PCP018861	7,372168569	7,459431619	8,273002948	11,07748336	PCP018861	XM_009345692
PCP018890	6,988684687	7,212666605	7,510408147	9,994353437	PCP018890	XM_009337573
PCP018891	7,928784831	8,048050866	8,206184105	8	PCP018891	XM_009337572
PCP018894	8,554588852	8,70158371	8,862110391	8,686500527	PCP018894	XM_008368671
PCP018936	7,528805707	7,263691734	7,470292816	7,853808564	PCP018936	XM_008393284
PCP018961	7,22881869	6,81159947	6,906890596	7,014913158		
PCP018975	7,878541438	7,61227877	8,075051225	7,847558521	PCP018975	XM_009344414

PCP018985	8,805195705	8,479780264	8,528766645	8,392317423		
PCP018989	7,886733033	7,634230226	7,828644274	7,602364826	PCP018989	XM_009342622
PCP018993	8,320439548	7,695785075	8,212715235	7,674757228	PCP018993	XM_009369234
PCP019020	8,583722152	8,018645301	8,606109055	8,536558066		
PCP019021	7,533952624	7,494495616	7,71651083	8,053491515	PCP019021	XM_009380571
PCP019022	8,379378367	8,169925001	8,255831156	8,254225537	PCP019022	XM_009355656
PCP019039	8,494535617	8,540438054	8,716545126	9,436024381	PCP019039	XM_009354331
PCP019040	6,777025123	6,977279923	7,667679281	7,007531912	PCP019040	XM_008353585
PCP019160	8,62935662	8,693486957	8,466260038	7,936637939		
PCP019182	7,142923928	7,577428828	8,048050866	8,487840034	PCP019182	XM_018650006
PCP019199	7,46760555	7,700439718	7,990557838	8,485145023	PCP019199	XM_009358618
PCP019255	7,986809101	8,005624549	7,977279923	7,634230226	PCP019255	XM_018645710
PCP019261	7,95419631	7,930737338	7,938579853	7,559721231	PCP019261	XM_018645710
PCP019269	8,166564822	7,918863237	8,186510462	8,44431092	PCP019269	XM_009355068
PCP019273	4,938756261	6,094447222	7,71651083	10,81404566	PCP019273	XM_009355064
PCP019297	7,139551352	7,17990909	6,832890014	6,579994693		
PCP019298	7,886733033	7,787902559	7,841281284	8,487840034	PCP019298	XM_008244726
PCP019307	7,470292816	6,662917555	7,400879436	6,47037417		
PCP019313	8,6794801	8,923832563	8,528766645	8,265239967	PCP019313	XM_018647929
PCP019314	7,451211112	7,212666605	7,315874125	7,073284692		
PCP019315	6,902917719	7,291585141	6,902917719	6,686500527	PCP019315	XM_009360176
PCP019319	7,196430001	7,380850638	7,186559982	6,653490009	PCP019319	XM_008396101
PCP019327	7,351645995	7,247927513	7,730164413	6,857980995	PCP019327	XM_017327517
PCP019336	8,151422517	8,178266468	8,076815597	7,950293432	PCP019336	XM_009360127
PCP019345	7,273049587	7,196430001	7,437377568	7,763743526		
PCP019376	8,293103743	8,21916852	8,28692734	8,716545126	PCP019376	XM_009372542
PCP019410	7,614709844	7,709635275	7,602364826	7,518299077		
PCP019412	7,900866808	7,672425342	6,961970533	6,196331634	PCP019412	XM_009377164
PCP019413	7,389480771	7,122362117	7,577428828	7,257387843	PCP019413	XM_009377165
PCP019422	7,122362117	7,269874722	7,626950122	7,554588852	PCP019422	XM_009377174
PCP019427	7,64385619	7,567347696	7,448488033	7,440204752		
PCP019441	8,042589623	7,930737338	7,975389442	8,694636474	PCP019441	XM_009339439
PCP019450	8,787902559	8,743723645	8,751544059	8,660602089	PCP019450	XM_009339310
PCP019464	8,494535617	8,354602022	8,581200582	8,999069838		
PCP019479	8,703903573	8,514398461	8,781359714	9,108524457	PCP019479	XM_009377673
PCP019483	8,022367813	8,247927513	8,110248388	8,042589623	PCP019483	XM_017333273
PCP019489	7,730164413	7,136170875	6,426264755	6,624539604	PCP019489	XM_009377656
PCP019499	8,003770871	7,866228001	7,888743249	7,639015048		
PCP019500	7,118941073	7,238404739	7,183188734	6,781359714	PCP019500	XM_009377635
PCP019509	7,383704292	7,655852677	8,186510462	8,545620932	PCP019509	XM_008378845
PCP019511	7,499845887	7,312882955	7,912889336	8,062477937	PCP019511	XM_008378988
PCP019527	6,599912842	6,380764075	6,886672074	7,761551232		
PCP019537	8,888743249	8,514398461	8,277659361	7,910912508	PCP019537	XM_008378904
PCP019548	7,473056289	7,132679654	7,499845887	7,818070833		
PCP019563	6,81159947	6,958030641	7,08395793	7,481153605		
PCP019574	8,139551352	8,096293483	8,106798463	7,779194046	PCP019574	XM_009372479

PCP019577	8,548166865	8,503825738	8,434628228	8,453970202	PCP019577	XM_009372503
PCP019588	8,400879436	7,988684687	8,057071136	7,928784831	PCP019588	XM_009365724
PCP019600	7,291585141	8,112022407	7,74819285	8,385129006	PCP019600	XM_009365707
PCP019603	7,874489611	7,868328714	8,049848549	8,434628228	PCP019603	XM_009364311
PCP019614	7,888743249	8,129283017	8,667714757	9,150585062		
PCP019616	8,317412614	8,422064766	8,466260038	8,062477937	PCP019616	XM_009367176
PCP019629	8,447083226	8,511752654	8,73809226	9,098032083		
PCP019637	6,996275749	7,173227395	7,139551352	6,459431619	PCP019637	XM_008357526
PCP019645	8,473015688	8,263644792	8,503825738	8,306836389	PCP019645	XM_009339553
PCP019649	8,220765252	7,763743526	7,617136829	6,977279923	PCP019649	XM_009336173
PCP019672	7,372168569	7,557119022	7,670089681	7,994353437	PCP019672	XM_009357508
PCP019673	8,806291831	8,680640826	8,631795481	8,246360579		
PCP019677	8,654636029	8,553322101	8,622051819	8,48651327	PCP019677	XM_009357518
PCP019693	8,303780748	8,271463028	8,225593068	7,727920455	PCP019693	XM_009357616
PCP019712	8,022367813	8,016808288	7,898873426	7,686500527		
PCP019720	8,266786541	8,372125027	8,489165579	8,845490051	PCP019720	XM_009342996
PCP019769	8,279239123	8,201290788	8,154818109	8,659389441	PCP019769	XM_009360867
PCP019795	7,094552786	6,84962403	7,101503009	6,60481265	PCP019795	XM_017326043
PCP019797	7,853808564	7,641473777	8,113742166	8,56350137	PCP019797	XM_009354635
PCP019800	8,388017285	8,375039431	8,473015688	9,224001674	PCP019800	XM_009354638
PCP019819	8,110248388	8,224001674	8,390899794	8,61717357	PCP019819	XM_008366830
PCP019824	7,961912672	7,641473777	7,62935662	7,285402219	PCP019824	XM_018642625
PCP019833	8,033423002	7,700439718	7,886733033	8,209453366	PCP019833	XM_009335617
PCP019849	7,238404739	7,497213158	7,820178962	8,174925683	PCP019849	XM_008375761
PCP019866	6,648609245	6,890811455	7,132679654	7,412188747		
PCP019869	7,156538193	7,193081765	7,982993575	8,571107931	PCP019869	XM_009380418
PCP019870	8,732438807	8,64385619	8,70276142	9,084808388	PCP019870	XM_008347011
PCP019884	7,714245518	7,914863459	8,022367813	8,438791853	PCP019884	XM_009336394
PCP019897	7,351645995	7,434628228	7,363433935	7,17990909	PCP019897	XM_009381198
PCP019899	7,965784285	7,886733033	8,181549844	8,430452552	PCP019899	XM_009346068
PCP019917	7,297649983	7,139551352	7,21916852	6,984931073	PCP019917	XM_009336001
PCP019920	7,456724026	7,531381461	7,890750668	8,276124405	PCP019920	XM_017336911
PCP019940	8,302273349	8,403736386	8,360495967	8,32494558	PCP019940	XM_009352207
PCP019944	7,026025399	7,440204752	7,321928095	7,681800619	PCP019944	XM_018652200
PCP019945	8,007475849	7,950293432	7,996219247	7,847558521		
PCP019950	8,174925683	8,105018005	8,069691427	7,952275317	PCP019950	XM_009367512
PCP019959	8,132731056	8,173277373	8,112022407	8,076815597		
PCP019974	7,900866808	7,727920455	7,956114749	8,515699838	PCP019974	XM_009374641
PCP019975	8,018645301	7,832890014	7,874489611	8,390899794		
PCP019982	9,361220052	9,369226538	8,268331458	7,285402219	PCP019982	XM_009372216
PCP019988	8,436003553	8,098032083	8,239980333	8,753785022		
PCP020005	7,851749041	8,207844058	8,569855608	9,004698008	PCP020005	XM_009355590
PCP020012	8,323415108	8,73470962	8,736976865	8,813781191	PCP020012	XM_009355598
PCP020019	8,61717357	8,596189756	8,591223118	8,471675214		
PCP020034	7,860031646	7,691185174	8,225593068	8,300718622	PCP020034	XM_008354444
PCP020036	7,589988142	7,815895594	8,057071136	8,06608919	PCP020036	XM_009345972

PCP020042	7,400879436	7,257387843	8,020535537	7,677155841	PCP020042	XM_009335421
PCP020054	8,470333494	8,339850003	8,178266468	8,305286574		
PCP020064	7,285402219	7,531381461	7,453929061	8,494535617	PCP020064	XM_009339826
PCP020071	8,407989993	8,112022407	8,493174961	8,824417827	PCP020071	XM_009355947
PCP020073	6,841344192	7,321928095	7,406587689	7,489205728	PCP020073	XM_009355942
PCP020093	6,845490051	7,215969746	7,395148508	7,342785837	PCP020093	XM_009350321
PCP020111	8,183238369	8,412231097	8,526186214	8,859006685	PCP020111	XM_009351281
PCP020134	7,014913158	7,040782866	6,894817763	6,658211483	PCP020134	XM_009355408
PCP020183	6,894817763	6,609843592	6,807354922	7,294620749	PCP020183	XM_008367646
PCP020188	7,759355602	7,572359168	7,952275317	8,112022407	PCP020188	XM_008367712
PCP020201	7,073284692	7,058857621	7,324900589	6,754887502	PCP020201	XM_009366551
PCP020203	8,120652609	8,252665432	8,293103743	8,63542792	PCP020203	XM_009366509
PCP020205	7,386552314	7,122362117	7,700439718	7,707359132	PCP020205	XM_009338109
PCP020213	7,969645536	7,878541438	7,87036472	7,741466986	PCP020213	XM_009364897
PCP020218	8,122413888	7,857980995	7,969645536	6,345715709	PCP020218	XM_018649093
PCP020221	7,260496187	7,101503009	7,499845887	7,033423002	PCP020221	XM_018645877
PCP020253	7,412188747	7,300764373	7,189824559	7,087462841		
PCP020254	8,851749041	8,224001674	7,832890014	8,120652609	PCP020254	XM_008347812
PCP020270	7,592457037	7,900866808	7,874489611	8,19313106		
PCP020281	8,046196362	7,948367232	7,772611496	8,129283017		
PCP020282	7,42063398	7,30679083	7,473056289	7,942514505	PCP020282	XM_009364671
PCP020283	7,386552314	7,369204723	8,055282436	9,980610876	PCP020283	XR_001953603
PCP020288	8,434628228	8,50779464	8,522267655	8,412231097	PCP020288	XM_009367542
PCP020319	7,285402219	7,412188747	7,634230226	7,975389442		
PCP020320	7,658211483	7,118941073	8,265239967	8,124121312	PCP020320	XM_009368912
PCP020330	7,122362117	7,266786541	7,372168569	8,035238993		
PCP020333	8,305286574	8,463524373	8,493174961	8,427648072		
PCP020359	7,822284016	8,082149041	7,684187561	7,709635275	PCP020359	XM_009379812
PCP020360	7,839203788	8	7,98111057	8,356099782		
PCP020369	7,222360218	7,426264755	7,475733431	7,860031646	PCP020369	XM_009344458
PCP020416	7,636624621	7,641473777	8,082149041	8,806291831	PCP020416	XM_009355337
PCP020421	7,969645536	7,855927425	7,594921715	7,282347131	PCP020421	XM_008394087
PCP020447	8,112022407	8,06608919	7,98111057	8,523561956	PCP020447	XM_009380457
PCP020476	8,710806434	8,685344507	8,880593701	8,589950702		
PCP020492	8,511752654	8,636624621	8,843387202	8,372125027	PCP020492	XM_009345784
PCP020497	7,044394119	7,369204723	7,62935662	8,246360579	PCP020497	XM_008377714
PCP020508	7,440204752	7,122362117	7,108524457	6,254178286	PCP020508	XM_009361526
PCP020509	8,566054038	8,366322214	8,591223118	8,877529547	PCP020509	XM_009345265
PCP020522	6,772545519	6,965784285	7,139551352	7,536596918	PCP020522	XM_018643677
PCP020532	6,714245518	6,841344192	7,30679083	8,623259662	PCP020532	XM_009369231
PCP020546	6,902917719	6,497133304	7,266786541	7,076815597	PCP020546	XM_009368848
PCP020547	7,513095909	7,291585141	7,470292816	7,007531912	PCP020547	XM_008359956
PCP020565	7,101503009	7,470292816	7,582480735	8,071462363	PCP020565	XM_008379525
PCP020582	8,103287808	7,796558821	7,824449651	7,783522135		
PCP020662	7,878541438	7,754887502	7,464913269	7,523561956		
PCP020666	7,423494135	7,533952624	8,361943774	8,173277373	PCP020666	XM_008377166

PCP020695	7,451211112	7,28845085	7,28845085	7,011227255	PCP020695	XM_009362776
PCP020729	7,928784831	7,667679281	7,691185174	7,74819285	PCP020729	XM_009365689
PCP020752	6,639087423	7,363433935	7,285402219	7,478405614	PCP020752	XM_008339325
PCP020844	7,973439079	7,526147081	8,044394119	7,554588852	PCP020844	XM_009336822
PCP020857	6,609843592	7,417852515	7,619633096	9,332417038	PCP020857	XM_009381263
PCP020880	8,283875484	8,220765252	8,243173983	7,934693407	PCP020880	XM_009377779
PCP020885	6,454011343	6,961970533	6,988684687	8,269921462	PCP020885	XM_009376843
PCP020887	7,266786541	7,77036657	7,892816	8,198052084	PCP020887	XM_009376744
PCP020901	7,589988142	7,46760555	7,648681141	8,108524457	PCP020901	XM_008365901
PCP020904	8,212715235	7,725673	7,807354922	7,569855608	PCP020904	XM_018651155
PCP020915	6,599912842	6,454011343	6,695854658	8,146339557	PCP020915	XM_008390828
PCP020916	8,273002948	8,156487632	8,174925683	8,035238993	PCP020916	XM_017326527
PCP020918	7,83080039	8,071462363	8,41502341	9,002815016	PCP020918	XM_008390833
PCP020941	7,624466495	7,8008999	8,178266468	9,001886668		
PCP020968	7,92677039	7,890750668	7,952275317	7,723353774	PCP020968	XM_008361796
PCP020975	7,557119022	7,426264755	7,677155841	8,254225537	PCP020975	XM_009373291
PCP020980	7,695785075	7,273049587	7,383704292	7,011227255	PCP020980	XM_018651400
PCP020987	8,055282436	8,254225537	8,885696373	9,849139214	PCP020987	XM_018651392
PCP020991	8,696967526	8,514398461	8,783554874	9,207819661		
PCP021002	8,44431092	8,302273349	8,470333494	8,364878797		
PCP021004	8,014968933	7,884658968	7,862141394	7,279192684	PCP021004	XM_018651878
PCP021005	7,125878364	6,910852562	7,007531912	6,50255338	PCP021005	XM_008339955
PCP021006	7,815895594	7,453929061	7,602364826	7,597456684	PCP021006	XM_009378179
PCP021014	8,586201784	8,297695831	8,54689446	8,35169016	PCP021014	XM_009360723
PCP021022	6,828707735	6,700439718	6,667750232	7,684187561		
PCP021041	7,866228001	7,967687386	7,973439079	8,628153873	PCP021041	XM_009351243
PCP021061	6,667750232	6,64385619	7,044394119	7,518299077		
PCP021105	7,176621973	7,459431619	7,076815597	6,969587981		
PCP021112	8,370687407	8,524855097	8,882643049	9,258165557		
PCP021122	7,634230226	7,648681141	7,579919292	7,389480771	PCP021122	XM_009365499
PCP021127	6,624539604	6,420718183	7,098032083	8,856954575	PCP021127	XM_009365506
PCP021130	8,583722152	8,74708524	8,84024291	9,110274492		
PCP021137	8,075051225	8,166564822	8,255831156	8,626913629		
PCP021142	6,977279923	7,577428828	8,048050866	7,783522135	PCP021142	XM_009365602
PCP021143	7,251056285	7,448488033	7,528805707	7,979167931	PCP021143	NM_001302302
PCP021145	8,204571144	8,296136161	7,574934058	7,486473046	PCP021145	XM_018649055
PCP021159	7,910912508	7,932687205	7,928784831	7,582480735	PCP021159	XM_009337262
PCP021160	8,537878433	8,537878433	8,37794796	8,413627929		
PCP021175	7,244791942	7,426264755	7,587440004	8,078577814	PCP021175	XM_009337232
PCP021179	6,977279923	6,958030641	7,300764373	6,914923239	PCP021179	XM_009337227
PCP021184	7,176621973	6,922792504	6,958030641	6,64385619	PCP021184	XM_009375029
PCP021193	7,971543554	7,761551232	7,763743526	7,872397856	PCP021193	XM_009344297
PCP021203	7,159871337	7,007531912	7,619633096	9,11113567	PCP021203	XM_008389050
PCP021207	8,417852515	8,306836389	8,356099782	8,939579214	PCP021207	XM_008389077
PCP021209	6,129283017	6,820178962	7,809478757	7,636624621	PCP021209	XM_017327502
PCP021222	6,648609245	6,705010253	7,033423002	7,54689446	PCP021222	XM_008389190

PCP021228	7,979167931	8,188193194	7,888743249	7,761551232		
PCP021239	8,060695932	8,027905997	8,198052084	8,583722152		
PCP021241	7,988684687	8	8,016808288	7,896816753		
PCP021251	7,434628228	7,50779464	7,670089681	8,255831156	PCP021251	XM_008373345
PCP021259	8,051643995	8,309840108	8,659389441	9,098032083	PCP021259	XM_008365963
PCP021260	7,470292816	8,087462841	7,864186145	7,686500527	PCP021260	XM_008365964
PCP021261	7,594921715	8,146339557	7,8008999	7,73470962	PCP021261	XM_017332465
PCP021293	8,452570726	8,188193194	8,518338423	8,68183575	PCP021293	XM_008342383
PCP021301	7,857980995	7,98111057	8,163247124	8,63542792	PCP021301	XM_009378842
PCP021306	7,533952624	7,783522135	8,191454081	9,098900598	PCP021306	XM_009378839
PCP021314	7,969645536	7,95419631	8,011227255	8,413627929	PCP021314	XM_009378827
PCP021321	8,225593068	8,214319121	8,39373366	8,830768706	PCP021321	XM_009345583
PCP021331	6,981053471	6,882643049	6,926829678	8,027905997	PCP021331	XM_009345670
PCP021332	8,470333494	8,717676423	8,699294818	9,121533517		
PCP021339	7,044394119	7,125878364	7,28845085	7,709635275		
PCP021340	8,19313106	7,884658968	8,003770871	7,494495616		
PCP021348	7,333870928	7,552054236	7,963878669	8,380807357	PCP021348	XM_008377315
PCP021385	7,942514505	7,986809101	8,076815597	8,452570726	PCP021385	XM_008384193
PCP021409	8,583722152	8,601139355	8,810571635	9,227206781		
PCP021412	8,46760555	8,482485305	8,583722152	9,121533517	PCP021412	XM_009346001
PCP021436	7,714245518	7,74819285	7,63175911	7,351645995		
PCP021458	8,332394659	8,406545173	8,566054038	8,895817605		
PCP021460	8,827596761	8,727920455	8,89684702	8,764871591	PCP021460	XM_008340329
PCP021461	6,798698597	6,914923239	7,318949464	7,779194046		
PCP021468	6,741466986	6,72342204	7,018700931	7,483815777		
PCP021484	7,285402219	7,714245518	8,048050866	8,537878433	PCP021484	XM_008388933
PCP021486	8,730198386	8,790087794	8,73809226	8,566054038	PCP021486	XM_008364807
PCP021488	7,63175911	7,193081765	8,073231127	8,620842965	PCP021488	XM_009372431
PCP021504	6,918863237	6,790120385	6,695854658	7,351645995	PCP021504	XM_009372133
PCP021511	7,08395793	7,442943496	7,406587689	7,98111057	PCP021511	XM_009370440
PCP021516	7,888743249	8,290018847	7,967687386	8,265239967	PCP021516	XM_009341163
PCP021559	7,888743249	8,108524457	8,132731056	8,520932916		
PCP021572	7,040782866	7,312882955	7,132679654	6,691115365		
PCP021582	7,707359132	7,792269724	8,053491515	8,962896005		
PCP021588	7,599912842	7,651051691	8,101555535	8,772578508	PCP021588	XM_009340157
PCP021604	7,677155841	7,908872939	8,022367813	8,345760055	PCP021604	XM_009349014
PCP021606	7,98111057	7,888743249	8,103287808	8,510447709	PCP021606	XM_008345086
PCP021612	8,159871337	7,483815777	7,206232954	6,497133304	PCP021612	XM_018651931
PCP021619	7,557119022	7,847558521	8,215921225	8,658211483		
PCP021645	7,845490051	7,779194046	7,958088658	8,305286574	PCP021645	XM_009377302
PCP021651	6,409390936	6,686500527	7,928784831	7,098032083	PCP021651	XM_008382199
PCP021671	7,08395793	7,279192684	7,417852515	7,986809101	PCP021671	XM_008369499
PCP021684	7,011227255	6,824386003	7,342785837	7,544346278	PCP021684	XM_018647054
PCP021685	8,555854491	8,714245518	8,62935662	9,059804103	PCP021685	XM_018647053
PCP021692	8,035238993	8,330916878	8,675957033	8,619596417	PCP021692	XM_008357078
PCP021699	7,011227255	6,95419631	7,062531903	7,864186145		

PCP021716	8,883651361	9,032514148	8,615923847	8,122413888	PCP021716	XM_009345262
PCP021718	8,007475849	8,087462841	8,294620749	8,493174961		
PCP021724	7,866228001	7,672425342	7,743689989	8,412231097		
PCP021727	8,082149041	8,265239967	8,364878797	8,665335917	PCP021727	XM_017333361
PCP021731	8,189824559	7,607330314	7,702726796	7,597456684	PCP021731	XM_008354953
PCP021735	7,451211112	7,785746699	7,74819285	8,035238993	PCP021735	XM_009368500
PCP021756	8,103287808	7,98111057	8,251103639	8,545620932	PCP021756	XM_008384098
PCP021783	7,609769734	7,841281284	7,946438456	8,326429487	PCP021783	XM_009366239
PCP021786	8,45532722	8,438791853	8,392317423	8,113742166		
PCP021794	7,741466986	8,41079052	8,300718622	8,764871591	PCP021794	XM_009364861
PCP021808	6,785681319	7,17990909	7,21916852	7,494495616	PCP021808	XM_008357444
PCP021819	8,189824559	8,246360579	8,407989993	8,249492747	PCP021819	XM_008363703
PCP021823	8,715378619	8,626913629	8,780277286	9,159871337	PCP021823	XR_669751
PCP021865	8,309840108	8,350187812	8,353146825	8,215921225	PCP021865	XM_009379864
PCP021875	8,780277286	7,691185174	7,080337882	5,906890596	PCP021875	XM_009354267
PCP021897	8,184875343	8,336863563	8,445718428	8,686500527	PCP021897	XM_009377867
PCP021909	7,526147081	7,279192684	7,528805707	7,062531903	PCP021909	XM_009337195
PCP021923	7,400879436	7,380850638	7,670089681	8,209453366	PCP021923	XM_009358440
PCP021931	7,348728154	7,206232954	6,878602742	6,942514505	PCP021931	XM_009364468
PCP021936	6,878602742	6,882643049	7,294620749	7,526147081		
PCP021944	7,520972191	7,648681141	7,818070833	8,268331458		
PCP021948	7,22881869	7,483815777	7,88062431	8,168270966	PCP021948	XM_009374933
PCP021951	8,168270966	8,189824559	8,053491515	7,977279923	PCP021951	XM_018642384
PCP021953	7,395148508	7,572359168	7,750405521	8,027905997		
PCP021955	8,835008208	8,329437582	8,48651327	8,073231127	PCP021955	XM_009335438
PCP021956	8,110248388	8,19313106	7,896816753	7,698149009	PCP021956	XM_009335440
PCP021958	8,503825738	8,320439548	8,50779464	8,335390355	PCP021958	XM_008367671
PCP021959	7,238404739	7,260496187	7,513095909	8,513056419		
PCP021964	7,263691734	7,549438149	7,952275317	8,290018847	PCP021964	XM_009349425
PCP021977	8,089212109	8,08571145	8,049848549	8,540438054		
PCP021979	7,62935662	7,752681699	7,888743249	8,164906927	PCP021979	XM_009335712
PCP022006	8,069691427	8,560982378	8,723387907	9,082149041	PCP022006	XM_008374462
PCP022015	7,54689446	7,266786541	7,354646096	7,069637728		
PCP022026	7,567347696	5,77267747	7,648681141	9,553302899	PCP022026	NM_001302284
PCP022036	7,159871337	7,291585141	7,497213158	8,013071384		
PCP022037	8,016808288	7,772611496	7,787902559	7,868328714		
PCP022038	8,466260038	8,48651327	8,262094845	8,254225537	PCP022038	XM_009339271
PCP022043	6,624539604	7,026025399	7,047996356	7,377904593	PCP022043	XM_009352046
PCP022051	7,994353437	8,024197765	8,309840108	8,60855054	PCP022051	XM_017335882
PCP022057	7,062531903	6,977279923	7,309885571	7,660566438	PCP022057	XM_008390598
PCP022070	7,392317423	7,429030064	7,882643049	7,159871337	PCP022070	XM_008375078
PCP022081	7,483815777	7,515699838	7,363433935	7,136170875	PCP022081	XM_009359878
PCP022087	6,539158811	6,523561956	6,857980995	7,604886762	PCP022087	XM_009361444
PCP022103	7,118941073	7,14974712	7,44567705	7,723353774	PCP022103	XM_009361494
PCP022104	7	7,06608919	7,260496187	7,916894583	PCP022104	XM_009361486
PCP022109	8,038918989	7,969645536	8,201290788	8,532667615		

PCP022110	8,255831156	8,462175047	8,579956993	9,10938668	PCP022110	XM_009350147
PCP022122	8,379378367	8,247927513	8,431831861	9,044394119	PCP022122	XM_009364283
PCP022137	7,752681699	8,370687407	8,306836389	8,181549844		
PCP022144	7,658211483	7,698149009	7,878541438	8,274587815	PCP022144	XM_009352860
PCP022145	7,303780748	7,481153605	7,776959347	7,998139076		
PCP022168	8,844454703	8,239980333	8,033423002	8,215921225	PCP022168	XM_009352988
PCP022192	8,459431619	8,50779464	8,579956993	9,413627929	PCP022192	XM_008389930
PCP022201	8,09976859	7,754887502	8,385129006	8,105018005	PCP022201	NM_001302320
PCP022204	7,539158811	7,453929061	7,750405521	8,257387843	PCP022204	XM_008392572
PCP022237	8,448446735	8,56350137	8,416459769	8,062477937	PCP022237	XM_009364157
PCP022271	7,541716163	7,348728154	7,406587689	6,890811455	PCP022271	XM_008345793
PCP022285	8,372125027	8,089212109	7,950293432	7,269874722	PCP022285	XM_018652419
PCP022359	8,184875343	8,506486109	8,154818109	8,694636474	PCP022359	XM_009379889
PCP022362	7,973439079	7,92677039	7,815895594	7,658211483	PCP022362	XM_009339895
PCP022365	8,132731056	7,884658968	8,258942852	7,658211483		
PCP022367	7,528805707	7,510408147	7,459431619	7,321928095	PCP022367	XM_008352870
PCP022373	8,108524457	8,335390355	8,479780264	8,74708524		
PCP022424	7,260496187	7,333870928	7,624466495	8,383704292	PCP022424	XM_009376791
PCP022484	6,97349648	7,111970261	7,700439718	7,938579853		
PCP022487	8,54303182	8,776992236	8,757123282	9,095397023	PCP022487	XM_008388346
PCP022520	8,08390476	8,124121312	8,900866808	9,605479518		
PCP022535	7,963878669	8,120652609	8,037107627	8,462175047	PCP022535	XM_009355515
PCP022543	8,366322214	8,053491515	8,13442632	8,163247124	PCP022543	XM_009355504
PCP022552	7,533952624	7,169925001	7,285402219	7,354646096		
PCP022560	6,727920455	6,709704193	7,225641265	7,50517639	PCP022560	XM_009355458
PCP022562	7,920829209	7,868328714	7,579919292	6,97349648		
PCP022569	7,403693786	7,202907418	7,375039431	7,783522135	PCP022569	XM_009337088
PCP022585	8,396604781	8,54689446	8,776992236	9,193944186	PCP022585	XM_009337271
PCP022588	8,091012169	7,820178962	8,117227504	8,379378367	PCP022588	XM_009337250
PCP022598	6,667750232	6,785681319	7,044394119	7,510408147	PCP022598	XM_009337381
PCP022604	8,254225537	8,501160452	8,369248353	8,968666793	PCP022604	XM_009337426
PCP022625	7,62935662	7,626950122	7,670089681	7,440204752		
PCP022626	8,078577814	8,14974712	8,113742166	8,613494819	PCP022626	XR_667130
PCP022633	7,026025399	7,403693786	7,494495616	7,77478706	PCP022633	XM_009357104
PCP022638	7,944448531	8,033423002	8,202956379	8,678318438	PCP022638	XM_017333803
PCP022649	8,08390476	8,263644792	8,37794796	8,596189756		
PCP022652	8,277659361	8,049848549	8,14974712	8,038918989		
PCP022662	7,108524457	6,781359714	7,442943496	7,206232954	PCP022662	XM_009379721
PCP022664	8,568602197	8,502513601	8,6794801	9,405843489		
PCP022690	7,87036472	8	7,787902559	7,552054236		
PCP022694	8,475733431	8,675957033	8,857980995	9,310612782	PCP022694	XM_009379770
PCP022709	8,206184105	7,691185174	7,878541438	7,866228001	PCP022709	XM_009379798
PCP022714	6,781359714	7,069637728	7,176621973	7,757089938		
PCP022724	8,21106088	7,967687386	8,124121312	7,890750668	PCP022724	XM_009346102
PCP022729	7,417852515	7,624466495	7,711976642	7,415065677		
PCP022734	7,811663685	7,730164413	7,876516947	7,105070402	PCP022734	XM_018652367

PCP022737	7,136170875	7,544346278	7,069637728	7,044394119	PCP022737	XM_009380532
PCP022740	7,50779464	7,470292816	7,303780748	7,822284016	PCP022740	XM_009377483
PCP022756	8,329437582	7,864186145	8,011227255	7,783522135	PCP022756	XM_009341058
PCP022767	8,28692734	8,283875484	8,32791187	7,890750668	PCP022767	XM_008368309
PCP022784	6,926829678	7,007531912	7,222360218	7,564759219	PCP022784	XM_009339367
PCP022785	7,073284692	6,996275749	7,339850003	7,641473777	PCP022785	XM_009339368
PCP022786	8,092757141	7,765998774	7,998139076	7,634230226		
PCP022795	7,587440004	7,499845887	7,988684687	7,874489611	PCP022795	XM_009339362
PCP022816	7,429030064	7,273049587	7,386552314	7,047996356	PCP022816	XM_009367760
PCP022825	9,14381685	8,724513853	8,32494558	8,54303182	PCP022825	XM_008394722
PCP022828	8,335390355	8,257387843	7,847558521	8,174925683	PCP022828	XM_009346296
PCP022831	7,417852515	7,491853096	7,333870928	8,051643995		
PCP022843	7,058857621	7,389480771	7,291585141	7,896816753	PCP022843	XM_009346134
PCP022844	7,956114749	7,727920455	7,855927425	8,332394659		
PCP022846	7,159871337	7,21916852	7,956114749	8,568602197	PCP022846	XM_009346095
PCP022879	7,429030064	7,483815777	7,63175911	8,011227255	PCP022879	XM_009352412
PCP022893	7,122362117	6,890811455	7,132679654	7,64385619	PCP022893	XM_008358165
PCP022901	7,139551352	7,254272787	7,231989133	6,97349648	PCP022901	XM_009350259
PCP022926	6,950351762	8,246360579	8,369248353	7,672425342	PCP022926	XM_008380353
PCP022934	8,41079052	8,390899794	8,269921462	8,647458426	PCP022934	XM_008349562
PCP022945	7,129283017	7,247927513	7,377904593	7,811663685	PCP022945	XM_009367940
PCP022956	8,530094158	8,345760055	8,388017285	8,390899794		
PCP022957	8,276124405	8,146339557	8,28077077	8,63542792	PCP022957	XM_009367915
PCP022962	8,144658243	8,027905997	8,049848549	8,031604721	PCP022962	XM_009367895
PCP022982	8,120652609	7,648681141	7,222360218	6,882643049	PCP022982	XM_009352291
PCP022983	8,993419626	8,545620932	8,503825738	8,805195705		
PCP022991	8,514398461	8,449850215	8,672425342	9,159871337	PCP022991	XM_009368086
PCP023006	7,159871337	6,926829678	6,279285561	7,312882955		
PCP023011	7,094552786	7,215969746	7,183188734	6,946379968		
PCP023014	7,599912842	7,440204752	7,653418353	8,176572111	PCP023014	XM_009347767
PCP023021	7,526147081	7,324900589	7,257387843	6,87036472	PCP023021	XM_009364095
PCP023040	7,115511897	7,263691734	7,569855608	7,74819285		
PCP023052	7,139551352	7,369204723	7,730164413	7,971543554	PCP023052	XM_009378066
PCP023055	6,969587981	6,981053471	7,225641265	7,607330314		
PCP023059	7,592457037	7,698149009	7,589988142	7,231989133	PCP023059	XM_008377759
PCP023061	8,014968933	7,790055203	8,076815597	7,886733033	PCP023061	XR_001952709
PCP023069	7,260496187	7,241554209	7,464913269	7,853808564	PCP023069	XM_009341482
PCP023071	7,321928095	7,058857621	7,462134139	7,752681699	PCP023071	XM_021964791
PCP023086	7,845490051	7,868328714	8,037107627	8,336863563		
PCP023089	7,118941073	6,930737338	7,166615031	7,768184325		
PCP023093	8,64385619	8,202956379	8,528766645	7,702726796	PCP023093	XM_009365045
PCP023102	7,429030064	7,022367813	6,845490051	6,369291982	PCP023102	XM_018644160
PCP023122	7,754887502	7,309885571	7,456724026	7,324900589	PCP023122	XM_008372482
PCP023123	7,759355602	7,641473777	7,896816753	8,207844058	PCP023123	XM_008347492
PCP023127	6,862079387	7,108524457	7,212666605	7,497213158	PCP023127	XM_008372472
PCP023139	8,550746785	8,666508075	8,606109055	8,473015688	PCP023139	XM_008369546

PCP023151	7,634230226	7,50517639	7,470292816	7,406587689	PCP023151	XM_009340113
PCP023175	8,011227255	8,385129006	8,731319031	9,278449458		
PCP023176	8,862110391	8,554588852	8,035238993	7,169925001	PCP023176	XM_009369324
PCP023200	6,80309785	6,624539604	7,044394119	7,924812504	PCP023200	XM_009371914
PCP023212	8,606109055	8,630558365	8,820178962	8,68183575		
PCP023221	8,376472723	8,536558066	8,651051691	9,189009107		
PCP023223	8,262094845	8,293103743	8,481113232	8,894817763		
PCP023229	7,497213158	7,044394119	7,115511897	7,196430001	PCP023229	XM_018650817
PCP023231	6,942514505	7,007531912	7,531381461	8,28077077	PCP023231	XM_009373396
PCP023232	7,003714662	7,251056285	7,051698368	6,357552005	PCP023232	XM_009373395
PCP023238	7,602364826	7,641473777	7,62935662	8,651051691	PCP023238	XM_008381807
PCP023239	8,54303182	7,969645536	8,509101985	9,273795599	PCP023239	XR_001787032
PCP023243	8,510447709	8,28077077	8,163247124	8,020535537	PCP023243	XM_008367336
PCP023268	7,392317423	7,725673	7,389480771	7,037052702	PCP023268	XM_008377279
PCP023283	7,159871337	6,922792504	7,244791942	7,539158811		
PCP023286	7,336908182	7,294620749	7,648681141	7,434628228	PCP023286	XR_001787708
PCP023316	7,95419631	7,805227956	7,992485211	8,396604781		
PCP023320	8,285402219	8,666508075	8,782441329	8,309840108	PCP023320	XM_009380357
PCP023323	7,961912672	7,244791942	7,946438456	7,757089938	PCP023323	XM_009380361
PCP023326	8,395191361	8,055282436	8,106798463	8,048050866	PCP023326	XM_009381228
PCP023329	8,009380767	8,255831156	8,383704292	8,526186214		
PCP023334	7,853808564	8,159871337	8,199672345	8,08571145	PCP023334	XM_009361191
PCP023336	8,027905997	8,243173983	7,971543554	8,082149041		
PCP023340	8,153146652	8,263644792	8,649866903	8,693486957	PCP023340	XM_008394624
PCP023341	7,822284016	8,33391564	8,58747751	8,991521846	PCP023341	XM_009361200
PCP023350	7,924812504	8,033423002	7,691185174	7,456724026	PCP023350	XM_009343381
PCP023351	7,315874125	7,497213158	7,557119022	8,276124405		
PCP023356	7,718841075	7,700439718	7,602364826	7,462134139	PCP023356	XM_018648801
PCP023379	7,958088658	7,718841075	7,876516947	7,486473046	PCP023379	XM_009342097
PCP023417	7,977279923	7,952275317	8,46760555	8,74819285	PCP023417	XM_008341755
PCP023418	8,348728154	8,539158811	8,581200582	8,915879379		
PCP023419	6,777025123	6,38663853	7,087462841	7,6794801	PCP023419	XM_008341766
PCP023426	7,562242424	7,494495616	7,297649983	7,950293432	PCP023426	XM_021453800
PCP023432	7,375039431	6,594996337	7,062531903	6,790120385		
PCP023441	7,409390936	7,820178962	7,61227877	7,260496187	PCP023441	XM_008364929
PCP023447	8,573647187	8,612241904	8,72904287	9,244768161	PCP023447	XM_008363557
PCP023463	7,080337882	7,163196797	7,309885571	7,845490051		
PCP023469	8,614709844	8,761551232	8,664162806	8,692336525	PCP023469	XM_009356405
PCP023473	8,473015688	9,047123912	8,684152487	8,367764188	PCP023473	XM_018642781
PCP023475	8,113742166	8,09976859	7,967687386	8,020535537	PCP023475	XM_009337149
PCP023496	9,015888903	8,58747751	8,703903573	8,966736149	PCP023496	XM_009345055
PCP023500	7,695785075	7,475733431	7,309885571	7,475733431	PCP023500	XM_009347979
PCP023510	8,434628228	8,596189756	8,654636029	8,348728154	PCP023510	XM_009380801
PCP023527	8,181549844	8,569855608	8,523561956	8,434628228	PCP023527	XM_008365240
PCP023559	7,282347131	7,115511897	7,282347131	7,700439718		
PCP023560	7,437377568	7,186559982	7,98111057	10,28924659		

PCP023578	7,273049587	6,857980995	7,073284692	7,069637728	PCP023578	XM_017323332
PCP023580	8,862110391	8,73470962	8,652235507	9,054387253		
PCP023593	7,520972191	6,894817763	7,011227255	6,369291982	PCP023593	XM_009362005
PCP023594	6,785681319	6,705010253	6,845490051	7,315874125	PCP023594	XM_009362007
PCP023596	6,988684687	7,251056285	7	6,981053471	PCP023596	XM_009362000
PCP023601	7,055282436	7,129283017	7,360452072	7,839203788	PCP023601	XR_001786932
PCP023604	7,406587689	7,491853096	8,462175047	10,01495499	PCP023604	XM_009351133
PCP023616	6,727920455	7,026025399	7,462134139	8,24474438	PCP023616	XM_009378031
PCP023619	7,026025399	6,965784285	7,209453366	7,648681141	PCP023619	XM_009342538
PCP023629	7,186559982	7,354646096	7,417852515	7,860031646		
PCP023641	8,122413888	8,279239123	7,948367232	10,44639065	PCP023641	NM_001302329
PCP023646	7,8008999	7,761551232	7,996219247	8,832890014	PCP023646	XM_009345584
PCP023659	8,199672345	8,163247124	8,269921462	8,711942238		
PCP023663	8,246360579	8,437419184	8,537878433	8,793343194		
PCP023685	7,491853096	7,544346278	7,674757228	8,051643995		
PCP023695	7,392317423	7,61227877	7,908872939	8,62935662	PCP023695	XM_008347948
PCP023696	6,918863237	6,894817763	7,333870928	7,787902559		
PCP023705	7,129283017	7,429030064	7,686500527	7,952275317		
PCP023707	8,510447709	8,75598914	8,685344507	8,6794801	PCP023707	XM_009378089
PCP023724	8,087462841	8,105018005	8,139551352	7,928784831	PCP023724	XM_009376639
PCP023734	7,626950122	7,169925001	7,357552005	7,156538193		
PCP023735	8,485145023	8,376472723	8,420676082	7,916894583	PCP023735	XM_018647713
PCP023767	8,515699838	8,466260038	8,726797165	8,971543554	PCP023767	XR_001951388
PCP023776	8,188193194	8,474395481	8,830768706	9,303027245	PCP023776	XM_009363905
PCP023790	7,862141394	7,924812504	7,956114749	7,324900589	PCP023790	XM_009339479
PCP023792	7,058857621	7,294620749	7,622051819	7,922851957	PCP023792	XM_009375072
PCP023798	7,667679281	7,693486957	7,691185174	7,429030064	PCP023798	XM_009374047
PCP023831	7,739240553	7,910912508	8,3994702	8,946409212	PCP023831	XM_009350412
PCP023868	8,038918989	7,882643049	7,857980995	7,395148508	PCP023868	XM_018652464
PCP023878	9	8,35169016	8,148069774	7,489205728	PCP023878	XM_009364277
PCP023940	8,058911723	8,28077077	8,478446064	8,873444113		
PCP023995	6,599912842	6,80309785	6,672425342	7,624466495	PCP023995	XM_008394899
PCP024122	8,28077077	8,161535025	8,549476655	8,285402219		
PCP024127	6,768184325	7,101503009	7,294620749	7,549438149	PCP024127	XM_009368683
PCP024149	6,528727582	6,828707735	7,037052702	8,417852515	PCP024149	XM_009345018
PCP024153	7,515699838	7,619633096	7,318949464	7,094552786	PCP024153	XM_009345007
PCP024169	7,624466495	7,718841075	7,896816753	8,232037117	PCP024169	XM_008370393
PCP024179	9,382991408	9,164906927	8,154818109	8,089212109	PCP024179	XM_009350122
PCP024198	7,360452072	7,707359132	8,344295908	9,137836494	PCP024198	XM_009344489
PCP024201	8,018645301	8,21757002	8,45532722	8,853839746		
PCP024206	8,806291831	8,591223118	8,105018005	7,796558821	PCP024206	XM_008391556
PCP024208	8,268331458	8,442943496	8,339850003	8,338379842	PCP024208	XM_009360998
PCP024211	6,898812977	7,069637728	7,055282436	8,136119594	PCP024211	XM_008391544
PCP024244	8,048050866	8,366322214	8,74929961	9,132705355	PCP024244	XM_008391507
PCP024269	7,42063398	7,725673	7,906890596	8,519636253	PCP024269	XM_008388390
PCP024279	7,910912508	7,826548487	7,572359168	8,051643995	PCP024279	XM_009348831

PCP024282	7,473056289	7,730164413	8,471675214	8,552015799	PCP024282	XM_009348836
PCP024289	9,014941046	8,686500527	8,559682997	8,654636029		
PCP024290	8,363390129	8,463524373	8,291539098	8,338379842		
PCP024313	6,922792504	6,97349648	7,115511897	7,557119022	PCP024313	XM_009374807
PCP024319	7,473056289	7,50779464	7,732472726	6,934634441	PCP024319	XM_009374825
PCP024336	7,241554209	7,254272787	7,22881869	7,772611496	PCP024336	XM_018648433
PCP024347	7,63175911	8,43325159	8,171577143	8,46760555	PCP024347	XM_008365968
PCP024348	7,499845887	7,779194046	8,058911723	8,412231097	PCP024348	XM_009362584
PCP024357	8,438791853	8,727920455	8,77478706	9,059804103		
PCP024376	7,459431619	7,757089938	7,582480735	7,456724026	PCP024376	XM_018647613
PCP024407	7,199672345	7,473056289	8,020535537	8,874458871	PCP024407	XM_018644175
PCP024414	8,714245518	8,742578916	8,926800034	8,77478706		
PCP024427	7,681800619	7,377904593	7,321928095	7,087462841		
PCP024430	8,630558365	8,545620932	8,864186145	9,252665432	PCP024430	XR_525001
PCP024438	8,430452552	8,385129006	8,515699838	8,843387202	PCP024438	XM_008372468
PCP024439	6,686500527	7,026025399	7,235248379	7,464913269	PCP024439	XM_009377120
PCP024457	7,456724026	7,132679654	7,076815597	6,841344192	PCP024457	XM_009376793
PCP024474	7,21916852	7,125878364	7,173227395	7,693486957	PCP024474	XM_018644751
PCP024476	6,528727582	6,790120385	7,058857621	7,380850638	PCP024476	XM_009349710
PCP024501	8,101555535	8,227230852	8,32791187	8,698114274	PCP024501	XM_009367256
PCP024519	8,522267655	8,330916878	8,540438054	9,234410306		
PCP024529	7,709635275	7,557119022	7,982993575	8,137862104	PCP024529	XM_009367308
PCP024547	7,589988142	7,730164413	7,946438456	8,792237182	PCP024547	XM_018651471
PCP024565	7,607330314	7,574934058	7,670089681	7,269874722	PCP024565	XM_009337533
PCP024577	8,550746785	8,60855054	8,67479253	9,075024475		
PCP024583	6,988684687	7,531381461	7,244791942	8,268331458	PCP024583	XM_009348794
PCP024586	7,363433935	7,599912842	7,670089681	7,360452072	PCP024586	XM_008343333
PCP024591	7,369204723	7,866228001	7,851749041	9,120678526		
PCP024611	7,677155841	7,153095972	7,42063398	7,108524457	PCP024611	XM_009367820
PCP024642	6,87036472	6,807354922	6,95419631	8,429071922	PCP024642	XM_009369446
PCP024649	7,765998774	7,577428828	7,515699838	7,28845085	PCP024649	XM_009369459
PCP024658	6,988684687	6,790120385	6,910852562	7,473056289	PCP024658	XM_008394211
PCP024661	7,809478757	8,003770871	7,998139076	7,839203788	PCP024661	XM_009354108
PCP024668	7,494495616	7,552054236	7,351645995	7,199672345	PCP024668	XM_008394227
PCP024709	8,41502341	8,382278171	8,474395481	8,901862467	PCP024709	XM_008364662
PCP024725	7,309885571	7,163196797	7,412188747	7,153095972	PCP024725	XM_018644706
PCP024727	7,132679654	7,489205728	7,481153605	7,3458044	PCP024727	XM_008387892
PCP024729	7,862141394	7,952275317	8,064311643	8,357552005		
PCP024731	6,403778984	6,832890014	6,981053471	7,309885571		
PCP024733	7,969645536	7,754887502	7,998139076	8,341318667		
PCP024734	6,709704193	7,014913158	7,115511897	7,54689446		
PCP024747	7,930737338	7,963878669	8,044394119	7,658211483		
PCP024748	7,818070833	8,018645301	8,22881869	7,922851957		
PCP024766	7,383704292	7,431873638	7,963878669	7,709635275	PCP024766	XM_009369796
PCP024769	8,227230852	8,434628228	8,04072808	7,380850638		
PCP024781	7,809478757	8,053491515	8,269921462	8,459431619		

PCP024803	7,918863237	8,075051225	7,721099189	7,297649983	PCP024803	XM_009372735
PCP024807	8,877529547	8,882643049	8,851749041	8,649866903	PCP024807	XM_009372801
PCP024818	8,503825738	8,478446064	8,398016818	8,32494558	PCP024818	XM_009369043
PCP024830	7,743689989	7,321928095	7,62935662	7,518299077	PCP024830	XR_667089
PCP024846	7,8008999	7,639015048	7,736943052	7,42063398	PCP024846	XM_009361168
PCP024890	7,111970261	7,22881869	6,882643049	6,745909573		
PCP024892	7,212666605	6,894817763	7,464913269	7,132679654	PCP024892	XM_009338347
PCP024903	8,357552005	8,22881869	8,279239123	8,232037117	PCP024903	XM_008390090
PCP024912	7,42063398	7,518299077	7,597456684	8,201290788		
PCP024919	7,417852515	7,562242424	7,321928095	7,315874125	PCP024919	XM_009374699
PCP024921	7,822284016	7,8008999	7,839203788	8,302273349		
PCP024930	7,336908182	7,526147081	7,803033252	7,736943052	PCP024930	XM_009343459
PCP024950	8,113742166	8,142974967	8,332394659	8,754887502		
PCP024953	6,97349648	7,333870928	7,28845085	6,918863237	PCP024953	XM_008346240
PCP024961	6,984931073	7,08395793	6,946379968	6,72342204	PCP024961	XM_018649133
PCP024964	7,080337882	7,300764373	7,166615031	7,06608919		
PCP024980	8,136119594	8,282300792	8,382278171	8,176572111	PCP024980	XM_009353895
PCP024990	8,124121312	8,279239123	8,163247124	8,075051225		
PCP025012	8,022367813	8,144658243	8,189824559	8,001858526	PCP025012	XM_017330361
PCP025019	8,416459769	8,490530019	8,903881846	9,451891079		
PCP025058	6,874428132	7,058857621	7,146390476	7,636624621	PCP025058	XM_008375047
PCP025060	7,297649983	7,386552314	7,406587689	7,225641265	PCP025060	XM_009369139
PCP025066	8,442943496	8,619596417	8,743723645	9,138706975	PCP025066	XM_009342162
PCP025077	8,073231127	8,21916852	8,356099782	7,973439079	PCP025077	XM_009381326
PCP025090	7,973439079	7,857980995	7,924812504	7,375039431	PCP025090	XM_021968721
PCP025097	7,860031646	7,339850003	6,965784285	6,420718183	PCP025097	XM_009377199
PCP025120	7,794415866	7,930737338	7,934693407	7,779194046	PCP025120	XM_009361756
PCP025128	7,051698368	6,686500527	6,938638658	7,451211112	PCP025128	XM_009362620
PCP025130	8,069691427	7,950293432	8,181549844	8,602401945	PCP025130	XM_009362650
PCP025146	7,582480735	7,429030064	7,351645995	7,186559982		
PCP025152	8,129283017	8,130982335	8,078577814	7,952275317	PCP025152	XM_009350117
PCP025164	7,984874125	8,09976859	8,171577143	8,567309664	PCP025164	XM_009371543
PCP025177	8,930737338	8,75043902	8,77807713	8,8008999	PCP025177	XM_009379237
PCP025179	8,526186214	8,42488011	8,693486957	9,014941046	PCP025179	XM_009379244
PCP025181	7,146390476	7,705079392	7,815895594	8,254225537	PCP025181	XM_009379249
PCP025182	8,033423002	8,269921462	8,369248353	8,619596417	PCP025182	XM_009379246
PCP025193	7,77036657	7,440204752	8,042589623	8,022367813		
PCP025196	6,794415866	6,686500527	7,026025399	7,594921715	PCP025196	XM_008370683
PCP025202	6,750472519	6,906890596	7,026025399	7,564759219	PCP025202	XM_008388389
PCP025203	7,6794801	7,641473777	7,614709844	8,105018005		
PCP025211	7,291585141	7,714245518	7,080337882	5,77267747	PCP025211	XM_009365744
PCP025218	8,43325159	8,296136161	8,153146652	8,21916852	PCP025218	XM_009365737
PCP025228	6,890811455	6,68187088	7,029784146	7,46760555	PCP025228	XM_009361467
PCP025233	7,956114749	7,988684687	9,039823818	10,22239645	PCP025233	XM_018646238
PCP025248	7,83080039	8,112022407	8,115459877	8,440163216		
PCP025256	8,029728942	7,536596918	8,049848549	7,776959347	PCP025256	XM_009339472

PCP025262	8,276124405	8,144658243	8,24474438	8,83709175		
PCP025283	8,956143797	8,67479253	8,915879379	8,430452552	PCP025283	XM_018649958
PCP025284	7,898873426	7,707359132	7,709635275	8,578674597	PCP025284	XM_009369850
PCP025291	8,300718622	8,329437582	8,247927513	8,022367813	PCP025291	XM_009352059
PCP025294	7,451211112	7,984874125	8,503825738	9,095397023	PCP025294	XM_009361280
PCP025312	7,574934058	8,04072808	8,407989993	9	PCP025312	XM_009346019
PCP025317	7,996219247	7,92677039	8,50779464	9,056177063	PCP025317	XM_009357989
PCP025320	6,853870927	7,111970261	7,572359168	8,239980333	PCP025320	XM_008350125
PCP025322	7,276124405	6,906890596	7,318949464	7,658211483		
PCP025324	6,50779464	6,750472519	7,142923928	7,619633096	PCP025324	XM_009335969
PCP025330	8,826548487	8,73470962	8,69115027	8,693486957		
PCP025332	8,277659361	8,293103743	8,357552005	8,744833837	PCP025332	XM_009373942
PCP025382	7,884658968	8,178266468	8,262094845	8,710806434		
PCP025396	7,634230226	7,646234675	7,64385619	8,176572111	PCP025396	XM_009366443
PCP025499	7,796558821	7,54689446	7,796558821	7,333870928		
PCP025639	6,942514505	7,273049587	6,969587981	6,898812977	PCP025639	XM_008347713
PCP025691	7,798763389	7,619633096	7,750405521	7,491853096	PCP025691	XM_008377346
PCP025702	7	7,108524457	7,139551352	7,698149009	PCP025702	XM_008375909
PCP025711	8,28077077	8,283875484	7,497213158	6,369291982	PCP025711	XM_009347652
PCP025733	7,942514505	8,020535537	7,990557838	8,514398461	PCP025733	XM_008352293
PCP025740	6,648609245	7,122362117	6,984931073	7,369204723	PCP025740	XM_009335961
PCP025743	7,269874722	7,572359168	7,549438149	7,395148508	PCP025743	XM_009335993
PCP025746	7,300764373	7,74819285	7,805227956	8,033423002	PCP025746	XM_009336002
PCP025760	8,098032083	7,952275317	7,942514505	8,353146825	PCP025760	XM_009336177
PCP025765	7,196430001	6,60481265	7,044394119	6,853870927	PCP025765	XM_009336248
PCP025768	7,073284692	7,46760555	7,291585141	7,727920455	PCP025768	XM_009336279
PCP025770	6,609843592	6,700439718	6,958030641	7,276124405	PCP025770	XM_008378996
PCP025779	8,156487632	8,19313106	8,361943774	8,779161208	PCP025779	XM_009336441
PCP025790	8,232037117	8,069691427	8,016808288	8,060695932		
PCP025797	8,456682963	8,260543232	8,169925001	8,141238626	PCP025797	XM_009336631
PCP025809	7,136170875	6,930737338	7,266786541	7,688809791		
PCP025810	8,671257984	9,079484784	8,976334992	8,583722152	PCP025810	XM_009336810
PCP025814	8,830768706	8,533991546	8,544307635	8,356099782		
PCP025819	7,888743249	8,235200503	8,24474438	7,839203788	PCP025819	XM_009370383
PCP025820	7,818070833	7,768184325	7,702726796	8,186510462	PCP025820	XM_009370382
PCP025822	7,238404739	7,189824559	7,291585141	7,928784831	PCP025822	XM_009370380
PCP025830	6,333960351	6,80309785	6,938638658	7,868328714	PCP025830	XM_008378510
PCP025846	8,803065551	8,386595423	8,614709844	8,668884984	PCP025846	XM_009339337
PCP025850	8,09976859	8,230404783	8,405141463	8,80196697	PCP025850	XM_009339330
PCP025854	7,828644274	7,658211483	7,587440004	7,354646096	PCP025854	XM_009339322
PCP025878	8,207844058	8,212715235	8,251103639	8,693486957	PCP025878	XM_009338671
PCP025892	7,882643049	8,252665432	8,744833837	9,6171552	PCP025892	XM_009338686
PCP025893	8,251103639	8,73470962	8,916864735	9,218369492		
PCP025896	8,423452115	8,459431619	8,568602197	9,00750388		
PCP025897	8,687655621	8,523561956	8,764871591	9,099794885	PCP025897	XM_008390313
PCP025916	8,146339557	8,142974967	7,619633096	8,051643995		

PCP025933	7,853808564	7,820178962	7,944448531	7,700439718	PCP025933	XM_009375019
PCP025943	8,35169016	7,912889336	8,13442632	7,906890596	PCP025943	XM_009364733
PCP025952	8,127581695	7,876516947	7,982993575	8,035238993	PCP025952	XM_018648871
PCP025954	8,356099782	8,236827423	8,146339557	7,798763389	PCP025954	XM_009364706
PCP025968	6,934634441	6,922792504	6,853870927	7,491853096	PCP025968	XM_008361864
PCP025978	7,599912842	7,847558521	8,06786455	8,011227255	PCP025978	XM_008352669
PCP025979	8,06608919	7,510408147	7,790055203	7,813781191	PCP025979	XM_009358118
PCP025980	8,315919398	8,136119594	8,048050866	7,961912672		
PCP025993	7,515699838	7,688809791	7,691185174	8,08571145		
PCP026009	7,892816	7,531381461	7,536596918	7,462134139	PCP026009	XM_009371507
PCP026014	8,291539098	8,726797165	8,900866808	9,108524457	PCP026014	XM_009371366
PCP026017	7,672425342	8,141238626	8,148069774	7,884658968	PCP026017	XM_009371345
PCP026020	6,163297449	6,878602742	7,360452072	8,54303182	PCP026020	XM_009371311
PCP026030	9,341340908	8,277659361	8,606109055	7,723353774		
PCP026036	8,475733431	8,67479253	8,636624621	8,501160452	PCP026036	XM_009362927
PCP026038	7,594921715	7,64385619	8,038918989	8,339850003	PCP026038	XM_009362913
PCP026046	7,209453366	7,300764373	7,389480771	7,884658968	PCP026046	XM_009354723
PCP026048	8,390899794	8,402287298	8,464872438	8,258942852	PCP026048	XM_009354729
PCP026052	8,703903573	8,678318438	8,694636474	8,510447709		
PCP026053	8,283875484	8,30833903	8,402287298	8,850718177		
PCP026069	7,400879436	7,212666605	7,330916878	7,173227395	PCP026069	XM_009340434
PCP026073	6,922792504	7,336908182	7,297649983	7,672425342	PCP026073	XM_009340388
PCP026088	7,928784831	7,592457037	7,531381461	7,930737338	PCP026088	XM_018643318
PCP026090	7,820178962	7,732472726	7,994353437	7,693486957	PCP026090	XM_008395005
PCP026093	8,379378367	8,528766645	8,665335917	9,114601277	PCP026093	XM_009340037
PCP026099	7,88062431	7,934693407	8,232037117	8,479780264		
PCP026101	7,040782866	7,003714662	7,225641265	7,910912508	PCP026101	XM_009339943
PCP026128	8,09976859	7,750405521	7,809478757	8,142974967		
PCP026130	7,579919292	7,544346278	7,759355602	7,528805707	PCP026130	XR_526409
PCP026141	7,847558521	7,805227956	8,427648072	8,035238993		
PCP026145	7,938579853	7,727920455	7,266786541	7,225641265	PCP026145	XM_008354771
PCP026155	7,513095909	7,491853096	7,815895594	8,044394119		
PCP026161	8,178266468	8,41502341	8,341318667	8,356099782	PCP026161	XM_017335003
PCP026178	7,291585141	7,415065677	7,453929061	8,062477937	PCP026178	XM_009354455
PCP026183	7,639015048	7,486473046	7,456724026	7,366322214	PCP026183	XM_009367456
PCP026199	8,330916878	8,222408523	8,265239967	8,815927606		
PCP026216	9,399448837	8,559682997	8,596189756	7,994353437	PCP026216	XM_009347771
PCP026221	8,029728942	7,973439079	7,936637939	7,87036472	PCP026221	XM_009347781
PCP026227	7,044394119	7,375039431	7,607330314	8,176572111	PCP026227	XM_009347794
PCP026233	7,212666605	7,684187561	7,74819285	7,965784285	PCP026233	XM_009370883
PCP026244	6,559644763	6,619559738	6,984931073	8,651051691	PCP026244	XM_018644663
PCP026252	8,285402219	7,752681699	8,042589623	7,843418611	PCP026252	XM_009363249
PCP026265	8,819125283	9,049848549	8,864186145	8,540438054	PCP026265	XM_009364462
PCP026281	6,569855608	7,142923928	7,851749041	8,430452552	PCP026281	XM_009364435
PCP026284	7,843418611	7,763743526	7,874489611	8,300718622	PCP026284	XM_009364430
PCP026298	7,225641265	6,667750232	7,202907418	7,159871337	PCP026298	XM_009360287

PCP026302	8,206184105	8,326429487	8,17990909	8,161535025	PCP026302	XM_009342383
PCP026315	8,08390476	7,725673	7,906890596	7,730164413	PCP026315	XM_018644510
PCP026322	8,243173983	8,380807357	8,437419184	8,273002948	PCP026322	XM_018644533
PCP026332	8,108524457	7,960001932	7,834976616	7,554588852		
PCP026356	8,132731056	7,851749041	8,344295908	8,348728154	PCP026356	XM_020568187
PCP026369	7,557119022	7,619633096	7,906890596	8,268331458	PCP026369	XM_009359990
PCP026376	7,624466495	7,50247382	8,141238626	9,131856961		
PCP026381	8,206184105	8,274587815	8,252665432	8,792237182		
PCP026384	8,545620932	8,75598914	8,528766645	8,555854491		
PCP026398	6,609843592	6,714245518	6,965784285	7,702726796		
PCP026408	6,745909573	6,902917719	7,173227395	7,672425342	PCP026408	XM_009380081
PCP026420	7,589988142	7,765998774	8,051643995	8,598685286	PCP026420	XM_009360775
PCP026425	8,589950702	8,813781191	8,709669735	8,526186214		
PCP026441	8,252665432	8,146339557	8,199672345	8,141238626		
PCP026487	7,658211483	7,354646096	7,878541438	7,087462841		
PCP026490	8,279239123	8,303780748	8,479780264	8,28692734	PCP026490	XM_009338996
PCP026499	8,037107627	8,169925001	8,62935662	9,08833774	PCP026499	XM_009345553
PCP026524	6,807354922	6,523561956	6,882643049	7,434628228	PCP026524	XM_009344141
PCP026528	7,904905525	8,075051225	8,196380818	7,837123296	PCP026528	XM_018647856
PCP026541	7,483815777	7,567347696	7,462134139	7,415065677		
PCP026542	7,309885571	7,291585141	7,412188747	7,146390476		
PCP026557	8,518338423	8,255831156	8,497173232	8,39373366	PCP026557	XM_009367697
PCP026561	7,321928095	7,196430001	7,196430001	7,022367813	PCP026561	XM_009367654
PCP026577	7,634230226	7,584962501	7,087462841	7,044394119	PCP026577	XM_009354030
PCP026582	7,040782866	6,86628983	7,209453366	6,837060204	PCP026582	XM_009356480
PCP026593	7,125878364	7,574934058	7,215969746	7,094552786	PCP026593	XM_009375515
PCP026597	8,407989993	8,567309664	8,530094158	7,961912672		
PCP026618	8,268331458	8,792237182	8,739206792	8,660602089		
PCP026634	7,77036657	8,007475849	8,136119594	8,544307635	PCP026634	NM_001294081
PCP026637	7,868328714	7,651051691	8,014968933	8,74708524	PCP026637	XM_017325744
PCP026645	7,209453366	7,166615031	7,21916852	7,721099189	PCP026645	XM_008391620
PCP026648	8,462175047	8,703903573	8,685344507	8,672425342	PCP026648	XM_008352732
PCP026653	8,3994702	8,447083226	8,584962501	8,920858975	PCP026653	XM_018652075
PCP026660	7,783522135	7,515699838	7,357552005	7,790055203	PCP026660	XM_008380061
PCP026665	7,862141394	7,898873426	7,700439718	7,406587689	PCP026665	XM_008371444
PCP026668	7,254272787	7,520972191	7,567347696	8,026080745	PCP026668	XM_009361614
PCP026672	7,531381461	7,398059585	7,691185174	6,996275749	PCP026672	XM_018644906
PCP026712	8,076815597	7,730164413	7,853808564	7,924812504	PCP026712	XM_009362343
PCP026721	6,837060204	7,339850003	7,30679083	7,222360218	PCP026721	XM_008369875
PCP026723	7,244791942	7,159871337	7,17990909	6,84962403		
PCP026725	7,837123296	7,745976779	8,005624549	8,447083226		
PCP026731	8,949330653	8,668884984	8,540438054	8,825499451	PCP026731	XM_009339769
PCP026735	8,607330314	8,781359714	8,715378619	8,767091963	PCP026735	XM_009339760
PCP026740	7,83080039	8,098032083	8,120652609	8,438791853	PCP026740	XM_018652192
PCP026752	6,781359714	7,08395793	7,14974712	7,459431619	PCP026752	XM_009343420
PCP026753	8,033423002	8,106798463	8,19313106	7,750405521	PCP026753	XM_009343421

PCP026764	7,658211483	7,745976779	7,960001932	8,232037117		
PCP026765	7,06608919	7,333870928	7,266786541	7,142923928		
PCP026787	7,222360218	7,366322214	6,965784285	6,700439718	PCP026787	XM_009370481
PCP026789	8,62935662	7,950293432	7,92677039	9,107661718	PCP026789	XM_018650121
PCP026796	7,779194046	8,113742166	7,944448531	7,853808564		
PCP026826	8,21916852	8,096293483	8,254225537	8,838163917		
PCP026834	7,672425342	8,024197765	8,426264755	7,739240553		
PCP026855	6,820178962	7,098032083	7,125878364	7,841281284	PCP026855	XM_008388884
PCP026874	7,569855608	7,354646096	7,721099189	7,906890596	PCP026874	XM_008345943
PCP026875	8,069691427	7,658211483	7,336908182	7,087462841	PCP026875	XM_018645978
PCP026882	7,648681141	7,853808564	7,74819285	7,486473046	PCP026882	XM_009347695
PCP026930	7,741466986	7,567347696	7,392317423	6,946379968	PCP026930	XM_018645929
PCP026959	8,741466986	8,76927586	8,520932916	8,628153873	PCP026959	XM_008349733
PCP026967	8,715378619	8,818038868	8,795487741	8,60855054	PCP026967	XM_009336437
PCP026975	8,366322214	8,32494558	8,306836389	8,176572111	PCP026975	XM_008346333
PCP026980	7,862141394	7,641473777	8,115459877	8,895817605	PCP026980	XM_009367201
PCP026981	8,141238626	8,076815597	8,06786455	8,001858526	PCP026981	XM_009343279
PCP026994	8,434628228	8,483815777	8,531381461	8,419243918	PCP026994	XM_009356779
PCP027011	6,950351762	7,269874722	7,294620749	7,577428828	PCP027011	XM_018649448
PCP027017	8,505136683	8,073231127	8,30833903	7,434628228	PCP027017	XM_017335200
PCP027050	7,754887502	7,807354922	8,048050866	8,406545173	PCP027050	XM_009360325
PCP027057	7,189824559	7,431873638	7,486473046	7,920829209		
PCP027097	6,653490009	6,709704193	7,037052702	7,559721231	PCP027097	XM_017333902
PCP027118	7,497213158	7,423494135	7,614709844	8,161535025	PCP027118	XM_008367397
PCP027180	7,297649983	7,360452072	7,336908182	6,950351762	PCP027180	XM_009374178
PCP027186	7,882643049	7,759355602	7,639015048	7,062531903		
PCP027208	7,129283017	7,06608919	7,090959258	7,723353774		
PCP027241	8,252665432	8,207844058	8,027905997	8,06786455	PCP027241	XM_018646285
PCP027251	7,139551352	6,841344192	7,14974712	6,824386003	PCP027251	XM_009346734
PCP027304	7,321928095	6,81159947	6,84962403	7,297649983	PCP027304	XM_009344811
PCP027309	6,718772592	6,785681319	6,981053471	7,536596918	PCP027309	XM_009343434
PCP027315	7,051698368	6,828707735	7,159871337	7,515699838	PCP027315	XM_018644118
PCP027318	7,533952624	7,604886762	7,8008999	8,413627929	PCP027318	XM_009343447
PCP027333	8,815927606	8,693486957	8,677120596	8,517000043	PCP027333	XM_009346814
PCP027334	7,440204752	7,312882955	7,61227877	7,872397856	PCP027334	XR_527841
PCP027359	8,395191361	8,04072808	8,048050866	7,982993575	PCP027359	XM_009339906
PCP027361	7,990557838	8	8,082149041	8,478446064	PCP027361	XM_009339907
PCP027368	7,163196797	7,342785837	6,741466986	6,759289016	PCP027368	XM_017331705
PCP027382	8,241601875	8,305286574	8,456682963	8,274587815	PCP027382	XM_018643332
PCP027391	7,92677039	8,046196362	8,449850215	8,21916852	PCP027391	XM_008372189
PCP027394	8,266786541	8,354602022	8,184875343	8,215921225	PCP027394	XM_009339956
PCP027396	8,227230852	8,163247124	8,273002948	8,80841723	PCP027396	XM_009339953
PCP027399	7,423494135	7,851749041	7,781359714	7,996219247		
PCP027403	8,309840108	8,367764188	8,214319121	8,246360579	PCP027403	XM_009378339
PCP027408	6,554588852	6,894817763	7,055282436	7,736943052		
PCP027426	7,811663685	7,839203788	7,843418611	7,641473777		

PCP027436	8,037107627	8,115459877	8,376472723	8,654636029	PCP027436	XM_009370446
PCP027448	8,118941073	8,080391184	7,695785075	7,658211483	PCP027448	XM_009370436
PCP027460	7,655852677	7,665335917	7,693486957	8,117227504	PCP027460	XM_009370421
PCP027462	6,902917719	7,033423002	7,315874125	7,794415866	PCP027462	XM_009370417
PCP027477	7,986809101	7,783522135	7,809478757	7,62935662	PCP027477	XM_009359046
PCP027497	7,139551352	7,483815777	7,700439718	8,274587815	PCP027497	XM_009342191
PCP027503	8,246360579	8,21916852	8,383704292	8,703903573		
PCP027510	7,115511897	7,037052702	7,206232954	7,61227877		
PCP027532	7,470292816	7,641473777	7,928784831	8,572397068	PCP027532	XM_009345409
PCP027534	7,440204752	8,016808288	8,269921462	9,932701967	PCP027534	XR_532260
PCP027535	8,63542792	8,555854491	8,830768706	8,6794801	PCP027535	XM_017323388
PCP027546	7,544346278	6,906890596	7,073284692	6,363346321	PCP027546	XM_009381246
PCP027551	6,750472519	6,934634441	7,136170875	7,380850638	PCP027551	XM_009381256
PCP027554	8,511752654	8,60855054	8,494535617	8,176572111		
PCP027555	8,772578508	8,509101985	8,767091963	8,523561956		
PCP027566	7,342785837	7,459431619	7,686500527	8,064311643	PCP027566	XM_009342232
PCP027576	7,754887502	7,87036472	7,651051691	7,539158811		
PCP027583	7,855927425	7,996219247	8,230404783	8,673591756	PCP027583	XM_009342375
PCP027591	8,048050866	8,069691427	8,037107627	7,963878669		
PCP027594	7,973439079	7,448488033	7,17990909	6,845490051	PCP027594	XM_009346491
PCP027606	7,21916852	7,614709844	7,781359714	7,986809101	PCP027606	XM_009370620
PCP027609	7,212666605	7,076815597	7,136170875	7,711976642	PCP027609	XM_009370617
PCP027612	7,330916878	7,944448531	7,928784831	7,857980995	PCP027612	XM_008345263
PCP027614	8,222408523	8,148069774	8,206184105	8,113742166	PCP027614	XM_009355823
PCP027618	8,560982378	8,661778098	8,772578508	9,108524457		
PCP027620	7,531381461	7,318949464	7,163196797	7,622051819	PCP027620	XM_009355905
PCP027625	8,37794796	8,146339557	8,101555535	8,506486109	PCP027625	XM_008357950
PCP027631	8,094500005	8,37794796	8,557157324	8,874458871	PCP027631	XM_018647071
PCP027651	7,552054236	7,22881869	7,169925001	7,634230226	PCP027651	XM_008386575
PCP027662	7,912889336	7,839203788	7,936637939	8,338379842	PCP027662	XM_009373230
PCP027675	6,832890014	7,241554209	7,44567705	7,597456684	PCP027675	XM_009349596
PCP027678	8,262094845	8,482485305	8,514398461	8,426264755	PCP027678	XM_009349603
PCP027693	7,494495616	7,619633096	7,956114749	8,238404739		
PCP027695	8,70276142	8,531381461	8,50779464	8,249492747	PCP027695	XM_008363584
PCP027719	8,470333494	8,293103743	8,594959026	8,957102042	PCP027719	XM_009373080
PCP027734	6,857980995	6,841344192	6,824386003	7,409390936	PCP027734	XM_008367714
PCP027768	7,969645536	7,64385619	7,74819285	7,624466495	PCP027768	XM_009366565
PCP027772	7,303780748	7,022367813	7,294620749	8,388017285	PCP027772	XM_009361334
PCP027773	8,623259662	8,519636253	8,607330314	9,139551352	PCP027773	XM_009361331
PCP027791	7,609769734	7,805227956	7,944448531	8,481113232	PCP027791	XM_009338828
PCP027820	7,589988142	7,526147081	7,820178962	8,348728154	PCP027820	XM_009337371
PCP027821	7,022367813	6,80309785	6,794415866	7,667679281	PCP027821	XR_001789422
PCP027834	8,037107627	7,828644274	7,930737338	7,813781191		
PCP027838	7,674757228	7,928784831	8,120652609	8,471675214	PCP027838	XM_018646291
PCP027854	8,490530019	8,696967526	8,797661526	9,024225474	PCP027854	XM_009348391
PCP027870	8,613494819	8,579956993	8,725638921	8,540438054	PCP027870	XM_008342648

PCP027884	8,101555535	8,031604721	8,230404783	8,812722827		
PCP027895	7,510408147	8,137862104	8,057071136	7,820178962		
PCP027898	8,120652609	8,17990909	7,906890596	7,815895594	PCP027898	XM_009350611
PCP027912	7,375039431	7,369204723	7,74819285	7,494495616	PCP027912	XM_008380127
PCP027937	8,014968933	8,176572111	8,291539098	7,619633096	PCP027937	XM_009374692
PCP027949	8,156487632	8,024197765	8,171577143	7,828644274		
PCP027955	7,979167931	7,884658968	7,98111057	8,483815777	PCP027955	XM_009336263
PCP027961	8,653454181	8,318904285	8,509101985	8,603626345		
PCP027970	7,139551352	7,209453366	7,434628228	7,761551232	PCP027970	XM_009363025
PCP027972	8,445718428	8,146339557	8,366322214	7,956114749	PCP027972	XM_009363019
PCP027977	7,398059585	7,579919292	7,62935662	8,148069774	PCP027977	XM_009363013
PCP027978	8,103287808	8,209453366	8,186510462	8,791162889		
PCP027983	7,375039431	7,318949464	7,327956767	7,930737338	PCP027983	XM_009363002
PCP027995	7,597456684	7,604886762	7,824449651	7,533952624	PCP027995	XM_009356162
PCP027999	6,513016928	6,705010253	7,090959258	7,475733431		
PCP028009	7,958088658	8,458078458	9,709083813	9,108524457	PCP028009	NM_001294363
PCP028015	7,186559982	6,965784285	7,330916878	7,602364826		
PCP028016	7,8008999	7,707359132	7,745976779	7,442943496		
PCP028026	7,478405614	7,494495616	7,718841075	8,103287808	PCP028026	XM_008342555
PCP028034	7,54689446	7,796558821	7,868328714	8,266786541	PCP028034	XM_018646861
PCP028035	8,199672345	7,928784831	7,860031646	8,026080745	PCP028035	XM_009355286
PCP028043	8,326429487	8,48651327	8,614709844	8,380807357		
PCP028045	7,691185174	7,489205728	7,406587689	7,528805707		
PCP028054	8,241601875	8,344295908	8,625708843	8,653454181		
PCP028057	7,473056289	7,132679654	7,033423002	6,824386003	PCP028057	XM_018645837
PCP028062	7,003714662	6,845490051	7,222360218	7,853808564	PCP028062	XM_009350749
PCP028073	8,50779464	8,103287808	8,108524457	8,075051225		
PCP028076	7,569855608	7,695785075	7,765998774	8,078577814		
PCP028092	7,963878669	8,108524457	8,43325159	9,945443836	PCP028092	XM_008343259
PCP028094	7,711976642	8,013071384	8,17990909	9,009352771	PCP028094	XM_008343280
PCP028105	7,667679281	7,486473046	7,54689446	7,423494135	PCP028105	XM_009368585
PCP028106	7,772611496	7,660566438	7,686500527	7,342785837	PCP028106	XM_009368583
PCP028107	7,360452072	7,222360218	7,470292816	7,087462841	PCP028107	XM_009379424
PCP028110	8,092757141	8,501160452	8,503825738	8,866258916		
PCP028112	7,988684687	8,236827423	8,108524457	8,094500005		
PCP028118	7,54689446	7,695785075	7,589988142	7,303780748	PCP028118	XM_009368731
PCP028125	8,568602197	7,928784831	8,392317423	8,33391564	PCP028125	XM_009354274
PCP028131	8,479780264	8,511752654	8,540438054	9,057991723	PCP028131	XM_008354113
PCP028138	7,604886762	7,815895594	7,930737338	8,164906927	PCP028138	XM_009370319
PCP028156	8,67479253	8,791162889	8,603626345	9,023283079		
PCP028178	8,74929961	8,478446064	8,427648072	8,225593068	PCP028178	XM_018651619
PCP028197	8,530094158	8,220765252	8,463524373	9,011227255		
PCP028198	7,597456684	7,06608919	7,222360218	7,125878364	PCP028198	XM_008381739
PCP028205	8,053491515	8,209453366	8,183238369	8,06608919	PCP028205	XM_009341778
PCP028250	7,412188747	7,549438149	7,624466495	8,263644792		
PCP028269	8,184875343	8,21916852	8,183238369	8,044394119		

PCP028282	7,351645995	7,412188747	8,049848549	7,855927425	PCP028282	XM_009373700
PCP028300	7,754887502	7,796558821	7,884658968	8,28692734		
PCP028303	8,723387907	8,784634846	8,72904287	8,584962501	PCP028303	XM_009352619
PCP028333	8,06786455	7,579919292	7,662988741	7,022367813	PCP028333	XM_018652193
PCP028334	8,04072808	7,904905525	8,247927513	7,459431619		
PCP028335	7,528805707	7,315874125	7,552054236	7,022367813	PCP028335	XM_008369098
PCP028337	8,567309664	8,353146825	8,481113232	8,144658243	PCP028337	XM_009368426
PCP028351	8,080391184	7,403693786	7,898873426	8,440163216	PCP028351	XM_008380191
PCP028377	8,016808288	8,3994702	8,262094845	8,736976865	PCP028377	XM_017331129
PCP028380	9,06250492	8,646270682	8,21916852	7,254272787	PCP028380	XM_018644228
PCP028423	7,169925001	7,173227395	7,380850638	7,866228001	PCP028423	XM_009340749
PCP028424	6,80309785	8,353146825	8,30833903	8,212715235		
PCP028462	7,377904593	7,431873638	7,357552005	7,225641265	PCP028462	XM_009371107
PCP028464	8,238404739	8,003770871	8,042589623	7,750405521	PCP028464	XM_009371105
PCP028467	7,146390476	7,297649983	7,196430001	7,139551352	PCP028467	XM_009359594
PCP028469	8,350187812	8,113742166	8,273002948	8,062477937	PCP028469	XM_009359596
PCP028516	8,375039431	8,511752654	8,532667615	7,916894583		
PCP028529	8,577428828	8,784634846	8,601139355	7,811663685	PCP028529	XM_017324520
PCP028533	6,564835417	6,824386003	7,014913158	7,222360218	PCP028533	XM_008394290
PCP028537	8,227230852	8,513056419	8,805195705	9,261319247	PCP028537	XM_008384228
PCP028561	8,169925001	8,154818109	8,306836389	8,665335917		
PCP028581	6,981053471	7,094552786	6,938638658	6,589913261	PCP028581	XM_008348814
PCP028604	7,757089938	7,698149009	7,809478757	8,269921462	PCP028604	XM_009343095
PCP028625	8,618385502	8,634193917	8,64385619	9,093628836	PCP028625	XM_009336727
PCP028627	6,54426899	6,321928095	6,977279923	8,044394119	PCP028627	XM_009376507
PCP028647	6,50779464	6,60481265	7,007531912	7,318949464		
PCP028674	8,493174961	8,677120596	8,68183575	8,46760555	PCP028674	XM_009355812
PCP028751	7,515699838	7,321928095	7,231989133	6,988684687	PCP028751	XR_001952085
PCP028756	8,979196518	8,315919398	8,773699688	8,869347076	PCP028756	XM_009358003
PCP028833	8,499845887	8,788979283	8,584962501	8,370687407	PCP028833	XM_009373783
PCP028843	7,406587689	7,42063398	7,662988741	8,125826717	PCP028843	XR_001954346
PCP028845	7,533952624	7,677155841	8,009380767	8,277659361		
PCP028872	7,351645995	7,497213158	7,765998774	7,478405614	PCP028872	XM_009338024
PCP028883	8,103287808	8,161535025	8,293103743	8,636624621	PCP028883	XM_009371659
PCP028903	8,710806434	8,559682997	8,535275377	8,361943774	PCP028903	XM_009341601
PCP028904	6,763809907	6,677085351	6,898812977	7,44567705		
PCP028916	8,282300792	8,360495967	8,389437748	8,859006685	PCP028916	XM_008339313
PCP028917	7,400879436	7,619633096	7,847558521	8,146339557	PCP028917	XM_009372884
PCP028919	6,988684687	7,339850003	7,658211483	8,096293483	PCP028919	XM_009372878
PCP028921	8,460783512	8,246360579	8,927777962	8,725638921	PCP028921	XM_009372875
PCP028924	7,263691734	7,559721231	7,688809791	8,309840108	PCP028924	XM_008355369
PCP028925	8,522267655	8,50779464	8,603626345	9,050746552	PCP028925	NM_001328872
PCP028944	6,727920455	6,845490051	7,007531912	7,386552314	PCP028944	XM_008343938
PCP028951	7,003714662	7,183188734	7,523561956	7,63175911	PCP028951	XM_008343947
PCP028952	6,594996337	7,040782866	6,72342204	7,321928095	PCP028952	XM_008343951
PCP028955	8,588714636	8,489165579	8,599912842	9,005624549	PCP028955	XM_009366918

PCP028957	7,260496187	7,894817763	7,888743249	7,934693407	PCP028957	XM_008343960
PCP028958	7,321928095	7,363433935	7,003714662	6,594996337	PCP028958	XM_008343967
PCP028974	6,54426899	6,619559738	7,080337882	7,225641265	PCP028974	XM_009337081
PCP028980	8,382278171	8,460783512	8,594959026	8,359002767	PCP028980	XM_009337092
PCP028987	8,288496992	8,326429487	8,442943496	8,82336724	PCP028987	XM_009348257
PCP028991	8,232037117	8,274587815	8,533991546	8,770399609	PCP028991	XM_009335747
PCP029005	7,294620749	7,531381461	7,400879436	7,342785837	PCP029005	XM_009335760
PCP029007	6,777025123	7,047996356	7,375039431	7,71651083	PCP029007	XM_009335762
PCP029032	8,427648072	8,412231097	8,598685286	9,491853096	PCP029032	XM_009358529
PCP029033	7,916894583	7,725673	8,044394119	7,866228001	PCP029033	XM_009358532
PCP029034	7,251056285	7,354646096	7,426264755	7,173227395	PCP029034	XM_009358534
PCP029041	6,886672074	7,007531912	7,014913158	7,579919292		
PCP029047	7,462134139	7,711976642	8,033423002	7,772611496	PCP029047	XM_009345315
PCP029061	7,101503009	6,977279923	6,977279923	6,594996337		
PCP029080	7,967687386	8,101555535	8,481113232	7,834976616	PCP029080	XM_009364782
PCP029082	8,178266468	8,386595423	8,649866903	8,856954575		
PCP029083	7,440204752	7,339850003	7,826548487	8,148069774	PCP029083	XM_009364788
PCP029096	7,456724026	7,129283017	7,300764373	7,14974712	PCP029096	XM_009376947
PCP029103	8,071462363	8,300718622	8,356099782	8,665335917	PCP029103	XM_009377905
PCP029108	7,528805707	7,222360218	7,238404739	6,84962403		
PCP029120	8,367764188	8,276124405	8,299208018	8,224001674	PCP029120	XM_008339227
PCP029129	6,950351762	7,176621973	7,269874722	7,660566438		
PCP029166	7,094552786	7,101503009	7,166615031	6,68187088	PCP029166	XM_009354581
PCP029171	7,478405614	7,386552314	7,592457037	7,357552005		
PCP029178	7,646234675	6,857980995	7,409390936	7,354646096	PCP029178	XM_008380814
PCP029185	7,132679654	6,926829678	6,95419631	6,832890014	PCP029185	XM_009346907
PCP029202	8,648645193	8,673591756	9,038013592	8,827596761	PCP029202	XM_008380841
PCP029209	8,283875484	8,409390936	7,584962501	8,717676423	PCP029209	NM_001328782
PCP029226	6,579994693	6,996275749	6,988684687	7,28845085	PCP029226	XM_009346729
PCP029239	7,510408147	7,759355602	8,051643995	8,249492747	PCP029239	XM_008381746
PCP029251	8,277659361	8,591223118	8,742578916	8,815927606	PCP029251	XM_018649340
PCP029258	8,453970202	8,35169016	8,291539098	8,329437582	PCP029258	XM_009378955
PCP029289	8,503825738	8,692336525	8,548166865	9,14890869		
PCP029294	7,125878364	7,71651083	8,382278171	7,787902559	PCP029294	XM_008376268
PCP029306	7,398059585	7,609769734	7,655852677	8,122413888	PCP029306	XM_009348456
PCP029307	7,30679083	7,497213158	7,412188747	7,942514505		
PCP029313	7,383704292	7,459431619	7,572359168	7,930737338		
PCP029314	8,515699838	8,568602197	8,922822231	9,108524457	PCP029314	XM_017337295
PCP029322	7,536596918	6,918863237	7,670089681	8,491853096	PCP029322	XM_018646237
PCP029329	6,741466986	6,594996337	7,442943496	8,174925683	PCP029329	XM_009352584
PCP029369	8,458078458	8,262094845	8,214319121	8,279239123	PCP029369	XM_009381472
PCP029373	8,026080745	8,141238626	8,320439548	8,640244936		
PCP029383	8,429071922	8,437419184	8,939579214	9,315896762	PCP029383	XM_009336952
PCP029389	7,007531912	6,906890596	7,033423002	7,552054236	PCP029389	XM_008367725
PCP029393	8,531381461	8,518338423	8,770399609	8,632995197	PCP029393	XM_008356128
PCP029394	7,21916852	7,189824559	7,369204723	7,080337882	PCP029394	XM_009365673

PCP029439	8,342830273	8,354602022	8,293103743	8,194756854		
PCP029441	8,528766645	8,282300792	8,249492747	7,619633096	PCP029441	XR_530718
PCP029445	7,462134139	7,339850003	7,297649983	7,206232954	PCP029445	XM_008360189
PCP029456	8,578674597	8,95419631	8,382278171	8,366322214	PCP029456	XM_008363913
PCP029462	7,189824559	7,199672345	7,392317423	7,932687205	PCP029462	XM_009362701
PCP029492	7,761551232	7,727920455	7,597456684	7,241554209		
PCP029497	8,315919398	8,54303182	8,495855027	8,441574774	PCP029497	XM_009343586
PCP029508	6,54426899	7,169925001	7,523561956	8,103287808	PCP029508	XM_008371293
PCP029513	7,276124405	7,129283017	6,914923239	6,732404887	PCP029513	XM_009363843
PCP029533	6,667750232	6,961970533	6,824386003	7,732472726	PCP029533	NM_001294124
PCP029538	7,389480771	7,50779464	7,718841075	8,137862104	PCP029538	XM_008342350
PCP029539	7,624466495	7,398059585	7,587440004	7,406587689		
PCP029554	8,732438807	8,910882535	8,639051236	8,893817229	PCP029554	XM_008378636
PCP029555	8,571107931	8,68183575	8,612241904	8,660602089	PCP029555	XM_009362958
PCP029570	8,271463028	8,263644792	8,735826768	8,659389441		
PCP029571	8,206184105	7,975389442	8,136119594	8,559682997	PCP029571	XM_009366662
PCP029593	7,087462841	7,139551352	7,3458044	7,700439718	PCP029593	XM_009368099
PCP029597	8,125826717	8,06786455	8,181549844	8,003770871	PCP029597	XM_009368103
PCP029600	7,64385619	7,577428828	7,531381461	6,369291982		
PCP029617	6,777025123	7,297649983	7,209453366	7,64385619		
PCP029623	8,018645301	7,315874125	7,74819285	7,294620749	PCP029623	NM_001328983
PCP029631	7,254272787	7,156538193	7,318949464	7,750405521	PCP029631	XM_009375307
PCP029642	7,238404739	7,403693786	7,554588852	8,106798463	PCP029642	XM_009361415
PCP029644	7,934693407	7,648681141	7,434628228	7,614709844	PCP029644	XM_018648196
PCP029652	8,087462841	8,285402219	8,317412614	8,722226922	PCP029652	XR_001952641
PCP029671	6,60481265	6,260590275	7,818070833	7,263691734	PCP029671	XM_009345853
PCP029711	8,819125283	8,440163216	8,3994702	8,027905997	PCP029711	XM_009344724
PCP029712	8,14974712	7,996219247	8,020535537	8,046196362		
PCP029723	8,189824559	8,338379842	8,345760055	8,733591606	PCP029723	XM_008359170
PCP029724	8,505136683	8,306836389	8,350187812	7,90285744	PCP029724	XM_008379951
PCP029725	7,105070402	7,132679654	7,212666605	7,876516947	PCP029725	XM_008379821
PCP029760	8,243173983	8,194756854	8,407989993	8,146339557	PCP029760	XM_018651595
PCP029761	7,183188734	7,132679654	7,451211112	7,805227956	PCP029761	XM_018646338
PCP029767	7,811663685	7,725673	8,046196362	8,540438054	PCP029767	XM_008387234
PCP029771	8,495855027	8,158205728	8,101555535	8,206184105	PCP029771	XM_009339128
PCP029796	6,691115365	6,841344192	7,055282436	7,497213158	PCP029796	XM_009339181
PCP029812	8,011227255	8,139551352	8,13442632	8,566054038		
PCP029828	7,291585141	7,125878364	7,169925001	7,018700931	PCP029828	XM_009352514
PCP029860	7,743689989	7,798763389	7,894817763	8,306836389	PCP029860	XM_009351737
PCP029870	6,392317423	7,956114749	7,952275317	8,296136161	PCP029870	XM_009378746
PCP029911	8,56350137	8,566054038	8,499845887	9,033423002		
PCP029916	7,990557838	8,051643995	8,171577143	8,577428828		
PCP029917	7,725673	7,992485211	8,139551352	8,779161208		
PCP029939	7,977279923	7,711976642	7,622051819	7,453929061		
PCP029940	6,874428132	7,018700931	7,17990909	7,577428828		
PCP029955	7,822284016	7,792269724	7,963878669	8,417852515	PCP029955	XM_008390006

PCP029972	7,273049587	7,051698368	6,87036472	6,902917719	PCP029972	XM_009339033
PCP029983	8,74708524	8,853839746	8,247927513	7,781359714	PCP029983	XM_009381004
PCP029987	7,894817763	7,667679281	7,990557838	8,477070141		
PCP030010	7,044394119	7,269874722	7,464913269	7,979167931	PCP030010	XM_009337562
PCP030109	7,564759219	7,785746699	7,577428828	7,655852677	PCP030109	XM_009347403
PCP030114	7,087462841	7,186559982	7,896816753	8,372125027		
PCP030137	8,003770871	7,826548487	8,113742166	7,890750668	PCP030137	XM_016792276
PCP030138	7,254272787	7,146390476	7,531381461	8,171577143		
PCP030147	8,103287808	7,930737338	7,813781191	7,499845887	PCP030147	XM_009343135
PCP030211	7,562242424	7,389480771	7,977279923	7,415065677		
PCP030241	7,809478757	7,714245518	7,940577883	7,392317423	PCP030241	XM_009349744
PCP030272	8,103287808	7,83080039	7,300764373	6,862079387		
PCP030350	6,86628983	7,111970261	7,238404739	7,882643049	PCP030350	XM_009357448
PCP030353	7,451211112	7,459431619	7,815895594	8,058911723	PCP030353	XM_018645733
PCP030373	8,637820329	8,54303182	8,636624621	9,044394119		
PCP030379	7,824449651	8,027905997	8,318904285	7,912889336	PCP030379	XM_018642638
PCP030397	7,146390476	7,047996356	7,351645995	7,022367813	PCP030397	XM_009379231
PCP030406	7,026025399	7,142923928	7,386552314	7,87036472	PCP030406	NM_001328778
PCP030407	7,244791942	6,926829678	7,251056285	6,794415866	PCP030407	NM_001328779
PCP030416	6,958030641	6,906890596	7,244791942	6,534030467	PCP030416	XM_008381048
PCP030440	7,209453366	7,564759219	7,723353774	7,765998774	PCP030440	XM_008381088
PCP030467	7,312882955	7,440204752	7,28845085	7,235248379		
PCP030480	8,573647187	8,5824431	8,717676423	9,06608919		
PCP030493	6,946379968	7,062531903	7,257387843	7,864186145	PCP030493	XM_009362189
PCP030498	7,489205728	7,470292816	7,686500527	7,260496187		
PCP030499	7,526147081	7,389480771	7,14974712	7,768184325	PCP030499	XM_009365233
PCP030500	7,619633096	7,176621973	7,354646096	7,231989133	PCP030500	XM_009365231
PCP030516	7,705079392	7,44567705	7,327956767	7,481153605	PCP030516	XM_018648981
PCP030551	6,705010253	6,862079387	6,653490009	7,431873638	PCP030551	XM_008391575
PCP030553	8,101555535	8,21757002	8,266786541	8,70276142	PCP030553	XM_008391577
PCP030554	8,703903573	8,44431092	8,483815777	8,481113232	PCP030554	XM_008352728
PCP030559	7,351645995	8,09976859	7,688809791	6,564835417	PCP030559	XM_008391588
PCP030565	7,730164413	7,910912508	8,057071136	8,323415108	PCP030565	XM_008391594
PCP030573	7,186559982	7,653418353	7,587440004	7,693486957	PCP030573	XM_021959164
PCP030580	5,57500972	7,473056289	8,257387843	12,72238064	PCP030580	XM_009341717
PCP030584	8,438791853	8,637820329	8,782441329	8,973467779	PCP030584	XM_009341762
PCP030610	8,202956379	8,016808288	8,254225537	7,868328714	PCP030610	XM_009373675
PCP030623	7,95419631	7,354646096	8,031604721	7,241554209	PCP030623	XM_008370140
PCP030635	7,090959258	7,552054236	7,973439079	9,086587411	PCP030635	XM_008367509
PCP030647	7,451211112	7,557119022	7,908872939	8,471675214	PCP030647	XM_008380402
PCP030651	8,125826717	9,063395081	8,43325159	7,820178962	PCP030651	XM_009349836
PCP030678	8,326429487	8,323415108	8,156487632	8,189824559	PCP030678	XM_008379016
PCP030705	8,515699838	8,526186214	8,654636029	9,202123824	PCP030705	XM_017324981
PCP030714	7,090959258	7,369204723	7,193081765	6,777025123	PCP030714	XM_009372385
PCP030716	8,536558066	8,3994702	8,125826717	7,510408147	PCP030716	XM_009372387
PCP030726	7,908872939	7,761551232	7,698149009	7,50247382	PCP030726	XM_008383448

PCP030731	7,973439079	8,103287808	8,089212109	8,485145023	PCP030731	XM_009353345
PCP030733	8,463524373	8,622051819	8,739206792	9,169925001	PCP030733	XM_009353347
PCP030745	7,426264755	6,820178962	7	6,464831606	PCP030745	XM_018646443
PCP030752	8,795487741	8,649866903	8,706219712	8,530094158		
PCP030758	7,333870928	7,459431619	7,491853096	7,166615031		
PCP030779	8,32494558	8,479780264	8,482485305	8,87036472	PCP030779	XM_009351105
PCP030780	7,22881869	7,241554209	7,212666605	7,721099189	PCP030780	XM_009351107
PCP030790	7,785746699	7,938579853	7,813781191	7,772611496	PCP030790	XM_008380898
PCP030791	8,220765252	8,50779464	8,742578916	9,053464362		
PCP030817	8,448446735	8,283875484	8,403736386	8,252665432	PCP030817	XM_008356829
PCP030822	8,233619677	7,815895594	8,186510462	7,354646096	PCP030822	XM_009348974
PCP030823	7,518299077	7,667679281	7,866228001	7,574934058	PCP030823	XM_009348975
PCP030868	8,615923847	8,574896225	8,67479253	9,031577154		
PCP030872	7,8008999	7,592457037	7,914863459	8,130982335	PCP030872	XM_009371980
PCP030888	8,373604714	8,21757002	8,191454081	7,837123296	PCP030888	XM_009366687
PCP030893	7,709635275	8,130982335	8,495855027	8,677120596	PCP030893	XM_009359920
PCP030904	6,235152624	6,409390936	6,946379968	7,807354922	PCP030904	XM_009347201
PCP030910	8,687655621	8,54303182	8,329437582	8,830768706	PCP030910	XM_009380508
PCP030915	8,937609223	8,682994584	8,861086906	8,821231873		
PCP030924	7,389480771	7,478405614	7,646234675	8,154818109	PCP030924	XR_664385
PCP030931	8,832890014	8,659389441	8,721099189	8,725638921	PCP030931	XM_008360111
PCP030933	7,069637728	7,459431619	7,597456684	8,001858526		
PCP030955	7,456724026	7,992485211	7,87036472	7,426264755	PCP030955	XM_009342301
PCP030957	7,483815777	7,754887502	7,473056289	7,136170875	PCP030957	XM_009342310
PCP030975	7,908872939	8,19313106	8,106798463	8,696967526		
PCP030979	6,745909573	7,173227395	7,247927513	7,743689989	PCP030979	XM_018648999
PCP030992	8,08390476	7,946438456	8,174925683	8,607330314	PCP030992	XM_008350106
PCP030993	8,383704292	8,125826717	8,548166865	8,463524373	PCP030993	XM_008381819
PCP031004	7,646234675	7,366322214	7,483815777	7,251056285	PCP031004	XM_008347289
PCP031020	8,687655621	8,655816908	8,732438807	8,601139355	PCP031020	XM_018649542
PCP031027	7,572359168	7,351645995	7,554588852	8,323415108	PCP031027	XM_009380032
PCP031029	7,969645536	8,202956379	8,020535537	8,715378619	PCP031029	XM_009379308
PCP031053	7,691185174	7,750405521	7,714245518	8,173277373	PCP031053	XM_009377598
PCP031070	6,86628983	6,667750232	7,276124405	7,403693786	PCP031070	XM_018647588
PCP031105	7,403693786	7,080337882	6,862079387	6,254178286	PCP031105	XM_009380891
PCP031108	7,892816	7,884658968	8,288496992	8,652235507		
PCP031111	7,958088658	7,872397856	8,225593068	8,70158371	PCP031111	XM_008359326
PCP031135	8,317412614	8,28077077	8,588714636	8,409390936	PCP031135	XM_009370349
PCP031155	6,754887502	7,06608919	7,044394119	7,429030064		
PCP031165	7,619633096	7,589988142	7,489205728	8,096293483	PCP031165	XM_009381013
PCP031184	6,705010253	6,942514505	6,961970533	7,426264755	PCP031184	XM_008353598
PCP031186	7,146390476	7,564759219	8,055282436	8,241601875	PCP031186	XM_017322507
PCP031220	8,60855054	8,675957033	8,666508075	8,19313106	PCP031220	XM_018643715
PCP031222	7,723353774	7,798763389	8,206184105	8,904875427	PCP031222	XM_009341449
PCP031249	7,247927513	6,977279923	6,934634441	7,711976642	PCP031249	XM_009364145
PCP031252	9,05709822	8,910882535	8,86727874	7,730164413	PCP031252	XM_009336773

PCP031267	6,80309785	7,473056289	8,230404783	9,083027168	PCP031267	XM_018649615
PCP031271	7,805227956	7,975389442	8,101555535	8,312882955		
PCP031317	7,17990909	7,183188734	7,285402219	6,828707735	PCP031317	XM_009380272
PCP031325	7,007531912	7,118941073	7,528805707	7,648681141	PCP031325	XM_009378544
PCP031330	7,940577883	8,078577814	7,916894583	8,376472723	PCP031330	XM_009365176
PCP031335	7,626950122	7,688809791	7,839203788	8,233619677	PCP031335	XM_009365180
PCP031338	8,569855608	8,687655621	8,772578508	9,026053072	PCP031338	XM_018648968
PCP031339	7,037052702	7,199672345	7,426264755	7,007531912	PCP031339	XM_018648969
PCP031352	8,042589623	8,386595423	8,460783512	8,184875343		
PCP031354	7,589988142	7,759355602	7,783522135	8,437419184		
PCP031357	8,74819285	8,841312739	8,554588852	8,646270682		
PCP031361	7,577428828	7,520972191	7,841281284	8,196380818	PCP031361	XM_018651243
PCP031370	7,351645995	7,417852515	8,315919398	8,606109055	PCP031370	XM_009350556
PCP031373	7,944448531	7,392317423	7,282347131	7,129283017	PCP031373	XM_009360449
PCP031391	7,244791942	7,241554209	7,677155841	7,969645536		
PCP031397	7,315874125	6,910852562	6,815959618	6,81159947		
PCP031410	8,184875343	8,166564822	8,238404739	8,70276142		
PCP031424	8,370687407	8,431831861	8,594959026	8,861086906	PCP031424	XM_009379891
PCP031439	7,007531912	7,369204723	7,115511897	6,72342204	PCP031439	XM_009381053
PCP031451	8,427648072	8,236827423	8,347267018	8,146339557	PCP031451	XM_020565225
PCP031453	7,357552005	7,646234675	7,922851957	7,996219247		
PCP031469	8,042589623	8,073231127	8,020535537	8,005624549	PCP031469	XM_009367417
PCP031484	8,290018847	7,849686575	8,407989993	9,302250498	PCP031484	XM_008366373
PCP031488	7,554588852	7,607330314	7,908872939	7,617136829	PCP031488	XM_008351660
PCP031492	8,163247124	8,08390476	8,057071136	7,910912508		
PCP031493	8,577428828	8,303780748	8,354602022	8,458078458	PCP031493	XM_009340098
PCP031514	7,83080039	7,672425342	7,46760555	7,64385619	PCP031514	XM_008356426
PCP031518	7,64385619	7,403693786	7,686500527	7,369204723	PCP031518	XM_009335986
PCP031520	7,426264755	7,528805707	7,318949464	7,403693786	PCP031520	XM_009335988
PCP031536	7,763743526	7,222360218	6,87036472	6,497133304	PCP031536	XM_017336038
PCP031547	8,813781191	8,441574774	8,46760555	7,910912508	PCP031547	XM_018652348
PCP031562	7,834976616	8,383704292	8,75043902	9,069664578	PCP031562	XM_009350678
PCP031571	8,227230852	8,181549844	8,136119594	8,533991546		
PCP031572	7,058857621	7,139551352	7,315874125	7,741466986	PCP031572	XM_017323727
PCP031587	7,173227395	7,142923928	7,206232954	6,691115365	PCP031587	XM_018647899
PCP031591	7,904905525	7,677155841	7,357552005	7,136170875	PCP031591	XM_009379413
PCP031598	7,54689446	7,961912672	7,707359132	6,64385619	PCP031598	XM_009345347
PCP031614	8,436003553	8,323415108	8,477070141	8,842350343		
PCP031668	6,857980995	6,727920455	6,841344192	7,486473046	PCP031668	XM_009380571
PCP031684	7,888743249	7,743689989	8,018645301	7,513095909	PCP031684	XM_018646986
PCP031690	7,448488033	7,318949464	7,206232954	6,988684687	PCP031690	XM_018651433
PCP031705	7,22881869	7,014913158	6,658211483	6,619559738	PCP031705	XM_009356639
PCP031767	8,658211483	8,625708843	8,631795481	9,188168462		
PCP031781	7,448488033	7,478405614	7,30679083	7,186559982	PCP031781	XM_008370937
PCP031811	7,389480771	7,932687205	7,523561956	7,473056289	PCP031811	XM_009352290
PCP031819	8,593689902	8,130982335	8,252665432	7,975389442	PCP031819	XM_009353431

PCP031860	8,667714757	8,596189756	8,623259662	8,382278171	PCP031860	XM_009344604
PCP031885	7,700439718	7,557119022	7,567347696	7,354646096	PCP031885	XM_018649973
PCP031894	7,321928095	7,244791942	7,260496187	7,790055203	PCP031894	XM_009353831
PCP031928	6,857980995	6,853870927	7,22881869	7,478405614	PCP031928	XM_008345935
PCP031950	8,77478706	8,87958325	8,84862294	8,718806834	PCP031950	XM_009367240
PCP031983	8,204571144	7,665335917	7,752681699	8,462175047	PCP031983	XM_009379407
PCP031990	8,533991546	8,501160452	8,868297843	9,155653112		
PCP032019	8,796591265	8,710806434	8,757123282	8,710806434		
PCP032069	8,511752654	8,532667615	8,601139355	9,082149041		
PCP032102	8,350187812	8,291539098	8,273002948	8,171577143		
PCP032162	7,818070833	7,87036472	8,003770871	8,395191361		
PCP032175	8,890781061	8,463524373	8,225593068	8,227230852	PCP032175	XM_018645127
PCP032178	8,060695932	7,711976642	8,020535537	7,61227877	PCP032178	XM_009347990
PCP032179	7,851749041	7,607330314	7,662988741	7,206232954	PCP032179	XM_009347952
PCP032183	8,576181982	8,46760555	8,81483878	9,027905997	PCP032183	XM_009348173
PCP032184	7,202907418	7,28845085	7,257387843	7,062531903		
PCP032189	8,288496992	8,447083226	8,615923847	8,828676005	PCP032189	XM_008379457
PCP032192	7,653418353	7,478405614	7,739240553	8,164906927		
PCP032228	8,022367813	8,329437582	8,207844058	8,239980333	PCP032228	XM_017331225
PCP032260	8,603626345	8,518338423	8,736976865	8,975360779	PCP032260	XM_009363613
PCP032292	8,447083226	8,819125283	8,732438807	8,845490051	PCP032292	XM_009343229
PCP032444	6,961970533	7,029784146	7,279192684	8,049848549	PCP032444	XM_009361276
PCP032450	7,906890596	8,049848549	8,013071384	7,984874125	PCP032450	XM_018649827
PCP032498	7,058857621	7,366322214	8,382278171	8,073231127	PCP032498	XM_008376152
PCP032512	7,42063398	7,098032083	7,285402219	7,044394119		
PCP032534	7,360452072	7,670089681	7,602364826	8,125826717		
PCP032539	7,21916852	7,431873638	7,730164413	8,103287808		
PCP032550	6,691115365	6,84962403	7,022367813	7,330916878		
PCP032576	8,730198386	8,317412614	8,173277373	8,222408523		
PCP032579	8,417852515	8,816983623	8,78571401	9,108524457		
PCP032602	8,495855027	8,235200503	8,470333494	8,429071922	PCP032602	XM_009341008
PCP032604	8,436003553	8,363390129	8,43325159	9,121533517	PCP032604	XM_017327798
PCP032610	8,30833903	8,441574774	8,497173232	8,805195705		
PCP032616	8,176572111	8,628153873	8,689997971	8,641437649		
PCP032626	8,241601875	8,376472723	8,348728154	8,141238626		
PCP032642	7,531381461	7,451211112	7,554588852	7,251056285	PCP032642	XM_009359158
PCP032789	7,235248379	7,251056285	7,61227877	7,333870928	PCP032789	XM_009360340
PCP032791	8,62450305	8,571107931	8,572397068	8,960001932	PCP032791	XR_668950
PCP032819	8,277659361	8,581200582	8,5824431	8,935666001		
PCP032827	7,665335917	7,754887502	7,552054236	8,115459877		
PCP032871	7,562242424	7,609769734	7,62935662	8,071462363	PCP032871	XM_009374920
PCP032873	8,373604714	8,388017285	8,420676082	8,341318667		
PCP032893	8,406545173	8,106798463	7,88062431	7,956114749	PCP032893	XM_009351195
PCP032907	8,053491515	8,168270966	8,375039431	9,07325791		
PCP032923	7,695785075	7,705079392	7,515699838	8,169925001		
PCP032932	8,594959026	8,540438054	8,74708524	9,237616296		

PCP033027	7,176621973	7,266786541	7,142923928	7,693486957		
PCP033080	7,297649983	7,383704292	7,515699838	7,212666605	PCP033080	XM_009348643
PCP033090	6,886672074	6,918863237	6,95419631	7,743689989	PCP033090	XM_008394930
PCP033092	7,285402219	7,129283017	7,257387843	6,705010253		
PCP033094	8,499845887	8,598685286	8,772578508	9,031577154	PCP033094	XM_008387056
PCP033096	8,550746785	8,321928095	8,422064766	8,189824559	PCP033096	XM_008351519
PCP033107	6,878602742	6,890811455	7,231989133	8,271463028	PCP033107	XM_009353284
PCP033110	7,781359714	7,462134139	7,892816	7,651051691	PCP033110	XM_009353258
PCP033112	8,640244936	8,460783512	8,721099189	9,218369492	PCP033112	XM_018646421
PCP033128	8,84024291	8,174925683	8,422064766	8,020535537	PCP033128	XM_009367360
PCP033132	6,54426899	6,481072857	7,300764373	8,222408523	PCP033132	XM_009363028
PCP033176	7,660566438	7,597456684	7,582480735	8,28077077		
PCP033183	8,09976859	8,336863563	7,904905525	7,202907418		
PCP033188	7,977279923	7,936637939	8,137862104	8,420676082		
PCP033195	7,50517639	7,206232954	7,473056289	7,855927425		
PCP033198	7,572359168	7,798763389	7,834976616	8,125826717	PCP033198	XM_009370210
PCP033201	6,777025123	8,520932916	8,631795481	8,491853096	PCP033201	XM_018650071
PCP033217	5,746043983	6,414981143	7,166615031	8,243173983	PCP033217	XM_009373639
PCP033219	6,965784285	7,029784146	6,759289016	6,807354922	PCP033219	XM_018652392
PCP033239	7,714245518	7,693486957	7,698149009	8,269921462	PCP033239	XM_009347165
PCP033246	7,372168569	6,918863237	6,750472519	6,54426899	PCP033246	XM_009373037
PCP033264	7,872397856	7,832890014	7,914863459	8,369248353		
PCP033283	7,263691734	7,202907418	6,86628983	7,018700931	PCP033283	XM_009342635
PCP033414	7,183188734	6,772545519	6,894817763	6,658211483	PCP033414	XM_009374524
PCP033421	6,03716255	6,183288001	7,672425342	7,798763389	PCP033421	XM_008379846
PCP033439	6,862079387	7,029784146	7,363433935	8,342830273		
PCP033481	7,173227395	7,169925001	6,662917555	7,700439718	PCP033481	XM_009339388
PCP033523	7,604886762	7,641473777	7,653418353	8,106798463		
PCP033538	7,212666605	7,153095972	7,366322214	7,77036657	PCP033538	XM_009353077
PCP033600	7,111970261	7,470292816	7,822284016	8,382278171	PCP033600	XM_008350367
PCP033609	6,97349648	7,105070402	6,918863237	7,434628228		
PCP033630	7,497213158	7,366322214	7,691185174	8,144658243	PCP033630	XM_009339849
PCP033657	8,294620749	8,252665432	8,241601875	8,709669735		
PCP033660	7,007531912	7,209453366	7,442943496	7,839203788		
PCP033700	7,007531912	7,118941073	7,417852515	7,125878364	PCP033700	XM_009380354
PCP033765	8,510447709	8,497173232	8,794415866	9,197216693	PCP033765	XM_008377886
PCP033793	7,108524457	7,483815777	7,111970261	6,222456826	PCP033793	XM_009342438
PCP033798	7,698149009	7,285402219	7,50247382	7,303780748		
PCP033800	7,996219247	7,845490051	8,144658243	7,900866808	PCP033800	XM_009374085
PCP033833	7,574934058	7,564759219	8,04072808	8,080391184		
PCP033848	8,094500005	8,118941073	8,249492747	8,611024797	PCP033848	XR_665987
PCP033850	7,71651083	7,992485211	7,847558521	8,359002767		
PCP033868	8,700439718	8,623259662	8,613494819	8,515699838	PCP033868	XM_009359948
PCP033890	8,714245518	8,526186214	8,56350137	9,283088353		
PCP033934	8,220765252	8,311385042	8,309840108	8,033423002	PCP033934	XM_009339009
PCP033962	7,051698368	7,062531903	7,153095972	7,670089681	PCP033962	XM_009367582

PCP033989	7,28845085	7,303780748	7,244791942	6,882643049	PCP033989	XR_001953124
PCP033998	7,297649983	7,674757228	7,695785075	7,549438149		
PCP034140	8,273002948	8,257387843	8,139551352	8,588714636	PCP034140	XM_008351817
PCP034153	7,327956767	7,06608919	6,950351762	7,018700931		
PCP034170	8,400879436	8,482485305	8,733591606	9,274564521	PCP034170	XM_009338093
PCP034191	7,225641265	7,403693786	7,189824559	6,790120385	PCP034191	XM_009351549
PCP034196	7,787902559	7,886733033	8,194756854	8,531381461	PCP034196	XM_009344000
PCP034204	8,742578916	8,794415866	8,768184325	8,499845887	PCP034204	XM_009342990
PCP034404	7,398059585	7,434628228	8,183238369	7,193081765	PCP034404	XM_018650233
PCP034421	7,193081765	6,922792504	7,029784146	7,562242424		
PCP034453	7,8008999	7,727920455	8,098032083	8,385129006		
PCP034475	6,599912842	6,579994693	7,202907418	7,386552314	PCP034475	XM_009380848
PCP034493	7,757089938	7,803033252	7,866228001	8,297695831		
PCP034497	8,303780748	8,406545173	8,698114274	9,158180476	PCP034497	XM_009345513
PCP034524	8,163247124	8,031604721	8,194756854	8,520932916	PCP034524	XM_009369321
PCP034527	7,08395793	6,878602742	7,040782866	7,667679281		
PCP034645	6,459431619	6,777025123	7,156538193	7,136170875	PCP034645	XM_009373347
PCP034650	8,649866903	8,622051819	8,649866903	8,485145023	PCP034650	XM_008339822
PCP034660	7,986809101	6,351734323	8,115459877	7,186559982		
PCP034679	7,872397856	7,360452072	7,752681699	7,324900589	PCP034679	XM_018649220
PCP034714	6,420718183	6,950351762	7,146390476	6,981053471		
PCP034716	7,451211112	7,779194046	7,607330314	7,541716163	PCP034716	XM_009365406
PCP034718	7,792269724	7,790055203	8,014968933	8,382278171		
PCP034719	8,120652609	8,198052084	8,456682963	8,751544059		
PCP034739	7,431873638	7,489205728	7,651051691	8,031604721		
PCP034745	8,225593068	8,350187812	8,402287298	8,739206792	PCP034745	XM_009365372
PCP034752	8,309840108	8,163247124	8,130982335	8,166564822	PCP034752	XM_009365793
PCP034769	7,047996356	7,30679083	7,28845085	7,658211483	PCP034769	XM_008371565
PCP034780	8,695819867	8,382278171	8,50779464	8,573647187	PCP034780	XM_009359219
PCP034800	7,156538193	7,309885571	7,478405614	7,920829209	PCP034800	XR_666931
PCP034806	7,567347696	7,303780748	7,28845085	7,315874125	PCP034806	XM_009375127
PCP034824	6,99242856	7,105070402	7,189824559	6,709704193		
PCP034859	7,080337882	6,754887502	7,577428828	7,622051819	PCP034859	XM_008384297
PCP034936	7,389480771	7,369204723	7,533952624	7,22881869		
PCP034957	7,533952624	8,007475849	8,069691427	8,375039431		
PCP034993	8,202956379	8,174925683	8,338379842	8,682994584		
PCP035053	6,97349648	6,781359714	7,040782866	7,451211112		
PCP035058	7,910912508	7,303780748	8,173277373	7,92677039	PCP035058	XM_017332417
PCP035064	7,118941073	7,626950122	8,186510462	9,202931899	PCP035064	XM_021977884
PCP035107	7,383704292	7,559721231	7,73470962	8,001858526	PCP035107	XR_001787405
PCP035113	8,345760055	8,495855027	8,659389441	9,116343961	PCP035113	XM_018649358
PCP035115	7,639015048	7,440204752	7,523561956	7,206232954	PCP035115	XM_009371556
PCP035116	7,022367813	6,984931073	7,146390476	6,768184325	PCP035116	XM_009371551
PCP035150	7,890750668	8,003770871	7,714245518	7,772611496		
PCP035241	8,598685286	8,780277286	8,54689446	8,708497652	PCP035241	XM_018642596
PCP035287	8,626913629	8,489165579	8,550746785	8,526186214	PCP035287	XM_009341476

PCP035326	7,324900589	7,459431619	7,898873426	8,375039431	PCP035326	XM_009362864
PCP035332	6,475733431	6,977279923	7,222360218	7,739240553	PCP035332	XM_009356960
PCP035409	6,108524457	6,260590275	6,763809907	8,45532722	PCP035409	XR_669584
PCP035444	8,283875484	8,204571144	8,233619677	8,078577814	PCP035444	XM_009344114
PCP035541	7,691185174	7,257387843	7,489205728	7,938579853		
PCP035559	8,089212109	8,441574774	9,092757141	9,641455713	PCP035559	XM_009339319
PCP035592	7,549438149	7,459431619	7,3458044	7,357552005	PCP035592	XM_009372999
PCP035594	6,81159947	7,094552786	7,222360218	7,750405521		
PCP035604	7,982993575	7,874489611	9,029756544	9,151447887		
PCP035611	8,263644792	8,055282436	8,118941073	8,144658243	PCP035611	XM_008349278
PCP035615	7,994353437	7,805227956	7,855927425	7,695785075	PCP035615	XM_009336462
PCP035621	7,125878364	6,886672074	6,97349648	6,918863237	PCP035621	XM_009338027
PCP035623	8,252665432	8,130982335	8,379378367	8,222408523		
PCP035632	7,297649983	7,412188747	7,574934058	8,087462841		
PCP035642	7,743689989	7,815895594	7,975389442	7,77478706	PCP035642	XM_009367510
PCP035644	8,201290788	8,178266468	8,222408523	8,74929961	PCP035644	XM_009353867
PCP035686	8,672425342	8,682994584	8,664162806	8,619596417	PCP035686	XM_018649228
PCP035687	7,464913269	7,448488033	7,300764373	7,176621973		
PCP035745	8,598685286	8,517000043	8,603626345	8,966736149		
PCP035780	7,375039431	6,942514505	7,273049587	6,339850003		
PCP035854	8,630558365	8,623259662	8,56350137	8,194756854		
PCP035860	8,141238626	8,257387843	8,184875343	8,118941073	PCP035860	XM_009378982
PCP035861	7,961912672	7,884658968	8,258942852	8,695819867	PCP035861	XM_018652190
PCP035866	7,667679281	7,44567705	7,470292816	7,975389442	PCP035866	XM_009374268
PCP035875	7,118941073	7,544346278	7,415065677	7,423494135	PCP035875	XM_009375942
PCP035922	7,21916852	7,451211112	7,202907418	6,902917719		
PCP035952	7,982993575	8,113742166	8,148069774	8,505136683		
PCP035955	8,251103639	8,184875343	7,619633096	7,730164413	PCP035955	XM_018647763
PCP035984	7,360452072	7,451211112	7,518299077	7,916894583	PCP035984	XM_009366222
PCP035994	8,230404783	8,695819867	8,539158811	8,623259662	PCP035994	XM_017327416
PCP035995	6,695854658	6,64385619	6,794415866	7,718841075	PCP035995	XM_009379867
PCP036017	7,17990909	7,369204723	7,768184325	8,118941073	PCP036017	XM_009339270
PCP036035	7,796558821	7,965784285	8,215921225	8,438791853		
PCP036076	7,845490051	7,624466495	7,665335917	7,64385619		
PCP036087	7,380850638	7,336908182	7,520972191	8,098032083		
PCP036200	8,268331458	8,235200503	7,695785075	7,139551352	PCP036200	XM_009338194
PCP036218	9,104153166	8,654636029	8,694636474	8,43325159	PCP036218	XM_009346201
PCP036221	7,531381461	7,257387843	7,22881869	7,351645995	PCP036221	XM_009346095
PCP036256	8,189824559	8,038918989	8,168270966	7,934693407	PCP036256	XM_009341485
PCP036257	7,589988142	7,562242424	8,312882955	8,222408523		
PCP036268	6,837060204	6,672425342	6,95419631	7,569855608	PCP036268	XM_009356070
PCP036343	7,499845887	7,577428828	7,779194046	8,630558365	PCP036343	XM_009337868
PCP036359	7,648681141	8,031604721	7,98111057	8,445718428	PCP036359	XM_009379514
PCP036374	7,033423002	6,918863237	6,930737338	6,759289016	PCP036374	XM_018644089
PCP036457	7,994353437	7,641473777	7,876516947	7,619633096	PCP036457	XM_009373718
PCP036485	8,744833837	8,44431092	8,553322101	8,677120596	PCP036485	XM_008391521

PCP036500	8,076815597	8,458078458	8,136119594	7,670089681	PCP036500	XM_008371996
PCP036502	7,965784285	7,423494135	7,342785837	7,014913158	PCP036502	XM_008390090
PCP036513	7,058857621	7,159871337	7,263691734	7,963878669		
PCP036604	7,273049587	7,342785837	7,662988741	7,948367232		
PCP036605	8,020535537	8,046196362	7,820178962	7,798763389		
PCP036624	8,367764188	6,926829678	7,61227877	8,049848549	PCP036624	XM_009353898
PCP036629	8,32791187	8,258942852	8,274587815	8,204571144		
PCP036634	8,038918989	8,299208018	8,311385042	9,136991112	PCP036634	XM_008339651
PCP036670	7,222360218	7,244791942	7,176621973	6,709704193		
PCP036703	8,491853096	8,082149041	7,924812504	7,739240553	PCP036703	XM_009369475
PCP036798	7,982993575	8,024197765	7,88062431	8,344295908	PCP036798	XM_009335917
PCP036813	7,862141394	7,315874125	7,285402219	6,189824559	PCP036813	XM_008389727
PCP036824	7,044394119	7,040782866	7,153095972	7,820178962	PCP036824	XM_008345143
PCP036825	7,781359714	7,845490051	7,884658968	8,550746785		
PCP036851	6,609843592	6,667750232	6,890811455	7,518299077		
PCP036856	7,523561956	7,395148508	7,651051691	8,168270966		
PCP036879	7,820178962	8,014968933	7,98111057	8,385129006		
PCP036895	9,072347016	8,539158811	8,466260038	8,859006685	PCP036895	XM_009338205
PCP036965	7,617136829	8,058911723	8,376472723	9,842868866	PCP036965	XM_009374306
PCP036977	7,080337882	7,196430001	7,448488033	8,029728942	PCP036977	XM_009368915
PCP037142	6,918863237	7,129283017	6,97349648	6,745909573	PCP037142	XR_525870
PCP037164	7,815895594	8,022367813	8,382278171	8,67479253	PCP037164	XM_008344069
PCP037243	7,105070402	6,777025123	6,794415866	6,824386003	PCP037243	XM_018646648
PCP037254	7,051698368	7,189824559	7,247927513	7,639015048	PCP037254	XM_009341786
PCP037353	7,920829209	7,882643049	7,772611496	8,202956379		
PCP037431	8,3994702	8,419243918	8,675957033	8,865207434	PCP037431	XM_009373768
PCP037481	8,459431619	8,417852515	8,447083226	8,851749041	PCP037481	XM_009354223
PCP037483	6,72342204	6,72342204	7,040782866	7,400879436	PCP037483	XM_009354215
PCP037495	9,124121312	8,833933692	8,792237182	7,44567705		
PCP037514	7,437377568	7,483815777	7,634230226	7,984874125	PCP037514	XM_009352621
PCP037521	8,73470962	8,586201784	8,659389441	8,564797319	PCP037521	XM_018649068
PCP037525	8,227230852	8,458078458	8,513056419	9,092757141		
PCP037538	8,811631578	8,323415108	8,632995197	8,383704292	PCP037538	XM_009338445
PCP037565	8,552015799	8,640244936	8,753785022	9,169925001	PCP037565	XM_009363787
PCP037580	7,462134139	7,285402219	6,357552005	7,209453366		
PCP037599	6,528727582	7,142923928	6,235152624	8,125826717		
PCP037600	8,660602089	8,653454181	8,757123282	9,146365017		
PCP037604	8,001858526	8,265239967	8,515699838	8,630558365	PCP037604	XM_009378743
PCP037626	8,224001674	8,125826717	8,04072808	8,459431619	PCP037626	XM_009372247
PCP037794	6,922792504	6,965784285	7,241554209	7,536596918	PCP037794	XM_017327858
PCP037800	7,686500527	7,653418353	7,890750668	8,396604781	PCP037800	XM_009359928
PCP037806	7,437377568	7,363433935	7,732472726	8,003770871	PCP037806	XM_009377004
PCP037869	7,324900589	7,257387843	7,497213158	7,920829209	PCP037869	XM_008382516
PCP037972	8,176572111	7,894817763	8,268331458	7,882643049	PCP037972	XM_009364077
PCP038033	7,375039431	7,497213158	7,77036657	8,013071384	PCP038033	XM_009357363
PCP038035	8,064311643	8,103287808	8,173277373	7,977279923		

PCP038048	7,324900589	7,44567705	7,369204723	7,900866808		
PCP038049	8,567309664	8,409390936	8,658211483	9,152284842		
PCP038106	7,741466986	8,101555535	8,373604714	8,22881869	PCP038106	XM_009356934
PCP038137	8,151422517	8,329437582	8,431831861	8,658211483	PCP038137	XM_008394169
PCP038151	8,291539098	8,493174961	8,775873612	9,359024737	PCP038151	XM_009348342
PCP038219	7,544346278	7,894817763	7,922851957	8,201290788	PCP038219	XM_009375075
PCP038303	7,569855608	7,660566438	7,360452072	7,327956767	PCP038303	XM_009362491
PCP038321	7,055282436	7,291585141	7,602364826	7,464913269	PCP038321	XM_018650520
PCP038347	7,247927513	7,202907418	7,238404739	7,787902559	PCP038347	XM_009359606
PCP038357	8,09976859	7,434628228	6,946379968	7,489205728	PCP038357	XM_009353070
PCP038379	6,965784285	6,653490009	6,824386003	7,333870928		
PCP038384	7,541716163	6,266786541	7,101503009	8,118941073	PCP038384	XM_008240044
PCP038385	7,183188734	7,609769734	7,279192684	7,50247382	PCP038385	XM_021946104
PCP038410	6,247927513	6,737010678	6,882643049	7,641473777	PCP038410	XM_008390049
PCP038435	7,451211112	7,369204723	7,269874722	7,105070402		
PCP038445	8,775873612	8,843387202	8,839203788	8,479780264	PCP038445	XM_008366781
PCP038576	8,946409212	8,416459769	8,850718177	9,01402047	PCP038576	XM_009356975
PCP038607	6,886672074	6,705010253	6,988684687	7,587440004		
PCP038625	7,805227956	7,736943052	7,813781191	8,357552005	PCP038625	XM_009344565
PCP038637	7,624466495	7,950293432	8,329437582	8,359002767	PCP038637	XM_008366667
PCP038643	7,459431619	7,464913269	7,464913269	8,752648252		
PCP038654	7,577428828	7,269874722	7,646234675	7,990557838		
PCP038671	8,577428828	7,54689446	7,478405614	7,617136829		
PCP038700	7,475733431	6,886672074	7,315874125	7,206232954	PCP038700	XR_001786501
PCP038874	8,528766645	8,535275377	8,667714757	9,084808388	PCP038874	XM_009341265
PCP038902	7,589988142	7,567347696	7,667679281	8,108524457	PCP038902	XM_009342784
PCP038912	7,3458044	7,754887502	7,497213158	7,483815777	PCP038912	XM_009353483
PCP038916	8,060695932	8,101555535	8,164906927	8,813781191		
PCP038917	7,660566438	7,792269724	7,826548487	8,370687407		
PCP038925	7,269874722	7,108524457	7,617136829	8,013071384	PCP038925	XR_001954399
PCP038951	8,680640826	8,489165579	8,535275377	8,536558066	PCP038951	XM_009374231
PCP038995	7,986809101	7,994353437	8,447083226	8,805195705	PCP038995	XM_009367558
PCP038998	8,161535025	8,198052084	8,459431619	8,212715235	PCP038998	XM_009348512
PCP039021	7,794415866	7,686500527	7,700439718	7,639015048	PCP039021	XM_009370465
PCP039027	7,946438456	8,127581695	7,914863459	7,602364826	PCP039027	XM_009370185
PCP039032	8,505136683	8,625708843	8,594959026	9,08127038	PCP039032	XM_009341358
PCP039035	6,513016928	6,737010678	8,464872438	7,807354922	PCP039035	NM_001319261
PCP039058	7,263691734	7,423494135	7,617136829	8,124121312		
PCP039062	8,038918989	7,826548487	8,317412614	8,761551232	PCP039062	XM_009347602
PCP039087	7,244791942	6,926829678	6,950351762	6,902917719		
PCP039100	8,460783512	8,168270966	8,339850003	8,146339557	PCP039100	XM_009355812
PCP039124	7,721099189	7,448488033	7,389480771	7,21916852	PCP039124	XM_008366547
PCP039130	7,014913158	6,965784285	6,790120385	7,375039431	PCP039130	XM_018649514
PCP039167	7,348728154	7,473056289	7,398059585	7,260496187		
PCP039192	7,805227956	8,470333494	8,220765252	8,505136683		
PCP039240	8,144658243	8,078577814	8,305286574	8,009380767	PCP039240	XM_009364837

PCP039279	7,215969746	7,011227255	7,146390476	7,604886762		
PCP039292	8,321928095	8,419243918	8,61717357	8,911901261	PCP039292	XR_001953444
PCP039378	8,057071136	8,014968933	8,511752654	8,478446064		
PCP039452	7,122362117	7,142923928	7,363433935	7,783522135		
PCP039457	6,677085351	7,189824559	7,011227255	7,090959258	PCP039457	XM_018642983
PCP039468	7,536596918	7,843418611	7,691185174	7,648681141	PCP039468	XM_009380354
PCP039497	8,082149041	8,426264755	8,42488011	8,724513853	PCP039497	XM_017337017
PCP039515	8,163247124	8,255831156	8,21106088	8,049848549	PCP039515	XM_009356631
PCP039528	7,707359132	7,582480735	7,813781191	8,199672345		
PCP039534	6,609843592	6,768184325	6,72342204	7,327956767	PCP039534	XM_009335823
PCP039547	7,451211112	7,582480735	7,64385619	8,009380767		
PCP039574	8,309840108	8,21106088	8,345760055	8,705044823	PCP039574	XM_017335225
PCP039581	5,513174885	7,122362117	7,74819285	7,564759219	PCP039581	XM_009363631
PCP039584	8,009380767	8,434628228	8,555854491	8,682994584	PCP039584	XM_008377264
PCP039590	7,122362117	6,922792504	7,215969746	7,559721231		
PCP039594	7,977279923	7,973439079	7,847558521	8,645045922		
PCP039595	8,514398461	8,42488011	8,725638921	8,973467779		
PCP039610	8,202956379	8,169925001	7,822284016	7,662988741	PCP039610	XM_009362146
PCP039614	8,390899794	8,687655621	8,528766645	8,668884984	PCP039614	XM_009353034
PCP039650	6,894817763	7,403693786	7,736943052	8,24474438		
PCP039687	8,856954575	8,594959026	8,661778098	9,054387253	PCP039687	XM_008347484
PCP039790	8,184875343	8,194756854	8,698114274	8,470333494	PCP039790	XM_017332151
PCP039795	8,232037117	7,967687386	7,860031646	7,750405521	PCP039795	XM_009346750
PCP039814	7,196430001	7,007531912	7,768184325	7,453929061		
PCP039987	7,451211112	7,101503009	7,42063398	6,624539604	PCP039987	XM_018643145
PCP040042	8,579956993	8,302273349	8,163247124	8,055282436	PCP040042	XM_008358734
PCP040047	7,648681141	7,83080039	7,662988741	7,688809791	PCP040047	XM_009372722
PCP040109	7,276124405	7,132679654	7,459431619	7,736943052		
PCP040131	7,686500527	7,920829209	7,709635275	7,153095972	PCP040131	XM_008374694
PCP040157	8,06786455	8,235200503	7,826548487	7,196430001	PCP040157	XM_009361802
PCP040158	8,269921462	8,412231097	8,578674597	8,761551232	PCP040158	XM_009361800
PCP040178	6,763809907	6,981053471	7,50517639	8,113742166	PCP040178	XM_009370099
PCP040191	7,156538193	7,282347131	7,478405614	7,798763389	PCP040191	XM_009348358
PCP040225	7,462134139	7,634230226	7,360452072	6,894817763	PCP040225	XM_018648639
PCP040264	7,44567705	6,714245518	7,018700931	7,115511897	PCP040264	XM_008234567
PCP040283	7,781359714	7,725673	7,984874125	8,596189756	PCP040283	XM_009364016
PCP040381	7,196430001	7,132679654	6,68187088	6,559644763	PCP040381	XM_009342554
PCP040406	7,183188734	7,285402219	7,448488033	7,822284016	PCP040406	XM_008341616
PCP040423	7,351645995	7,609769734	7,321928095	7,348728154		
PCP040455	8,483815777	8,356099782	8,447083226	8,835008208	PCP040455	XM_009339859
PCP040467	8,073231127	7,942514505	7,890750668	7,832890014		
PCP040479	7,845490051	8,073231127	8,300718622	8,522267655	PCP040479	XM_009359277
PCP040490	8,434628228	8,437419184	8,522267655	8,986780664		
PCP040532	5,448570626	6,64385619	7,515699838	8,283875484	PCP040532	XM_009358905
PCP040533	8,390899794	8,273002948	8,345760055	7,996219247	PCP040533	XM_009381229
PCP040549	8,283875484	8,318904285	8,379378367	8,764871591		

PCP040636	8,76927586	8,655816908	8,606109055	7,918863237	PCP040636	XM_009379206
PCP040702	8,541754876	8,199672345	8,463524373	8,588714636		
PCP040722	8,21106088	8,320439548	8,493174961	8,770399609		
PCP040723	8,271463028	8,188193194	8,514398461	9,236014192		
PCP040735	7,956114749	8,230404783	8,285402219	8,708497652		
PCP040749	7,453929061	7,327956767	7,705079392	7,866228001	PCP040749	XM_018645941
PCP040752	7,73470962	7,878541438	7,916894583	8,348728154		
PCP040754	8,110248388	8,220765252	8,426264755	8,70276142	PCP040754	XM_018650835
PCP040782	7,247927513	7,383704292	7,415065677	7,83080039		
PCP040800	7,798763389	8,022367813	8,073231127	7,882643049	PCP040800	XM_009361953
PCP040808	6,369291982	6,741466986	6,845490051	7,440204752	PCP040808	XM_009353217
PCP040837	8,282300792	8,309840108	8,288496992	8,062477937	PCP040837	XR_001954450
PCP040852	8,283875484	8,317412614	8,199672345	8,24474438	PCP040852	XM_009357134
PCP040854	8,660602089	8,462175047	8,568602197	7,963878669	PCP040854	XM_009357143
PCP040857	7,330916878	7,273049587	7,176621973	7,847558521		
PCP040864	7,523561956	7,291585141	7,589988142	7,215969746	PCP040864	XM_018649171
PCP041086	8,260543232	8,075051225	8,45532722	8,640244936		
PCP041092	6,279285561	6,619559738	7,139551352	7,400879436		
PCP041095	7,660566438	7,843418611	7,709635275	8,269921462		
PCP041108	7,06608919	7,136170875	7,423494135	7,730164413		
PCP041110	8,497173232	8,524855097	8,491853096	8,373604714	PCP041110	XM_018650861
PCP041133	7,741466986	7,536596918	7,665335917	7,28845085	PCP041133	XM_009351209
PCP041178	8,148069774	8,222408523	8,323415108	8,83709175		
PCP041190	8,212715235	8,533991546	8,481113232	8,487840034	PCP041190	XM_017324838
PCP041225	8,335390355	8,057071136	7,958088658	7,395148508	PCP041225	XM_017324555
PCP041351	7,672425342	7,754887502	7,90285744	8,479780264		
PCP041363	7,862141394	7,928784831	8,173277373	8,75598914		
PCP041367	7,080337882	7,111970261	7,033423002	6,297741678	PCP041367	XM_009356246
PCP041398	8,260543232	8,06786455	7,963878669	7,63175911		
PCP041414	7,415065677	7,592457037	7,674757228	8,345760055		
PCP041420	7,389480771	7,584962501	7,732472726	8		
PCP041448	8,607330314	8,618385502	8,891783703	9,077696975		
PCP041466	7,604886762	7,260496187	7,336908182	7,090959258	PCP041466	XM_009361580
PCP041476	8,452570726	7,798763389	7,222360218	5,922911406	PCP041476	XM_009361011
PCP041552	8,568602197	8,763776717	8,639051236	8,482485305		
PCP041593	7,754887502	7,837123296	7,886733033	8,58747751		
PCP041594	7,87036472	7,677155841	7,934693407	8,478446064		
PCP041601	8,305286574	8,124121312	7,984874125	8,196380818	PCP041601	XM_018647922
PCP041602	7,752681699	7,604886762	8,130982335	7,752681699	PCP041602	XM_009360150
PCP041627	8,158205728	7,928784831	7,934693407	7,832890014	PCP041627	XM_009346364
PCP041653	8,740354199	8,513056419	8,312882955	8,357552005	PCP041653	XM_018642464
PCP041668	8,338379842	8,518338423	8,376472723	8,767091963	PCP041668	XM_018652182
PCP041682	7,83080039	7,942514505	8,020535537	8,369248353		
PCP041704	8,305286574	8,225593068	8,181549844	8,615923847	PCP041704	XM_009337029
PCP041716	6,737010678	7,108524457	7,173227395	7,648681141	PCP041716	XM_009344681
PCP041722	8,105018005	8,176572111	8,430452552	8,263644792	PCP041722	XM_017325659

PCP041732	7,383704292	7,730164413	7,860031646	8,005624549	PCP041732	XM_008385106
PCP041810	7,166615031	7,291585141	7,520972191	7,21916852		
PCP041884	7,900866808	7,470292816	7,526147081	7,818070833	PCP041884	XM_009359617
PCP041895	7,592457037	7,375039431	7,300764373	6,768184325	PCP041895	XM_009353226
PCP041928	7,327956767	6,906890596	6,938638658	7,572359168		
PCP041960	7,475733431	7,660566438	7,930737338	8,125826717	PCP041960	XM_009376926
PCP041961	8,813781191	9,082149041	8,83709175	8,416459769	PCP041961	XM_009376928
PCP041983	7,900866808	7,855927425	8,089212109	8,903881846	PCP041983	XM_009378436
PCP042034	8,611024797	8,460783512	8,593689902	8,320439548		
PCP042070	8,016808288	8,094500005	7,851749041	8,13442632	PCP042070	XM_009370055
PCP042071	7,971543554	8,125826717	8,189824559	8,464872438		
PCP042073	8,583722152	8,526186214	8,568602197	9,066977144	PCP042073	XM_008390597
PCP042074	8,154818109	8,48651327	8,955155849	8,453970202		
PCP042088	7,641473777	7,864186145	7,6794801	8,438791853		
PCP042094	8,553322101	8,84024291	8,412231097	8,532667615	PCP042094	XM_009380591
PCP042103	8,08390476	8,204571144	8,251103639	8,581200582	PCP042103	XM_009339805
PCP042213	7,303780748	7,14974712	7,17990909	7,944448531	PCP042213	XM_009336946
PCP042217	9,896332404	4,564987801	10,45258102	10,03754695	PCP042217	XM_009337215
PCP042258	8,510447709	8,58747751	8,845490051	9,343563277	PCP042258	XM_009367932
PCP042261	8,481113232	8,214319121	8,269921462	8,385129006	PCP042261	XM_018649610
PCP042277	7,118941073	7,567347696	7,90285744	8,204571144	PCP042277	XM_009351526
PCP042310	6,564835417	7,136170875	7,294620749	8,232037117		
PCP042314	7,247927513	7,456724026	7,330916878	7,956114749	PCP042314	XM_009342089
PCP042328	7,033423002	7,08395793	7,626950122	7,745976779	PCP042328	XM_009380796
PCP042349	6,878602742	6,845490051	7,06608919	7,599912842	PCP042349	XR_525318
PCP042351	7,936637939	8,164906927	8,277659361	8,447083226	PCP042351	XM_018643660
PCP042358	6,686500527	6,99242856	7,06608919	7,429030064	PCP042358	XM_009361986
PCP042396	7,73470962	7,752681699	7,803033252	7,462134139		
PCP042521	7,285402219	7,400879436	7,475733431	7,839203788	PCP042521	XM_009367260
PCP042569	7,241554209	7,354646096	7,567347696	8,146339557	PCP042569	XM_008395281
PCP042579	7,73470962	7,787902559	7,988684687	7,662988741	PCP042579	XM_009343460
PCP042588	7,14974712	6,882643049	6,86628983	6,60481265		
PCP042593	7,707359132	8,130982335	8,594959026	8,087462841	PCP042593	XM_008354721
PCP042610	8,189824559	7,866228001	8,014968933	8,471675214	PCP042610	XM_009354535
PCP042692	6,718772592	6,857980995	6,99242856	7,354646096	PCP042692	XM_018651811
PCP042760	7,318949464	7,037052702	7,153095972	6,820178962		
PCP042784	8,181549844	8,348728154	8,318904285	8,744833837	PCP042784	XM_009374997
PCP042785	7,193081765	7,360452072	7,333870928	7,792269724	PCP042785	XM_008390263
PCP042790	8,736976865	8,64385619	8,584962501	8,984902599	PCP042790	XM_009375007
PCP042804	8,005624549	7,841281284	7,839203788	7,886733033	PCP042804	XM_009354742
PCP042809	6,772545519	6,902917719	7,146390476	7,779194046	PCP042809	XM_009354755
PCP042825	8,673591756	8,460783512	8,564797319	8,367764188		
PCP042839	6,798698597	7,029784146	7,254272787	7,541716163	PCP042839	XM_018648549
PCP042842	8,19313106	7,609769734	6,658211483	6,80309785	PCP042842	XM_009363246
PCP042861	8,520932916	8,696967526	8,792237182	9	PCP042861	XM_009360772
PCP042866	6,874428132	7,076815597	7,330916878	7,718841075	PCP042866	XM_009340417

PCP042881	8,452570726	8,74819285	8,77478706	8,597419437	PCP042881	XM_009362261
PCP042887	6,807354922	6,768184325	6,853870927	7,412188747	PCP042887	XM_018642533
PCP042928	8,122413888	8,536558066	8,988684687	8,951284715		
PCP042957	6,709704193	6,662917555	6,857980995	7,389480771	PCP042957	XM_018645208
PCP042990	7,686500527	7,718841075	8,112022407	8,315919398		
PCP043012	7,327956767	7,403693786	7,820178962	8,296136161		
PCP043067	6,47037417	6,579994693	6,667750232	8,577428828	PCP043067	XM_008378443
PCP043082	8,342830273	8,468949809	8,513056419	8,423452115	PCP043082	XM_009381247
PCP043086	7,900866808	8,166564822	8,125826717	8,046196362	PCP043086	XM_009381250
PCP043119	8,317412614	8,335390355	8,453970202	8,30833903	PCP043119	XM_009336650
PCP043122	8,202956379	8,144658243	8,082149041	8,051643995		
PCP043194	7,912889336	8,003770871	8,148069774	8,50779464	PCP043194	XM_009368742
PCP043197	6,815959618	7,111970261	7,238404739	7,499845887	PCP043197	XM_009370315
PCP043208	8,17990909	8,350187812	8,62450305	9,137836494		
PCP043233	7,473056289	7,594921715	7,541716163	7,398059585	PCP043233	XM_009360740
PCP043235	8,398016818	8,020535537	8,163247124	7,988684687	PCP043235	XR_001953390
PCP043237	6,878602742	7,300764373	7,209453366	7,928784831		
PCP043247	8,204571144	8,422064766	8,727920455	8,360495967	PCP043247	XM_009363762
PCP043248	7,845490051	7,83080039	8,342830273	7,942514505	PCP043248	XM_008377166
PCP043376	8,78571401	8,494535617	8,637820329	8,678318438	PCP043376	XM_009337072
PCP043393	7,73470962	7,494495616	7,691185174	8,202956379	PCP043393	XM_008368680
PCP043435	8,057071136	8,168270966	8,390899794	8,851749041	PCP043435	XM_009336960
PCP043444	8	8,060695932	8,161535025	8,54689446	PCP043444	XM_009372668
PCP043464	7,851749041	8,33391564	8,631795481	9,214319121	PCP043464	XM_018644887
PCP043487	8,416459769	7,684187561	7,868328714	7,92677039	PCP043487	XM_009356557
PCP043511	7,069637728	6,942514505	7,309885571	7,684187561	PCP043511	XM_009351738
PCP043535	8,209453366	7,849686575	8,283875484	8,523561956		
PCP043651	9,003742767	8,576181982	8,694636474	8,602401945	PCP043651	XM_009346286
PCP043692	8,612241904	8,854868383	8,698114274	8,41079052	PCP043692	XM_008391583
PCP043742	6,981053471	6,777025123	7,235248379	7,604886762		
PCP043760	8,589950702	8,696967526	8,865207434	9,054387253	PCP043760	XM_009342303
PCP043799	7,920829209	7,888743249	8,033423002	8,562242424	PCP043799	XM_008364744
PCP043803	7,183188734	7,115511897	7,330916878	7,798763389	PCP043803	XM_008356208
PCP043827	8,212715235	8,247927513	8,459431619	8,726797165		
PCP043929	7,251056285	7,440204752	7,958088658	8,920858975	PCP043929	XM_009346160
PCP043944	8,385129006	8,318904285	8,32494558	8,22881869	PCP043944	XM_009358067
PCP043998	8,382278171	8,339850003	8,376472723	8,279239123	PCP043998	XM_009346037
PCP044010	8,291539098	8,489165579	8,678318438	8,937609223	PCP044010	XM_009341279
PCP044014	7,732472726	7,674757228	7,952275317	8,372125027	PCP044014	XM_017324398
PCP044016	8,73470962	8,689997971	8,794415866	8,395191361		
PCP044032	6,420718183	6,768184325	6,828707735	7,834976616		
PCP044074	8,022367813	8,103287808	7,946438456	7,741466986	PCP044074	XM_018650796
PCP044082	7,936637939	7,956114749	7,874489611	7,741466986		
PCP044097	7,108524457	7,231989133	7,254272787	7,026025399	PCP044097	XM_009376826
PCP044101	7,312882955	7,324900589	7,626950122	8,159871337	PCP044101	XM_017336642
PCP044104	7,209453366	7,533952624	7,617136829	7,888743249	PCP044104	XM_008355678

PCP044108	7,087462841	7,146390476	7,470292816	7,843418611		
PCP044125	7,969645536	8,064311643	8,198052084	8,545620932	PCP044125	XM_008384161
PCP044126	8,062477937	7,569855608	7,577428828	7,315874125	PCP044126	XR_667722
PCP044164	8,449850215	8,670054263	8,809510912	9,327170869	PCP044164	XM_009335751
PCP044166	8,318904285	8,468949809	8,670054263	9,193944186	PCP044166	XR_527174
PCP044170	7,225641265	7,787902559	7,727920455	7,944448531		
PCP044174	6,363346321	7,022367813	7,64385619	7,312882955	PCP044174	XM_009359741
PCP044181	7,231989133	7,884658968	7,584962501	7,705079392	PCP044181	XM_004290967
PCP044190	7,691185174	7,266786541	7,730164413	7,409390936	PCP044190	XM_018647470
PCP044191	7,569855608	7,757089938	7,813781191	8,326429487	PCP044191	XM_009358160
PCP044194	8,009380767	8,082149041	8,379378367	8,48651327	PCP044194	XM_009381252
PCP044202	8,28077077	8,431831861	8,519636253	9,319672121		
PCP044209	8,320439548	8,32494558	8,388017285	8,214319121	PCP044209	XM_009359504
PCP044211	6,50779464	6,906890596	6,768184325	7,494495616		
PCP044233	7,837123296	8	7,562242424	7,811663685		
PCP044243	8,30833903	8,370687407	8,326429487	8,268331458		
PCP044244	7,88062431	8,031604721	8,42488011	8,828676005	PCP044244	XM_008376437
PCP044247	7,641473777	7,655852677	7,727920455	7,153095972	PCP044247	XM_009375680
PCP044251	6,832890014	6,946379968	6,996275749	6,691115365		
PCP044257	7,417852515	7,54689446	7,604886762	8,178266468	PCP044257	XM_017327410
PCP044258	7,866228001	8,300718622	7,958088658	8,255831156	PCP044258	XM_009364791
PCP044259	8,321928095	8,613494819	8,618385502	8,474395481	PCP044259	XM_009346513
PCP044267	7,912889336	8,129283017	8,241601875	8,550746785	PCP044267	XM_009343248
PCP044285	7,725673	7,462134139	7,890750668	8,091012169	PCP044285	XM_018644750
PCP044287	7,700439718	7,327956767	7,8008999	7,906890596	PCP044287	XM_009376949
PCP044302	8,164906927	8,255831156	8,227230852	8,639051236	PCP044302	XM_008353698
PCP044312	7,785746699	7,811663685	7,813781191	8,342830273		
PCP044321	8,558420713	8,562242424	8,645045922	8,148069774		
PCP044324	7,456724026	7,660566438	7,572359168	8,04072808		
PCP044337	8,24474438	8,294620749	8,118941073	7,8008999		
PCP044339	7,674757228	7,572359168	7,826548487	8,271463028		
PCP044346	8,164906927	8,299208018	8,453970202	8,74708524	PCP044346	XM_009348564
PCP044381	8,74708524	8,631795481	8,718806834	9,164077264	PCP044381	XM_008367459
PCP044419	8,330916878	8,255831156	8,416459769	8,21757002	PCP044419	XM_009362896
PCP044421	7,557119022	7,958088658	7,958088658	8,042589623	PCP044421	XM_009360882
PCP044435	8,076815597	7,693486957	8,058911723	7,942514505		
PCP044443	7,423494135	7,914863459	7,910912508	8,483815777	PCP044443	XM_009359381
PCP044445	8,613494819	8,447083226	8,540438054	8,361943774	PCP044445	XM_009359402
PCP044450	8,370687407	8,265239967	8,535275377	8,314379315	PCP044450	XM_009349315
PCP044467	8,246360579	7,794415866	8,43325159	9,164906927	PCP044467	XM_008348493
PCP044483	7,300764373	6,594996337	6,886672074	6,841344192	PCP044483	XM_009356970
PCP044500	7,670089681	7,92677039	8,042589623	8,412231097	PCP044500	XM_009380416
PCP044504	7,040782866	7,351645995	7,285402219	7,721099189		
PCP044509	7,206232954	7,50247382	7,539158811	7,92677039	PCP044509	XM_009376680
PCP044516	7	7,186559982	7,176621973	7,718841075	PCP044516	XM_009371943
PCP044524	6,785681319	7,003714662	8,353146825	9,545601627	PCP044524	XM_009346628

PCP044541	8,321928095	8,118941073	8,533991546	8,74708524	PCP044541	XM_009343682
PCP044542	7,199672345	6,890811455	7,108524457	6,996275749		
PCP044544	6,475733431	6,853870927	7,139551352	7,639015048		
PCP044556	7,037052702	6,459431619	7,22881869	7,564759219	PCP044556	XM_008388478
PCP044558	6,714245518	7,497213158	7,231989133	7,339850003	PCP044558	XM_009345461
PCP044572	7,42063398	7,266786541	7,464913269	7,904905525	PCP044572	XM_009369409
PCP044574	8,951284715	8,934663924	8,897845456	8,395191361	PCP044574	XM_018642524
PCP044587	7,434628228	7,475733431	7,914863459	8,260543232	PCP044587	XM_009370515
PCP044595	7,448488033	7,569855608	7,342785837	7,330916878	PCP044595	XM_008364727
PCP044616	8,314379315	7,984874125	8,136119594	8,677120596	PCP044616	XM_009348866
PCP044637	8,001858526	8,419243918	8,659389441	8,724513853	PCP044637	XM_009336142
PCP044701	8,451211112	8,510447709	8,609806663	9,182394353	PCP044701	XM_008378652
PCP044704	8,481113232	8,431831861	8,470333494	8,353146825	PCP044704	XM_004309761
PCP044735	8,372125027	7,960001932	8,014968933	7,750405521	PCP044735	XM_017333919
PCP044738	7,047996356	7,339850003	7,453929061	7,711976642	PCP044738	XM_009337521
PCP044743	7,209453366	7,080337882	7,348728154	7,736943052		
PCP044768	7,478405614	7,577428828	7,851749041	8,153146652	PCP044768	XM_009377950
PCP044769	7,044394119	7,50247382	7,369204723	7,209453366		
PCP044813	7,456724026	7,30679083	7,549438149	7,868328714	PCP044813	XM_009361301
PCP044818	7,132679654	7,377904593	7,811663685	8,148069774		
PCP044856	7,660566438	8,018645301	8,049848549	7,88062431	PCP044856	XM_008395262
PCP044870	7,14974712	7,069637728	7,238404739	6,938638658	PCP044870	XR_001952024
PCP044873	7,811663685	7,761551232	7,768184325	8,279239123		
PCP044892	7,754887502	7,212666605	7,360452072	7,95419631	PCP044892	XM_009361144
PCP044900	6,934634441	7,159871337	7,156538193	6,431790083	PCP044900	XM_009354508
PCP044907	8,42488011	8,28692734	8,315919398	8,233619677		
PCP044909	7,28845085	6,914923239	7,674757228	7,300764373		
PCP044921	6,672425342	6,574858391	7,173227395	7,383704292	PCP044921	XM_009344313
PCP044927	7,122362117	7,014913158	7,423494135	7,122362117	PCP044927	XM_009377240
PCP044930	7,357552005	7,665335917	7,862141394	8,356099782		
PCP044943	8,207844058	8,323415108	8,520932916	8,739206792	PCP044943	XM_009346494
PCP044974	7,847558521	7,845490051	8,011227255	8,815927606		
PCP044978	6,99242856	7,510408147	7,83080039	8,84024291	PCP044978	XM_008225340
PCP044981	8,071462363	8,158205728	8,227230852	8,665335917	PCP044981	XM_008388096
PCP044987	7,904905525	8,112022407	7,872397856	7,646234675		
PCP045009	7,279192684	7,752681699	8,139551352	8,588714636		
PCP045017	7,026025399	6,857980995	7,241554209	7,594921715		
PCP045036	7,372168569	7,494495616	7,456724026	8,112022407	PCP045036	XM_009341546
PCP045045	7,14974712	7,163196797	7,333870928	7,90285744	PCP045045	XM_009375737
PCP045056	8,357552005	8,220765252	8,407989993	8,705044823	PCP045056	XM_009373030
PCP045091	8,336863563	7,922851957	8,127581695	8,238404739	PCP045091	XM_009349238
PCP045103	6,677085351	7,029784146	7,238404739	7,587440004		
PCP045120	7,276124405	7,453929061	7,515699838	7,276124405	PCP045120	XM_008377151

Cluster_7

Gene	H1	H2	H3	H4		
PCP000117	3,368768349	2,321928095	1,735522177	-0,577766999	PCP000117	XM_018651203
PCP000118	4,321928095	2,939226578	1,416839742	1,875780063		
PCP000123	2,503348735	3,544732656	2,873813198	0,739848103	PCP000123	XM_008375329
PCP000167	3,459431619	2,584962501	2,321928095	-0,577766999		
PCP000349	3,368768349	2,939226578	2,503348735	0	PCP000349	XM_018650428
PCP000403	3,502075956	2,321928095	2,114367025	1	PCP000403	XM_008341822
PCP000531	3,772941338	3	2,321928095	1,735522177	PCP000531	XM_009360571
PCP000834	3,415488271	1,584962501	1,735522177	0	PCP000834	XR_668649
PCP000924	3,27351589	2,414135533	1,735522177	0	PCP000924	XM_017334345
PCP001002	2,22342255	3,624100895	3,700439718	1	PCP001002	XM_009357157
PCP001203	1,416839742	3,058316496	2,321928095	0		
PCP001279	4,392317423	3,169925001	1,735522177	0	PCP001279	XM_018652359
PCP002222	3,736604875	4	2,22342255	0,739848103	PCP002222	XM_009343022
PCP002546	3,544732656	3,368768349	3,058316496	1	PCP002546	XR_001951292
PCP002548	3,772941338	2,414135533	1,735522177	1	PCP002548	XM_009361676
PCP002659	3,874796966	4,029452886	1,584962501	-0,577766999	PCP002659	XM_009360713
PCP002710	4,222650022	2,939226578	2,807354922	0		
PCP003210	3,058316496	0	2,807354922	1,220329955	PCP003210	XR_001952457
PCP003557	4,029452886	3,169925001	2,503348735	0	PCP003557	XM_009351112
PCP003749	0,739848103	5,513174885	2,503348735	1	PCP003749	XM_008376256
PCP004287	1,220329955	5,058749412	2,737686761	1,220329955	PCP004287	XM_009371711
PCP004383	0	4,272769732	5,309976492	0		
PCP005221	3,459431619	1,584962501	3,368768349	0,739848103	PCP005221	XM_009340854
PCP005383	4,544114402	3,169925001	1	1,220329955	PCP005383	XM_009375906
PCP005698	2,503348735	2	2,737686761	0	PCP005698	XM_008357087
PCP006023	2,737686761	2,22342255	2,321928095	0	PCP006023	XM_009365560
PCP006133	3,368768349	2,807354922	2,321928095	0	PCP006133	XM_009340436
PCP006480	3,221877081	2,22342255	2,114367025	0	PCP006480	XM_008395200
PCP006656	3,772941338	3,058316496	2,503348735	1,584962501	PCP006656	XM_008366386
PCP007117	4,059182199	1	2,503348735	1	PCP007117	XM_009343165
PCP007178	1,735522177	3,27351589	2,584962501	0		
PCP007297	3,058316496	2,807354922	2,939226578	0	PCP007297	XM_008375174
PCP007847	3,169925001	2,807354922	1,735522177	-1,59946207	PCP007847	XM_018644501
PCP007969	3,840966704	2,873813198	1,735522177	0,411426246		
PCP008058	2,114367025	2,873813198	1,875780063	0	PCP008058	XM_008359291
PCP008288	3,27351589	3	2,321928095	0	PCP008288	XM_009363996
PCP008575	2,321928095	2,6622055	1,735522177	0	PCP008575	XM_018648080
PCP008875	3,321928095	3,169925001	2,6622055	0	PCP008875	XM_017335268
PCP009103	3,415488271	4,143230135	2,807354922	0	PCP009103	XM_009365974
PCP009216	3,874796966	2,22342255	2,873813198	1	PCP009216	XM_009342138
PCP009336	3,116031993	2,873813198	2,737686761	0	PCP009336	XM_009365146
PCP009469	4,029452886	2,584962501	2,22342255	0,739848103	PCP009469	XM_008373381
PCP009536	4,297925053	2,6622055	2,584962501	0		

PCP009585	3,969933275	2,737686761	2,503348735	0		
PCP009808	2,873813198	2,584962501	1,416839742	0	PCP009808	XM_018646706
PCP009922	4,480911346	3,840966704	2,22342255	-0,577766999		
PCP010004	2,807354922	2,737686761	1,735522177	0	PCP010004	XM_009364978
PCP010006	0,739848103	3,116031993	3,969933275	0,411426246	PCP010006	XM_009364980
PCP010035	3,772941338	3,27351589	2	0,411426246	PCP010035	XM_009365013
PCP010109	2,6622055	1,735522177	2,114367025	0	PCP010109	XM_009357461
PCP010700	2,503348735	1	2,737686761	0	PCP010700	XM_008353476
PCP010818	1,875780063	4,73714592	4,169925001	0	PCP010818	XM_009363629
PCP011347	3,840966704	2,584962501	3,116031993	0,411426246	PCP011347	XM_008339178
PCP011385	3,700439718	3,27351589	2,584962501	1,220329955	PCP011385	XM_008354048
PCP011392	2,503348735	6,420718183	1,416839742	0	PCP011392	XM_006450957
PCP012201	2,584962501	1,416839742	2,737686761	0		
PCP012344	3,368768349	3,169925001	2,22342255	-0,577766999	PCP012344	XM_018651941
PCP012560	3,459431619	2,737686761	1,220329955	0	PCP012560	XM_009336602
PCP012676	3,502075956	2,6622055	2,414135533	0	PCP012676	XM_009361149
PCP013044	2,873813198	2,503348735	2,737686761	0		
PCP013424	2,414135533	3,584962501	2,584962501	1	PCP013424	XM_009370823
PCP013616	3,736604875	2,873813198	1,416839742	1	PCP013616	XM_009369621
PCP013697	3,415488271	1,220329955	3,169925001	0,739848103	PCP013697	XM_018645755
PCP014128	3,169925001	3,584962501	1,875780063	0,411426246	PCP014128	XM_009359187
PCP014734	3,700439718	0,411426246	2,737686761	0	PCP014734	XM_009366288
PCP014789	3,544732656	1,416839742	2	-0,577766999	PCP014789	XM_009370288
PCP015476	4,029452886	3,169925001	2,584962501	1,220329955		
PCP015752	3,969933275	3,584962501	2,22342255	0	PCP015752	XM_009354740
PCP015762	3,459431619	1,416839742	1,735522177	0,739848103	PCP015762	XM_008387269
PCP017539	3,663344619	3,415488271	2,939226578	1	PCP017539	XM_009353124
PCP018019	4,247927513	3,368768349	2,6622055	0		
PCP018119	3,459431619	3	1,416839742	0,739848103	PCP018119	XM_009358338
PCP018239	0	5,309976492	2,321928095	2	PCP018239	XM_009361266
PCP019102	3,27351589	1,584962501	2	0,739848103	PCP019102	XM_009340088
PCP020459	3,116031993	1,416839742	2,114367025	0	PCP020459	XM_018650951
PCP020578	3,27351589	2,584962501	1,584962501	0,411426246		
PCP020869	3,807354922	2,114367025	2,321928095	1,220329955	PCP020869	XM_018652120
PCP021247	2	3,584962501	2,6622055	1	PCP021247	XM_018646539
PCP021319	2,873813198	3	3,584962501	0	PCP021319	XM_009345566
PCP021707	3,624100895	2,584962501	2,503348735	1,416839742	PCP021707	XM_009348714
PCP021810	2,939226578	2,22342255	1,735522177	0	PCP021810	XM_009340481
PCP022102	3	3,058316496	2,22342255	0	PCP022102	XM_009361516
PCP022128	4,480911346	2,6622055	1,584962501	0	PCP022128	XM_008362333
PCP022356	3,415488271	2,584962501	2,321928095	-0,577766999	PCP022356	XM_009366828
PCP022615	6,339850003	5,260402093	-0,577766999	-1,59946207		
PCP022719	1,584962501	5,961854808	1,416839742	2,873813198		
PCP022859	3,584962501	3,116031993	1	0		
PCP023637	5	4,414812061	0	0,411426246	PCP023637	XM_009377498
PCP023650	3,502075956	2,873813198	2,22342255	0	PCP023650	XM_018649835

PCP023806	1,416839742	3,415488271	3,27351589	0	PCP023806	XM_009356771
PCP024063	2,414135533	3,736604875	3,700439718	0,739848103		
PCP024366	4,437627248	3,772941338	2,873813198	0		
PCP024557	4,857980995	2,807354922	0,411426246	2,584962501	PCP024557	XM_009379548
PCP024618	3,807354922	2,807354922	1,875780063	2	PCP024618	XM_017323630
PCP024956	0,739848103	3,938285792	3,27351589	1,875780063	PCP024956	XM_008392607
PCP025052	3,368768349	2,321928095	1,220329955	0		
PCP025059	2,737686761	5,297558281	1,735522177	0,739848103	PCP025059	XM_009346316
PCP025368	3,502075956	3,736604875	3	0	PCP025368	XM_008379041
PCP026470	2,6622055	3,459431619	4,34553831	0		
PCP026736	2	2,737686761	2	0	PCP026736	XM_008344463
PCP026910	2,939226578	3,544732656	2,321928095	0	PCP026910	XM_018651334
PCP026997	3,27351589	2,414135533	2,321928095	0	PCP026997	XM_009347329
PCP027414	3,169925001	4,143230135	3,840966704	-0,577766999	PCP027414	XM_009379125
PCP028160	3	2,807354922	1,735522177	0	PCP028160	XM_009361397
PCP028277	2,503348735	1,735522177	2,114367025	0		
PCP029766	3,221877081	3,700439718	1,416839742	0,411426246	PCP029766	XM_009339120
PCP030782	4,059182199	3,058316496	2,414135533	0		
PCP030819	4,502712486	2,321928095	1,220329955	0	PCP030819	XM_008346593
PCP031001	2,6622055	2,414135533	2,807354922	0	PCP031001	XM_008367346
PCP032188	3,321928095	2,503348735	2,22342255	0	PCP032188	XM_008379456
PCP032802	3,415488271	2	3,368768349	0,411426246	PCP032802	XM_009340838
PCP033131	3,27351589	3	1,584962501	0,411426246	PCP033131	XM_009367366
PCP034125	4,544114402	0	3,874796966	0	PCP034125	XM_009356910
PCP034272	3,27351589	3,116031993	3	0		
PCP034358	3,874796966	3,321928095	1,416839742	1,875780063	PCP034358	XM_009345685
PCP034897	3,321928095	1,875780063	1,735522177	0	PCP034897	XM_018646965
PCP035959	3,584962501	1,584962501	2,6622055	0,411426246	PCP035959	XM_009348483
PCP036209	4,564987801	3,840966704	1	1	PCP036209	XM_009369309
PCP036392	4,169925001	2,737686761	1,735522177	0		
PCP036471	2,584962501	2,114367025	2,321928095	0		
PCP036488	3,840966704	3,544732656	2,22342255	1		
PCP036833	3,906890596	3	2,114367025	0,411426246	PCP036833	XM_009364743
PCP037168	2	2,737686761	2,22342255	0		
PCP037435	2,503348735	3,368768349	2,414135533	0	PCP037435	XM_009373783
PCP038753	3,874796966	3,058316496	3	1	PCP038753	XM_009352739
PCP038901	3,415488271	2,114367025	2,114367025	-0,577766999	PCP038901	XM_008390889
PCP039947	3	2,503348735	2	-0,577766999		
PCP040285	3,663344619	3,415488271	2,584962501	0,411426246	PCP040285	XR_524702
PCP040769	4,321928095	3,624100895	3,058316496	0		
PCP042186	4,222650022	3,459431619	1,416839742	1,584962501		
PCP042372	3,772941338	1,416839742	2,939226578	1,735522177		
PCP042625	0	4,985044962	6,619559738	0	PCP042625	XM_009336751
PCP043140	0	7,076815597	1,220329955	1,735522177		
PCP043254	0	3,502075956	4,321928095	0	PCP043254	XR_669773
PCP043630	2,114367025	4,34553831	2,503348735	2,321928095	PCP043630	XM_009373950

PCP043930	2,807354922	3,700439718	3,544732656	0,411426246		
PCP044442	3,624100895	2,6622055	1,735522177	0,411426246	PCP044442	XM_017331535
PCP044456	3,058316496	3,736604875	2,939226578	0	PCP044456	XM_008390352
PCP044510	3,700439718	1,584962501	3,415488271	0,411426246	PCP044510	XM_018649265
PCP044594	3,624100895	3,459431619	2,321928095	0	PCP044594	XM_009366112
PCP044786	1,584962501	6,534030467	1,875780063	1,735522177	PCP044786	XM_007226218
PCP044894	2,321928095	1,416839742	2,22342255	0		
PCP044965	3,544732656	3,321928095	2,873813198	-0,577766999	PCP044965	XM_009360005

Cluster_8

Gene	H1	H2	H3	H4		
PCP000019	4,841469999	3,938285792	4,087462841	3,624100895	PCP000019	XR_665912
PCP000056	4,297925053	2,584962501	3,27351589	2,6622055	PCP000056	XM_009350701
PCP000092	5,459431619	4,029452886	3,736604875	2,6622055	PCP000092	XM_008375294
PCP000114	4,247927513	3,116031993	3,700439718	2,807354922		
PCP000171	4,272769732	3,906890596	3,700439718	2,807354922	PCP000171	XM_008381119
PCP000193	4,34553831	3,415488271	3,700439718	2,737686761	PCP000193	XM_018643791
PCP000387	4,34553831	3,906890596	2,939226578	2,6622055	PCP000387	XM_008341840
PCP000568	4,841469999	4,790250739	4,34553831	2,807354922		
PCP000740	3,906890596	4,392317423	3,906890596	2,503348735	PCP000740	XM_009340769
PCP000874	4,544114402	3,415488271	2,873813198	2,939226578		
PCP000907	4,321928095	4,369466484	2,6622055	3	PCP000907	XM_008372851
PCP000917	5,142821844	3,772941338	4,247927513	1,416839742		
PCP000920	4,222650022	4,059182199	3,938285792	2,584962501	PCP000920	XM_017325918
PCP000942	4,459431619	4,392317423	3,584962501	2,807354922		
PCP000950	4,604664415	4,857980995	3,938285792	3,663344619	PCP000950	XM_018651193
PCP001519	3,700439718	4,321928095	3,502075956	2,114367025	PCP001519	XM_008345255
PCP001655	5,115615931	4,890933022	4,662775172	3	PCP001655	XM_009355878
PCP001855	4,369466484	2,584962501	3,169925001	1,416839742		
PCP001865	3,221877081	5,681730355	2,584962501	3	PCP001865	XM_009369970
PCP001957	4,143230135	5,260402093	4,584962501	4,196134881	PCP001957	XM_009374565
PCP001968	4,115199749	3,663344619	2,6622055	2,584962501	PCP001968	XM_009374586
PCP002022	4,087462841	3	2,114367025	2,503348735	PCP002022	XM_017328092
PCP002044	4,392317423	4,115199749	3,459431619	3,058316496	PCP002044	XM_008349271
PCP002172	4,297925053	4,584962501	4,624685811	2,939226578	PCP002172	XM_017333443
PCP002193	5,115615931	4,247927513	3,502075956	3,459431619	PCP002193	XM_009375613
PCP002332	4,874305166	3,502075956	4,584962501	1,735522177		
PCP002830	4,682011391	4,682011391	1,584962501	1,584962501	PCP002830	XM_018647411
PCP002867	5,554588852	4,544114402	3,736604875	2,873813198	PCP002867	XM_009344743
PCP002933	5,345893086	4,807354922	4,297925053	2,807354922	PCP002933	XM_009369332
PCP003049	3,969933275	5,866166169	4,604664415	3,415488271	PCP003049	XM_009356577
PCP003123	4,824258697	4,272769732	4,682011391	3,663344619	PCP003123	XM_008394017
PCP003322	5,222263604	4,392317423	3,584962501	2,321928095		
PCP003399	4,938756261	3,624100895	4,392317423	3,116031993	PCP003399	XM_018645946
PCP003421	4,874305166	2,503348735	3,544732656	2,873813198	PCP003421	XM_009361215
PCP003533	4,459431619	4	3,663344619	3,169925001	PCP003533	XM_009378996

PCP003541	4,64385619	3,807354922	5,073391816	3,502075956	PCP003541	XM_009378406
PCP003729	6,163297449	4,115199749	3,368768349	3,700439718		
PCP003744	4,64385619	4,857980995	3,584962501	1,875780063	PCP003744	XM_008376346
PCP003967	3,938285792	4,059182199	2,939226578	1,220329955	PCP003967	XM_009362304
PCP004146	4,857980995	4,700439718	3,663344619	3,584962501	PCP004146	NM_001293944
PCP004208	4,143230135	2,414135533	2,737686761	1,584962501	PCP004208	XM_009355922
PCP004231	4,857980995	3,938285792	4,143230135	3,807354922	PCP004231	XM_018650080
PCP004560	4,754887502	4,564987801	4,321928095	3,584962501	PCP004560	XM_009378850
PCP004565	3,938285792	4,222650022	3,221877081	2,584962501		
PCP004586	3,906890596	3	4,544114402	2,584962501	PCP004586	XM_008350967
PCP004602	5,087462841	4,480911346	4,414812061	4,029452886	PCP004602	XM_008380666
PCP004664	2,807354922	4,272769732	1,875780063	2,6622055	PCP004664	XM_009370967
PCP004938	3,906890596	3,116031993	2,503348735	1,584962501	PCP004938	XM_008375294
PCP004965	4,414812061	4,34553831	4,34553831	3,221877081	PCP004965	XM_009338928
PCP004971	5,59484709	4,95419631	4,321928095	2,807354922	PCP004971	XM_018643119
PCP005471	4,414812061	4,523561956	4,169925001	3,169925001	PCP005471	XM_009346137
PCP005503	3,221877081	3,938285792	4,824258697	2,414135533	PCP005503	XM_009380633
PCP005506	4,369466484	4,115199749	4,059182199	2,939226578		
PCP005562	5,48123435	4,64385619	4,34553831	3,807354922	PCP005562	XR_001951281
PCP005564	4,222650022	3	2,873813198	2,414135533	PCP005564	XM_009354149
PCP005658	4,754887502	4,059182199	4,718635616	3,502075956		
PCP005661	4,790250739	3,840966704	4,414812061	2,503348735	PCP005661	XM_009370675
PCP005727	5,309976492	4,64385619	3,938285792	2,584962501	PCP005727	XM_009351469
PCP005749	4,059182199	3,700439718	4,772413555	3,27351589	PCP005749	XM_009357859
PCP005810	4,95419631	4,772413555	2,873813198	2,584962501	PCP005810	XM_009346180
PCP005904	3,459431619	5,380937195	4,544114402	3,840966704	PCP005904	XM_009368644
PCP006683	4,890933022	4,890933022	3,807354922	2,6622055		
PCP006786	5,273142859	3,969933275	4,523561956	3,663344619	PCP006786	XM_018646433
PCP006880	5,196528361	4,584962501	3,840966704	3,415488271		
PCP006949	2,584962501	6,732404887	3,058316496	3,058316496		
PCP006992	4,222650022	5,235344128	4,437627248	4,222650022	PCP006992	XM_018648503
PCP007180	4,564987801	3,663344619	3,415488271	3,321928095	PCP007180	XM_018646695
PCP007213	4,857980995	4,682011391	4,682011391	3,874796966	PCP007213	XM_017331851
PCP007237	5	4,906890596	4,73714592	3,058316496	PCP007237	XM_008379759
PCP007373	5,169925001	3,736604875	4,222650022	4,222650022		
PCP007595	5,260402093	5,044394119	4,059182199	3,27351589	PCP007595	XM_009373947
PCP007596	2,807354922	4,564987801	3,169925001	2,503348735	PCP007596	XM_009373950
PCP007614	3,736604875	3,415488271	3	1,584962501	PCP007614	XM_009363916
PCP007739	4,196134881	3,459431619	2,503348735	2,414135533	PCP007739	NM_001328842
PCP007769	4,662775172	4,247927513	4	1,584962501	PCP007769	XM_009349918
PCP007928	4,059182199	2,737686761	2	2,414135533	PCP007928	XM_018643513
PCP008041	3,736604875	3,321928095	2,737686761	1,416839742	PCP008041	XM_017333688
PCP008154	4,564987801	4,480911346	4,682011391	3,169925001	PCP008154	XM_018650554
PCP008158	3,700439718	3,169925001	3,058316496	1,220329955	PCP008158	XM_009372355
PCP008258	5,297558281	4,700439718	4,73714592	3,415488271		
PCP008276	4,369466484	3,502075956	3,700439718	2,6622055	PCP008276	XM_009363981

PCP008315	3,368768349	4,718635616	4,459431619	2,807354922	PCP008315	XM_008391439
PCP008534	4,564987801	3,840966704	4,087462841	3	PCP008534	XM_009381428
PCP008573	4,790250739	3,840966704	3,584962501	3,169925001	PCP008573	XM_009360970
PCP008851	4,222650022	3,459431619	2,939226578	2,584962501	PCP008851	XM_008377522
PCP008974	5,014801602	4,437627248	3,624100895	4,087462841	PCP008974	XM_008343268
PCP008979	4,754887502	3,772941338	4,459431619	3,169925001	PCP008979	XM_009367087
PCP008980	4,807354922	3,807354922	3,736604875	3,221877081		
PCP009121	5,369117459	4,95419631	4,247927513	3,700439718	PCP009121	XM_009373234
PCP009169	4,564987801	3,938285792	3,415488271	2,873813198	PCP009169	XM_009378024
PCP009172	4,564987801	3,969933275	4,196134881	3,058316496	PCP009172	XM_008342943
PCP009222	4,938756261	4,584962501	4,682011391	3,772941338	PCP009222	XM_017335744
PCP009257	5,297558281	4,196134881	4	2,414135533	PCP009257	XM_009340088
PCP009620	3,772941338	4,824258697	4,029452886	3,938285792	PCP009620	XM_009358661
PCP009832	5,866166169	5,57500972	4,247927513	2	PCP009832	XM_018644752
PCP009854	4,841469999	4,624685811	4,169925001	3,584962501	PCP009854	XM_009354783
PCP009883	4,906890596	4,029452886	3,772941338	3,368768349	PCP009883	XM_009357773
PCP009888	3,27351589	6,254178286	4,985044962	3,368768349		
PCP010056	2,737686761	6,279285561	4	2,584962501	PCP010056	XM_009350804
PCP010210	3,938285792	4,523561956	4,321928095	3,321928095		
PCP010220	3,624100895	4,059182199	3,415488271	2,414135533	PCP010220	XM_009373517
PCP010384	5,02989455	3,906890596	3,700439718	3		
PCP010400	4,392317423	2,584962501	3,27351589	2,22342255	PCP010400	XM_017327374
PCP010426	4,718635616	4,321928095	4,480911346	3,058316496		
PCP010452	4,247927513	3,368768349	2,939226578	2,807354922	PCP010452	XM_008378920
PCP010519	4,584962501	3,27351589	3,058316496	3,502075956	PCP010519	XM_008383244
PCP010597	4,087462841	3,584962501	2,22342255	2	PCP010597	NM_001328990
PCP010680	4,059182199	4,34553831	3,938285792	2,939226578	PCP010680	XM_009366247
PCP010930	4,222650022	4,754887502	4,029452886	3,544732656	PCP010930	XM_009339893
PCP011154	4,807354922	4,247927513	4,059182199	3,874796966	PCP011154	XM_008382537
PCP011203	4,169925001	4,459431619	4,969472865	3,874796966	PCP011203	XM_009368506
PCP011326	2,6622055	4,624685811	3,544732656	2,807354922	PCP011326	XM_009370598
PCP011342	4,64385619	4,95419631	2,873813198	2,22342255	PCP011342	XM_009370572
PCP011373	4,369466484	4,059182199	3,807354922	2,584962501	PCP011373	XM_009370538
PCP011394	4,938756261	4,874305166	4,790250739	3,27351589		
PCP011478	4,480911346	4,321928095	4,414812061	3,221877081	PCP011478	XM_009355207
PCP011480	5,044394119	3,906890596	4,95419631	4,196134881	PCP011480	XM_009355204
PCP011591	4,922673593	4,247927513	4,392317423	3,624100895	PCP011591	XM_009380050
PCP011636	4,459431619	3,840966704	2,22342255	4,34553831	PCP011636	XM_008341779
PCP011646	4,392317423	3,840966704	4,169925001	2,414135533	PCP011646	XM_008341797
PCP011753	4,718635616	3,116031993	3,874796966	3,415488271	PCP011753	XM_009336102
PCP011921	3,736604875	3,415488271	3,502075956	1,416839742		
PCP012000	4,115199749	4,624685811	4,938756261	3,700439718	PCP012000	XM_009359876
PCP012001	5,156639311	4,718635616	4,321928095	3,415488271	PCP012001	XM_009359877
PCP012075	4,392317423	3,772941338	3,544732656	2,6622055	PCP012075	XM_009343478
PCP012172	4,95419631	5,02989455	4,321928095	2,873813198	PCP012172	XM_009363678
PCP012227	5,169925001	5,984817174	4,807354922	1		

PCP012345	3,058316496	4,196134881	4,459431619	2,737686761		
PCP012359	5,470211457	4,841469999	3,906890596	2,6622055	PCP012359	XM_018649524
PCP012461	4,392317423	3,906890596	3,807354922	1	PCP012461	XM_009358873
PCP012540	4,906890596	4,222650022	4,059182199	3,415488271		
PCP012698	4,272769732	2	3,058316496	1,735522177	PCP012698	XM_008388294
PCP012702	3,874796966	4,624685811	3,906890596	2,321928095	PCP012702	XR_001954107
PCP012723	4,297925053	4,700439718	3,807354922	2,6622055		
PCP012899	4,874305166	4,34553831	4	3,906890596	PCP012899	XM_009367254
PCP012913	3,938285792	2,807354922	2,873813198	2,22342255	PCP012913	XM_009336415
PCP013351	3,874796966	3,969933275	0	4,34553831		
PCP013355	3,736604875	3,624100895	2,737686761	1,584962501	PCP013355	XM_009376285
PCP013432	4,64385619	4,115199749	3,058316496	3,544732656	PCP013432	XM_009342565
PCP013516	4,297925053	4,196134881	3,906890596	2,414135533	PCP013516	XM_009359275
PCP013622	4,874305166	4,297925053	4,029452886	3,772941338	PCP013622	XM_009355342
PCP013706	3,736604875	3,584962501	4,115199749	2,22342255	PCP013706	XM_009371150
PCP013937	4,874305166	4,059182199	3,221877081	1,416839742	PCP013937	XM_008360308
PCP013947	5,60496087	4,143230135	3,169925001	3,221877081	PCP013947	XR_665912
PCP014058	4,922673593	4,502712486	4,523561956	3,969933275	PCP014058	XM_009338844
PCP014093	4,890933022	4,321928095	4,029452886	3,27351589	PCP014093	XM_009376374
PCP014525	4,222650022	3,736604875	4,115199749	2,737686761	PCP014525	XM_009342434
PCP014588	5,142821844	4,392317423	5,073391816	0	PCP014588	XM_008357523
PCP014752	4,969472865	3,368768349	3,807354922	3,772941338		
PCP015103	4,938756261	5,115615931	4,459431619	3,663344619	PCP015103	XM_009377286
PCP015139	4,523561956	5,392317423	5,044394119	3,169925001	PCP015139	XM_009379474
PCP015275	5,014801602	4,414812061	3,874796966	1,416839742	PCP015275	XM_009365806
PCP015464	4,059182199	4,564987801	4,392317423	1,416839742	PCP015464	XM_008390080
PCP015465	4,64385619	3,969933275	4,824258697	3,058316496	PCP015465	XM_008371984
PCP015694	5,426264755	4,790250739	3,906890596	2,503348735	PCP015694	XM_008357319
PCP015739	4,392317423	3,969933275	2,6622055	2,414135533	PCP015739	XM_009361782
PCP016435	5	4,143230135	3,736604875	4,34553831		
PCP016452	4,029452886	3,938285792	3,624100895	1,584962501	PCP016452	XM_017330975
PCP016470	4,414812061	3,169925001	2,807354922	2,414135533	PCP016470	XM_009351699
PCP016502	5,02989455	4,682011391	3,502075956	3,663344619	PCP016502	XM_008342332
PCP016512	5,247927513	4,523561956	4,247927513	3,544732656	PCP016512	XM_008361801
PCP016598	4,115199749	3,874796966	3,169925001	1,220329955		
PCP016668	4,321928095	4,718635616	4,029452886	3,415488271	PCP016668	XM_009366576
PCP016741	4,624685811	4,169925001	4,459431619	3,221877081	PCP016741	XM_008351056
PCP016781	3,969933275	3,415488271	2,321928095	1,875780063	PCP016781	XM_009336187
PCP016914	6,807354922	5,513174885	4,059182199	0	PCP016914	XM_009379185
PCP017207	4,584962501	3,459431619	3,584962501	3,544732656	PCP017207	XM_009360786
PCP017546	4,321928095	3,221877081	4,73714592	3,221877081		
PCP017674	4,115199749	3,221877081	3,624100895	2,584962501	PCP017674	XM_009363550
PCP017898	5,285402219	3,221877081	4,392317423	3,584962501	PCP017898	XR_667876
PCP017900	5,309976492	4,392317423	4,169925001	3,459431619	PCP017900	XM_018645011
PCP017944	4,459431619	3,459431619	3,938285792	2,584962501	PCP017944	XM_009340801
PCP018355	3,116031993	4,247927513	4	2,503348735	PCP018355	XM_009338815

PCP018405	4,143230135	3,624100895	4,169925001	1,875780063	PCP018405	XM_009335635
PCP018409	4,874305166	5,087462841	3,27351589	3,368768349	PCP018409	XM_008353535
PCP018447	5	3,663344619	3,27351589	3,116031993	PCP018447	XM_009360334
PCP018452	4,414812061	4,029452886	3,116031993	1	PCP018452	XM_008359854
PCP018497	4,64385619	4,523561956	4,480911346	3,415488271		
PCP018920	4,502712486	3,663344619	3,221877081	1,416839742	PCP018920	XM_009355767
PCP019466	4,544114402	3,807354922	3,321928095	3,169925001	PCP019466	XM_009339055
PCP019493	4,392317423	4,369466484	4,682011391	3,058316496	PCP019493	XM_009377647
PCP019501	3,969933275	3,906890596	4,369466484	2,873813198	PCP019501	XM_018651749
PCP019610	4,73714592	3,874796966	3,874796966	2,584962501	PCP019610	XM_009364299
PCP019655	4,906890596	4,480911346	4,321928095	3,584962501	PCP019655	XM_017331465
PCP019979	4,34553831	2,873813198	2,114367025	1,735522177	PCP019979	XM_009372221
PCP019987	3,700439718	4,143230135	2,807354922	2,6622055	PCP019987	XM_009374622
PCP020002	3,459431619	5,058749412	4,414812061	3,624100895	PCP020002	XM_009355585
PCP020255	4,718635616	4,985044962	4,392317423	4,059182199	PCP020255	XM_018651168
PCP020305	4,196134881	3,807354922	3,459431619	1,220329955	PCP020305	XM_009339025
PCP020343	4,222650022	3,169925001	3,116031993	2,321928095	PCP020343	XM_008390316
PCP020355	5,584962501	4,662775172	3,807354922	3,624100895	PCP020355	XM_009368723
PCP020396	5,115615931	4,502712486	4,369466484	3,415488271	PCP020396	XM_018646256
PCP020409	4,143230135	3,169925001	3,221877081	2,321928095	PCP020409	XM_018647971
PCP020510	4,682011391	4,938756261	3,584962501	2,873813198	PCP020510	XR_666921
PCP020570	4,73714592	4,143230135	3,807354922	3,116031993	PCP020570	XM_018646409
PCP020779	4,222650022	3,624100895	2,737686761	2,873813198	PCP020779	XM_018644417
PCP020783	5,129283017	5,156639311	4,459431619	2,939226578	PCP020783	XM_009378477
PCP020831	2,939226578	6,176522247	2,737686761	3,368768349	PCP020831	XM_018649489
PCP020841	4,414812061	3,938285792	3,459431619	1,735522177	PCP020841	XM_009337139
PCP020895	5,426264755	4,059182199	4,059182199	4,115199749		
PCP020898	4,523561956	3,840966704	3,663344619	2,114367025	PCP020898	XM_009374747
PCP021141	3,807354922	3,736604875	3,459431619	1,416839742		
PCP021161	4,906890596	4,143230135	4,196134881	3,27351589	PCP021161	XM_009337258
PCP021183	4,64385619	4,624685811	4,059182199	3,459431619	PCP021183	XM_009375034
PCP021257	4,890933022	4,369466484	4,922673593	4	PCP021257	XM_008373357
PCP021625	5,273142859	4,222650022	4,34553831	3,058316496	PCP021625	XM_009359463
PCP021658	6,176522247	4,523561956	1,875780063	0	PCP021658	XM_009366643
PCP021667	5	4,459431619	4,321928095	2,807354922		
PCP021693	4,73714592	4,502712486	4,222650022	3,624100895	PCP021693	XM_009356086
PCP021748	4,874305166	4,857980995	4,369466484	2,873813198	PCP021748	XM_009358820
PCP021762	4,985044962	4,169925001	4,115199749	3	PCP021762	XM_009359983
PCP022011	3,544732656	4,73714592	4,321928095	3,459431619		
PCP022105	4,700439718	4,938756261	4,718635616	3,772941338		
PCP022247	4,564987801	4,564987801	4,272769732	2,939226578	PCP022247	XM_018650498
PCP022364	4,700439718	4,029452886	3,969933275	3,116031993		
PCP022551	5,02989455	3,874796966	3,368768349	3,321928095	PCP022551	XM_009355485
PCP022670	4,196134881	3,736604875	3,169925001	2,321928095	PCP022670	XM_009352436
PCP023098	5,763677142	5,403608584	4,196134881	0		
PCP023195	4,115199749	3,700439718	2	1,735522177	PCP023195	XM_009351600

PCP023645	5,624393382	4,115199749	2,939226578	1,735522177	PCP023645	XM_009335635
PCP023882	3,906890596	5,890689878	3,906890596	4,222650022	PCP023882	XM_008390952
PCP023916	4,169925001	3,502075956	1,584962501	2,939226578	PCP023916	XM_009355134
PCP024226	5,681730355	4,938756261	4,169925001	2,737686761	PCP024226	XM_008352718
PCP024317	4,682011391	4,143230135	3,368768349	1,875780063	PCP024317	XM_009374823
PCP024370	4,143230135	4,437627248	3,544732656	2,737686761	PCP024370	XM_009362684
PCP024491	4,985044962	4,73714592	4,544114402	3,169925001	PCP024491	XM_008349175
PCP024628	4,321928095	3,663344619	3,321928095	0,739848103	PCP024628	XM_009341658
PCP024726	4,459431619	5,297558281	4,087462841	4,297925053	PCP024726	XM_008387893
PCP024844	3,736604875	3,116031993	2,939226578	1,735522177	PCP024844	XM_009361164
PCP025044	4,824258697	3,169925001	2,873813198	0,739848103	PCP025044	XM_009347583
PCP025082	4,874305166	4,321928095	3,058316496	3,321928095	PCP025082	XM_018644291
PCP025102	4,985044962	4,718635616	4,459431619	3,874796966	PCP025102	XM_009377218
PCP025127	4,938756261	4,369466484	4,682011391	3,874796966	PCP025127	XM_018649361
PCP025240	4,115199749	4,029452886	3,700439718	2,6622055	PCP025240	XM_017325366
PCP025367	3,584962501	4,34553831	3,368768349	2,503348735	PCP025367	XM_008379041
PCP025395	5,02989455	4,662775172	4,321928095	2,737686761	PCP025395	XR_001953322
PCP025528	4,64385619	4,414812061	3,840966704	3,221877081	PCP025528	XM_009368867
PCP025657	4,196134881	3,27351589	3,169925001	0,739848103	PCP025657	XM_009378477
PCP025793	4,459431619	3,584962501	2,503348735	2,22342255	PCP025793	XM_009336589
PCP025936	4,841469999	5,058749412	4,437627248	3,415488271	PCP025936	XM_008394808
PCP026165	5,101397952	4,369466484	4,414812061	2,6622055		
PCP026215	4,34553831	4,34553831	4,700439718	3,058316496	PCP026215	XM_009347773
PCP026262	4,807354922	4	4,584962501	3,700439718		
PCP026323	4,502712486	3,169925001	4,087462841	1,416839742	PCP026323	XM_009345306
PCP026578	5,044394119	3,969933275	3,544732656	4,414812061	PCP026578	XM_008376954
PCP026590	3,624100895	3,459431619	3,807354922	1,416839742	PCP026590	XM_009356485
PCP027056	3,544732656	3,27351589	3,221877081	1,220329955	PCP027056	XM_018646033
PCP027151	5,345893086	3,874796966	2,737686761	2,414135533		
PCP027182	3,584962501	4,414812061	3,502075956	3,169925001		
PCP027202	5,297558281	4,564987801	3,969933275	3,221877081	PCP027202	XM_018643170
PCP027568	5,513174885	5,247927513	4,272769732	3,221877081	PCP027568	XM_018643789
PCP027587	4,564987801	3,840966704	0	4,604664415	PCP027587	XM_009346477
PCP027600	4,700439718	3,969933275	4,143230135	3,368768349	PCP027600	XM_009346499
PCP027639	4	4,414812061	4,087462841	2		
PCP027641	4,297925053	3,874796966	3,459431619	2,584962501	PCP027641	XM_009340650
PCP027671	3,969933275	3,27351589	5,672425342	3,058316496	PCP027671	XM_009349608
PCP027764	4,247927513	4,682011391	5,087462841	3,502075956	PCP027764	XM_018649288
PCP027797	3,772941338	4,502712486	3,700439718	2,503348735	PCP027797	XM_009374286
PCP027945	5,247927513	3,663344619	2,939226578	3,116031993	PCP027945	XM_009376512
PCP028047	5,681730355	4,369466484	3,772941338	3,624100895	PCP028047	XM_009335398
PCP028196	4,222650022	4,169925001	4,115199749	1,735522177		
PCP028267	4,222650022	5,169925001	4,459431619	3,058316496	PCP028267	XM_008342534
PCP028295	4,700439718	5,273142859	3,584962501	4,247927513		
PCP028332	4,64385619	4,272769732	3,906890596	3,169925001	PCP028332	XM_018652195
PCP028382	4,624685811	3,221877081	3,969933275	2,22342255	PCP028382	XM_009349564

PCP028524	4,824258697	4,196134881	4,922673593	3,772941338	PCP028524	XM_008375595
PCP028526	4,73714592	4,544114402	4,34553831	3,700439718		
PCP028644	4,922673593	4,369466484	4	3,116031993		
PCP028794	5,709566354	5,247927513	4,059182199	3,058316496	PCP028794	XM_009374989
PCP029010	5,101397952	3,938285792	4,564987801	3,415488271	PCP029010	XM_009335769
PCP029022	4,662775172	4,392317423	2,6622055	2,503348735	PCP029022	XR_001953224
PCP029024	5,196528361	4,700439718	4,222650022	3,169925001	PCP029024	XM_009375398
PCP029128	5,584962501	4,95419631	4,369466484	2,873813198	PCP029128	XM_008340156
PCP029846	4,64385619	1,875780063	3	3,116031993	PCP029846	XM_018647637
PCP030112	4,169925001	3,736604875	4,369466484	2,414135533	PCP030112	XM_008366116
PCP030425	4,437627248	4,143230135	3,938285792	2,807354922	PCP030425	XM_009337492
PCP030457	2,503348735	5,369117459	3,906890596	3,368768349	PCP030457	XM_009349672
PCP030491	3,700439718	6,523561956	4	3,969933275		
PCP030697	5,156639311	4,604664415	3,906890596	1,875780063	PCP030697	XM_009341751
PCP030728	4,807354922	4,754887502	3,807354922	2,807354922	PCP030728	XM_009353358
PCP030814	4,247927513	4,604664415	3,459431619	3,058316496	PCP030814	NM_001294072
PCP030840	4,480911346	3,807354922	3,700439718	2,321928095		
PCP030855	5,101397952	4,772413555	3,874796966	3,221877081	PCP030855	XM_009371932
PCP031050	4,369466484	4,718635616	2,6622055	0	PCP031050	XM_009351008
PCP031100	3,27351589	4,143230135	2,873813198	2,321928095	PCP031100	XM_009380882
PCP031114	4,169925001	4,459431619	3,169925001	1,735522177	PCP031114	XM_009360070
PCP031210	4,087462841	4,34553831	5,247927513	3,27351589	PCP031210	XM_009366088
PCP031224	4,662775172	4,906890596	4,34553831	3,415488271	PCP031224	XM_009336626
PCP031273	5,169925001	4,459431619	3,807354922	0,411426246	PCP031273	XM_009344316
PCP031654	4,682011391	3,874796966	3,969933275	2,939226578	PCP031654	XR_001952698
PCP031844	4,272769732	4,143230135	3,969933275	2,873813198	PCP031844	XM_009350909
PCP031896	4,874305166	3,938285792	4,247927513	3,736604875		
PCP032025	4,392317423	3,938285792	1,875780063	2,114367025		
PCP032375	4,437627248	3,969933275	3,772941338	3,116031993	PCP032375	XM_009376170
PCP032622	4,624685811	3,663344619	3,874796966	3,544732656		
PCP032878	5,415150205	4,321928095	3,502075956	0,739848103	PCP032878	XM_009357851
PCP032881	3,321928095	4,143230135	4,906890596	3,321928095		
PCP033046	4,564987801	3,584962501	3,502075956	2,939226578	PCP033046	XM_009340997
PCP033152	4,938756261	4,459431619	4,272769732	4	PCP033152	XM_009359493
PCP033597	4,922673593	3,584962501	1,416839742	2		
PCP033835	4,874305166	4,029452886	4,029452886	3,700439718	PCP033835	XM_009357031
PCP033836	5,115615931	4,523561956	4,169925001	3,663344619	PCP033836	XM_009357037
PCP033886	4,682011391	3,772941338	3,700439718	2,414135533	PCP033886	XM_009363227
PCP034096	4	3,169925001	3,27351589	2,321928095		
PCP034508	2,321928095	3,321928095	4,414812061	2,114367025	PCP034508	XM_009354520
PCP034947	4	4,564987801	3,772941338	2,737686761		
PCP034953	7,14974712	6,189824559	0	0	PCP034953	XM_009364792
PCP035298	5,101397952	4,459431619	4,369466484	3,906890596	PCP035298	XM_009372940
PCP036482	4,414812061	3,906890596	1,875780063	2,584962501	PCP036482	XM_009345013
PCP036522	4,459431619	3,544732656	4,297925053	3	PCP036522	XM_009377060
PCP036761	5,448570626	4,841469999	3,544732656	3,415488271	PCP036761	XM_018646603

PCP036924	5,073391816	3,906890596	3,906890596	3,938285792	PCP036924	XM_009367594
PCP037547	4,922673593	4,523561956	4,459431619	3,874796966	PCP037547	XM_009347390
PCP037576	3,906890596	2,22342255	3,938285792	1,416839742		
PCP038023	4,824258697	4,459431619	4,772413555	3,27351589		
PCP039116	3,772941338	3,502075956	3,502075956	1,416839742	PCP039116	XM_009358338
PCP039311	4,143230135	4,34553831	2,584962501	2,6622055	PCP039311	XM_018646726
PCP039351	5,183089461	4,890933022	1,220329955	0,411426246	PCP039351	XM_009338362
PCP039467	4,682011391	4,196134881	4,143230135	3,502075956	PCP039467	XM_009378762
PCP039702	5,142821844	4,754887502	2,873813198	2,939226578	PCP039702	XM_009359229
PCP039813	4,718635616	4,029452886	3,807354922	3	PCP039813	XM_009372295
PCP039844	4,841469999	0,411426246	3,840966704	2,6622055		
PCP040050	5,196528361	4,890933022	4,059182199	3,736604875		
PCP040107	5	3,772941338	3,624100895	3,772941338		
PCP040118	3,840966704	3,368768349	3	1,584962501	PCP040118	XM_009368202
PCP040509	4,807354922	2,873813198	3,700439718	3,368768349	PCP040509	XM_008360825
PCP040698	4,169925001	3,459431619	3,584962501	2,6622055	PCP040698	XM_009376037
PCP040802	4,272769732	3,502075956	2,737686761	2	PCP040802	XM_008348775
PCP041469	5,357552005	4,523561956	4,502712486	3,27351589		
PCP041761	4,874305166	3,736604875	4,059182199	3,807354922	PCP041761	XM_009355760
PCP041844	4,523561956	3,663344619	0	3,368768349		
PCP042700	4,523561956	3,663344619	3,321928095	3,116031993		
PCP042805	4,718635616	4,772413555	3,368768349	-0,577766999	PCP042805	XM_009354741
PCP043513	3,874796966	4,196134881	4,544114402	2,414135533	PCP043513	XM_008382032
PCP043990	3,321928095	4,523561956	3,502075956	3,116031993	PCP043990	XM_018642604
PCP044000	4	3,502075956	3,27351589	2,503348735		
PCP044006	4,682011391	2,584962501	2,503348735	1,584962501	PCP044006	XM_009358221
PCP044141	5,156639311	4,523561956	3,459431619	0	PCP044141	XM_009353308
PCP044176	4,682011391	3,368768349	4,502712486	3	PCP044176	XM_009359661
PCP044236	4,196134881	4,841469999	4,682011391	3,700439718		
PCP044309	3,840966704	3,459431619	2,807354922	2,114367025	PCP044309	XM_009366063
PCP044441	3,906890596	2,807354922	3,221877081	1,735522177	PCP044441	XM_009345807
PCP044736	4,029452886	4,544114402	3,663344619	2,114367025	PCP044736	XM_008380335
PCP044845	4,414812061	4,222650022	2,873813198	2,807354922		
PCP045179	4,34553831	3,938285792	3,840966704	2,939226578	PCP045179	XM_018647787

Cluster_9

Gene	H1	H2	H3	H4		
PCP000143	3,116031993	3,169925001	4,841469999	3,502075956	PCP000143	XM_009378004
PCP000222	3,321928095	3,969933275	5,044394119	4,906890596	PCP000222	XM_018643281
PCP000340	3,415488271	4,523561956	4,369466484	5,196528361	PCP000340	XM_008349295
PCP000411	3,415488271	0	5,196528361	3,415488271	PCP000411	XM_009369928
PCP000412	3,27351589	0,739848103	5,101397952	3,058316496	PCP000412	XM_009369928
PCP000801	2,414135533	2,807354922	4,544114402	4,297925053	PCP000801	XM_009350099
PCP000862	3,058316496	4,824258697	4,392317423	4,938756261		
PCP001273	2,584962501	2,737686761	4,564987801	7,541716163		
PCP001307	3,116031993	3,807354922	4,459431619	5,321928095	PCP001307	XM_008385457
PCP001647	0	3,840966704	4,807354922	3,663344619		
PCP001690	3,27351589	5,02989455	4,437627248	4,143230135	PCP001690	XM_009350830
PCP002206	1,584962501	3,772941338	4,059182199	4,544114402	PCP002206	XM_009341083
PCP002357	2,22342255	3,116031993	2,503348735	4,34553831	PCP002357	XM_009365929
PCP002369	2,873813198	4,502712486	5,142821844	3,736604875	PCP002369	XM_008343257
PCP004091	3,624100895	3,840966704	5,115615931	3,874796966		
PCP004355	2,737686761	3,169925001	3,807354922	5,129283017	PCP004355	XM_008370671
PCP005830	3,321928095	3,502075956	4,922673593	4,754887502	PCP005830	XM_021977058
PCP006179	3,700439718	2,807354922	2,114367025	5,044394119	PCP006179	XM_009358357
PCP006314	4,392317423	3,415488271	3,116031993	5,369117459	PCP006314	XM_018648753
PCP006737	2,503348735	3,459431619	4,700439718	4,890933022	PCP006737	XM_018650288
PCP006808	0,739848103	3,700439718	3,624100895	5,533874777		
PCP006815	3,415488271	3,321928095	5,437294331	5,044394119	PCP006815	XM_008355845
PCP006837	4,029452886	3,807354922	3,27351589	5,044394119	PCP006837	XM_017328280
PCP006890	3,27351589	3	3,772941338	5,02989455	PCP006890	XM_009355148
PCP007280	2,114367025	4,95419631	3,663344619	3,700439718	PCP007280	XM_009348032
PCP007395	2,414135533	3	3,772941338	4,790250739	PCP007395	XM_009337213
PCP007541	3,502075956	3,736604875	3,502075956	5,763677142		
PCP007558	2,737686761	3,840966704	4,029452886	5,533874777	PCP007558	XM_017329960
PCP007973	0	5,426264755	5,247927513	5,491853096	PCP007973	XM_018652457
PCP008180	2	3,874796966	4,754887502	5,235344128	PCP008180	XM_008356658
PCP008473	0	3,938285792	5,523561956	3,736604875		
PCP008688	2,22342255	3,321928095	3,906890596	4,369466484	PCP008688	XM_008366023
PCP008737	4,604664415	0	4,754887502	5,044394119	PCP008737	XM_009344816
PCP008840	4	3,584962501	3,321928095	4,95419631	PCP008840	XM_009362430
PCP008901	2,873813198	3,700439718	4,824258697	4,95419631		
PCP008930	3,321928095	2,22342255	3,368768349	6,176522247	PCP008930	XM_017323934
PCP009022	2,114367025	3,874796966	4,480911346	4,059182199		
PCP009188	2,22342255	2,414135533	3,874796966	4,584962501	PCP009188	XM_009338280
PCP009685	1,416839742	3,544732656	4,34553831	5,247927513	PCP009685	XM_008343921
PCP009708	0,411426246	3,27351589	3,906890596	3,700439718	PCP009708	XM_008391789
PCP009739	5,309976492	0,411426246	5,491853096	5,624393382	PCP009739	XM_009358833
PCP009775	2	2,807354922	3,368768349	4,272769732	PCP009775	XM_009350417
PCP009802	1,735522177	2,584962501	5,101397952	7,473056289	PCP009802	XM_008354694
PCP009919	3,415488271	4,222650022	4,985044962	5,156639311	PCP009919	XM_018650545

PCP010160	3,700439718	2,584962501	3	5,789990019	PCP010160	XM_009375995
PCP010504	2	3,736604875	3,544732656	5,554588852	PCP010504	XM_009354285
PCP010591	2,114367025	1,220329955	3,624100895	4,369466484	PCP010591	XM_009359972
PCP010717	3,502075956	2,737686761	3,544732656	4,523561956	PCP010717	XM_009344608
PCP010952	3,772941338	3,772941338	4,906890596	5,156639311	PCP010952	XM_008341625
PCP011831	4,143230135	3	4,321928095	5,727920455	PCP011831	XM_008343280
PCP011951	1,220329955	1,875780063	5,380937195	5,426264755	PCP011951	XM_008374667
PCP012070	4,544114402	3,544732656	3,736604875	5,60496087	PCP012070	XM_009352004
PCP012130	2	4,392317423	3,368768349	3	PCP012130	XR_001951418
PCP012138	3	4	5,345893086	5,60496087	PCP012138	XM_008355191
PCP012255	3,584962501	4,169925001	3,169925001	5,491853096	PCP012255	XM_008370743
PCP012399	2,503348735	2,6622055	4,754887502	4,272769732	PCP012399	XM_008358632
PCP012412	3,221877081	3,415488271	3,874796966	7,251056285	PCP012412	XM_008386869
PCP012809	0	2,737686761	3,415488271	5,073391816	PCP012809	XM_009358391
PCP013011	4,624685811	2,321928095	3,840966704	4,087462841	PCP013011	XM_009365208
PCP013217	1,584962501	2,22342255	3,321928095	6,007419784	PCP013217	XM_021950325
PCP013272	3,969933275	2,503348735	5,058749412	3,874796966	PCP013272	XM_009366106
PCP013292	3,772941338	4,437627248	4,087462841	5,380937195	PCP013292	XM_017328562
PCP013454	3,584962501	3,969933275	4,874305166	5,014801602	PCP013454	XM_009354890
PCP013523	3,221877081	4,115199749	4,196134881	6,156437068		
PCP013599	4,890933022	4,222650022	3,116031993	5,014801602		
PCP013608	2,6622055	4,564987801	3,116031993	4,700439718		
PCP013709	3,321928095	3,321928095	5,247927513	3,27351589	PCP013709	XM_009371149
PCP013957	1,416839742	2,584962501	2,737686761	4,938756261	PCP013957	XM_009338476
PCP013988	2,807354922	2	5,156639311	3,624100895	PCP013988	XM_008383931
PCP014592	2,584962501	3,368768349	4,029452886	4,857980995		
PCP014599	3,700439718	2,807354922	5,977279923	4,222650022	PCP014599	XM_009366067
PCP014817	3,969933275	3,663344619	3,368768349	5,196528361		
PCP014930	2,114367025	3,502075956	3,736604875	4,604664415	PCP014930	XM_009380720
PCP015259	2,6622055	1,735522177	3,840966704	5,763677142		
PCP015521	3,116031993	4,321928095	4,890933022	5,297558281	PCP015521	XM_009356385
PCP015887	3,415488271	4,459431619	5,392317423	3,969933275	PCP015887	XM_009352937
PCP015995	1,584962501	0	3,663344619	5,333781501	PCP015995	XM_009347735
PCP016800	2,321928095	3,116031993	4,414812061	2,807354922	PCP016800	XM_008372503
PCP017407	2,414135533	2,22342255	4,34553831	2,6622055	PCP017407	XM_008383931
PCP017678	3,807354922	4,321928095	4,480911346	5,247927513		
PCP017796	2,114367025	4,480911346	5,156639311	5,984817174	PCP017796	XM_009371480
PCP017800	1,875780063	2,807354922	4,222650022	6,044394119	PCP017800	XM_009371483
PCP017982	3,321928095	3,906890596	4,682011391	5,718909554	PCP017982	XM_018651555
PCP018510	3,169925001	2,807354922	5,824513297	4,297925053	PCP018510	XM_009352865
PCP018540	3	3,772941338	4,718635616	5,554588852	PCP018540	XM_009344489
PCP018561	2,321928095	3,321928095	5,101397952	4,115199749		
PCP018744	2,114367025	3,584962501	4,624685811	3,700439718		
PCP018749	2,22342255	2,321928095	3,221877081	4,584962501	PCP018749	XM_009343842
PCP019266	2,321928095	3,116031993	3,27351589	6,409390936	PCP019266	XM_009355069
PCP019275	3	3,058316496	2	5,502394256	PCP019275	XM_009355036

PCP019864	2,321928095	4,369466484	2,114367025	3,415488271	PCP019864	XM_018652357
PCP019999	3,807354922	3,321928095	4,922673593	3,736604875	PCP019999	XM_009363999
PCP020123	0,411426246	3,058316496	5,415150205	5,57500972	PCP020123	XM_009348097
PCP020139	1,875780063	4,087462841	4,772413555	5,718909554		
PCP020142	3,840966704	3,938285792	5,491853096	4,459431619	PCP020142	XM_009343633
PCP020206	1,416839742	2,22342255	4,502712486	3,368768349	PCP020206	XM_009338173
PCP020243	2,807354922	3,584962501	4,584962501	3,874796966	PCP020243	XM_017325241
PCP020366	3,368768349	4,115199749	4,544114402	4,938756261		
PCP020899	4,222650022	4,196134881	3,169925001	4,790250739	PCP020899	XM_009375425
PCP021132	2,503348735	3,700439718	4,64385619	4,34553831	PCP021132	XM_017322719
PCP021133	2,503348735	3,221877081	4,790250739	3,969933275	PCP021133	XM_018649040
PCP021204	3,116031993	4	3,772941338	5,115615931	PCP021204	XM_008389068
PCP021373	3,116031993	2,737686761	4,115199749	5,459431619	PCP021373	XM_009380371
PCP021941	3,938285792	4,059182199	3,663344619	5,437294331		
PCP021980	0	2,114367025	3,874796966	7,318949464	PCP021980	XM_009335711
PCP022338	3,116031993	4,222650022	4,272769732	4,874305166	PCP022338	XM_017330400
PCP022732	1	3,584962501	3,116031993	4,059182199	PCP022732	XM_009380534
PCP022907	2,939226578	3,874796966	4,824258697	4,196134881	PCP022907	XM_008375897
PCP022961	-0,577766999	5,672425342	4,196134881	5,02989455	PCP022961	XM_009367896
PCP023118	3,116031993	2,807354922	5,073391816	4,321928095	PCP023118	XM_008372483
PCP023224	3,584962501	4,624685811	4,321928095	5,502394256	PCP023224	XM_009373380
PCP023407	4,169925001	2,114367025	4,662775172	5,142821844		
PCP023849	3,938285792	3,736604875	4,604664415	5,222263604	PCP023849	XR_531212
PCP024009	3,663344619	4,029452886	5,058749412	5,014801602		
PCP024056	2,503348735	2,873813198	4,272769732	5,273142859	PCP024056	XM_009348478
PCP024321	1,735522177	4,143230135	4,922673593	4,196134881	PCP024321	XM_009374827
PCP024534	2,939226578	2,503348735	2,321928095	5,502394256	PCP024534	XM_009343430
PCP024738	1,416839742	3,169925001	4,115199749	4,564987801		
PCP024752	2,584962501	1,584962501	3,368768349	5,285402219	PCP024752	XM_009375419
PCP024774	2,807354922	3,116031993	4,143230135	4,624685811	PCP024774	XM_009369812
PCP024942	2,584962501	2,939226578	5,02989455	3,624100895	PCP024942	XM_009349097
PCP024943	2,873813198	2,807354922	4,857980995	3,321928095	PCP024943	XM_009349095
PCP024945	2,321928095	2,414135533	5,470211457	3,27351589	PCP024945	XM_008352951
PCP025134	2,737686761	2,503348735	2,939226578	4,523561956	PCP025134	XM_009362332
PCP025158	2	3,368768349	4,169925001	2,6622055	PCP025158	XM_008369512
PCP025264	2,6622055	3,736604875	3,906890596	5,273142859	PCP025264	XM_018644489
PCP026217	1,220329955	3,116031993	3,584962501	4,196134881	PCP026217	XM_009347778
PCP026446	3,058316496	4,059182199	4,890933022	4,437627248		
PCP026854	2,414135533	4,247927513	4,64385619	4,437627248	PCP026854	XM_009374304
PCP026877	2,939226578	2,321928095	3,221877081	4,874305166	PCP026877	XM_009376764
PCP026892	3,459431619	2,503348735	3,321928095	4,459431619	PCP026892	XM_009378588
PCP027343	2,6622055	3,906890596	3,807354922	4,718635616	PCP027343	XM_009373108
PCP027666	2,939226578	4,272769732	5,044394119	4,369466484	PCP027666	XM_009367401
PCP027716	3,221877081	3,663344619	3,874796966	5,101397952	PCP027716	XM_008368760
PCP027939	3,368768349	3,584962501	4,584962501	4,874305166		
PCP028137	2,6622055	3,368768349	4,437627248	4,682011391	PCP028137	XM_009370322

PCP028317	1,584962501	3,906890596	2,873813198	5,321928095	PCP028317	XM_009344423
PCP028728	3,459431619	2,6622055	2,414135533	4,790250739	PCP028728	XM_009358742
PCP028767	2,414135533	3,874796966	4,414812061	3,700439718	PCP028767	XM_009341956
PCP028989	1,735522177	4,143230135	4,115199749	3,663344619	PCP028989	XM_008384160
PCP029287	4	4,087462841	4,564987801	5,273142859	PCP029287	XM_008394855
PCP029298	3,624100895	3,321928095	2,807354922	8,051643995	PCP029298	XM_018650273
PCP029645	4,059182199	3	3,874796966	4,718635616		
PCP029869	5,260402093	2,584962501	3,584962501	5,663059924		
PCP029980	4,272769732	3,459431619	4,369466484	4,985044962	PCP029980	XM_008366172
PCP030701	2,807354922	4,718635616	5,02989455	4,584962501	PCP030701	XM_009351604
PCP030958	1,584962501	3,27351589	4,272769732	5,129283017	PCP030958	XM_009342312
PCP031375	2,939226578	1,416839742	4,222650022	5,073391816	PCP031375	XM_009360326
PCP032234	0	4,604664415	5,169925001	6,845490051		
PCP032270	2,414135533	3,169925001	1,735522177	4,115199749		
PCP032639	4,059182199	3,27351589	4,369466484	6,051589621	PCP032639	XM_009341259
PCP032640	3,321928095	2,22342255	3,938285792	5,544423562	PCP032640	XM_009341259
PCP032814	2,321928095	1	3,459431619	6,454011343	PCP032814	XM_008394273
PCP032838	2,737686761	3,27351589	4,321928095	5,115615931	PCP032838	XM_009380631
PCP033182	4,392317423	2,737686761	4,624685811	3,772941338		
PCP033518	4,143230135	0	4,624685811	4,272769732	PCP033518	XM_016579852
PCP033580	3	2,584962501	2,807354922	4,772413555	PCP033580	XM_009346207
PCP034194	1,584962501	3,116031993	3,544732656	4,502712486	PCP034194	XM_009366772
PCP034195	0	0	5,523561956	6,902917719		
PCP034698	3,221877081	4,169925001	4,321928095	4,807354922		
PCP034913	3,624100895	4,087462841	4,369466484	5,142821844		
PCP035441	3,624100895	3,663344619	1,220329955	4,544114402	PCP035441	XM_008364817
PCP035902	4,544114402	0	6,327866971	6,403778984	PCP035902	XM_009344288
PCP035945	3,938285792	4,969472865	1,416839742	6,06608919	PCP035945	XM_008374830
PCP037904	2,737686761	2,807354922	2,114367025	4,564987801	PCP037904	XM_021954335
PCP038828	3,058316496	4,059182199	1,735522177	5,874551087		
PCP038955	2,503348735	4,480911346	3,544732656	3,459431619	PCP038955	XM_009376559
PCP039193	5,513174885	2,22342255	4,247927513	4,824258697		
PCP039365	2,807354922	2,873813198	3,321928095	4,662775172	PCP039365	XM_008352466
PCP039627	0	0	4,624685811	8,08390476	PCP039627	XM_008364037
PCP040205	3,624100895	4,34553831	3,772941338	5,073391816		
PCP040481	4,321928095	3,415488271	4,523561956	5,142821844	PCP040481	XM_009359279
PCP040756	4,196134881	4,115199749	4,143230135	5,513174885	PCP040756	XM_009343151
PCP041063	5,209453366	1	5,544423562	5,101397952	PCP041063	XM_008246811
PCP041331	3,221877081	1,220329955	2,321928095	4,624685811	PCP041331	XM_009360481
PCP041369	3,624100895	3,221877081	3,969933275	5,087462841		
PCP042455	4,414812061	0	4,662775172	4,624685811		
PCP042653	4,029452886	2,584962501	3,700439718	5,044394119	PCP042653	XM_008358578
PCP042749	2,22342255	3,116031993	4,414812061	5,101397952	PCP042749	XM_008341460
PCP043142	0	5,247927513	0	6,914923239		
PCP043740	4,222650022	0	3,27351589	4,807354922		
PCP043951	2,939226578	3,906890596	3,807354922	4,824258697	PCP043951	XM_009372575

PCP044057	3,840966704	3,321928095	5,014801602	5,59484709	PCP044057	XM_009357750
PCP044299	3,27351589	0,411426246	4,143230135	3,874796966		
PCP044405	1,735522177	3,772941338	3,321928095	4,196134881	PCP044405	XM_008393352
PCP044889	2,737686761	2,737686761	3,624100895	5,832890014	PCP044889	XM_009361567

Cluster_10

**Gene**	**H1**	**H2**	**H3**	**H4**		
PCP001825	2,321928095	2,873813198	0,411426246	0	PCP001825	XM_009338347
PCP005893	0	3,736604875	1	0,411426246	PCP005893	XM_009349811
PCP005924	0	2,807354922	0	0	PCP005924	XM_008347889
PCP006202	0	4,414812061	0	0	PCP006202	XM_009351630
PCP006730	0	5,014801602	1,416839742	-0,577766999	PCP006730	XM_009345156
PCP010806	0	3,906890596	0,411426246	0	PCP010806	XM_009342604
PCP011019	1	2,321928095	1,220329955	0	PCP011019	XM_009352216
PCP011690	0	3,221877081	0	0,411426246	PCP011690	XM_009356910
PCP011882	0	3,874796966	0	0,739848103	PCP011882	XM_009347412
PCP013201	0	3,874796966	0	0,739848103	PCP013201	XM_009370905
PCP015040	-0,577766999	3,459431619	0,411426246	0		
PCP015143	0	3,058316496	1	0	PCP015143	XM_009379477
PCP015306	0	6,658211483	0	2,414135533		
PCP016257	0	3,116031993	0	0	PCP016257	XM_009371251
PCP016788	1,220329955	2,414135533	-0,577766999	0	PCP016788	XM_009366208
PCP017511	1,220329955	4,222650022	1,416839742	0,411426246	PCP017511	XM_009340251
PCP017826	0	2,584962501	0,411426246	0	PCP017826	XM_009354003
PCP018932	0	2,6622055	0	0	PCP018932	XM_008350168
PCP020121	0,411426246	2,873813198	0	0	PCP020121	XM_009375926
PCP020550	0,411426246	4,73714592	0	1	PCP020550	XM_008374582
PCP020997	1,584962501	2,321928095	1,584962501	0	PCP020997	XM_009375941
PCP021243	1	2,807354922	0,411426246	0	PCP021243	XM_009353888
PCP021374	0	5,014801602	0	1,220329955	PCP021374	XM_009380370
PCP021767	0	3,221877081	0,411426246	0	PCP021767	XM_009340631
PCP021974	0	4	0	0	PCP021974	XM_009377686
PCP022084	0	3,116031993	0	0	PCP022084	XM_009361441
PCP023655	3,368768349	3,874796966	0	2,873813198		
PCP024030	0	2,807354922	0	0		
PCP024062	2,414135533	2,873813198	1,416839742	0	PCP024062	XM_009366234
PCP025154	0	3,840966704	0	0	PCP025154	XM_009350115
PCP027297	1,735522177	3,058316496	0	2,22342255	PCP027297	XM_009350750
PCP028399	1,584962501	2,503348735	1	0	PCP028399	XM_018650869
PCP029986	0	3,700439718	1	0	PCP029986	XM_018651526
PCP031851	2,414135533	4	0	0	PCP031851	XM_009378007
PCP032530	0	4,222650022	0	0	PCP032530	XM_009350265
PCP035390	0	3,321928095	1,416839742	0		
PCP036241	2,503348735	3,058316496	1,220329955	0		
PCP036639	2,114367025	2,6622055	0	0	PCP036639	XM_009362572
PCP038280	0	4,196134881	0	-0,577766999	PCP038280	XM_009358284

PCP041536	0	3,368768349	0	0	PCP041536	XM_009351564
PCP042492	0	7,342785837	0	0		
PCP043449	2,737686761	3,415488271	0	2,321928095	PCP043449	XM_008347718
PCP043837	1,875780063	2,503348735	1	0		
PCP043943	1,416839742	2,503348735	0,739848103	0	PCP043943	XM_018648322
PCP044004	0	4,437627248	0,411426246	-0,577766999	PCP044004	XM_009358284

Cluster_11

Gene	H1	H2	H3	H4		
PCP000233	3,058316496	3,058316496	0	-0,577766999	PCP000233	XM_009339674
PCP000802	2,737686761	1,220329955	0	1,584962501	PCP000802	XM_009350098
PCP001828	2,321928095	1	1	0	PCP001828	XM_009357917
PCP001874	2,6622055	0	-0,577766999	0	PCP001874	XM_018648374
PCP002798	2,414135533	1,875780063	1,584962501	0		
PCP003409	3,058316496	1	0	0	PCP003409	XM_008361518
PCP004002	2,321928095	0,411426246	0	0	PCP004002	XM_009371072
PCP004198	3,415488271	2,321928095	0	0	PCP004198	XM_009379603
PCP004389	2,584962501	2,6622055	0	0	PCP004389	XM_009366369
PCP004984	4,115199749	0	0	0	PCP004984	XM_009363422
PCP006961	3,368768349	2,321928095	0	0	PCP006961	XM_009367373
PCP007325	3,221877081	2,873813198	0	0	PCP007325	XM_009374614
PCP007895	2,807354922	0	0	0	PCP007895	XM_017328151
PCP008572	3,221877081	2,737686761	0	0	PCP008572	XM_018648106
PCP008982	2,873813198	2	0	1,875780063		
PCP010099	2,503348735	0	0	0	PCP010099	XM_009342481
PCP011594	2,737686761	0	0,411426246	0	PCP011594	XM_009380053
PCP013620	3,321928095	0	1,416839742	0	PCP013620	XM_018649895
PCP013746	4,059182199	-0,577766999	1,584962501	0,739848103		
PCP013827	3,116031993	-0,577766999	0	0	PCP013827	XM_009340679
PCP014251	2,321928095	1,220329955	0,411426246	0	PCP014251	XM_008384375
PCP014303	2,807354922	1,220329955	1,416839742	0		
PCP014856	2,321928095	2,584962501	0,411426246	0		
PCP015675	2,6622055	0	1	0	PCP015675	XM_009335996
PCP015899	2,873813198	0	0	0	PCP015899	XR_666287
PCP016390	2,939226578	2,414135533	1,220329955	0	PCP016390	XM_009362917
PCP016448	2,6622055	0	0	0		
PCP019349	2,503348735	0	0,411426246	0		
PCP019707	3,221877081	2,22342255	0	0,411426246	PCP019707	XM_008378316
PCP022974	2,503348735	0	0	0	PCP022974	XM_009378694
PCP023084	3,169925001	1,584962501	1,584962501	0	PCP023084	XM_002950787
PCP023233	3,27351589	2,939226578	0	0	PCP023233	XM_009373397
PCP023585	2,737686761	0	1,875780063	0	PCP023585	XM_008394216
PCP023608	2,6622055	1	0	1,416839742	PCP023608	XM_009350982
PCP023830	2,584962501	0	0	0		
PCP024143	2,503348735	0,411426246	0,411426246	0	PCP024143	XM_009368887
PCP024364	2,414135533	2	0	0	PCP024364	XM_008360397

PCP024510	2,414135533	1,220329955	0	0	PCP024510	XM_009367270
PCP024678	3	1,584962501	1	0	PCP024678	XM_008367739
PCP024932	3,058316496	2,321928095	0	0	PCP024932	XM_009371690
PCP025006	3	0	0	0	PCP025006	XM_017330311
PCP025204	2,584962501	1	-0,577766999	0	PCP025204	XM_009367164
PCP025815	3,058316496	0,411426246	1,584962501	0	PCP025815	XM_009336888
PCP026440	2,807354922	2,114367025	0	0	PCP026440	XM_008352001
PCP026694	2,321928095	1,416839742	0,411426246	0		
PCP027388	2,807354922	0	0	0	PCP027388	XM_009339945
PCP028363	2,6622055	0,411426246	0	0		
PCP028698	2,807354922	1,416839742	1	0	PCP028698	XM_009357769
PCP028788	2,503348735	1,220329955	0,739848103	0	PCP028788	XM_009345155
PCP029316	3	0,739848103	1	0	PCP029316	XM_009352704
PCP030505	2,737686761	1,220329955	0,411426246	0	PCP030505	XM_008391395
PCP030616	2,503348735	2,114367025	0	0		
PCP030738	3,938285792	2,873813198	0	0,411426246	PCP030738	XM_009353340
PCP030831	2,939226578	0,739848103	1,875780063	0	PCP030831	XM_008386562
PCP031530	2,6622055	2,22342255	0,739848103	0	PCP031530	XM_009350056
PCP032513	2,584962501	2,22342255	0,411426246	0	PCP032513	XM_009366829
PCP033098	3,321928095	1	0	1,416839742	PCP033098	XM_008368127
PCP033768	2,22342255	2,414135533	0	0	PCP033768	XM_017333249
PCP035279	4,297925053	1,735522177	0	0	PCP035279	XM_009371464
PCP039037	2,414135533	0,411426246	2,414135533	0		
PCP040594	3,624100895	3,058316496	0	0,411426246		
PCP041149	3,058316496	0,739848103	0,739848103	0		
PCP042542	2,873813198	-0,577766999	0	0	PCP042542	XM_009354107
PCP042566	2,503348735	2,503348735	0	0		
PCP042671	2,873813198	2,503348735	0,411426246	0		
PCP043251	3,807354922	1,735522177	0	2	PCP043251	XM_018646971
PCP044447	3	2,22342255	0	0	PCP044447	XM_009348171
PCP045100	3,544732656	-0,577766999	0,739848103	0,411426246	PCP045100	XM_008394039

Average

cluster	H1	H2	H3	H4
cl_1	5,880715017	5,857427279	5,972417687	5,955666718
cl_2	0,13141695	0,19184928	0,842662862	4,233484062
cl_3	0,42965634	2,902193757	1,749434837	3,494579744
cl_4	0,939010736	0,62706897	3,596867671	2,695973288
cl_5	9,97323514	9,969444416	10,07146689	10,26117955
cl_6	7,757726986	7,769941228	7,889533429	8,050892473
cl_7	3,165817735	3,000319343	2,361399789	0,382418301
cl_8	4,540403499	4,168089575	3,734976483	2,788392192
cl_9	2,829940897	3,183906649	4,112230991	4,899243804
cl_10	0,683515165	3,555738072	0,394112138	0,289651184
cl_11	2,897337318	1,211022558	0,398033084	0,148561884

Supplementary Table S3

N.	%	Cellular_component
601	40,69058903	integral component of membrane
220	14,89505755	nucleus
73	4,942450914	intracellular membrane-bounded organelle
69	4,671631686	ribosome
64	4,333107651	mitochondria
57	3,859174001	plasma membrane
43	2,911306703	membrane
41	2,775897089	chloroplast
27	1,82802979	cytoplasm
18	1,218686527	plasmodesma
16	1,083276913	cytosol
15	1,015572106	extracellular
15	1,015572106	photosystem
12	0,812457684	cell
12	0,812457684	microtubule
11	0,744752877	nucleolus
11	0,744752877	trans-Golgi network
11	0,744752877	vacuole
9	0,609343263	protein phosphatase type 2A complex
8	0,541638456	cell wall
8	0,541638456	plastoglobule
7	0,473933649	Golgi
7	0,473933649	viral nucleocapsid
6	0,406228842	large ribosomal subunit
6	0,406228842	SCF ubiquitin ligase complex
5	0,338524035	endoplasmic reticulum
5	0,338524035	proton-transporting
5	0,338524035	transcriptional repressor complex
4	0,270819228	Cul3-RING ubiquitin ligase complex
4	0,270819228	proteasome regulatory particle, lid subcomplex
4	0,270819228	small ribosomal subunit
4	0,270819228	stromule
3	0,203114421	clathrin-coated vesicle
3	0,203114421	cytoskeleton
3	0,203114421	nascent polypeptide-associated complex
3	0,203114421	respiratory chain
3	0,203114421	TORC1 complex
2	0,135409614	90S preribosome
2	0,135409614	anchored component of plasma membrane
2	0,135409614	exocyst
2	0,135409614	glycerol-3-phosphate dehydrogenase complex
2	0,135409614	isoamylase complex
2	0,135409614	nuclear pore
2	0,135409614	P-body

2	0,135409614	plant-type vacuole membrane
2	0,135409614	plastid
2	0,135409614	plastid nucleoid
2	0,135409614	signal peptidase complex
2	0,135409614	signal recognition particle receptor complex
2	0,135409614	small-subunit processome
2	0,135409614	spliceosomal complex
2	0,135409614	TAT protein transport complex
2	0,135409614	TRAPP complex
2	0,135409614	U5 snRNP
2	0,135409614	ubiquitin ligase complex
1	0,067704807	AP-3 adaptor complex
1	0,067704807	apoplast
1	0,067704807	cohesin complex
1	0,067704807	COP9 signalosome
1	0,067704807	COPII vesicle coat
1	0,067704807	dynein complex
1	0,067704807	ESCRT I complex
1	0,067704807	eukaryotic translation initiation factor 3 complex
1	0,067704807	FACT complex
1	0,067704807	histone acetyltransferase complex
1	0,067704807	Ino80 complex
1	0,067704807	mediator complex
1	0,067704807	molybdopterin synthase complex
1	0,067704807	monolayer-surrounded lipid storage body
1	0,067704807	NatA complex
1	0,067704807	Ndc80 complex
1	0,067704807	nuclear chromosome, telomeric region
1	0,067704807	nuclear telomere cap complex
1	0,067704807	nucleosome
1	0,067704807	peroxisomal membrane
1	0,067704807	peroxisome
1	0,067704807	plastid chromosome
1	0,067704807	pollen tube
1	0,067704807	retromer complex
1	0,067704807	RISC complex
1	0,067704807	SNARE complex
1	0,067704807	synaptonemal complex
1	0,067704807	thylakoid lumen

N.	%	Biological_Process
104	8,724832215	transcription, DNA-templated
74	6,208053691	translation
51	4,27852349	metabolic process
46	3,859060403	signal transduction

31	2,600671141	regulation of transcription
28	2,348993289	proteasome-mediated ubiquitin-dependent protein catabolic process
24	2,013422819	defense response
22	1,845637584	RNA modification
22	1,845637584	transmembrane transport
20	1,677852349	response to biotic stimulus
19	1,593959732	carbohydrate metabolic process and transport
19	1,593959732	protein ubiquitination
17	1,426174497	lipid metabolism
17	1,426174497	protein phosphorylation
17	1,426174497	response to auxin
15	1,258389262	photosynthesis
14	1,174496644	response to light
13	1,090604027	microtubule-based process
13	1,090604027	response to oxidative stress
11	0,922818792	glycolytic process
11	0,922818792	protein glycosylation
10	0,838926174	intracellular transport and signalling
10	0,838926174	vesicle-mediated transport
9	0,755033557	cell redox homeostasis
9	0,755033557	fatty acid metabolism
9	0,755033557	glycerol metabolic process
9	0,755033557	metal ion transport
9	0,755033557	plant-type cell wall organization
9	0,755033557	protein transport
8	0,67114094	cell wall modification and organization
8	0,67114094	proteolysis
8	0,67114094	response to water
8	0,67114094	secondary metabolite biosynthetic process
7	0,587248322	negative regulation of translation
7	0,587248322	protein folding
7	0,587248322	proton transport
7	0,587248322	recognition of pollen
7	0,587248322	response to stress
6	0,503355705	biosynthetic process
6	0,503355705	iron-sulfur cluster assembly
6	0,503355705	maturation of LSU-rRNA
6	0,503355705	oxidation-reduction process
6	0,503355705	S-adenosylmethionine biosynthetic process
6	0,503355705	tricarboxylic acid cycle
5	0,419463087	ATP synthesis and hydrolisis
5	0,419463087	cellular amino acid metabolic process
5	0,419463087	cellulose biosynthetic process
5	0,419463087	chitin catabolic process
5	0,419463087	chloroplast RNA processing

5	0,419463087	methylation
5	0,419463087	polysaccharide process
5	0,419463087	protein import into mitochondrial matrix
5	0,419463087	protein metabolic process
5	0,419463087	regulation of proteolysis
5	0,419463087	ubiquitin-dependent protein catabolic process
4	0,33557047	DNA methylation and repair
4	0,33557047	phosphate ion transport
4	0,33557047	pyridoxal phosphate biosynthetic process
4	0,33557047	response to cadmium ion
4	0,33557047	response to cytokinin
4	0,33557047	RNA splicing
4	0,33557047	spermine biosynthetic process
4	0,33557047	stress-activated protein kinase signaling cascade
3	0,251677852	actin cytoskeleton organization
3	0,251677852	base-excision repair
3	0,251677852	chloroplast organization and
3	0,251677852	chorismate biosynthetic process
3	0,251677852	clathrin coat assembly
3	0,251677852	coenzyme A metabolic process
3	0,251677852	exocytosis
3	0,251677852	flower development
3	0,251677852	fucose metabolic process
3	0,251677852	gene silencing by RNA
3	0,251677852	isoprenoid biosynthetic process
3	0,251677852	L-phenylalanine metabolism
3	0,251677852	mitochondrion organization
3	0,251677852	mRNA splicing, via spliceosome
3	0,251677852	nucleosome assembly
3	0,251677852	pentose-phosphate shunt, non-oxidative branch
3	0,251677852	pigment biosynthetic process
3	0,251677852	porphyrin-containing compound biosynthetic process
3	0,251677852	protein-chromophore linkage
3	0,251677852	response to abscisic acid
3	0,251677852	response to heat
3	0,251677852	response to karrikin
3	0,251677852	ribosome biogenesis
3	0,251677852	RNA processing
3	0,251677852	root development
3	0,251677852	rRNA processing
3	0,251677852	SCF-dependent proteasomal ubiquitin-dependent protein catabolic process
3	0,251677852	sterol metabolism
3	0,251677852	trehalose biosynthetic process
3	0,251677852	xyloglucan biosynthetic process
2	0,167785235	amino acid transmembrane transport

2	0,167785235	cellular glucan metabolic process
2	0,167785235	chloroplast ribulose bisphosphate carboxylase complex biogenesis
2	0,167785235	cytidine to uridine editing
2	0,167785235	embryo development ending in seed dormancy
2	0,167785235	G-protein coupled receptor signaling pathway
2	0,167785235	GDP-mannose metabolic process
2	0,167785235	glucose catabolic process
2	0,167785235	Golgi vesicle budding
2	0,167785235	ion transmembrane transport
2	0,167785235	L-serine biosynthetic process
2	0,167785235	methionine biosynthetic process
2	0,167785235	mitochondrial fission
2	0,167785235	mitochondrial mRNA modification
2	0,167785235	multicellular organism development
2	0,167785235	myo-inositol hexakisphosphate biosynthetic process
2	0,167785235	nicotianamine biosynthetic process
2	0,167785235	pectin catabolic process
2	0,167785235	pentose-phosphate shunt
2	0,167785235	positive regulation of organ growth
2	0,167785235	protein autophosphorylation
2	0,167785235	protein catabolic process
2	0,167785235	protein kinase C-activating G-protein coupled receptor signaling pathway
2	0,167785235	protein secretion
2	0,167785235	purine nucleobase biosynthetic process
2	0,167785235	regulation of defense response
2	0,167785235	regulation of flower development
2	0,167785235	regulation of gene expression
2	0,167785235	regulation of root morphogenesis
2	0,167785235	response to chitin
2	0,167785235	response to far red light
2	0,167785235	response to hydrogen peroxide
2	0,167785235	response to salt stress
2	0,167785235	response to temperature stimulus
2	0,167785235	response to zinc ion
2	0,167785235	ribosomal small subunit biogenesis
2	0,167785235	rRNA (guanine-N7)-methylation
2	0,167785235	seed coat development
2	0,167785235	seed germination
2	0,167785235	signal peptide processing
2	0,167785235	starch catabolic process
2	0,167785235	steroid biosynthetic process
2	0,167785235	systemic acquired resistance
2	0,167785235	tetrapyrrole biosynthetic process
2	0,167785235	thiosulfate transport

2	0,167785235	thylakoid membrane organization
2	0,167785235	tissue development
2	0,167785235	TOR signaling
2	0,167785235	toxin catabolic process
2	0,167785235	transcription elongation from RNA polymerase II promoter
2	0,167785235	triglyceride biosynthetic process
2	0,167785235	ubiquinone biosynthetic process
2	0,167785235	UDP-L-arabinose metabolic process
2	0,167785235	unidimensional cell growth
1	0,083892617	'de novo' IMP biosynthetic process
1	0,083892617	(1->3)-beta-D-glucan biosynthetic process
1	0,083892617	actin filament bundle assembly
1	0,083892617	actin filament organization
1	0,083892617	amine metabolic process
1	0,083892617	attachment of mitotic spindle microtubules to kinetochore
1	0,083892617	carbohydrate derivative metabolic process
1	0,083892617	carotenoid biosynthetic process
1	0,083892617	cell cycle
1	0,083892617	cell differentiation
1	0,083892617	cell division
1	0,083892617	cellular respiration
1	0,083892617	cellulose catabolic process
1	0,083892617	cellulose microfibril organization
1	0,083892617	ceramide metabolic process
1	0,083892617	chloride transmembrane transport
1	0,083892617	chlorophyll biosynthetic process
1	0,083892617	chromatin silencing
1	0,083892617	cyanate metabolic process
1	0,083892617	cysteine biosynthetic process from serine
1	0,083892617	cytidine deamination
1	0,083892617	cytokinin metabolic process
1	0,083892617	D-amino acid catabolic process
1	0,083892617	DNA topological change
1	0,083892617	DNA-templated transcription, termination
1	0,083892617	double-strand break repair via homologous recombination
1	0,083892617	endonucleolytic cleavage
1	0,083892617	folic acid-containing compound metabolic process
1	0,083892617	galactose metabolic process
1	0,083892617	galacturonate biosynthetic process
1	0,083892617	gluconeogenesis
1	0,083892617	glucose transmembrane transport
1	0,083892617	glutamate metabolic process
1	0,083892617	GTP biosynthetic process
1	0,083892617	heme a biosynthetic process
1	0,083892617	histidine biosynthetic process
1	0,083892617	histone acetylation

1	0,083892617	hormone transport
1	0,083892617	IMP salvage
1	0,083892617	inositol phosphate biosynthetic process
1	0,083892617	intra-Golgi vesicle-mediated transport
1	0,083892617	leucine biosynthetic process
1	0,083892617	mitochondrial electron transport, NADH to ubiquinone
1	0,083892617	mitochondrial RNA modification
1	0,083892617	mitochondrial translational elongation
1	0,083892617	mitotic sister chromatid cohesion
1	0,083892617	molybdopterin cofactor biosynthetic process
1	0,083892617	mucilage biosynthetic process
1	0,083892617	nitrate assimilation
1	0,083892617	nitrogen compound metabolic process
1	0,083892617	nucleobase-containing compound metabolic process
1	0,083892617	one-carbon metabolic process
1	0,083892617	ornithine metabolic process
1	0,083892617	pantothenate biosynthetic process from valine
1	0,083892617	pathogenesis
1	0,083892617	pectin biosynthetic process
1	0,083892617	phosphorelay signal transduction system
1	0,083892617	phylloquinone biosynthetic process
1	0,083892617	plant-type secondary cell wall biogenesis
1	0,083892617	pollen tube growth
1	0,083892617	pollen tube guidance
1	0,083892617	positive regulation of cyclin-dependent protein kinase activity
1	0,083892617	production of siRNA involved in chromatin silencing by small RNA
1	0,083892617	production of siRNA involved in RNA interference
1	0,083892617	proteasome regulatory particle assembly
1	0,083892617	protection from non-homologous end joining at telomere
1	0,083892617	protein deneddylation
1	0,083892617	protein deubiquitination
1	0,083892617	protein import into chloroplast stroma
1	0,083892617	protein import into mitochondrial outer membrane
1	0,083892617	protein import into nucleus
1	0,083892617	protein lipoylation
1	0,083892617	protein localization to endoplasmic reticulum exit site
1	0,083892617	protein maturation by iron-sulfur cluster transfer
1	0,083892617	protein polyubiquitination
1	0,083892617	protein retention in ER lumen
1	0,083892617	protein stabilization
1	0,083892617	protein targeting
1	0,083892617	protein targeting to chloroplast
1	0,083892617	pseudouridine synthesis
1	0,083892617	PSII associated light-harvesting complex II catabolic process
1	0,083892617	purine nucleotide biosynthetic process

1	0,083892617	reciprocal meiotic recombination
1	0,083892617	regulation of ARF protein signal transduction
1	0,083892617	regulation of membrane potential
1	0,083892617	regulation of monopolar cell growth
1	0,083892617	regulation of peroxisome size
1	0,083892617	regulation of pollen tube growth
1	0,083892617	regulation of RNA metabolic process
1	0,083892617	regulation of stomatal movement
1	0,083892617	regulation of stomatal opening
1	0,083892617	regulation of systemic acquired resistance
1	0,083892617	regulation of vesicle fusion
1	0,083892617	removal of superoxide radicals
1	0,083892617	response to arsenic-containing substance
1	0,083892617	response to cold
1	0,083892617	response to desiccation
1	0,083892617	response to ethylene
1	0,083892617	response to gibberellin
1	0,083892617	response to high light intensity
1	0,083892617	response to hormone
1	0,083892617	response to metal ion
1	0,083892617	response to red light
1	0,083892617	response to wounding
1	0,083892617	retrograde transport, endosome to Golgi
1	0,083892617	riboflavin biosynthetic process
1	0,083892617	ribosomal large subunit export from nucleus
1	0,083892617	RNA secondary structure unwinding
1	0,083892617	RNA surveillance
1	0,083892617	root hair elongation
1	0,083892617	root morphogenesis
1	0,083892617	seed development
1	0,083892617	seed trichome initiation
1	0,083892617	sexual reproduction
1	0,083892617	shoot system development
1	0,083892617	siderophore biosynthetic process
1	0,083892617	simple leaf morphogenesis
1	0,083892617	skotomorphogenesis
1	0,083892617	small GTPase mediated signal transduction
1	0,083892617	snRNA pseudouridine synthesis
1	0,083892617	specification of floral organ number
1	0,083892617	spermidine biosynthetic process
1	0,083892617	spliceosomal snRNP assembly
1	0,083892617	SRP-dependent cotranslational protein targeting to membrane
1	0,083892617	sulfate reduction
1	0,083892617	superoxide metabolic process
1	0,083892617	telomeric loop formation
1	0,083892617	tetrahydrofolate interconversion

1	0,083892617	tetrahydrofolate metabolic process
1	0,083892617	threonine metabolic process
1	0,083892617	transcription from plastid promoter
1	0,083892617	transport
1	0,083892617	tRNA modification
1	0,083892617	tryptophan catabolic process to kynurenine
1	0,083892617	tyrosine biosynthetic process
1	0,083892617	vacuolar transport
1	0,083892617	valine biosynthetic process
1	0,083892617	valyl-tRNA aminoacylation
1	0,083892617	very long-chain fatty acid biosynthetic process
1	0,083892617	xylan biosynthetic process
1	0,083892617	xylem and phloem pattern formation
1	0,083892617	xyloglucan metabolic process

**N.**	**%**	**Molecular_Function**
118	6,25	DNA binding
104	5,508474576	ATP binding
104	5,508474576	transferase activity
92	4,872881356	oxidoreductase activity
69	3,654661017	structural constituent of ribosome
66	3,495762712	RNA binding
63	3,336864407	metal ion binding
62	3,283898305	protein kinase activity
57	3,019067797	zinc ion binding
47	2,48940678	hydrolase activity
43	2,277542373	ADP binding
41	2,171610169	kinase activity
37	1,959745763	nucleic acid binding
36	1,906779661	sequence-specific DNA binding
32	1,694915254	ubiquitin protein ligase activity
27	1,430084746	iron ion binding
27	1,430084746	protein serine/threonine kinase activity
25	1,324152542	methyltransferase activity
24	1,271186441	ligase activity
23	1,218220339	transmembrane transporter activity
22	1,165254237	GTPase activity
20	1,059322034	calcium ion binding
20	1,059322034	protein dimerization activity
20	1,059322034	ubiquitin-protein transferase activity
18	0,953389831	catalytic activity
16	0,847457627	protein serine/threonine phosphatase activity
14	0,741525424	serine-type endopeptidase activity
13	0,688559322	protein heterodimerization activity
12	0,63559322	ATPase activity

12	0,63559322	protein disulfide oxidoreductase activity
11	0,582627119	peptidase activity
11	0,582627119	translation initiation factor activity
10	0,529661017	aspartic-type endopeptidase activity
10	0,529661017	carbohydrate binding
10	0,529661017	structural constituent of cytoskeleton
10	0,529661017	transporter activity
9	0,476694915	GTP binding
9	0,476694915	O-methyltransferase activity
8	0,423728814	calmodulin binding
8	0,423728814	cysteine-type peptidase activity
8	0,423728814	polysaccharide binding
8	0,423728814	protein phosphatase regulator activity
7	0,370762712	heme binding
7	0,370762712	pectinesterase activity
7	0,370762712	peptidyl-prolyl cis-trans isomerase activity
7	0,370762712	translation elongation factor activity
7	0,370762712	unfolded protein binding
6	0,31779661	chlorophyll binding
6	0,31779661	enzyme inhibitor activity
6	0,31779661	isomerase activity
6	0,31779661	quercetin 7-O-glucosyltransferase activity
5	0,264830508	amino acid transmembrane transporter activity
5	0,264830508	channel activity
5	0,264830508	electron transfer activity
5	0,264830508	fructose-bisphosphate aldolase activity
5	0,264830508	helicase activity
5	0,264830508	inorganic diphosphatase activity
5	0,264830508	lyase activity
5	0,264830508	proton-transporting ATP synthase activity, rotational mechanism
5	0,264830508	receptor binding
5	0,264830508	S-adenosylmethionine-dependent methyltransferase activity
5	0,264830508	ubiquitin protein ligase binding
4	0,211864407	adenosylmethionine decarboxylase activity
4	0,211864407	calcium-dependent phospholipid binding
4	0,211864407	cellulose synthase (UDP-forming) activity
4	0,211864407	chitinase activity
4	0,211864407	drug transmembrane transporter activity
4	0,211864407	flavin adenine dinucleotide binding
4	0,211864407	iron-sulfur cluster binding
4	0,211864407	lipid binding
4	0,211864407	metal ion transmembrane transporter activity
4	0,211864407	pigment binding
4	0,211864407	protein homodimerization activity
4	0,211864407	serine-type carboxypeptidase activity

4	0,211864407	structural molecule activity
4	0,211864407	transition metal ion binding
4	0,211864407	transmembrane receptor protein serine/threonine kinase activity
4	0,211864407	ubiquinol-cytochrome-c reductase activity
3	0,158898305	2-alkenal reductase [NAD(P)] activity
3	0,158898305	3-deoxy-7-phosphoheptulonate synthase activity
3	0,158898305	antiporter activity
3	0,158898305	chaperone binding
3	0,158898305	DNA-3-methyladenine glycosylase activity
3	0,158898305	glycopeptide alpha-N-acetylgalactosaminidase activity
3	0,158898305	hydroxymethylglutaryl-CoA reductase (NADPH) activity
3	0,158898305	lipoate synthase activity
3	0,158898305	metalloendopeptidase activity
3	0,158898305	methionine adenosyltransferase activity
3	0,158898305	NAD(P)H dehydrogenase (quinone) activity
3	0,158898305	NADH dehydrogenase (ubiquinone) activity
3	0,158898305	peroxidase activity
3	0,158898305	phosphatase activity
3	0,158898305	phosphatidylinositol binding
3	0,158898305	phosphoenolpyruvate carboxylase activity
3	0,158898305	phosphoglycerate kinase activity
3	0,158898305	protein binding involved in protein folding
3	0,158898305	protein domain specific binding
3	0,158898305	pyruvate kinase activity
3	0,158898305	ribose-5-phosphate isomerase activity
3	0,158898305	thiosulfate transmembrane transporter activity
3	0,158898305	transcription factor activity, transcription factor binding
3	0,158898305	transcription regulatory region DNA binding
2	0,105932203	3-oxo-pimeloyl-[acp] methyl ester reductase activity
2	0,105932203	actin binding
2	0,105932203	acyl-[acyl-carrier-protein] desaturase activity
2	0,105932203	beta-amylase activity
2	0,105932203	carboxyl- or carbamoyltransferase activity
2	0,105932203	diacylglycerol O-acyltransferase activity
2	0,105932203	double-stranded DNA binding
2	0,105932203	FAD binding
2	0,105932203	galactosyltransferase activity
2	0,105932203	GDP-mannose 4,6-dehydratase activity
2	0,105932203	glutathione transferase activity
2	0,105932203	hydro-lyase activity
2	0,105932203	hydrogen ion transmembrane transporter activity
2	0,105932203	inorganic phosphate transmembrane transporter activity
2	0,105932203	isoamylase activity
2	0,105932203	L-malate dehydrogenase activity
2	0,105932203	long-chain-alcohol oxidase activity

2	0,105932203	magnesium ion binding
2	0,105932203	manganese ion binding
2	0,105932203	NAD+ kinase activity
2	0,105932203	NADP binding
2	0,105932203	nicotianamine synthase activity
2	0,105932203	nucleotide binding
2	0,105932203	O-phospho-L-serine:2-oxoglutarate aminotransferase activity
2	0,105932203	oxalate-CoA ligase activity
2	0,105932203	pectinesterase inhibitor activity
2	0,105932203	peptide-methionine (S)-S-oxide reductase activity
2	0,105932203	peptide-N4-(N-acetyl-beta-glucosaminyl)asparagine amidase activity
2	0,105932203	phosphogluconate dehydrogenase (decarboxylating) activity
2	0,105932203	phosphoglycerate mutase activity
2	0,105932203	phospholipid-translocating ATPase activity
2	0,105932203	phosphoprotein phosphatase activity
2	0,105932203	phosphoric diester hydrolase activity
2	0,105932203	polygalacturonase activity
2	0,105932203	polygalacturonate 4-alpha-galacturonosyltransferase activity
2	0,105932203	protein channel activity
2	0,105932203	protein transporter activity
2	0,105932203	reticuline oxidase activity
2	0,105932203	ribulose-bisphosphate carboxylase activity
2	0,105932203	RNA polymerase II regulatory region sequence-specific DNA binding
2	0,105932203	RNA-directed 5'-3' RNA polymerase activity
2	0,105932203	RNA-directed DNA polymerase activity
2	0,105932203	rRNA (guanine) methyltransferase activity
2	0,105932203	rRNA binding
2	0,105932203	scopolin beta-glucosidase activity
2	0,105932203	secondary active sulfate transmembrane transporter activity
2	0,105932203	signal transducer activity
2	0,105932203	sn-glycerol-3-phosphate:ubiquinone-8 oxidoreductase activity
2	0,105932203	strictosidine synthase activity
2	0,105932203	thioredoxin-disulfide reductase activity
2	0,105932203	threonine synthase activity
2	0,105932203	transaminase activity
2	0,105932203	transcription cofactor activity
2	0,105932203	translation release factor activity
2	0,105932203	transmembrane receptor protein tyrosine kinase activity
2	0,105932203	UDP-arabinopyranose mutase activity
2	0,105932203	uroporphyrinogen decarboxylase activity
2	0,105932203	voltage-gated anion channel activity
2	0,105932203	water channel activity
1	0,052966102	1-phosphatidylinositol binding
1	0,052966102	1,3-beta-D-glucan synthase activity

1	0,052966102	2,3-dihydro-2,3-dihydroxybenzoate dehydrogenase activity
1	0,052966102	3-isopropylmalate dehydrogenase activity
1	0,052966102	6-phosphofructokinase activity
1	0,052966102	6-phosphogluconolactonase activity
1	0,052966102	acetylglucosaminyltransferase activity
1	0,052966102	acid phosphatase activity
1	0,052966102	actin filament binding
1	0,052966102	acyl carrier activity
1	0,052966102	amidophosphoribosyltransferase activity
1	0,052966102	AMP deaminase activity
1	0,052966102	ARF guanyl-nucleotide exchange factor activity
1	0,052966102	arginine decarboxylase activity
1	0,052966102	arogenate dehydratase activity
1	0,052966102	aspartate kinase activity
1	0,052966102	beta-glucosidase activity
1	0,052966102	caffeoyl-CoA: alcohol caffeoyl transferase activity
1	0,052966102	capsorubin synthase activity
1	0,052966102	carboxylic ester hydrolase activity
1	0,052966102	cation transmembrane transporter activity
1	0,052966102	cellulase activity
1	0,052966102	chlorophyllide a oxygenase [overall] activity
1	0,052966102	clathrin binding
1	0,052966102	cobalt ion binding
1	0,052966102	coenzyme binding
1	0,052966102	copper ion binding
1	0,052966102	copper ion transmembrane transporter activity
1	0,052966102	coproporphyrinogen oxidase activity
1	0,052966102	cyclic-nucleotide phosphodiesterase activity
1	0,052966102	cysteine-type endopeptidase inhibitor activity
1	0,052966102	cytidine deaminase activity
1	0,052966102	cytidylate kinase activity
1	0,052966102	cytochrome-c oxidase activity
1	0,052966102	D-aminoacyl-tRNA deacylase activity
1	0,052966102	dioxygenase activity
1	0,052966102	DNA (cytosine-5-)-methyltransferase activity
1	0,052966102	DNA topoisomerase type II (ATP-hydrolyzing) activity
1	0,052966102	DNA-directed 5'-3' RNA polymerase activity
1	0,052966102	double-stranded telomeric DNA binding
1	0,052966102	endopeptidase activity
1	0,052966102	epoxide hydrolase activity
1	0,052966102	ethylene binding
1	0,052966102	fatty acid elongase activity
1	0,052966102	formate-tetrahydrofolate ligase activity
1	0,052966102	G-protein coupled receptor activity
1	0,052966102	galactinol-raffinose galactosyltransferase activity
1	0,052966102	glucan endo-1,3-beta-glucanase activity, C-3 substituted

		reducing group
1	0,052966102	glycine hydroxymethyltransferase activity
1	0,052966102	glycogen phosphorylase activity
1	0,052966102	GTPase activator activity
1	0,052966102	guanyl-nucleotide exchange factor activity
1	0,052966102	homoserine kinase activity
1	0,052966102	imidazoleglycerol-phosphate dehydratase activity
1	0,052966102	inositol 3-alpha-galactosyltransferase activity
1	0,052966102	intramolecular transferase activity
1	0,052966102	iron-sulfur transferase activity
1	0,052966102	magnesium chelatase activity
1	0,052966102	magnesium protoporphyrin IX methyltransferase activity
1	0,052966102	malate dehydrogenase (decarboxylating) (NAD+) activity
1	0,052966102	MAP kinase kinase kinase activity
1	0,052966102	mediator complex binding
1	0,052966102	methylenetetrahydrofolate dehydrogenase (NADP+) activity
1	0,052966102	microtubule motor activity
1	0,052966102	microtubule-severing ATPase activity
1	0,052966102	molybdopterin cofactor binding
1	0,052966102	molybdopterin synthase activity
1	0,052966102	mRNA binding
1	0,052966102	NAD+ ADP-ribosyltransferase activity
1	0,052966102	nitrate reductase (NADH) activity
1	0,052966102	nuclease activity
1	0,052966102	nucleobase-containing compound kinase activity
1	0,052966102	nucleosome binding
1	0,052966102	nucleotidyltransferase activity
1	0,052966102	nutrient reservoir activity
1	0,052966102	omega peptidase activity
1	0,052966102	P-P-bond-hydrolysis-driven protein transmembrane transporter activity
1	0,052966102	pantoate-beta-alanine ligase activity
1	0,052966102	peptide alpha-N-acetyltransferase activity
1	0,052966102	peptide-methionine (R)-S-oxide reductase activity
1	0,052966102	phenylalanine ammonia-lyase activity
1	0,052966102	phosphatase activator activity
1	0,052966102	phosphatidylethanolamine binding
1	0,052966102	phosphatidylinositol phosphate kinase activity
1	0,052966102	phosphoadenylyl-sulfate reductase (thioredoxin) activity
1	0,052966102	phosphodiesterase I activity
1	0,052966102	phosphoenolpyruvate carboxykinase (ATP) activity
1	0,052966102	phosphorelay sensor kinase activity
1	0,052966102	phosphoribosylamine-glycine ligase activity
1	0,052966102	phosphoribosylformylglycinamidine synthase activity
1	0,052966102	phosphotransferase activity, alcohol group as acceptor
1	0,052966102	Photinus-luciferin 4-monooxygenase (ATP-hydrolyzing) activity

1	0,052966102	poly(A)-specific ribonuclease activity
1	0,052966102	potassium channel activity
1	0,052966102	potassium ion binding
1	0,052966102	potassium ion transmembrane transporter activity
1	0,052966102	prephenate dehydratase activity
1	0,052966102	prephenate dehydrogenase (NADP+) activity
1	0,052966102	primary amine oxidase activity
1	0,052966102	protease binding
1	0,052966102	protein serine/threonine phosphatase inhibitor activity
1	0,052966102	protochlorophyllide reductase activity
1	0,052966102	proton-transporting ATPase activity, rotational mechanism
1	0,052966102	pseudouridine synthase activity
1	0,052966102	pyridoxal phosphate binding
1	0,052966102	quercetin 3-O-glucosyltransferase activity
1	0,052966102	quinone binding
1	0,052966102	Rab GTPase binding
1	0,052966102	racemase activity, acting on amino acids and derivatives
1	0,052966102	racemase and epimerase activity, acting on carbohydrates and derivatives
1	0,052966102	Ran GTPase binding
1	0,052966102	rhodopsin kinase activity
1	0,052966102	riboflavin synthase activity
1	0,052966102	ribosomal large subunit binding
1	0,052966102	RNA polymerase II carboxy-terminal domain kinase activity
1	0,052966102	RNA polymerase II transcription cofactor activity
1	0,052966102	RNA polymerase II transcription factor activity, sequence-specific DNA binding
1	0,052966102	rRNA (adenine-N6,N6-)-dimethyltransferase activity
1	0,052966102	rRNA (guanosine-2'-O-)-methyltransferase activity
1	0,052966102	sarcosine oxidase activity
1	0,052966102	selenocysteine lyase activity
1	0,052966102	serine O-acetyltransferase activity
1	0,052966102	SNARE binding
1	0,052966102	solute:proton antiporter activity
1	0,052966102	superoxide dismutase activity
1	0,052966102	TBP-class protein binding
1	0,052966102	thiamine pyrophosphate binding
1	0,052966102	thiol-dependent ubiquitin-specific protease activity
1	0,052966102	threonine-type endopeptidase activity
1	0,052966102	toxin activity
1	0,052966102	transcription coactivator activity
1	0,052966102	transcription factor activity, RNA polymerase II transcription factor binding
1	0,052966102	translation initiation factor binding
1	0,052966102	triose-phosphate isomerase activity
1	0,052966102	tRNA methyltransferase activity

1	0,052966102	tryptophan synthase activity
1	0,052966102	tyramine N-feruloyltransferase activity
1	0,052966102	UDP-glucose 6-dehydrogenase activity
1	0,052966102	UDP-glucuronate 4-epimerase activity
1	0,052966102	valine-tRNA ligase activity
1	0,052966102	voltage-gated chloride channel activity
1	0,052966102	voltage-gated potassium channel activity
1	0,052966102	xyloglucan 6-xylosyltransferase activity
1	0,052966102	xyloglucan:xyloglucosyl transferase activity

Supplementary Table S4

interaction (F_MAX)	name	Strength
neg_interaction	F_max_interaction_PCP001326	-0.9504270450831735
neg_interaction	F_max_interaction_PCP029800	-0.9507025280679555
neg_interaction	F_max_interaction_PCP044863	-0.9507065386258193
neg_interaction	F_max_interaction_PCP009753	-0.9516368617388606
neg_interaction	F_max_interaction_PCP036481	-0.9517109370756323
neg_interaction	F_max_interaction_PCP017080	-0.9520571097151868
neg_interaction	F_max_interaction_PCP011779	-0.952112859464925
neg_interaction	F_max_interaction_PCP014031	-0.9521887362403411
neg_interaction	F_max_interaction_PCP008428	-0.9523250562051825
neg_interaction	F_max_interaction_PCP039793	-0.9524226699849822
neg_interaction	F_max_interaction_PCP008901	-0.9524797215452292
neg_interaction	F_max_interaction_PCP004420	-0.9525139779238556
neg_interaction	F_max_interaction_PCP025832	-0.9527465942748633
neg_interaction	F_max_interaction_PCP030800	-0.9529228255735326
neg_interaction	F_max_interaction_PCP010960	-0.9534828263198536
neg_interaction	F_max_interaction_PCP002971	-0.9538111912090615
neg_interaction	F_max_interaction_PCP015613	-0.9540314982871785
neg_interaction	F_max_interaction_PCP016921	-0.9542534988330947
neg_interaction	F_max_interaction_PCP028054	-0.9543565645222846
neg_interaction	F_max_interaction_PCP039914	-0.9544675362312522
neg_interaction	F_max_interaction_PCP013597	-0.9548108530517054
neg_interaction	F_max_interaction_PCP042045	-0.9548754383219409
neg_interaction	F_max_interaction_PCP000204	-0.9553301763989338
neg_interaction	F_max_interaction_PCP022181	-0.9553380810704621
neg_interaction	F_max_interaction_PCP000401	-0.9554938037090299
neg_interaction	F_max_interaction_PCP013496	-0.9557026108141012
neg_interaction	F_max_interaction_PCP024009	-0.9564126313032355
neg_interaction	F_max_interaction_PCP009919	-0.9566956946269072
neg_interaction	F_max_interaction_PCP006852	-0.9570719940052288
neg_interaction	F_max_interaction_PCP010065	-0.957202199307227
neg_interaction	F_max_interaction_PCP000857	-0.9586208039456402
neg_interaction	F_max_interaction_PCP026854	-0.9590805069270322
neg_interaction	F_max_interaction_PCP029251	-0.9591419304112063
neg_interaction	F_max_interaction_PCP011023	-0.9593497027280472
neg_interaction	F_max_interaction_PCP043214	-0.9602368778901574
neg_interaction	F_max_interaction_PCP030436	-0.9608952071154415
neg_interaction	F_max_interaction_PCP013157	-0.9610470049917227
neg_interaction	F_max_interaction_PCP015341	-0.9612187594316431
neg_interaction	F_max_interaction_PCP000700	-0.9615890468809171
neg_interaction	F_max_interaction_PCP006190	-0.9617119643650598
neg_interaction	F_max_interaction_PCP009022	-0.9617507880965114
neg_interaction	F_max_interaction_PCP003944	-0.9617802397995477
neg_interaction	F_max_interaction_PCP010888	-0.9622530303760785
neg_interaction	F_max_interaction_PCP000218	-0.962657094020461

neg_interaction	F_max_interaction_PCP030615	-0.9632179444066407
neg_interaction	F_max_interaction_PCP010826	-0.9636576858068524
neg_interaction	F_max_interaction_PCP002652	-0.9644369990372422
neg_interaction	F_max_interaction_PCP003339	-0.9654823856405986
neg_interaction	F_max_interaction_PCP027077	-0.9661526985536869
neg_interaction	F_max_interaction_PCP018576	-0.9664363435763177
neg_interaction	F_max_interaction_PCP042536	-0.9671772528502744
neg_interaction	F_max_interaction_PCP026446	-0.9680972384225289
neg_interaction	F_max_interaction_PCP017722	-0.9682554783509676
neg_interaction	F_max_interaction_PCP031453	-0.9684822858847598
neg_interaction	F_max_interaction_PCP013976	-0.9685261500810636
neg_interaction	F_max_interaction_PCP006327	-0.9689456608631547
neg_interaction	F_max_interaction_PCP030440	-0.9696235373979192
neg_interaction	F_max_interaction_PCP024562	-0.9696381998767968
neg_interaction	F_max_interaction_PCP008236	-0.970214007642211
neg_interaction	F_max_interaction_PCP001822	-0.9707356205346344
neg_interaction	F_max_interaction_PCP000901	-0.9709596015639821
neg_interaction	F_max_interaction_PCP007726	-0.971328515859397
neg_interaction	F_max_interaction_PCP000675	-0.9714540365329046
neg_interaction	F_max_interaction_PCP044601	-0.971544093364962
neg_interaction	F_max_interaction_PCP041866	-0.9715890262386848
neg_interaction	F_max_interaction_PCP017338	-0.972501412476144
neg_interaction	F_max_interaction_PCP019657	-0.9726607654490025
neg_interaction	F_max_interaction_PCP007419	-0.9726782562053043
neg_interaction	F_max_interaction_PCP026432	-0.973220463032113
neg_interaction	F_max_interaction_PCP008634	-0.9733440970409607
neg_interaction	F_max_interaction_PCP009825	-0.973404559434755
neg_interaction	F_max_interaction_PCP044637	-0.9735149929616961
neg_interaction	F_max_interaction_PCP011606	-0.973654862046739
neg_interaction	F_max_interaction_PCP000262	-0.973847775764776
neg_interaction	F_max_interaction_PCP044174	-0.9749011837160232
neg_interaction	F_max_interaction_PCP002434	-0.9751066383056091
neg_interaction	F_max_interaction_PCP025186	-0.9751545990284086
neg_interaction	F_max_interaction_PCP002911	-0.9758660478291683
neg_interaction	F_max_interaction_PCP018281	-0.9760432237997047
neg_interaction	F_max_interaction_PCP017818	-0.9763110668038161
neg_interaction	F_max_interaction_PCP000528	-0.9769666413059191
neg_interaction	F_max_interaction_PCP000222	-0.9773683670191241
neg_interaction	F_max_interaction_PCP019597	-0.9779644750218404
neg_interaction	F_max_interaction_PCP021142	-0.9782102567663082
neg_interaction	F_max_interaction_PCP000158	-0.9786934488128981
neg_interaction	F_max_interaction_PCP028203	-0.9789991549818572
neg_interaction	F_max_interaction_PCP021679	-0.9795037158323459
neg_interaction	F_max_interaction_PCP023644	-0.9797789508100941
neg_interaction	F_max_interaction_PCP005638	-0.9800160459617584
neg_interaction	F_max_interaction_PCP040830	-0.980158189226693

neg_interaction	F_max_interaction_PCP007235	-0.9802489908588101
neg_interaction	F_max_interaction_PCP010607	-0.9802545503544877
neg_interaction	F_max_interaction_PCP019422	-0.9802644125045968
neg_interaction	F_max_interaction_PCP020027	-0.9804919624118216
neg_interaction	F_max_interaction_PCP014018	-0.9805816312896984
neg_interaction	F_max_interaction_PCP020093	-0.9817042192150481
neg_interaction	F_max_interaction_PCP011848	-0.9817154259256354
neg_interaction	F_max_interaction_PCP017275	-0.9818210321143342
neg_interaction	F_max_interaction_PCP025476	-0.9819567471042175
neg_interaction	F_max_interaction_PCP038637	-0.9822196140142638
neg_interaction	F_max_interaction_PCP004153	-0.9824557009359807
neg_interaction	F_max_interaction_PCP027159	-0.9828813408955925
neg_interaction	F_max_interaction_PCP023958	-0.9834760289906533
neg_interaction	F_max_interaction_PCP038106	-0.9836606651978987
neg_interaction	F_max_interaction_PCP045114	-0.9837111053759215
neg_interaction	F_max_interaction_PCP004361	-0.9837260184763398
neg_interaction	F_max_interaction_PCP009708	-0.9838838140591399
neg_interaction	F_max_interaction_PCP018152	-0.9841548903077931
neg_interaction	F_max_interaction_PCP021209	-0.9841859963289933
neg_interaction	F_max_interaction_PCP030518	-0.9843183642011645
neg_interaction	F_max_interaction_PCP013445	-0.986676404480544
neg_interaction	F_max_interaction_PCP032244	-0.9868304711676139
neg_interaction	F_max_interaction_PCP038321	-0.9872070232737163
neg_interaction	F_max_interaction_PCP020957	-0.9887358873639066
neg_interaction	F_max_interaction_PCP022491	-0.9887418241137887
neg_interaction	F_max_interaction_PCP028795	-0.9889215262256281
neg_interaction	F_max_interaction_PCP020036	-0.9891777953605071
neg_interaction	F_max_interaction_PCP008327	-0.9898805642094749
neg_interaction	F_max_interaction_PCP011965	-0.9899037962042468
neg_interaction	F_max_interaction_PCP029049	-0.9901036219430391
neg_interaction	F_max_interaction_PCP005384	-0.990616986451057
neg_interaction	F_max_interaction_PCP026279	-0.9919025928024505
neg_interaction	F_max_interaction_PCP001799	-0.992141507722775
neg_interaction	F_max_interaction_PCP008290	-0.992145115870776
neg_interaction	F_max_interaction_PCP026713	-0.9922929629235022
neg_interaction	F_max_interaction_PCP021730	-0.9925978166631747
neg_interaction	F_max_interaction_PCP034645	-0.9926468750775052
neg_interaction	F_max_interaction_PCP021132	-0.9928619217633923
neg_interaction	F_max_interaction_PCP000614	-0.9928986080128371
neg_interaction	F_max_interaction_PCP039581	-0.9930342796700358
neg_interaction	F_max_interaction_PCP042928	-0.9949750646755191
neg_interaction	F_max_interaction_PCP022495	-0.9953687796590108
neg_interaction	F_max_interaction_PCP001840	-0.9959733548605151
neg_interaction	F_max_interaction_PCP024930	-0.9960252388798205
neg_interaction	F_max_interaction_PCP027703	-0.9961040789873425
neg_interaction	F_max_interaction_PCP016842	-0.9971687397358004

neg_interaction	F_max_interaction_PCP031664	-0.9979895941389629
neg_interaction	F_max_interaction_PCP021692	-0.9980814797724393
neg_interaction	F_max_interaction_PCP013929	-0.9988717861612466
neg_interaction	F_max_interaction_PCP025978	-0.9991629073351048
neg_interaction	F_max_interaction_PCP037522	-0.9991843360874256
pos_interaction	F_max_interaction_PCP020054	0.9500137951766581
pos_interaction	F_max_interaction_PCP005268	0.9500393647638944
pos_interaction	F_max_interaction_PCP011275	0.9500580733748496
pos_interaction	F_max_interaction_PCP044472	0.950088748095583
pos_interaction	F_max_interaction_PCP018315	0.9501503641023241
pos_interaction	F_max_interaction_PCP027151	0.9502223851946696
pos_interaction	F_max_interaction_PCP020254	0.9502303411557265
pos_interaction	F_max_interaction_PCP028794	0.9502665565508177
pos_interaction	F_max_interaction_PCP023455	0.9504728702844173
pos_interaction	F_max_interaction_PCP020402	0.9505967795069429
pos_interaction	F_max_interaction_PCP044442	0.9509141415096586
pos_interaction	F_max_interaction_PCP034153	0.9516249568364235
pos_interaction	F_max_interaction_PCP044845	0.9516636608118691
pos_interaction	F_max_interaction_PCP022658	0.9517711190232274
pos_interaction	F_max_interaction_PCP003409	0.9518481253940869
pos_interaction	F_max_interaction_PCP021687	0.9518923405787078
pos_interaction	F_max_interaction_PCP026875	0.9519646045958449
pos_interaction	F_max_interaction_PCP002370	0.9520186894137164
pos_interaction	F_max_interaction_PCP017504	0.952175188869346
pos_interaction	F_max_interaction_PCP022849	0.95223778296796
pos_interaction	F_max_interaction_PCP039351	0.9522557629775082
pos_interaction	F_max_interaction_PCP002867	0.952419811059413
pos_interaction	F_max_interaction_PCP007096	0.9524226699849888
pos_interaction	F_max_interaction_PCP020651	0.9527114167266674
pos_interaction	F_max_interaction_PCP009961	0.9527343156187716
pos_interaction	F_max_interaction_PCP022982	0.9527680906689568
pos_interaction	F_max_interaction_PCP020578	0.9530772228021576
pos_interaction	F_max_interaction_PCP030413	0.9535758577819349
pos_interaction	F_max_interaction_PCP002552	0.9538863769806412
pos_interaction	F_max_interaction_PCP016914	0.9539944336830074
pos_interaction	F_max_interaction_PCP001279	0.9545232719944791
pos_interaction	F_max_interaction_PCP010943	0.9546446880243487
pos_interaction	F_max_interaction_PCP043122	0.954707788828419
pos_interaction	F_max_interaction_PCP012204	0.9552319422867226
pos_interaction	F_max_interaction_PCP006414	0.9553088863757837
pos_interaction	F_max_interaction_PCP021997	0.955402394909617
pos_interaction	F_max_interaction_PCP009922	0.9555540992748401
pos_interaction	F_max_interaction_PCP040802	0.956252536384012
pos_interaction	F_max_interaction_PCP000385	0.9563633136802899
pos_interaction	F_max_interaction_PCP044044	0.9564167376094868

pos_interaction	F_max_interaction_PCP008851	0.9566980914071654
pos_interaction	F_max_interaction_PCP024971	0.9567574939571047
pos_interaction	F_max_interaction_PCP001807	0.9569632398009208
pos_interaction	F_max_interaction_PCP000531	0.9569988539366784
pos_interaction	F_max_interaction_PCP040050	0.9575228978630133
pos_interaction	F_max_interaction_PCP007917	0.9578013985145868
pos_interaction	F_max_interaction_PCP008956	0.9582930640859897
pos_interaction	F_max_interaction_PCP029022	0.958480577284205
pos_interaction	F_max_interaction_PCP013255	0.958609866517489
pos_interaction	F_max_interaction_PCP029046	0.9587869610171609
pos_interaction	F_max_interaction_PCP033179	0.9588454328499647
pos_interaction	F_max_interaction_PCP004002	0.9591868453831539
pos_interaction	F_max_interaction_PCP017752	0.9593888797475633
pos_interaction	F_max_interaction_PCP025052	0.9593904831565103
pos_interaction	F_max_interaction_PCP016236	0.9593938701871095
pos_interaction	F_max_interaction_PCP043663	0.959917069184069
pos_interaction	F_max_interaction_PCP038764	0.9599621353226463
pos_interaction	F_max_interaction_PCP004900	0.9602418103949663
pos_interaction	F_max_interaction_PCP022315	0.9604953708048615
pos_interaction	F_max_interaction_PCP007969	0.9605244950764615
pos_interaction	F_max_interaction_PCP009402	0.9608745497790605
pos_interaction	F_max_interaction_PCP025125	0.9613493415170111
pos_interaction	F_max_interaction_PCP019046	0.9613503715582211
pos_interaction	F_max_interaction_PCP021658	0.9614383894407754
pos_interaction	F_max_interaction_PCP008572	0.9616869837939649
pos_interaction	F_max_interaction_PCP025797	0.9617436835060343
pos_interaction	F_max_interaction_PCP002022	0.9618494156624345
pos_interaction	F_max_interaction_PCP024179	0.9621298146561897
pos_interaction	F_max_interaction_PCP041893	0.9626606461546201
pos_interaction	F_max_interaction_PCP021025	0.9626734536895383
pos_interaction	F_max_interaction_PCP018084	0.962978995592626
pos_interaction	F_max_interaction_PCP009020	0.9629990990742465
pos_interaction	F_max_interaction_PCP036199	0.9630966617292755
pos_interaction	F_max_interaction_PCP035412	0.963141859635216
pos_interaction	F_max_interaction_PCP003826	0.9633641869910942
pos_interaction	F_max_interaction_PCP023233	0.9634476944807074
pos_interaction	F_max_interaction_PCP005409	0.963679967050657
pos_interaction	F_max_interaction_PCP019466	0.963967044247871
pos_interaction	F_max_interaction_PCP000118	0.9644531109510337
pos_interaction	F_max_interaction_PCP030616	0.9646210849425596
pos_interaction	F_max_interaction_PCP029258	0.9655130571970676
pos_interaction	F_max_interaction_PCP008537	0.9656552817350256
pos_interaction	F_max_interaction_PCP011013	0.9658265034154871
pos_interaction	F_max_interaction_PCP002706	0.9658635873353574
pos_interaction	F_max_interaction_PCP011950	0.9659016834854471
pos_interaction	F_max_interaction_PCP018333	0.9659667379371061

pos_interaction	F_max_interaction_PCP029690	0.9661573627451797
pos_interaction	F_max_interaction_PCP016361	0.9663745322490098
pos_interaction	F_max_interaction_PCP018119	0.9671808693638083
pos_interaction	F_max_interaction_PCP024364	0.9672892395952428
pos_interaction	F_max_interaction_PCP013388	0.9677673155679183
pos_interaction	F_max_interaction_PCP002442	0.9679239704111743
pos_interaction	F_max_interaction_PCP009727	0.9682740165633505
pos_interaction	F_max_interaction_PCP006880	0.9683087479637839
pos_interaction	F_max_interaction_PCP005226	0.9683706999499411
pos_interaction	F_max_interaction_PCP012899	0.9685880432756853
pos_interaction	F_max_interaction_PCP032175	0.9692249969115024
pos_interaction	F_max_interaction_PCP007325	0.9694196399617379
pos_interaction	F_max_interaction_PCP010905	0.9695497816501967
pos_interaction	F_max_interaction_PCP025818	0.9704972253781688
pos_interaction	F_max_interaction_PCP025330	0.9707415492148834
pos_interaction	F_max_interaction_PCP000535	0.970850702307551
pos_interaction	F_max_interaction_PCP027241	0.9708779406892132
pos_interaction	F_max_interaction_PCP024835	0.9708815912003962
pos_interaction	F_max_interaction_PCP015289	0.9710027721735508
pos_interaction	F_max_interaction_PCP013539	0.9712221278212633
pos_interaction	F_max_interaction_PCP012628	0.9713092832857038
pos_interaction	F_max_interaction_PCP013091	0.9719854502748335
pos_interaction	F_max_interaction_PCP023823	0.9720580332920632
pos_interaction	F_max_interaction_PCP020355	0.9721769178275254
pos_interaction	F_max_interaction_PCP024932	0.9724763840431652
pos_interaction	F_max_interaction_PCP018093	0.972602670754235
pos_interaction	F_max_interaction_PCP009081	0.972812689176013
pos_interaction	F_max_interaction_PCP017600	0.9730189750333859
pos_interaction	F_max_interaction_PCP029520	0.9730836543003035
pos_interaction	F_max_interaction_PCP024618	0.9733560716937554
pos_interaction	F_max_interaction_PCP002193	0.9734549389958091
pos_interaction	F_max_interaction_PCP019562	0.9735902691507486
pos_interaction	F_max_interaction_PCP023195	0.9736052676668911
pos_interaction	F_max_interaction_PCP041151	0.9741457313822718
pos_interaction	F_max_interaction_PCP029326	0.9741944489926356
pos_interaction	F_max_interaction_PCP005383	0.9743984952342049
pos_interaction	F_max_interaction_PCP025929	0.9746632814897994
pos_interaction	F_max_interaction_PCP029669	0.9748162960305213
pos_interaction	F_max_interaction_PCP024805	0.9750095816845793
pos_interaction	F_max_interaction_PCP016781	0.9751265833276846
pos_interaction	F_max_interaction_PCP003285	0.9751864698957602
pos_interaction	F_max_interaction_PCP007333	0.9752805498652897
pos_interaction	F_max_interaction_PCP026164	0.9753866371101996
pos_interaction	F_max_interaction_PCP017342	0.9755628508383964
pos_interaction	F_max_interaction_PCP000387	0.9755951912474762
pos_interaction	F_max_interaction_PCP015739	0.9760350529189888

pos_interaction	F_max_interaction_PCP023759	0.976398039082554
pos_interaction	F_max_interaction_PCP033597	0.9766890301913151
pos_interaction	F_max_interaction_PCP006961	0.976869692665302
pos_interaction	F_max_interaction_PCP011867	0.9770764571095784
pos_interaction	F_max_interaction_PCP039702	0.9775003320587695
pos_interaction	F_max_interaction_PCP021477	0.9776501667162952
pos_interaction	F_max_interaction_PCP005863	0.9778974202527162
pos_interaction	F_max_interaction_PCP012044	0.9786231913621144
pos_interaction	F_max_interaction_PCP012039	0.9787134643408756
pos_interaction	F_max_interaction_PCP042778	0.9789047423375978
pos_interaction	F_max_interaction_PCP025793	0.9790066174182429
pos_interaction	F_max_interaction_PCP026145	0.9793159349839919
pos_interaction	F_max_interaction_PCP011597	0.9793426022910694
pos_interaction	F_max_interaction_PCP008307	0.9796682183084937
pos_interaction	F_max_interaction_PCP013053	0.9800318528285441
pos_interaction	F_max_interaction_PCP009532	0.9810193414234034
pos_interaction	F_max_interaction_PCP014232	0.9812949817153925
pos_interaction	F_max_interaction_PCP016072	0.9816966976822504
pos_interaction	F_max_interaction_PCP022825	0.9818251923695596
pos_interaction	F_max_interaction_PCP023916	0.9819925737387323
pos_interaction	F_max_interaction_PCP010675	0.9821435506674422
pos_interaction	F_max_interaction_PCP010597	0.9823971155218086
pos_interaction	F_max_interaction_PCP013616	0.9832289132944829
pos_interaction	F_max_interaction_PCP012225	0.9832841469653065
pos_interaction	F_max_interaction_PCP030207	0.9833167705880872
pos_interaction	F_max_interaction_PCP044677	0.9833888747687392
pos_interaction	F_max_interaction_PCP031705	0.9836353953232132
pos_interaction	F_max_interaction_PCP030120	0.984028748249437
pos_interaction	F_max_interaction_PCP022854	0.9840597846167374
pos_interaction	F_max_interaction_PCP023637	0.9843017134528834
pos_interaction	F_max_interaction_PCP008611	0.9845399498581934
pos_interaction	F_max_interaction_PCP019427	0.9846911002188483
pos_interaction	F_max_interaction_PCP032893	0.9846993131928783
pos_interaction	F_max_interaction_PCP032025	0.9849086910091613
pos_interaction	F_max_interaction_PCP014364	0.9851557140158719
pos_interaction	F_max_interaction_PCP015860	0.9852354543340397
pos_interaction	F_max_interaction_PCP008418	0.9857080151160783
pos_interaction	F_max_interaction_PCP007739	0.985858588355876
pos_interaction	F_max_interaction_PCP001691	0.9861499681520733
pos_interaction	F_max_interaction_PCP023142	0.9868778171348198
pos_interaction	F_max_interaction_PCP006791	0.9870249548037471
pos_interaction	F_max_interaction_PCP007629	0.9870453691074466
pos_interaction	F_max_interaction_PCP001968	0.9871288020951999
pos_interaction	F_max_interaction_PCP016502	0.9872845888815668
pos_interaction	F_max_interaction_PCP008974	0.9873142672897257
pos_interaction	F_max_interaction_PCP026440	0.9873721486460871

pos_interaction	F_max_interaction_PCP040594	0.9874452597536774
pos_interaction	F_max_interaction_PCP004331	0.9875930310547296
pos_interaction	F_max_interaction_PCP007628	0.9884844656139179
pos_interaction	F_max_interaction_PCP016179	0.9887053348232446
pos_interaction	F_max_interaction_PCP007189	0.9887290871654909
pos_interaction	F_max_interaction_PCP036761	0.9889982167723219
pos_interaction	F_max_interaction_PCP023425	0.9890228821986128
pos_interaction	F_max_interaction_PCP024510	0.9895997897530964
pos_interaction	F_max_interaction_PCP019752	0.9897509634014603
pos_interaction	F_max_interaction_PCP007714	0.990214210283074
pos_interaction	F_max_interaction_PCP020126	0.9905779122523264
pos_interaction	F_max_interaction_PCP029972	0.9906834280857565
pos_interaction	F_max_interaction_PCP020662	0.9912076442155132
pos_interaction	F_max_interaction_PCP036209	0.9912396924197867
pos_interaction	F_max_interaction_PCP022615	0.9912885688534941
pos_interaction	F_max_interaction_PCP017302	0.9913894536905041
pos_interaction	F_max_interaction_PCP004198	0.9916660329667002
pos_interaction	F_max_interaction_PCP025218	0.9917936287870748
pos_interaction	F_max_interaction_PCP021931	0.9920593765584416
pos_interaction	F_max_interaction_PCP017243	0.9925352013020766
pos_interaction	F_max_interaction_PCP034953	0.9927632737950389
pos_interaction	F_max_interaction_PCP041653	0.9928228105214897
pos_interaction	F_max_interaction_PCP019489	0.9929263458097791
pos_interaction	F_max_interaction_PCP008538	0.993429736567977
pos_interaction	F_max_interaction_PCP015294	0.9937152123507398
pos_interaction	F_max_interaction_PCP027769	0.9938342109613099
pos_interaction	F_max_interaction_PCP042186	0.9947339643294721
pos_interaction	F_max_interaction_PCP036482	0.9948679027502545
pos_interaction	F_max_interaction_PCP011011	0.9949053243538425
pos_interaction	F_max_interaction_PCP020779	0.9956717978298362
pos_interaction	F_max_interaction_PCP034358	0.9956748268904991
pos_interaction	F_max_interaction_PCP017660	0.9958352582516051
pos_interaction	F_max_interaction_PCP009837	0.9958408691397015
pos_interaction	F_max_interaction_PCP021796	0.995974243426042
pos_interaction	F_max_interaction_PCP006452	0.9960322542174783
pos_interaction	F_max_interaction_PCP008897	0.9966434230800663
pos_interaction	F_max_interaction_PCP042842	0.9967486335896131
pos_interaction	F_max_interaction_PCP030738	0.9972064351404585
pos_interaction	F_max_interaction_PCP035592	0.9972115195399233
pos_interaction	F_max_interaction_PCP012289	0.9973495687797436
pos_interaction	F_max_interaction_PCP013432	0.9973774153076526
pos_interaction	F_max_interaction_PCP000075	0.9974326251038016
pos_interaction	F_max_interaction_PCP028294	0.9979130597083181
pos_interaction	F_max_interaction_PCP019577	0.9983069383167706
pos_interaction	F_max_interaction_PCP025082	0.9984559213752698
pos_interaction	F_max_interaction_PCP019707	0.998526532380294

pos_interaction	F_max_interaction_PCP000407	0.9985984122102679
pos_interaction	F_max_interaction_PCP044447	0.9990774539883003
pos_interaction	F_max_interaction_PCP030751	0.9991188269445793

**interaction (Firmness)**	**name**	**Strength**
neg_interaction	Firmness_interaction_PCP023663	-0.9500987293409108
neg_interaction	Firmness_interaction_PCP018076	-0.9501613376898963
neg_interaction	Firmness_interaction_PCP007148	-0.9501743950201011
neg_interaction	Firmness_interaction_PCP044271	-0.9501750002118906
neg_interaction	Firmness_interaction_PCP043823	-0.9503273385524306
neg_interaction	Firmness_interaction_PCP042749	-0.9503894264468606
neg_interaction	Firmness_interaction_PCP025294	-0.9503921171772906
neg_interaction	Firmness_interaction_PCP029800	-0.9505040329090185
neg_interaction	Firmness_interaction_PCP027838	-0.9506483197406426
neg_interaction	Firmness_interaction_PCP015560	-0.9506602500602985
neg_interaction	Firmness_interaction_PCP009952	-0.950701935175218
neg_interaction	Firmness_interaction_PCP017519	-0.9509078758470977
neg_interaction	Firmness_interaction_PCP012281	-0.9509280706879814
neg_interaction	Firmness_interaction_PCP044118	-0.950956273405796
neg_interaction	Firmness_interaction_PCP006014	-0.95101155932472
neg_interaction	Firmness_interaction_PCP016419	-0.951030387415019
neg_interaction	Firmness_interaction_PCP010167	-0.9512301894833433
neg_interaction	Firmness_interaction_PCP023246	-0.9513924600318197
neg_interaction	Firmness_interaction_PCP030958	-0.9514795646190289
neg_interaction	Firmness_interaction_PCP005324	-0.9515941384687678
neg_interaction	Firmness_interaction_PCP032163	-0.9516007146728876
neg_interaction	Firmness_interaction_PCP036017	-0.9516082469276924
neg_interaction	Firmness_interaction_PCP009130	-0.9516479443710248
neg_interaction	Firmness_interaction_PCP004116	-0.951745610158335
neg_interaction	Firmness_interaction_PCP017187	-0.9518501732409076
neg_interaction	Firmness_interaction_PCP033244	-0.9519643413265836
neg_interaction	Firmness_interaction_PCP006999	-0.9519694904265297
neg_interaction	Firmness_interaction_PCP021808	-0.9520949340305767
neg_interaction	Firmness_interaction_PCP028974	-0.9521296801612863
neg_interaction	Firmness_interaction_PCP027939	-0.9521381505490945
neg_interaction	Firmness_interaction_PCP035847	-0.9521515432185521
neg_interaction	Firmness_interaction_PCP018043	-0.9522264131517026
neg_interaction	Firmness_interaction_PCP003953	-0.9524127864957337
neg_interaction	Firmness_interaction_PCP040177	-0.9524128443851736
neg_interaction	Firmness_interaction_PCP001817	-0.9524338455941083
neg_interaction	Firmness_interaction_PCP011348	-0.9525338486813394
neg_interaction	Firmness_interaction_PCP031156	-0.9525514043553943
neg_interaction	Firmness_interaction_PCP002656	-0.9525967088764398
neg_interaction	Firmness_interaction_PCP029387	-0.9526329133446292
neg_interaction	Firmness_interaction_PCP029660	-0.9526484142784511

neg_interaction	Firmness_interaction_PCP025181	-0.9526799026234717
neg_interaction	Firmness_interaction_PCP009685	-0.9529754840932386
neg_interaction	Firmness_interaction_PCP013657	-0.9530216157218251
neg_interaction	Firmness_interaction_PCP039588	-0.9530776816152449
neg_interaction	Firmness_interaction_PCP017958	-0.9534004066493433
neg_interaction	Firmness_interaction_PCP001711	-0.9534344110020285
neg_interaction	Firmness_interaction_PCP022495	-0.9534423448613829
neg_interaction	Firmness_interaction_PCP043175	-0.9534786013849745
neg_interaction	Firmness_interaction_PCP030565	-0.9535127421312859
neg_interaction	Firmness_interaction_PCP023127	-0.9536307755739373
neg_interaction	Firmness_interaction_PCP044528	-0.953732560884995
neg_interaction	Firmness_interaction_PCP021953	-0.9537457697382414
neg_interaction	Firmness_interaction_PCP021692	-0.9537606278882627
neg_interaction	Firmness_interaction_PCP003725	-0.9537716062689018
neg_interaction	Firmness_interaction_PCP019641	-0.953791094550047
neg_interaction	Firmness_interaction_PCP021484	-0.9538699972166469
neg_interaction	Firmness_interaction_PCP012334	-0.9539016484440999
neg_interaction	Firmness_interaction_PCP029508	-0.9540516082783179
neg_interaction	Firmness_interaction_PCP014816	-0.9541614832384157
neg_interaction	Firmness_interaction_PCP007637	-0.9541987927186408
neg_interaction	Firmness_interaction_PCP040532	-0.9542195230712436
neg_interaction	Firmness_interaction_PCP005917	-0.9542207608042779
neg_interaction	Firmness_interaction_PCP006679	-0.95441173172876
neg_interaction	Firmness_interaction_PCP018551	-0.9545136022765626
neg_interaction	Firmness_interaction_PCP005591	-0.9548720758052816
neg_interaction	Firmness_interaction_PCP000244	-0.9548789105934942
neg_interaction	Firmness_interaction_PCP024828	-0.9549970137919731
neg_interaction	Firmness_interaction_PCP003569	-0.9550014147234553
neg_interaction	Firmness_interaction_PCP028919	-0.9550466495020642
neg_interaction	Firmness_interaction_PCP000374	-0.9552443886423988
neg_interaction	Firmness_interaction_PCP029049	-0.9553601624424691
neg_interaction	Firmness_interaction_PCP002344	-0.95541922644423
neg_interaction	Firmness_interaction_PCP029420	-0.9554898174714558
neg_interaction	Firmness_interaction_PCP005668	-0.9556359940257795
neg_interaction	Firmness_interaction_PCP024630	-0.955684353284117
neg_interaction	Firmness_interaction_PCP016358	-0.9559066284761811
neg_interaction	Firmness_interaction_PCP007205	-0.9559338638485368
neg_interaction	Firmness_interaction_PCP038033	-0.9561164386650546
neg_interaction	Firmness_interaction_PCP003290	-0.9561207894876173
neg_interaction	Firmness_interaction_PCP005757	-0.956151578699835
neg_interaction	Firmness_interaction_PCP003650	-0.9562595353842043
neg_interaction	Firmness_interaction_PCP022484	-0.9562953664081869
neg_interaction	Firmness_interaction_PCP026281	-0.9563288300384304
neg_interaction	Firmness_interaction_PCP035107	-0.9564145521075804
neg_interaction	Firmness_interaction_PCP016478	-0.9564566215696713
neg_interaction	Firmness_interaction_PCP015360	-0.9564597646600737

neg_interaction	Firmness_interaction_PCP011437	-0.956601402557766
neg_interaction	Firmness_interaction_PCP025458	-0.9566183165656994
neg_interaction	Firmness_interaction_PCP005980	-0.9566515328415508
neg_interaction	Firmness_interaction_PCP044943	-0.9567066008298245
neg_interaction	Firmness_interaction_PCP020123	-0.9567944064547428
neg_interaction	Firmness_interaction_PCP041420	-0.957113679040366
neg_interaction	Firmness_interaction_PCP018572	-0.9571554326571137
neg_interaction	Firmness_interaction_PCP025143	-0.9573175075113523
neg_interaction	Firmness_interaction_PCP028845	-0.9573234283099222
neg_interaction	Firmness_interaction_PCP039650	-0.957678744715591
neg_interaction	Firmness_interaction_PCP009610	-0.9577518528314489
neg_interaction	Firmness_interaction_PCP011954	-0.9578050984651407
neg_interaction	Firmness_interaction_PCP006796	-0.9580873035363092
neg_interaction	Firmness_interaction_PCP038137	-0.9581766823393263
neg_interaction	Firmness_interaction_PCP015435	-0.9582466414738877
neg_interaction	Firmness_interaction_PCP020005	-0.9582834014982918
neg_interaction	Firmness_interaction_PCP045012	-0.9583270435271976
neg_interaction	Firmness_interaction_PCP034957	-0.9584471188402947
neg_interaction	Firmness_interaction_PCP015523	-0.9589742648782466
neg_interaction	Firmness_interaction_PCP025832	-0.9590674328314496
neg_interaction	Firmness_interaction_PCP010829	-0.9592653889474531
neg_interaction	Firmness_interaction_PCP032427	-0.9593067742153583
neg_interaction	Firmness_interaction_PCP035557	-0.9593412860104817
neg_interaction	Firmness_interaction_PCP005843	-0.9593491382223239
neg_interaction	Firmness_interaction_PCP003461	-0.9593518345118285
neg_interaction	Firmness_interaction_PCP007025	-0.9594705192095188
neg_interaction	Firmness_interaction_PCP022637	-0.9594737608594861
neg_interaction	Firmness_interaction_PCP017230	-0.9594871319223703
neg_interaction	Firmness_interaction_PCP027610	-0.959789574569161
neg_interaction	Firmness_interaction_PCP013603	-0.9599472761717834
neg_interaction	Firmness_interaction_PCP025057	-0.9599524124878538
neg_interaction	Firmness_interaction_PCP021730	-0.9600385793738535
neg_interaction	Firmness_interaction_PCP007219	-0.9601567923690062
neg_interaction	Firmness_interaction_PCP012685	-0.9601993184017954
neg_interaction	Firmness_interaction_PCP020380	-0.9602670327865539
neg_interaction	Firmness_interaction_PCP018811	-0.9603758255639269
neg_interaction	Firmness_interaction_PCP004145	-0.960428910032815
neg_interaction	Firmness_interaction_PCP020027	-0.9604756421038273
neg_interaction	Firmness_interaction_PCP003906	-0.9605181336925602
neg_interaction	Firmness_interaction_PCP016130	-0.9605319388593272
neg_interaction	Firmness_interaction_PCP019849	-0.9605694516209349
neg_interaction	Firmness_interaction_PCP023340	-0.9606122029176758
neg_interaction	Firmness_interaction_PCP021245	-0.9606835516737666
neg_interaction	Firmness_interaction_PCP044429	-0.9608089594949146
neg_interaction	Firmness_interaction_PCP014191	-0.9609414741350385
neg_interaction	Firmness_interaction_PCP010839	-0.9609476296078165

neg_interaction	Firmness_interaction_PCP043760	-0.9610481078653675
neg_interaction	Firmness_interaction_PCP024515	-0.9611824334044221
neg_interaction	Firmness_interaction_PCP005828	-0.9614703904595621
neg_interaction	Firmness_interaction_PCP020562	-0.9614724490798572
neg_interaction	Firmness_interaction_PCP025182	-0.9614810828804072
neg_interaction	Firmness_interaction_PCP024282	-0.9616339845834503
neg_interaction	Firmness_interaction_PCP008428	-0.9617028007812298
neg_interaction	Firmness_interaction_PCP014018	-0.9619193750547562
neg_interaction	Firmness_interaction_PCP001098	-0.9620015762068858
neg_interaction	Firmness_interaction_PCP028917	-0.9620345677366806
neg_interaction	Firmness_interaction_PCP024348	-0.962080017094495
neg_interaction	Firmness_interaction_PCP026564	-0.9621552315662021
neg_interaction	Firmness_interaction_PCP009792	-0.9622234205094002
neg_interaction	Firmness_interaction_PCP013590	-0.9622250519149147
neg_interaction	Firmness_interaction_PCP001799	-0.9622851512956611
neg_interaction	Firmness_interaction_PCP003398	-0.9623085214105445
neg_interaction	Firmness_interaction_PCP016633	-0.9623759664513368
neg_interaction	Firmness_interaction_PCP044104	-0.9624963156242806
neg_interaction	Firmness_interaction_PCP018461	-0.9627227473799261
neg_interaction	Firmness_interaction_PCP007585	-0.9627768475569132
neg_interaction	Firmness_interaction_PCP042861	-0.9630071697557963
neg_interaction	Firmness_interaction_PCP025746	-0.9631526108958035
neg_interaction	Firmness_interaction_PCP044194	-0.9632346361474565
neg_interaction	Firmness_interaction_PCP019509	-0.9633278123427734
neg_interaction	Firmness_interaction_PCP016523	-0.9633851472580485
neg_interaction	Firmness_interaction_PCP027631	-0.9633994413655113
neg_interaction	Firmness_interaction_PCP022373	-0.963427317857494
neg_interaction	Firmness_interaction_PCP006697	-0.9635814710331886
neg_interaction	Firmness_interaction_PCP001039	-0.9637043662574695
neg_interaction	Firmness_interaction_PCP006369	-0.9637183631122155
neg_interaction	Firmness_interaction_PCP022181	-0.9638338200261464
neg_interaction	Firmness_interaction_PCP000218	-0.9638964076961593
neg_interaction	Firmness_interaction_PCP044818	-0.9639426397272005
neg_interaction	Firmness_interaction_PCP027854	-0.9641671377642808
neg_interaction	Firmness_interaction_PCP022649	-0.9644557619921634
neg_interaction	Firmness_interaction_PCP023989	-0.9644899241217212
neg_interaction	Firmness_interaction_PCP024244	-0.964539447631384
neg_interaction	Firmness_interaction_PCP009578	-0.9645596027234584
neg_interaction	Firmness_interaction_PCP017796	-0.9645930597804855
neg_interaction	Firmness_interaction_PCP034698	-0.9647262770912148
neg_interaction	Firmness_interaction_PCP019666	-0.9649430751718345
neg_interaction	Firmness_interaction_PCP026233	-0.9649563974949348
neg_interaction	Firmness_interaction_PCP031325	-0.9649966368805419
neg_interaction	Firmness_interaction_PCP013128	-0.9653967729452245
neg_interaction	Firmness_interaction_PCP029007	-0.965430104161118
neg_interaction	Firmness_interaction_PCP044010	-0.9656352214652566

neg_interaction	Firmness_interaction_PCP028138	-0.9656761330805709
neg_interaction	Firmness_interaction_PCP001732	-0.9656937070605109
neg_interaction	Firmness_interaction_PCP014264	-0.9657110677737067
neg_interaction	Firmness_interaction_PCP037164	-0.9657125693645369
neg_interaction	Firmness_interaction_PCP000901	-0.9657175518665811
neg_interaction	Firmness_interaction_PCP001822	-0.9657565842930932
neg_interaction	Firmness_interaction_PCP008689	-0.9659144751744552
neg_interaction	Firmness_interaction_PCP016621	-0.9660082847711018
neg_interaction	Firmness_interaction_PCP042928	-0.9660412531175212
neg_interaction	Firmness_interaction_PCP027407	-0.9660667374759633
neg_interaction	Firmness_interaction_PCP024476	-0.9661338614526912
neg_interaction	Firmness_interaction_PCP038032	-0.9661788594303604
neg_interaction	Firmness_interaction_PCP022633	-0.966341879811196
neg_interaction	Firmness_interaction_PCP011745	-0.9665341053961295
neg_interaction	Firmness_interaction_PCP000116	-0.9668573033765475
neg_interaction	Firmness_interaction_PCP031271	-0.9669687381981492
neg_interaction	Firmness_interaction_PCP020545	-0.9670628246379686
neg_interaction	Firmness_interaction_PCP003789	-0.9670641872280756
neg_interaction	Firmness_interaction_PCP009428	-0.9671267832437167
neg_interaction	Firmness_interaction_PCP042839	-0.9671280259642449
neg_interaction	Firmness_interaction_PCP013003	-0.9671419835055529
neg_interaction	Firmness_interaction_PCP019182	-0.9672804607459957
neg_interaction	Firmness_interaction_PCP007419	-0.967489475457604
neg_interaction	Firmness_interaction_PCP045103	-0.9674900117076191
neg_interaction	Firmness_interaction_PCP011614	-0.9675636072263025
neg_interaction	Firmness_interaction_PCP045009	-0.967627886447297
neg_interaction	Firmness_interaction_PCP028054	-0.9677970504842757
neg_interaction	Firmness_interaction_PCP018220	-0.9679095333765992
neg_interaction	Firmness_interaction_PCP013454	-0.967955141506401
neg_interaction	Firmness_interaction_PCP027551	-0.968079878025011
neg_interaction	Firmness_interaction_PCP028494	-0.968571733114039
neg_interaction	Firmness_interaction_PCP023792	-0.9686614195919212
neg_interaction	Firmness_interaction_PCP022186	-0.9690541394710048
neg_interaction	Firmness_interaction_PCP001840	-0.9691599239169255
neg_interaction	Firmness_interaction_PCP032189	-0.9691837800956067
neg_interaction	Firmness_interaction_PCP017080	-0.9691900636041076
neg_interaction	Firmness_interaction_PCP021964	-0.9691980932407323
neg_interaction	Firmness_interaction_PCP014042	-0.9692384049742029
neg_interaction	Firmness_interaction_PCP005697	-0.9692543662012216
neg_interaction	Firmness_interaction_PCP006762	-0.9693772444352802
neg_interaction	Firmness_interaction_PCP014412	-0.9694259152056826
neg_interaction	Firmness_interaction_PCP027964	-0.9694397307659833
neg_interaction	Firmness_interaction_PCP007525	-0.9698730516260022
neg_interaction	Firmness_interaction_PCP034645	-0.9699069770585149
neg_interaction	Firmness_interaction_PCP039914	-0.9699626649276948
neg_interaction	Firmness_interaction_PCP044738	-0.970006147921666

neg_interaction	Firmness_interaction_PCP016638	-0.9700986115552216
neg_interaction	Firmness_interaction_PCP026299	-0.9701228023379597
neg_interaction	Firmness_interaction_PCP012959	-0.9703303291025122
neg_interaction	Firmness_interaction_PCP010900	-0.9703462772592183
neg_interaction	Firmness_interaction_PCP003757	-0.9704887020073933
neg_interaction	Firmness_interaction_PCP005284	-0.9705147977038648
neg_interaction	Firmness_interaction_PCP030791	-0.9705758743360438
neg_interaction	Firmness_interaction_PCP014994	-0.9706235044577739
neg_interaction	Firmness_interaction_PCP042567	-0.9707469870567917
neg_interaction	Firmness_interaction_PCP009501	-0.9708296617791325
neg_interaction	Firmness_interaction_PCP022006	-0.9709684396995095
neg_interaction	Firmness_interaction_PCP024731	-0.9710473788966129
neg_interaction	Firmness_interaction_PCP009369	-0.9710838149019989
neg_interaction	Firmness_interaction_PCP021406	-0.9711087584762709
neg_interaction	Firmness_interaction_PCP040138	-0.9711424864345957
neg_interaction	Firmness_interaction_PCP030542	-0.9711878948190812
neg_interaction	Firmness_interaction_PCP017890	-0.9712206349970839
neg_interaction	Firmness_interaction_PCP000614	-0.9712673061240212
neg_interaction	Firmness_interaction_PCP015053	-0.9713995824883356
neg_interaction	Firmness_interaction_PCP018666	-0.9714128657298164
neg_interaction	Firmness_interaction_PCP000094	-0.9714327038984454
neg_interaction	Firmness_interaction_PCP011360	-0.971613917709247
neg_interaction	Firmness_interaction_PCP013644	-0.9717450539698923
neg_interaction	Firmness_interaction_PCP043197	-0.9719245264597087
neg_interaction	Firmness_interaction_PCP013513	-0.9719649362204813
neg_interaction	Firmness_interaction_PCP019866	-0.9720263695668351
neg_interaction	Firmness_interaction_PCP044863	-0.9720495341042306
neg_interaction	Firmness_interaction_PCP001986	-0.972065268246645
neg_interaction	Firmness_interaction_PCP017830	-0.9720686163501451
neg_interaction	Firmness_interaction_PCP007476	-0.9722740837110456
neg_interaction	Firmness_interaction_PCP036035	-0.972676339454183
neg_interaction	Firmness_interaction_PCP023341	-0.9728178538849922
neg_interaction	Firmness_interaction_PCP012505	-0.9728331499659675
neg_interaction	Firmness_interaction_PCP036562	-0.9728725241146703
neg_interaction	Firmness_interaction_PCP027006	-0.9729091811198547
neg_interaction	Firmness_interaction_PCP005129	-0.9731125849407245
neg_interaction	Firmness_interaction_PCP042045	-0.9731400955763249
neg_interaction	Firmness_interaction_PCP020887	-0.9732722627966837
neg_interaction	Firmness_interaction_PCP043711	-0.9734127530873466
neg_interaction	Firmness_interaction_PCP042536	-0.9734130089032066
neg_interaction	Firmness_interaction_PCP007997	-0.9735072024383117
neg_interaction	Firmness_interaction_PCP011606	-0.97353715557127
neg_interaction	Firmness_interaction_PCP021948	-0.9738164250326856
neg_interaction	Firmness_interaction_PCP004181	-0.9739994092464422
neg_interaction	Firmness_interaction_PCP014950	-0.9741419762768597
neg_interaction	Firmness_interaction_PCP005629	-0.9743744099478863

neg_interaction	Firmness_interaction_PCP034181	-0.9744170179578611
neg_interaction	Firmness_interaction_PCP018636	-0.9745126396752241
neg_interaction	Firmness_interaction_PCP012059	-0.97454355553563
neg_interaction	Firmness_interaction_PCP040158	-0.9745844602010628
neg_interaction	Firmness_interaction_PCP008881	-0.9746611550635819
neg_interaction	Firmness_interaction_PCP022145	-0.9746698874561504
neg_interaction	Firmness_interaction_PCP012551	-0.9749728276149205
neg_interaction	Firmness_interaction_PCP002075	-0.974995594085163
neg_interaction	Firmness_interaction_PCP016856	-0.975052171345744
neg_interaction	Firmness_interaction_PCP007024	-0.9751723594793502
neg_interaction	Firmness_interaction_PCP032244	-0.9751812448657681
neg_interaction	Firmness_interaction_PCP012705	-0.9753378132307562
neg_interaction	Firmness_interaction_PCP003886	-0.9754758150449296
neg_interaction	Firmness_interaction_PCP012138	-0.9755150323712973
neg_interaction	Firmness_interaction_PCP028873	-0.9755430719984619
neg_interaction	Firmness_interaction_PCP002106	-0.9756836790192153
neg_interaction	Firmness_interaction_PCP008901	-0.9757252341933241
neg_interaction	Firmness_interaction_PCP012266	-0.9757556420378724
neg_interaction	Firmness_interaction_PCP013399	-0.9757822280930205
neg_interaction	Firmness_interaction_PCP005636	-0.9758197547023837
neg_interaction	Firmness_interaction_PCP003715	-0.9759275378827353
neg_interaction	Firmness_interaction_PCP000300	-0.9759594868593479
neg_interaction	Firmness_interaction_PCP023040	-0.9760053603526637
neg_interaction	Firmness_interaction_PCP009356	-0.9762017243280057
neg_interaction	Firmness_interaction_PCP001044	-0.9763888126042372
neg_interaction	Firmness_interaction_PCP005468	-0.9764581698899764
neg_interaction	Firmness_interaction_PCP017605	-0.9764985358609064
neg_interaction	Firmness_interaction_PCP014662	-0.97671214532608
neg_interaction	Firmness_interaction_PCP040424	-0.9767956187404567
neg_interaction	Firmness_interaction_PCP030891	-0.9768986931587005
neg_interaction	Firmness_interaction_PCP002057	-0.9769041031433083
neg_interaction	Firmness_interaction_PCP012078	-0.9769161917033129
neg_interaction	Firmness_interaction_PCP008395	-0.9769951798786113
neg_interaction	Firmness_interaction_PCP006737	-0.9769961985224648
neg_interaction	Firmness_interaction_PCP017043	-0.9770518850340723
neg_interaction	Firmness_interaction_PCP030440	-0.9771232487430569
neg_interaction	Firmness_interaction_PCP025874	-0.9774512265949618
neg_interaction	Firmness_interaction_PCP004423	-0.9777329179915925
neg_interaction	Firmness_interaction_PCP004361	-0.9779965791394711
neg_interaction	Firmness_interaction_PCP002206	-0.9780794108863963
neg_interaction	Firmness_interaction_PCP012009	-0.9781187613159206
neg_interaction	Firmness_interaction_PCP000150	-0.978177725871899
neg_interaction	Firmness_interaction_PCP028137	-0.9781786007340344
neg_interaction	Firmness_interaction_PCP028279	-0.9783135377448597
neg_interaction	Firmness_interaction_PCP004152	-0.9783829593518393
neg_interaction	Firmness_interaction_PCP023052	-0.9784560740910294

neg_interaction	Firmness_interaction_PCP030800	-0.9787202300460438
neg_interaction	Firmness_interaction_PCP024823	-0.9787748145727531
neg_interaction	Firmness_interaction_PCP003076	-0.9788609591933678
neg_interaction	Firmness_interaction_PCP008236	-0.978950529558207
neg_interaction	Firmness_interaction_PCP029756	-0.979162042191987
neg_interaction	Firmness_interaction_PCP044270	-0.9794512013235875
neg_interaction	Firmness_interaction_PCP002318	-0.9794622625906109
neg_interaction	Firmness_interaction_PCP005508	-0.9794800525440565
neg_interaction	Firmness_interaction_PCP029191	-0.9795431912862883
neg_interaction	Firmness_interaction_PCP010311	-0.9798146793258334
neg_interaction	Firmness_interaction_PCP001320	-0.9800131994351123
neg_interaction	Firmness_interaction_PCP014796	-0.9801460466613338
neg_interaction	Firmness_interaction_PCP002219	-0.9801815642454322
neg_interaction	Firmness_interaction_PCP039584	-0.9801915706242245
neg_interaction	Firmness_interaction_PCP028795	-0.9804678971866562
neg_interaction	Firmness_interaction_PCP013989	-0.9804892308621489
neg_interaction	Firmness_interaction_PCP026021	-0.9805185219614717
neg_interaction	Firmness_interaction_PCP003311	-0.9808084425080714
neg_interaction	Firmness_interaction_PCP019367	-0.9810001411877903
neg_interaction	Firmness_interaction_PCP020458	-0.9810511304853744
neg_interaction	Firmness_interaction_PCP044598	-0.9810624229771538
neg_interaction	Firmness_interaction_PCP012050	-0.9811823852430845
neg_interaction	Firmness_interaction_PCP008521	-0.981230224663977
neg_interaction	Firmness_interaction_PCP010800	-0.9812927601386428
neg_interaction	Firmness_interaction_PCP013496	-0.9815230615076213
neg_interaction	Firmness_interaction_PCP010395	-0.9815641396861902
neg_interaction	Firmness_interaction_PCP026217	-0.9816446768274417
neg_interaction	Firmness_interaction_PCP041960	-0.9816572299540699
neg_interaction	Firmness_interaction_PCP015888	-0.9816707250076814
neg_interaction	Firmness_interaction_PCP026151	-0.9816737062159362
neg_interaction	Firmness_interaction_PCP020036	-0.9817052878904943
neg_interaction	Firmness_interaction_PCP030584	-0.9818533187877208
neg_interaction	Firmness_interaction_PCP021689	-0.9818576044912324
neg_interaction	Firmness_interaction_PCP021880	-0.9819026871600113
neg_interaction	Firmness_interaction_PCP016282	-0.9819683363171128
neg_interaction	Firmness_interaction_PCP011018	-0.9821000460174635
neg_interaction	Firmness_interaction_PCP014171	-0.982170209884817
neg_interaction	Firmness_interaction_PCP025893	-0.982233031671566
neg_interaction	Firmness_interaction_PCP002911	-0.9824834177994304
neg_interaction	Firmness_interaction_PCP041092	-0.9824950399630543
neg_interaction	Firmness_interaction_PCP007570	-0.9825942629138521
neg_interaction	Firmness_interaction_PCP030615	-0.9829275994628156
neg_interaction	Firmness_interaction_PCP009879	-0.9829885266645685
neg_interaction	Firmness_interaction_PCP040479	-0.9829973743151325
neg_interaction	Firmness_interaction_PCP023705	-0.9830024520684835
neg_interaction	Firmness_interaction_PCP029082	-0.9833420226784171

neg_interaction	Firmness_interaction_PCP009783	-0.983474918364157
neg_interaction	Firmness_interaction_PCP022491	-0.9835492987157448
neg_interaction	Firmness_interaction_PCP032824	-0.983659974206397
neg_interaction	Firmness_interaction_PCP022894	-0.9840347407745971
neg_interaction	Firmness_interaction_PCP029665	-0.9840801177173193
neg_interaction	Firmness_interaction_PCP025880	-0.9841197078309902
neg_interaction	Firmness_interaction_PCP024466	-0.9842835702139743
neg_interaction	Firmness_interaction_PCP038637	-0.9843097017235068
neg_interaction	Firmness_interaction_PCP019586	-0.9844228917186643
neg_interaction	Firmness_interaction_PCP024127	-0.9846969521851769
neg_interaction	Firmness_interaction_PCP013401	-0.9847170791509483
neg_interaction	Firmness_interaction_PCP028951	-0.9847480422857131
neg_interaction	Firmness_interaction_PCP021924	-0.9847954914383402
neg_interaction	Firmness_interaction_PCP011322	-0.9849394842302552
neg_interaction	Firmness_interaction_PCP000857	-0.985013788594371
neg_interaction	Firmness_interaction_PCP040626	-0.9853032374262606
neg_interaction	Firmness_interaction_PCP014304	-0.9853096005584809
neg_interaction	Firmness_interaction_PCP004607	-0.9854100204392953
neg_interaction	Firmness_interaction_PCP004153	-0.9854212045425008
neg_interaction	Firmness_interaction_PCP022959	-0.9855292365616909
neg_interaction	Firmness_interaction_PCP016840	-0.9857796880260876
neg_interaction	Firmness_interaction_PCP041866	-0.9857997856859295
neg_interaction	Firmness_interaction_PCP031467	-0.9858513048914482
neg_interaction	Firmness_interaction_PCP024718	-0.9860597421491055
neg_interaction	Firmness_interaction_PCP042351	-0.9861649008426252
neg_interaction	Firmness_interaction_PCP026647	-0.9867054939268003
neg_interaction	Firmness_interaction_PCP009200	-0.9873563405789596
neg_interaction	Firmness_interaction_PCP030544	-0.9874336272915815
neg_interaction	Firmness_interaction_PCP004200	-0.9874848615972412
neg_interaction	Firmness_interaction_PCP017016	-0.9874896236667403
neg_interaction	Firmness_interaction_PCP026243	-0.9875927430426557
neg_interaction	Firmness_interaction_PCP018086	-0.9877192728242857
neg_interaction	Firmness_interaction_PCP015491	-0.9880638969211469
neg_interaction	Firmness_interaction_PCP025186	-0.9880749582159569
neg_interaction	Firmness_interaction_PCP011779	-0.9881050494008925
neg_interaction	Firmness_interaction_PCP028533	-0.9883375410074343
neg_interaction	Firmness_interaction_PCP043766	-0.9885881736235907
neg_interaction	Firmness_interaction_PCP005981	-0.9888590919803716
neg_interaction	Firmness_interaction_PCP027159	-0.9889648657778419
neg_interaction	Firmness_interaction_PCP042277	-0.9890695911305523
neg_interaction	Firmness_interaction_PCP020366	-0.9891828732239556
neg_interaction	Firmness_interaction_PCP008347	-0.9892760142819017
neg_interaction	Firmness_interaction_PCP007000	-0.9893907836257878
neg_interaction	Firmness_interaction_PCP016971	-0.9894060539664803
neg_interaction	Firmness_interaction_PCP029251	-0.9894442167339831
neg_interaction	Firmness_interaction_PCP008387	-0.9894865560015478

neg_interaction	Firmness_interaction_PCP029239	-0.9895178993001689
neg_interaction	Firmness_interaction_PCP013888	-0.989620951015365
neg_interaction	Firmness_interaction_PCP020117	-0.9896796855099026
neg_interaction	Firmness_interaction_PCP044637	-0.9897225236017366
neg_interaction	Firmness_interaction_PCP008688	-0.9897814051496635
neg_interaction	Firmness_interaction_PCP041732	-0.9898506219868775
neg_interaction	Firmness_interaction_PCP017758	-0.9899930106276531
neg_interaction	Firmness_interaction_PCP002740	-0.9900125056612195
neg_interaction	Firmness_interaction_PCP006224	-0.9900904449486735
neg_interaction	Firmness_interaction_PCP002124	-0.9901102747267104
neg_interaction	Firmness_interaction_PCP024439	-0.9905882180134504
neg_interaction	Firmness_interaction_PCP002435	-0.9906708602812908
neg_interaction	Firmness_interaction_PCP018424	-0.9906796076275821
neg_interaction	Firmness_interaction_PCP015729	-0.9907410990384593
neg_interaction	Firmness_interaction_PCP001097	-0.9909061213098085
neg_interaction	Firmness_interaction_PCP021679	-0.9909285657591506
neg_interaction	Firmness_interaction_PCP027606	-0.9909616875511423
neg_interaction	Firmness_interaction_PCP026014	-0.9911459114687828
neg_interaction	Firmness_interaction_PCP024781	-0.9912324287715568
neg_interaction	Firmness_interaction_PCP005638	-0.9912754185586921
neg_interaction	Firmness_interaction_PCP031562	-0.9913330296201303
neg_interaction	Firmness_interaction_PCP014515	-0.9914040871117709
neg_interaction	Firmness_interaction_PCP012231	-0.9916256763365396
neg_interaction	Firmness_interaction_PCP026800	-0.9918278402047577
neg_interaction	Firmness_interaction_PCP021185	-0.9919546535308955
neg_interaction	Firmness_interaction_PCP004420	-0.9921286530607524
neg_interaction	Firmness_interaction_PCP019597	-0.9921511633527503
neg_interaction	Firmness_interaction_PCP009919	-0.992446416218263
neg_interaction	Firmness_interaction_PCP031396	-0.9926175144045606
neg_interaction	Firmness_interaction_PCP009753	-0.9926839380984
neg_interaction	Firmness_interaction_PCP021367	-0.9927098059974453
neg_interaction	Firmness_interaction_PCP024738	-0.9927884803932928
neg_interaction	Firmness_interaction_PCP022985	-0.9928231091056736
neg_interaction	Firmness_interaction_PCP044272	-0.9928623867712157
neg_interaction	Firmness_interaction_PCP008180	-0.9930154990063202
neg_interaction	Firmness_interaction_PCP026432	-0.9930713092304182
neg_interaction	Firmness_interaction_PCP007785	-0.993093488401597
neg_interaction	Firmness_interaction_PCP014077	-0.9933368488843541
neg_interaction	Firmness_interaction_PCP031186	-0.9934440215972798
neg_interaction	Firmness_interaction_PCP011731	-0.9936261018799006
neg_interaction	Firmness_interaction_PCP023329	-0.993665908341458
neg_interaction	Firmness_interaction_PCP027077	-0.9939188432466153
neg_interaction	Firmness_interaction_PCP014088	-0.9940773012550611
neg_interaction	Firmness_interaction_PCP024140	-0.9942525437036995
neg_interaction	Firmness_interaction_PCP002298	-0.9944430599444071
neg_interaction	Firmness_interaction_PCP012166	-0.9946090246137925

neg_interaction	Firmness_interaction_PCP001793	-0.9946588572720007
neg_interaction	Firmness_interaction_PCP021754	-0.9947549243875474
neg_interaction	Firmness_interaction_PCP006327	-0.9948108892471887
neg_interaction	Firmness_interaction_PCP006190	-0.9949960096765255
neg_interaction	Firmness_interaction_PCP034470	-0.9950875639888209
neg_interaction	Firmness_interaction_PCP017208	-0.9952861294485604
neg_interaction	Firmness_interaction_PCP015521	-0.9953682816745487
neg_interaction	Firmness_interaction_PCP003339	-0.995442638419624
neg_interaction	Firmness_interaction_PCP011678	-0.9956132596455247
neg_interaction	Firmness_interaction_PCP031453	-0.9960118936818387
neg_interaction	Firmness_interaction_PCP036745	-0.9961787956357636
neg_interaction	Firmness_interaction_PCP011023	-0.9961997791270345
neg_interaction	Firmness_interaction_PCP017955	-0.9964390093485871
neg_interaction	Firmness_interaction_PCP011452	-0.996494999061546
neg_interaction	Firmness_interaction_PCP027675	-0.9965784664958881
neg_interaction	Firmness_interaction_PCP013976	-0.9967152100048847
neg_interaction	Firmness_interaction_PCP004014	-0.9967611347126937
neg_interaction	Firmness_interaction_PCP027928	-0.9967845661130442
neg_interaction	Firmness_interaction_PCP004573	-0.9968975190142186
neg_interaction	Firmness_interaction_PCP021189	-0.9970434590011046
neg_interaction	Firmness_interaction_PCP010491	-0.9972977047293379
neg_interaction	Firmness_interaction_PCP000538	-0.9975777243817013
neg_interaction	Firmness_interaction_PCP023623	-0.9976397371363112
neg_interaction	Firmness_interaction_PCP008900	-0.9977484309641287
neg_interaction	Firmness_interaction_PCP006768	-0.9980631348192514
neg_interaction	Firmness_interaction_PCP039793	-0.9982681280622843
neg_interaction	Firmness_interaction_PCP010888	-0.9982911275301496
neg_interaction	Firmness_interaction_PCP043214	-0.9983234773495314
neg_interaction	Firmness_interaction_PCP017440	-0.9983644580787115
neg_interaction	Firmness_interaction_PCP005162	-0.9984390047617282
neg_interaction	Firmness_interaction_PCP024222	-0.9984491133938406
neg_interaction	Firmness_interaction_PCP030893	-0.9986948707156158
neg_interaction	Firmness_interaction_PCP013597	-0.9987804090518695
neg_interaction	Firmness_interaction_PCP037604	-0.9990908085284317
neg_interaction	Firmness_interaction_PCP044040	-0.9992113752532393
neg_interaction	Firmness_interaction_PCP000204	-0.9992579823941758
neg_interaction	Firmness_interaction_PCP010814	-0.9994244051025057
pos_interaction	Firmness_interaction_PCP039467	0.9500053766959518
pos_interaction	Firmness_interaction_PCP021038	0.9500629169411768
pos_interaction	Firmness_interaction_PCP022364	0.9501273846745764
pos_interaction	Firmness_interaction_PCP017243	0.9502843358592271
pos_interaction	Firmness_interaction_PCP025395	0.9503096796110891
pos_interaction	Firmness_interaction_PCP030840	0.9503278490566942
pos_interaction	Firmness_interaction_PCP037547	0.9506082008285639
pos_interaction	Firmness_interaction_PCP029972	0.9508536971079413

pos_interaction	Firmness_interaction_PCP015930	0.9509829780199877
pos_interaction	Firmness_interaction_PCP009441	0.9512631919189215
pos_interaction	Firmness_interaction_PCP016790	0.9512716683334824
pos_interaction	Firmness_interaction_PCP017793	0.9513000161189038
pos_interaction	Firmness_interaction_PCP016718	0.9513488052900495
pos_interaction	Firmness_interaction_PCP008897	0.9514861378392024
pos_interaction	Firmness_interaction_PCP026310	0.9516891808294059
pos_interaction	Firmness_interaction_PCP022886	0.9516904679991325
pos_interaction	Firmness_interaction_PCP019776	0.951697443049827
pos_interaction	Firmness_interaction_PCP013355	0.9517068771086052
pos_interaction	Firmness_interaction_PCP026981	0.9517321813668307
pos_interaction	Firmness_interaction_PCP044845	0.9517606316811673
pos_interaction	Firmness_interaction_PCP023645	0.9517803874699754
pos_interaction	Firmness_interaction_PCP028160	0.951803510924949
pos_interaction	Firmness_interaction_PCP034458	0.9518885761351193
pos_interaction	Firmness_interaction_PCP019950	0.9518993295869571
pos_interaction	Firmness_interaction_PCP028903	0.9521319126952884
pos_interaction	Firmness_interaction_PCP012173	0.9521917121097101
pos_interaction	Firmness_interaction_PCP003826	0.952278104992597
pos_interaction	Firmness_interaction_PCP010112	0.9523054673212676
pos_interaction	Firmness_interaction_PCP004146	0.9525695065363957
pos_interaction	Firmness_interaction_PCP036502	0.9527512442563031
pos_interaction	Firmness_interaction_PCP035298	0.9529072557644855
pos_interaction	Firmness_interaction_PCP016623	0.9531175824260948
pos_interaction	Firmness_interaction_PCP034042	0.9531429723316972
pos_interaction	Firmness_interaction_PCP006682	0.9532874657828654
pos_interaction	Firmness_interaction_PCP025683	0.9535052840028253
pos_interaction	Firmness_interaction_PCP040196	0.9535137445252714
pos_interaction	Firmness_interaction_PCP010905	0.9535451409726812
pos_interaction	Firmness_interaction_PCP006654	0.9535836088519553
pos_interaction	Firmness_interaction_PCP039351	0.9536567603294586
pos_interaction	Firmness_interaction_PCP029711	0.9538294962050432
pos_interaction	Firmness_interaction_PCP012340	0.9539124969380036
pos_interaction	Firmness_interaction_PCP028759	0.9539882156465409
pos_interaction	Firmness_interaction_PCP021658	0.9543190795730067
pos_interaction	Firmness_interaction_PCP000349	0.9543938146693066
pos_interaction	Firmness_interaction_PCP022658	0.9545262054602274
pos_interaction	Firmness_interaction_PCP000591	0.954546260286199
pos_interaction	Firmness_interaction_PCP030738	0.9546047119280314
pos_interaction	Firmness_interaction_PCP012676	0.9546768020854416
pos_interaction	Firmness_interaction_PCP012344	0.9549030183327503
pos_interaction	Firmness_interaction_PCP020216	0.9550049748156856
pos_interaction	Firmness_interaction_PCP040381	0.9551443591319921
pos_interaction	Firmness_interaction_PCP007836	0.9552103709798825
pos_interaction	Firmness_interaction_PCP041357	0.9552854071917793
pos_interaction	Firmness_interaction_PCP005237	0.9553471931523738

pos_interaction	Firmness_interaction_PCP009257	0.955487829819279
pos_interaction	Firmness_interaction_PCP016086	0.9556412922496564
pos_interaction	Firmness_interaction_PCP004900	0.9557037793718968
pos_interaction	Firmness_interaction_PCP015289	0.9557388259463097
pos_interaction	Firmness_interaction_PCP010690	0.9557500440069715
pos_interaction	Firmness_interaction_PCP006866	0.9558246958154939
pos_interaction	Firmness_interaction_PCP026930	0.9560976726477253
pos_interaction	Firmness_interaction_PCP005647	0.9561063539740844
pos_interaction	Firmness_interaction_PCP019872	0.9561076801931877
pos_interaction	Firmness_interaction_PCP023233	0.9563604100881583
pos_interaction	Firmness_interaction_PCP006662	0.9563815611701084
pos_interaction	Firmness_interaction_PCP002193	0.9565095344335738
pos_interaction	Firmness_interaction_PCP027122	0.9566268235419825
pos_interaction	Firmness_interaction_PCP007191	0.9567079252307915
pos_interaction	Firmness_interaction_PCP017900	0.9567533566014819
pos_interaction	Firmness_interaction_PCP011059	0.9569416639742703
pos_interaction	Firmness_interaction_PCP042402	0.9569558082787482
pos_interaction	Firmness_interaction_PCP030844	0.9571782469839134
pos_interaction	Firmness_interaction_PCP028294	0.9573120130858458
pos_interaction	Firmness_interaction_PCP011154	0.9573221764708058
pos_interaction	Firmness_interaction_PCP016598	0.9573392969358944
pos_interaction	Firmness_interaction_PCP030616	0.9573628357030922
pos_interaction	Firmness_interaction_PCP039637	0.9575235444169083
pos_interaction	Firmness_interaction_PCP009585	0.9575591378947865
pos_interaction	Firmness_interaction_PCP008418	0.9576081777131772
pos_interaction	Firmness_interaction_PCP027041	0.957762665047191
pos_interaction	Firmness_interaction_PCP010675	0.9577924487421198
pos_interaction	Firmness_interaction_PCP007614	0.9579244183332201
pos_interaction	Firmness_interaction_PCP012575	0.9580174412179739
pos_interaction	Firmness_interaction_PCP006627	0.9583506990801315
pos_interaction	Firmness_interaction_PCP040696	0.9585248081724859
pos_interaction	Firmness_interaction_PCP000171	0.9585443087768455
pos_interaction	Firmness_interaction_PCP032535	0.9586905629283063
pos_interaction	Firmness_interaction_PCP016361	0.9588544611341279
pos_interaction	Firmness_interaction_PCP020898	0.9588580353215505
pos_interaction	Firmness_interaction_PCP018095	0.9589272605399735
pos_interaction	Firmness_interaction_PCP007999	0.958955372568957
pos_interaction	Firmness_interaction_PCP028381	0.9589983680933087
pos_interaction	Firmness_interaction_PCP017688	0.9592221911868427
pos_interaction	Firmness_interaction_PCP034358	0.9593167266223002
pos_interaction	Firmness_interaction_PCP016502	0.9594649412928958
pos_interaction	Firmness_interaction_PCP021784	0.9594689002925435
pos_interaction	Firmness_interaction_PCP012493	0.9595701279120149
pos_interaction	Firmness_interaction_PCP024364	0.959629353572458
pos_interaction	Firmness_interaction_PCP007168	0.9597791951959183
pos_interaction	Firmness_interaction_PCP016835	0.9601040274079459

pos_interaction	Firmness_interaction_PCP024818	0.9602524491166168
pos_interaction	Firmness_interaction_PCP012403	0.9602561230893216
pos_interaction	Firmness_interaction_PCP029022	0.9603832636048845
pos_interaction	Firmness_interaction_PCP036392	0.9603881584800557
pos_interaction	Firmness_interaction_PCP029386	0.960411894654011
pos_interaction	Firmness_interaction_PCP025204	0.960514070153254
pos_interaction	Firmness_interaction_PCP027273	0.9606050441495775
pos_interaction	Firmness_interaction_PCP028698	0.9608075972953463
pos_interaction	Firmness_interaction_PCP021749	0.9610584951992742
pos_interaction	Firmness_interaction_PCP025082	0.9610671553604634
pos_interaction	Firmness_interaction_PCP011485	0.9610786601426465
pos_interaction	Firmness_interaction_PCP020396	0.9612552452268782
pos_interaction	Firmness_interaction_PCP003910	0.9614330334471598
pos_interaction	Firmness_interaction_PCP012628	0.9616021883573515
pos_interaction	Firmness_interaction_PCP000535	0.9616039407355422
pos_interaction	Firmness_interaction_PCP030505	0.9619848231614143
pos_interaction	Firmness_interaction_PCP032254	0.9621818984384233
pos_interaction	Firmness_interaction_PCP012414	0.9623780347538878
pos_interaction	Firmness_interaction_PCP028011	0.9624054451636115
pos_interaction	Firmness_interaction_PCP041398	0.9625891551780482
pos_interaction	Firmness_interaction_PCP007333	0.9626134483180042
pos_interaction	Firmness_interaction_PCP020779	0.9626857915784768
pos_interaction	Firmness_interaction_PCP013589	0.962701792033716
pos_interaction	Firmness_interaction_PCP024844	0.9627110000265345
pos_interaction	Firmness_interaction_PCP011926	0.9628367267237481
pos_interaction	Firmness_interaction_PCP022356	0.9628453227167246
pos_interaction	Firmness_interaction_PCP000668	0.9629380635112932
pos_interaction	Firmness_interaction_PCP040285	0.9629929138051516
pos_interaction	Firmness_interaction_PCP007634	0.9630568679076725
pos_interaction	Firmness_interaction_PCP024457	0.9631253203661233
pos_interaction	Firmness_interaction_PCP024179	0.9631771065662437
pos_interaction	Firmness_interaction_PCP019466	0.9633524003493931
pos_interaction	Firmness_interaction_PCP010049	0.9634386858039273
pos_interaction	Firmness_interaction_PCP016799	0.9635211134637601
pos_interaction	Firmness_interaction_PCP008538	0.963634066271963
pos_interaction	Firmness_interaction_PCP007802	0.9636401819515017
pos_interaction	Firmness_interaction_PCP031506	0.9638329662749111
pos_interaction	Firmness_interaction_PCP000167	0.9638722533915441
pos_interaction	Firmness_interaction_PCP043243	0.9639543571624571
pos_interaction	Firmness_interaction_PCP032025	0.9642260464417549
pos_interaction	Firmness_interaction_PCP005562	0.9642709325314841
pos_interaction	Firmness_interaction_PCP042842	0.9642871865953995
pos_interaction	Firmness_interaction_PCP020926	0.9644206662144528
pos_interaction	Firmness_interaction_PCP024678	0.9644282099935102
pos_interaction	Firmness_interaction_PCP008486	0.9644811300866513
pos_interaction	Firmness_interaction_PCP007099	0.9646010289470278

pos_interaction	Firmness_interaction_PCP002857	0.9646272454117867
pos_interaction	Firmness_interaction_PCP019562	0.9646501673554957
pos_interaction	Firmness_interaction_PCP013462	0.9647632661716907
pos_interaction	Firmness_interaction_PCP009027	0.9647799991364654
pos_interaction	Firmness_interaction_PCP011471	0.964789309134043
pos_interaction	Firmness_interaction_PCP024427	0.9649044743948654
pos_interaction	Firmness_interaction_PCP025797	0.9649156014436976
pos_interaction	Firmness_interaction_PCP034151	0.9649703806891761
pos_interaction	Firmness_interaction_PCP030147	0.9649953944466683
pos_interaction	Firmness_interaction_PCP007048	0.9650358491417966
pos_interaction	Firmness_interaction_PCP039702	0.9650618684618748
pos_interaction	Firmness_interaction_PCP007754	0.9650741721440594
pos_interaction	Firmness_interaction_PCP013255	0.9650759783687731
pos_interaction	Firmness_interaction_PCP020355	0.965356405187545
pos_interaction	Firmness_interaction_PCP024628	0.9654986320633694
pos_interaction	Firmness_interaction_PCP003006	0.9655144280768518
pos_interaction	Firmness_interaction_PCP028178	0.9655950777147648
pos_interaction	Firmness_interaction_PCP019655	0.9656489394951893
pos_interaction	Firmness_interaction_PCP003894	0.9658887577586295
pos_interaction	Firmness_interaction_PCP012540	0.9660159260557251
pos_interaction	Firmness_interaction_PCP021057	0.966338228671843
pos_interaction	Firmness_interaction_PCP013053	0.9663679371437722
pos_interaction	Firmness_interaction_PCP015263	0.9665022135901749
pos_interaction	Firmness_interaction_PCP010055	0.9665293199909647
pos_interaction	Firmness_interaction_PCP032181	0.9666611220113819
pos_interaction	Firmness_interaction_PCP011513	0.9668277284679141
pos_interaction	Firmness_interaction_PCP026183	0.9669555186663595
pos_interaction	Firmness_interaction_PCP025854	0.9670045854902436
pos_interaction	Firmness_interaction_PCP035592	0.9670359979389446
pos_interaction	Firmness_interaction_PCP024649	0.9674589826796556
pos_interaction	Firmness_interaction_PCP026332	0.9675757632791445
pos_interaction	Firmness_interaction_PCP019416	0.9676050909307586
pos_interaction	Firmness_interaction_PCP008537	0.9676486782532764
pos_interaction	Firmness_interaction_PCP027619	0.9677977396383992
pos_interaction	Firmness_interaction_PCP032188	0.9678106756306969
pos_interaction	Firmness_interaction_PCP038435	0.9678225565075996
pos_interaction	Firmness_interaction_PCP039124	0.9678385095888087
pos_interaction	Firmness_interaction_PCP016724	0.9678586106797692
pos_interaction	Firmness_interaction_PCP012437	0.9678854003725479
pos_interaction	Firmness_interaction_PCP016072	0.9679354805386845
pos_interaction	Firmness_interaction_PCP028788	0.9681511197879843
pos_interaction	Firmness_interaction_PCP016065	0.9682207311710284
pos_interaction	Firmness_interaction_PCP030726	0.9683677859455572
pos_interaction	Firmness_interaction_PCP040594	0.9683884731849524
pos_interaction	Firmness_interaction_PCP000771	0.9684985450386255
pos_interaction	Firmness_interaction_PCP013360	0.9685278184720347

pos_interaction	Firmness_interaction_PCP028378	0.968589428168679
pos_interaction	Firmness_interaction_PCP012899	0.9686906033473964
pos_interaction	Firmness_interaction_PCP025645	0.968730297915072
pos_interaction	Firmness_interaction_PCP017994	0.9688036559202989
pos_interaction	Firmness_interaction_PCP039947	0.9688883706037889
pos_interaction	Firmness_interaction_PCP044694	0.9690981143821852
pos_interaction	Firmness_interaction_PCP028623	0.9693096939987194
pos_interaction	Firmness_interaction_PCP035757	0.969319992052756
pos_interaction	Firmness_interaction_PCP020421	0.969502238372106
pos_interaction	Firmness_interaction_PCP010847	0.9695733423741308
pos_interaction	Firmness_interaction_PCP017302	0.969610826323326
pos_interaction	Firmness_interaction_PCP018273	0.9696530858842791
pos_interaction	Firmness_interaction_PCP019649	0.9697430607870144
pos_interaction	Firmness_interaction_PCP017485	0.9698313419293054
pos_interaction	Firmness_interaction_PCP009808	0.9700886075206192
pos_interaction	Firmness_interaction_PCP008208	0.9702998925343608
pos_interaction	Firmness_interaction_PCP021693	0.9703689240471632
pos_interaction	Firmness_interaction_PCP019692	0.9706281267453976
pos_interaction	Firmness_interaction_PCP005531	0.9707381685476837
pos_interaction	Firmness_interaction_PCP023637	0.9707389573968752
pos_interaction	Firmness_interaction_PCP013684	0.9708037459219322
pos_interaction	Firmness_interaction_PCP014611	0.9708422196919102
pos_interaction	Firmness_interaction_PCP018057	0.9708490397421046
pos_interaction	Firmness_interaction_PCP010767	0.9710119792539132
pos_interaction	Firmness_interaction_PCP017660	0.9711824388673148
pos_interaction	Firmness_interaction_PCP028225	0.9712659288407536
pos_interaction	Firmness_interaction_PCP010261	0.9712748796376891
pos_interaction	Firmness_interaction_PCP012039	0.9713337771100865
pos_interaction	Firmness_interaction_PCP016755	0.9713375561405816
pos_interaction	Firmness_interaction_PCP033246	0.9717749917457593
pos_interaction	Firmness_interaction_PCP027769	0.9718839556543236
pos_interaction	Firmness_interaction_PCP024510	0.9718941603077885
pos_interaction	Firmness_interaction_PCP010074	0.9721354399248013
pos_interaction	Firmness_interaction_PCP027002	0.972148972692616
pos_interaction	Firmness_interaction_PCP040118	0.9722162769893331
pos_interaction	Firmness_interaction_PCP021810	0.9722720733264635
pos_interaction	Firmness_interaction_PCP005409	0.9727378828660213
pos_interaction	Firmness_interaction_PCP021875	0.972787082277896
pos_interaction	Firmness_interaction_PCP007189	0.9730354195679011
pos_interaction	Firmness_interaction_PCP023878	0.9732248699667412
pos_interaction	Firmness_interaction_PCP013622	0.9733051915342102
pos_interaction	Firmness_interaction_PCP039813	0.9734235718444559
pos_interaction	Firmness_interaction_PCP023151	0.9736585716277383
pos_interaction	Firmness_interaction_PCP028057	0.9737393255015389
pos_interaction	Firmness_interaction_PCP042186	0.9737504321593505
pos_interaction	Firmness_interaction_PCP023352	0.9739141107415112

pos_interaction	Firmness_interaction_PCP036703	0.9741006291341892
pos_interaction	Firmness_interaction_PCP031690	0.9741297374143163
pos_interaction	Firmness_interaction_PCP014886	0.974458736648902
pos_interaction	Firmness_interaction_PCP018708	0.9746099955083767
pos_interaction	Firmness_interaction_PCP006878	0.9746517744431611
pos_interaction	Firmness_interaction_PCP020062	0.9746520047576451
pos_interaction	Firmness_interaction_PCP025102	0.97469514036704
pos_interaction	Firmness_interaction_PCP031105	0.974796143818488
pos_interaction	Firmness_interaction_PCP017358	0.9749291826424128
pos_interaction	Firmness_interaction_PCP004206	0.9749402666727625
pos_interaction	Firmness_interaction_PCP004198	0.9749717071014639
pos_interaction	Firmness_interaction_PCP005413	0.9749893255591611
pos_interaction	Firmness_interaction_PCP007739	0.975383507167378
pos_interaction	Firmness_interaction_PCP007595	0.9753849327886563
pos_interaction	Firmness_interaction_PCP028751	0.9753878685750937
pos_interaction	Firmness_interaction_PCP006133	0.9754219308782589
pos_interaction	Firmness_interaction_PCP012075	0.9755458015853238
pos_interaction	Firmness_interaction_PCP026440	0.9755526489189172
pos_interaction	Firmness_interaction_PCP022615	0.9758261743320431
pos_interaction	Firmness_interaction_PCP026164	0.9758409842638224
pos_interaction	Firmness_interaction_PCP024976	0.9759105544718666
pos_interaction	Firmness_interaction_PCP016512	0.9759274065797615
pos_interaction	Firmness_interaction_PCP020841	0.976266166088413
pos_interaction	Firmness_interaction_PCP008851	0.9762979019178469
pos_interaction	Firmness_interaction_PCP033131	0.9764646156394854
pos_interaction	Firmness_interaction_PCP033152	0.9765156586690817
pos_interaction	Firmness_interaction_PCP010484	0.9765726620556148
pos_interaction	Firmness_interaction_PCP001467	0.9766364137082065
pos_interaction	Firmness_interaction_PCP017504	0.9766508345775379
pos_interaction	Firmness_interaction_PCP006791	0.9768014968575477
pos_interaction	Firmness_interaction_PCP023195	0.9769210108460687
pos_interaction	Firmness_interaction_PCP015489	0.9769271756045403
pos_interaction	Firmness_interaction_PCP001313	0.9770591516686582
pos_interaction	Firmness_interaction_PCP011734	0.9770720504154944
pos_interaction	Firmness_interaction_PCP030207	0.9771535623887093
pos_interaction	Firmness_interaction_PCP025793	0.9771560061516211
pos_interaction	Firmness_interaction_PCP023455	0.9772347025037973
pos_interaction	Firmness_interaction_PCP024242	0.9772404552342593
pos_interaction	Firmness_interaction_PCP032375	0.977268135125683
pos_interaction	Firmness_interaction_PCP034953	0.9775449292575122
pos_interaction	Firmness_interaction_PCP005274	0.9776333310368385
pos_interaction	Firmness_interaction_PCP015860	0.9776987084458212
pos_interaction	Firmness_interaction_PCP011760	0.9777224169572993
pos_interaction	Firmness_interaction_PCP009832	0.9777502224660164
pos_interaction	Firmness_interaction_PCP036209	0.9778109388734881
pos_interaction	Firmness_interaction_PCP001691	0.9778312750778182

pos_interaction	Firmness_interaction_PCP026039	0.977920821393814
pos_interaction	Firmness_interaction_PCP005855	0.9779640123918746
pos_interaction	Firmness_interaction_PCP044000	0.9780045509296573
pos_interaction	Firmness_interaction_PCP035017	0.978090552189366
pos_interaction	Firmness_interaction_PCP010025	0.9782557591392541
pos_interaction	Firmness_interaction_PCP002867	0.978384222536316
pos_interaction	Firmness_interaction_PCP005141	0.9783933006495843
pos_interaction	Firmness_interaction_PCP011867	0.978412960294434
pos_interaction	Firmness_interaction_PCP035228	0.9786359319923137
pos_interaction	Firmness_interaction_PCP039795	0.9787184611783453
pos_interaction	Firmness_interaction_PCP019752	0.9788910633237318
pos_interaction	Firmness_interaction_PCP006413	0.9789414930733523
pos_interaction	Firmness_interaction_PCP018093	0.9790309193272188
pos_interaction	Firmness_interaction_PCP026024	0.9791076161755737
pos_interaction	Firmness_interaction_PCP004369	0.97915122540583
pos_interaction	Firmness_interaction_PCP021612	0.9791853725545675
pos_interaction	Firmness_interaction_PCP023759	0.979199146564898
pos_interaction	Firmness_interaction_PCP015830	0.9792401454545713
pos_interaction	Firmness_interaction_PCP007629	0.979274541434036
pos_interaction	Firmness_interaction_PCP016914	0.9793391859307352
pos_interaction	Firmness_interaction_PCP023650	0.9794869552682142
pos_interaction	Firmness_interaction_PCP029939	0.9795203018051145
pos_interaction	Firmness_interaction_PCP007628	0.979557719119509
pos_interaction	Firmness_interaction_PCP027303	0.9796042796765054
pos_interaction	Firmness_interaction_PCP023098	0.9796584348168215
pos_interaction	Firmness_interaction_PCP019412	0.9797162607846586
pos_interaction	Firmness_interaction_PCP011417	0.979880280412906
pos_interaction	Firmness_interaction_PCP027431	0.979900363972099
pos_interaction	Firmness_interaction_PCP043652	0.9799324666951716
pos_interaction	Firmness_interaction_PCP005066	0.9802369478064582
pos_interaction	Firmness_interaction_PCP011698	0.9802784025506164
pos_interaction	Firmness_interaction_PCP042671	0.9803008286428097
pos_interaction	Firmness_interaction_PCP036488	0.9804155969422965
pos_interaction	Firmness_interaction_PCP009854	0.9804430270921568
pos_interaction	Firmness_interaction_PCP007847	0.9804922314337504
pos_interaction	Firmness_interaction_PCP014364	0.9804970810580705
pos_interaction	Firmness_interaction_PCP000117	0.9805884171728033
pos_interaction	Firmness_interaction_PCP027568	0.98064279536112
pos_interaction	Firmness_interaction_PCP004786	0.9806854016418471
pos_interaction	Firmness_interaction_PCP029231	0.9807434619275327
pos_interaction	Firmness_interaction_PCP026145	0.980809252794464
pos_interaction	Firmness_interaction_PCP015275	0.9808243171122937
pos_interaction	Firmness_interaction_PCP017906	0.9809899980928345
pos_interaction	Firmness_interaction_PCP013434	0.9810104542924768
pos_interaction	Firmness_interaction_PCP023102	0.9811072610023688
pos_interaction	Firmness_interaction_PCP030120	0.9811236424507013

pos_interaction	Firmness_interaction_PCP029445	0.9811718060498513
pos_interaction	Firmness_interaction_PCP026221	0.981432915641986
pos_interaction	Firmness_interaction_PCP032513	0.9817622761251529
pos_interaction	Firmness_interaction_PCP036007	0.9817644894141283
pos_interaction	Firmness_interaction_PCP027900	0.9818745566219382
pos_interaction	Firmness_interaction_PCP025125	0.9819011215602257
pos_interaction	Firmness_interaction_PCP018920	0.9819365180317049
pos_interaction	Firmness_interaction_PCP008956	0.9822284758157882
pos_interaction	Firmness_interaction_PCP001968	0.9822715412673516
pos_interaction	Firmness_interaction_PCP014204	0.9823443217651224
pos_interaction	Firmness_interaction_PCP026064	0.982359007796334
pos_interaction	Firmness_interaction_PCP008572	0.9823684957125547
pos_interaction	Firmness_interaction_PCP025146	0.982392923857808
pos_interaction	Firmness_interaction_PCP031008	0.9826580783857523
pos_interaction	Firmness_interaction_PCP003707	0.9826836663718189
pos_interaction	Firmness_interaction_PCP040467	0.9828085434871915
pos_interaction	Firmness_interaction_PCP013964	0.982861386317872
pos_interaction	Firmness_interaction_PCP019427	0.9828679874257666
pos_interaction	Firmness_interaction_PCP040042	0.9829114893695254
pos_interaction	Firmness_interaction_PCP015739	0.9829516992070796
pos_interaction	Firmness_interaction_PCP017752	0.9830688223071423
pos_interaction	Firmness_interaction_PCP036761	0.9831266505012604
pos_interaction	Firmness_interaction_PCP025528	0.9831333853901764
pos_interaction	Firmness_interaction_PCP013616	0.9832620306448777
pos_interaction	Firmness_interaction_PCP040769	0.9833205404274731
pos_interaction	Firmness_interaction_PCP025052	0.9834076130711549
pos_interaction	Firmness_interaction_PCP029443	0.9835772817967128
pos_interaction	Firmness_interaction_PCP001279	0.9835933223930735
pos_interaction	Firmness_interaction_PCP027594	0.9836544930871565
pos_interaction	Firmness_interaction_PCP009402	0.9838694452050474
pos_interaction	Firmness_interaction_PCP023176	0.9840202264528305
pos_interaction	Firmness_interaction_PCP026580	0.984040515935136
pos_interaction	Firmness_interaction_PCP012439	0.9841278765843035
pos_interaction	Firmness_interaction_PCP010455	0.984130813531318
pos_interaction	Firmness_interaction_PCP015752	0.9841881289979234
pos_interaction	Firmness_interaction_PCP030782	0.9842201976229236
pos_interaction	Firmness_interaction_PCP007240	0.9843957608969081
pos_interaction	Firmness_interaction_PCP031379	0.9844601074514729
pos_interaction	Firmness_interaction_PCP032878	0.9844930353322121
pos_interaction	Firmness_interaction_PCP018452	0.9845538342260399
pos_interaction	Firmness_interaction_PCP010597	0.9845987354903825
pos_interaction	Firmness_interaction_PCP014093	0.9847074854669808
pos_interaction	Firmness_interaction_PCP001219	0.9851916413019335
pos_interaction	Firmness_interaction_PCP003341	0.9852027680534783
pos_interaction	Firmness_interaction_PCP002905	0.985332604191773
pos_interaction	Firmness_interaction_PCP014251	0.9854177613041161

pos_interaction	Firmness_interaction_PCP031705	0.9854252831642704
pos_interaction	Firmness_interaction_PCP029669	0.9855359580945061
pos_interaction	Firmness_interaction_PCP003566	0.9855403366169482
pos_interaction	Firmness_interaction_PCP008041	0.9856824017927432
pos_interaction	Firmness_interaction_PCP001961	0.9857700330993444
pos_interaction	Firmness_interaction_PCP008611	0.9857792927213781
pos_interaction	Firmness_interaction_PCP009532	0.9857805058158906
pos_interaction	Firmness_interaction_PCP017342	0.9858273812758617
pos_interaction	Firmness_interaction_PCP012254	0.9859254027855412
pos_interaction	Firmness_interaction_PCP025596	0.9859935795955084
pos_interaction	Firmness_interaction_PCP028380	0.9860080651042227
pos_interaction	Firmness_interaction_PCP005202	0.9861228346459894
pos_interaction	Firmness_interaction_PCP033179	0.9862086050537827
pos_interaction	Firmness_interaction_PCP002933	0.9865199580892257
pos_interaction	Firmness_interaction_PCP017315	0.9865880999136545
pos_interaction	Firmness_interaction_PCP036084	0.9866753074159105
pos_interaction	Firmness_interaction_PCP022634	0.9869207745813353
pos_interaction	Firmness_interaction_PCP028332	0.987000909033789
pos_interaction	Firmness_interaction_PCP018119	0.9870440552209252
pos_interaction	Firmness_interaction_PCP012001	0.987157194193351
pos_interaction	Firmness_interaction_PCP030309	0.9871677323560648
pos_interaction	Firmness_interaction_PCP012388	0.9872034064065266
pos_interaction	Firmness_interaction_PCP044677	0.9872182913532349
pos_interaction	Firmness_interaction_PCP007489	0.9872371944380558
pos_interaction	Firmness_interaction_PCP005035	0.9872622133717597
pos_interaction	Firmness_interaction_PCP043963	0.9872809990862064
pos_interaction	Firmness_interaction_PCP042050	0.9873220196447319
pos_interaction	Firmness_interaction_PCP031273	0.9873782433308581
pos_interaction	Firmness_interaction_PCP004639	0.9873879218551662
pos_interaction	Firmness_interaction_PCP011597	0.9874817732783185
pos_interaction	Firmness_interaction_PCP022859	0.9876139446682632
pos_interaction	Firmness_interaction_PCP011275	0.9876212568806397
pos_interaction	Firmness_interaction_PCP001150	0.9876355798397071
pos_interaction	Firmness_interaction_PCP000531	0.9877005127331633
pos_interaction	Firmness_interaction_PCP015476	0.9877547720685355
pos_interaction	Firmness_interaction_PCP020570	0.9878123451853935
pos_interaction	Firmness_interaction_PCP021687	0.9878129547449354
pos_interaction	Firmness_interaction_PCP011929	0.9878889748529073
pos_interaction	Firmness_interaction_PCP043409	0.9879362498613031
pos_interaction	Firmness_interaction_PCP008306	0.9880741131276453
pos_interaction	Firmness_interaction_PCP025980	0.9881972798565009
pos_interaction	Firmness_interaction_PCP044442	0.9882128071819596
pos_interaction	Firmness_interaction_PCP030424	0.9882391490252644
pos_interaction	Firmness_interaction_PCP033836	0.9882687372216972
pos_interaction	Firmness_interaction_PCP010238	0.9882699207799343
pos_interaction	Firmness_interaction_PCP042778	0.9883894649154282

pos_interaction	Firmness_interaction_PCP002706	0.9884098848425293
pos_interaction	Firmness_interaction_PCP006961	0.9884638482203252
pos_interaction	Firmness_interaction_PCP003151	0.9885096502469213
pos_interaction	Firmness_interaction_PCP040050	0.9885130145424225
pos_interaction	Firmness_interaction_PCP027641	0.9885706055505757
pos_interaction	Firmness_interaction_PCP002307	0.9886541132523867
pos_interaction	Firmness_interaction_PCP028644	0.9886764551135169
pos_interaction	Firmness_interaction_PCP044438	0.9887677415931724
pos_interaction	Firmness_interaction_PCP007969	0.9890864932706825
pos_interaction	Firmness_interaction_PCP001059	0.9891098825364056
pos_interaction	Firmness_interaction_PCP023530	0.9891148999906638
pos_interaction	Firmness_interaction_PCP031536	0.989239870899502
pos_interaction	Firmness_interaction_PCP017494	0.98926960868455
pos_interaction	Firmness_interaction_PCP031530	0.9892918272298339
pos_interaction	Firmness_interaction_PCP007510	0.9894072314047385
pos_interaction	Firmness_interaction_PCP017009	0.9895045595224814
pos_interaction	Firmness_interaction_PCP007034	0.9895200921351717
pos_interaction	Firmness_interaction_PCP043663	0.9897903468412843
pos_interaction	Firmness_interaction_PCP003557	0.9899021786697104
pos_interaction	Firmness_interaction_PCP002525	0.989911439986627
pos_interaction	Firmness_interaction_PCP011385	0.9899668467458405
pos_interaction	Firmness_interaction_PCP040256	0.9899914395486688
pos_interaction	Firmness_interaction_PCP004938	0.9900104689710408
pos_interaction	Firmness_interaction_PCP013388	0.9900588945829073
pos_interaction	Firmness_interaction_PCP008393	0.9902070799171256
pos_interaction	Firmness_interaction_PCP023243	0.9902988209388285
pos_interaction	Firmness_interaction_PCP002432	0.9903637368458678
pos_interaction	Firmness_interaction_PCP030697	0.9903825149524359
pos_interaction	Firmness_interaction_PCP028844	0.9905027722695099
pos_interaction	Firmness_interaction_PCP018019	0.9905295833286869
pos_interaction	Firmness_interaction_PCP009299	0.9905415286164151
pos_interaction	Firmness_interaction_PCP002406	0.9906298809181748
pos_interaction	Firmness_interaction_PCP000387	0.9906343725880462
pos_interaction	Firmness_interaction_PCP000924	0.9908604227606785
pos_interaction	Firmness_interaction_PCP029024	0.9908607004181461
pos_interaction	Firmness_interaction_PCP024206	0.9909189691352114
pos_interaction	Firmness_interaction_PCP030855	0.9909208048460291
pos_interaction	Firmness_interaction_PCP008307	0.990939678128544
pos_interaction	Firmness_interaction_PCP007868	0.991273212616154
pos_interaction	Firmness_interaction_PCP027202	0.9913354074232252
pos_interaction	Firmness_interaction_PCP012204	0.9915186731734558
pos_interaction	Firmness_interaction_PCP018944	0.9916686870791176
pos_interaction	Firmness_interaction_PCP040802	0.9917721758380016
pos_interaction	Firmness_interaction_PCP043114	0.99191126970965
pos_interaction	Firmness_interaction_PCP002044	0.9920865143661431
pos_interaction	Firmness_interaction_PCP021477	0.9920931409019479

pos_interaction	Firmness_interaction_PCP000287	0.9921363071115795
pos_interaction	Firmness_interaction_PCP000429	0.9921761659513257
pos_interaction	Firmness_interaction_PCP023554	0.9922182862909416
pos_interaction	Firmness_interaction_PCP002165	0.9922953172009681
pos_interaction	Firmness_interaction_PCP019537	0.9923819816804005
pos_interaction	Firmness_interaction_PCP006656	0.9924303096176187
pos_interaction	Firmness_interaction_PCP036833	0.9924939764629486
pos_interaction	Firmness_interaction_PCP029128	0.9925866183045451
pos_interaction	Firmness_interaction_PCP003499	0.9926321150923172
pos_interaction	Firmness_interaction_PCP024366	0.9926786560647177
pos_interaction	Firmness_interaction_PCP012937	0.9927170664787299
pos_interaction	Firmness_interaction_PCP036199	0.9927221851568592
pos_interaction	Firmness_interaction_PCP016390	0.9930122882638063
pos_interaction	Firmness_interaction_PCP037899	0.9930336119970252
pos_interaction	Firmness_interaction_PCP006414	0.9930588282033438
pos_interaction	Firmness_interaction_PCP028442	0.9930971056592091
pos_interaction	Firmness_interaction_PCP009169	0.9931570814139618
pos_interaction	Firmness_interaction_PCP020651	0.9932025516451608
pos_interaction	Firmness_interaction_PCP025929	0.9934096691518942
pos_interaction	Firmness_interaction_PCP007917	0.9935145304180812
pos_interaction	Firmness_interaction_PCP003322	0.993542701186548
pos_interaction	Firmness_interaction_PCP013154	0.9936020075248384
pos_interaction	Firmness_interaction_PCP025097	0.9937262678881355
pos_interaction	Firmness_interaction_PCP016781	0.9937309467676687
pos_interaction	Firmness_interaction_PCP041476	0.9937754741998704
pos_interaction	Firmness_interaction_PCP032497	0.993784071226259
pos_interaction	Firmness_interaction_PCP029513	0.9938271541978674
pos_interaction	Firmness_interaction_PCP000331	0.9938714595610589
pos_interaction	Firmness_interaction_PCP030272	0.9939252747578123
pos_interaction	Firmness_interaction_PCP006880	0.9942106208007149
pos_interaction	Firmness_interaction_PCP004971	0.9942552976003315
pos_interaction	Firmness_interaction_PCP026875	0.9942611323144257
pos_interaction	Firmness_interaction_PCP024932	0.9942943379532412
pos_interaction	Firmness_interaction_PCP008655	0.9943247663503035
pos_interaction	Firmness_interaction_PCP003533	0.9944208086838253
pos_interaction	Firmness_interaction_PCP018847	0.9946385254041477
pos_interaction	Firmness_interaction_PCP018614	0.9946889433147246
pos_interaction	Firmness_interaction_PCP029690	0.9948921141455344
pos_interaction	Firmness_interaction_PCP016152	0.9949147947274285
pos_interaction	Firmness_interaction_PCP009020	0.9949296087729601
pos_interaction	Firmness_interaction_PCP003459	0.9949911270452426
pos_interaction	Firmness_interaction_PCP028794	0.9951060826414436
pos_interaction	Firmness_interaction_PCP008973	0.9951410146665808
pos_interaction	Firmness_interaction_PCP009782	0.9952674882740382
pos_interaction	Firmness_interaction_PCP026694	0.9953807630313408
pos_interaction	Firmness_interaction_PCP011013	0.9954942263411707

pos_interaction	Firmness_interaction_PCP011689	0.9955172687971293
pos_interaction	Firmness_interaction_PCP013937	0.9955784105654963
pos_interaction	Firmness_interaction_PCP019013	0.9957726165446238
pos_interaction	Firmness_interaction_PCP013897	0.9957946054580458
pos_interaction	Firmness_interaction_PCP030646	0.9960886380799461
pos_interaction	Firmness_interaction_PCP010035	0.9961213333156431
pos_interaction	Firmness_interaction_PCP022982	0.9961691658152906
pos_interaction	Firmness_interaction_PCP005572	0.9961802734260802
pos_interaction	Firmness_interaction_PCP015273	0.9962525049961539
pos_interaction	Firmness_interaction_PCP000523	0.9963063109276805
pos_interaction	Firmness_interaction_PCP024317	0.996404632799667
pos_interaction	Firmness_interaction_PCP009961	0.9964723862728854
pos_interaction	Firmness_interaction_PCP024226	0.9965344438172086
pos_interaction	Firmness_interaction_PCP021016	0.9965865087635233
pos_interaction	Firmness_interaction_PCP014299	0.9966497930497679
pos_interaction	Firmness_interaction_PCP022670	0.9967905332633553
pos_interaction	Firmness_interaction_PCP041284	0.996794249838243
pos_interaction	Firmness_interaction_PCP044309	0.9969329143394349
pos_interaction	Firmness_interaction_PCP024261	0.996963404338055
pos_interaction	Firmness_interaction_PCP035412	0.9969723437661157
pos_interaction	Firmness_interaction_PCP005727	0.9969818069553421
pos_interaction	Firmness_interaction_PCP022849	0.9970935314787813
pos_interaction	Firmness_interaction_PCP011950	0.997310274379242
pos_interaction	Firmness_interaction_PCP020253	0.9973744729977077
pos_interaction	Firmness_interaction_PCP021315	0.9974229334352125
pos_interaction	Firmness_interaction_PCP012976	0.9974864318653972
pos_interaction	Firmness_interaction_PCP044141	0.9974935033443404
pos_interaction	Firmness_interaction_PCP007160	0.9978302567911616
pos_interaction	Firmness_interaction_PCP009922	0.9980822875863689
pos_interaction	Firmness_interaction_PCP018084	0.9982635255592518
pos_interaction	Firmness_interaction_PCP007096	0.9982681280622898
pos_interaction	Firmness_interaction_PCP000385	0.9983537986100609
pos_interaction	Firmness_interaction_PCP022315	0.9983807669358585
pos_interaction	Firmness_interaction_PCP019046	0.9985327422658113
pos_interaction	Firmness_interaction_PCP031591	0.9986254337257576
pos_interaction	Firmness_interaction_PCP029075	0.9987623310460275
pos_interaction	Firmness_interaction_PCP016236	0.9988055934485602
pos_interaction	Firmness_interaction_PCP045037	0.9988335139689042
pos_interaction	Firmness_interaction_PCP013757	0.9988354255578434
pos_interaction	Firmness_interaction_PCP010943	0.9988451062794323
pos_interaction	Firmness_interaction_PCP011931	0.9988837752328219
pos_interaction	Firmness_interaction_PCP009121	0.9988849734846125
pos_interaction	Firmness_interaction_PCP023293	0.9989140210095316
pos_interaction	Firmness_interaction_PCP016401	0.9989718343368682
pos_interaction	Firmness_interaction_PCP020578	0.9991264725811461
pos_interaction	Firmness_interaction_PCP044687	0.9991862922843678

pos_interaction	Firmness_interaction_PCP001807	0.9992065278102938
pos_interaction	Firmness_interaction_PCP004748	0.9992534764199578
pos_interaction	Firmness_interaction_PCP017056	0.999317660458235
pos_interaction	Firmness_interaction_PCP009791	0.9993424559205382
pos_interaction	Firmness_interaction_PCP041054	0.9993836594829353
pos_interaction	Firmness_interaction_PCP000690	0.9994083322049064
pos_interaction	Firmness_interaction_PCP043122	0.999412180816026
pos_interaction	Firmness_interaction_PCP032339	0.999414218701544
pos_interaction	Firmness_interaction_PCP022826	0.9994214642871279
pos_interaction	Firmness_interaction_PCP021997	0.999481555945986
pos_interaction	Firmness_interaction_PCP026394	0.9994925483092055
pos_interaction	Firmness_interaction_PCP015694	0.9994985152299602
pos_interaction	Firmness_interaction_PCP002370	0.999763756695111
pos_interaction	Firmness_interaction_PCP012560	0.9998278905952158
pos_interaction	Firmness_interaction_PCP018315	0.9998671815904856
pos_interaction	Firmness_interaction_PCP012359	0.9999244611998958
pos_interaction	Firmness_interaction_PCP016932	0.9999251785550011

Supplementary Table S5

primer name	primer sequence (5'-3')	target gene
Pc_ACS_for2	ATGCTGGCTTGTTCTGTTGG	PCP011500
Pc_ACS_rev2	AGGTTCCGTGCAATGACAAG	
Pc_ACO_for1	AAGGTCAGCAACTACCCTCC	PCP011683
Pc_ACO_rev1	TGTCATCCTGGAAGAGCAGG	
Pc_ERS1_for2	TGAAGTTCACAAAGCAGGGC	PCP004450
Pc_ERS1_rev2	CGTAGGTAGAAGTGGCCCTC	
Pc_ERS2_for1	TGACTGTTGCCAAGGTTTCG	PCP026971
Pc_ERS2_rev1	GACAGCTCCCGTTTCTTGAC	
Pc_ERF1_for2	AACATTCGAAACGGCGGAAG	PCP015040
Pc_ERF1_rev2	CGAGGACTGAGACGCATTTG	
Pc_ERF2_for1	TGGCTTCACTCCAGATGACC	PCP002056
Pc_ERF2_rev1	ATATCCGGCATTTTCGCACC	
Pc_IAA_for2	AAACCAGTTGAAACCAGCGG	PCP003908
Pc_IAA_rev2	CGGAAGAGTTCATCGACAGC	
Pc_PG1_for2	GGAGTGTGTGTGAATGGAGC	PCP006771
Pc_PG1_rev2	TCTGGTCGATGATGATGGGG	
Pc_LOX1_for1	CTTCAACGGAGAATCAGGCG	PCP002320
Pc_LOX1_rev1	TCGGTTATGTCATCGAGGGG	
Pc_ADH2_for1	GTTTGTTCACCTAAGCCGGG	PCP031262
Pc_ADH2_rev1	TAGCAACCCGACAATTTGGC	
Pc_AAT2_for2	AGAGGCGCAGATGCACCATC	PCP024687
Pc_AAT2_rev2	GTTACTGAATGCGTATGAGCCATC	
Pc_HPL2_for2	CTCTCTCAACCACAACCACAACC	PCP012365
Pc_HPL2_rev2	CTCGGTGCGCTTCTTGAAG	
Md_8283_for	CTCGTCGTCTTGTTCCCTGA	PCP030439
Md_8283_rev	GCCTAAGGACAGGTGGTCTATG	
